# Hypoxia and low-glucose environments co-induced HGDILnc1 promote glycolysis and angiogenesis

**DOI:** 10.1038/s41420-024-01903-w

**Published:** 2024-03-12

**Authors:** Qing-Wei Zhang, Xiao-Lu Lin, Zi-Hao Dai, Ran Zhao, Yi-Chao Hou, Qian Liang, Yan Zhang, Zhi-Zheng Ge

**Affiliations:** 1 Division of Gastroenterology and Hepatology, NHC Key Laboratory of Gastroenterology and Hepatology, Ministry of Health, Renji Hospital, School of Medicine, Shanghai Jiao Tong University, Shanghai Institute of Digestive Disease, Shanghai, China; 2 Department of Digestive Endoscopy Center, Fujian Provincial Hospital, Shengli Clinical Medical College of Fujian Medical University, Fuzhou, Fujian, China; 3 Department of Gastroenterology, Guangdong Provincial People’s Hospital, Guangdong Academy of Medical Sciences, Guangzhou, China; 4 Department of Gastroenterology, Shanghai Nineth People’s Hospital, School of Medicine, Shanghai Jiao Tong University, Shanghai, China; 5 Department of Gastroenterology, Tongji Institute of Digestive Diseases, Tongji Hospital, School of Medicine, Tongji University, Shanghai, China

**Keywords:** Translational research, Pathogenesis

## Abstract

Small bowel vascular malformation disease (SBVM) commonly causes obscure gastrointestinal bleeding (OGIB). However, the pathogenetic mechanism and the role of lncRNAs in SBVM remain largely unknown. Here, we found that hypoxia and low-glucose environments co-augment angiogenesis and existed in SBVM. Mechanistically, hypoxia and low-glucose environments supported angiogenesis via activation of hypoxia and glucose deprivation-induced lncRNA (HGDILnc1) transcription by increasing binding of the NeuroD1 transcription factor to the HGDILnc1 promoter. Raised HGDILnc1 acted as a suppressor of α-Enolase 1 (ENO1) small ubiquitin-like modifier modification (SUMOylation)-triggered ubiquitination, and an activator of transcription of Aldolase C (ALDOC) via upregulation of Histone H2B lysine 16 acetylation (H2BK16ac) level in the promoter of ALDOC, and consequently promoting glycolysis and angiogenesis. Moreover, HGDILnc1 was clinically positively correlated with Neurogenic differentiation 1 (NeuroD1), ENO1, and ALDOC in SBVM tissues, and could function as a biomarker for SBVM diagnosis and therapy. These findings suggest that hypoxia and low-glucose environments were present in SBVM tissues, and co-augmented angiogenesis. Hypoxia and low-glucose environments co-induced HGDILnc1, which is higher expressed in SBVM tissue compared with normal tissue, could promoted glycolysis and angiogenesis.

## Introduction

Small bowel vascular malformation (SBVM) is a frequent reason for obscure gastrointestinal bleeding (OGIB) [1]. There is an association between SBVM and aberrant angiogenesis [1, 2]. Other factors, including hypoxia, aging, and biochemical factors, may also be responsible [3–5]. Chronic intermittent obstruction of vessels, resulting in mucosal ischemia and hypoxia, has also been suggested [6, 7]. It is suggested that increased bowel wall pressures and chronic hypoxia can induce the partial obstruction of submucosal veins, leading to capillary congestion, failure of the pre-capillary sphincters, and eventually the formation of permanent angioectasia [6, 7]. Supposing capillary congestion existed in the SBVM, both hypoxic and low-glucose environments may, thus, be present in SBVM. It was reported that Solute Carrier Family 2, Facilitated Glucose Transporter Member 1 (SLC2A1) was the most upregulated protein in cells with low-glucose environment. Cells with high SLC2A1 expression showed the enhanced glucose uptake and glycolysis and survived in low-glucose conditions [8]. Results showed SLC2A1 was up-regulated in the low-glucose environments, which was a marker of low-glucose environments [8, 9]. Meanwhile, hypoxia-inducible factor 1-alpha (HIF-1α) have also been validated as a marker of hypoxia [10]. Nevertheless, the underlying pathogenetic mechanism remains unknown.

Long non-coding RNAs (lncRNAs) are a class of non-coding transcripts >200 nucleotides in length. Recent studies have revealed that lncRNAs may affect angiogenesis [11, 12]. For example, lncRNAs, LncEGFL7OS, STEEL, and GATA6-AS may play a role in angiogenesis [11–13]. In addition, lncRNAs have been identified as important players in the regulation of the glycolysis [14, 15] which have been proved to supply up to 85% of the total cellular ATP content for endothelial cells [16]. However, However, no study has ever investigated the role of lncRNAs in SBVM development, glycolysis induced angiogenesis, which need to be further teased out.

Glycolysis is a compensatory process of energy metabolism during hypoxia, which is a common pathological condition contributing to diverse diseases like cancer and neovascularization [16]. α-Enolase 1 (ENO1) and Aldolase C (ALDOC) is a critical glycolytic enzyme whose aberrant expression drives the pathogenesis of neovascularization [17]. ENO1 belongs to the enolase family that contains three distinct isoforms, alpha- or non-neuronal enolase, beta- or muscle-specific enolase and gamma- or neuron-specific enolase and ENO1 is ubiquitously expressed in most human tissues [18]. The aldolase family is the fourth enzyme involved in glycolysis and contains ALDOA, ALDOB and ALDOC, which are expressed in different human organs [19]. However, the detailed mechanism of glycolysis including ENO1 and ALODC in SBVM development.

Small ubiquitin-like modifier modification (SUMOylation) is a posttranslational modification where a small ubiquitin-like modifier (SUMO) is reversibly attached to lysine residues in the target protein [20]. SUMOylation modulates a variety of processes, including protein and genome stability, transport, mRNA functions, and cell growth [21–24].

Here, we demonstrated that hypoxia and a low-glucose environment together enhance angiogenesis, resulting in SBVM. We simulated these conditions and identified candidate lncRNAs, showing that lncRNA (MSTRG.6431.1) was linked to SBVM development. This lncRNA was, therefore, termed the hypoxia and glucose deprivation-induced lncRNA (HGDILnc1).

Mechanistically, hypoxia and glucose deprivation co-induced HGDILnc1 visa transcription factor Neurogenic differentiation 1 (NeuroD1) was observed to promote angiogenesis by suppressing ENO1 SUMOylation-triggered ubiquitination and the activation of ALDOC transcription. Thus, our findings show a critical pro-angiogenesis role of the NeuroD1/HGDILnc1/ENO1/ALDOC axis in SBVM development.

## Results

### Hypoxia and low-glucose environment co-induce SBVM

**Fig. 1:**
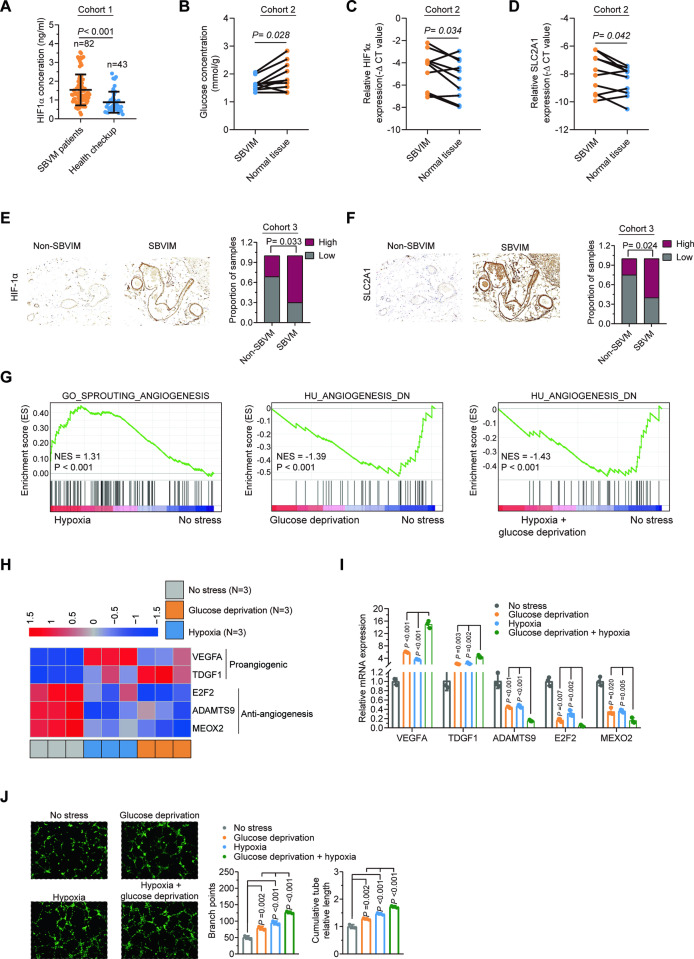
Hypoxia and low-glucose environment co-induced SBVM. **A** HIF-1α expression in the serum of non-SBVM (*n* = 42) and SBVM patients (*n* = 82) in cohort 1. **B** Glucose concentration in SBVM tissues and paired adjacent normal tissues (*n* = 10) measured by colorimetric analysis in cohort 2. **C** HIF-1α expression levels in SBVM and paired adjacent normal tissues (*n* = 10) measured by using qRT–PCR in cohort 2. **D** SLC2A1 expression levels in SBVM tissues and paired adjacent normal tissues (*n* = 10) measured by using qRT–PCR in cohort 2. **E** Representative immunohistochemical images (left) of HIF-1α in SBVM and non-SBVM tissues using IHC and proportions of high and low staining (right) in cohort 3. Scale bars: 100 μm. **F** IHC images (left) of SLC2A1 and analysis of staining levels in SBVM tissues and non-SBVM tissues (right) in cohort 3. Scale bars: 100 μm. **G** GSEA identified angiogenesis-related genes between HUVECs with hypoxia and HUVEC with controls. **H** Heatmap showing angiogenesis-related genes in cells with glucose deprivation, hypoxia, and controls for RNA microarray. **I** mRNA expression of angiogenesis-related genes in HUVEC cells measured by qRT–PCR after glucose deprivation, hypoxia, or glucose deprivation with hypoxia treatment. **J** Capillary tube formation for evaluation of angiogenesis in HUVECs after glucose deprivation, hypoxia, or glucose deprivation with hypoxia treatment. Scale bars: 100 μm.

HIF-1α levels were found to be higher in SBVM sera (Fig. 1A) in cohort 1, indicating the presence of a hypoxic environment, as previously noted [4, 25]. Glucose concentrations were lower in SBVM tissue (Fig. 1B) compared to the paired adjacent normal tissues in the cohort 2, indicating the presence of a low-glucose environment. It has been reported HIF-1α is a biomarker for hypoxia [10] and that SLC2A1 is upregulated in the low-glucose environment [8, 9]. qRT-PCR showed elevated mRNA levels of HIF-1α and SLC2A1 in SBVM tissue (Fig. 1C, D) compared to the paired adjacent normal tissues in cohort 2. These results were confirmed by immunohistochemical (IHC) staining for HIF-1α (Fig. 1E) and SLC2A1 (Fig. 1F) in SBVM vessels compared to non-SBVM tissue (Fig. S1A) in cohort 3. Collectively, the data strongly suggest that hypoxia and low glucose co-existed in the SBVM tissues.

To investigate the combined effects of hypoxia and low-glucose environments on angiogenesis, we first performed two microarrays to profile mRNA expression in Human Umbilical Vein Endothelial Cells (HUVEC) cells with hypoxia, glucose deprivation, or no stress treatment using the “Agilent Human lncRNA Microarray 2019” (4*180k, Design ID:086188). This identified 402 upregulated and 197 downregulated genes (over twofold) after hypoxia treatment (Table S1) and 386 upregulated and 245 downregulated genes after glucose deprivation (Table S2 and Fig. S1B). GSEA showed the gene sets related to angiogenesis correlated with hypoxia (“GO_SPROUTING_ANGIOGENESIS”, Fig. 1G), glucose deprivation (“HU_ANGIOGENESIS_DN”, Fig. 1G), and hypoxia treatment or glucose deprivation treatment (“HU_ANGIOGENESIS_DN”, Fig. 1G). Some pro-angiogenic genes were augmented while anti-angiogenic genes were reduced after hypoxia or glucose deprivation treatment (Fig. 1H). The findings were verified by qRT-PCR (Fig. 1I). In addition, the co-treatment had a stronger effect on the expression of these pro-angiogenic and anti-angiogenic genes than the single treatments (Fig. 1I). In vitro capillary tube formation assay also showed that either treatment promoted angiogenesis, as well as co-treatment had a stronger effect than the single treatments (Fig. 1J). To test whether hypoxic and low-glucose environment enhance each other to induce angiogenesis, we first treated HUVEC with hypoxia and found SLC2A1 was significantly increased (Fig. S1C) and relative glucose level in cell medium was significantly decreased (Fig. S1D) after hypoxic treatment. Also, HIF-1α, marker of hypoxia, was significantly increased after glucose deprivation (Fig. S1E). Thus, hypoxia and low-glucose environments co-augment angiogenesis.

### Glucose deprivation and hypoxia co-induce HGDILnc1-promoted angiogenesis both in vitro and in vivo

**Fig. 2:**
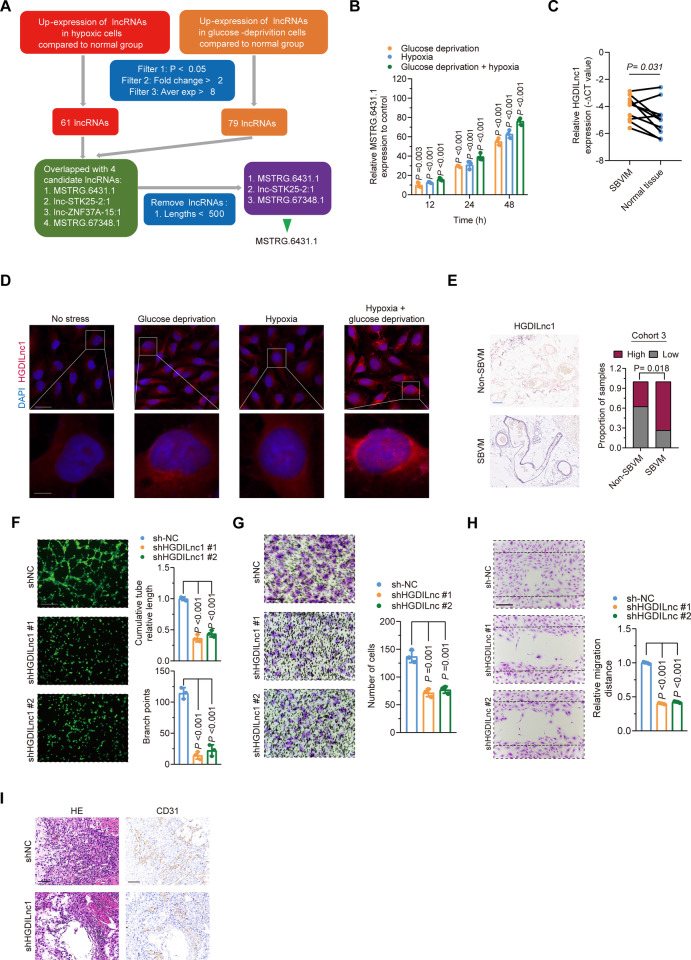
Glucose deprivation and hypoxia co-induced HGDILnc1 to promote angiogenesis. **A** Flowchart for selection of candidate lncRNAs from upregulated lncRNAs in glucose-deprived and hypoxic HUVEC cells. **B** MSTRG.6431.1 levels in HUVEC cell line measured by qRT–PCR after glucose deprivation, hypoxia, or glucose deprivation with hypoxia treatment for 12, 24, and 48 h. **C** HGDILnc1 levels in SBVM and paired adjacent tissues measured by qRT–PCR. **D** FISH of HGDILnc1 (red) in HUVEC cells after glucose deprivation, hypoxia, or glucose deprivation with hypoxia. Nuclei are seen in blue (DAPI). Scale bars, up: 20 μm; down: 5 μm. **E** Representative ISH images (left) of HGDIlnc1 in SBVM and non-SBVM tissues, and analysis (right) of high and low staining in SBVM and non-SBVM tissues in cohort 3. Scale bars: 100 μm. **F** Capillary tube formation for determining angiogenesis in HGDILnc1-silenced HUVECs. Scale bars: 100 μm. **G** Transwell assays for evaluating migration in HGDILnc1-silenced HUVECs. Scale bars: 100 μm. **H** Wound-healing assay for evaluating migration in HGDILnc1-silenced HUVECs of HGDILnc1. Scale bars: 100 μm. **I** H&E-stained 10 µm paraffin sections and CD31-labeled sections of the Matrigel plugs after HGDILnc1 silencing in an in vivo Matrigel implantation model. Scale bars: 100 μm.

lncRNAs are implicated in angiogenesis [26, 27]. A microarray of lncRNA expression in HUVEC cells identified 6812 differently expressed lncRNAs after glucose deprivation and 4451 after hypoxia treatment (Fig. S2A and Tables S3 and S4). Of these, we selected 61 up-regulated lncRNAs from the hypoxia group and 79 from the low-glucose group (*P* < 0.05, Fold change >2, Average expression >8, Fig. 2A). Using more stringent criteria (lncRNA length >500 bp, distinct from other coding transcripts, Fig. 2A) identified three candidate lncRNAs, namely MSTRG.6431.1, lnc-STK25-2:1, and MSTRG.67348.1. All three were significantly overexpressed in hypoxic or glucose deprivation HUVEC cells and might regulate angiogenesis as well.

Verification of the microarray data demonstrated significant elevation of the levels of two of the three lncRNAs after hypoxia with glucose deprivation (Figs. 2B and S2B). Comparison of the expression of these lncRNAs in 10 SBVM and paired adjacent normal tissues indicated that only MSTRG.6431.1 was raised in SBVM (Figs. 2C and S2C). We, therefore, focused on MSTRG.6431.1, and named it “hypoxia and glucose deprivation-induced lncRNA” (HGDILnc1). The PCR results were confirmed by FISH (Fig. 2D) and further in situ hybridization (ISH) assessment of 30 SBVM and 16 non-SBVM tissues confirmed the elevated levels of HGDILnc1 in SBVM (Fig. 2E) in cohort 3.

Sequencing of HGDILnc1 using 5′ and 3′ rapid amplification of cDNA ends (RACE)-PCR (Fig. S2D) to identify the 5’ and 3’ ends and the transcription start site (TSS). Sequencing indicated the boundary between HGDILnc1 and the universal anchor primer (Fig. S2D). The low probability of HGDILnc1 encoding a protein was determined using the CAPT, CPC2.0, and PORTRAIT databases (Fig. S2E). RT-PCR of nuclear and cytoplasmic cell fractions showed that HGDILnc1 was predominantly cytoplasmic (Fig. S2F). The findings were verified by FISH. Thus, HGDILnc1 is a lncRNA that is strongly expressed in SBVM.

To investigate the function of HGDILnc1, we used shHGDILnc1 Lentivirus and HGDILnc1 Lentivirus to silence and over-express it in HUVEC cells (Fig. S2H). Knockdown of HGDILnc1 significantly inhibited capillary tube formation of HUVECs (Fig. 2F), while HGDILnc1 overexpression significantly promoted angiogenesis (Fig. S2I). Neither knockdown nor overexpression affected cell proliferation, shown by CCK8 (Fig. S2J). The Transwell assay showed reduced migration in silenced cells (Fig. 2G), while HGDILnc1 overexpression promoted cell migration (Fig. S2K). In addition, the wound-healing assay confirmed that HGDILnc1 silencing inhibited migration (Fig. 2H), and HGDILnc1 overexpression promoted cell migration of HUVECs cell lines (Fig. S2L). We then evaluated the effect of HGDILnc1 on angiogenesis in vivo, injecting shHGDILnc1 or control cells with Matrigel into nude mice and examining the primary vascular network by anti-CD31 staining after 14 days. Fewer vessels were seen in the HGDILnc1-silenced group (Fig. 2I) while more were visible in the HGDILnc1-overexpressed group (Fig. S2M). Collectively, HGDILnc1 promoted angiogenesis via regulation of cell migration.

### Glucose deprivation and hypoxia co-induce upregulation of HGDILnc1 via transcription factor NeuroD1

**Fig. 3:**
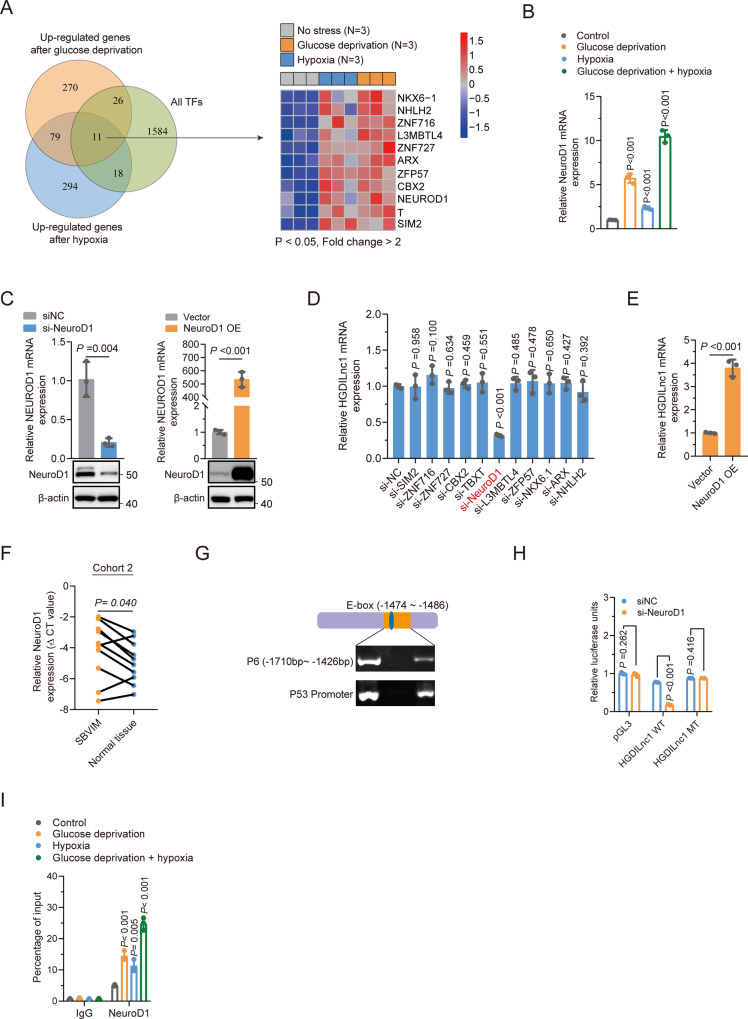
Glucose deprivation and hypoxia co-induced upregulation of HGDILnc1 via transcription factor NeuroD1. **A** Left: schematic diagram of screening procedure for differential expression (Fold change >2, *P* < 0.05) after hypoxia and glucose deprivation. Right: Heatmap of of transcription factors differentially upregulated between experimental and control groups. **B** NeuroD1 mRNA expression levels in HUVEC cells measured by using qRT–PCR after glucose deprivation, hypoxia, or glucose deprivation with hypoxia treatment. **C** NeuroD1 mRNA and protein expression levels in HUVEC cells by qRT–PCR and Western blot, respectively, after NeuroD1 silencing or NeuroD1 overexpression. **D** HGDILnc1 expression levels in HUVEC cells by qRT–PCR after silencing of candidate transcription factors. **E** HGDILnc1 expression levels in HUVEC cells measured by qRT–PCR after overexpression of NeuroD1. **F** NeuroD1 expression levels in SBVM and paired adjacent tissues measured by using qRT–PCR in cohort 2. **G** PCR amplification of NeuroD1-binding fragments of post-ChIP HGDILnc1 promoter fragments with anti-NeuroD1 antibody in cell HUVEC cell lysates. Fragments of P53 promoter serves as a positive control for NeuroD1-binging fragments. **H** Luciferase activity in NeuroD1-silenced HUVEC cells after transfection with HGDILnc1 wild-type (WT) or deletion mutants (MT) promoter luciferase reporter vectors. **I** Binding capacity of NeuroD1 to the indicated post-ChIP HGDILnc1 promoter fragments with anti-NeuroD1 antibody in in HUVEC cell lysates after glucose deprivation, hypoxia, or glucose deprivation with hypoxia treatment by ChIP-qPCR. The negative control was an anti-IgG antibody.

Transcription factors are known to regulate lncRNA transcription [28, 29]. Here, found that NeuroD1 could directly regulate HGDILnc1 transcription by binding to the HGDILnc1 promoter. Using stringent filtering criteria (*P* < 0.05, Fold change >2, Fig. 3A), we overlapped up-regulated genes after glucose deprivation (*n* = 386), hypoxia (*n* = 402), and all potential transcriptional factors (*n* = 1639). This identified 11 genes as potential regulators of HGDILnc1 transcription. The expression of these genes was then determined in cells after different treatments (Figs. 3B and S3A), finding that the expression patterns of NeuroD1, ZFP57, TBXT, ZNF716, SIM2, and NHLH2 were consistent with the microarray data (Figs. 3B and S3A). The genes were then silenced (Fig. 3C left panel, Fig. S3B) and HGDILnc1 mRNA expression was measured. This showed that HGDILnc1 levels were downregulated only after NeuroD1 knockdown (Fig. 3D). Overexpression of NeuroD1 (Fig. 3C right panel) resulted in upregulation of HGDILnc1 (Fig. 3E). NeuroD1 was also expressed to a greater extent in the 10 SBVM tissues compared to controls (Fig. 3F) in cohort 2. Collectively, NeuroD1 is highly expressed in SBVM and may upregulate HGDILnc1 expression.

Since NeuroD1, as a transcription factor, is primarily located in the nucleus, we hypothesized that it binds directly to the HGDILnc1 promoter. ChIP showed that NeuroD1 bound to fragments #6 (−1710 to −1426) of the HGDILnc1 promoter (Figs. 3G and S3C). Further in silico prediction using this binding motif in the JASPAR database (Fig. S3D) identified one putative NeuroD1 binding site in the HGDILnc1 promoter (Fig. S3E). To verify that the function of this site on HGDILnc1 expression, the wild-type (WT) or mutant HGDILnc1 (MT) promoters were cloned into a luciferase reporter plasmid (Fig. S3F) and transfected the reporter construct into control and si-NeuroD1-transfected HUVEC cells. Decreased WT activity was seen in the NeuroD1 knockdown while activity was restored in the MT cells (Fig. 3H). Similarly, NeuroD1 overexpression increased the luciferase activity of the WT, but not the MT (Fig. S3F). Since HGDILnc1 expression was increased after hypoxia and glucose deprivation, we speculated that the binding capacity of NeuroD1 to the HGDILnc1 promoter may also increase after hypoxia and glucose deprivation. Interestingly, ChIP-qPCR results showed more NeuroD1 binding to the HGDILnc1 promoter in the hypoxic and glucose-deprived with more binding to the promoter seen in the co-treated group (Fig. 3I).

### HGDILnc1 promotes glycolysis via ENO1

**Fig. 4:**
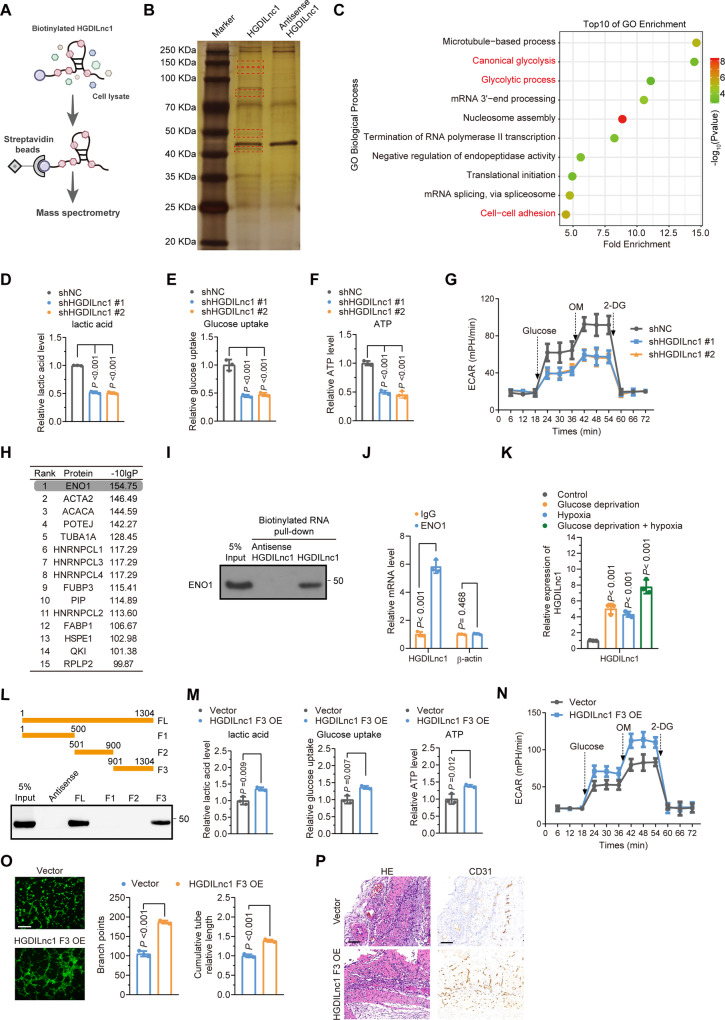
HGDILnc1 promoted glycolysis via ENO1. **A** Pull-down assay experimental design. Biotinylated HGDILnc1 and antisense-HGDILnc1 RNA were incubated with HUVEC cell lysates. **B** Silver staining of HGDILnc1-associated proteins. Five HGDILnc1-associated bands (rectangular box) were excised and analyzed by mass spectrometry. **C** GO enrichment of HGDILnc1-associated proteins after RNA pull-down. **D**–**F** Lactate production (**D**), glucose uptake (**E**), and ATP production (**F**) in HGDILnc1-silenced HUVEC cells by colorimetric analysis. **G** Extracellular acid ratio (ECAR) in HGDILnc1-silenced HUVEC cells. OM oligomycin, 2-DG 2-deoxyglucose. **H** Top 15 HGDILnc1-associated cellular proteins according to the −10lgP among the HGDILnc1-RNA pull-down proteins. **I** Western blot of ENO1 from antisense HGDILnc1 and HGDILnc1 pull-down assays. **J** RNA immunoprecipitation with anti-ENO1 antibody and specific primers were used to detect HGDILnc1. β-actin was used as a negative control. **K** Binding capacity of ENO1 to HGDILnc1 following RNA immunoprecipitation using an antibody against ENO1 in HUVEC cell lysates after measurement of glucose deprivation, hypoxia, or glucose deprivation with hypoxia treatment by qRT-PCR. **L** Western blot of ENO1 in samples pulled down by full-length (FL) or truncated HGDILnc1 (F1: 1–500, F2: 501–900, F3: 901–1304). **M** Lactate production (left), glucose uptake (middle), and ATP production (right) were measured in HUVEC cells overexpressing the truncated HGDILnc1 F3 fragment by colorimetric analysis. **N** Extracellular acid ratio (ECAR) in cells after overexpression of the truncated HGDILnc1 F3 fragment in HUVEC cells. OM oligomycin, 2-DG 2-deoxyglucose. **O** Capillary tube formation for evaluating angiogenesis in HUVECs after overexpression of the truncated HGDILnc1 F3 fragment. **P** H&E-stained 10 µm paraffin sections (left) and CD31-labeled paraffin sections (right) of the Matrigel plugs after overexpression of truncated HGDILnc1 F3 fragment in an in vivo Matrigel implantation model.

LncRNAs may interact with various cellular components, including glycolysis-related enzymes [14, 15]. We used pull-down experiments to identify HGDILnc1 interaction partners. Total protein was extracted from cells and incubated with either biotinylated HGDILnc1 or antisense HGDILnc1 RNA (negative control), followed by pull-down with streptavidin (Fig. 4A), followed by SDS-PAGE with silver staining. This resulted in five specific pull-down bands (Fig. 4B) which were then analyzed by mass spectrometry, resulting in the identification of 240 proteins in the HGDILnc1 pull-down group compared to the antisense HGDILnc1 pull-down (Table S5). To further investigate any potential role of these HGDILnc1-associated cellular proteins, we performed Gene ontology (GO) and Kyoto Encyclopedia of Genes and Genomes (KEGG) analyses. GO showed significant association of HGDILnc1 with “glycolysis”, “RNA process and metabolism”, “transcription”, and “translation” as well as “cell–cell adhesion” (Fig. 4C), and KEGG analysis revealed that HGDILnc1 is significantly associated with metabolic pathways involved (Fig. S4A). As glycolysis was enriched in both the GO and KEGG analyses, we speculated that HGDILnc1 may directly influence glycolytic metabolism to modulate angiogenesis. To verify this, we added 2-DG (glycolysis inhibitor) to the HGDILnc1-overexpressed cells, finding that this significantly blocked HGDILnc1 overexpression-induced angiogenesis (Fig. S4B). These findings indicate that HGDILnc1 may regulate glycolysis to promote angiogenesis.

We next investigated whether HGDILnc1 regulates glycolytic metabolism. HGDILnc1 silencing resulted in reduced glucose uptake, lactate secretion, ATP level, and extracellular acidification rate (ECAR) [30] relative to controls (Fig. 4D–F), while cells overexpression HGDILnc1 showed increased glucose uptake, lactate secretion, ATP level, and ECAR (Fig. S4C–F). To analyze the action of HGDILnc1 in glycolysis, we analyzed the top 15 proteins according to the -10lgP (Fig. 4H) selecting the top protein ENO1, a glycolysis enzyme [28], for verification. Western blotting indicated that ENO1 bound to HGDILnc1 (Fig. 4I). Immunoprecipitation of ENO1 from cell lysates and PCR analysis of bound RNA showed direct binding between ENO1 and HGDILnc1 (Fig. 4J). Interestingly, this binding was enhanced by glucose deprivation and hypoxia (Fig. 4K). Thus, HGDILnc1 binds specifically to ENO1 in a glucose and hypoxia-dependent manner.

To identify the ENO1-binding site on HGDILnc1, three fragments GClnc1 (1–500F1, 501–900F2, 901–1304F3) were constructed, biotinylated, and used for pull-down experiments, identifying the 3′ HGDILnc1 fragment of HGDILnc1 as containing the interaction site (Fig. 4L). Overexpression of the F3 fragment increased glucose uptake, lactate secretion, ATP levels, and ECAR (Fig. 4M, N) as well as promoting angiogenesis in vitro and in vivo (Fig. 4O, P). Thus, the 3′ fragment of HGDILnc1 may mediate interaction with ENO1 in glycolysis and angiogenesis.

### HGDILnc1 regulates the stability of ENO1 via suppression of ENO1 SUMOylation-triggered ubiquitination

**Fig. 5:**
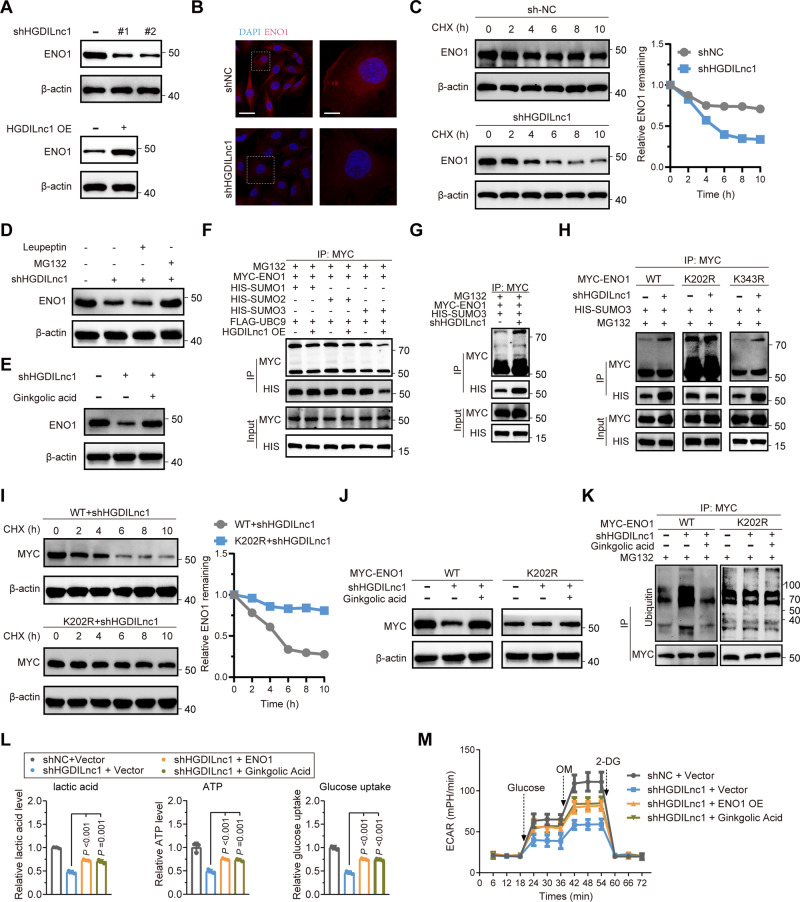
HGDILnc1 regulates the stability of ENO1 through suppression of ENO1 SUMOylation-triggered ubiquitination. **A** Western blots of ENO1 after ENO1 silencing (up) and overexpression of HGDILnc1 (down). **B** Representative image of immunofluorescence staining of ENO1 expression in HGDILnc1-silenced HUVEC cells. Scale bars, left: 20 μm; right: 5 μm. **C** Western blot was used for measuring the half-life of ENO1 after treatment with 20 μM cycloheximide (CHX) in HGDILnc1-silenced HUVECs. **D** Western blot of ENO1 in HGDILnc1-silenced HUVEC cells after treatment of 10 μM Leupeptin or 20 μM MG132. **E** Western blot of ENO1 in HGDILnc1-silenced HUVEC cells with and without treatment with 20 μM ginkgolic acid. **F** Co-immunoprecipitation (co-IP) analysis for detection of SUMOylation in HGDILnc1-overexpressing cells after transfection with MYC-ENO1 and FLAG-UBC9 and HIS-SUMOs using the specified antibodies. **G** Co-IP analysis for detection of SUMOylation in HGDILnc1-silenced HUVEC cells with co-transfection of MYC-ENO1 and HIS-SUMO3 using the specified antibodies. **H** Co-IP analysis for the detection of SUMOylation in HGDILnc1-silenced HUVEC cells with co-transfection of HIS-SUMO3 and MYC-ENO1 WT, K202R, and K343R using the specified antibodies. **I** Western blot was used for measurement of the half-life of MYC-ENO1 WT and K202R after treatment with 20 μM CHX in HGDILnc1-silenced HUVEC cells. **J** Western blot of the MYC-ENO1 WT and K202R in HGDILnc1-silenced HUVEC cells with or without treatment with the 20 μM ginkgolic acid. **K** Co-IP analysis for the detection of ubiquitination in HGDILnc1-silenced HUVEC cells with transfection of MYC-ENO1 WT or K202R with or without treatment with20 μM ginkgolic acid using the specified antibodies. **L** Lactate production (left), glucose uptake (middle) and ATP production (right) were measured in HGDILnc1-silenced HUVEC cells with ENO1 transfection or treatment with 20 μM ginkgolic acid. **M** ECAR in HGDILnc1-silenced HUVEC cells with transfection of ENO1 or treatment of 20 μM ginkgolic acid.

HGDILnc1 silencing resulted in reduced ENO1 protein (Fig. 5A, up) but not mRNA levels (Fig. S5A, left), while overexpression resulted in increased ENO1 protein (Fig. 5A, down) but not mRNA levels (Fig. S5A, right). IF confirmed these results for HGDILnc1 silencing (Fig. 5B) and overexpression (Fig. S5B). The cycloheximidechase (CHX) assay revealed that HGDILnc1 silencing accelerated ENO1 degradation (Fig. 5C). This degradation could be rescued by the protease inhibitor MG132 but not the lysosomal inhibitor Leupeptin (Fig. 5D). SUMOylation has been reported to modulate protein stability by triggering ubiquitination induced proteasomal degradation [23, 24] and could be regulated by lncRNAs [31, 32]. Addition of the SUMOylation inhibitor ginkgolic acid to silenced cells also rescued ENO1 degradation (Fig. 5E), indicating that ENO1 SUMOylation may promote HGDILnc1 silencing-induced ENO1 protein destabilization. To verify ENO1 SUMOylation, cells were separately co-transfected with ENO1 and UBC9 (ubiquitin-conjugating enzyme E2I), SUMO1, SUMO2, or SUMO3 and analyzed by immunoprecipitation. SUMO-conjugated ENO1 was identified using an anti-MYC monoclonal antibody (Fig. S5C). However, SUMO3-conjugated ENO1, but not SUMO1 or SUMO2, was less precipitated in HGDILnc1-overexpressing cells compared with controls (Fig. 5F), while SUMO3-conjugated ENO1 was enhanced after HGDILnc1 silencing (Fig. 5G). To find which SUMOylation site mediated HGDILnc1 function, ENO1 was immunopurified and subjected to mass spectrometric analysis, which confirmed SUMOylation on residues K202 and K343 (Fig. S5D). Plasmids expressing WT or mutant ENO1 (K202R and K343R) were transfected into cells, followed by SUMOylation measurement. Interestingly, SUMOylated ENO1-WT, ENO1-K343R but not K202R was increased after HGDILnc1 silencing, indicating residue K202 of ENO1 may mediate HGDILnc1 function (Fig. 5H). The CHX assay showed HGDILnc1 silencing accelerated ENO1-WT degradation but not ENO1-K202R (Fig. 5I). In addition, ginkgolic acid could rescue HGDILnc1 silencing-induced ENO1-WT but not K202R degradation (Fig. 5J). Ubiquitination of ENO1-WT but not ENO1-K202R was increased in silenced cells and was rescued by addition of ginkgolic acid (Fig. 5K). The above results confirmed that HGDILnc1 regulates the stability of ENO1 via suppression of ENO1 SUMOylation-triggered ubiquitination.

To then investigate whether ENO1 SUMOylation mediated HGDILnc1 function. In glycolysis metabolism assays, overexpression of ENO1 or addition of ginkgolic acid dramatically rescue HGDILnc1 silencing-induced decrease in glucose uptake, lactate secretion, ATP levels, and ECAR (Fig. 5L, M).

### HGDILnc1 regulates the transcription of ALDOC via upregulation of H2BK16ac level in the promoter of ALDOC

ENO1 overexpression only partially rescued reduced glycolysis resulting from HGDILnc1 silencing (Fig. 5L, M) and we speculate there may be another mechanism for HGDILnc1 in the regulation of glycolysis metabolism. Since about 40% of HGDILnc1 localized to the nucleus (Fig. S2F, G), we hypothesized that HGDILnc1 may regulate transcription of glycolysis-related genes and performed RNA-Seq to compare gene levels in the shHGDILnc1 and control transfectants. This showed 692 downregulated and 916 upregulated genes after HGDILnc1 knockdown (Table S6). GSEA identified the gene sets “GO_NEGATIVE_REGULATION_OF_SPROUTING_ANGIOGENESIS” (angiogenesis), “HALLMARK_GLYCOLYSIS” (glycolysis), “GO_CELLULAR_RESPONSE_TO_GLUCOSE _STARVATION” (genes upregulated in control in comparison with low glucose), and “GO_RESPONSE_TO_OXYGEN_LEVELS” (genes upregulated in control in comparison with hypoxia) as correlated with HGDILnc1 downregulation (Fig. S6A–D). Glycolysis was also enriched in the KEGG analysis of differently expressed genes (Fig. S6E).

**Fig. 6:**
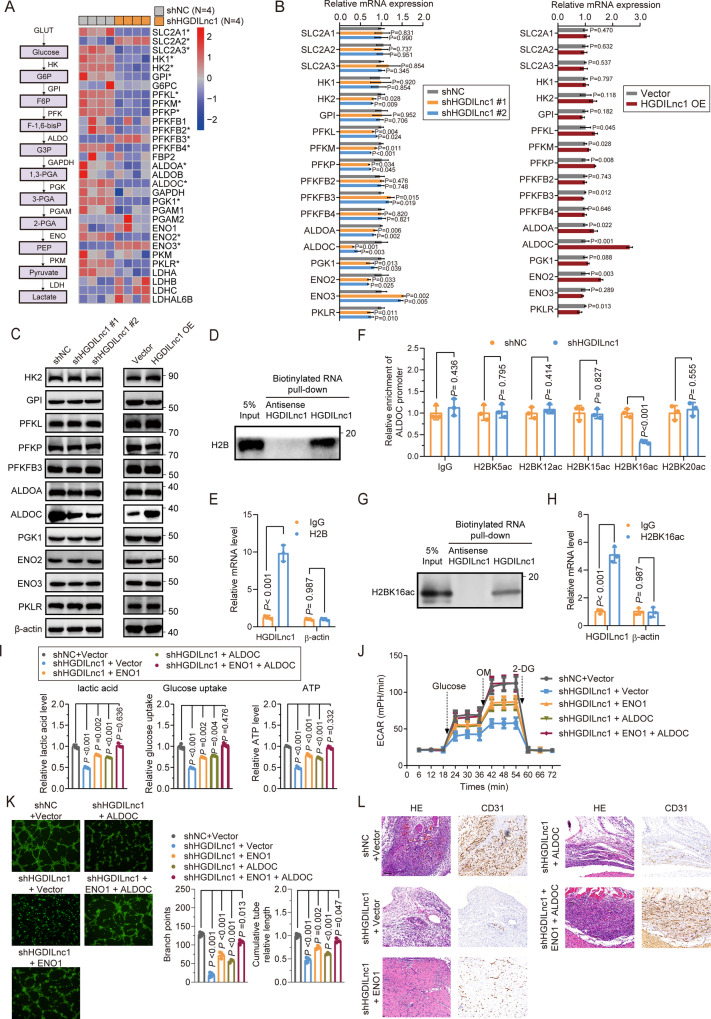
HGDILnc1 regulates the transcription of ALDOC through upregulation of H2BK16ac levels in the ALDOC promoter. **A** Left: schematic illustration for aerobic glycolysis-related enzyme genes. Right: Heatmap showing differentially expressed glycolysis-related enzyme genes between HGDILnc1-silenced cells and controls, by RNA-sequencing. Asterisk (*) indicates a significant difference in expression of the indicated gene between HGDILnc1-silenced cells and controls. **B** mRNA expression levels of potential differentially expressed aerobic glycolysis-related genes evaluated by qRT–PCR in HGDILnc1-silenced HUVECs (left) or HGDILnc1-overexpressing cells (right). **C** Western blot of potential differentially expressed aerobic glycolysis-related proteins in HGDILnc1-silenced HUVEC cells (left) or HGDILnc1-overexpressing cells (right). **D** Western blot of histone H2B from antisense HGDILnc1 and HGDILnc1 pull-down assays. **E** RNA immunoprecipitation experiments were performed using an anti-H2B antibody, and specific primers were used to detect HGDILnc1. β-actin was used as a negative control. **F** Binding capacity of candidate H2B acetylation to the ALDOC promoter measured by ChIP-qPCR after knocking down HGDILnc1. An IgG antibody was used as a negative control. **G** Western blot of histone H2BK16ac from antisense HGDILnc1 and HGDILnc1 pull-down assays. **H** RNA immunoprecipitation experiments were performed using an anti-H2BK16ac antibody, and specific primers were used to detect HGDILnc1. β-actin was used as a negative control. **I** Lactate production (left), glucose uptake (middle) and ATP production (right) were measured in HGDILnc1-silenced HUVEC cells with transfection of ENO1 or ALDOC or co-transfection of ENO1 and ALDOC, shown by colorimetric analysis. **J** Extracellular acid ratio (ECAR) in HGDILnc1-silenced HUVEC cells with transfection of ENO1 or ALDOC or co-transfection of ENO1 and ALDOC. OM oligomycin, 2-DG 2-deoxyglucose. **K** Capillary tube formation for evaluating angiogenesis in HGDILnc1-silenced HUVEC cells with transfection of ENO1 or ALDOC or co-transfection of ENO1 and ALDOC. **L** Representative images of H&E-stained 10 µm paraffin sections (left) and CD31-labeled paraffin sections (right) of the Matrigel plugs after HGDILnc1 silencing with overexpression of ENO1 or ALDOC or co-overexpression of ENO1 and ALDOC in an in vivo Matrigel implantation model.

To further elucidate the suppression of glycolysis by HGDILnc1 silencing, we assessed the RNA-Seq expression of genes linked to aerobic glycolysis (Fig. 6A), verifying the differentially expressed genes by qRT-PCR and Western blotting. This identified only ALDOC as a regulated gene (Fig. 6B, C). This was confirmed by IF results, where HGDILnc1 silencing downregulated ALDOC and HGDILnc1 overexpression upregulated ALDOC (Fig. S6F). LncRNAs have been reported to modulate gene expression in trans by regulating histone modification [33, 34]. Results showed POL II at the ALDOC locus was reduced by silencing HGDILnc1 (Fig. S6G) or augmented by HGDILnc1 overexpression (Fig. S6H), indicating HGDILnc1 augmented transcription of ALDOC. Mass spectrometry of HGDILnc1 pull-down RNA indicated that histone H2B may be regulated by HGDILnc1 (Table S5), verified by pull-down (Fig. 6D) and RIP (Fig. 6E). Differently acetylated H2B forms have been reported, namely, H2BK5ac, H2BK12ac, H2BK15ac, H2BK16ac, and H2BK20ac, which may be regulated by HGDILnc1 [35]. ChIP-PCR showed that only H2BK16ac was significantly reduced after silencing HGDILnc1 (Fig. 6F) and was augmented by HGDILnc1 overexpression (Fig. S6I). To validate direct relationship between HGDILnc1 and H2BK16ac, we performed RNA pull-down (Fig. 6G) and RIP experiment (Fig. 6H), which showed HGDILnc1 directly interact with H2BK16ac. Together, these data suggested HGDILnc1 regulate the transcription of ALDOC via upregulation of H2BK16ac level in the promoter of ALDOC.

We next hypothesized that ENO1 and ALDOC co-mediated the biological function of HGDILnc1. We first eliminated direct regulation relationship of ENO1 and ALDOC via WB analysis of another protein expression after knockdown of ENO1 or ALDOC (Fig. S6J, K). In glycolysis metabolism assays, overexpression of ENO1 or ALDOC could partially rescue but co-transfection of ENO1 and ALDOC could totally rescue HGDILnc1 silencing-induced decrease in glucose uptake, lactate secretion, ATP levels, and ECAR (Fig. 6I, J). Analysis of angiogenesis showed that overexpression of ENO1 or ALDOC partially rescued the inhibition mediated by HGDILnc1 silencing and co-transfection of ENO1 and ALDOC could rescue angiogenesis inhibited by HGDILnc1 silencing to a greater extent (Fig. 6K, L). These results indicated that HGDILnc1 promoted angiogenesis via co-regulation of ENO1 and ALDOC in a different way from glycolysis.

### Clinical significance of NeuroD1/HGDILnc1/ENO1/ALDOC axis in SBVM patients

**Fig. 7:**
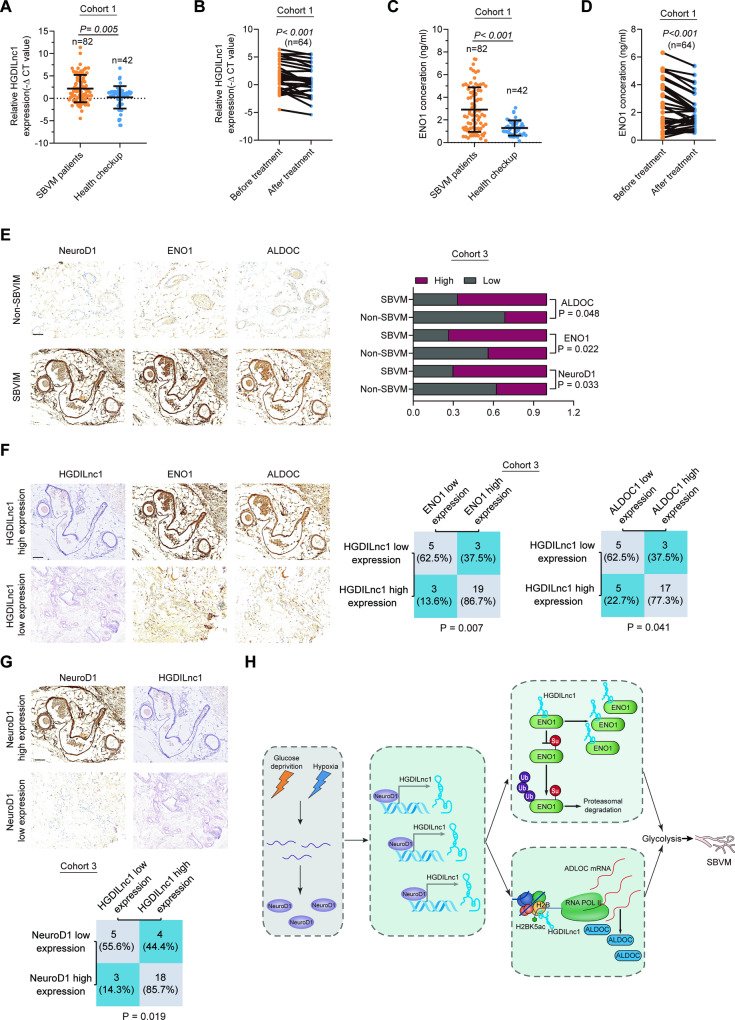
Clinical significance of the NeuroD1/HGDILnc1/ENO1/ALDOC axis in SBVM patients. **A** HGDILnc1 expression in the sera of healthy individuals (*n* = 42) and SBVM patients (*n* = 82) in cohort 1. **B** HGDILnc1 expression in the sera of SBVM patients before and after thalidomide treatment (*n* = 32) in cohort 1. **C** ENO1 expression in the sera of healthy individuals (*n* = 42) and SBVM patients (*n* = 82) in cohort 1. **D** ENO1 expression in the sera of SBVM patients before and after thalidomide treatment (*n* = 32) in cohort 1. **E** Representative immunohistochemical images (left) of NeuroD1, ENO1, and ALDOC in SBVM tissues and non-SBVM tissues using IHC analysis and statistical analysis of the proportions of high and low staining in SBVM tissues and non-SBVM tissues (right). Scale bars: 100 μm. **F** Representative in situ hybridization images of HGDILnc1 and immunohistochemical images of ENO1 and ALDOC expression in SBVM with high expression and low HGDILnc1 expression (left), and statistical analysis of SBVM tissues under different staining conditions (right) in cohort 3. Scale bars: 100 μm. **G** Representative immunohistochemical images of NeuroD1 expression and in situ hybridization images of HGDILnc1 expression in SBVM with high and low NeuroD1 expression (up), and statistical analysis of SBVM tissues under different staining conditions (down) in cohort 3. Scale bars: 100 μm. **H** Schematic diagram of the relationship among glucose deprivation, hypoxia, NeuroD1, HGDILnc1, ENO1, ALDOC, glycolysis metabolism, and SBVM. Note: the representative immunohistochemical images of ENO1, ALDOC in SBVM tissue in (**E**) were same to the representative immunohistochemical images of ENO1, ALDOC in SBVM with high expression of HGDILnc1 in (**F**) for illustration of different proteins expression in the same SBVM patients.

We first compared plasma HGDILnc1 levels between SBVM patients and controls, observing higher levels (Fig. 7A) in cohort 1. In addition, HGDILnc1 expression was decreased after thalidomide treatment (Fig. 7B) in cohort 1. Higher expression of ENO1 was also observed in SBVM plasma (Fig. 7C) and ENO1 was decreased after thalidomide treatment (Fig. 7D) in cohort 1. Investigation of the association between plasma HIF1α, HGDILnc1, and ENO1 in the plasma demonstrated that the samples expressing high HIF1α in the plasma had raised HGDILnc1 (Fig. S7A) and ENO1 (Fig. S7B) and that the samples expressing high HGDILnc1 in the plasma had high expression of ENO1 (Fig. S7C) in cohort 1.

IHC indicated higher levels of NeuroD1, ENO1, and ALDOC in SBVM tissues (Fig. 7E) in cohort 3. High levels of HGDILnc1 correlated with high ENO1 and ALDOC expression (Fig. 7F) in cohort 3. Similarly, samples with high NeuroD1 tended to have high expression of HGDILnc1 (Fig. 7G), ENO1 and ALDOC expression (Fig. S7D) in cohort 3.

Thus, we have demonstrated that hypoxia and glucose deprivation co-induced HGDILnc1 visa transcription factor NeuroD1 may function as an angiogenesis gene by suppression of ENO1 SUMOylation-triggered ubiquitination, and activation of ALDOC transcription via upregulation of H2BK16ac level in the promoter of ALDOC (Fig. 7H).

## Discussion

Here, we have shown that hypoxia and low-glucose environments are present in SBVM and that these factors co-augmented HGDILnc1 expression and its function in promoting angiogenesis through the targeting of glycolysis-related genes, both stimulating glycolysis and aberrant angiogenesis.

Previous studies have proposed that raised tension in the bowel wall produces low-grade obstruction, leading to mucosal ischemia and hypoxia [6, 7]. The present study confirmed hypoxia and low-glucose environment in SBVM tissues using ELISA, immunohistochemistry, and qRT-PCR. In addition, hypoxia and a low-glucose environment co-induce angiogenesis, which is also supported by previous studies [36–39].

The involvement of hypoxia and reduced glucose in the functional co-regulation of any lncRNA or lncRNA in SBVM has not been reported in the literature. Epigenetic modifications of nucleic acids usually result from environmental changes [40, 41]. Bioinformatics analysis revealed that hypoxia and low-glucose environment might regulate HGDILnc1 via NeuroD1. NeuroD1, member of NeuroD family of basic helix–loop–helix (bHLH) transcription factors, forms heterodimers with other bHLH proteins and activates transcription of genes that contain a specific DNA sequence known as the E-box, have shown it function in regulates expression of the insulin gene, regulation of beta-cell development and nervous system development [42, 43]. In this study, we found NeuroD1 regulate HGDILnc1 expression via directly binding to the promoter of HGDILnc1. Further analysis indicated that NeuroD1 may directly regulate HGDILnc1 expression in response to hypoxia and glucose deprivation treatment.

Enhancement of glycolysis is largely the result of raised expression of factors responsible for glucose uptake and lactate synthesis and secretion [44]. Here, we have elucidated the role of HGDILnc1 in activating glycolysis and leading to angiogenesis. We discovered that HGDILnc1 directly interacts with ENO1 using mass spectrometric analysis and western blot analysis of RNA pull-down samples, and suppresses its SUMOylation, which has been reported to decrease protein stability by triggering ubiquitination-induced proteasomal degradation [23, 24]. We also showed that HGDILnc1 suppressed SUMO3 binding to the K202 site of ENO1 to protect its ubiquitination-induced proteasomal degradation. In support of our observation, lncRNAs PSTAR, and RMST use modified SUMOylation of hnRNPK and FUS to mediate their functions [31, 32]. On the other hand, RNA-Seq after HGDILnc1 silencing and subsequent verification also classified HGDILnc1 function on ALDOC transcription in trans [33, 34]. We confirmed that HGDILnc1 activated ALDOC transcription via upregulation of H2BK16ac level in the promoter of ALDOC. These findings indicate that HGDILnc1 promotes angiogenesis by modulating glycolysis and ENO1 protein stability and ALDOC transcription.

This work may have relevance for treating SBVM patients. We have found that HGDILnc1 and ENO1 levels in the serum or SBVM tissues are higher than in healthy controls or paired adjacent normal tissues, indicating HGDILnc1 or ENO1 may be biomarkers for SBVM diagnosis. In addition, HGDILnc1 or ENO1 in the serum was significantly decreased after thalidomide treatment for SBVM, and measurement of HGDILnc1, or ENO1 before and after treatment may predict patients’ response to thalidomide treatment.

To conclude, our study suggests that hypoxia and low-glucose environments were present in SBVM tissues, and co-augmented angiogenesis. Hypoxia and low-glucose environments co-induced HGDILnc1, which is higher expressed in SBVM tissue compared with normal tissue, could promoted glycolysis and angiogenesis.

## Materials and methods

### Patients and samples

Cohort 1 consisted of serum samples from 82 SBVM patients before thalidomide treatment collected from 2003 to 2020 together with 42 normal sera. In addition, paired serum samples from 32 SBVM patients treated with or without thalidomide were also collected in the cohort 1. The course of treatment was 4 months and the follow-up time was 12 months. The detailed clinical characteristics of patients in the cohort 1 was shown in the Table S7.1. The cohort 2 consisted of 10 SBVM tissues and paired para-SBVM normal tissue from 10 different patients for measure of RNA and glucose level. The detailed clinical characteristics of patients in the cohort 2 was shown in the Table S7.2. The cohort 3 consisted of 30 paraffin-embedded SBVM samples collected from patients in the Renji hospital who underwent surgery due to massive bleeding from 2000 to 2020 and 16 randomized selected non-SBVM samples. The detailed clinical characteristics of SBVM patients in the cohort 3 was shown in the Table S7.3. All patients provided informed consent and the study was approved by the Biomedical Ethics Committee of Renji Hospital (2015-088k).

### Cell culture

HUVECs were obtained from FuHeng Cell Center (Shanghai, China) and were grown in Endothelial Cell Medium (ScienCell, USA) at 37 °C in a 5% CO_2_ humidified environment. To produce hypoxia, cells were grown with 1% O_2_ and for glucose deprivation, they were grown in glucose-free medium.

### Lentivirus and plasmid construction and transfection

The control, control shRNA, HGDILnc1 shRNA adenovirus were constructed by Hanbio Biotechnology Co. Ltd (Shanghai, China) The control plasmid, HGDILnc1, ENO1, ALDOC, NeuroD1, HGDILnc1 F1/F2/F3, ENO1-K202R/K343R-overexpressing plasmids, and ENO1- overexpression plasmids were constructed by Shenggong Company (Shanghai, China). The siRNA of ENO1, ALDOC, NeuroD1 and the other 10 transcription factors were designed and constructed by Genepharma Technology (Shanghai, China). The FuGENE transfection reagent (Life Technologies, USA) was used for transfection.

### CCK8 assay

Cells (2 × 10^3^ per well) were seeded in 96-well plates. Viability was assessed using the Cell Counting Kit (Dojindo Molecular Technologies, Japan) at 0, 24, 48, and 72 h after treatment. Absorbances at 450 nm were measured using a VERSA Max microplate reader (MDS Analytical Technologies).

### Matrigel migration assay

Pretreated cells (1 × 10^4^ in 200 µl medium without FBS) were placed in the Transwell upper chamber (6 µm pore size; Millipore) chambers in 24-well plates while 600 µl of medium containing 20% FBS were placed in the lower chamber, and incubated at 37 °C. At specified times, cells on the lower part of the membrane were removed, fixed (4% paraformaldehyde), and stained (0.1% crystal violet). The remaining membrane was washed in PBS, air-dried, and the adherent cells counted under light microscopy (×400 magnification), using the average of cell numbers in five randomly selected fields.

### Tube formation assay

Cells (1 × 10^4^ per well) were seeded in Matrigel-coated (BD Biosciences, San Jose, CA, USA) 24-well plates. After 12 h, cells were stained using Calcein AM (Abcam, Cambridge, UK) and the endothelial tube formation was examined by confocal microscopy. Image J was used to measure tube lengths and branches.

### Animals and in vivo angiogenesis assay

The procedures of animal experiments were confirmed through the Institutional Animal Care and Use Committee of Renji Hospital, School of Medicine, Shanghai Jiaotong University. Each 3 BALB/cAnN-nu (Nude) female mice (6–8 weeks of age) were chosen for each group without randomization and utilized for in vivo angiogenesis assessment. In vivo analysis of Matrigel was exerted as explained. After treatment, cells were subsequently trypsinized and around 5 × 10^5^ cells were blended with 50 ml ECM and 350 ml ice-cold Matrigel (BD Biosciences). Next, the mixture was employed under the back skin of 8 week-old BALB/cAnN-nu (Nude) female mice. Ten to 14 days later, for histological assessment, Matrigel plugs were harvested. The CD31+ capillary density was evaluated as already described. Binding was done for all animals in this study.

### Lactate production assay

Lactate was measured using the L-Lactate Assay kit (ab65331, Abcam), per instructions. Transfected cells were seeded in 96-well plates and cultured overnight. The cells were then starved for 2 h, and the lactate concentration in the culture supernatant measured. Absorbances at 450 nm were measured).

### Glucose uptake assay

The Glucose Uptake Colorimetric Assay Kit (ab136955, Abcam)) was used per instructions. Pretreated cells (1 × 10^4^ per well) were cultured overnight, incubated with 100 µl/well of Krebs Ringer phosphate/HEPES for 40 min, after which 10 µl 10 mM 2-DG was added to each well and allowed to stand for 20 min. After harvesting in extraction buffer, glucose uptake was assessed, using an absorbance of 412 nm.

### ATP production assay

ATP was measured using the ATP Assay Kit (ab83355, Abcam), per instructions. One hundred microliters of cell lysate were added to 100 µl of the ATP reaction solution, incubated for 30 min, and absorbances at 570 nm read.

### Measurement of ECAR

Cell metabolic changes were measured with the Seahorse Extracellular Flux Analyzer XF96 (Seahorse Bioscience) per instructions. Cells (2 × 10^4^/well) cultured overnight, then serum-starved for 24 h. After incubation in unbuffered medium with 10 mM glucose, 1 µM oligomycin, and 80 mM deoxyglucose, ECAR measurements were normalized to total protein and expressed as mpH/min.

### RNA analysis and qRT-PCR

Total RNA was extracted by TRIzol reagent (Invitrogen) and 1 μg was reverse-transcribed using the PrimeScript RT Reagent Kit (Perfect Real-Time; TaKaRa, Japan), and measured by qRT-PCR. ACTB was used for normalization. miRNAs (0.5 µg) were reverse-transcribed to cDNA using a specific miRNA stem loop primer, and amplified transcripts were normalized to U6.. The primers were provided by Shenggong Company and are listed in Table S7.

### Western blotting

Proteins were separated on 8–15% SDS-PAGE and transferred to PVDF. Membranes were probed with primary and peroxidase-conjugated secondary antibodies (Kangchen, China) and visualized using chemoluminescence. The antibodies are shown in Table S8.

### Rapid amplification of cDNA ends

RACE PCR products were obtained using a GeneRacer^TM^ Kit (Invitrogen, USA) per instructions, separated on agarose, extracted, cloned into a pGM-T vector (Shenggong, Shanghai, China), and sequenced. RACE primers are shown in Table S7.

The details of immunofluorescence, fluorescent in situ hybridization (FISH), RNA in situ hybridization (ISH), immunohistochemistry, enzyme-linked immunosorbent assay (ELISA), co-immunoprecipitation (co-IP), chromatin immunoprecipitation (ChIP), RNA immunoprecipitation (RIP), RNA pull-down, Luciferase assay, liquid chromatography-mass spectrometry (LC-MS), RNA high-throughput sequencing, microarray, and statistical analysis are described in Supplementary Materials and Methods. All experiments have been replicated for three times except RNA high-throughput sequencing, microarray, and liquid chromatography-mass spectrometry.

### Supplementary information

Supplementary

Full and uncropped western blots

## Data Availability

The RNA sequence data have been deposited in NCBIs Gene Expression Omnibus (GEO, http://www.ncbi.nlm.nih.gov/geo/) and are accessible through GEO Series accession number GSE186473, GSE186474. Data are available upon reasonable request. All data relevant to the study are included in the article or uploaded as Supplementary Information. All data and source associated with this study are available from the corresponding author on reasonable request.
