# Supplementary material for: Hypoxia and low-glucose environments co-induced HGDILnc1 promote glycolysis and angiogenesis
Source: Cell Death Discov. 2024 Mar 12;10:132. doi: 10.1038/s41420-024-01903-w (PMC10933424; doi:10.1038/s41420-024-01903-w)
Supplement: Supplementary file 1 — Supplementary [file 41420_2024_1903_MOESM1_ESM.pdf]

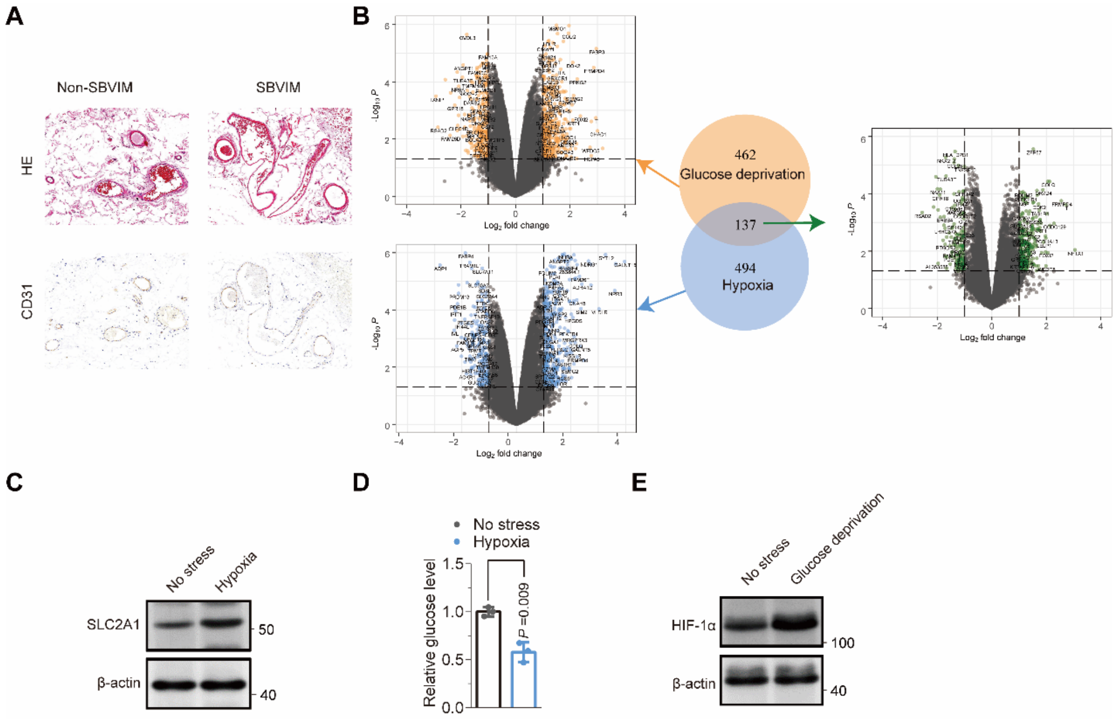

Fig. S1 Hypoxia and low glucose environment co-induced SBVM. **A.** H&E staining of 10 μm paraffin sections (up) and IHC (left) of CD31 in SBVM tissues and non-SBVM tissues. Scale Bars: 100 μm. **B.** Schematic illustration and volcano plot for differentially expressed genes (Fold change > 2, $P < 0.05$) after hypoxic and glucose deprivation treatment. **C.** Western blot of SLC2A1 expression in HUVEC cells after hypoxic treatment. **D.** Relative Glucose level in culture medium of HUVEC after hypoxic treatment measured by colorimetric analysis in cohort 2. **E.** Western blot of HIF-1α expression in HUVEC cells after glucose deprivation.

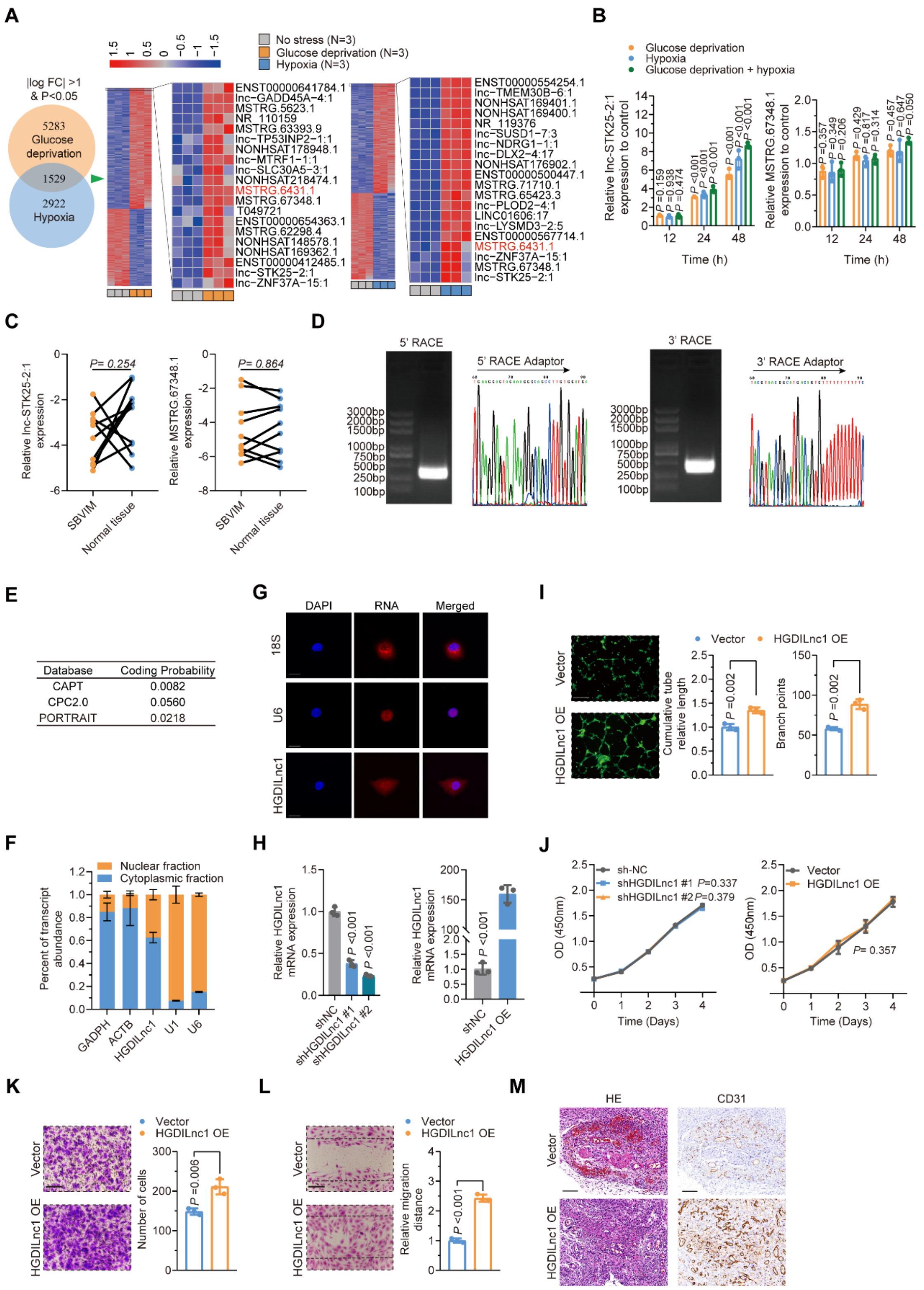

Fig. S2 Glucose deprivation and hypoxia co-induced HGDILnc1 stimulate angiogenesis both in vitro and in vivo. **A.** Left: Schematic diagram showing screening for differentially expressed LncRNAs after hypoxic and glucose-deprivation treatment. Middle: Heatmap of differentially expressed LncRNAs between glucose-deprived cells and controls. Right: Heatmap showing differentially expressed LncRNAs between hypoxic cells and controls. **B.** Candidate lncRNA

expression in HUVEC cell line measured by using qRT–PCR after glucose deprivation, hypoxia, or glucose deprivation with hypoxia treatment for 12, 24, 48 hours. **C.** Candidate lncRNA expression in SBVM and paired adjacent tissues measured by using qRT–PCR. **D.** Representative image of PCR products from the 5'-RACE with the sequences of the PCR products (left) and 3'-RACE with the sequences of the PCR products (right). **E.** The coding probability for the HGDILnc1 sequence was determined using CAPT, CPC2.0, and PORTRAIT databases. **F.** Expression of HGDILnc1 in cytoplasmic and nuclear fractions of HUVEC cells. U1, U6 RNA serves as a positive control for nuclear gene expression. GADPH and β-actin RNA serve as positive controls for cytoplasmic gene expression. **G.** Representative image of FISH for HGDILnc1 (red) in HUVEC cells. Nuclei are blue (DAPI). U6 RNA was a positive control for nuclear gene expression. 18S RNA was a positive control for cytoplasmic gene expression. Scale Bars: 20 μm. **H.** HGDILnc1 expression levels in HUVEC cells determined by qRT–PCR after HGDILnc1 silencing (left) and HGILnc1 overexpression (right). **I.** Capillary tube formation for evaluation of angiogenesis in HUVECs after HGDILnc1 overexpression. Scale Bars: 100 μm. **J.** CCK8 assays to determine the cell viability of HUVEC cells with HGDILnc1 silencing (left) or HGILnc1 overexpression (right). **K.** Transwell assays for evaluation of migration in HUVECs after HGDILnc1 overexpression. Scale Bars: 100 μm. **L.** Wound-healing assay for evaluation of migration in HUVECs after HGDILnc1 overexpression. Scale Bars: 100 μm. **M.** H&E staining (left) and CD31-labeling (right) of paraffin sections of the Matrigel plugs after HGDILnc1 overexpression in an *in vivo* model.

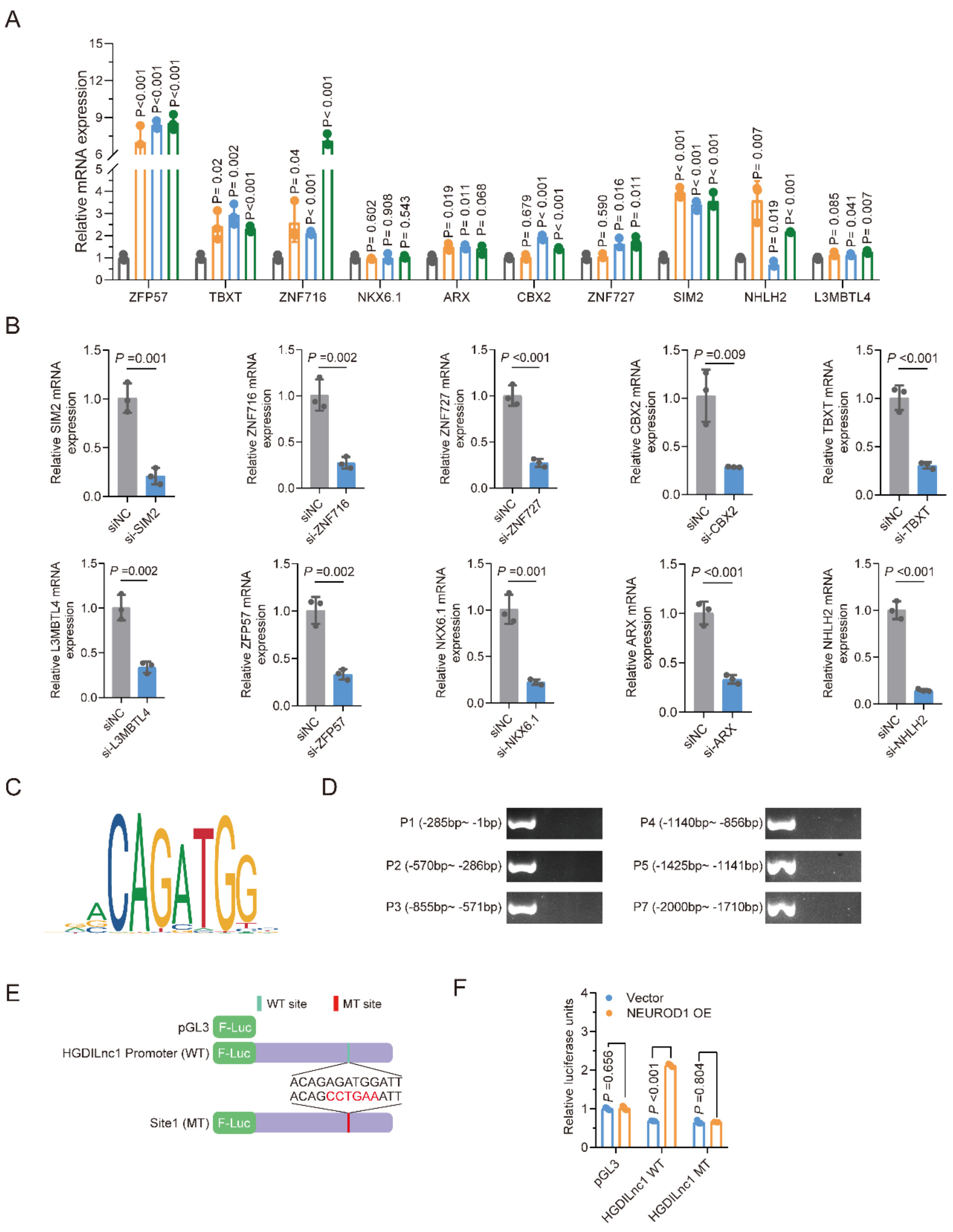

Fig S3. Glucose deprivation and hypoxia co-induced upregulation of HGDILnc1 by NeuroD1. **A.** mRNA expression of candidate transcription factors in HUVEC cells by qRT–PCR after glucose deprivation, hypoxia, or glucose deprivation with hypoxia treatment. **B.** Expression levels of candidate transcription factors in HUVEC cells by qRT–PCR after silencing of indicated transcription factors. **C.** PCR amplification of post-ChIP NeuroD1-binding fragments using anti-NeuroD1 antibody in HUVEC cell lysates. **D.** NeuroD1 motif for promoter binding. **E.** Diagram of HGDILnc1 wild-type (WT) and deletion mutants (MT) promoter-containing fragments showing putative NueroD1-binding sites. **F.** Luciferase activity in NeuroD1-overexpressing HUVEC cells

after transfection with HGDILnc1 wild-type (WT) or deletion mutants (MT) promoter luciferase reporter vectors.

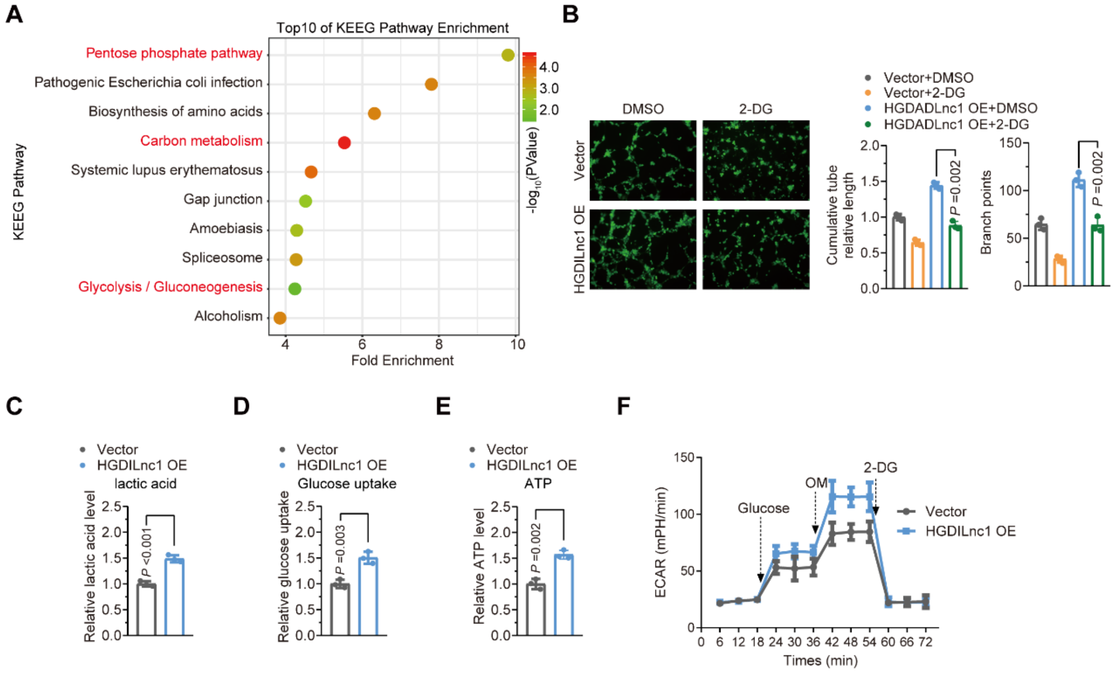

Fig S4. HGDILnc1 promotes glycolysis via ENO1. **A.** KEGG enrichment analysis (biological process) of HGDILnc1-associated cellular proteins using RNA pull-down. **B.** Capillary tube formation for evaluating angiogenesis in HGDILnc1-overexpressing after treatment with 5 mM 2-DG. 2-DG, and 2-deoxyglucose. **C-E** Lactate production (C), glucose uptake (D) and ATP production (E) were measured in HUVEC cells with HGDILnc1-overexpressing cells by colorimetric analysis. **F.** Extracellular acid ratio (ECAR) in HGDILnc1-overexpressing HUVEC cells.

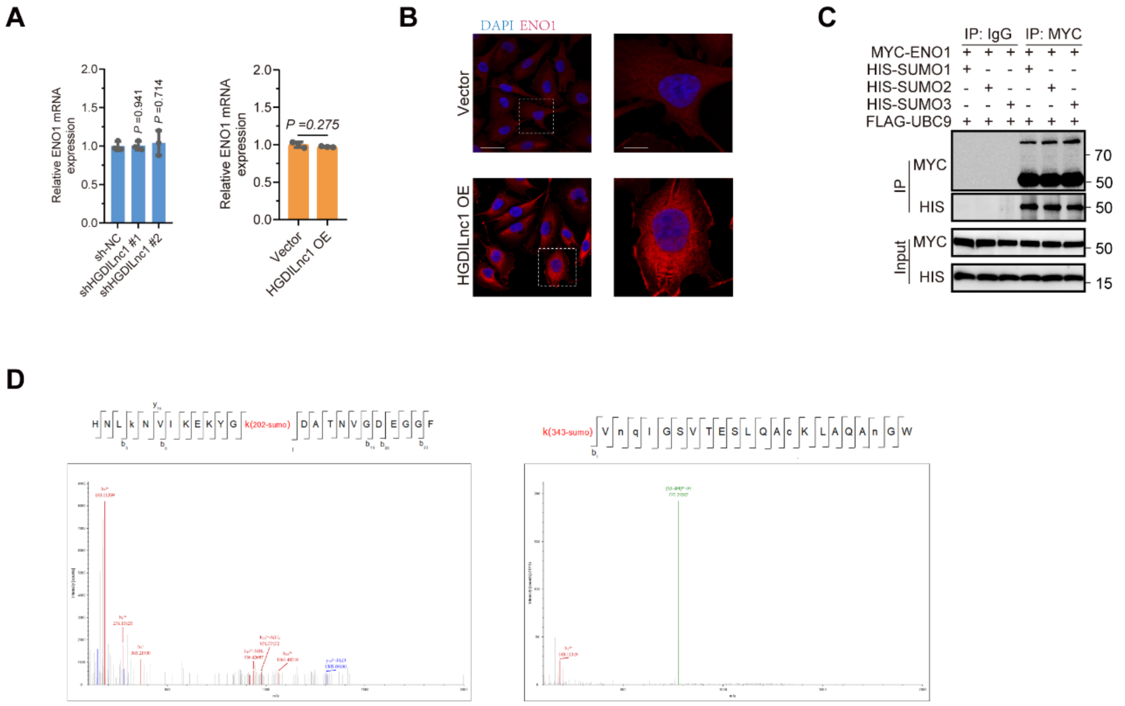

Fig S5. HGDILnc1 regulates the stability of ENO1 through suppression of ENO1 SUMOylation-triggered ubiquitination. **A.** mRNA expression levels of ENO1 in HUVEC cells measured by qRT–PCR after ENO1 silencing (left) and HGDILnc1 overexpression (right). **B.** Representative image of immunofluorescence staining of ENO1 expression in HGDILnc1-overexpressing HUVEC cells. Scale Bars, left: 20 μm; right: 5 μm. **C.** Coimmunoprecipitation (coIP) analysis for SUMOylation detection in HUVEC cells with co-transfection of MYC-ENO1 and FLAG-UBC9 and HIS-SUMO1, HIS-SUMO2, or HIS-SUMO3 using the specified antibodies. **D.** Mass spectrometric identification of SUMOylation sites.

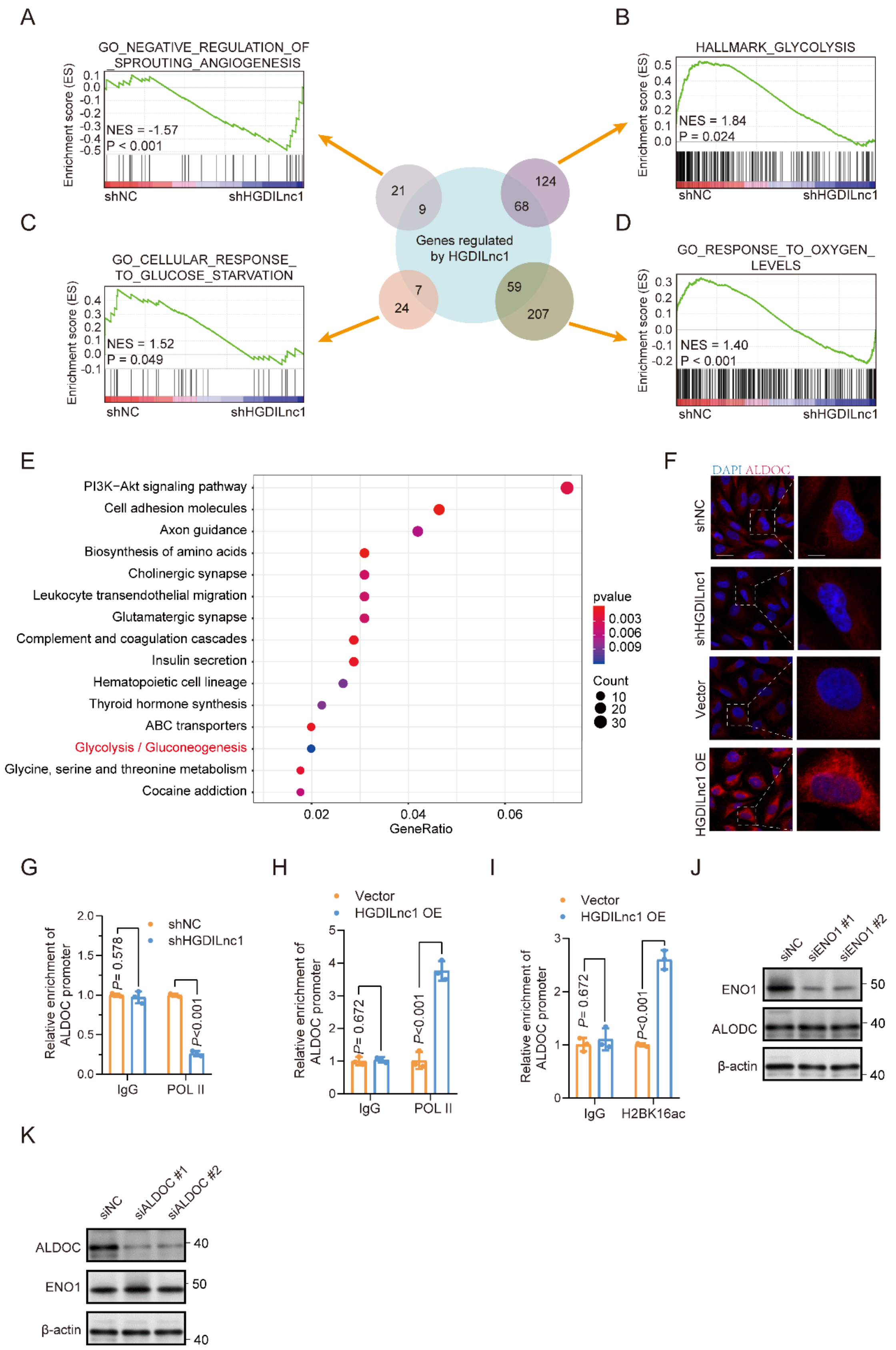

Fig S6. HGDILnc1 regulates the transcription of ALDOC though upregulation of H2BK16ac levels in the ALDOC promoter. **A-D.** Overview of GSEA used to identify the differential gene profiles between HGDILnc1-silenced HUVECs and controls. **E.** KEGG analysis of genes regulated by

HGDILnc1 using RNA-sequencing datasets of differentially expressed genes between HGDILnc1-silenced cells and controls. **F.** Representative image of immunofluorescence staining of ENO1 expression in HGDILnc1-silenced HUVEC cells (up) and HGDILnc1-overexpressing HUVEC cells (down). Scale Bars, left: 20 µm; right: 5 µm. **G.** Binding of RNA POL II to the ALDOC promoter measured by ChIP-qPCR after HGDILnc1 silencing. An IgG antibody was used as a negative control. **H.** Binding of RNA POL II to the ALDOC promoter measured by ChIP-qPCR after HGDILnc1 overexpression. An IgG antibody was used as a negative control. **I.** Binding capacity of H2BK16ac to the ALDOC promoter measured by ChIP-qPCR after knocking down HGDILnc1. An IgG antibody was used as a negative control. J. Western blot of ALDOC expression in ENO1-silenced HUVEC cells. K. Western blot of ENO1 expression in ALDOC-silenced HUVEC cells.

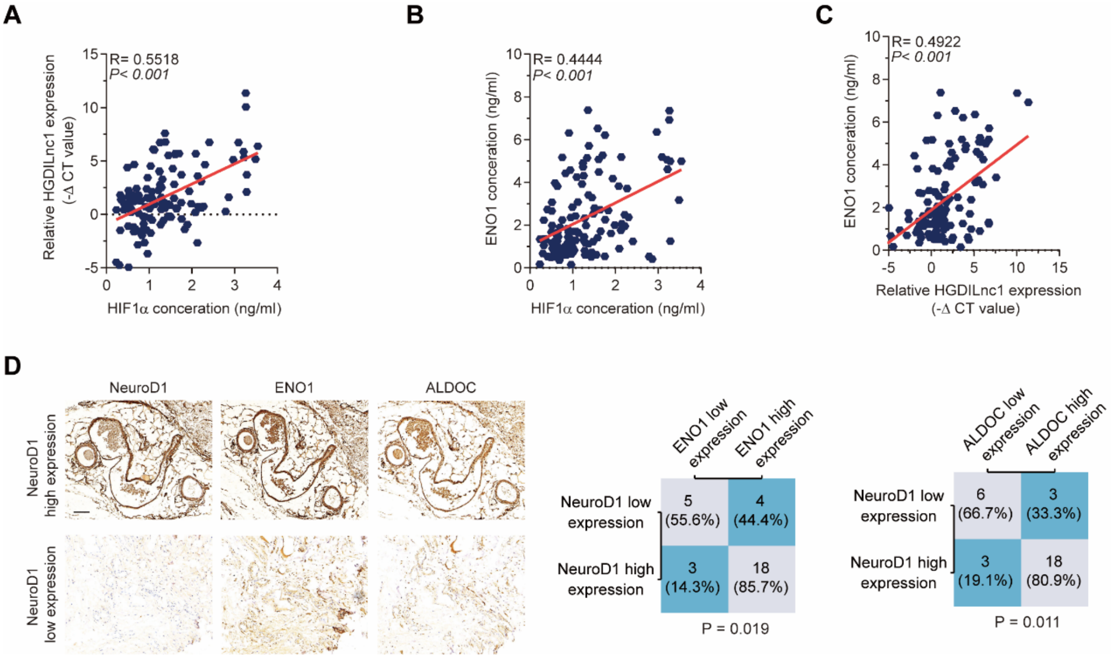

Fig S7. Clinical significance of the NeuroD1/HGDILnc1/ENO1/ALDOC axis in SBVM patients. **A** Correlation between HIF-1α and HGDILnc1 expression in the sera of healthy individuals (n = 42) and SBVM patients (n = 82). **B.** Correlation between HIF-1α and ENO1 expression in the sera of healthy individuals (n = 42) and SBVM patients (n = 82). **C.** Correlation between HGDILnc1 and ENO1 expression in the sera of healthy individuals (n = 42) and SBVM patients (n = 82). **D.** Representative immunohistochemical images of NeuroD1, ENO1, and ALDOC expression in SBVM with high and low NeuroD1 expression (left), and statistical analysis of SBVM tissues under different staining conditions (right). Scale Bars: 100 µm.

Martials and Method

Patients and Samples

Cohort 1 consisted of serum samples from 82 SBVM patients before thalidomide treatment collected from 2003 to 2020 together with 42 normal sera. In addition, paired serum samples from 32 SBVM patients treated with or without thalidomide were also collected in the Cohort 1. The course of treatment was 4 months and the follow-up time was 12 months. The detailed clinical characteristics of patients in the Cohort 1 was shown in the Table S7.1. The cohort 2 consisted of 10 SBVM tissues and paired para-SBVM normal tissue from 10 different patients for measure of RNA and glucose level. The detailed clinical characteristics of patients in the Cohort 2 was shown in the Table S7.2. The cohort 3 consisted of 30 paraffin-embedded SBVM samples collected from patients in the Renji hospital who underwent surgery due to massive bleeding from 2000 to 2020 and 16 randomized selected non-SBVM samples. The detailed clinical characteristics of SBVM patients in the Cohort 3 was shown in the Table S7.3. The patients were presented informed agreement through the Biomedical Ethics Committee of Renji Hospital (2015-088k).

Cell cultivation

HUVECs were purchased from FuHeng Cell Center (Shanghai, China). According to the introduction, HUVECs were cultivated in the Endothelial Cell Medium (ECM) (ScienCell, USA) medium. Both cell lines were grown in an incubator in a 5% CO2 humidified environment at 37°C. For hypoxia treatment, cells were incubated in a hypoxia chamber under 1% O2 after transfection for 24 hours or directly. Cells were cultivated in the glucose-free ECM (GF-ECM) (1001-GF, ScienCell, USA) for glucose deprivation treatment after transfection for 24 hours or directly.

Lentivirus and plasmids construction and transfection

The control, control shRNA, HGDILnc1 shRNA adenovirus were created through Hanbio Biotechnology Co.Ltd (Shanghai, China). The control plasmid, HGDILnc1, ENO1, ALDOC, NeuroD1, HGDILnc1 F1/F2/F3, ENO1 overexpression plasmids, ENO1-K202R/K343R overexpressing plasmids were created through Shenggong Company (Shanghai, China). The siRNA of ENO1, ALDOC, NeuroD1 and the other 10 transcription factors were designed and constructed by Genepharma Technology (Shanghai, China). The transfection of the plasmids into the cells was exerted using the FuGENE transfection reagent (Life Technologies, USA), and the transfection of siRNAs into the HUVEC cell was executed by implementing the DharmaFECT 1 siRNA transfection reagent (Thermo Scientific Dharmacon Inc., USA).

CCK8 assessment

By taking advantage of a plate containing 96 wells, two thousand cells were seeded into each well. According to the introduction of the Cell Counting Kit (Dojindo Molecular Technologies, Japan), cell viability was measured at the indicated time points (0h, 24h, 48h, 72h) after corresponding treatment. The optical density (OD) at 450 nm was evaluated by employing a VERSA Max microplate reader (MDS Analytical Technologies).

Matrigel Migration assessment

The assay of Matrigel migration was executed by employing transwells containing 24 wells (6-μm pore size; Millipore). 1×10^4 pre-processed cells suspended in 200 μL non-FBS cell cultivation milieu was increased to the upper chamber and 600 μL cultivation milieu comprising 20% FBS was increased to the lower chamber and cultivated at 37°C. At the considered time, cells that had migrated onto the lower surfaces of the membrane were fixed by utilizing 4% paraformaldehyde for 15 min and stained by utilizing crystallization violet for 20 min. Following an additional rinsing by utilizing PBS, the

membranes were air-dried and cell number on the membrane was measured under light microscopy at × 400 magnifications. As the average of five randomly selected fields, the number of migrated cells was expressed.

Tube formation assessment

HUVECs at density 1 × 10^4 per well were seeded to matrigel-coated (BD Biosciences, San Jose, CA) plates containing 24 wells with circumstance milieus of HUVEC cells. Following the incubation for 12 hours at 37 °C, by utilizing Calcein AM (Abcam, Cambridgeshire, UK) cells were stained and the endothelial tubule creation was recorded by applying an inverted confocal microscope. Branch points and cumulative tube length were appraised by utilizing the Image J computer program.

Animals and in vivo angiogenesis assessment

The procedures of animal experiments were confirmed through the Institutional Animal Care and Use Committee of Renji Hospital, School of Medicine, Shanghai Jiaotong University. Each 3 BALB/cAnN-nu (Nude) female mice (6 - 8 weeks of age) were chosen for each group without randomization and utilized for in vivo angiogenesis assessment. In vivo analysis of Matrigel was exerted as explained. After treatment, cells were subsequently trypsinized and around 5*10^5 cells were blended with 50 ml ECM and 350 ml ice-cold Matrigel (BD Biosciences). Next, the mixture was employed under the back skin of 8 week-old BALB/cAnN-nu (Nude) female mice. 10-14 days later, for histological assessment, Matrigel plugs were harvested. The CD31+ capillary density was evaluated as already described. Binding was done for all animals in this study.

Lactate production assessment

To evaluate the lactate productions, L-Lactate Assay kit (Colorimetric) was employed

(ab65331, Abcam, Cambridgeshire, UK) conforming to the protocols of the manufacturer. The transfected cells were planted into the plates of cell culture containing 96 wells and incubated at 37℃, during the night hours. Following the starvation for 2 hours, the supernatant was accumulated to evaluate the lactate productions. The levels of lactate productions were evaluated at 450 nm in a microplate reader.

Glucose uptake assessment

Glucose Uptake Colorimetric Assay Kit (ab136955, Abcam, Cambridgeshire, UK) was implemented to ascertain glucose uptake conforming to the protocols of the manufacturer. 1×10^4 pretreated cells were planted into the plates of cell culture containing 96 wells and incubated at 37℃, during the night hours. Following the incubation with 100μl Krebs-Ringer-Phosphate-HEPES for 40 minutes, the injection of 10μl 10mM 2-DG was done into each well and incubated for 20 minutes. And then cells were collected with the buffer of extraction and applied for determining the glucose uptake. The glucose uptake was evaluated through OD at 412 nm wavelength.

ATP production assessment

ATP Assay Kit (ab83355, Abcam, Cambridgeshire, UK) was employed to evaluate cellular ATP contents conforming to the protocols of the manufacturer. In brief, 100 μl of the cell lysate was blended with 100 μl of ATP reaction mix and then incubated for 30 min. With the aid of OD, absorbance was evaluated at 570 nm wavelength.

Assessment of ECAR

The Seahorse Extracellular Flux Analyzer XF96 (Seahorse Bioscience) was implemented to observe in vitro cells metabolic changes, conforming to the instructions

of the manufacturer. The seeding of the cells was fulfilled in a XF96-well plate at a density of 2×10^4 per well following the corresponding treatment and permitted to bind during the night hour, succeeded by serum starvation for 1 day. To detect the real-time glycolytic rate (ECAR), an indicator of net proton loss during glycolysis, the incubation of the cell was carried out by employing unbuffered milieu succeeded by a sequential injection of 1 μM oligomycin, 80mM 2-deoxyglucose, and 10mM glucose. ECAR assessments were normalized to the total content of protein and explained as mpH/min. Each specimen was ascertained in triplicate.

RNA assessment, extraction, and quantitative real-time PCR

The extraction of total RNA was accomplished through TRIzol reagent (Invitrogen), and 1 μg of total RNA was reverse transcribed by employing the PrimeScript RT Reagent Kit (Perfect Real-Time; Takara) and was evaluated by implementing a real-time quantitative PCR system. The amplified transcript level of each specific gene was normalized to ACTB. Subsequently, 0.5 μg of the total RNA was reverse transcribed into cDNA for miRNAs with the aid of a specific miRNA stem loop primer. The level of the amplified transcript for each specific gene was normalized to U6. The primers were acquired from Shenggong Company and showed in Table S7.

Western blots and antibodies

The extractions of the proteins were resolved with 8%-15% SDS-PAGE, transferred to PVDF membranes, and then with primary antibodies were probed. Peroxidase-conjugated rabbit antibody or anti-mouse (Kangchen, China) was employed as secondary antibody and the antigen-antibody reaction was visualized through enhanced chemiluminescence assessment. The commercial antibodies utilized for WB were given in the Table S8.

Rapid amplification of cDNA ends (RACE)

The products of RACE PCR were achieved employing a GeneRacer^{TM} Kit (Invitrogen, USA) and on a 1.2% agarose gel were separated. The products of the gel were extracted employing a kit of gel extraction (Omega, Norcross, GA, USA), cloned into a pGM-T vector (Shenggong, Shanghai, China) and sequenced. The specific 5′ RACE and 3′ RACE primers are given in Table S7.

Immunofluorescence

Cells growing on the coverslips were rinsed by utilizing PBS, fixed in 4% paraformaldehyde for 15 min and processed with 0.3% triton-x in PBS for 5 min. Cells were blocked with 3% BSA at ambient temperature for 1 h and then incubation was done by utilizing specific primary antibodies and correspondingly dyeconjugated secondary antibody. Ultimately, cells were counterstained with DAPI (Vector Laboratories, Bulingame, CA). With the aid of a laser-scanning confocal microscope (LSM-710, Zeiss, Germany) the images were obtained.

Fluorescent in situ hybridization (FISH)

HUVECs were washed cursorily in 1X PBS and subsequently fixed in 4% paraformaldehyde at ambient temperature for 10 min. Cells were permeabilized in 1X PBS comprising 0.5% Triton X-100 at 4°Cfor 5 min, subsequently rinsed in 13PBS for 5 min. 200 mL of Pre-hybridization Buffer was increased at 37°C washed for 30 min. Hybridization was conducted with a FISH probe at 37°C in a moist chamber in the dark during the night hours employing RiboTM Fluorescent In Situ Hybridization Kit (C10910, RiboBio, China). The slides were rinsed three times with Wash Buffer I (4X SSC with 0.1% Tween-20), once each with Wash Buffer II (2X SSC), Wash Buffer III (1X SSC) in the dark at 42°C for 5 min and once with 1X PBS at ambient temperature. Subsequently, the HUVECs were stained by utilizing DAPI for 10 min in the dark.

HGDILnc1-cy3 FISH probes (LNC1CM001, RiboBio, China) were designed and prepared through RiboBio Co., Ltd. Human U6 FISH probes (LNC110101, RiboBio, China) and human 18S FISH probes (LNC110102, RiboBio, China) were employed as the nuclear and cytoplasmic controls accordingly. The images were attained with laser-scanning confocal microscope (LSM-710, Zeiss, Germany).

RNA in situ hybridization (ISH)

For ISH, the MSTRG.6431.1 probe was labeled with digoxin (339501, QIAGEN, Germany). The paraffin-embedded SBVM section was dewaxed and hydrated and subsequently rinsed with PBS. The section was incubated with 15 μg/ml protease K for 40 min at 37°C. Following the rinsing with PBS, the section was dehydrated through a gradient of 70%, 96%, and 100% ethanol. Then, 50-100 μl of the solution of hybridization was increased to each section. The section was covered on 22 × 22 glass and hybridized for 1 h at 50°C. Then, the section was washed with 5× SSC, 1× SSC, and 0.2× SSC at 50°C and washed with PBS at room temperature. After washing the section was placed into the sealing solution and sealed for 15 min (1 ml sealing solution: 10× Roche sealing solution 100 μl/1× maleic acid buffer 900 μl). The sealant was dried with paper and incubated with probe fragments during the night hours at 4°C. Following the incubation, the slide was rinsed with TBST. The reaction NBT/BCIP reaction was incubated for 1 h in a wet box in the dark (diluted NBT/BCIP buffer: 20 μl Roche reagent buffer/1 ml NBT/BCIP diluent). The slide was washed with TBST, and 200 μl of red nuclear fixation dye was added for 1 min. Then, the slide was placed under running water for 10 min and dehydrated with alcohol. The slide was sealed with glycerin buffer. Imaging of vessels was observed after scanning of the sections using Digital Pathology Slide Scanner (KF-PRO-120, KFBIO, China). The staining intensity score was defined as low expression (negative or weak) or high expression (moderate or strong).

Immunohistochemistry

Paraffin-embedded SBVM tissue sections from the Renji hospital were used to perform an immunohistochemistry assay. Briefly, the sections were deparaffinized and rehydrated, and, after retrieval of antigen and blocking, incubated with the primary antibody. The primary antibodies employed in the present research was listed in the Supplementary Table 2. The section was incubated with peroxidase-labeled secondary antibody and stained by DAB reagent. Imaging of vessels was observed after scanning of the sections using Digital Pathology Slide Scanner (KF-PRO-120, KFBIO, China). The staining intensity score was defined as low expression (negative or weak) or high expression (moderate or strong).

Enzyme linked immunosorbent assay (ELISA)

Enzyme linked immunosorbent assay (ELISA) was accomplished as described in the HIF1α ELISA Kit (CSB-E12112h, CUSABIO, China) and ENO1 ELISA Kit (CSB-E17177h, CUSABIO, China). First, we added 100 μl of benchmark solution or sample to each well and incubate for 2 hours at 37°C. Subsequently, 100 μl of Biotin-antibody was added to each well and the plate was incubated for 60 min at 37°C. After that, we aspirated and washed the plate and added 100 μl of HRP-avidin to each well for 60 min at 37°C. Finally, we read the optical density (OD) at 450 nm after the addition of 90 μl of TMB substrate for 15-30 minutes and 50 μl of stop solution. The concentrations were evaluated based on a linear standard curve made by the concentration of standards and the mean value of OD.

Co-immunoprecipitation (co-IP)

The incubation of four micrograms normal rabbit IgG (A7016, Beyotime, China) or IP antibody, 40 μl suspended IP Matrix (sc45039, Santa Cruz, USA) and 500 μl PBS was executed for at least an hour on a rotator at 4 °C. Next, the solution was centrifuged and

rinsed three times by utilizing PBS in the presence of a mixture of protease inhibitor (phosphatase inhibitor, Protease inhibitors, PMSF; KangChen, Shanghai, China) and subsequently discard supernatant attentively. The transfected cells were lysed for 48h and then transferred to the matrix, and also incubated on a rotator at 4 °C during the night hours. Furthermore, the matrix was centrifuged and rinsed five times. SDS-PAGE sample loading buffer (P0015, Beyotime, China) was added to the immunoprecipitates succeeded by boiling at 100 °C for 10 min. The input proteins and IP were identified through western blot.

Chromatin immunoprecipitation (ChIP)

Magna HiSens Chromatin immunoprecipitation (ChIP) kit (17-10461, Millipore, New Bedford, MA) was employed conforming to the instructions of the manufacturer. HUVEC cells cultivated in a 10-cm dish were fixed with 16% formaldehyde to crosslink proteins to DNA. The extraction of chromatin was acquired from the cells by implementing corresponding antibodies and crosslinked DNA was sheared into 250–500 bp fragments. IgG and was employed as negative. PCR primers (Supplementary Table 1) were implemented to identify putative binding fragments.

RNA immunoprecipitation (RIP)

RNA Immunoprecipitation (RIP) assessments were executed by employing the Magna RIP Kit (17-700, Millipore, New Bedford, MA) conforming to the protocols of the manufacturer. Cells were provided utilizing RIP lysis buffer and the RNA-protein complexes were immunoprecipitated employing corresponding antibodies, and normal rabbit IgG. The purification of co-precipitated RNAs was exerted by implementing phenol:chloroform:isoamyl alcohol and exposed to reverse transcription-PCR or real-time PCR assessment. A control amplification was conducted on the input RNA prior to immunoprecipitation.

RNA Pull-down

Biotinylated lncRNAs were refolded in NEB enzyme buffer with RNase-out (Invitrogen, USA) at a final concentration of 200 ng/μL. The incubation of diluted RNAs was carried out for 10 min at 60 °C and gradually cooled to 4 °C. Aliquots of 2 μg of folded RNAs were employed for pull-down assessments. For preparation of cell lysates, HUVEC cells were harvested into 5 mL of buffer A [10 mM Tris·HCl, pH 7.0, 1.5 mM MgCl2, 10 mM KCl, 0.5 mM DTT, 1 mM PMSF, and protease inhibitor mixture (Roche Molecular Biochemicals, Mannheim, Germany)]. Cells were lysed through adding 0.25% Nonidet P-40 and incubating at 4 °C for 10 min. The lysates were centrifuged at 2,500 × g for 15 min, and the supernatant was eliminated. Pellets comprising the nuclear fractions were re-suspended in 3 mL of buffer C (25 mM Tris·HCl, pH 7.0, 0.5% Nonidet P-40, 150 mM KCl, 0.5 mM DTT, and protease inhibitor mixture) and mechanically sheared through homogenizing for 15–20 strokes. The specimens were cleared through centrifuging at 15,000 × g for 10 min. For the pull-down incubations, nuclear lysates containing 1 mg of protein were pre-cleared by utilizing streptavidin beads and subsequently incubated with 2 μg of biotinylated RNA and 40 μl of streptavidin beads at 4 °C for 2 hours. Beads were accumulated through centrifugation and rinsed with buffer C three times. The elution of RNA-associated proteins was fulfilled and then resolved through SDS/PAGE succeeded by silver staining (Bio-Rad, USA).

Luciferase assessment

To discover the effect of NEUROD1 on the HGDILnc1 promoter transcriptional performance, pGL3-HGDILnc1 promoter wild-type (WT) plasmid with related mut-type (MT) plasmid was designed and fabricated (GENEray Company, Shanghai, China). The reporter plasmids and the NEUROD1 siRNA or vector were transferred simultaneously into cells. Luciferase activity was evaluated by implementing a

FLUOstar device (11402ES60, YEASEN, Shanghai, China), with the Dual-Luciferase reporter assay system (Promega). The efficiency of transfection was normalized through separating the luciferase activity of the construct by the analogous Renilla luciferase activity.

Liquid Chromatography-Mass Spectrometry (LC-MS) Analysis, Database Search, and Protein Identification

To detect specific GClnc1, HGDILnc1 interactors, and Antisense HGDILnc1 pulled-down eluates were scrutinized, and the bands that were principally demonstrated only in the HGDILnc1 pulled-down specimen were selected. The bands were excised to conduct in-gel trypsin digestion and peptide extraction. For the identification of SUMOylation site of ENO1, and the bands that were mainly located in range of ENO1 protein size were chosen. The bands were excised to perform in-gel chymotrypsin digestion and the extraction of peptides. In brief, coomassie brilliant blue (CBB) dye on gel slices were eliminated by utilizing 50% acetonitrile (ACN)/50mM ammonium bicarbonate and dehydrated in 100% ACN. The gel slices then were reconstituted in an in-gel digestion buffer comprising sequencing-grade trypsin (V5113, Promega, USA) or sequencing-grade chymotrypsin (V1061, Promega, USA) overnight at 37 oC for protein digestion. The extraction of tryptic peptides was done from the gel pieces with 50 % ACN/0.1 % trifluoroacetic acid (TFA) and lyophilized.

The peptide specimens were scrutinized on Thermo Fisher LTQ Obitrap ETD mass spectrometry, in brief, loaded specimen onto the system of HPLC chromatography named Thermo Fisher Easy-nLC 1000 supplemented with a C18 colume (1.8mm, 0.15×1,00mm). Solvent A and B included 0.1% formic acid and 100% acetonitrile, respectively. The elution gradient was from 4% to 18% in 182 min, 18% to 90% in 13

min solvent B at a flow rate of 300nL/min. The analysis of mass spectrometry was conducted at the AIMSMASS Co., Ltd. (Shanghai, China) in the positive-ion mode with an automated data-dependent MS/MS assessment with complete scans (350-1600 m/z) procured employing FTMS at a mass resolution of 30,000 and the ten most intense precursor ions were chosen for MS/MS. The MS/MS was obtained by applying higher-energy collision dissociation at 35% collision energy at a mass resolution of 15,000.

Raw MS files were scrutinized through MaxQuant (version 1.5.2.8), the criterion employed for the analysis of data including trypsin as the protease with a maximum of two missed cleavages permitted. The mass tolerance for fragment ions and precursor ions was set to 4.5ppm and 20 ppm, accordingly. The search contained the modification of variables related to deamidation and methionine oxidation, and fixed modification of carbamidomethyl cysteine. Minimal length of the peptide was set to six amino acids and a maximum of two miscleavages was permitted. The false discovery rate (FDR) was set to 0.01 for identifications of protein and peptide.

RNA high-throughput sequencing and bioinformatics assessment

The isolation of total RNA was accomplished and purified by employing TRIzol reagent (Invitrogen, Carlsbad, CA, USA) according to the procedure of the manufacturer after silencing of HGDILnc1 or not. The amount of RNA and purity of each specimen was appraised by applying NanoDrop ND-1000 (NanoDrop, Wilmington, DE, USA). The integrity of RNA was evaluated through Bioanalyzer 2100 (Agilent, CA, USA) with RIN number >7.0, and approved by electrophoresis with denaturing agarose gel. Poly (A) RNA is purified from 1μg total RNA employing Dynabeads Oligo (dT) 25-61005 (Thermo Fisher, CA, USA) implementing two rounds of purification. Subsequently, the poly(A) RNA was fragmented into smaller pieces employing Magnesium RNA Fragmentation Module (e6150, NEB, USA) under 94℃ 5-7min. Next, the cleaved RNA fragments were reverse-transcribed to form the cDNA

via SuperScript™ II Reverse Transcriptase (1896649, Invitrogen, USA), which were then applied to synthesise U-labeled second-stranded DNAs with E. coli DNA polymerase I (m0209, NEB, USA), RNase H (m0297, NEB, USA) and dUTP Solution (R0133, Thermo Fisher, USA). An A-base is next increased to the blunt ends of each strand, preparing them for ligation to the indexed adapters. Each adapter includes a T-base overhang for ligating the adapter to the A-tailed fragmented DNA. Single- or dual-index adapters are ligated to the fragments, and size selection was carried out with AMPureXP beads. Following the heat-labile UDG enzyme (m0280, NEB, USA) processing of the U-labeled second-stranded DNAs, the ligated productions are amplified through PCR according to the following circumstances: primary denaturation for 3 min at 95°C; 8 cycles of denaturation for 15 sec at 98°C, annealing for 15 sec at 60°C, and extension for 30 sec at 72°C; and then final extension for 5 min at 72°C. The mean insert size for the final cDNA library was 300±50 bp. Ultimately, we conducted the 2×150bp paired-end sequencing (PE150) on an illumina Novaseq™ 6000 (LC-Bio Technology CO., Ltd., Hangzhou, China) conforming to the vendor's suggested protocols.

Fastp computer program (https://github.com/OpenGene/fastp) was implemented to eliminate the reads that included adaptor pollution, lower quality bases and unspecified bases with default parameters. Subsequently, the quality of the sequence was also confirmed by implementing fastp. We employed HISAT2 (https://ccb.jhu.edu/software/hisat2) to map reads to the reference genome of Homo sapiens GRCh38. The mapped reads of each specimen were collected by utilizing StringTie (https://ccb.jhu.edu/software/stringtie) with default parameters. Subsequently, all transcriptomes from all specimens were appeared to reconstruct a thorough transcriptome by applying gffcompare (https://github.com/gpertea/gffcompare/). Following the generation of the last transcriptome, StringTie was utilized to evaluate the levels of expression for all transcripts. StringTie was employed to execute the level

of expression for mRNAs through the calculation of FPKM (FPKM = [total_exon_fragments / mapped_reads(millions) × exon_length(kB)]). The differentially expressed mRNAs were chosen with fold change > 2 or fold change < 0.5 and with parametric F-test in comparison with the nested linear models (p value < 0.05) by R package edgeR (https://bioconductor.org/packages/release/bioc/html/edgeR.html). The data of the RNA sequence has been deposited in NCBIs Gene Expression Omnibus (GEO) and are accessible through GEO Series accession number GSE186473.

Microarray Information and analysis

The Agilent Human lncRNA Microarray 2019 (4*180k, Design ID:086188) was employed in the current assessment. After extraction and quantification of total RNA and assessment of RNA integrity, the labeling of the sample, hybridization of microarray and rinsing were conducted conforming to the standard protocols of the manufacturer. In brief, total RNAs were transcribed to double-strand cDNA, subsequently, synthesized into cRNA and labeled with Cyanine-3-CTP. The labeled cRNAs were hybridized onto the microarray. Following the rinsing, the arrays were scanned via the Agilent Scanner G2505C (Agilent Technologies). Feature Extraction computer program (version10.7.1.1, Agilent Technologies) was applied to scrutinize the images of the array to obtain raw data. To begin with, the raw data was normalized by utilizing the quantile algorithm. Differentially expressed genes were next characterized via fold change and also P value assessed with t-test. The threshold set for up- and down-regulated genes was a fold change>= 2.0 and a P value<= 0.05. The data of the Agilent Human lncRNA Microarray has been deposited in NCBIs Gene Expression Omnibus (GEO) and are accessible through GEO Series accession number GSE186474.

Statistical investigations

The statistical assessments were conducted employing GraphPad. The data was assessed whether they were normally distributed with the One-Sample Kolmogorov–Smirnov assessment. If the data were normally distributed and the variation between groups were comparable, the comparisons of assessments data between the two groups were executed implementing Student' t-test. The comparisons among three or more groups were initially exerted through one-way analysis of variance (ANOVA) assessments if the variation between groups were comparable. If the outcomes demonstrated considerable discrepancy, the analysis of Student Newman Keuls was employed to examine the differences between the two groups. For the clinicopathologic assessment, the Chi-square assay or Fisher exact assay (two-sided) were accomplished. All p-values were two-sided unless otherwise determined.

Table S1 significant different expression of mRNA after hypoxia treatment compared to controls.

Gene	logFC	AveExpr	t	P.Value	adj.P.Val	B
HSD17B2	3.464033	10.47816	45.0223	1.66E-08	0.00034	8.760261
SPAG4	2.8387	9.593727	36.68043	5.33E-08	0.000393	8.256367
ANKRD37	3.277741	10.85477	32.62908	1.04E-07	0.000393	7.908915
CLDN3	3.622782	8.261054	32.15692	1.13E-07	0.000393	7.862513
NOG	2.46529	9.664616	32.01477	1.16E-07	0.000393	7.848271
NPTX1	4.626304	6.274846	29.86931	1.71E-07	0.000393	7.616901
VEGFA	2.722522	10.04916	29.51361	1.83E-07	0.000393	7.575348
AK4	2.681546	9.1898	29.48576	1.84E-07	0.000393	7.572054
P4HA1	2.213634	10.5156	29.32748	1.90E-07	0.000393	7.553215
TMEM158	3.635404	13.64587	29.27889	1.92E-07	0.000393	7.547392
EGLN3	4.965124	6.007153	27.61173	2.68E-07	0.000498	7.335804
LOX	2.544888	13.26273	27.19233	2.92E-07	0.000498	7.278743
ADAMTS15	1.74113	6.683101	23.84078	6.15E-07	0.000854	6.75799
NMU	3.332934	5.269127	23.58848	6.53E-07	0.000854	6.713552
MEGF6	1.750103	11.08927	23.46284	6.73E-07	0.000854	6.691119
ADORA2A	2.309964	8.325483	23.27597	7.05E-07	0.000854	6.657371
RAB17	2.949843	7.30821	23.24926	7.09E-07	0.000854	6.65251
ADM	2.008385	14.0741	22.69479	8.13E-07	0.000888	6.549416
HMOX1	-2.14513	13.84458	-22.6361	8.25E-07	0.000888	6.53825
FABP4	-1.8698	9.274952	-21.7779	1.03E-06	0.001018	6.369389
INHBA	1.763252	10.00427	21.49926	1.10E-06	0.001018	6.312199
APLN	1.681869	12.62331	21.27706	1.17E-06	0.001018	6.265716
SYTL2	3.268728	4.736417	21.26499	1.17E-06	0.001018	6.263169
ADSSL1	2.109805	8.724219	21.20346	1.19E-06	0.001018	6.250148
STC2	1.707892	8.299094	21.03177	1.25E-06	0.001023	6.213488
ENO2	1.893405	12.31809	20.8234	1.32E-06	0.00104	6.16835
ANGPT2	1.539971	8.697005	20.19447	1.57E-06	0.001131	6.027642
STC1	1.99289	6.244911	20.0939	1.62E-06	0.001131	6.004503
ERRFI1	2.014712	10.42292	20.0205	1.65E-06	0.001131	5.9875
PLOD2	1.56529	13.041	19.7924	1.76E-06	0.001131	5.934034
SLC2A1	1.402732	9.035908	19.62888	1.85E-06	0.001131	5.895117
CALHM6	2.154176	8.319076	19.58174	1.87E-06	0.001131	5.883803
BHLHE40	1.820308	9.474874	19.48253	1.92E-06	0.001131	5.859857
BNIP3L	1.760102	11.20795	19.42223	1.96E-06	0.001131	5.845212
NDRG1	2.642035	10.63824	19.38827	1.98E-06	0.001131	5.836933
BNIP3	1.910318	9.241442	19.34675	2.00E-06	0.001131	5.82678
GALNT15	3.965421	6.107343	19.27308	2.05E-06	0.001131	5.808687
TRAM1L1	-1.70657	7.789485	-19.0337	2.19E-06	0.001182	5.749155
EDN2	3.63549	7.26524	18.66641	2.45E-06	0.001281	5.655575
PPP1R3G	2.726458	10.06823	18.5242	2.56E-06	0.001281	5.618598
NNAT	2.222007	12.65929	18.43672	2.63E-06	0.001281	5.595642
RASSF4	1.870179	8.797999	18.43321	2.63E-06	0.001281	5.594717
AQP1	-2.79864	7.108505	-18.3137	2.73E-06	0.001298	5.563084
LOXL2	1.247896	14.27339	18.13587	2.88E-06	0.00134	5.515438
TNFSF15	1.391258	13.83629	17.91579	3.09E-06	0.001403	5.455514
PPP1R13L	1.313847	11.53384	17.77199	3.23E-06	0.001422	5.415777

DUSP6	1.663628	8.300321	17.71455	3.29E-06	0.001422	5.39977
SLC7A11	-1.24794	9.71412	-17.4984	3.52E-06	0.001422	5.338851
IFIT3	-1.64711	8.891477	-17.4951	3.53E-06	0.001422	5.337924
Z83844.1	1.927584	5.873891	17.43516	3.60E-06	0.001422	5.320834
ARRDC3	1.806258	10.39904	17.42659	3.61E-06	0.001422	5.318387
SDCBP2	1.58275	11.60737	17.4197	3.61E-06	0.001422	5.316413
PDLIM2	1.113382	1.648117	17.12383	3.98E-06	0.001536	5.2307
PLAT	1.389836	12.08584	16.24044	5.35E-06	0.002025	4.96177
SLC2A14	1.362352	9.701011	16.14694	5.53E-06	0.002025	4.93211
ZNF395	1.240856	11.74039	16.14155	5.54E-06	0.002025	4.930393
FAM26F	2.31151	7.836056	15.58048	6.76E-06	0.002425	4.747228
CDCP1	2.175789	7.421616	15.20034	7.76E-06	0.002737	4.617964
SLAMF9	2.1372	5.522241	15.01243	8.32E-06	0.002884	4.55245
TMEM45A	1.952936	8.643202	14.88715	8.71E-06	0.002972	4.508167
KDM3A	1.364417	7.93084	14.77934	9.07E-06	0.003036	4.469662
APOLD1	1.042891	11.51664	14.74298	9.20E-06	0.003036	4.45659
SLC2A3	1.430785	8.720797	14.52449	1.00E-05	0.003248	4.37715
SLC30A3	-1.4194	10.06678	-14.3651	1.06E-05	0.0034	4.31819
AKR1B10	-1.80528	6.453478	-14.2409	1.12E-05	0.003498	4.271683
GPR98	1.522433	6.128334	14.21383	1.13E-05	0.003498	4.261477
P4HA2	1.428119	11.32063	14.02373	1.22E-05	0.003657	4.189071
TUBA3E	-1.40785	3.287005	-14.0072	1.22E-05	0.003657	4.182721
NGF	1.31264	9.041382	13.95393	1.25E-05	0.003657	4.162164
ADRA1D	2.402252	5.984817	13.95247	1.25E-05	0.003657	4.161602
RAB3IL1	-1.48237	9.555603	-13.8361	1.31E-05	0.003745	4.116347
ST8SIA6	2.731765	4.526964	13.79013	1.34E-05	0.003745	4.098352
NAA11	-2.12176	3.005429	-13.7889	1.34E-05	0.003745	4.097885
NARF	1.057923	12.94821	13.57191	1.46E-05	0.004035	4.011825
ASB11	1.144766	1.679514	13.4209	1.55E-05	0.004237	3.950916
SDSL	-1.19107	12.27531	-13.3029	1.63E-05	0.004334	3.902716
AKR1B15	-1.28777	6.118929	-13.1695	1.72E-05	0.004519	3.847585
FGF16	1.577499	6.681694	13.13232	1.75E-05	0.004519	3.8321
HIF3A	1.683092	6.254191	13.04144	1.82E-05	0.004571	3.794009
ZFP57	1.564829	2.021206	13.02735	1.83E-05	0.004571	3.788072
TGFBI	1.114065	14.30289	12.98015	1.87E-05	0.004608	3.768132
SMAD7	1.029267	10.84327	12.77157	2.04E-05	0.004777	3.678941
GOLGA8B	1.363869	7.790587	12.76068	2.05E-05	0.004777	3.674236
NPR3	3.607356	4.43187	12.70691	2.10E-05	0.004782	3.65093
DDIT4	1.865838	14.17381	12.67037	2.14E-05	0.004805	3.635025
AL445524.2	1.131982	9.132603	12.56414	2.24E-05	0.004915	3.588463
PNMA2	1.177592	11.98473	12.55662	2.25E-05	0.004915	3.585145
HOXD1	1.268463	7.67695	12.54509	2.26E-05	0.004915	3.580058
GRIN3B	1.516842	7.616688	12.49005	2.31E-05	0.004931	3.555701
GYPC	1.574233	5.914899	12.41867	2.39E-05	0.005038	3.523921
SIRPB2	1.216433	9.671468	12.32892	2.49E-05	0.005166	3.483642
PLAC8	1.317872	13.16438	12.31696	2.50E-05	0.005166	3.47825
SLC22A4	-1.11196	9.77777	-12.2483	2.58E-05	0.005234	3.447148
HIST1H2BD	1.114886	9.308835	12.24298	2.58E-05	0.005234	3.444749

PRDM13	-2.11351	3.058635	-12.1823	2.66E-05	0.005234	3.417068
ANG	1.012158	9.581899	12.17875	2.66E-05	0.005234	3.41546
TNFSF18	1.12764	10.92059	12.13821	2.71E-05	0.00528	3.396883
CLCA4	-1.26087	2.204769	-12.0863	2.77E-05	0.005356	3.372979
MYCT1	1.013338	12.39055	11.95441	2.95E-05	0.005585	3.31172
ITGA11	1.590182	5.381297	11.76742	3.22E-05	0.005982	3.223478
HIST1H2BA	-1.13575	5.279954	-11.728	3.28E-05	0.006039	3.204664
SIRPB1	1.005622	7.148454	11.65531	3.39E-05	0.006138	3.169777
TNFRSF10D	1.062156	10.88851	11.4833	3.68E-05	0.006308	3.086206
NTN4	1.094358	11.68078	11.46773	3.71E-05	0.006308	3.078574
CKB	1.144396	7.31498	11.25274	4.11E-05	0.006843	2.971897
UGCG	1.098238	14.76788	11.18974	4.24E-05	0.006944	2.940189
PGM1	1.447628	8.800726	11.09099	4.45E-05	0.007215	2.890083
DNAH5	2.21159	2.126748	11.08014	4.48E-05	0.007215	2.884546
TFRC	-1.26258	14.52345	-11.0573	4.53E-05	0.00724	2.872888
C4orf47	1.497736	6.733328	11.00734	4.64E-05	0.007307	2.847242
IFIT2	-1.27127	6.075889	-10.9641	4.74E-05	0.007307	2.824949
PLAU	1.066746	10.31005	10.94398	4.79E-05	0.007307	2.81454
FOLH1B	1.94313	5.780761	10.91254	4.87E-05	0.007307	2.798235
APCDD1L	2.100513	5.550271	10.90271	4.89E-05	0.007307	2.793128
FYB	-1.4224	1.91471	-10.8662	4.98E-05	0.00738	2.774104
GIMAP7	-1.04157	9.369003	-10.8544	5.01E-05	0.00738	2.767969
GJB2	1.939229	6.01148	10.81937	5.10E-05	0.007458	2.749606
C1orf110	1.047128	10.11603	10.72843	5.34E-05	0.007603	2.701683
RALGPS2	1.025532	12.63457	10.71491	5.38E-05	0.007603	2.694522
SLC22A23	1.256702	6.329965	10.6789	5.48E-05	0.007603	2.675395
LBH	1.438993	6.389115	10.63057	5.62E-05	0.007603	2.649609
SCUBE1	1.217662	4.471842	10.58727	5.75E-05	0.007603	2.626391
ZNF727	1.145315	3.753961	10.58487	5.75E-05	0.007603	2.625106
AC090517.4	2.099334	2.073083	10.57399	5.79E-05	0.007603	2.619252
C1QL4	1.165693	7.545292	10.55796	5.83E-05	0.007603	2.610623
AQP10	1.003187	5.150881	10.49834	6.02E-05	0.007793	2.578385
PGM5	1.036937	7.972618	10.46764	6.11E-05	0.007819	2.56171
PDE1B	-2.15909	2.39618	-10.4531	6.16E-05	0.00783	2.553817
DUSP1	1.095186	10.2812	10.43334	6.22E-05	0.007862	2.543011
INHBB	1.434507	7.353933	10.40866	6.31E-05	0.007916	2.529515
MYCN	-1.20025	4.96451	-10.3881	6.37E-05	0.007953	2.518269
TAGLN	1.222432	9.629877	10.25414	6.84E-05	0.008187	2.444214
TSPAN18	1.164875	8.683715	10.17537	7.14E-05	0.008213	2.400187
CDH11	1.070932	7.293387	10.15199	7.23E-05	0.008213	2.387045
UBD	-1.58326	6.608093	-10.0721	7.54E-05	0.008213	2.341906
MAGI1	1.029767	9.241988	10.03856	7.68E-05	0.008317	2.322828
IDO1	-2.26993	4.646755	-9.98541	7.91E-05	0.008471	2.292469
GUCY1B3	-1.50751	3.601006	-9.94691	8.08E-05	0.008561	2.270365
CNTN1	1.080576	1.626696	9.925976	8.17E-05	0.008571	2.258309
CD82	1.147492	9.953857	9.886444	8.35E-05	0.008583	2.235468
EEF1A2	1.248432	10.19184	9.838381	8.57E-05	0.008691	2.207564
STARD6	-1.17236	4.473641	-9.83671	8.58E-05	0.008691	2.206593

CHRNB1	1.396422	7.467052	9.81781	8.67E-05	0.008739	2.195577
RNF39	1.671898	2.671906	9.785351	8.83E-05	0.008783	2.176607
MTL5	1.030793	7.074448	9.782298	8.84E-05	0.008783	2.17482
FST	-1.16121	8.066464	-9.74325	9.04E-05	0.008865	2.151903
SIM2	2.367198	2.208506	9.742825	9.04E-05	0.008865	2.151652
GIMAP1-GIMAP5	1.118675	11.1303	9.739589	9.05E-05	0.008865	2.149749
VLDLR	3.027257	4.938313	9.701459	9.25E-05	0.00897	2.127267
NPPB	1.565968	6.549604	9.673578	9.39E-05	0.009056	2.110768
VSTM1	1.62119	5.602455	9.619829	9.68E-05	0.009122	2.07882
ZNF704	1.094143	8.570992	9.613384	9.72E-05	0.009122	2.074976
HLA-DPB1	-1.30031	2.904916	-9.58373	9.88E-05	0.00915	2.057254
GIMAP5	1.634035	6.007199	9.442537	0.000107	0.009486	1.972084
IFIT1	-2.2286	9.404754	-9.41283	0.000109	0.009548	1.953992
WISP2	1.554583	5.746328	9.412827	0.000109	0.009548	1.953992
CDKN1C	1.017379	8.16387	9.408692	0.000109	0.009548	1.951469
WFDC10B	1.387829	4.120345	9.298189	0.000116	0.009924	1.883618
RAB40B	1.031963	4.860484	9.129474	0.000128	0.010428	1.778389
MAGED4	1.162137	8.278926	9.121324	0.000129	0.010428	1.773255
TCF7L1	1.415853	8.401753	9.119441	0.000129	0.010428	1.772068
TNFRSF1B	-1.09836	9.766683	-9.07579	0.000133	0.010493	1.744483
DOK7	-1.09943	8.75354	-9.0083	0.000138	0.01055	1.701567
PAEP	1.287881	1.792579	9.0037	0.000138	0.01055	1.698631
MEDAG	1.065166	9.25539	8.977293	0.000141	0.01055	1.68174
ZCCHC5	1.32485	5.808991	8.97631	0.000141	0.01055	1.68111
BACE2	1.501251	7.350341	8.96445	0.000142	0.01055	1.673507
SIRPD	2.015459	4.188011	8.964007	0.000142	0.01055	1.673222
CORIN	-1.51455	4.409398	-8.90611	0.000147	0.01069	1.635954
AKAP12	1.283184	14.85612	8.892456	0.000148	0.01069	1.627131
BCL2A1	1.375752	6.86016	8.889659	0.000148	0.01069	1.625321
PRB3	-1.01921	9.09711	-8.84003	0.000153	0.01085	1.593121
ELMOD1	-1.65533	7.10417	-8.83594	0.000153	0.01085	1.590458
FGF1	1.612364	6.743932	8.824518	0.000154	0.01087	1.583016
EDIL3	1.165954	9.780367	8.79657	0.000157	0.010951	1.564768
COL5A1	1.00971	11.41852	8.704778	0.000166	0.011436	1.50442
IGFBP5	1.547374	8.983475	8.667381	0.00017	0.01165	1.479651
C6orf223	1.283301	1.668157	8.648613	0.000172	0.01165	1.467179
UPB1	4.094258	4.144979	8.642836	0.000172	0.01165	1.463335
GJD4	1.802157	2.340978	8.57966	0.000179	0.011802	1.421126
TBC1D3B	1.008058	5.676492	8.552618	0.000182	0.011843	1.402964
HPGDS	2.067485	2.133964	8.537466	0.000184	0.011843	1.392763
YPEL1	1.094553	8.370638	8.510464	0.000187	0.011904	1.374538
CRTAC1	1.135401	7.419296	8.489589	0.00019	0.01199	1.360409
AMY2A	1.169698	3.960214	8.460447	0.000193	0.012089	1.340628
OAS2	-1.10826	8.410634	-8.39115	0.000202	0.012296	1.293316
PDGFRL	1.040556	7.628455	8.382565	0.000203	0.012296	1.287429
PPARG	1.279443	5.951305	8.325858	0.000211	0.012527	1.248387
LMNTD2	1.041416	8.919218	8.318896	0.000212	0.012546	1.243576

PTGES	-1.88982	2.369153	-8.31172	0.000213	0.012567	1.238616
CXorf58	1.512282	1.990722	8.300844	0.000214	0.012609	1.231082
BGN	1.136884	11.73218	8.222844	0.000225	0.012924	1.176791
RSAD2	-2.26289	3.973807	-8.2068	0.000227	0.012924	1.165563
VCAM1	-1.42494	5.059256	-8.09254	0.000245	0.013347	1.084967
GPR88	-1.55844	2.037971	-8.07374	0.000248	0.013426	1.071602
CCDC64	-1.00795	3.231064	-8.06183	0.00025	0.013496	1.063117
NCKAP5	1.037835	7.627235	8.028858	0.000255	0.013571	1.039568
SGIP1	1.041652	9.906044	7.997773	0.000261	0.013682	1.017282
GDF5	1.474377	5.410378	7.95012	0.000269	0.013883	0.982955
MN1	1.176062	11.10691	7.932911	0.000272	0.01394	0.97051
TMEM98	1.094599	5.447766	7.879842	0.000282	0.014051	0.93197
TIMP3	1.103216	6.406833	7.837005	0.00029	0.014249	0.90068
IFI44L	-2.00862	5.558661	-7.81391	0.000295	0.014276	0.883742
MAP3K9	-1.47603	2.113541	-7.78297	0.000301	0.014414	0.860981
CCDC89	-1.76927	2.104651	-7.7593	0.000306	0.014498	0.843502
SNX33	1.31622	6.465924	7.739067	0.00031	0.014596	0.828528
AL355987.3	1.339644	6.637541	7.721992	0.000314	0.014596	0.81586
FAM83G	-1.06707	8.932657	-7.71799	0.000315	0.014596	0.812887
CHST2	1.016828	7.887761	7.700723	0.000319	0.014682	0.800045
OLFML2B	1.132571	2.790328	7.69738	0.000319	0.014682	0.797555
CSF3	-1.28496	7.200749	-7.61092	0.000339	0.014976	0.732811
KLHDC7A	-1.37966	7.288983	-7.58405	0.000345	0.014996	0.712547
CD48	1.348691	1.982554	7.543074	0.000355	0.015143	0.681522
CNTNAP2	-1.64244	2.1181	-7.51188	0.000363	0.015316	0.657794
MTRNR2L11	-1.23182	7.548585	-7.45041	0.000379	0.0157	0.610774
CCL23	-1.34857	5.792329	-7.43382	0.000383	0.01571	0.598029
CCDC27	-1.03268	5.330677	-7.42071	0.000387	0.015777	0.587928
TNNI3	1.293363	4.374053	7.401333	0.000392	0.015868	0.572979
SHC2	1.071064	7.447597	7.351962	0.000406	0.016054	0.534721
CCDC183	1.602837	5.773296	7.33639	0.000411	0.01617	0.522607
BCL2L12	-1.07907	7.780948	-7.27712	0.000429	0.016528	0.476274
ETDB	-1.25402	2.747711	-7.27624	0.000429	0.016528	0.475589
DPEP2	1.769225	2.118419	7.240984	0.00044	0.016705	0.447862
OPRL1	1.083088	8.535061	7.17134	0.000463	0.017218	0.392734
CLEC3B	1.119455	5.843415	7.153962	0.000469	0.017342	0.378903
OR7G3	-1.42926	4.025544	-7.12994	0.000477	0.017366	0.359736
EFNB3	1.094795	6.520137	7.097579	0.000488	0.017563	0.333819
IVL	-2.2768	3.972095	-7.02492	0.000515	0.018208	0.275246
PDK1	1.19894	6.063073	6.992722	0.000528	0.018236	0.249117
TM4SF4	-1.18798	8.549649	-6.98903	0.000529	0.018236	0.246114
PFKFB4	1.885872	4.313516	6.974884	0.000535	0.018236	0.234595
C1orf21	1.519416	4.534483	6.966753	0.000538	0.018236	0.227965
IFNLR1	-1.25859	5.590582	-6.92587	0.000555	0.018364	0.194522
GPR142	-1.06556	5.69123	-6.90493	0.000563	0.018504	0.177331
CENPS-CORT	-1.30483	1.921696	-6.87944	0.000574	0.018711	0.156337
OR7D4	1.908205	4.548306	6.869131	0.000579	0.018738	0.147824
ASB18	-1.1716	4.000564	-6.84822	0.000588	0.018886	0.130524

FBLN7	1.213581	7.374447	6.801046	0.000609	0.019204	0.091337
NKX2-2	-1.61985	2.017078	-6.78207	0.000618	0.019303	0.075501
DENND6B	1.049043	2.199238	6.769371	0.000624	0.019395	0.064889
HERC5	-1.12674	5.383361	-6.76208	0.000628	0.01943	0.058783
ADCYAP1	-1.43755	4.421955	-6.71184	0.000652	0.019831	0.016578
PTGIS	1.700034	7.145168	6.62251	0.000699	0.020664	-0.05915
IFI44	-1.18028	6.653671	-6.60159	0.00071	0.020856	-0.07701
SLIT1	1.057556	1.578382	6.599144	0.000712	0.02086	-0.07911
NRG2	-1.17751	6.6324	-6.58385	0.00072	0.020998	-0.0922
C19orf67	-1.60251	2.393964	-6.574	0.000726	0.021001	-0.10064
PLA2G4A	-1.372	5.739979	-6.57158	0.000727	0.021011	-0.10272
MXI1	1.003784	5.947138	6.566203	0.00073	0.021011	-0.10734
WDR64	-1.6329	4.116791	-6.54086	0.000745	0.021307	-0.12915
MX2	-1.57374	7.417144	-6.52017	0.000757	0.021441	-0.14699
CPT1B	1.034352	6.41953	6.502375	0.000768	0.02156	-0.16239
SLCO4A1	1.046465	8.752495	6.488432	0.000776	0.021578	-0.17447
CFAP161	-1.56843	2.530058	-6.46649	0.00079	0.021578	-0.19354
CLVS2	1.401951	1.779534	6.443161	0.000804	0.021716	-0.21387
VIP	1.030271	6.088871	6.411693	0.000825	0.021903	-0.24139
PROC	-1.59127	4.206969	-6.40209	0.000831	0.021944	-0.24981
LRRN2	-1.50673	2.0631	-6.38992	0.000839	0.021955	-0.2605
MRGPRX3	2.120672	2.874146	6.35995	0.000859	0.022177	-0.28689
SPINT2	1.191602	7.096956	6.292247	0.000908	0.022819	-0.34688
CXCL10	-2.01577	4.614833	-6.27744	0.000918	0.022974	-0.36008
PDE6A	1.129534	1.837167	6.273712	0.000921	0.022997	-0.3634
ELSPBP1	-1.53791	3.147713	-6.24917	0.00094	0.023056	-0.38534
GRAMD2	-1.36139	2.024386	-6.2149	0.000966	0.023427	-0.41608
CNGA1	1.22645	1.912823	6.185467	0.00099	0.023828	-0.44261
AL139142.2	1.423011	3.554369	6.181389	0.000993	0.023844	-0.44629
KIAA1524	-1.89209	2.749975	-6.17148	0.001001	0.023954	-0.45524
LYVE1	-1.56463	9.496664	-6.16159	0.001009	0.024072	-0.46419
NFATC2	1.17554	4.994622	6.139248	0.001028	0.02442	-0.48446
CABP1	1.069324	1.973361	6.112394	0.001051	0.024558	-0.50889
MX1	-1.20089	5.148848	-6.11083	0.001052	0.024558	-0.51032
FAM47E	-1.85674	2.091611	-6.08794	0.001073	0.024621	-0.53122
SLC45A1	1.107991	4.756631	6.021103	0.001134	0.02521	-0.59261
BPIFA2	1.214647	1.880202	6.019009	0.001136	0.02521	-0.59454
RTP3	1.586473	3.983129	5.992687	0.001161	0.025375	-0.61888
FIBIN	1.276805	1.659839	5.97885	0.001175	0.025375	-0.6317
KRT73	-1.19625	5.862883	-5.9772	0.001176	0.025375	-0.63323
UGT2A1	-1.00191	4.62295	-5.94527	0.001209	0.025705	-0.66293
ASAH2	1.046096	1.608132	5.897764	0.001258	0.026215	-0.70735
ALG12	1.156051	4.809496	5.886879	0.00127	0.026324	-0.71756
GTSF1L	-1.18893	6.417396	-5.87936	0.001278	0.026346	-0.72463
GPR18	-1.59926	2.950764	-5.86697	0.001291	0.026426	-0.73629
GUCY1A3	-1.35903	2.825465	-5.82483	0.001339	0.026716	-0.77609
TLE2	1.038391	6.765434	5.792873	0.001376	0.02705	-0.80642
COLQ	2.154428	3.908292	5.792847	0.001376	0.02705	-0.80644

SEMA4B	1.111791	6.490399	5.789917	0.00138	0.027065	-0.80923
RGMA	-1.08039	5.445007	-5.77941	0.001392	0.027207	-0.81923
PLD4	1.991644	2.273385	5.775975	0.001396	0.027262	-0.82251
ERBB4	-1.81249	2.29657	-5.75949	0.001416	0.027505	-0.83824
PRR15	-1.24681	7.073447	-5.75508	0.001422	0.027574	-0.84246
SP140	-1.60737	2.998665	-5.74309	0.001437	0.027756	-0.85392
RNF17	1.769759	2.616997	5.725322	0.001459	0.028081	-0.87096
NEURL3	-1.58995	3.65241	-5.70399	0.001486	0.028446	-0.89147
OAS1	-1.00815	9.166775	-5.70292	0.001488	0.028446	-0.89249
SLC39A5	1.025107	4.9722	5.655632	0.00155	0.029022	-0.93816
OR1S1	-1.58809	2.410272	-5.58643	0.001648	0.029579	-1.00552
LRRC25	-1.3652	2.351943	-5.58561	0.001649	0.029579	-1.00633
RAB33A	1.175968	7.617539	5.575578	0.001663	0.029741	-1.01614
RTP4	-1.40303	5.866726	-5.54685	0.001706	0.029959	-1.04433
GDF7	1.199194	1.661859	5.545371	0.001709	0.029959	-1.04579
SNAI2	1.218895	5.167253	5.490261	0.001795	0.030828	-1.10018
ZBTB8B	-1.78285	3.776259	-5.48032	0.001811	0.030902	-1.11003
ACP5	-2.18302	7.412682	-5.46949	0.001828	0.030997	-1.12078
UCMA	1.308835	2.142528	5.468237	0.001831	0.031007	-1.12203
FLT3LG	1.559321	2.155967	5.465162	0.001836	0.031032	-1.12509
FAM189A2	1.069924	5.566555	5.4579	0.001848	0.03109	-1.13231
GALNT5	2.333656	2.218347	5.444071	0.001871	0.031197	-1.14608
CLEC1B	-1.66233	4.931031	-5.44306	0.001872	0.0312	-1.1471
NAV1	1.26714	4.765512	5.439155	0.001879	0.031259	-1.15099
DOK2	1.329104	5.691398	5.395507	0.001955	0.03187	-1.19466
TRMT1	-1.5033	2.433424	-5.34847	0.00204	0.032664	-1.242
HERC6	-1.23382	6.197603	-5.34733	0.002042	0.032664	-1.24315
HS3ST5	1.869334	3.218709	5.323671	0.002087	0.032974	-1.26709
ADAMTS8	-1.1664	1.700065	-5.29939	0.002134	0.033458	-1.29173
MIA	1.306055	4.527076	5.292401	0.002148	0.033505	-1.29885
CMA1	-1.12531	3.333573	-5.28588	0.00216	0.033634	-1.30548
PLEKHB1	1.122122	4.432424	5.283129	0.002166	0.033652	-1.30829
ARX	1.128892	4.390212	5.281264	0.00217	0.033685	-1.31019
BPIFC	-1.72147	3.322869	-5.20862	0.002321	0.03457	-1.38459
CTSW	1.73969	1.941441	5.199629	0.00234	0.034753	-1.39385
DRD5	1.335992	2.825996	5.191572	0.002358	0.034835	-1.40215
S1PR5	-1.3295	2.022328	-5.15492	0.00244	0.035382	-1.44006
HEY2	-1.20903	8.519626	-5.14664	0.002459	0.03543	-1.44864
CHRNA1	1.158412	6.11305	5.142554	0.002468	0.035445	-1.45289
CCDC158	1.975853	2.402461	5.134107	0.002488	0.035574	-1.46167
OR2G2	1.793935	2.378547	5.09612	0.002578	0.036198	-1.50126
DNAH1	1.795003	3.062744	5.090089	0.002593	0.03622	-1.50757
ODF3B	1.031778	7.132211	5.035701	0.00273	0.037325	-1.56466
CPLX1	1.06917	4.423434	4.95675	0.002945	0.038847	-1.6483
C16orf89	-1.25166	6.558544	-4.95122	0.00296	0.038904	-1.65419
CT62	1.62401	2.596785	4.948293	0.002969	0.038964	-1.65731
DDIT4L	1.535585	4.760148	4.919447	0.003052	0.039707	-1.68813
KISS1R	2.025103	3.142551	4.904755	0.003096	0.039964	-1.70387

GIPC3	1.007806	5.560157	4.870941	0.0032	0.040407	-1.74022
LYZL4	1.837507	2.654449	4.869888	0.003203	0.040407	-1.74135
EVX1	1.930453	2.028653	4.867912	0.003209	0.040407	-1.74348
OVOL3	-1.12114	2.298645	-4.83369	0.003318	0.040954	-1.78046
AC007846.2	-1.12333	1.8852	-4.83357	0.003318	0.040954	-1.78059
FBLN2	1.117859	5.150255	4.833539	0.003318	0.040954	-1.78062
ASCL3	-1.28816	2.834138	-4.82238	0.003355	0.041238	-1.79272
ZNF716	1.556204	1.873509	4.813977	0.003383	0.041303	-1.80183
SH3D21	1.088955	6.837822	4.786743	0.003474	0.041546	-1.83146
CCND2	1.144583	4.124596	4.780238	0.003497	0.041599	-1.83855
KCNV1	1.254887	4.494458	4.764975	0.00355	0.041829	-1.85522
C11orf40	-1.37438	2.165805	-4.75491	0.003585	0.041988	-1.86623
SLC22A18AS	-1.11419	4.679711	-4.74772	0.003611	0.042172	-1.8741
LIPI	-1.06097	5.758688	-4.74555	0.003619	0.042191	-1.87647
PRKAG3	-1.27951	3.529152	-4.71649	0.003724	0.042802	-1.90838
NDUFA4L2	1.247862	3.950521	4.701631	0.00378	0.043142	-1.92475
PRG2	1.803976	3.055893	4.699215	0.003789	0.043147	-1.92741
FGF3	-1.37911	2.785203	-4.69918	0.003789	0.043147	-1.92746
CBX2	1.310182	9.280386	4.674538	0.003884	0.043694	-1.95467
RHD	1.159805	3.645683	4.661266	0.003936	0.043957	-1.96937
FAS	-1.07345	8.65245	-4.6471	0.003992	0.044288	-1.98509
CCDC79	1.298913	2.09821	4.645187	0.004	0.044288	-1.98721
KIF17	1.383295	3.730652	4.622146	0.004093	0.044706	-2.01285
TP63	-1.70205	2.020279	-4.60408	0.004169	0.045154	-2.03301
OR2L3	-2.01187	2.821836	-4.60252	0.004175	0.045164	-2.03474
OR12D2	1.160863	4.114683	4.592064	0.004219	0.045322	-2.04643
PPIAL4G	1.363675	2.301669	4.579363	0.004274	0.045597	-2.06066
C2orf57	1.023064	3.125316	4.555965	0.004377	0.046185	-2.08692
PEBP4	-1.14812	2.27169	-4.54545	0.004424	0.046447	-2.09874
FRMPD4	2.229981	5.655234	4.544515	0.004428	0.046447	-2.0998
IL17F	-1.41533	2.070603	-4.53454	0.004473	0.046656	-2.11104
PIWIL4	1.201886	3.931234	4.534474	0.004474	0.046656	-2.11111
SDR16C5	1.157721	1.854544	4.529019	0.004499	0.046799	-2.11727
SLC6A9	1.044144	3.908463	4.528468	0.004501	0.046799	-2.11789
FRAS1	1.095279	5.0345	4.524645	0.004519	0.046934	-2.1222
OPRPN	1.196033	1.677291	4.522809	0.004527	0.046951	-2.12428
SIAE	1.097048	5.21196	4.498895	0.004639	0.04743	-2.15132
MC5R	-1.18735	1.984079	-4.49765	0.004645	0.04743	-2.15274
OR8J3	1.017064	8.779919	4.458124	0.004838	0.04845	-2.19764
SDK1	1.087083	3.259103	4.44349	0.004911	0.048732	-2.21432
TSPO2	-1.63457	2.392393	-4.39818	0.005147	0.049822	-2.26619
CALCR	-1.08808	2.453937	-4.39295	0.005175	0.049952	-2.2722
IGHV2-5	-1.57266	2.574921	-4.3908	0.005187	0.050017	-2.27467
NBPF6	1.403535	2.147958	4.382375	0.005233	0.05022	-2.28435
CALY	1.539346	2.470915	4.379199	0.00525	0.050245	-2.28801
CIDEA	1.180741	6.137302	4.366402	0.00532	0.050673	-2.30275
TBC1D29	-1.20222	4.750646	-4.36423	0.005333	0.050703	-2.30525
OR2W3	1.097096	1.620945	4.359383	0.00536	0.050867	-2.31084

CAPN12	1.082408	3.873228	4.339813	0.00547	0.051312	-2.33346
DMGDH	1.072728	2.336507	4.332155	0.005515	0.051407	-2.34232
C3orf36	-1.7264	3.391578	-4.32831	0.005537	0.051521	-2.34678
ST18	1.452538	2.207218	4.311191	0.005637	0.05196	-2.36664
S100A7A	1.480619	1.942486	4.306311	0.005666	0.052086	-2.37231
MOGAT2	-1.092	6.814661	-4.28819	0.005776	0.052774	-2.39339
T	2.482475	2.877232	4.287157	0.005782	0.052792	-2.39459
GOLGA6L22	1.192025	1.621924	4.269828	0.005889	0.053297	-2.41481
VWA7	-1.25108	4.869012	-4.26641	0.00591	0.053438	-2.4188
OR2C1	1.519765	2.54482	4.265685	0.005915	0.053456	-2.41965
TAS1R1	1.692907	2.161548	4.239658	0.00608	0.054566	-2.45011
ACSS3	-1.24049	8.922816	-4.23488	0.006111	0.054604	-2.45572
DYDC2	-1.13532	4.620921	-4.22529	0.006174	0.054839	-2.46697
LUM	-1.33883	5.408506	-4.19952	0.006346	0.055512	-2.49729
PPL	1.552518	3.756494	4.191823	0.006398	0.055762	-2.50636
OR5A2	1.146411	3.464804	4.189912	0.006411	0.055847	-2.50862
ATP6V1G3	-1.88007	3.448172	-4.16347	0.006595	0.056705	-2.53987
TGIF2LX	-1.6717	1.95026	-4.16344	0.006596	0.056705	-2.5399
TMEM40	1.146568	3.236862	4.156719	0.006643	0.056829	-2.54787
GYS2	1.070177	1.613637	4.136153	0.006792	0.057381	-2.57227
AQPEP	1.098041	1.606317	4.085974	0.007171	0.058856	-2.63209
ITK	1.358264	1.93352	4.072212	0.007279	0.059222	-2.64856
TENM2	-1.19104	2.321261	-4.04296	0.007514	0.060298	-2.68368
AMER3	1.558653	2.214165	4.033101	0.007596	0.060523	-2.69554
SELL	-1.227	4.474594	-4.03146	0.007609	0.060551	-2.69751
CAMKV	1.059913	2.02275	4.025324	0.007661	0.060707	-2.70491
C13orf46	2.109988	2.606797	4.018082	0.007722	0.060859	-2.71364
TMEM130	-1.09255	2.925837	-4.01784	0.007724	0.060859	-2.71393
DEFB125	1.086835	1.983285	4.015364	0.007745	0.060961	-2.71692
GZMA	1.172654	5.373665	3.884155	0.008954	0.066047	-2.87657
RBMY1F	-1.52253	2.49543	-3.8831	0.008964	0.066059	-2.87786
NKX6-1	1.341729	1.771038	3.878334	0.009012	0.066167	-2.88371
KCTD16	1.065081	5.300924	3.856511	0.009235	0.06708	-2.91054
DEFB115	1.108597	1.722776	3.848893	0.009314	0.067342	-2.91992
SPINK4	1.152224	3.427088	3.847555	0.009328	0.067342	-2.92157
TCEB3C	1.007382	3.184418	3.846663	0.009337	0.067342	-2.92267
TBX15	1.527258	2.30387	3.837256	0.009436	0.067685	-2.93427
C1orf131	-1.2137	7.843965	-3.83682	0.009441	0.067685	-2.93481
OR10A6	-1.08676	1.941564	-3.8292	0.009522	0.067914	-2.94421
RBFOX1	1.118855	3.60217	3.826568	0.009551	0.068068	-2.94747
AC074143.1	1.414612	4.240866	3.822941	0.00959	0.068133	-2.95195
EREG	-1.03625	2.080869	-3.81286	0.009699	0.068601	-2.96442
AC127029.3	-1.15177	2.847052	-3.79972	0.009844	0.06916	-2.9807
MACROD2	1.701891	4.818088	3.793066	0.009918	0.069316	-2.98895
KRTAP10-4	1.513378	2.696114	3.780111	0.010064	0.07	-3.00503
OR10A3	1.12679	1.718295	3.769825	0.010182	0.070457	-3.01782
SSC4D	-1.32987	2.529026	-3.76626	0.010224	0.070627	-3.02226
MAFA	-1.64175	2.506641	-3.76145	0.01028	0.07087	-3.02825

HTR5A	1.031038	1.602958	3.761448	0.01028	0.07087	-3.02825
HIST1H4G	-1.58742	2.773725	-3.7476	0.010443	0.071554	-3.04551
TRAV8-6	1.476763	3.637696	3.745621	0.010466	0.071624	-3.04798
SEMG2	1.933697	3.551691	3.730344	0.01065	0.07233	-3.06705
DMRT3	-1.36824	2.052885	-3.71776	0.010804	0.072755	-3.0828
KHDRBS2	1.051205	2.138547	3.715816	0.010828	0.072808	-3.08523
LIF	1.453525	2.571864	3.715813	0.010828	0.072808	-3.08523
ALLC	1.402114	2.443857	3.706732	0.010941	0.073125	-3.09661
GREM2	-1.37579	3.979242	-3.70444	0.010969	0.073125	-3.09949
GOLGA6D	1.417257	2.603566	3.697352	0.011059	0.073365	-3.10838
THBS2	1.924714	3.26266	3.697158	0.011061	0.073365	-3.10862
FAM47A	1.751801	2.865846	3.696373	0.011071	0.073375	-3.1096
LHX2	1.407246	2.455612	3.690036	0.011152	0.073575	-3.11756
EFS	1.104827	2.404957	3.689235	0.011162	0.073575	-3.11857
VWC2	-1.20152	2.331055	-3.68481	0.011219	0.073741	-3.12413
CCDC129	1.779413	2.652913	3.680937	0.011269	0.073974	-3.129
OOSP4A	1.280639	2.828606	3.672525	0.011378	0.074477	-3.13958
DEFA3	-1.65026	6.828437	-3.66868	0.011428	0.074553	-3.14441
MRGPRX1	1.08222	2.865758	3.659048	0.011556	0.074813	-3.15655
ITGA8	1.69706	2.014142	3.656944	0.011584	0.074842	-3.15921
F5	-1.01843	3.145749	-3.64828	0.0117	0.075118	-3.17014
CR1	1.479736	2.280083	3.645765	0.011734	0.075288	-3.17331
HDAC7	1.378387	2.257055	3.632269	0.011918	0.07598	-3.19037
NOXO1	1.191986	2.914465	3.628513	0.01197	0.076063	-3.19512
HHLA2	1.02865	2.47993	3.614547	0.012165	0.076824	-3.2128
PCDHA13	1.941255	2.806946	3.605431	0.012294	0.077234	-3.22436
GPR111	-1.28114	2.52862	-3.57716	0.012704	0.078915	-3.26028
RGS7	1.511565	2.90237	3.572915	0.012767	0.079042	-3.26568
MAGEL2	1.014129	3.187998	3.568788	0.012828	0.079231	-3.27094
LOC339862	1.051045	4.86095	3.56533	0.01288	0.079368	-3.27534
TDRD15	-1.20501	1.808839	-3.56366	0.012905	0.079418	-3.27748
PRSS56	1.364951	6.530314	3.560721	0.012949	0.07959	-3.28122
DGKB	-1.35176	2.666365	-3.54045	0.01326	0.080617	-3.3071
L3MBTL4	1.007728	4.350171	3.533148	0.013373	0.08084	-3.31643
SERTM1	1.116398	3.445707	3.521731	0.013553	0.081429	-3.33104
CCL1	-1.68328	2.779385	-3.51883	0.013599	0.081558	-3.33475
MINAR1	1.087536	4.4554	3.506811	0.013793	0.082233	-3.35016
EFCAB6	-1.05777	6.99687	-3.49562	0.013975	0.082887	-3.36453
SNAP25	1.007199	3.925411	3.486094	0.014133	0.083353	-3.37677
VAV3	1.377066	4.402281	3.483466	0.014177	0.083452	-3.38015
KLK12	-1.08799	1.988407	-3.4759	0.014303	0.083982	-3.38988
PDZRN3	-1.25824	2.19284	-3.46594	0.014473	0.084448	-3.40271
LY6H	1.413918	2.224482	3.45564	0.01465	0.085141	-3.41599
SPATA22	1.017969	1.912804	3.384489	0.015942	0.089448	-3.50813
CNR1	-1.51184	3.829947	-3.37482	0.016127	0.090061	-3.52071
DEFB106B	-1.42034	3.253984	-3.35497	0.016514	0.091255	-3.54657
TF	1.084925	4.148222	3.344379	0.016725	0.091773	-3.56038
APOBEC2	-1.13449	3.497465	-3.33796	0.016855	0.092189	-3.56877

LY6G6F-LY6G6D	1.447186	4.143687	3.33703	0.016873	0.092235	-3.56998
ACKR1	-1.82294	2.35164	-3.32821	0.017053	0.092693	-3.5815
THEMIS	1.211366	4.753116	3.326832	0.017081	0.09272	-3.58331
GPR174	1.287567	1.73903	3.326534	0.017088	0.09272	-3.5837
GUCY1A1	-1.00366	2.588266	-3.29478	0.017754	0.094752	-3.62529
FCGR1B	-1.33839	2.072978	-3.29352	0.017781	0.094791	-3.62695
TRBV5-1	-1.6664	3.117935	-3.28763	0.017908	0.095251	-3.63468
CSPG5	-1.16041	7.182818	-3.28622	0.017938	0.09529	-3.63654
AC244517.10	-1.1487	2.290509	-3.28133	0.018045	0.095656	-3.64296
NKX2-8	-1.12807	2.143611	-3.26181	0.018476	0.09708	-3.66862
RGS9	1.698411	2.844175	3.261461	0.018484	0.09708	-3.66909
LRRC31	-1.05524	6.86625	-3.25997	0.018518	0.097138	-3.67105
SCGB1D2	1.343278	1.764332	3.243315	0.018896	0.098232	-3.693
SOX9	1.082911	3.723847	3.232233	0.019152	0.099085	-3.70762
GJD2	-1.10195	2.574921	-3.22792	0.019253	0.099387	-3.71332
TP53TG5	1.445694	3.242623	3.223364	0.01936	0.099734	-3.71933
ASPRV1	1.192306	2.870557	3.204985	0.019799	0.101022	-3.74364
PTH1R	1.126331	1.987643	3.204829	0.019802	0.101022	-3.74385
LRRC37A	-1.29036	2.854109	-3.19891	0.019946	0.101413	-3.75169
AGTR1	-1.08262	2.996449	-3.19254	0.020102	0.101915	-3.76013
SMIM2	-1.05542	3.176872	-3.1911	0.020138	0.101946	-3.76204
CLCA1	1.797718	3.087365	3.169277	0.020683	0.103374	-3.79099
SYNGR3	1.087918	5.745233	3.156024	0.021022	0.104294	-3.8086
ARRDC5	1.27084	3.021047	3.151931	0.021128	0.104481	-3.81405
LRRC39	1.310773	1.906925	3.14878	0.02121	0.10473	-3.81824
HEY1	-1.20439	3.259526	-3.14155	0.0214	0.105163	-3.82787
OR5B21	1.716095	3.205539	3.132428	0.021641	0.105791	-3.84001
OR3A3	1.378466	1.939145	3.12481	0.021845	0.106168	-3.85017
FAM65C	1.086068	3.76691	3.121584	0.021932	0.106271	-3.85447
TMPRSS2	1.073366	2.879391	3.12017	0.021971	0.106313	-3.85636
KRT1	1.41698	2.580433	3.118671	0.022011	0.106427	-3.85836
CEACAM16	1.517645	2.312869	3.117439	0.022045	0.106538	-3.86
KRTAP4-8	1.027677	2.491646	3.112383	0.022183	0.106991	-3.86675
GPR128	1.325455	2.589273	3.098109	0.022578	0.107899	-3.88582
GNG3	1.5101	2.791091	3.083985	0.022976	0.108803	-3.90471
TEX26	2.026128	2.966163	3.083452	0.022991	0.108818	-3.90543
CPED1	1.207986	3.642752	3.074758	0.02324	0.109472	-3.91707
DCDC2B	1.328294	2.714885	3.060049	0.023669	0.110573	-3.93678
SLCO2A1	-1.2967	2.127648	-3.05581	0.023794	0.111056	-3.94247
LDLRAD2	1.689942	3.294928	3.04413	0.024142	0.111861	-3.95815
OR6C4	-1.09317	2.555402	-3.02933	0.024591	0.11328	-3.97804
CD3EAP	1.018365	5.235436	3.028283	0.024624	0.113307	-3.97945
SEMA7A	1.021053	4.448354	3.019612	0.024892	0.114174	-3.99111
SYT10	-1.16822	2.625229	-3.01366	0.025077	0.114744	-3.99913
AADACL4	-1.04601	2.583594	-2.99953	0.025525	0.11606	-4.01817
NEUROD1	1.013778	1.886734	2.987763	0.025903	0.117083	-4.03403
CD38	1.051495	3.845088	2.985488	0.025977	0.117288	-4.0371

GCKR	1.677312	2.574189	2.977007	0.026255	0.118026	-4.04855
OCM2	1.192586	3.890854	2.976977	0.026256	0.118026	-4.04859
GUCY1A2	-1.37348	2.58819	-2.97571	0.026298	0.118162	-4.0503
AL160272.2	1.277684	2.468995	2.969169	0.026515	0.118641	-4.05913
AC024592.3	-1.20353	2.30524	-2.96549	0.026638	0.119078	-4.06411
SH2D1B	1.118455	3.656434	2.961086	0.026786	0.119466	-4.07006
THEGL	1.616732	3.529875	2.959247	0.026848	0.119615	-4.07255
ADAP1	-1.1544	3.135471	-2.95498	0.026992	0.119912	-4.07832
TRBV7-9	1.201581	1.77905	2.954973	0.026992	0.119912	-4.07833
ZIM3	1.146954	4.258519	2.945947	0.027301	0.120759	-4.09054
NEU4	-1.29145	2.590552	-2.93836	0.027563	0.121366	-4.10081
AL357673.1	1.366364	1.731065	2.930868	0.027825	0.122151	-4.11096
AFF2	1.246102	2.537998	2.930618	0.027833	0.122163	-4.1113
CXorf51B	1.020596	2.266857	2.925445	0.028016	0.122595	-4.11832
ECE2	1.039711	2.845398	2.921027	0.028172	0.12277	-4.12431
TRAPPC3L	-1.24015	2.76437	-2.92049	0.028191	0.122779	-4.12503
TRBV7-6	1.528251	2.45841	2.919213	0.028237	0.12281	-4.12677
KRTAP9-6	-1.37772	2.765243	-2.90594	0.028715	0.124063	-4.14478
OR4K17	1.086945	3.932203	2.903556	0.028802	0.124232	-4.14801
ZSCAN4	-1.01773	2.239591	-2.88504	0.029485	0.126034	-4.17318
LOR	1.648667	2.707655	2.88135	0.029623	0.126335	-4.17819
ATP13A4	1.067329	1.60826	2.875332	0.029851	0.126887	-4.18638
AKR1C4	1.729299	2.437934	2.872607	0.029954	0.127003	-4.19009
FNDC8	1.003763	2.950788	2.869526	0.030071	0.127283	-4.19428
MORN5	1.822396	2.516568	2.847062	0.030943	0.129519	-4.22488
KRT25	1.105831	3.571882	2.844692	0.031037	0.129739	-4.22811
MYO7A	1.31967	2.11122	2.834476	0.031443	0.130678	-4.24205
ACSM2A	-1.03013	2.481607	-2.82663	0.03176	0.131632	-4.25276
SLC22A3	1.465552	1.807523	2.819352	0.032056	0.132324	-4.26269
KRT5	-1.00104	1.873212	-2.81766	0.032126	0.132505	-4.26501
AL445989.1	1.529674	3.501813	2.814249	0.032266	0.132688	-4.26966
CASP14	1.380622	2.350278	2.808575	0.032501	0.133048	-4.27742
DPPA5	1.306016	2.885827	2.80763	0.03254	0.133167	-4.27871
ANO1	1.035753	3.279485	2.806218	0.032599	0.133274	-4.28064
ANKRD31	1.340384	2.047922	2.79808	0.03294	0.134072	-4.29176
NHLH2	1.284222	5.453854	2.794455	0.033093	0.134543	-4.29672
OR51A4	1.492088	2.625924	2.791338	0.033226	0.134867	-4.30099
PAQR9	1.055518	3.527935	2.779694	0.033725	0.136087	-4.31692
PPEF1	-1.09993	8.983387	-2.77002	0.034147	0.137294	-4.33017
AMELX	-1.34955	1.677174	-2.75723	0.034712	0.138511	-4.34769
ADGRB3	1.036057	2.677942	2.747592	0.035146	0.139619	-4.36091
ANTXRL	-1.12517	2.073088	-2.73632	0.03566	0.140889	-4.37638
PRDM8	1.453396	2.211727	2.73154	0.03588	0.141494	-4.38294
OR7A17	-1.04127	2.836594	-2.72432	0.036216	0.142262	-4.39286
PDCL2	1.14663	3.074706	2.715963	0.036608	0.143126	-4.40433
PLPPR1	1.563725	2.715584	2.706394	0.037063	0.144099	-4.41748
SPATA1	-1.24808	2.309773	-2.70554	0.037104	0.14419	-4.41865
WNT7A	1.022307	9.70113	2.69915	0.037412	0.144922	-4.42744

TRAT1	-1.00035	3.023334	-2.68774	0.037969	0.146065	-4.44315
NPS	-1.15026	3.152184	-2.67541	0.03858	0.147611	-4.46011
OR5B12	-1.02859	2.38071	-2.67025	0.038839	0.148188	-4.46722
DPP6	1.479569	3.263787	2.66834	0.038936	0.148306	-4.46984
IDO2	-1.40625	2.632861	-2.66782	0.038962	0.148378	-4.47056
PRR18	1.166754	3.611996	2.651266	0.039808	0.150454	-4.49337
MAGEB1	-1.23831	2.236914	-2.64506	0.040131	0.1513	-4.50192
CDH17	1.009094	3.316592	2.639513	0.040421	0.151985	-4.50957
APOBEC3A	-1.34876	2.245473	-2.63594	0.04061	0.152414	-4.5145
OR2H2	-1.29898	3.719853	-2.62622	0.041127	0.153772	-4.52791
MTLN	-1.34746	3.239562	-2.62122	0.041396	0.15426	-4.53481
HECW1	1.224044	2.746589	2.608119	0.042109	0.155783	-4.55289
PRAMEF4	1.409215	1.812943	2.60681	0.042181	0.155852	-4.5547
TRGV1	1.318542	3.378988	2.601052	0.042499	0.156435	-4.56265
PARD6B	-1.25177	2.333672	-2.59942	0.04259	0.156607	-4.56491
WFDC5	1.241666	4.995598	2.57452	0.043999	0.15976	-4.59932
RNF182	1.138989	3.302759	2.572239	0.044131	0.159994	-4.60247
OR10AD1	1.388503	3.586856	2.567895	0.044383	0.160509	-4.60847
CFAP44	1.023054	3.509591	2.554976	0.045141	0.161888	-4.62634
NOX5	1.244589	2.172695	2.553047	0.045255	0.162087	-4.62901
HRC	1.184839	2.803556	2.539795	0.046049	0.163585	-4.64735
AIF1	-1.20557	2.642063	-2.53782	0.046168	0.163782	-4.65008
IFNA5	1.098618	1.67862	2.52128	0.047183	0.166057	-4.67298
TBX2	1.111204	2.42698	2.511282	0.047808	0.167177	-4.68683
CFC1	1.476027	3.13634	2.510715	0.047843	0.167231	-4.68761
LOC100144595	1.130165	2.021045	2.50877	0.047966	0.16734	-4.6903
CALR3	1.225055	2.956538	2.485261	0.049475	0.170892	-4.72288

Table S2 significant different expression of mRNA after glucose deprivation compared to controls.

Gene	logFC	AveExpr	t	P.Value	adj.P.Val	B
MSMO1	1.500312	11.2172	22.64719	1.06E-06	0.007561	5.795352
TM7SF2	1.958381	9.365773	22.56162	1.09E-06	0.007561	5.782969
CXCL13	1.533383	1.837828	21.60967	1.38E-06	0.007561	5.638787
COLQ	1.940565	3.80136	20.15751	2.02E-06	0.007561	5.39393
SH2D5	1.540058	9.756061	20.05252	2.08E-06	0.007561	5.374949
OVOL3	-1.78532	1.966555	-19.8234	2.22E-06	0.007561	5.332882
LDLR	1.221553	9.149798	18.09624	3.66E-06	0.00887	4.9852
ZNF716	1.753492	1.972153	17.91279	3.87E-06	0.00887	4.944821
LOC79999	1.193893	8.2778	17.85975	3.93E-06	0.00887	4.933013
ACAT2	1.365095	13.70753	17.5461	4.33E-06	0.00887	4.861943
C14orf1	1.080791	12.29567	16.73289	5.62E-06	0.009874	4.667251
PNPLA3	1.532699	7.662876	16.64319	5.79E-06	0.009874	4.644809
FOXS1	1.269488	8.92906	16.3661	6.35E-06	0.009992	4.574207
FABP3	2.957048	5.777199	16.08341	6.98E-06	0.010206	4.500136
FAM13A	-1.03544	5.579762	-14.8707	1.07E-05	0.012893	4.157219
OR10Z1	1.120577	1.762389	14.86232	1.07E-05	0.012893	4.154684
CDC25A	-1.02438	7.320389	-14.7988	1.10E-05	0.012893	4.135515
RAB3IL1	1.021764	10.80767	14.49328	1.23E-05	0.012893	4.041448
CYP51A1	1.213513	10.9615	13.8541	1.58E-05	0.012893	3.834647
SC5D	1.05157	12.04741	13.776	1.62E-05	0.012893	3.808402
S100A7L2	1.403502	1.777465	13.72149	1.66E-05	0.012893	3.789955
INSIG1	1.643854	11.58264	13.70787	1.67E-05	0.012893	3.785329
SCD	1.349115	10.56101	13.56062	1.77E-05	0.012893	3.734885
TCF4	-1.06755	5.592805	-13.4796	1.83E-05	0.012893	3.706799
SQLE	1.254082	12.99613	13.30913	1.96E-05	0.012893	3.64686
DOK2	2.095897	6.074794	13.11231	2.12E-05	0.012893	3.57628
OR8J3	1.20235	8.872562	13.09255	2.14E-05	0.012893	3.569109
ZNF681	-1.19584	3.735101	-12.9796	2.24E-05	0.012893	3.527824
CA2	-1.20278	7.012551	-12.9768	2.25E-05	0.012893	3.526805
NPTX1	1.472324	4.697856	12.8416	2.38E-05	0.012893	3.476695
MKI67	-1.00276	8.232993	-12.7432	2.48E-05	0.012893	3.439777
MIER2	-1.04623	5.385262	-12.6541	2.57E-05	0.012893	3.405974
MYCBP	-1.05472	9.916922	-12.6382	2.59E-05	0.012893	3.399883
ANGPT1	-1.92154	4.816365	-12.6331	2.60E-05	0.012893	3.397954
MUC12	-1.25932	1.815232	-12.6027	2.63E-05	0.012893	3.386313
FAM71E1	1.236813	8.602949	12.53536	2.71E-05	0.012893	3.360409
SREBF2	1.045902	9.025359	12.37083	2.91E-05	0.013007	3.296291
AGMO	-1.31977	7.708661	-12.273	3.04E-05	0.013007	3.257587
MEST	-1.40808	9.197336	-12.1875	3.15E-05	0.013099	3.223426

FRMPD4	2.865152	5.972819	12.15438	3.20E-05	0.013099	3.210124
ITK	1.656963	2.08287	11.86933	3.64E-05	0.013361	3.093401
FAM95C	-1.42079	5.439155	-11.8041	3.75E-05	0.013361	3.066174
PSG8	1.201253	8.866507	11.76472	3.82E-05	0.013361	3.049614
NEB	-1.0371	7.0734	-11.5222	4.27E-05	0.014085	2.946093
DHRS2	-1.30756	7.788302	-11.412	4.49E-05	0.01431	2.898076
LRRC37A	-2.13679	2.430896	-11.3842	4.55E-05	0.01431	2.885843
OPLAH	1.238928	8.191673	11.35539	4.62E-05	0.01431	2.873166
GRXCR1	1.504747	1.949711	10.87896	5.81E-05	0.0145	2.657015
MGARP	-1.12936	13.1057	-10.8664	5.84E-05	0.0145	2.651153
TNFSF10	-1.03407	10.3584	-10.8535	5.88E-05	0.0145	2.645144
CEND1	-1.44884	6.501167	-10.8371	5.93E-05	0.0145	2.637455
ACMSD	1.360449	3.066638	10.74402	6.21E-05	0.0145	2.593576
FOLR3	1.045209	4.86394	10.69961	6.35E-05	0.0145	2.572476
HAPLN1	-1.42127	6.440694	-10.6932	6.37E-05	0.0145	2.569437
CIZ1	-1.32473	6.644002	-10.6693	6.45E-05	0.0145	2.55803
PCDHAC1	1.015947	5.184958	10.66467	6.46E-05	0.0145	2.5558
GGT6	1.021511	2.058234	10.624	6.59E-05	0.0145	2.536297
TRBV12-3	-1.03861	6.776751	-10.5105	6.98E-05	0.0145	2.481359
TUBA3E	-1.9453	3.018279	-10.4999	7.02E-05	0.0145	2.47621
HLA-DPB1	-1.39384	2.858151	-10.4764	7.11E-05	0.0145	2.464726
FAM111B	-1.08702	8.459847	-10.4722	7.12E-05	0.0145	2.462664
MSH4	-1.1607	1.658752	-10.4658	7.14E-05	0.0145	2.45953
PDIA4	1.067303	11.69869	10.36447	7.52E-05	0.014957	2.409616
CCL23	-1.41964	5.756797	-10.3603	7.54E-05	0.014957	2.407547
PLA2G3	1.220288	4.813873	10.28947	7.82E-05	0.014957	2.372272
PRKG2	2.261763	3.224057	10.23323	8.05E-05	0.01513	2.344044
HIST1H2BJ	-1.03019	7.399531	-10.2318	8.06E-05	0.01513	2.34331
HES6	1.03718	10.55793	10.15465	8.39E-05	0.015397	2.304288
CYP26B1	-2.5751	4.909946	-10.1463	8.43E-05	0.015397	2.30004
ERVFRD-1	1.38043	1.851	10.10131	8.63E-05	0.015402	2.277089
E2F2	-1.29525	6.068074	-10.0349	8.94E-05	0.015402	2.242992
GDF3	-1.63252	6.977092	-9.86961	9.76E-05	0.015802	2.156862
GJB4	1.514916	7.596674	9.847054	9.88E-05	0.015802	2.144977
PPIAL4G	1.698013	2.468838	9.846687	9.88E-05	0.015802	2.144783
AL031777.3	-1.00278	5.720321	-9.8054	0.000101	0.015802	2.122943
ART4	-1.07006	6.651188	-9.75833	0.000104	0.016031	2.097904
INA	-1.31837	4.002492	-9.73528	0.000105	0.016031	2.085591
TMEM100	-1.5642	4.342728	-9.69067	0.000108	0.016184	2.061666
C12orf45	-1.11599	4.975613	-9.65285	0.00011	0.016284	2.041277
DMBX1	1.286248	4.245658	9.506795	0.000119	0.016954	1.961638
HIST1H3H	-1.06479	7.440963	-9.48141	0.000121	0.016954	1.947648
DEPDC1	-1.14716	8.167862	-9.43458	0.000124	0.016954	1.921729
GFI1	1.45374	2.942064	9.404204	0.000126	0.016954	1.904834
UBE2S	-1.05886	5.961223	-9.3691	0.000129	0.016954	1.885228
NR6A1	-2.07761	2.147821	-9.15799	0.000145	0.017235	1.765512
CRELD2	1.032569	14.48918	9.131897	0.000147	0.017235	1.750492
ELMOD1	-1.11452	7.374577	-9.1223	0.000148	0.017235	1.744958

LIPJ	1.104336	4.590389	9.114006	0.000149	0.017235	1.740166
PRSS56	1.609275	6.652476	9.093573	0.000151	0.017235	1.728345
MEN1	1.430641	2.117261	9.086637	0.000151	0.017235	1.724325
CLEC9A	1.384302	5.95251	9.08217	0.000152	0.017235	1.721734
LSS	1.219223	6.785687	9.071544	0.000152	0.017235	1.715566
MUC13	1.259823	4.116511	8.93999	0.000165	0.017781	1.638508
LPL	1.077109	3.991337	8.845562	0.000174	0.018173	1.582401
SLC7A9	1.128771	3.979675	8.727394	0.000187	0.018442	1.511231
LRIT2	1.02883	4.935004	8.708621	0.000189	0.018442	1.499826
PCSK9	1.172407	6.906464	8.697167	0.00019	0.018442	1.492854
SMIM33	1.376811	6.416385	8.692639	0.000191	0.018442	1.490094
GOLGA6L22	1.086812	1.569317	8.681437	0.000192	0.018442	1.483261
HSP90B1	1.291304	13.18505	8.662209	0.000194	0.018442	1.47151
GEM	1.027936	6.784498	8.637358	0.000197	0.018442	1.456279
PTN	1.167287	5.534211	8.622881	0.000199	0.018442	1.447384
ANKRD33	1.503376	7.806753	8.595116	0.000202	0.018485	1.430278
NKX2-2	-1.76019	1.946905	-8.58206	0.000204	0.018549	1.422215
TDGF1	1.739899	6.502429	8.539118	0.000209	0.01871	1.395591
OPRPN	1.837417	1.997983	8.468972	0.000219	0.018946	1.351785
HIST1H2BA	-1.32335	5.186152	-8.45488	0.000221	0.018946	1.34294
OCM2	1.382195	3.985659	8.446535	0.000222	0.018946	1.33769
TUBA4A	-1.07667	13.46887	-8.4284	0.000224	0.018946	1.326264
WDR72	-1.56088	2.322154	-8.40098	0.000228	0.01901	1.308942
KIR2DS3	1.775916	2.165726	8.382809	0.000231	0.019049	1.297431
TNFSF14	1.652665	4.26384	8.353845	0.000235	0.019049	1.279023
HAND2	1.58167	5.81369	8.351542	0.000235	0.019049	1.277556
CBLN2	1.062352	4.918391	8.342995	0.000236	0.019049	1.272109
AC020909.1	1.073449	8.192611	8.313782	0.000241	0.019049	1.253446
KIF5A	-1.17482	5.118321	-8.30893	0.000241	0.019049	1.25034
ATP6V1G3	-2.37898	3.198717	-8.29239	0.000244	0.019049	1.239733
VIT	-1.26501	5.568967	-8.2836	0.000245	0.019049	1.234089
DGCR14	-1.06206	8.235687	-8.2794	0.000246	0.019049	1.23139
SCYGR4	1.267035	2.36677	8.249455	0.000251	0.019049	1.212099
SLIT1	1.354469	1.726839	8.2179	0.000256	0.019049	1.191689
ALDH3B1	1.011051	7.37196	8.198342	0.000259	0.019049	1.178997
TRAV8-3	-1.03846	4.555173	-8.1822	0.000262	0.019049	1.168498
ZFP57	1.48473	1.981156	8.149149	0.000267	0.019248	1.146929
PLEKHB1	1.32652	4.534623	8.115253	0.000273	0.019601	1.124711
IGFALS	1.02027	6.147737	8.024357	0.000289	0.019874	1.064643
OR7G1	1.357361	4.420782	7.954354	0.000303	0.019977	1.017889
SEMG2	2.128874	3.649279	7.947938	0.000304	0.019977	1.013582
C12orf56	-1.35901	3.021654	-7.92698	0.000308	0.020157	0.999491
RASSF10	1.146699	4.433793	7.905022	0.000313	0.02028	0.98468
TXNIP	-2.919	7.464884	-7.86601	0.000321	0.02028	0.958264
ARX	1.485272	4.568402	7.856466	0.000323	0.02028	0.95178
CCDC150	-1.00641	5.759936	-7.85097	0.000324	0.02028	0.948044
ST6GALNAC1	-1.68287	2.248102	-7.83732	0.000327	0.020381	0.938752
CT45A3	-1.16505	3.422446	-7.82934	0.000329	0.020381	0.933306

MANBAL	-2.05263	2.814282	-7.72306	0.000353	0.020863	0.860286
LSP1	1.15901	3.198201	7.702973	0.000358	0.020905	0.846371
C1orf185	1.38723	2.094855	7.693996	0.00036	0.02091	0.84014
ZNF273	-1.02897	5.846768	-7.6636	0.000367	0.021067	0.818983
OGA	-1.07021	9.965848	-7.59615	0.000384	0.021137	0.771724
OTOL1	1.811671	4.463898	7.590872	0.000386	0.021137	0.768009
TRAV13-2	-1.06302	6.640527	-7.56782	0.000392	0.021341	0.751752
TENT5A	-1.19039	4.564198	-7.56413	0.000393	0.021341	0.749141
SCN3A	2.100489	3.027808	7.538984	0.000399	0.02143	0.731337
FOXA1	-1.42382	6.868144	-7.52214	0.000404	0.02143	0.71938
C2orf57	1.873422	3.550495	7.498272	0.000411	0.021603	0.702383
DARS2	-1.6272	3.078743	-7.4867	0.000414	0.021672	0.694124
TMEFF2	-1.42821	1.992358	-7.46399	0.00042	0.021672	0.67788
NDUFC2	-1.18341	7.260462	-7.44561	0.000426	0.021729	0.664694
LAMB3	1.028755	12.0536	7.428531	0.000431	0.021823	0.652413
CBX2	1.250509	9.250549	7.353628	0.000454	0.022249	0.598217
SIM2	1.880944	1.965379	7.258708	0.000485	0.022846	0.52874
KCNV1	1.221532	4.477781	7.249632	0.000488	0.022907	0.52205
HIST1H4E	-1.06317	6.078679	-7.22606	0.000496	0.022934	0.504636
SESN3	-1.04869	5.119635	-7.19099	0.000509	0.022934	0.478623
KRT73	-1.20887	5.856575	-7.14448	0.000526	0.023117	0.443927
WNT7A	1.090081	9.735016	7.121693	0.000534	0.023117	0.426851
BIRC7	1.637962	8.829738	7.088805	0.000547	0.023183	0.402109
ENO4	1.11817	2.190424	7.05945	0.000559	0.023314	0.379929
LRCH1	-1.01937	6.947052	-7.0021	0.000583	0.023793	0.336339
NMBR	1.435755	5.490867	6.983282	0.000591	0.023793	0.32196
ELMO3	-1.15539	2.735527	-6.9615	0.0006	0.023793	0.305273
FAM46A	-1.18904	8.486865	-6.93755	0.000611	0.023977	0.286858
C4BPA	1.744836	1.935429	6.905861	0.000625	0.023977	0.26241
SPINK4	1.635465	3.668708	6.904025	0.000626	0.023977	0.260991
CEP57L1	-1.21219	3.512478	-6.8837	0.000635	0.024167	0.245249
NHLH2	1.802925	5.713205	6.88314	0.000635	0.024167	0.244812
LY6G6F-LY6G6D	2.070007	4.455097	6.878625	0.000638	0.024202	0.241309
GPR18	-2.22852	2.636136	-6.82233	0.000665	0.024635	0.197437
S100G	1.17906	1.709676	6.817464	0.000667	0.024643	0.193632
FYB	-1.33608	1.957872	-6.8086	0.000671	0.024643	0.186691
MT1G	1.253019	7.022501	6.796536	0.000677	0.024643	0.177225
MEOX2	-1.01112	9.590958	-6.78456	0.000683	0.024643	0.167813
SPANXN1	1.014608	4.364102	6.783614	0.000684	0.024643	0.167071
TAS2R46	-1.10412	4.158244	-6.69796	0.000729	0.025023	0.0993
OR4K1	1.315342	6.814435	6.628883	0.000768	0.025548	0.044047
SPANXC	-1.33331	4.723114	-6.5529	0.000814	0.026066	-0.01735
KRTAP4-5	1.538199	5.801783	6.52966	0.000829	0.026111	-0.03626
MYCBPAP	-1.59501	2.120034	-6.52775	0.00083	0.026111	-0.03781
SQSTM1	1.0923	11.39134	6.483675	0.000858	0.026459	-0.07386
CDH22	1.255744	3.587426	6.462158	0.000873	0.026572	-0.09153
TRBV3-1	1.461649	4.147196	6.445746	0.000884	0.026759	-0.10505

KRTAP9-4	1.055655	4.728573	6.391822	0.000922	0.027183	-0.14969
GDF11	-1.18429	4.85805	-6.38296	0.000928	0.027292	-0.15705
IFIT1	-1.54573	9.746189	-6.3505	0.000952	0.027342	-0.18413
TM4SF19	1.885402	5.723353	6.33432	0.000964	0.027342	-0.19767
TERB1	1.034021	4.555235	6.330297	0.000967	0.027342	-0.20104
DNAI1	1.086816	5.092061	6.314994	0.000979	0.027507	-0.21388
C19orf67	-1.11512	2.637656	-6.31228	0.000981	0.027507	-0.21616
NKX6-1	2.000653	2.1005	6.217581	0.001058	0.028502	-0.29628
L3MBTL4	1.594482	4.643548	6.189687	0.001082	0.028641	-0.32009
HIST1H4G	-1.35663	2.889121	-6.1375	0.001128	0.029191	-0.36488
GHRL	-1.01265	4.853884	-6.10182	0.001161	0.029574	-0.3957
BICDL1	1.361988	5.039916	6.099807	0.001163	0.029574	-0.39744
ITGAX	1.278735	3.695722	6.079776	0.001182	0.029766	-0.41482
LRRC69	-1.2877	3.25919	-6.07272	0.001189	0.029766	-0.42095
MOBP	1.251601	3.779729	6.05308	0.001208	0.02992	-0.43805
GPNMB	1.600258	2.310456	6.033257	0.001227	0.03028	-0.45536
AC026316.4	-1.53629	2.378888	-6.01014	0.001251	0.030464	-0.47561
NIN	-1.27878	4.995236	-6.00258	0.001259	0.030515	-0.48224
ELP4	-1.29418	3.346572	-5.97677	0.001286	0.030762	-0.50495
BHLHE23	1.014831	11.31349	5.94916	0.001315	0.031076	-0.52934
KRT25	1.148917	3.593425	5.944222	0.001321	0.0311	-0.53372
KLRG1	-1.05198	7.408066	-5.93121	0.001335	0.031216	-0.54525
PMIS2	1.123274	3.765795	5.928498	0.001338	0.031219	-0.54766
IGHV3-11	1.642468	3.912228	5.898621	0.001371	0.031531	-0.57424
SH3BP4	-1.13672	4.854946	-5.89482	0.001376	0.031534	-0.57763
HIST1H2AA	-1.86985	2.572546	-5.88738	0.001384	0.031598	-0.58427
CST4	-1.69035	2.829214	-5.82809	0.001455	0.032084	-0.63743
ORM2	1.279844	6.090749	5.8181	0.001467	0.032152	-0.64644
MFAP5	1.099764	2.858997	5.811302	0.001475	0.032187	-0.65257
IFI44L	-2.08582	5.520065	-5.80072	0.001488	0.032187	-0.66214
ZNF630	1.396398	3.517713	5.795435	0.001495	0.032187	-0.66691
GYPE	1.309488	4.348424	5.789258	0.001503	0.032187	-0.67251
NAP1L1	-1.70331	3.695512	-5.75032	0.001553	0.032827	-0.70786
PSG7	1.081409	5.427127	5.733519	0.001575	0.032944	-0.72319
FOXI2	2.377433	3.103749	5.731143	0.001579	0.032961	-0.72536
MEIS2	-1.02807	5.107981	-5.7265	0.001585	0.033057	-0.7296
PRAMEF4	1.023255	1.619963	5.707109	0.001611	0.033306	-0.74734
AL162596.1	1.177472	4.94177	5.66374	0.001672	0.033908	-0.78721
SLC22A1	1.012112	5.633874	5.65455	0.001685	0.034044	-0.79569
TAS2R19	-1.21359	4.140012	-5.64986	0.001692	0.034044	-0.80003
TRGV10	1.039721	5.492431	5.644933	0.001699	0.034073	-0.80458
OR56A1	1.174046	6.600298	5.584893	0.00179	0.034523	-0.86034
DUSP26	-1.33439	2.968494	-5.58067	0.001796	0.034573	-0.86429
IQCF3	1.178945	3.058974	5.570466	0.001812	0.03462	-0.87381
T	2.945318	3.108653	5.561436	0.001826	0.034666	-0.88226
THEMIS	1.107741	4.701303	5.549076	0.001846	0.034814	-0.89384
PPP1R2C	-1.11877	3.043777	-5.53777	0.001864	0.03482	-0.90445
DMRTB1	1.121566	1.587498	5.537345	0.001865	0.03482	-0.90485

MTMR8	-1.49569	2.370964	-5.53711	0.001865	0.03482	-0.90507
SMG9	-1.09506	3.726993	-5.52437	0.001886	0.03482	-0.91704
CSF2	1.498596	8.829127	5.491654	0.001941	0.034838	-0.9479
BLOC1S1	-1.51429	3.321288	-5.4536	0.002007	0.035333	-0.98398
GSTA2	1.009245	6.624647	5.449749	0.002014	0.035339	-0.98764
OR7D4	1.834356	4.511382	5.445413	0.002021	0.035414	-0.99177
TAS1R1	1.79898	2.214584	5.419178	0.002069	0.03589	-1.01679
PRSS35	1.453011	1.809466	5.4186	0.00207	0.03589	-1.01734
OR12D2	1.172407	4.120455	5.404658	0.002096	0.036136	-1.03067
OR52K1	-1.3021	2.038513	-5.3853	0.002132	0.036309	-1.04923
SULT1C2	1.103419	4.307088	5.362815	0.002175	0.036502	-1.07086
KRT1	2.099578	2.921733	5.354059	0.002192	0.036502	-1.0793
CLEC3B	-1.08656	4.740407	-5.34766	0.002205	0.036529	-1.08547
ECE2	1.273181	2.962132	5.296776	0.002308	0.037165	-1.13478
PCDHA4	1.309211	2.047372	5.291779	0.002318	0.037173	-1.13964
SH2D1B	-1.67009	2.262162	-5.29177	0.002318	0.037173	-1.13965
CCL3L1	1.345911	5.717874	5.286326	0.002329	0.037219	-1.14495
OPN4	-1.50452	2.156003	-5.26806	0.002368	0.037447	-1.16276
ATP1A3	1.383162	1.979597	5.24011	0.002429	0.037988	-1.19012
CCDC129	2.778	3.152207	5.239514	0.00243	0.037988	-1.1907
PDZD3	1.249157	6.499116	5.212805	0.00249	0.038663	-1.21695
OSCAR	1.597912	6.674299	5.20227	0.002514	0.03891	-1.22733
SPN	1.307301	3.25155	5.190966	0.00254	0.039117	-1.23848
ADGRF4	1.903226	2.602618	5.190661	0.002541	0.039117	-1.23878
SPI1	1.215657	3.030099	5.184636	0.002555	0.039244	-1.24474
TRAV17	1.292093	4.593794	5.141771	0.002657	0.039655	-1.28724
TAS2R20	-1.05052	5.957419	-5.13663	0.00267	0.039768	-1.29236
GJD4	1.522601	2.201201	5.117676	0.002717	0.040112	-1.31125
CCDC152	-1.16035	7.514923	-5.11427	0.002725	0.040174	-1.31465
CST2	-1.04819	2.786853	-5.06535	0.002851	0.041087	-1.36368
UGT2A1	-1.14944	4.549185	-5.05955	0.002867	0.041107	-1.36952
DYNAP	1.761435	2.292691	5.048344	0.002897	0.041249	-1.38081
SLA	1.570078	2.346717	5.036118	0.00293	0.041418	-1.39315
KCNH4	1.114055	4.759995	5.015795	0.002986	0.041879	-1.41371
PDYN	1.070889	3.085796	5.007429	0.00301	0.04215	-1.42219
PDLIM2	1.172923	1.677887	4.995593	0.003043	0.042385	-1.4342
AL353579.1	1.064873	10.77719	4.99243	0.003052	0.042428	-1.43742
PALM2	-1.20352	3.213252	-4.97261	0.00311	0.042783	-1.45759
IL18BP	-1.32805	4.025262	-4.96121	0.003143	0.04299	-1.46923
PASD1	1.033328	2.068357	4.959425	0.003148	0.043034	-1.47105
APOL5	1.416585	4.067745	4.940966	0.003204	0.04333	-1.48993
OR3A3	1.604058	2.051942	4.940481	0.003205	0.04333	-1.49043
BICDL2	1.466824	3.551274	4.903902	0.003318	0.044059	-1.52799
CLEC1B	-2.12018	4.702108	-4.88824	0.003368	0.044192	-1.54414
CAPN12	1.323749	3.993898	4.875537	0.003409	0.044456	-1.55726
CTSW	1.195109	1.669151	4.875072	0.00341	0.044456	-1.55774
ZNF727	1.561254	3.961931	4.866959	0.003437	0.044623	-1.56613
C12orf57	-1.33703	3.244724	-4.84099	0.003523	0.044968	-1.59306

IL15	-1.12471	4.573409	-4.84007	0.003526	0.044968	-1.59403
OR2V1	-1.65565	2.229801	-4.83619	0.00354	0.044968	-1.59806
KRT35	-1.30397	3.964669	-4.82271	0.003586	0.045178	-1.61209
CHFR	1.288687	6.909135	4.809409	0.003632	0.045486	-1.62596
SLC28A2	1.021856	3.216702	4.807673	0.003638	0.045515	-1.62777
SSC4D	-1.50909	2.439416	-4.80225	0.003657	0.045515	-1.63344
TMEM71	-1.23623	5.511292	-4.77688	0.003747	0.045961	-1.65999
SLC8A2	1.35771	5.821128	4.7717	0.003766	0.045964	-1.66543
C7orf62	1.039379	4.877972	4.750899	0.003843	0.046481	-1.68729
NAA11	-1.86751	3.132556	-4.73871	0.003889	0.046744	-1.70013
RSAD2	-2.84303	3.68374	-4.7355	0.003901	0.046744	-1.70352
SIGLEC11	-1.35589	2.275043	-4.7024	0.004029	0.047376	-1.73853
LDHAL6A	1.410367	1.807227	4.691075	0.004073	0.04763	-1.75054
ALG12	1.002272	4.732607	4.680458	0.004116	0.047828	-1.76183
GDF5	1.055452	5.200916	4.665291	0.004178	0.048133	-1.77797
AL357673.1	1.378391	1.737078	4.65677	0.004213	0.04832	-1.78706
BMP5	1.054991	3.782394	4.649699	0.004242	0.048425	-1.79461
TENT5C	-1.38118	2.408436	-4.64787	0.00425	0.048453	-1.79656
AC104304.1	1.492651	5.329712	4.646924	0.004254	0.048464	-1.79757
OR52D1	1.15329	2.091116	4.644016	0.004266	0.048549	-1.80068
JDP2	1.986835	10.69223	4.638037	0.004291	0.048736	-1.80708
ASNS	2.021931	10.57966	4.629806	0.004326	0.048826	-1.81589
KIAA1524	-2.03935	2.676343	-4.61096	0.004407	0.049094	-1.8361
DEFB104B	1.840175	2.80552	4.598867	0.004461	0.049423	-1.84911
TMEM178B	1.30916	8.908797	4.598175	0.004464	0.049423	-1.84985
NXPH1	1.544164	1.79276	4.595999	0.004473	0.049457	-1.85219
TRAV36DV7	1.162334	1.672603	4.5919	0.004491	0.049571	-1.85661
SLC16A6	1.073295	5.712248	4.585165	0.004522	0.049671	-1.86386
ERICH4	-1.51508	2.114517	-4.57858	0.004551	0.049748	-1.87097
AR	1.110969	5.007566	4.576321	0.004561	0.049806	-1.87341
CLDN25	1.728868	6.846051	4.550669	0.004679	0.050304	-1.90115
ZNF729	-1.25603	1.900985	-4.54604	0.004701	0.050387	-1.90616
LOC339862	1.126562	4.898708	4.540535	0.004727	0.050538	-1.91214
CD2	1.011537	4.28113	4.538195	0.004738	0.050601	-1.91468
GALNT14	-1.32929	3.56265	-4.51067	0.00487	0.051213	-1.9446
LCE5A	1.198758	6.824411	4.50918	0.004878	0.05124	-1.94623
MAGEA3	-1.07701	1.659323	-4.50572	0.004895	0.051336	-1.95001
NGF	1.001933	8.886029	4.492802	0.004959	0.051742	-1.96411
HTR2B	1.077092	8.587796	4.480628	0.00502	0.051943	-1.97742
TMEM200B	-1.38313	1.895991	-4.46855	0.005081	0.052108	-1.99065
TEX15	-2.13403	3.220534	-4.46039	0.005123	0.052285	-1.9996
KLK8	1.1549	4.39613	4.454069	0.005156	0.052439	-2.00654
GABPA	-1.20057	3.577438	-4.44953	0.00518	0.052521	-2.01153
FERMT1	-1.43455	3.405945	-4.44362	0.005211	0.052521	-2.01802
CHAC1	3.007312	9.565202	4.442173	0.005219	0.052526	-2.01962
IFITM1	-1.34578	10.72853	-4.43651	0.005249	0.052671	-2.02586
GPN3	1.085981	7.204602	4.430755	0.005279	0.052748	-2.03219
TNNI1	-1.17661	1.786096	-4.42699	0.0053	0.052847	-2.03634

OR10G4	1.165177	2.265971	4.426394	0.005303	0.052853	-2.037
SMIM9	1.028121	4.900385	4.422373	0.005325	0.05294	-2.04143
AC132217.2	-1.2526	3.216005	-4.41796	0.005348	0.053116	-2.04631
OR10T2	1.091336	4.069647	4.389852	0.005504	0.053953	-2.0774
PLD4	1.552337	2.053731	4.384615	0.005533	0.054058	-2.08321
SP140	-1.15795	3.223376	-4.37994	0.00556	0.054154	-2.0884
PRKACG	1.114016	2.511936	4.37982	0.00556	0.054154	-2.08853
MEIS1	-1.1076	3.958621	-4.37644	0.00558	0.054264	-2.09229
IFI44	-1.06995	6.708832	-4.37392	0.005594	0.054334	-2.09509
DDX53	1.238203	6.647009	4.372464	0.005602	0.054382	-2.0967
IL1RAPL2	1.062987	4.253929	4.361309	0.005667	0.054669	-2.10911
FAM227A	-1.01606	4.36333	-4.35464	0.005706	0.054789	-2.11654
KCNJ13	1.21148	2.031087	4.344671	0.005764	0.055006	-2.12765
C1orf228	1.064183	7.040035	4.341171	0.005785	0.055015	-2.13156
LRRC63	-1.05551	2.636643	-4.34008	0.005791	0.05504	-2.13277
EML5	-1.30431	3.292851	-4.3385	0.005801	0.055055	-2.13453
KRT6A	1.344684	4.295834	4.325261	0.00588	0.055497	-2.14934
DHRS4	-1.07956	3.488886	-4.32488	0.005883	0.055497	-2.14976
OGN	-1.71579	2.429745	-4.32187	0.005901	0.055533	-2.15314
AC007846.2	-1.09542	1.899158	-4.31553	0.00594	0.055555	-2.16023
VMAC	-1.55956	2.412749	-4.31134	0.005965	0.055555	-2.16493
REELD1	-1.28967	2.408129	-4.31015	0.005973	0.055555	-2.16625
PCK2	1.411666	11.32776	4.307878	0.005987	0.055583	-2.16881
MAP3K9	-1.21041	2.246351	-4.30785	0.005987	0.055583	-2.16884
SPATA22	1.223016	2.015328	4.301485	0.006026	0.055823	-2.17598
MRPL23	-1.45379	2.706519	-4.29005	0.006098	0.056168	-2.18883
C4orf50	1.11906	1.681744	4.289848	0.006099	0.056168	-2.18905
C3orf84	-1.25212	4.052729	-4.28811	0.00611	0.056244	-2.19101
CPB2	1.028079	3.865683	4.265169	0.006257	0.056682	-2.21685
TNFSF15	1.049871	13.6656	4.252477	0.006341	0.057009	-2.23118
AL603764.2	1.238802	5.930956	4.246834	0.006378	0.057239	-2.23756
MYO7A	1.445241	2.174005	4.24507	0.00639	0.057319	-2.23956
WFIKKN1	-1.30625	2.075168	-4.23831	0.006435	0.057522	-2.24721
SLC24A4	1.557489	2.149724	4.236375	0.006448	0.057592	-2.2494
ASB11	1.733155	1.973709	4.209227	0.006633	0.058429	-2.28021
FADS3	-1.04613	2.152918	-4.19906	0.006704	0.058672	-2.29178
HKDC1	1.884791	3.934131	4.191257	0.006759	0.05888	-2.30067
AADACL2	1.247778	1.814099	4.186654	0.006792	0.05899	-2.30592
IGHV3-30	1.035063	4.530737	4.166029	0.006941	0.059826	-2.32948
OR10G2	1.461243	2.861541	4.147911	0.007075	0.060528	-2.35023
MFSD4A	-1.21414	3.853223	-4.14606	0.007089	0.060528	-2.35236
HAP1	1.063348	2.133939	4.135388	0.007169	0.060718	-2.36461
OR2G2	1.16933	2.066245	4.132091	0.007194	0.060727	-2.36839
S100A8	1.285285	1.729864	4.129779	0.007212	0.060727	-2.37105
IL23R	1.016762	2.559859	4.117674	0.007305	0.060962	-2.38498
SPSB4	-1.0995	2.599549	-4.10745	0.007385	0.061182	-2.39676
FAM26D	-2.39707	3.239104	-4.09615	0.007474	0.061515	-2.4098
MS4A15	-1.04652	1.874486	-4.08755	0.007542	0.061758	-2.41975

DCLK1	-1.24811	3.979341	-4.08204	0.007587	0.061827	-2.42612
PCID2	-1.01942	5.054172	-4.08054	0.007599	0.061827	-2.42785
KAL1	-1.80081	2.9306	-4.04606	0.007884	0.062996	-2.46786
NEUROD1	1.229412	1.994551	4.046017	0.007884	0.062996	-2.46791
TRIML1	1.773515	1.934392	4.041622	0.007922	0.063182	-2.47302
DLG3	1.677002	6.504931	4.041052	0.007927	0.063182	-2.47368
FBP1	-1.30938	2.731241	-4.03631	0.007967	0.06331	-2.47921
LGR5	1.020073	1.592389	4.031732	0.008006	0.063423	-2.48454
GPR174	1.765711	1.978102	4.030214	0.008019	0.063442	-2.48631
TUBAL3	-1.26019	3.565789	-4.02752	0.008042	0.063514	-2.48945
PCDHGB2	-1.16874	2.749148	-4.02075	0.008101	0.063585	-2.49735
PTGDR2	-1.93113	2.500996	-4.01972	0.00811	0.063631	-2.49854
BOLA2-SMG1P6	-1.14081	3.815185	-4.01227	0.008175	0.063902	-2.50725
VEGFA	1.21035	9.293075	4.009618	0.008199	0.064007	-2.51034
SARM1	-1.2753	2.121936	-3.97312	0.008528	0.065357	-2.55311
PIWIL2	-1.14833	1.755163	-3.9703	0.008554	0.065436	-2.55643
EPSTI1	-1.06008	9.356257	-3.9649	0.008604	0.065561	-2.56277
CAMKV	1.029492	2.007539	3.961349	0.008638	0.065703	-2.56694
OSM	1.123495	5.505054	3.952247	0.008723	0.066158	-2.57766
CSF3	1.09678	8.391616	3.943744	0.008804	0.066501	-2.58768
OR10C1	1.130718	1.896639	3.940389	0.008836	0.066645	-2.59164
OR4A47	1.048713	6.716897	3.930958	0.008927	0.067061	-2.60277
NOXRED1	1.404016	3.361708	3.908193	0.009152	0.068041	-2.6297
KLHL38	1.52296	2.2244	3.907939	0.009154	0.068041	-2.63
GPR27	1.04451	4.39335	3.904415	0.00919	0.068142	-2.63418
ZIC3	1.260558	1.648955	3.900739	0.009227	0.068305	-2.63854
NOTCH3	1.3058	7.35031	3.895325	0.009281	0.068512	-2.64496
OR8U8	1.922395	2.656925	3.888354	0.009352	0.06876	-2.65324
CHRNA10	-1.12509	2.862402	-3.88433	0.009394	0.068796	-2.65802
LYPD5	-1.09062	2.599797	-3.8804	0.009434	0.069029	-2.66269
OR2V2	1.584705	2.799449	3.878644	0.009453	0.069102	-2.66478
SPACA3	1.705612	2.783292	3.878056	0.009459	0.069105	-2.66548
GRID1	1.134808	2.79728	3.871828	0.009523	0.069152	-2.67289
DEFB125	1.49569	2.187713	3.8688	0.009555	0.069259	-2.6765
EIF4E1B	1.770369	3.369003	3.86686	0.009576	0.069333	-2.67881
FOXG1	-1.22837	1.976939	-3.86402	0.009605	0.069451	-2.68219
LOC100505549	-1.00213	4.506151	-3.86284	0.009618	0.069468	-2.6836
LOR	1.522346	2.644494	3.861037	0.009637	0.069487	-2.68575
FOXD2	1.179932	1.873185	3.844507	0.009814	0.070091	-2.70548
C8orf58	-1.06431	3.8557	-3.83612	0.009905	0.070447	-2.7155
FAM204A	-1.5961	3.702184	-3.83068	0.009965	0.070749	-2.72202
PDZD4	1.092481	5.900281	3.819474	0.010089	0.071333	-2.73544
KLF4	1.837264	9.230617	3.818704	0.010097	0.071344	-2.73636
GPR87	-1.41798	2.702043	-3.81649	0.010122	0.071403	-2.73901
AC008537.1	1.017111	4.284511	3.816452	0.010123	0.071403	-2.73906
ADAMTS16	1.050668	2.765327	3.801215	0.010295	0.071772	-2.75735
ADAMTS15	-1.21723	5.203922	-3.78864	0.010439	0.072386	-2.77248

HDAC8	-1.07453	4.991931	-3.77722	0.010573	0.072832	-2.78624
CCDC27	-1.04823	5.322903	-3.75946	0.010784	0.073382	-2.80766
LIF	1.652718	2.67146	3.751601	0.010879	0.07371	-2.81717
NECAB2	1.076207	2.003221	3.747802	0.010925	0.073814	-2.82176
SGCZ	1.465251	2.488916	3.747672	0.010927	0.073814	-2.82192
FAM47A	1.854681	2.917287	3.724288	0.011216	0.074929	-2.85026
KRTAP10-11	1.592785	2.144087	3.71741	0.011303	0.075263	-2.85861
KRTAP12-2	1.016837	4.944711	3.707018	0.011436	0.075659	-2.87124
PDZK1	-1.12257	5.017244	-3.69569	0.011582	0.076201	-2.88503
OR2T27	1.520812	3.933686	3.695113	0.01159	0.076204	-2.88574
IDO1	-1.24348	5.159984	-3.69368	0.011608	0.076265	-2.88748
OR2T10	-1.36243	2.80547	-3.69329	0.011613	0.076265	-2.88796
KRT77	1.071645	2.836601	3.685588	0.011715	0.076631	-2.89735
BTLA	1.082697	2.333515	3.681262	0.011772	0.076714	-2.90262
MAPT	1.072069	8.334767	3.668953	0.011936	0.077463	-2.91766
DPYS	1.258606	2.074621	3.665752	0.01198	0.077556	-2.92157
PNMT	-1.24049	3.563711	-3.66012	0.012056	0.077723	-2.92846
RNF17	1.480598	2.472417	3.635374	0.012399	0.078887	-2.95879
TMPRSS11E	1.241222	1.689133	3.63201	0.012446	0.079056	-2.96292
TAS2R13	-1.27822	2.883327	-3.62525	0.012542	0.079435	-2.97122
ERBB4	-1.68375	2.360938	-3.62269	0.012578	0.079561	-2.97437
AMER3	1.816414	2.343045	3.612198	0.012729	0.079972	-2.98728
PCDHA1	1.539438	4.764627	3.601947	0.012879	0.080498	-2.99991
FXN	-1.29174	3.762975	-3.59113	0.013039	0.081007	-3.01325
C17orf104	-1.2331	2.085292	-3.59026	0.013052	0.081008	-3.01432
TRPC7	1.017056	5.365493	3.58757	0.013092	0.081035	-3.01764
CLEC2D	1.041227	3.938239	3.585971	0.013116	0.081128	-3.01962
CEBPB	1.27223	11.30855	3.573362	0.013306	0.08159	-3.0352
LOC93432	1.55091	2.304067	3.569757	0.013361	0.081681	-3.03966
PSAT1	1.098238	10.09327	3.567687	0.013393	0.081681	-3.04223
FER1L6	-1.61913	2.717035	-3.56579	0.013422	0.081763	-3.04457
C8orf89	1.030867	1.915836	3.563507	0.013457	0.081829	-3.0474
KIR2DS4	1.810543	6.096693	3.540132	0.013823	0.082993	-3.07639
SRRM3	1.233613	5.55322	3.539832	0.013827	0.082997	-3.07676
ZNF316	-1.33644	3.260236	-3.5259	0.014051	0.083824	-3.09409
PLP1	1.777765	2.385773	3.525677	0.014054	0.083824	-3.09436
CTAG2	-1.39469	2.849537	-3.52214	0.014112	0.08391	-3.09876
CKMT2	-1.02328	2.268749	-3.51739	0.014189	0.084128	-3.10468
TEX26	2.235112	3.070655	3.488523	0.01467	0.085741	-3.14069
OR5BS1P	1.333386	2.437086	3.472364	0.014947	0.086619	-3.16091
OTULINL	-1.62154	3.138712	-3.4653	0.01507	0.087159	-3.16975
IFNA10	2.035289	3.883616	3.455159	0.015249	0.087856	-3.18247
SYCP1	1.123035	3.932244	3.444865	0.015433	0.088553	-3.1954
TSGA10IP	1.05101	3.958579	3.443328	0.01546	0.088612	-3.19733
C1QTNF9	-1.10111	2.180431	-3.43662	0.015581	0.089057	-3.20575
SMIM10L2A	1.020838	3.300293	3.434968	0.015611	0.08913	-3.20784
CCDC158	1.394875	2.111972	3.430843	0.015687	0.08931	-3.21303
AC004805.1	-1.46267	3.487064	-3.4149	0.015981	0.090061	-3.23311

AL031847.2	-1.216	2.901352	-3.404	0.016186	0.090916	-3.24686
CENPE	-1.18884	10.56199	-3.39901	0.016281	0.091198	-3.25316
DPEP2	1.23538	1.851497	3.397064	0.016318	0.091331	-3.25561
SFRP4	1.151987	2.463023	3.384583	0.016559	0.092048	-3.27139
ACSM4	-1.05317	4.184155	-3.3729	0.016788	0.092803	-3.28618
PLEKHA6	-1.08855	5.61115	-3.36358	0.016973	0.093434	-3.29799
CLEC20A	-1.07291	4.008948	-3.33413	0.017572	0.095224	-3.33541
CAPS	-1.53118	2.87339	-3.33228	0.017611	0.095381	-3.33776
RYBP	-1.25611	2.252655	-3.32953	0.017668	0.095539	-3.34126
DLGAP1	-1.35971	2.486183	-3.32484	0.017766	0.095817	-3.34722
AC018709.1	1.08837	3.536617	3.32324	0.0178	0.095883	-3.34927
INSYN2A	-1.36987	2.977607	-3.31988	0.017871	0.096065	-3.35355
DPPA5	1.528327	2.996982	3.318397	0.017902	0.096095	-3.35544
BEND5	1.113891	3.825264	3.31542	0.017965	0.096283	-3.35923
MAFA	-1.4758	2.589613	-3.30929	0.018096	0.096681	-3.36706
CR1	1.500928	2.290679	3.30365	0.018218	0.097075	-3.37425
OR8A1	1.122774	5.69219	3.301203	0.018271	0.097206	-3.37737
ETNPPL	1.572378	2.396695	3.288504	0.018548	0.097969	-3.3936
CDY2A	1.108003	1.966367	3.287211	0.018577	0.098095	-3.39526
COL4A6	-1.16736	4.031724	-3.26214	0.019139	0.09983	-3.42737
TMEM210	1.29778	4.801904	3.261036	0.019165	0.099885	-3.42878
SLC6A9	1.752466	4.262624	3.256873	0.01926	0.100229	-3.43412
ZBTB8B	-1.19013	4.072622	-3.25231	0.019365	0.100648	-3.43997
PRR21	1.222322	7.02583	3.251085	0.019394	0.100698	-3.44155
WFDC5	2.729352	5.739442	3.239086	0.019674	0.101409	-3.45697
COL9A1	1.022718	2.657825	3.234867	0.019773	0.101606	-3.46239
IGHV7-4-1	1.418467	3.809354	3.228292	0.019929	0.101868	-3.47085
F2RL3	1.139475	7.764788	3.222717	0.020063	0.102274	-3.47803
CXCR1	1.006038	4.332915	3.200583	0.020603	0.103735	-3.50657
ABRA	-1.60928	2.480838	-3.19764	0.020676	0.10383	-3.51036
RRH	1.026897	3.748313	3.193389	0.020782	0.104061	-3.51586
TEX49	1.314096	1.752268	3.191919	0.020819	0.104181	-3.51776
ULBP1	1.233515	9.774881	3.181845	0.021073	0.104888	-3.53078
GRIP1	1.131683	4.900805	3.181376	0.021085	0.104896	-3.53139
GCNT7	1.280975	2.384209	3.179938	0.021121	0.105009	-3.53325
OR5AU1	1.365324	2.260462	3.178898	0.021148	0.105082	-3.53459
DDIT3	3.193181	11.29101	3.173699	0.021281	0.105538	-3.54132
LELP1	2.192317	3.00008	3.166697	0.021461	0.105843	-3.55039
REN	1.44162	2.778871	3.165227	0.0215	0.105954	-3.55229
TRAV13-1	-1.10058	2.604217	-3.16353	0.021544	0.10607	-3.5545
TCF23	1.284056	2.052952	3.163133	0.021554	0.106088	-3.55501
SIAH3	1.610559	3.595286	3.158096	0.021685	0.106384	-3.56153
ETDB	-1.35744	2.696003	-3.15379	0.021799	0.10655	-3.56712
SOGA3	1.784558	6.658376	3.143431	0.022074	0.107412	-3.58056
PCDHA13	2.01513	2.843884	3.142072	0.02211	0.107487	-3.58233
BMP4	-1.01876	13.08601	-3.13453	0.022313	0.108095	-3.59212
PCSK1N	1.196507	11.31991	3.129034	0.022462	0.108631	-3.59927
GREM2	-1.31078	4.011749	-3.12512	0.022569	0.108813	-3.60435

OR5B21	1.840976	3.26798	3.11819	0.02276	0.109188	-3.61338
GHSR	1.001863	3.767697	3.111975	0.022932	0.109685	-3.62147
S100A14	-1.26228	1.940794	-3.10261	0.023195	0.110381	-3.63367
IL17REL	1.003493	3.34138	3.102506	0.023198	0.110381	-3.63381
OR2L3	-1.29535	3.180098	-3.0848	0.023703	0.111729	-3.6569
CMKLR1	1.029402	2.264133	3.081929	0.023787	0.111952	-3.66066
OR10AD1	1.342467	3.563838	3.081476	0.0238	0.111952	-3.66125
TRIB3	1.368945	9.769752	3.074561	0.024001	0.112636	-3.67029
PRG2	1.203461	2.755636	3.070274	0.024127	0.113041	-3.67589
CXCL2	1.092671	11.74006	3.070162	0.024131	0.113041	-3.67604
MEP1A	-1.00586	2.679346	-3.06691	0.024227	0.113337	-3.6803
PRR18	1.215702	3.636471	3.066537	0.024238	0.113361	-3.68078
KLC1	-1.46248	2.315399	-3.06335	0.024333	0.113518	-3.68495
SLC22A8	1.028019	4.040758	3.052472	0.024659	0.114343	-3.69919
C12orf71	-1.44075	3.010993	-3.04555	0.024869	0.114808	-3.70827
HIST1H2BF	-1.82179	3.494667	-3.04542	0.024873	0.114808	-3.70844
CASP14	1.906799	2.613367	3.036451	0.025148	0.115504	-3.7202
BSX	1.534156	2.516186	3.027012	0.025441	0.116405	-3.73259
PPP1R42	-1.33356	2.781948	-3.02153	0.025613	0.116905	-3.73979
PROZ	-1.50162	1.915135	-3.01586	0.025792	0.117303	-3.74723
STX1B	1.050196	3.428598	3.012893	0.025886	0.117626	-3.75114
SLC16A9	-1.17843	2.982664	-3.00829	0.026033	0.117956	-3.75718
ACKR1	-1.74985	2.388187	-3.00277	0.026211	0.118524	-3.76445
MTUS2	1.40203	1.808924	2.996712	0.026407	0.118965	-3.77243
TMEM101	-1.00853	2.230724	-2.99328	0.026519	0.1192	-3.77694
C2CD4B	1.256605	12.00444	2.990574	0.026608	0.119501	-3.78051
C1orf159	-1.23767	3.414547	-2.98206	0.026889	0.12021	-3.79174
MC3R	1.040052	4.262185	2.976057	0.027089	0.120708	-3.79965
GAGE7	1.049233	2.816404	2.975849	0.027096	0.120713	-3.79992
CNDP1	1.184435	3.981526	2.960312	0.027622	0.12194	-3.82043
CSN3	-1.27164	2.077577	-2.959	0.027666	0.121999	-3.82216
EID1	-1.04992	4.649527	-2.95412	0.027834	0.122377	-3.82861
RASL11A	-1.26979	3.541352	-2.93759	0.028411	0.123717	-3.85048
DDIT4	1.551043	14.01642	2.935021	0.028502	0.12394	-3.85387
LURAP1L	1.660123	7.79418	2.915404	0.029205	0.125996	-3.87986
MOGAT3	1.0944	3.008656	2.912184	0.029323	0.126231	-3.88413
ZNF835	1.084142	2.917872	2.90044	0.029755	0.12714	-3.89971
TRAV34	1.133787	1.92488	2.89926	0.029799	0.127188	-3.90128
DEFB130B	-1.26113	1.816569	-2.89908	0.029805	0.12719	-3.90152
ADM2	1.034968	6.740174	2.894085	0.029992	0.127692	-3.90815
RASD1	1.322462	11.25629	2.893302	0.030021	0.127789	-3.90919
TLR10	1.458311	2.186718	2.890353	0.030132	0.127995	-3.91311
CFC1	1.683399	3.240026	2.88878	0.030191	0.12814	-3.9152
ASB18	-1.73083	3.720949	-2.87579	0.030685	0.129619	-3.93247
NPY5R	-1.10618	2.813929	-2.84435	0.031917	0.13236	-3.97434
TRMT1	-1.12275	2.6237	-2.84227	0.032001	0.132578	-3.97711
SLC10A1	1.373725	2.713413	2.829158	0.032532	0.133993	-3.9946
UBQLNL	-1.1553	2.516406	-2.82243	0.032808	0.134481	-4.00358

SPATA31A3	-1.08281	1.845884	-2.82117	0.032861	0.134612	-4.00527
PFKFB1	1.32621	3.222071	2.812238	0.033232	0.135575	-4.01721
FIGLA	1.34005	2.523285	2.809502	0.033347	0.135662	-4.02086
CACNA1D	1.140152	1.663194	2.809303	0.033355	0.135662	-4.02113
SERTAD1	-1.04726	2.098444	-2.80596	0.033496	0.135911	-4.0256
ZBTB7C	1.046889	4.948505	2.800866	0.033712	0.136268	-4.03241
FAM129A	1.016959	6.800862	2.799975	0.03375	0.136346	-4.0336
HSPA5	1.049297	12.92471	2.799108	0.033787	0.136414	-4.03476
LACTBL1	-1.02589	1.629845	-2.77676	0.034754	0.13884	-4.06468
ALDH1L2	1.277943	7.161056	2.776294	0.034774	0.13884	-4.0653
CORIN	-1.00761	4.662869	-2.76128	0.035441	0.140326	-4.08543
IP6K3	1.302669	2.845308	2.746714	0.036102	0.141575	-4.10498
PENK	-1.17772	3.171444	-2.74657	0.036108	0.141575	-4.10517
KIF17	1.148441	3.613225	2.738081	0.0365	0.142446	-4.11657
POM121L2	1.037401	3.760402	2.737956	0.036505	0.142446	-4.11674
IGFL3	-1.47903	1.860009	-2.73738	0.036532	0.142494	-4.11751
DNAJB9	1.847055	10.82932	2.737352	0.036533	0.142494	-4.11755
CDCP2	1.069581	2.239944	2.733879	0.036695	0.142774	-4.12222
OVOL2	1.006145	3.385922	2.731315	0.036815	0.143104	-4.12566
RNF212B	1.085718	1.96818	2.721638	0.03727	0.143865	-4.13867
RD3	-1.52266	2.899939	-2.71212	0.037724	0.144636	-4.15147
APOBEC3A	-1.38577	2.226967	-2.71201	0.03773	0.144636	-4.15162
HECW1	1.215811	2.742472	2.704444	0.038095	0.145342	-4.1618
OPN1MW3	1.458504	5.324175	2.703201	0.038156	0.145433	-4.16347
HMOX1	1.16586	15.50007	2.699018	0.03836	0.145732	-4.16911
HSPA6	2.775734	5.449317	2.697569	0.03843	0.145893	-4.17106
AGTR1	-1.05628	3.009619	-2.69585	0.038515	0.146094	-4.17338
VLDLR	1.084493	3.966931	2.695567	0.038529	0.146094	-4.17375
AMELX	-1.33465	1.68462	-2.69547	0.038533	0.146094	-4.17388
SEZ6L	1.363172	3.241005	2.692339	0.038688	0.146192	-4.1781
FRG2B	-1.04297	2.29117	-2.69116	0.038746	0.146385	-4.1797
CACNG7	-1.37377	4.146888	-2.68923	0.038842	0.146611	-4.1823
SLC1A4	1.17491	6.199676	2.682774	0.039163	0.147199	-4.19099
B3GALT2	-1.11591	4.278768	-2.67988	0.039308	0.147554	-4.19489
PRAC2	1.155182	2.866808	2.679231	0.039341	0.147597	-4.19577
LIN28A	1.87665	3.385732	2.674663	0.039571	0.148059	-4.20192
ERICH6	-1.35848	2.245487	-2.67463	0.039573	0.148059	-4.20197
SERTM1	1.005102	3.390059	2.668432	0.039887	0.148666	-4.21033
OR4X2	-1.03532	5.529648	-2.66739	0.03994	0.148784	-4.21173
PLPPR1	1.381578	2.62451	2.658147	0.040416	0.149546	-4.22421
STC2	1.321829	8.106062	2.645616	0.04107	0.150972	-4.24112
VN1R5	-1.42231	4.45686	-2.64383	0.041164	0.15112	-4.24354
SLC22A3	1.188478	1.668986	2.640652	0.041332	0.15134	-4.24783
BRSK2	1.263595	2.879322	2.630706	0.041863	0.152366	-4.26127
PCDHA12	1.031548	2.97005	2.630405	0.041879	0.152398	-4.26167
DRD5	1.455513	2.885756	2.629943	0.041904	0.152419	-4.2623
MYBPHL	1.284807	2.886606	2.629883	0.041907	0.152419	-4.26238
TNFAIP8L2	-1.03111	2.464432	-2.62126	0.042374	0.153354	-4.27404

KDF1	1.221056	1.682234	2.611971	0.042883	0.154214	-4.2866
CFAP206	2.044605	3.085803	2.610765	0.04295	0.154318	-4.28823
KIAA0226L	1.285055	1.980926	2.607943	0.043106	0.15464	-4.29205
TP53TG5	1.184457	3.112005	2.607904	0.043108	0.15464	-4.2921
CDH17	1.195518	3.409804	2.607184	0.043148	0.15464	-4.29308
RGS18	1.055742	3.051226	2.606141	0.043206	0.154695	-4.29449
C8orf4	1.167651	10.41839	2.602742	0.043396	0.155265	-4.29909
OR2C1	1.028032	2.298954	2.598059	0.043658	0.155877	-4.30543
OR5H6	-1.17788	2.842089	-2.58811	0.044221	0.156875	-4.3189
ZG16	1.111389	5.649657	2.581173	0.044619	0.157695	-4.3283
C2CD4A	1.178313	4.909297	2.575826	0.044928	0.158195	-4.33554
CYP2A7	1.790886	2.51802	2.532299	0.04753	0.163385	-4.39458
MBOAT4	-1.36042	1.802384	-2.52946	0.047705	0.163744	-4.39843
SLC17A1	1.089865	1.768964	2.528716	0.047751	0.163803	-4.39944
ITPRID1	1.450352	1.82359	2.525358	0.04796	0.16427	-4.404
HERPUD1	2.246728	4.890955	2.514586	0.048635	0.165474	-4.41863
CCR3	1.107778	3.988567	2.512361	0.048775	0.165815	-4.42165
GOLGA7B	1.266718	2.89401	2.501889	0.049443	0.167308	-4.43588

Table S3. significant different expression of lncRNA after hypoxia treatment compared to controls.

Gene	logFC	AveExpr	t	P.Value	adj.P.Val	B
NONHSAT169401.1	3.385126	8.749768	31.64504	2.01E-07	0.010746	5.391117
lnc-ZFP42-13:1	4.239886	3.183658	30.6738	2.39E-07	0.010746	5.347319
ENST00000554254.1	3.506397	11.3501	29.0718	3.20E-07	0.010746	5.266895
lnc-TMEM30B-6:1	3.4897	8.14294	27.23594	4.56E-07	0.011503	5.160025
lnc-NDRG1-1:1	2.665422	14.16377	26.1066	5.75E-07	0.011591	5.085045
NONHSAT164393.1	2.894341	7.794281	23.38107	1.05E-06	0.017605	4.867882
NONHSAT207308.1	2.811383	7.586095	20.83091	1.96E-06	0.023413	4.604351
lnc-EMB-10:1	-2.52947	2.368854	-20.2393	2.29E-06	0.023413	4.532593
MSTRG.72335.13	2.509028	6.86993	20.14872	2.35E-06	0.023413	4.521207
ENST00000435984.1	3.589056	2.905821	20.1391	2.36E-06	0.023413	4.51999
NONHSAT199820.1	-2.16909	2.485435	-19.8422	2.55E-06	0.023413	4.481825
NONHSAT176902.1	2.208677	11.35296	19.34536	2.93E-06	0.024359	4.415152
NR_119376	2.999875	10.4005	19.09962	3.14E-06	0.024359	4.380811
lnc-DLX2-4:17	2.399449	9.446251	18.59119	3.63E-06	0.026177	4.306729
ENST00000500447.1	2.206166	9.987725	17.88737	4.48E-06	0.029439	4.19696
lnc-TMEM30B-9:1	2.468019	7.028313	17.63648	4.83E-06	0.029439	4.155662
LINC01606:17	1.929067	9.543196	17.42933	5.15E-06	0.029439	4.120657
ENST00000423942.1	2.083009	2.218274	16.95337	5.98E-06	0.029439	4.036982
lnc-DCP1A-2:1	-1.84601	1.99765	-16.9445	6.00E-06	0.029439	4.035375
MSTRG.71710.1	2.141711	8.154212	16.89304	6.10E-06	0.029439	4.02604
T105619	3.195992	6.957088	16.79867	6.29E-06	0.029439	4.00877
lnc-ANKRD45-1:1	6.600498	4.899349	16.65819	6.58E-06	0.029439	3.982703
lnc-SUSD1-7:3	2.691221	8.612615	16.42924	7.09E-06	0.029439	3.939288
lnc-TNFSF9-1:1	-1.74015	3.066328	-16.3241	7.34E-06	0.029439	3.918945
MSTRG.64854.3	2.623706	5.966334	16.28452	7.43E-06	0.029439	3.911231
NONHSAT205166.1	2.138153	2.144748	16.19667	7.65E-06	0.029439	3.893962
NONHSAT223579.1	1.699963	6.938563	16.10815	7.88E-06	0.029439	3.876379
NONHSAT169400.1	3.068961	8.331057	15.91296	8.42E-06	0.029795	3.83694
NONHSAT203600.1	2.240486	2.60194	15.86048	8.57E-06	0.029795	3.826177
ENST00000414769.2	1.701862	10.34493	15.68952	9.08E-06	0.030107	3.790644
lnc-ELMOD3-1:1	2.867022	2.467577	15.63491	9.26E-06	0.030107	3.779137
ENST00000575331.1	1.954994	5.076501	15.52474	9.62E-06	0.030297	3.755692
lnc-ERMP1-5:1	1.683474	10.14397	15.41397	9.99E-06	0.030533	3.731799
lnc-ZNF730-2:1	2.337201	2.402761	14.66389	1.31E-05	0.036651	3.561194
NONHSAT193198.1	1.952886	2.159728	14.57678	1.35E-05	0.036651	3.540337

lnc-ERRFI1-1:2	2.412136	5.264809	14.50805	1.38E-05	0.036651	3.523718
MSTRG.32096.87	1.914008	7.09918	14.49794	1.39E-05	0.036651	3.521261
MSTRG.42620.1	1.876669	1.984239	14.47084	1.40E-05	0.036651	3.514663
MSTRG.18225.2	1.615691	6.096783	14.44254	1.42E-05	0.036651	3.507745
ENST00000526906.1	2.102713	4.345403	14.22969	1.54E-05	0.037102	3.454936
lnc-FLRT2-10:2	-1.58351	2.016866	-14.1969	1.55E-05	0.037102	3.446683
T203949	1.382111	1.800344	14.18333	1.56E-05	0.037102	3.443245
lnc-ACSL6-1:1	1.381177	13.80033	14.13887	1.59E-05	0.037102	3.431967
MSTRG.13771.4	1.795194	1.984356	14.05616	1.64E-05	0.037102	3.410822
lnc-ZNF680-12:1	1.693985	2.21322	13.97469	1.69E-05	0.037102	3.389774
lnc-CCDC39-1:3	1.891	5.679224	13.92857	1.72E-05	0.037102	3.377764
NONHSAT205212.1	-1.64695	3.313277	-13.8906	1.75E-05	0.037102	3.367816
lnc-SMPX-6:1	1.949936	2.064336	13.86207	1.77E-05	0.037102	3.360319
lnc-LSMEM1-2:1	1.999148	2.176688	13.79179	1.81E-05	0.037304	3.341724
NONHSAT221185.1	2.273695	4.912055	13.73721	1.85E-05	0.037304	3.327166
NONHSAT151071.1	2.138729	7.274719	13.69202	1.89E-05	0.037304	3.315038
NONHSAT211760.1	2.331415	4.485163	13.64004	1.93E-05	0.037339	3.300996
lnc-KLHL1-11:1	-2.42501	2.265529	-13.5582	1.99E-05	0.0378	3.278704
ENST00000417539.1	2.223755	6.213617	13.46374	2.06E-05	0.0378	3.252679
ENST00000566733.2	1.497557	7.749182	13.44304	2.08E-05	0.0378	3.246933
MSTRG.12643.4	-1.61403	1.948225	-13.4215	2.10E-05	0.0378	3.240943
lnc-PLOD2-4:1	2.000494	8.529442	13.26917	2.23E-05	0.038787	3.198065
lnc-ADTRP-7:4	1.838051	6.897614	13.23794	2.26E-05	0.038787	3.18917
lnc-LYSMD3-2:5	1.884088	9.07316	13.22709	2.27E-05	0.038787	3.186072
MSTRG.19670.1	1.515215	1.797923	13.09952	2.39E-05	0.040167	3.149313
T118234	-1.81491	3.749327	-13.0369	2.45E-05	0.040532	3.131036
NONHSAT216919.1	-1.33757	1.843826	-12.9698	2.52E-05	0.040991	3.111316
NONHSAT207426.1	1.818031	2.007447	12.86871	2.63E-05	0.04179	3.081232
lnc-SLC15A4-25:1	2.437806	2.324355	12.84634	2.65E-05	0.04179	3.074524
lnc-CYP7A1-9:1	1.383205	1.767122	12.7796	2.73E-05	0.041797	3.054395
lnc-C2orf74-4:1	1.447643	13.85538	12.73655	2.78E-05	0.041797	3.041315
MSTRG.28235.1	-1.47329	7.907366	-12.736	2.78E-05	0.041797	3.041155
lnc-ARL4A-3:1	-1.78137	3.69673	-12.4646	3.11E-05	0.044389	2.956975
NONHSAT173357.1	2.451199	5.173184	12.42546	3.17E-05	0.044389	2.94457
ENST00000454444.1	2.204575	5.031175	12.406	3.19E-05	0.044389	2.938383
MSTRG.35148.1	1.436885	1.973669	12.39429	3.21E-05	0.044389	2.934654
ENST00000438659.2	1.553407	1.844871	12.39222	3.21E-05	0.044389	2.933994
MSTRG.19441.1	-2.58539	2.758552	-12.3918	3.21E-05	0.044389	2.93386
lnc-FYB1-1:2	-1.86483	2.060094	-12.3324	3.30E-05	0.044401	2.914848
ENST00000561295.4	1.563872	1.845409	12.30274	3.34E-05	0.044401	2.905295
lnc-SLC2A14-2:1	1.37554	12.51933	12.27787	3.38E-05	0.044401	2.897259
NONHSAT188155.1	-1.67124	1.960524	-12.2595	3.40E-05	0.044401	2.891293
lnc-ADGRD1-3:1	2.159217	2.192538	12.23784	3.43E-05	0.044401	2.884268
NONHSAT167733.1	2.108993	2.166463	12.13381	3.59E-05	0.045204	2.850176
ENST00000665254.1	-2.06985	2.415472	-12.1338	3.59E-05	0.045204	2.850171
LINC01811:26	1.620877	7.61744	12.11038	3.63E-05	0.045204	2.842431
NR_026706	-2.33875	2.247882	-11.9389	3.92E-05	0.047101	2.785016
lnc-FAM13C-5:1	2.300352	2.207027	11.92245	3.95E-05	0.047101	2.779432

lnc-SNTG1-9:1	-1.42112	5.149614	-11.9152	3.96E-05	0.047101	2.776975
ENST00000567966.1	1.836429	2.011367	11.90845	3.97E-05	0.047101	2.774676
ENST00000522028.1	1.621311	2.008302	11.86732	4.04E-05	0.047417	2.760648
NONHSAT224142.1	-1.54219	6.300722	-11.7561	4.25E-05	0.049272	2.722341
lnc-XKR5-3:1	1.488846	9.058674	11.72479	4.31E-05	0.049359	2.711431
DIRC3-AS1:11	1.572639	4.499535	11.70201	4.36E-05	0.049359	2.703477
NONHSAT214644.1	1.576467	1.895388	11.65002	4.46E-05	0.049976	2.685229
lnc-ZNF669-1:1	1.382797	1.784581	11.56069	4.65E-05	0.051087	2.653579
lnc-SEL1L3-4:1	-1.33132	7.930899	-11.5199	4.73E-05	0.051087	2.638997
NONHSAT164791.1	-1.78489	2.825476	-11.4838	4.81E-05	0.051087	2.626032
lnc-DCAF12L2-2:1	-1.46162	6.405532	-11.4814	4.82E-05	0.051087	2.625141
lnc-WRNIP1-34:2	1.642791	1.889444	11.42884	4.94E-05	0.051087	2.606149
lnc-CERS4-3:1	-1.61509	2.042108	-11.4124	4.98E-05	0.051087	2.600164
NONHSAT167217.1	-1.67626	2.039453	-11.4056	4.99E-05	0.051087	2.597711
T273532	1.159687	1.676194	11.40361	5.00E-05	0.051087	2.596978
MSTRG.55402.1	-1.50691	4.218918	-11.3779	5.06E-05	0.051087	2.587601
lnc-ADGRV1-8:1	1.939478	6.784161	11.32983	5.17E-05	0.051087	2.569971
lnc-WASHC5-11:3	2.141746	2.481096	11.27765	5.30E-05	0.051087	2.550707
lnc-ZSWIM2-6:5	-1.56801	3.257526	-11.2683	5.32E-05	0.051087	2.547245
lnc-SPATA31A6-4:1	-1.19796	3.151868	-11.2629	5.33E-05	0.051087	2.545247
ENST00000445174.5	1.167634	12.84619	11.24527	5.38E-05	0.051087	2.53868
lnc-MYOF-3:1	-1.39521	6.29811	-11.2408	5.39E-05	0.051087	2.537018
T379934	2.474162	2.640527	11.19037	5.52E-05	0.051087	2.518171
ENST00000608775.1	1.424469	7.256506	11.18726	5.53E-05	0.051087	2.517003
lnc-CCDC38-7:1	1.171574	10.56158	11.1564	5.61E-05	0.051087	2.505403
ENST00000656747.1	1.169799	9.216324	11.152	5.62E-05	0.051087	2.503746
NONHSAT186902.1	2.295144	2.370188	11.10979	5.74E-05	0.051087	2.487785
lnc-KLF6-12:3	2.433022	2.476319	11.10634	5.74E-05	0.051087	2.486478
lnc-THSD7A-8:1	-1.56445	2.826412	-11.1021	5.76E-05	0.051087	2.484875
ENST00000625043.1	2.07187	5.756727	11.09398	5.78E-05	0.051087	2.481784
ENST00000557733.1	1.161881	6.175272	11.06302	5.86E-05	0.051087	2.469995
lnc-TEX2-1:1	1.83323	1.979728	11.04022	5.93E-05	0.051087	2.46128
LUCAT1:14	2.548327	5.456195	11.02866	5.96E-05	0.051087	2.456851
lnc-EDEM3-7:3	1.454854	7.313952	11.02068	5.98E-05	0.051087	2.453789
ENST00000428335.1	-2.14148	2.391653	-11.0143	6.00E-05	0.051087	2.451342
lnc-EXTL3-7:1	-1.43105	1.827103	-10.9884	6.08E-05	0.051087	2.441383
NONHSAT187468.1	1.450289	1.789734	10.97955	6.10E-05	0.051087	2.437954
lnc-ABCG5-3:1	1.750118	1.985067	10.97045	6.13E-05	0.051087	2.434439
ENST00000339037.4	1.15386	12.6281	10.95202	6.19E-05	0.05112	2.427305
lnc-TRIO-1:3	1.489674	7.47941	10.92244	6.27E-05	0.051433	2.41582
NONHSAT171432.1	1.416245	11.72569	10.89307	6.36E-05	0.051749	2.404366
NONHSAT172926.1	1.457971	2.530844	10.87025	6.43E-05	0.051906	2.395438
ENST00000665013.1	1.623082	7.638839	10.81058	6.62E-05	0.052851	2.37195
ENST00000670284.1	-1.96784	2.288065	-10.7781	6.73E-05	0.052851	2.359097
NONHSAT149210.1	1.507067	2.59875	10.77729	6.73E-05	0.052851	2.358763
MSTRG.5865.1	-1.43584	3.648265	-10.7686	6.76E-05	0.052851	2.355305
ENST00000419889.1	-1.22401	3.317419	-10.7395	6.86E-05	0.053198	2.343712
NONHSAT205252.1	1.714521	1.879607	10.6977	7.00E-05	0.053472	2.326986

ENST00000663862.1	-1.75294	4.905028	-10.683	7.05E-05	0.053472	2.321067
ENST00000633779.1	-1.67271	2.020731	-10.6827	7.05E-05	0.053472	2.320953
ENST00000416909.1	-1.92365	2.217932	-10.6415	7.20E-05	0.053793	2.304355
lnc-MLXIP-1:1	1.237569	8.221728	10.64032	7.20E-05	0.053793	2.303859
NR_110223	-1.39703	5.223138	-10.5868	7.40E-05	0.05427	2.282102
MSTRG.64493.1	1.472865	1.907211	10.58625	7.40E-05	0.05427	2.281895
ENST00000668962.1	1.549125	6.641342	10.56383	7.48E-05	0.05427	2.272734
lnc-NDRG2-7:1	-1.74779	1.988018	-10.5638	7.48E-05	0.05427	2.272707
NONHSAT177958.1	1.379125	1.760026	10.49295	7.75E-05	0.055656	2.243599
NONHSAT201592.1	1.184047	1.71896	10.48476	7.78E-05	0.055656	2.240213
lnc-ZNF680-14:1	1.079367	9.132593	10.43762	7.97E-05	0.055987	2.220647
ENST00000615349.1	-1.20246	8.701239	-10.4317	7.99E-05	0.055987	2.218201
MSTRG.44248.1	1.363052	8.059717	10.40551	8.10E-05	0.055987	2.207246
lnc-HTR1B-2:7	1.440608	6.067469	10.4051	8.10E-05	0.055987	2.207077
lnc-TCIM-4:1	-1.69067	2.451164	-10.4037	8.11E-05	0.055987	2.206511
NONHSAT181690.1	1.385946	1.77923	10.37014	8.25E-05	0.05599	2.192416
ENST00000567714.1	1.744161	8.567219	10.36439	8.27E-05	0.05599	2.189998
ENST00000432230.6	2.979175	2.86938	10.33492	8.39E-05	0.05599	2.177575
lnc-ACSL1-4:1	1.609528	1.909164	10.33038	8.41E-05	0.05599	2.175657
lnc-CENPP-14:1	-1.15893	5.942772	-10.3281	8.42E-05	0.05599	2.174696
lnc-PRDM13-9:1	1.033148	9.843964	10.32235	8.45E-05	0.05599	2.172262
NONHSAT213552.1	1.494312	1.914304	10.30055	8.54E-05	0.05599	2.163022
lnc-FYTTD1-8:4	-1.9258	4.221903	-10.2876	8.60E-05	0.05599	2.157527
ENST00000485473.1	1.800413	7.121982	10.2859	8.61E-05	0.05599	2.156795
ENST00000450443.1	1.434128	9.38344	10.22339	8.89E-05	0.056295	2.130087
T285881	3.523575	3.182184	10.22338	8.89E-05	0.056295	2.130083
MIR503HG:17	1.47937	6.00881	10.21578	8.92E-05	0.056295	2.126819
NONHSAT185724.1	1.312149	1.855077	10.2139	8.93E-05	0.056295	2.126011
lnc-TMEM268-3:1	1.176173	1.701235	10.18547	9.06E-05	0.056295	2.113769
NONHSAT195834.1	-1.0422	8.031084	-10.1836	9.07E-05	0.056295	2.112965
T006816	1.801282	2.002481	10.17915	9.09E-05	0.056295	2.11104
NONHSAT154126.1	-1.18436	8.229173	-10.1772	9.10E-05	0.056295	2.110202
lnc-ALG10-4:1	-1.11324	8.617555	-10.1652	9.16E-05	0.056298	2.105025
NONHSAT195317.1	-1.47245	5.646659	-10.1364	9.29E-05	0.056441	2.092528
NONHSAT183657.1	1.51988	2.079577	10.13445	9.30E-05	0.056441	2.091678
NONHSAT190719.1	1.011398	7.927458	10.11297	9.41E-05	0.056441	2.082325
NONHSAT190319.1	1.493353	1.795306	10.10373	9.45E-05	0.056441	2.078294
T288823	1.072423	10.22029	10.09245	9.51E-05	0.056441	2.073368
NONHSAT208275.1	1.395094	5.106222	10.08689	9.54E-05	0.056441	2.070935
MSTRG.59967.1	-1.82712	2.656438	-10.0797	9.57E-05	0.056441	2.067784
lnc-TMEM14A-1:3	1.131658	6.989113	10.06453	9.65E-05	0.056557	2.061136
T376410	1.579148	5.724197	10.04137	9.76E-05	0.056754	2.050955
MSTRG.65423.3	2.001875	9.433927	10.01068	9.92E-05	0.056754	2.037411
lnc-HSD17B2-1:4	-1.57398	3.184429	-10.0064	9.94E-05	0.056754	2.035523
ENST00000660983.1	1.009914	7.320945	10.00377	9.96E-05	0.056754	2.034352
lnc-RNASE11-1:1	-1.3776	9.560812	-9.99807	9.99E-05	0.056754	2.031827
NR_026850	1.052832	11.21156	9.992252	0.0001	0.056754	2.029247
MSTRG.37408.2	1.897786	5.556845	9.966838	0.000102	0.05689	2.017956

lnc-RBMX-6:1	-1.7316	1.984444	-9.9664	0.000102	0.05689	2.017761
NONHSAT178170.1	1.003801	12.39492	9.938952	0.000103	0.057201	2.005519
MSTRG.16372.1	1.56785	1.929874	9.928206	0.000104	0.057201	2.000713
ENST00000431293.2	1.974787	5.939624	9.924508	0.000104	0.057201	1.999058
ENST00000607047.1	-1.06294	9.34261	-9.91433	0.000104	0.057201	1.994498
lnc-POC5-3:1	-1.26655	8.381891	-9.86266	0.000107	0.058202	1.97124
lnc-BMPER-6:1	-1.24182	6.822127	-9.85489	0.000108	0.058202	1.967729
MSTRG.3164.1	-1.17974	5.751404	-9.83837	0.000109	0.058202	1.960245
ENST00000490375.1	1.481731	9.640331	9.827544	0.000109	0.058202	1.955334
ENST00000483262.1	1.372244	5.715399	9.810078	0.00011	0.058202	1.947394
lnc-SPARCL1-4:1	-1.09917	5.393044	-9.80284	0.000111	0.058202	1.9441
NONHSAT218479.1	1.569705	2.003974	9.801438	0.000111	0.058202	1.943459
lnc-NCAM2-12:1	-1.74896	2.10691	-9.77397	0.000112	0.058757	1.930913
NONHSAT163833.1	-1.55603	2.637891	-9.75013	0.000114	0.058991	1.919986
ENST00000455967.1	-1.11207	4.05816	-9.74729	0.000114	0.058991	1.918684
lnc-GMPS-4:1	-1.23211	6.058312	-9.73161	0.000115	0.059186	1.911473
ENST00000442442.1	1.29551	1.746089	9.717698	0.000116	0.059327	1.905063
NONHSAT165231.1	1.527177	2.131755	9.686102	0.000118	0.059818	1.890455
MSTRG.31397.1	1.456356	1.865925	9.678918	0.000118	0.059818	1.887124
ENST00000606622.1	-1.40714	1.891721	-9.64965	0.00012	0.059818	1.873517
lnc-CPNE1-2:1	2.059764	4.517651	9.641532	0.000121	0.059818	1.869735
lnc-HRASLS5-2:1	-1.35049	8.262739	-9.63917	0.000121	0.059818	1.868635
lnc-BPY2-5:1	-1.19433	3.599703	-9.6302	0.000122	0.059818	1.864445
NONHSAT195038.1	1.107285	1.667123	9.627303	0.000122	0.059818	1.863093
lnc-NSFL1C-1:1	1.179583	4.971408	9.62691	0.000122	0.059818	1.862909
NR_033972	1.232819	7.344923	9.619727	0.000122	0.059818	1.85955
NONHSAT154564.1	-1.35189	1.968172	-9.59731	0.000124	0.060001	1.849046
ENST00000585189.1	2.034221	4.339076	9.587992	0.000124	0.060001	1.844669
ENST00000450800.1	1.037591	1.596912	9.587508	0.000124	0.060001	1.844441
NONHSAT211935.1	1.283021	1.840055	9.544627	0.000127	0.061111	1.824224
NONHSAT163300.1	-1.90459	5.131295	-9.52745	0.000129	0.061111	1.81609
NONHSAT172103.1	2.245474	2.143324	9.521214	0.000129	0.061111	1.813132
lnc-OLFML3-4:1	2.087382	2.369737	9.516069	0.000129	0.061111	1.81069
ENST00000455974.1	1.6507	7.728536	9.507285	0.00013	0.061111	1.806516
NONHSAT181351.1	1.129812	8.787812	9.502166	0.00013	0.061111	1.804081
lnc-FZD8-3:1	1.488546	2.05417	9.463884	0.000133	0.061861	1.785813
NONHSAT205189.1	-1.06254	1.608765	-9.44636	0.000134	0.061861	1.777419
ENST00000511867.1	-1.01927	1.655647	-9.43611	0.000135	0.061861	1.772497
T073019	3.516486	3.435794	9.430282	0.000136	0.061861	1.769695
lnc-USP44-2:1	1.162296	10.15818	9.428009	0.000136	0.061861	1.768602
NONHSAT206327.1	1.55874	5.646561	9.419282	0.000136	0.061861	1.764401
MSTRG.39833.2	-1.12995	7.489377	-9.41373	0.000137	0.061861	1.761726
lnc-PXDC1-15:6	1.459411	7.569173	9.404723	0.000137	0.061893	1.757382
ENST00000515728.1	1.174242	3.554642	9.383067	0.000139	0.061906	1.746914
NONHSAT187991.1	-1.4778	1.86914	-9.36748	0.00014	0.061906	1.739356
lnc-HNRNPU-8:1	-1.0988	10.33189	-9.36626	0.00014	0.061906	1.738764
NONHSAT196380.1	-2.77922	3.346385	-9.36601	0.00014	0.061906	1.738644
lnc-NPFFR2-2:2	-1.12062	9.219469	-9.35573	0.000141	0.061906	1.733653

NONHSAT217842.1	1.381435	1.867265	9.323385	0.000144	0.061906	1.717892
NONHSAT199539.1	2.089954	2.365972	9.30485	0.000145	0.061906	1.708828
lnc-MICAL2-1:4	1.304949	7.706686	9.291621	0.000146	0.061906	1.702343
lnc-ANKRD42-5:1	-1.49494	4.728624	-9.28894	0.000147	0.061906	1.701027
NONHSAT185991.1	1.495765	1.942804	9.283559	0.000147	0.061906	1.698385
lnc-ATXN7-14:1	1.813658	3.84389	9.270355	0.000148	0.061906	1.691893
lnc-BEGAIN-3:1	2.145942	2.090605	9.263361	0.000149	0.061906	1.688449
ENST00000624102.1	-1.45309	6.69888	-9.26108	0.000149	0.061906	1.687324
MSTRG.33020.1	1.315028	4.047195	9.252156	0.00015	0.061906	1.682924
ENST00000437764.5	1.093291	11.48909	9.248781	0.00015	0.061906	1.681258
lnc-ST6GALNAC2-1:1	1.870217	2.029618	9.246185	0.00015	0.061906	1.679976
NONHSAT189471.1	1.447462	1.764647	9.229686	0.000152	0.061906	1.671817
ENST00000664141.1	-1.6936	3.205841	-9.21636	0.000153	0.061906	1.665215
lnc-PIGM-4:1	1.347802	2.236199	9.213855	0.000153	0.061906	1.66397
NR_015421	1.107202	8.312133	9.212553	0.000153	0.061906	1.663324
lnc-PITRM1-2:8	1.045829	4.362114	9.204923	0.000154	0.061906	1.659534
lnc-PRSS16-1:1	-1.18254	5.172584	-9.20308	0.000154	0.061906	1.658617
ENST00000512406.5	1.236721	1.745881	9.197951	0.000154	0.061906	1.656068
LINC01638:16	2.184171	3.546558	9.197836	0.000154	0.061906	1.656011
ENST00000662762.1	-1.164	6.48583	-9.18326	0.000156	0.061906	1.648754
NONHSAT169246.1	1.324701	2.037405	9.178223	0.000156	0.061906	1.646241
TMEM147-AS1:8	-1.13865	9.474526	-9.17078	0.000157	0.061906	1.642526
lnc-MPZL3-4:1	-1.6116	9.541349	-9.16099	0.000158	0.061906	1.637634
lnc-SP8-5:1	1.262284	6.97691	9.160599	0.000158	0.061906	1.637438
NONHSAT193263.1	1.240815	1.864801	9.159163	0.000158	0.061906	1.63672
NONHSAT205786.1	1.982739	2.721083	9.143079	0.000159	0.061906	1.628664
lnc-UBAP1-6:2	-1.10415	2.28273	-9.14299	0.000159	0.061906	1.628617
ENST00000527726.1	1.238846	6.191172	9.141771	0.000159	0.061906	1.628008
lnc-SLC2A3-4:1	1.778798	1.944338	9.138655	0.00016	0.061906	1.626445
MSTRG.20529.1	-1.01397	8.257091	-9.12374	0.000161	0.062006	1.618954
MSTRG.31811.3	-1.36128	2.879392	-9.12235	0.000161	0.062006	1.618253
lnc-BTBD19-1:1	1.041877	8.136049	9.09873	0.000163	0.062607	1.606354
lnc-MRPL14-4:1	2.814253	2.444228	9.06892	0.000166	0.06281	1.591277
lnc-STBD1-5:2	-1.47463	8.035625	-9.06008	0.000167	0.06281	1.586794
lnc-CDY1B-13:1	-1.28897	4.648343	-9.05958	0.000167	0.06281	1.586537
lnc-GBP5-5:1	1.541351	2.034498	9.059506	0.000167	0.06281	1.586501
NONHSAT211369.1	-1.01703	5.823547	-9.0536	0.000168	0.06281	1.583504
NONHSAT221322.1	-1.70654	2.51824	-9.04417	0.000168	0.062917	1.578708
NONHSAT193723.1	1.619413	8.434719	9.021981	0.000171	0.063489	1.5674
MSTRG.66702.1	1.792722	2.10925	8.999333	0.000173	0.06365	1.555821
MSTRG.14601.5	2.039225	2.245126	8.988678	0.000174	0.06365	1.55036
NONHSAT176375.1	1.446846	2.998872	8.98445	0.000174	0.06365	1.548191
lnc-LIMS3-2:5	1.010749	7.60258	8.980776	0.000175	0.06365	1.546304
NONHSAT167431.1	1.831199	2.206503	8.968111	0.000176	0.06365	1.539795
NONHSAT221753.1	1.238607	1.749036	8.967102	0.000176	0.06365	1.539276
lnc-SRC-3:1	-1.10149	3.66183	-8.94658	0.000178	0.06418	1.528698
ENST00000455576.1	1.587875	1.992821	8.937126	0.000179	0.064303	1.523817
lnc-MCM3-3:2	2.498624	2.505425	8.904345	0.000183	0.065306	1.506833

NR_033752	-1.52776	7.571411	-8.89092	0.000184	0.065576	1.499853
MSTRG.35588.1	-1.04667	6.65138	-8.88511	0.000185	0.065576	1.49683
lnc-RAB39A-2:1	-1.90143	4.570954	-8.86442	0.000187	0.065978	1.486039
ENST00000491934.2	1.709546	7.198127	8.862615	0.000187	0.065978	1.485093
NONHSAT209573.1	2.208331	2.418039	8.829816	0.000191	0.066764	1.467912
ENST00000623995.1	-1.13236	7.904707	-8.82452	0.000191	0.066764	1.465131
SLC7A11-AS1:6	-1.07919	7.173882	-8.81472	0.000192	0.066861	1.459977
lnc-TTC38-1:1	1.317053	4.069752	8.788353	0.000195	0.066861	1.446073
ENST00000623678.1	1.159113	8.924593	8.775114	0.000197	0.066861	1.439071
NONHSAT224232.1	1.050327	1.590258	8.77298	0.000197	0.066861	1.437941
NR_004384	-1.17918	13.35879	-8.77172	0.000197	0.066861	1.437274
NONHSAT152008.1	1.830413	3.214727	8.764939	0.000198	0.066861	1.433681
T230587	1.185975	1.759526	8.760862	0.000199	0.066861	1.431519
T163143	-1.56072	2.272954	-8.7562	0.000199	0.066861	1.429044
T172702	1.120726	1.63566	8.745429	0.0002	0.066861	1.423322
MSTRG.24999.1	-1.29541	5.881398	-8.74448	0.000201	0.066861	1.422817
T009292	2.205088	2.275848	8.742718	0.000201	0.066861	1.42188
NONHSAT215593.1	-1.02254	5.521949	-8.74171	0.000201	0.066861	1.421342
ENST00000652227.1	1.060071	8.040943	8.718098	0.000204	0.067357	1.40876
lnc-ADGRV1-2:1	1.835187	3.560256	8.710352	0.000205	0.067446	1.404623
lnc-BTN3A2-1:4	-1.45236	1.908019	-8.69826	0.000206	0.067539	1.398152
MSTRG.59871.1	2.102992	2.222659	8.68858	0.000207	0.06766	1.392967
NONHSAT212824.1	1.328359	1.78031	8.640497	0.000213	0.068926	1.367091
lnc-NSMCE1-6:1	1.854549	2.156282	8.640428	0.000213	0.068926	1.367053
MSTRG.14961.7	-1.04012	6.624678	-8.63917	0.000214	0.068926	1.366372
NONHSAT164688.1	1.961761	2.27978	8.63405	0.000214	0.068926	1.363607
MSTRG.63783.1	-2.10985	2.820455	-8.63055	0.000215	0.068926	1.361715
MSTRG.44418.1	1.209927	1.648402	8.609498	0.000217	0.069342	1.35031
NONHSAT183089.1	1.901244	5.964434	8.607154	0.000218	0.069342	1.349038
ENST00000660762.1	-1.68209	2.239874	-8.60461	0.000218	0.069342	1.347656
lnc-OR11H1-3:1	1.027286	11.15256	8.59672	0.000219	0.069452	1.34337
lnc-UHRF1BP1L-4:1	1.962189	2.296992	8.565669	0.000223	0.069932	1.326451
MSTRG.26947.1	2.254551	2.202358	8.564871	0.000223	0.069932	1.326015
MSTRG.68207.1	1.254519	6.154853	8.564188	0.000223	0.069932	1.325643
lnc-ALDH9A1-3:2	-1.35995	2.644588	-8.55695	0.000224	0.069932	1.321686
NONHSAT179128.1	-1.1499	10.08924	-8.55445	0.000225	0.069932	1.320322
NONHSAT163249.1	1.674235	4.324947	8.553618	0.000225	0.069932	1.319864
NONHSAT195938.1	-1.04929	1.588025	-8.53413	0.000227	0.069932	1.309184
lnc-ALDH3B2-2:1	-1.44675	4.648861	-8.52239	0.000229	0.069932	1.302736
LINC01234:6	-1.16537	7.169994	-8.51743	0.00023	0.069932	1.300012
lnc-KCNS3-3:4	1.146596	6.671245	8.517342	0.00023	0.069932	1.299962
lnc-FMR1-3:1	-1.37668	10.19833	-8.50971	0.000231	0.069932	1.295759
ENST00000668970.1	1.439525	6.547731	8.503744	0.000232	0.069932	1.292475
NONHSAT221803.1	1.570445	1.964472	8.501996	0.000232	0.069932	1.291511
NONHSAT188005.1	-1.52052	5.586582	-8.4969	0.000233	0.069932	1.2887
lnc-SLC25A51-4:2	1.587886	7.035218	8.493998	0.000233	0.069932	1.287098
lnc-DPH1-3:1	-1.10469	1.58434	-8.47534	0.000236	0.070154	1.276783
NONHSAT206058.1	1.334471	1.738681	8.466815	0.000237	0.070259	1.272062

T357080	1.554607	2.168049	8.453053	0.000239	0.070554	1.264426
NONHSAT168794.1	-1.88211	2.237025	-8.45028	0.000239	0.070554	1.262887
lnc-RMI1-8:1	-1.24654	7.712847	-8.4236	0.000243	0.0713	1.248034
ENST00000544421.1	-1.27089	7.569378	-8.39712	0.000247	0.071861	1.233227
NONHSAT180069.1	1.061727	4.804231	8.392388	0.000248	0.071861	1.230578
NONHSAT213595.1	-1.10719	5.820858	-8.37825	0.00025	0.071861	1.222645
lnc-COL6A5-3:1	1.25721	1.814675	8.376198	0.00025	0.071861	1.221492
MSTRG.40230.1	1.169886	1.781794	8.372368	0.000251	0.071861	1.21934
NR_034147	1.204896	6.15227	8.36933	0.000251	0.071861	1.217631
lnc-NEURL4-3:3	1.19428	6.041026	8.359508	0.000253	0.071861	1.212103
ENST00000411427.3	-1.7765	6.215933	-8.3456	0.000255	0.071861	1.204259
MSTRG.22909.1	1.802832	2.03503	8.330892	0.000257	0.071861	1.19595
NONHSAT152748.1	1.256815	1.913024	8.319979	0.000259	0.071861	1.189771
NONHSAT213325.1	-1.38594	2.298649	-8.3193	0.000259	0.071861	1.189385
lnc-OR51B4-3:1	-1.1575	6.511834	-8.31139	0.000261	0.071861	1.184899
NONHSAT210691.1	-1.18036	1.809499	-8.30656	0.000261	0.071861	1.18216
lnc-SLC2A12-11:1	-1.03619	3.112884	-8.29969	0.000262	0.071861	1.178257
lnc-ACER2-4:1	-1.02391	9.728321	-8.29878	0.000263	0.071861	1.177737
NONHSAT161348.1	-1.54199	2.080095	-8.29769	0.000263	0.071861	1.177121
lnc-TMBIM4-2:1	1.030413	2.337252	8.296846	0.000263	0.071861	1.17664
NONHSAT197456.1	1.741594	1.977398	8.294286	0.000263	0.071861	1.175184
lnc-GPT2-13:1	1.157909	2.426038	8.291206	0.000264	0.071861	1.173431
lnc-C2CD4B-7:1	-1.21309	6.906156	-8.28405	0.000265	0.071861	1.169359
lnc-RIOX2-7:1	-1.01631	8.861398	-8.27601	0.000266	0.071861	1.164771
NR_040079	1.151897	6.074681	8.271499	0.000267	0.071861	1.1622
lnc-RXFP3-2:1	-1.62116	7.418752	-8.26569	0.000268	0.071861	1.158882
ENST00000669185.1	1.361975	1.836051	8.262403	0.000269	0.071861	1.157004
ENST00000607898.1	1.366006	5.677944	8.261686	0.000269	0.071861	1.156594
lnc-RPL11-7:1	-1.12803	10.44235	-8.25733	0.000269	0.071861	1.154105
lnc-MYOM1-10:2	-1.59649	3.035424	-8.2432	0.000272	0.071861	1.146014
MSTRG.56604.1	1.637767	1.919911	8.242698	0.000272	0.071861	1.145724
lnc-RNGTT-1:1	1.288603	2.017987	8.23728	0.000273	0.071861	1.142617
NONHSAT223451.1	1.413671	1.784225	8.231187	0.000274	0.071861	1.13912
ENST00000668509.1	2.324937	2.619519	8.228283	0.000274	0.071861	1.137451
MSTRG.64592.1	-1.61472	5.290136	-8.21924	0.000276	0.071966	1.132254
T206470	1.533986	6.023362	8.213269	0.000277	0.071966	1.128816
ENST00000521444.1	-1.38739	2.303782	-8.20957	0.000278	0.071966	1.126683
lnc-GPR65-19:2	-1.31293	11.48399	-8.20558	0.000278	0.071966	1.124383
NONHSAT205462.1	-1.78875	2.219144	-8.20527	0.000278	0.071966	1.124209
lnc-AASS-1:1	-1.63856	4.121499	-8.18804	0.000281	0.072147	1.114259
MSTRG.27714.1	-1.34688	4.1152	-8.18542	0.000282	0.072147	1.112746
MSTRG.26604.1	1.552784	4.255779	8.180906	0.000283	0.072147	1.110135
lnc-PRPF18-8:9	1.724606	1.956365	8.175568	0.000284	0.072206	1.107044
NONHSAT197558.1	1.265305	1.78981	8.163227	0.000286	0.072417	1.099891
lnc-MAFB-7:1	-1.06996	5.933971	-8.15455	0.000287	0.072417	1.094854
ENST00000609720.1	1.258562	1.697937	8.152145	0.000288	0.072417	1.093456
NONHSAT174261.1	1.087711	1.891179	8.14697	0.000289	0.072417	1.090447
ENST00000510767.5	1.210352	6.974145	8.132757	0.000291	0.072886	1.082171

lnc-NPY5R-9:1	-1.12903	4.421733	-8.11489	0.000295	0.072955	1.071745
lnc-USP9Y-10:1	-1.22909	1.722461	-8.11289	0.000295	0.072955	1.070574
NONHSAT209338.1	1.333401	5.381597	8.108976	0.000296	0.072955	1.068285
NR_024062	-1.00497	9.590439	-8.10684	0.000296	0.072955	1.067033
lnc-TTLL12-1:2	1.769466	5.034882	8.105482	0.000296	0.072955	1.06624
ENST00000658867.1	-1.24135	8.151774	-8.10395	0.000297	0.072955	1.065344
NONHSAT172460.1	-2.02867	2.371683	-8.09654	0.000298	0.073119	1.061004
lnc-SELENOF-11:1	1.949687	2.021291	8.083031	0.000301	0.073419	1.053077
MSTRG.64319.1	1.4417	1.940589	8.059387	0.000305	0.073878	1.039166
NONHSAT224379.1	1.664046	2.052619	8.055347	0.000306	0.073878	1.036784
lnc-CDY2B-13:1	-1.54931	5.531772	-8.04499	0.000308	0.073878	1.030672
lnc-SNX14-6:1	-1.1789	6.620998	-8.04233	0.000308	0.073878	1.0291
NONHSAT158450.1	-1.00661	6.359763	-8.03432	0.00031	0.073993	1.024361
NONHSAT187771.1	1.634269	4.424868	8.029255	0.000311	0.073993	1.021365
lnc-SHISAL2B-2:1	-1.02221	9.449075	-8.02873	0.000311	0.073993	1.021054
ENST00000655217.1	1.28629	2.193416	8.000793	0.000317	0.074897	1.004476
MSTRG.54604.1	-1.10059	3.900426	-7.99807	0.000317	0.074897	1.002857
ENST00000563521.2	-1.08323	4.008843	-7.99498	0.000318	0.074897	1.001019
NONHSAT155095.1	-1.41203	2.005167	-7.98268	0.00032	0.075039	0.993688
NONHSAT148836.1	1.286633	5.643157	7.979907	0.000321	0.075039	0.992036
MSTRG.26614.1	1.0182	13.26447	7.972794	0.000322	0.075039	0.98779
NONHSAT219875.1	1.75617	6.628182	7.972353	0.000323	0.075039	0.987526
lnc-SEMA3A-1:1	-1.08269	8.481122	-7.97027	0.000323	0.075039	0.986282
lnc-CSTF3-5:1	2.030651	2.129303	7.942676	0.000329	0.076077	0.969762
ENST00000432488.1	-1.1528	4.080509	-7.93045	0.000331	0.076077	0.962418
lnc-MFNG-1:1	1.253657	8.002204	7.928005	0.000332	0.076077	0.960949
lnc-UCP2-2:2	-1.02847	8.315984	-7.9248	0.000333	0.076077	0.95902
MSTRG.57936.1	1.350029	1.705377	7.924448	0.000333	0.076077	0.95881
lnc-DUSP26-6:1	-2.3061	2.842198	-7.92047	0.000333	0.076077	0.956413
MSTRG.18011.9	-1.24184	7.534443	-7.91445	0.000335	0.076157	0.95279
ENST00000671654.1	1.691708	1.972816	7.907111	0.000336	0.076218	0.948365
lnc-SLC39A10-7:1	1.986376	2.19473	7.901839	0.000338	0.076293	0.945184
NR_028328	-2.26689	7.11574	-7.89154	0.00034	0.076472	0.93896
ENST00000623604.2	1.22556	3.348973	7.8839	0.000341	0.076522	0.934339
ENST00000450930.1	1.145375	5.928922	7.879812	0.000342	0.076522	0.931864
NONHSAT155404.1	1.275053	4.523362	7.869067	0.000345	0.076522	0.925349
T362344	-1.20322	7.163922	-7.83943	0.000351	0.077272	0.907328
MSTRG.63976.1	1.016547	1.521897	7.834883	0.000352	0.077272	0.904554
ENST00000433460.1	1.469556	5.129982	7.831906	0.000353	0.077272	0.902738
ENST00000623980.1	-1.63072	7.313646	-7.83074	0.000353	0.077272	0.902028
lnc-PHLDA1-3:1	-1.1349	9.551852	-7.8284	0.000354	0.077272	0.900599
lnc-CA9-2:1	-1.64181	4.253203	-7.81914	0.000356	0.077272	0.894943
MSTRG.57589.1	-1.14059	1.709746	-7.81786	0.000356	0.077272	0.894161
lnc-RHBDL2-1:1	-1.16711	5.804577	-7.81488	0.000357	0.077272	0.892333
NONHSAT156588.1	1.152716	4.279163	7.811536	0.000358	0.077272	0.890289
lnc-ADAT2-2:1	1.090297	5.198359	7.790012	0.000363	0.078198	0.877092
lnc-PIK3C3-14:1	-1.3462	4.637713	-7.78571	0.000364	0.07825	0.874452
MSTRG.27436.1	1.624882	2.184364	7.770803	0.000368	0.078682	0.865278

NONHSAT157747.1	-1.20897	11.35986	-7.75254	0.000372	0.079461	0.854013
ENST00000657495.1	1.150433	1.798782	7.745281	0.000374	0.079672	0.849527
MSTRG.4616.1	1.953931	2.061542	7.736314	0.000376	0.0797	0.843978
NONHSAT165061.1	1.406811	3.485397	7.732603	0.000377	0.0797	0.84168
MSTRG.63251.25	1.049263	5.456071	7.731931	0.000377	0.0797	0.841264
NONHSAT197981.1	1.376486	2.310734	7.7115	0.000382	0.080027	0.828584
NONHSAT222260.1	1.06179	1.672943	7.708241	0.000383	0.080027	0.826558
ENST00000563205.1	1.043303	8.785009	7.699671	0.000385	0.080027	0.821225
NONHSAT149533.1	-1.09248	7.588034	-7.68783	0.000388	0.080027	0.813843
NONHSAT193506.1	1.035082	7.543953	7.676092	0.000391	0.080027	0.806516
lnc-AKR1E2-13:1	1.114973	5.196937	7.660134	0.000395	0.080027	0.79653
NONHSAT206852.1	1.603245	1.938336	7.654144	0.000397	0.080027	0.792776
lnc-COPS2-5:1	-1.14049	9.125598	-7.65245	0.000397	0.080027	0.791716
lnc-FAM149B1-8:1	1.515049	4.294993	7.646757	0.000399	0.080027	0.788141
NONHSAT185214.1	1.45756	1.818813	7.645058	0.000399	0.080027	0.787075
lnc-VPS33B-8:2	-1.4227	2.571047	-7.63583	0.000402	0.080027	0.781279
T297391	2.225389	3.486592	7.633605	0.000402	0.080027	0.779876
lnc-CASP6-4:1	1.43149	1.979969	7.61611	0.000407	0.080027	0.768856
MSTRG.12445.1	-1.02142	4.743196	-7.60962	0.000409	0.080027	0.764759
lnc-SLC25A35-2:1	-1.34417	4.449081	-7.60469	0.00041	0.080027	0.761647
lnc-ICOSLG-6:12	2.270578	2.300671	7.585273	0.000416	0.080027	0.749359
MSTRG.38314.27	-1.16037	4.8542	-7.57891	0.000417	0.080027	0.745327
NONHSAT218324.1	1.70036	1.902271	7.577568	0.000418	0.080027	0.744473
NONHSAT180712.1	-1.31764	6.896325	-7.55822	0.000423	0.080027	0.732176
lnc-GGTLC1-6:1	-1.03502	5.752497	-7.54951	0.000426	0.080027	0.726632
MYOSLID:14	1.35984	5.452351	7.533583	0.00043	0.080027	0.716469
NONHSAT199561.1	1.940828	2.245826	7.527112	0.000432	0.080027	0.712333
lnc-CLDN5-2:1	-1.04138	5.588046	-7.519	0.000434	0.080027	0.707141
NONHSAT207539.1	1.568795	2.000525	7.508142	0.000438	0.080027	0.700185
NONHSAT201940.1	1.400474	1.852704	7.501155	0.00044	0.080027	0.695701
lnc-KLF15-2:5	-1.40368	5.865279	-7.49733	0.000441	0.080027	0.693245
NONHSAT158772.1	-1.13831	1.687556	-7.49455	0.000442	0.080027	0.691457
ENST00000412996.1	-1.64049	3.524146	-7.49084	0.000443	0.080027	0.689073
NONHSAT150153.1	2.56914	2.377697	7.488292	0.000444	0.080027	0.687434
lnc-ZFAND5-2:1	2.049144	2.350952	7.486249	0.000444	0.080027	0.68612
lnc-ATG16L2-10:1	-2.12701	2.23035	-7.47717	0.000447	0.080027	0.680274
NONHSAT204375.1	-1.04001	10.97681	-7.47619	0.000447	0.080027	0.679642
NONHSAT221638.1	-1.22902	10.23009	-7.47483	0.000448	0.080027	0.678767
ENST00000600534.1	1.012114	5.181544	7.46417	0.000451	0.080027	0.671887
ENST00000609475.1	1.043839	8.308984	7.461436	0.000452	0.080027	0.670122
ENST00000671681.1	1.107433	3.398375	7.457203	0.000453	0.080027	0.667386
MSTRG.37754.1	1.039195	4.360253	7.431803	0.000461	0.080027	0.650934
ENST00000649038.1	1.645273	2.649124	7.41308	0.000467	0.080027	0.638766
MSTRG.27998.1	-1.18528	3.652774	-7.40843	0.000468	0.080027	0.635741
MSTRG.20779.1	1.276197	1.728781	7.404439	0.00047	0.080027	0.633138
NONHSAT219631.1	-1.72306	3.26034	-7.40252	0.00047	0.080027	0.631885
NONHSAT160720.1	-1.50963	4.393783	-7.40109	0.000471	0.080027	0.630955
NONHSAT195523.1	2.753521	2.46564	7.39419	0.000473	0.080027	0.626454

NONHSAT210186.1	-1.0364	2.731789	-7.39278	0.000473	0.080027	0.625535
lnc-RNF152-9:1	1.234283	3.759154	7.388281	0.000475	0.080027	0.622594
lnc-MBOAT4-8:1	-1.09168	5.414611	-7.37809	0.000478	0.080027	0.61593
MSTRG.5819.15	1.14595	6.076876	7.377468	0.000478	0.080027	0.615524
lnc-ATG2B-10:2	-1.14748	4.648803	-7.37569	0.000479	0.080027	0.614358
MSTRG.51309.1	-1.06564	10.23774	-7.36091	0.000484	0.080027	0.604672
lnc-DNER-4:1	1.844055	2.142162	7.353098	0.000486	0.080027	0.599544
ENST00000669110.1	2.440668	2.333446	7.344269	0.000489	0.080027	0.59374
NONHSAT192289.1	-1.66501	3.077244	-7.34246	0.00049	0.080027	0.592553
lnc-RASA1-21:1	1.401051	1.877588	7.336617	0.000492	0.080027	0.588703
lnc-TRIM32-8:1	1.373494	1.735084	7.335273	0.000492	0.080027	0.587818
ENST00000616866.1	1.187515	7.564878	7.327211	0.000495	0.080027	0.582504
MSTRG.70386.1	1.544885	2.074902	7.318466	0.000498	0.080027	0.576731
ENST00000565399.1	2.150718	2.28704	7.316735	0.000499	0.080027	0.575588
lnc-CPPED1-6:1	-1.09433	3.588143	-7.31596	0.000499	0.080027	0.575074
NONHSAT171431.1	1.200166	9.062833	7.314592	0.000499	0.080027	0.574172
ENST00000663787.1	-1.95957	2.545549	-7.31358	0.0005	0.080027	0.573501
lnc-HIST1H2AH-5:1	-1.39175	8.271854	-7.31215	0.0005	0.080027	0.572557
lnc-C11orf58-3:1	-1.02614	4.88644	-7.30857	0.000501	0.080027	0.570189
lnc-GJA4-2:1	-1.69833	5.085332	-7.30621	0.000502	0.080027	0.568626
NONHSAT172361.1	1.540962	4.246873	7.300022	0.000504	0.080027	0.564532
NONHSAT197816.1	-1.37181	3.928923	-7.29292	0.000507	0.080027	0.559822
MSTRG.52010.1	-1.26565	4.857154	-7.28194	0.000511	0.080027	0.55254
MSTRG.63624.1	1.054815	1.994129	7.280951	0.000511	0.080027	0.551881
NONHSAT211423.1	1.527579	1.812871	7.2696	0.000515	0.080027	0.544333
NONHSAT214907.1	1.150416	1.706462	7.25736	0.00052	0.080027	0.53618
lnc-WDR77-3:1	2.024139	2.062723	7.240807	0.000526	0.080027	0.525127
NONHSAT176042.1	1.791154	1.973906	7.239829	0.000526	0.080027	0.524474
T138549	2.452123	3.369913	7.237392	0.000527	0.080027	0.522844
NONHSAT185783.1	-1.98393	2.238952	-7.23504	0.000528	0.080027	0.521268
NONHSAT214027.1	1.711476	2.091292	7.202536	0.00054	0.080027	0.499466
MSTRG.30364.1	1.685496	1.888726	7.201141	0.00054	0.080027	0.498528
ENST00000657178.1	1.764962	2.00744	7.191224	0.000544	0.080027	0.491852
NONHSAT182669.1	1.79841	1.953699	7.180267	0.000548	0.080027	0.484464
LINC02246:26	1.250206	5.626534	7.177262	0.000549	0.080027	0.482435
T146351	-1.62237	1.989603	-7.175	0.00055	0.080027	0.480906
NONHSAT193328.1	-1.28508	1.717793	-7.17372	0.000551	0.080027	0.480042
T203331	1.856905	2.045955	7.171398	0.000552	0.080027	0.478474
ENST00000412014.1	-1.07478	1.664284	-7.16107	0.000556	0.080027	0.471486
ENST00000430861.1	-1.95162	2.705179	-7.16095	0.000556	0.080027	0.471409
NONHSAT168355.1	1.774853	2.232362	7.154702	0.000558	0.080027	0.467176
NR_004383	-1.02878	10.05314	-7.15194	0.000559	0.080027	0.465307
NONHSAT193652.1	-1.45268	1.90134	-7.15045	0.00056	0.080027	0.464295
lnc-LRP1B-5:1	-1.13108	4.476373	-7.14751	0.000561	0.080027	0.462298
NONHSAT158257.1	1.320206	1.712939	7.141108	0.000564	0.080027	0.457955
MSTRG.40488.1	1.286404	1.677475	7.139825	0.000564	0.080027	0.457085
lnc-CHD1L-4:1	1.150676	1.634034	7.138701	0.000564	0.080027	0.456321
MSTRG.49052.1	-1.89134	2.532196	-7.13764	0.000565	0.080027	0.455601

NONHSAT193725.1	1.700869	5.693469	7.13535	0.000566	0.080027	0.454044
NONHSAT223754.1	-1.12973	4.908521	-7.12336	0.000571	0.080027	0.445886
NONHSAT204910.1	-1.36653	2.022477	-7.12236	0.000571	0.080027	0.445205
NR_125950	-1.39751	2.795136	-7.12232	0.000571	0.080027	0.445179
MSTRG.11151.1	1.723747	1.943909	7.12208	0.000571	0.080027	0.445017
NONHSAT212907.1	1.250113	1.884781	7.112941	0.000575	0.080027	0.438788
lnc-ZNF672-1:3	-1.68757	3.163836	-7.11112	0.000576	0.080027	0.437546
ENST00000607744.1	1.02635	5.202631	7.108393	0.000577	0.080027	0.435685
NONHSAT199158.1	-1.04361	1.853308	-7.10822	0.000577	0.080027	0.435569
lnc-LTA4H-4:1	-1.00937	9.180161	-7.10655	0.000577	0.080027	0.434426
NONHSAT206835.1	1.538012	4.678498	7.105986	0.000578	0.080027	0.434042
NONHSAT186577.1	-1.74099	1.906379	-7.09613	0.000582	0.080027	0.427311
NONHSAT187095.1	1.941361	2.071413	7.094728	0.000582	0.080027	0.426349
NONHSAT152946.1	-1.05703	3.068009	-7.09347	0.000583	0.080027	0.425486
lnc-DAW1-5:1	-1.60984	4.815884	-7.09232	0.000583	0.080027	0.424705
T225505	-1.75404	2.163787	-7.08423	0.000587	0.080027	0.41916
NONHSAT181855.1	-1.36577	6.41131	-7.07935	0.000589	0.080027	0.415822
lnc-PLGRKT-4:1	1.808182	3.332304	7.069436	0.000593	0.080027	0.409016
lnc-PRR5-2:1	-1.09887	7.574958	-7.06774	0.000593	0.080027	0.407849
ENST00000558948.1	1.27712	4.710735	7.063934	0.000595	0.080027	0.405236
lnc-HIST1H4H-1:1	1.456746	5.065353	7.055279	0.000599	0.080027	0.399284
NONHSAT164765.1	1.37984	2.072143	7.054346	0.000599	0.080027	0.398642
NONHSAT177705.1	-1.16233	1.663342	-7.05032	0.000601	0.080027	0.395871
MSTRG.54672.2	-1.49787	1.826069	-7.04726	0.000602	0.080027	0.393761
ENST00000569677.1	1.531836	1.870471	7.047244	0.000602	0.080027	0.393751
ENST00000656319.1	1.100605	5.856367	7.040603	0.000605	0.080027	0.389173
lnc-AFDN-8:1	-1.50765	2.0717	-7.036	0.000607	0.080027	0.385994
lnc-TMA16-4:2	1.641419	3.909983	7.035044	0.000607	0.080027	0.385337
lnc-NFYB-1:5	1.125743	8.895134	7.023392	0.000613	0.080027	0.377285
MSTRG.71794.1	-1.89091	2.978468	-7.01997	0.000614	0.080027	0.374921
ENST00000670022.1	1.550095	2.151464	7.012602	0.000617	0.080027	0.369816
MSTRG.28398.1	1.408041	1.930106	7.007906	0.000619	0.080027	0.366561
lnc-B3GAT2-7:1	-1.27882	3.452253	-7.0011	0.000622	0.080027	0.361842
lnc-PIGP-1:1	1.214363	2.072759	6.991764	0.000627	0.080027	0.355355
NONHSAT188014.1	-1.11349	4.865259	-6.98836	0.000628	0.080027	0.352987
lnc-SYT13-8:1	1.245053	6.315713	6.981381	0.000631	0.080027	0.348133
lnc-RAP1B-5:3	-1.50279	1.887397	-6.98094	0.000631	0.080027	0.347823
NONHSAT192727.1	1.847353	1.974323	6.975594	0.000634	0.080027	0.344101
MSTRG.17975.21	2.193643	3.369567	6.972005	0.000635	0.080027	0.3416
NONHSAT174480.1	1.278195	3.863197	6.956875	0.000642	0.080027	0.331038
lnc-VASH1-1:1	-1.07436	9.329316	-6.95269	0.000644	0.080027	0.328113
ENST00000670306.1	1.423086	4.287747	6.950008	0.000646	0.080027	0.326236
lnc-SPANXN1-1:1	-1.15286	10.507	-6.94607	0.000647	0.080027	0.323481
lnc-SNTB1-3:1	-1.22136	6.060137	-6.94595	0.000647	0.080027	0.323394
lnc-M1AP-3:1	-1.55644	3.038437	-6.94506	0.000648	0.080027	0.322774
NONHSAT197128.1	-2.04733	2.660599	-6.93415	0.000653	0.080027	0.315127
ENST00000432413.2	1.473655	4.669327	6.93355	0.000653	0.080027	0.314706
NONHSAT185399.1	1.104358	3.70922	6.932748	0.000654	0.080027	0.314144

lnc-TRMT1L-3:1	-1.0343	5.485945	-6.92397	0.000658	0.080027	0.307983
lnc-RNF144B-5:1	-1.33801	3.916517	-6.92207	0.000659	0.080027	0.306649
NONHSAT201163.1	1.073749	3.852484	6.915376	0.000662	0.080027	0.301939
ENST00000605836.1	1.013049	5.936759	6.914817	0.000662	0.080027	0.301546
T219111	1.219527	4.429279	6.908807	0.000665	0.080027	0.297316
NONHSAT203830.1	1.384918	1.792361	6.901109	0.000669	0.080027	0.291892
NR_037839	1.300619	1.722274	6.891448	0.000673	0.080027	0.285075
NONHSAT168109.1	-1.30316	2.005192	-6.88545	0.000676	0.080027	0.280836
lnc-BATF-1:1	-1.42262	6.627265	-6.88267	0.000678	0.080027	0.278869
NONHSAT167728.1	1.965941	2.09258	6.881158	0.000678	0.080027	0.277803
NONHSAT210837.1	1.894453	2.035625	6.88057	0.000679	0.080027	0.277387
NONHSAT190516.1	1.590715	1.851097	6.880358	0.000679	0.080027	0.277237
NONHSAT158087.1	1.078709	1.758221	6.873984	0.000682	0.080027	0.272726
ENST00000491932.1	1.202806	4.721769	6.871271	0.000683	0.080027	0.270804
NONHSAT150157.1	1.203847	6.363695	6.869774	0.000684	0.080027	0.269744
lnc-FGF18-4:2	1.136339	1.684108	6.854613	0.000692	0.080027	0.258989
NONHSAT164439.1	1.222655	2.426078	6.854478	0.000692	0.080027	0.258893
lnc-CA8-9:1	-1.15062	3.659527	-6.854	0.000692	0.080027	0.258554
NONHSAT191877.1	-1.2204	4.49979	-6.84111	0.000699	0.080027	0.249386
lnc-NDUFAF4-2:1	-1.32891	2.784643	-6.83297	0.000703	0.080027	0.243591
lnc-SPNS3-1:5	1.135987	6.08002	6.827082	0.000706	0.080027	0.239393
lnc-MANEA-9:1	-1.14162	8.933094	-6.82683	0.000706	0.080027	0.239209
lnc-C13orf46-4:2	1.009153	6.227009	6.825977	0.000706	0.080027	0.238604
lnc-ME2-2:1	-2.11809	4.274908	-6.8196	0.00071	0.080027	0.234055
NONHSAT196570.1	1.776386	2.262775	6.803373	0.000718	0.080027	0.222448
MSTRG.7116.1	-1.03478	5.513292	-6.80141	0.000719	0.080027	0.221044
ENST00000653275.1	-1.75521	2.164816	-6.8003	0.00072	0.080027	0.220249
lnc-SEMA6A-1:2	1.685919	1.922791	6.79999	0.00072	0.080027	0.220025
lnc-DCP1B-9:2	-1.17295	2.162221	-6.79811	0.000721	0.080027	0.218682
NONHSAT186205.1	1.391847	2.291462	6.792936	0.000724	0.080027	0.214969
NONHSAT176959.1	-1.52066	3.917489	-6.7832	0.000729	0.080027	0.207984
NONHSAT203888.1	-1.19481	9.230616	-6.78303	0.000729	0.080027	0.207862
MSTRG.53241.1	1.241869	6.365451	6.776907	0.000732	0.080027	0.203459
ENST00000417385.2	-1.00743	8.049647	-6.7729	0.000734	0.080027	0.200579
lnc-BNIP1-5:4	1.136038	3.552105	6.763624	0.000739	0.080027	0.193898
NONHSAT196594.1	-1.97552	3.043373	-6.76083	0.000741	0.080027	0.191883
MSTRG.66149.1	-1.30203	1.950046	-6.75705	0.000743	0.080027	0.189158
lnc-NENF-5:1	-1.83455	2.811025	-6.75646	0.000743	0.080027	0.188731
lnc-ANO2-3:4	1.376913	6.092212	6.751744	0.000746	0.080027	0.18533
lnc-TSPYL6-5:1	-1.11855	5.744385	-6.75026	0.000747	0.080027	0.184257
ENST00000443284.1	-1.01202	3.260997	-6.74826	0.000748	0.080027	0.182817
lnc-FAM20B-2:1	1.523895	5.850438	6.744066	0.00075	0.080027	0.179784
T017018	2.133698	2.714689	6.742351	0.000751	0.080027	0.178545
lnc-BCAT2-1:1	-1.19711	9.503006	-6.73956	0.000753	0.080027	0.176524
NONHSAT215224.1	1.798946	2.003905	6.720518	0.000763	0.080027	0.162733
ENST00000513871.1	1.969117	2.166249	6.716849	0.000765	0.080027	0.160071
NONHSAT215185.1	-1.15778	3.393773	-6.71324	0.000767	0.080027	0.157448
lnc-JMJD1C-7:1	-1.34898	3.253669	-6.71204	0.000768	0.080027	0.156579

NONHSAT181733.1	-1.82488	2.016595	-6.7111	0.000769	0.080027	0.155894
MSTRG.72742.1	1.24134	1.697806	6.710984	0.000769	0.080027	0.155812
lnc-RASGRP3-5:1	1.412737	1.923288	6.701791	0.000774	0.080027	0.149128
NONHSAT215383.1	1.199706	6.185458	6.698628	0.000776	0.080027	0.146826
NONHSAT175806.1	2.162926	2.278206	6.697055	0.000777	0.080027	0.14568
NONHSAT188381.1	-1.55933	2.111304	-6.69106	0.00078	0.080027	0.14131
lnc-OTOR-3:2	-1.69185	3.880948	-6.69014	0.000781	0.080027	0.140641
NONHSAT199170.1	1.233236	3.800076	6.687992	0.000782	0.080027	0.139077
NONHSAT178915.1	1.367766	2.597984	6.686952	0.000782	0.080027	0.138318
lnc-SPATA31A1-5:6	-1.98481	2.454631	-6.68669	0.000783	0.080027	0.138129
lnc-TEKT4-2:1	-1.45203	8.027886	-6.68556	0.000783	0.080027	0.137305
T315079	-1.2447	8.639162	-6.68194	0.000785	0.080027	0.134664
lnc-DHX38-29:3	-1.95574	2.131124	-6.6811	0.000786	0.080027	0.134048
NONHSAT159921.1	2.383564	2.600832	6.680227	0.000786	0.080027	0.13341
ENST00000413286.1	-1.64712	1.988946	-6.67933	0.000787	0.080027	0.132757
MSTRG.30517.2	1.816027	3.615446	6.677899	0.000788	0.080027	0.131711
lnc-TRIM2-1:1	-1.10159	5.06617	-6.67302	0.000791	0.080027	0.128145
lnc-LY96-2:6	2.214756	2.171582	6.665299	0.000795	0.080027	0.122499
ENST00000611946.1	1.498077	3.80501	6.663875	0.000796	0.080027	0.121457
ENST00000446301.1	1.075135	6.799386	6.656431	0.0008	0.080027	0.116005
NONHSAT175662.1	1.39017	1.813611	6.653695	0.000802	0.080027	0.113999
NONHSAT158019.1	-1.6207	7.363321	-6.653	0.000802	0.080027	0.113489
MSTRG.8669.21	-1.43992	3.619293	-6.64488	0.000807	0.080027	0.107531
ENST00000507950.1	-1.18559	1.723842	-6.6422	0.000809	0.080027	0.105565
NONHSAT178179.1	1.168511	1.892345	6.632936	0.000815	0.080027	0.098754
lnc-AMZ1-1:1	1.523045	3.08592	6.627691	0.000818	0.080027	0.094894
ENST00000518598.1	1.362803	3.501837	6.623891	0.00082	0.080027	0.092096
NONHSAT169398.1	1.493152	1.846915	6.613825	0.000826	0.080027	0.084675
NONHSAT179771.1	1.149748	3.604831	6.611638	0.000828	0.080027	0.083061
lnc-CDK20-13:1	-1.09885	3.187746	-6.60863	0.00083	0.080027	0.080841
lnc-PPIL2-7:2	1.97211	2.640655	6.601912	0.000834	0.080027	0.075877
NONHSAT211913.1	1.702132	2.018191	6.598985	0.000836	0.080027	0.073713
NONHSAT163946.1	1.333212	5.768017	6.597128	0.000837	0.080027	0.07234
MSTRG.7117.1	-1.79092	2.415495	-6.59585	0.000837	0.080027	0.071397
lnc-RAP1B-6:1	-1.52974	7.592472	-6.59078	0.000841	0.080027	0.067644
NONHSAT156094.1	1.82529	2.122478	6.583575	0.000845	0.080027	0.062302
NONHSAT175498.1	1.013674	5.089804	6.578828	0.000848	0.080027	0.058782
lnc-LEPROTL1-13:4	1.63477	2.516886	6.576147	0.00085	0.080027	0.056793
lnc-GALNT12-1:1	-1.11462	7.785592	-6.57558	0.00085	0.080027	0.056368
MSTRG.58791.1	-1.20485	8.016013	-6.57136	0.000853	0.080027	0.053238
lnc-USP16-11:1	-1.79896	2.177762	-6.56789	0.000855	0.080027	0.050657
lnc-ANXA6-5:1	1.213862	5.78913	6.566312	0.000856	0.080027	0.049487
lnc-UHRF1BP1-2:1	-1.35837	9.983194	-6.56162	0.000859	0.080027	0.045997
NONHSAT180060.1	1.003554	8.090199	6.559811	0.000861	0.080027	0.044651
ENST00000454928.5	1.283032	5.620791	6.558722	0.000861	0.080027	0.043841
lnc-C19orf57-8:1	1.151021	6.248333	6.558065	0.000862	0.080027	0.043352
NONHSAT191743.1	1.997448	3.565959	6.538972	0.000874	0.080027	0.029118
lnc-CAV1-1:1	1.085756	3.603998	6.537573	0.000875	0.080027	0.028073

ENST00000456103.1	-2.14303	2.576584	-6.53513	0.000877	0.080027	0.02625
T194191	1.374917	2.113044	6.526957	0.000882	0.080027	0.020138
lnc-DEPDC1-1:1	-1.30564	3.233201	-6.5206	0.000886	0.080027	0.015378
ENST00000668625.1	-1.05517	1.757932	-6.51904	0.000887	0.080027	0.014211
ENST00000521660.1	-1.883	2.8561	-6.51468	0.00089	0.080027	0.010945
NONHSAT203193.1	1.056316	1.610153	6.511524	0.000893	0.080027	0.008579
NONHSAT156302.1	1.227015	2.738766	6.502171	0.000899	0.080027	0.00156
ENST00000565600.1	-1.0692	1.878041	-6.50215	0.000899	0.080027	0.001541
lnc-KDM5C-5:1	-1.01911	10.77778	-6.50059	0.0009	0.080027	0.000372
lnc-GRIK3-2:1	1.961477	2.455527	6.498946	0.000901	0.080027	-0.00086
lnc-ZNF680-15:1	1.549354	2.015284	6.495178	0.000904	0.080027	-0.0037
NONHSAT185478.1	-1.9027	2.441174	-6.49404	0.000904	0.080027	-0.00455
NONHSAT161124.1	-1.61374	2.69456	-6.49134	0.000906	0.080027	-0.00658
lnc-POLE4-8:3	2.188005	4.130314	6.488	0.000909	0.080027	-0.00909
T356625	1.320406	1.995661	6.487708	0.000909	0.080027	-0.00931
lnc-EGLN1-1:10	1.036023	7.253497	6.483542	0.000912	0.080027	-0.01245
ENST00000568729.1	-1.08451	9.739151	-6.47697	0.000916	0.080027	-0.01741
ENST00000665401.1	-1.25254	3.790249	-6.47682	0.000916	0.080027	-0.01752
NONHSAT211796.1	1.013208	5.432623	6.47346	0.000919	0.080027	-0.02005
NONHSAT161257.1	-1.75678	3.018586	-6.47242	0.000919	0.080027	-0.02084
lnc-RNF44-1:1	-1.15366	10.22896	-6.47083	0.000921	0.080027	-0.02203
lnc-AGPAT2-1:13	1.381268	5.080224	6.466081	0.000924	0.080027	-0.02562
ENST00000566957.1	1.522687	2.172298	6.465624	0.000924	0.080027	-0.02597
MSTRG.23181.1	1.440059	1.944644	6.465186	0.000925	0.080027	-0.0263
MSTRG.53475.5	-2.33966	2.56832	-6.46248	0.000926	0.080027	-0.02834
ENST00000649460.1	1.399459	1.855395	6.462048	0.000927	0.080027	-0.02867
NONHSAT196346.1	1.622067	2.010872	6.457688	0.00093	0.080027	-0.03197
NONHSAT214249.1	-1.11439	4.082097	-6.45683	0.00093	0.080027	-0.03261
lnc-MRPS14-4:1	-1.01248	4.07195	-6.45434	0.000932	0.080027	-0.0345
lnc-FOXO4-5:1	-1.04693	5.445051	-6.45352	0.000933	0.080027	-0.03512
lnc-JUN-2:8	1.107324	5.622691	6.451976	0.000934	0.080027	-0.03629
lnc-POU3F3-13:1	1.397397	1.925329	6.45188	0.000934	0.080027	-0.03636
lnc-RASGRF1-3:1	1.600539	4.322294	6.451151	0.000935	0.080027	-0.03691
lnc-ENDOD1-3:1	-1.85729	2.744466	-6.4488	0.000936	0.080027	-0.03869
lnc-PTGS2-3:1	-1.61922	2.90338	-6.44185	0.000941	0.080085	-0.04396
MSTRG.46712.1	-1.03377	3.551295	-6.43933	0.000943	0.080105	-0.04587
NONHSAT169970.1	-1.74889	2.796011	-6.4354	0.000946	0.080105	-0.04885
ENST00000655948.1	-1.68049	2.06507	-6.4315	0.000949	0.080105	-0.05181
NONHSAT187789.1	-1.50618	2.125271	-6.42701	0.000952	0.080105	-0.05523
lnc-KBTBD12-1:1	-1.27797	2.392583	-6.42694	0.000952	0.080105	-0.05528
lnc-PPP1R3B-3:1	-1.08314	1.798044	-6.42549	0.000953	0.080105	-0.05638
lnc-CIAPIN1-2:1	-1.99489	2.364052	-6.41632	0.00096	0.080312	-0.06336
NONHSAT214718.1	-1.62942	2.467703	-6.40285	0.00097	0.080455	-0.07363
NONHSAT209098.1	-1.44399	1.993352	-6.39357	0.000977	0.080455	-0.08071
lnc-C8orf86-3:1	-1.39752	2.025181	-6.38761	0.000981	0.080455	-0.08527
NONHSAT202334.1	1.665086	2.202561	6.386845	0.000982	0.080455	-0.08585
lnc-MPPE1-14:1	-1.20307	5.335332	-6.38679	0.000982	0.080455	-0.08589
NONHSAT221042.1	1.104405	3.423859	6.384727	0.000983	0.080455	-0.08747

ENST00000419613.1	-1.40811	4.376354	-6.38064	0.000986	0.080455	-0.0906
NONHSAT167388.1	-1.38819	6.354657	-6.38027	0.000987	0.080455	-0.09088
lnc-AKT1-3:1	-1.32721	8.622397	-6.37831	0.000988	0.080455	-0.09238
NR_001560	-1.09724	4.278544	-6.37683	0.000989	0.080455	-0.09352
NONHSAT213024.1	-1.50088	2.30272	-6.37681	0.000989	0.080455	-0.09354
NONHSAT201749.1	1.403677	1.815377	6.369204	0.000995	0.080455	-0.09937
lnc-DRD1-3:1	-1.93185	7.360945	-6.36611	0.000998	0.080455	-0.10174
NONHSAT160171.1	-1.39972	1.951583	-6.36313	0.001	0.080455	-0.10403
lnc-AHCTF1-4:1	-1.6956	2.847281	-6.35987	0.001002	0.080455	-0.10653
lnc-CEP128-2:1	2.619351	3.191546	6.359793	0.001003	0.080455	-0.10659
NONHSAT185502.1	2.442669	2.845071	6.356344	0.001005	0.080455	-0.10924
lnc-NTMT1-7:2	1.53622	1.89976	6.355156	0.001006	0.080455	-0.11016
MSTRG.25374.4	-1.74965	2.452854	-6.35226	0.001008	0.080455	-0.11238
NONHSAT210501.1	1.887356	2.090285	6.339898	0.001018	0.080455	-0.1219
NONHSAT207648.1	-1.43081	2.627142	-6.33858	0.001019	0.080455	-0.12291
NONHSAT205640.1	-1.69748	2.171848	-6.32978	0.001026	0.080455	-0.12971
NONHSAT162565.1	-1.75755	2.561029	-6.32561	0.001029	0.080455	-0.13292
ENST00000435108.1	1.841366	7.254913	6.322231	0.001032	0.080455	-0.13554
lnc-PCDH19-7:1	1.491296	1.884256	6.320415	0.001034	0.080455	-0.13694
T152001	1.250588	1.687711	6.319571	0.001034	0.080455	-0.13759
lnc-ZNF717-5:1	-1.15757	5.276948	-6.31927	0.001034	0.080455	-0.13782
lnc-C15orf41-2:4	1.266424	1.918799	6.317521	0.001036	0.080455	-0.13918
lnc-SAP30-1:2	1.44576	2.065335	6.3144	0.001038	0.080455	-0.14159
T117800	1.115036	5.186055	6.313212	0.001039	0.080455	-0.14251
lnc-PTPN23-2:1	1.108759	1.598791	6.312713	0.00104	0.080455	-0.1429
MSTRG.42199.1	-1.25722	2.945135	-6.30914	0.001043	0.080455	-0.14566
lnc-DPYS-1:2	1.007674	3.280977	6.307664	0.001044	0.080455	-0.14681
lnc-MAEA-1:2	-1.65502	2.735702	-6.29032	0.001058	0.080455	-0.16026
NONHSAT166171.1	-1.43766	1.853384	-6.28822	0.00106	0.080455	-0.16189
lnc-ACE-5:1	-1.38528	3.902395	-6.28804	0.00106	0.080455	-0.16204
NONHSAT192124.1	-1.35283	4.04956	-6.28757	0.00106	0.080455	-0.1624
lnc-TIMM21-7:1	-1.01318	8.312757	-6.28617	0.001061	0.080475	-0.16349
NONHSAT149069.1	-1.80128	2.883328	-6.27872	0.001068	0.080651	-0.16928
ENST00000669729.1	-1.07931	7.189595	-6.27803	0.001068	0.080651	-0.16982
lnc-MPDZ-4:1	-1.57292	5.768241	-6.27537	0.00107	0.080651	-0.17189
lnc-ZNF316-3:11	2.010528	2.860933	6.274978	0.001071	0.080651	-0.1722
NONHSAT160022.1	1.288413	8.865674	6.273726	0.001072	0.080651	-0.17317
lnc-RDH13-2:9	1.929546	2.16938	6.268587	0.001076	0.080651	-0.17718
ENST00000606924.1	1.610132	4.390633	6.268464	0.001076	0.080651	-0.17727
NR_033379	-1.23782	4.488561	-6.26223	0.001082	0.080651	-0.18213
lnc-ALG10B-3:2	1.945326	2.689126	6.260088	0.001083	0.080651	-0.18381
lnc-ERP44-4:1	-1.04823	5.715719	-6.25905	0.001084	0.080651	-0.18462
lnc-KCNE1B-6:1	-1.41527	2.242946	-6.2585	0.001085	0.080651	-0.18505
NONHSAT208391.1	-1.76097	1.993114	-6.25613	0.001087	0.080651	-0.1869
lnc-MAST2-5:1	-1.31456	6.248346	-6.25484	0.001088	0.080651	-0.18791
ENST00000655870.1	1.235237	2.222092	6.242246	0.001099	0.080771	-0.19775
lnc-EDEM2-2:2	1.580681	4.958621	6.240085	0.0011	0.080771	-0.19945
NONHSAT210240.1	1.076444	1.648142	6.239822	0.001101	0.080771	-0.19965

ENST00000661935.1	1.345799	5.6575	6.235953	0.001104	0.080771	-0.20268
NR_047550	-1.13251	5.703739	-6.2355	0.001104	0.080771	-0.20304
MSTRG.33672.1	-1.45372	2.165657	-6.22987	0.001109	0.080771	-0.20745
lnc-HIF1AN-5:1	1.901536	2.051573	6.227719	0.001111	0.080771	-0.20914
lnc-GLE1-1:3	1.411459	3.877114	6.227611	0.001111	0.080771	-0.20922
lnc-CLSTN1-7:2	-1.97028	5.762558	-6.22614	0.001113	0.080771	-0.21038
T265427	1.399649	2.00344	6.226129	0.001113	0.080771	-0.21039
NONHSAT218418.1	2.086574	2.344904	6.224795	0.001114	0.080771	-0.21143
NONHSAT204179.1	-1.12946	2.946592	-6.22369	0.001115	0.080771	-0.2123
lnc-LY6H-4:1	-1.27541	8.426837	-6.21643	0.001121	0.080771	-0.218
ENST00000668852.1	-1.69058	2.571143	-6.21642	0.001121	0.080771	-0.21801
ENST00000572811.1	1.50641	2.050164	6.216281	0.001121	0.080771	-0.21812
lnc-ENOPH1-1:1	1.199258	3.777143	6.215863	0.001122	0.080771	-0.21845
NONHSAT220197.1	-1.04934	7.078574	-6.21557	0.001122	0.080771	-0.21868
lnc-CD8B-3:2	1.065808	6.091931	6.210962	0.001126	0.080771	-0.22231
T283285	1.715401	4.069822	6.210393	0.001126	0.080771	-0.22275
NONHSAT218613.1	-1.66237	2.110417	-6.20912	0.001128	0.080771	-0.22376
lnc-OR10K1-1:1	-1.35216	4.744986	-6.2087	0.001128	0.080771	-0.22409
NONHSAT214398.1	-1.90744	3.53674	-6.20621	0.00113	0.080771	-0.22604
MSTRG.28178.1	1.179181	3.473602	6.201232	0.001135	0.080795	-0.22997
lnc-FAM25C-7:1	1.679496	1.902708	6.194491	0.001141	0.081103	-0.23528
lnc-ATXN7-15:1	2.263069	2.60419	6.185294	0.001149	0.08117	-0.24254
LINC01384:1	-1.45045	4.122104	-6.18452	0.00115	0.08117	-0.24315
ENST00000560339.2	-1.20203	1.893495	-6.17908	0.001155	0.081178	-0.24745
lnc-IL2RA-2:1	1.343632	4.971189	6.17836	0.001155	0.081178	-0.24802
NONHSAT217928.1	-1.232	10.55106	-6.17576	0.001158	0.081266	-0.25008
lnc-NDUFB4-3:1	-1.24561	2.714371	-6.17278	0.00116	0.081266	-0.25244
lnc-RASEF-4:1	-1.23623	2.114702	-6.16533	0.001167	0.081266	-0.25834
NR_027097	-1.04496	4.127058	-6.16287	0.00117	0.081266	-0.26029
ENST00000558375.1	-1.36237	2.034779	-6.15846	0.001174	0.081266	-0.26379
NONHSAT162834.1	1.677907	1.92332	6.153073	0.001179	0.081384	-0.26806
MSTRG.12842.1	-1.24636	2.035174	-6.14886	0.001183	0.081508	-0.27141
MSTRG.59583.3	1.630488	1.88861	6.142055	0.001189	0.081688	-0.27682
NONHSAT199678.1	-1.34297	6.283403	-6.14179	0.001189	0.081688	-0.27703
MSTRG.54671.1	1.534739	1.853117	6.13453	0.001196	0.082049	-0.28281
T081667	1.996549	2.013026	6.130078	0.0012	0.082093	-0.28636
NONHSAT204732.1	-1.21699	1.822819	-6.11919	0.001211	0.082163	-0.29504
lnc-CCM2-4:1	-1.53442	7.89677	-6.10986	0.00122	0.082345	-0.3025
ENST00000541885.1	2.399151	2.944714	6.099385	0.00123	0.082513	-0.31088
ENST00000569242.1	-1.48161	7.20146	-6.09769	0.001232	0.082513	-0.31224
lnc-XRCC4-3:1	1.183344	3.303216	6.093109	0.001236	0.082513	-0.31591
NONHSAT150603.1	-1.88868	4.657205	-6.09232	0.001237	0.082513	-0.31654
NONHSAT168114.1	-1.09853	3.183222	-6.09144	0.001238	0.082513	-0.31725
lnc-AZIN1-6:1	1.359784	2.337557	6.088969	0.00124	0.082513	-0.31923
lnc-GLP1R-1:1	1.139019	4.798514	6.08884	0.001241	0.082513	-0.31934
NONHSAT194307.1	1.562748	2.016885	6.07783	0.001252	0.082888	-0.32818
lnc-CWH43-4:1	-1.21363	16.05626	-6.07454	0.001255	0.082888	-0.33082
ENST00000584361.1	-1.106	4.306297	-6.07119	0.001258	0.082888	-0.33352

NONHSAT216070.1	-1.00298	6.935518	-6.07043	0.001259	0.082888	-0.33413
ENST00000609268.2	1.137601	2.099009	6.067856	0.001262	0.082888	-0.3362
lnc-TMEM130-1:3	-1.0627	7.038329	-6.05779	0.001272	0.083122	-0.34431
NONHSAT180563.1	1.021206	8.674477	6.054011	0.001276	0.083191	-0.34736
NONHSAT197059.1	1.571894	1.879539	6.053382	0.001276	0.083191	-0.34787
lnc-NMBR-4:1	-1.00515	3.400737	-6.04233	0.001288	0.083502	-0.35679
NONHSAT204602.1	1.620077	1.870931	6.038475	0.001292	0.083708	-0.35991
LINC00324:3	1.185533	10.49104	6.035892	0.001294	0.083774	-0.362
lnc-PNMA2-2:8	1.369698	7.354418	6.031143	0.001299	0.08388	-0.36584
NR_038888	-1.75033	3.819279	-6.02902	0.001302	0.083929	-0.36757
MSTRG.64181.1	-1.08026	9.338793	-6.0276	0.001303	0.083929	-0.36871
NONHSAT182249.1	1.015816	1.680461	6.024324	0.001307	0.083929	-0.37137
ENST00000625026.1	-1.673	2.618559	-6.02365	0.001307	0.083929	-0.37192
lnc-ATAD1-5:4	1.729364	2.292265	6.023179	0.001308	0.083929	-0.3723
NONHSAT198196.1	-1.09604	7.228192	-6.02216	0.001309	0.083929	-0.37313
NONHSAT195908.1	-1.86141	2.365651	-6.02193	0.001309	0.083929	-0.37331
ENST00000445039.2	-1.01745	3.760142	-6.02068	0.00131	0.083946	-0.37432
MSTRG.55873.1	1.461456	1.961867	6.018405	0.001313	0.084007	-0.37617
NONHSAT169902.1	1.80339	2.269367	6.011837	0.00132	0.084112	-0.3815
NONHSAT214664.1	-1.08325	6.322935	-6.00987	0.001322	0.084112	-0.3831
lnc-ZC3H12D-2:2	1.629105	1.930167	6.009338	0.001322	0.084112	-0.38353
NONHSAT178438.1	2.564374	2.46913	6.008767	0.001323	0.084112	-0.384
lnc-SESN1-6:2	1.72719	2.587996	6.002674	0.00133	0.084301	-0.38895
NONHSAT162586.1	-1.54505	2.115051	-6.00071	0.001332	0.084347	-0.39055
ENST00000649594.1	2.502184	2.353275	5.999113	0.001333	0.084403	-0.39185
lnc-CAPN14-2:1	-1.35498	1.801905	-5.99566	0.001337	0.084461	-0.39466
NONHSAT166899.1	1.484029	1.983003	5.980861	0.001353	0.084935	-0.40673
NONHSAT173282.1	1.195456	1.73963	5.972542	0.001362	0.084966	-0.41353
NONHSAT181891.1	1.210479	2.861823	5.972483	0.001363	0.084966	-0.41357
MSTRG.41610.78	1.09568	1.599579	5.967765	0.001368	0.085186	-0.41743
NONHSAT182872.1	-1.77319	2.01515	-5.96527	0.001371	0.085204	-0.41947
NONHSAT201694.1	2.312414	2.473996	5.964474	0.001371	0.085204	-0.42013
NONHSAT154599.1	1.099803	3.141728	5.95684	0.00138	0.085438	-0.42638
NONHSAT153965.1	1.032249	1.768401	5.954234	0.001383	0.085442	-0.42851
LINC02271:7	-1.0076	3.848079	-5.9518	0.001386	0.085457	-0.43051
NONHSAT207754.1	1.090595	1.576083	5.948718	0.001389	0.085506	-0.43304
lnc-EFHB-3:1	-1.18903	8.308393	-5.94766	0.00139	0.085506	-0.43391
lnc-CEP95-4:1	1.274484	4.786982	5.947046	0.001391	0.085506	-0.43441
lnc-FSHB-8:1	-1.10503	7.120293	-5.94576	0.001393	0.085506	-0.43546
lnc-SLFN14-1:1	-2.39848	4.171954	-5.94403	0.001394	0.085527	-0.43689
NONHSAT159699.1	1.556838	1.90994	5.942819	0.001396	0.085559	-0.43788
MSTRG.40285.1	1.392292	1.859237	5.936533	0.001403	0.085668	-0.44305
MSTRG.55846.1	-1.63596	2.26679	-5.93322	0.001407	0.085668	-0.44577
lnc-RTN4R-1:5	1.481694	1.860472	5.929018	0.001412	0.08573	-0.44923
NONHSAT156709.1	1.454524	2.269511	5.927517	0.001413	0.08573	-0.45047
lnc-SLC47A1-4:1	-1.36613	9.34374	-5.92704	0.001414	0.08573	-0.45086
lnc-TP53TG3F-17:1	2.829342	2.430639	5.920834	0.001421	0.085991	-0.45597
ENST00000658524.1	1.002302	1.543473	5.920381	0.001422	0.085991	-0.45635

NONHSAT177001.1	1.584216	3.428124	5.91722	0.001425	0.086103	-0.45895
lnc-FEV-4:1	-1.47986	5.782893	-5.91172	0.001432	0.08621	-0.46349
NONHSAT150369.1	-1.50992	2.158107	-5.91069	0.001433	0.08621	-0.46434
lnc-PAIP2-4:1	-1.91938	2.57114	-5.90375	0.001441	0.086407	-0.47007
lnc-TBL1Y-1:1	2.81313	4.47821	5.902785	0.001442	0.086407	-0.47087
lnc-SLC30A5-5:1	-1.39989	5.366735	-5.90209	0.001443	0.086407	-0.47145
LINC01058:4	-1.7966	2.785189	-5.8977	0.001448	0.08641	-0.47508
MSTRG.1334.12	-1.48326	2.132159	-5.88443	0.001464	0.086891	-0.48607
NONHSAT167776.1	-1.02622	4.050191	-5.88341	0.001465	0.086891	-0.48692
lnc-CENPP-12:1	-1.13779	10.59182	-5.88303	0.001466	0.086891	-0.48723
lnc-HMGB2-8:1	-1.45871	2.54421	-5.88274	0.001466	0.086891	-0.48747
ENST00000517623.1	1.320853	2.075791	5.882296	0.001467	0.086891	-0.48784
NONHSAT168303.1	1.138288	1.656218	5.882195	0.001467	0.086891	-0.48792
MSTRG.39584.1	-1.32102	1.983604	-5.88144	0.001468	0.086891	-0.48855
MSTRG.58001.1	-1.06903	3.586593	-5.87805	0.001472	0.086891	-0.49137
lnc-PAX5-7:1	-1.77712	4.122163	-5.87596	0.001474	0.086954	-0.4931
lnc-ACTRT1-3:1	-1.45523	3.680788	-5.87226	0.001479	0.086989	-0.49617
MSTRG.66186.1	-1.05911	3.823786	-5.87122	0.00148	0.086989	-0.49703
NONHSAT157663.1	1.927851	1.990171	5.870986	0.00148	0.086989	-0.49723
lnc-RGS18-11:1	-1.4853	6.32027	-5.86746	0.001485	0.08714	-0.50016
ENST00000566876.1	1.386965	1.754391	5.858608	0.001496	0.087474	-0.50753
MSTRG.9065.1	1.879375	2.32191	5.857524	0.001497	0.087474	-0.50843
NONHSAT165812.1	-1.0631	3.579662	-5.85648	0.001498	0.087474	-0.50929
NONHSAT164702.1	1.434036	4.339561	5.854569	0.001501	0.087561	-0.51089
ENST00000605480.1	-1.52505	2.728367	-5.847	0.00151	0.087569	-0.5172
T307868	1.310217	5.027017	5.845454	0.001512	0.087569	-0.51849
ENST00000414816.1	-1.23339	3.854776	-5.84445	0.001513	0.087569	-0.51933
lnc-INTS6-4:1	-1.21107	11.47419	-5.84223	0.001516	0.087569	-0.52118
ENST00000579033.1	1.081814	1.618215	5.837404	0.001522	0.087569	-0.52521
lnc-KCNS3-1:1	1.149556	6.1857	5.837337	0.001522	0.087569	-0.52527
lnc-ARHGAP12-2:2	1.251033	1.656918	5.836103	0.001524	0.087569	-0.5263
NONHSAT200046.1	-1.09633	3.530652	-5.835	0.001525	0.087569	-0.52722
T325176	-1.56049	2.35117	-5.83172	0.001529	0.087748	-0.52996
T160707	2.870454	2.478598	5.831146	0.00153	0.087748	-0.53044
lnc-MRPS18A-4:1	-1.47968	3.34976	-5.82398	0.001539	0.08797	-0.53644
MSTRG.22399.2	-1.09581	4.053402	-5.82172	0.001542	0.088067	-0.53833
NONHSAT177949.1	1.723509	1.943574	5.819234	0.001545	0.088067	-0.54041
MSTRG.23214.5	2.061931	2.513435	5.808622	0.001559	0.088461	-0.54931
lnc-COPS4-1:1	1.031275	6.160111	5.807561	0.00156	0.088461	-0.5502
ENST00000329015.2	-1.00373	2.906489	-5.80721	0.001561	0.088461	-0.5505
lnc-C1QTNF9B-3:1	1.818734	2.622112	5.805363	0.001563	0.088461	-0.55205
lnc-PCDH9-12:1	-1.64818	3.868501	-5.80328	0.001566	0.088461	-0.5538
lnc-AMN1-2:6	-1.28924	2.272158	-5.80238	0.001567	0.088461	-0.55455
lnc-ATP6V1D-8:4	-1.44191	1.994188	-5.80062	0.001569	0.088514	-0.55604
ENST00000667440.1	1.637457	4.281873	5.800247	0.00157	0.088514	-0.55635
NONHSAT224251.1	-1.84696	2.656736	-5.79653	0.001574	0.08852	-0.55948
NONHSAT160952.1	-1.10431	2.668456	-5.79363	0.001578	0.08852	-0.56191
lnc-RCL1-2:1	1.352678	1.768875	5.792156	0.00158	0.08852	-0.56315

lnc-SLC25A48-3:3	1.030073	1.99859	5.791705	0.001581	0.08852	-0.56353
lnc-BNC2-4:1	-1.57601	3.804866	-5.79027	0.001583	0.08852	-0.56474
NONHSAT166981.1	-1.37345	1.985071	-5.78911	0.001584	0.08852	-0.56571
ENST00000445300.1	1.464996	4.08549	5.788335	0.001585	0.08852	-0.56637
NR_037584	-1.00319	2.103483	-5.78471	0.00159	0.088602	-0.56942
MSTRG.25854.6	1.303321	3.61632	5.784421	0.00159	0.088602	-0.56967
lnc-RAD54L-4:1	1.22948	3.659166	5.784337	0.00159	0.088602	-0.56974
lnc-DYDC1-9:1	-1.29383	6.095168	-5.78231	0.001593	0.088671	-0.57145
MSTRG.51008.21	2.419938	2.398071	5.78197	0.001594	0.088671	-0.57173
lnc-TCEANC-3:1	-1.88051	4.143926	-5.77618	0.001601	0.088739	-0.57661
lnc-ATP6AP2-9:1	-1.27496	2.001361	-5.7758	0.001602	0.088739	-0.57694
ENST00000614876.1	-1.84121	2.314453	-5.76859	0.001611	0.08881	-0.58302
MSTRG.14682.2	1.188182	2.020895	5.768592	0.001611	0.08881	-0.58302
lnc-ADSS-6:1	-1.25101	11.03163	-5.76604	0.001615	0.08881	-0.58518
NR_024608	-1.15832	6.363302	-5.76591	0.001615	0.08881	-0.58529
NONHSAT162616.1	-1.03312	5.677731	-5.76456	0.001617	0.08881	-0.58643
lnc-EPHA7-5:1	-1.15424	3.072885	-5.76387	0.001618	0.08881	-0.58702
lnc-FERD3L-1:1	-1.2837	2.211452	-5.76257	0.00162	0.08881	-0.58811
lnc-FGF23-5:3	1.14778	6.757047	5.758758	0.001625	0.088885	-0.59134
lnc-CYP24A1-3:1	-1.01988	9.484978	-5.75492	0.00163	0.088885	-0.59458
lnc-PMEPA1-2:1	-1.2932	2.815434	-5.75108	0.001635	0.088885	-0.59784
lnc-CRYBB1-4:5	1.497043	3.865764	5.749988	0.001637	0.088885	-0.59876
MSTRG.54885.1	1.316706	4.584676	5.748916	0.001638	0.088885	-0.59967
T076734	-1.52111	2.632797	-5.74817	0.001639	0.088885	-0.6003
T048538	1.00513	5.085518	5.745931	0.001642	0.088885	-0.6022
lnc-RBMX2-3:1	-1.25586	1.793213	-5.74191	0.001648	0.089088	-0.60561
MSTRG.4903.1	1.948531	2.45309	5.740844	0.001649	0.089101	-0.60652
NONHSAT197967.1	-1.07177	4.696964	-5.74046	0.00165	0.089101	-0.60685
ENST00000449805.2	1.003954	5.865412	5.735844	0.001656	0.089264	-0.61076
ENST00000416430.1	1.358044	1.804346	5.735721	0.001656	0.089264	-0.61087
lnc-IQCF3-1:4	1.734782	2.464553	5.733449	0.00166	0.089296	-0.6128
ENST00000500076.2	-2.12394	2.500333	-5.73141	0.001662	0.089348	-0.61453
NONHSAT219924.1	-1.39533	1.788482	-5.72793	0.001667	0.089466	-0.61749
ENST00000664048.1	-1.36452	1.792011	-5.72425	0.001672	0.089502	-0.62062
lnc-SLC10A7-4:1	-2.07112	2.120172	-5.7237	0.001673	0.089502	-0.62109
T055045	1.360203	1.698711	5.722252	0.001675	0.089502	-0.62232
NONHSAT176767.1	2.65761	2.430094	5.720504	0.001678	0.089502	-0.62381
lnc-CANT1-3:3	-1.02227	3.440283	-5.71794	0.001681	0.089533	-0.62599
ENST00000669616.1	1.129618	2.736922	5.715555	0.001685	0.089542	-0.62802
ENST00000552324.1	-2.39523	4.435686	-5.71483	0.001686	0.08955	-0.62864
NONHSAT191551.1	-1.40832	2.710216	-5.70705	0.001697	0.089846	-0.63527
NONHSAT158118.1	-1.57357	3.973769	-5.7045	0.0017	0.089846	-0.63745
NONHSAT178987.1	1.595418	4.177146	5.704014	0.001701	0.089846	-0.63786
MSTRG.5229.1	1.339139	3.51467	5.703982	0.001701	0.089846	-0.63789
lnc-MAP3K9-3:29	1.229725	5.458542	5.703932	0.001701	0.089846	-0.63793
lnc-SEMA6D-3:1	-1.59223	5.592901	-5.7034	0.001702	0.089846	-0.63839
MSTRG.36418.1	-1.05884	4.581389	-5.69816	0.001709	0.090054	-0.64286
MSTRG.53302.1	1.227629	3.461402	5.697504	0.00171	0.090057	-0.64342

lnc-FEZ2-6:1	-1.19445	4.758367	-5.69546	0.001713	0.090081	-0.64517
MSTRG.21093.1	1.484644	2.729737	5.690589	0.00172	0.090206	-0.64934
NR_002147	1.223598	4.609851	5.688601	0.001723	0.09031	-0.65104
NONHSAT176820.1	1.803327	2.442742	5.684844	0.001729	0.090503	-0.65426
NONHSAT204943.1	-1.53322	2.225626	-5.68294	0.001731	0.090507	-0.65588
NONHSAT186899.1	1.858058	2.029279	5.68028	0.001735	0.090541	-0.65816
NONHSAT154535.1	-1.62793	7.440364	-5.67647	0.001741	0.090627	-0.66143
ENST00000317596.3	-1.27341	3.289385	-5.67049	0.00175	0.090873	-0.66656
lnc-ABI1-11:1	2.847381	3.587251	5.66648	0.001756	0.090873	-0.67
NONHSAT221718.1	1.097677	4.220507	5.665491	0.001757	0.090873	-0.67085
lnc-GGPS1-17:1	-1.41187	2.118776	-5.66535	0.001757	0.090873	-0.67098
ENST00000608941.1	1.060813	5.131512	5.661569	0.001763	0.091024	-0.67422
ENST00000666326.1	1.066083	4.513307	5.661382	0.001763	0.091024	-0.67438
MSTRG.14867.1	-1.02441	3.597591	-5.66018	0.001765	0.091024	-0.67541
lnc-ALDH1A1-6:1	-1.27541	8.324934	-5.65975	0.001766	0.091024	-0.67579
NONHSAT161158.1	-1.42797	3.964102	-5.65798	0.001768	0.091073	-0.67731
MSTRG.50492.1	-1.10254	9.765908	-5.65531	0.001772	0.091087	-0.6796
NONHSAT171134.1	-1.28275	4.255824	-5.64986	0.001781	0.091111	-0.68429
NONHSAT165033.1	1.2922	1.851145	5.648044	0.001783	0.091115	-0.68586
lnc-CSN2-1:1	1.954982	2.839151	5.646445	0.001786	0.091115	-0.68724
NONHSAT172942.1	1.321318	3.727705	5.634905	0.001803	0.09133	-0.69719
lnc-ST8SIA4-7:1	-1.10033	8.458752	-5.63424	0.001804	0.09133	-0.69777
lnc-AHR-8:3	-1.10091	4.387378	-5.63249	0.001807	0.09133	-0.69928
lnc-NPAS4-4:1	1.370575	2.598161	5.629162	0.001812	0.091478	-0.70215
NONHSAT200276.1	-1.9234	3.806464	-5.62741	0.001815	0.091478	-0.70367
NONHSAT148476.1	-1.01169	7.964154	-5.62705	0.001815	0.091478	-0.70398
NONHSAT173365.1	1.240261	3.16166	5.623176	0.001821	0.091645	-0.70733
NONHSAT174328.1	1.839551	2.077998	5.619757	0.001827	0.091807	-0.71028
NONHSAT167950.1	-1.13522	8.953678	-5.61927	0.001827	0.091807	-0.7107
lnc-PKD1L3-1:1	-1.5565	3.011837	-5.61883	0.001828	0.091807	-0.71109
MSTRG.61572.1	1.026654	4.225985	5.610881	0.001841	0.0921	-0.71797
lnc-ALCAM-9:1	-1.05317	6.331178	-5.60638	0.001848	0.092153	-0.72187
lnc-ZBTB39-2:2	1.190778	1.882013	5.606146	0.001848	0.092153	-0.72208
PACRG-AS1:5	1.543791	1.929274	5.604256	0.001851	0.092255	-0.72372
MSTRG.52949.1	1.359091	2.113898	5.601761	0.001855	0.092265	-0.72588
NONHSAT155210.1	-1.198	1.85655	-5.60133	0.001856	0.092265	-0.72626
MSTRG.9189.2	-1.60885	2.170909	-5.59762	0.001861	0.092277	-0.72948
lnc-ARHGAP6-3:1	-1.38698	2.007069	-5.59473	0.001866	0.092277	-0.73199
lnc-CTAGE5-9:1	-1.23738	8.203932	-5.59439	0.001867	0.092277	-0.73228
ENST00000553410.1	-1.04963	1.746713	-5.59437	0.001867	0.092277	-0.7323
NONHSAT213694.1	-1.59101	1.947513	-5.59417	0.001867	0.092277	-0.73247
lnc-MDM4-12:1	-1.14271	3.729568	-5.59278	0.001869	0.092342	-0.73368
NONHSAT171840.1	1.180052	2.598808	5.588969	0.001875	0.092381	-0.73699
NONHSAT218022.1	-1.36415	3.860042	-5.58883	0.001875	0.092381	-0.73711
NONHSAT185560.1	1.011413	8.22811	5.584726	0.001882	0.092514	-0.74069
MSTRG.23774.1	-1.22508	4.631088	-5.58443	0.001883	0.092514	-0.74094
ENST00000568110.1	-1.3522	2.561324	-5.5833	0.001884	0.092514	-0.74193
ENST00000649539.1	1.606875	5.18417	5.582348	0.001886	0.092514	-0.74276

NONHSAT162363.1	-1.3841	4.209903	-5.58208	0.001886	0.092514	-0.74299
lnc-YAE1D1-4:1	1.087209	6.391619	5.57925	0.001891	0.092514	-0.74545
lnc-ADRA1D-2:3	-1.68208	2.115777	-5.57919	0.001891	0.092514	-0.74551
ENST00000658564.1	-1.41319	2.006739	-5.57918	0.001891	0.092514	-0.74552
ENST00000665497.1	-1.05176	7.711229	-5.57314	0.001901	0.092693	-0.75078
NONHSAT170367.1	-1.03395	4.120057	-5.57243	0.001902	0.092693	-0.7514
NONHSAT200961.1	-1.1945	3.840902	-5.5722	0.001902	0.092693	-0.7516
NONHSAT184331.1	-1.06623	4.074049	-5.57183	0.001903	0.092693	-0.75192
ENST00000620143.1	1.284289	3.749035	5.569506	0.001907	0.092814	-0.75395
lnc-SLC23A2-1:1	-1.38275	3.615117	-5.56908	0.001907	0.092814	-0.75432
NONHSAT200168.1	-1.05917	8.937655	-5.56156	0.00192	0.093197	-0.76089
lnc-HIST1H2BK-1:1	1.475512	2.021173	5.558707	0.001924	0.093331	-0.76338
lnc-LSAMP-4:1	-1.47385	7.840393	-5.55644	0.001928	0.093347	-0.76536
NONHSAT215038.1	1.788068	2.235769	5.556312	0.001928	0.093347	-0.76547
lnc-IKZF5-4:1	2.233147	3.510165	5.553745	0.001933	0.093508	-0.76772
ENST00000452288.1	-1.73012	2.557269	-5.54728	0.001943	0.093803	-0.77337
DNAJC27-AS1:22	-1.07212	1.800871	-5.54593	0.001946	0.093868	-0.77456
MSTRG.60057.2	2.584296	3.152338	5.54358	0.00195	0.093869	-0.77661
MSTRG.66747.1	-1.56557	2.760846	-5.53979	0.001956	0.093895	-0.77993
MSTRG.18129.1	1.116432	1.805022	5.528422	0.001975	0.094298	-0.78991
NONHSAT173782.1	-1.2793	2.449493	-5.52657	0.001978	0.09437	-0.79153
lnc-PKHD1-7:1	-1.71144	3.42489	-5.52362	0.001983	0.094538	-0.79413
T356962	1.3884	1.740145	5.523276	0.001984	0.094538	-0.79443
lnc-LSM3-5:2	1.373315	5.784023	5.513408	0.002001	0.094897	-0.80311
MSTRG.47820.1	1.417204	2.008352	5.512445	0.002003	0.094926	-0.80396
NONHSAT214281.1	1.382719	2.076216	5.509291	0.002008	0.094946	-0.80674
NONHSAT205841.1	1.279819	1.696637	5.50656	0.002013	0.094946	-0.80914
MSTRG.52091.5	2.075835	2.612061	5.506065	0.002014	0.094946	-0.80958
T314711	1.847752	2.615975	5.504078	0.002017	0.094946	-0.81133
ENST00000655744.1	1.460222	1.871381	5.502117	0.00202	0.094979	-0.81306
NONHSAT214440.1	1.278332	1.835459	5.495471	0.002032	0.095215	-0.81893
NONHSAT211991.1	-1.29697	1.698228	-5.49221	0.002038	0.095319	-0.8218
ENST00000450486.1	1.325808	1.841556	5.49104	0.00204	0.095329	-0.82284
NONHSAT216702.1	-1.38789	4.469377	-5.49016	0.002041	0.095343	-0.82362
ENST00000448256.1	-1.08688	8.984181	-5.48723	0.002047	0.095516	-0.82621
lnc-SLC35F5-10:7	1.273373	5.899643	5.483618	0.002053	0.09559	-0.8294
NONHSAT158025.1	-1.40897	2.769489	-5.48177	0.002056	0.095595	-0.83104
lnc-LRRC52-1:1	-1.0288	8.424424	-5.48119	0.002057	0.095599	-0.83155
lnc-TSPAN2-1:1	1.302114	3.131312	5.478219	0.002063	0.095694	-0.83418
NONHSAT197326.1	1.489203	1.839579	5.475421	0.002068	0.095726	-0.83666
NONHSAT170209.1	1.149737	6.627289	5.473417	0.002071	0.095769	-0.83843
NONHSAT206940.1	-1.29202	7.027985	-5.47301	0.002072	0.095769	-0.8388
ENST00000418471.1	1.563234	2.214542	5.471874	0.002074	0.095769	-0.8398
lnc-TMEM74-1:2	1.117165	4.131384	5.460988	0.002094	0.096191	-0.84946
lnc-SMIM36-1:1	-1.44581	9.650788	-5.45992	0.002096	0.096227	-0.85041
NONHSAT199381.1	-1.24834	1.940755	-5.45843	0.002098	0.096245	-0.85173
lnc-SMARCAD1-1:1	-1.03669	8.628417	-5.45725	0.002101	0.096245	-0.85278
lnc-AASDHPPT-4:1	1.031343	1.548405	5.456561	0.002102	0.096245	-0.85339

MSTRG.13498.2	-1.12207	3.60767	-5.45307	0.002108	0.096406	-0.85649
NONHSAT169528.1	1.332588	4.910885	5.451453	0.002111	0.096406	-0.85793
lnc-PRRG4-4:1	-1.53959	3.033164	-5.4513	0.002111	0.096406	-0.85807
lnc-TEX47-4:1	-1.12837	2.689087	-5.44425	0.002124	0.096417	-0.86434
NONHSAT210153.1	1.145731	3.892831	5.443605	0.002126	0.096417	-0.86491
NONHSAT200029.1	-1.78249	2.548267	-5.43118	0.002149	0.096943	-0.87599
lnc-FSCN2-1:7	1.571776	3.664921	5.428807	0.002153	0.097033	-0.87811
MSTRG.57138.1	-1.17249	3.054058	-5.42328	0.002164	0.097313	-0.88304
NONHSAT206663.1	1.447806	2.415734	5.422535	0.002165	0.097313	-0.88371
lnc-NR5A2-6:1	-1.12044	3.041024	-5.41968	0.00217	0.097376	-0.88626
MSTRG.45998.1	1.12534	1.639803	5.418884	0.002172	0.097376	-0.88697
lnc-CDK5R1-3:1	-1.42156	2.117874	-5.41411	0.002181	0.097613	-0.89124
ENST00000445589.1	2.064268	5.2751	5.412864	0.002183	0.097676	-0.89235
lnc-TP53BP2-5:1	-1.35021	7.163099	-5.41123	0.002187	0.097772	-0.89382
ENST00000653622.1	1.825494	2.300635	5.408429	0.002192	0.09787	-0.89632
lnc-RHBDD3-3:3	1.096445	3.155992	5.403795	0.002201	0.098149	-0.90047
lnc-ENDOD1-6:1	-1.75441	2.628096	-5.39956	0.002209	0.098274	-0.90427
ENST00000457890.1	1.714756	2.27112	5.394867	0.002218	0.098402	-0.90848
lnc-AKR1D1-5:2	1.171868	1.669292	5.394046	0.00222	0.098402	-0.90922
NONHSAT167589.1	1.478418	2.000387	5.393747	0.00222	0.098402	-0.90949
lnc-MRPL44-5:1	-1.80708	5.544352	-5.39261	0.002223	0.098402	-0.91051
MSTRG.33210.1	1.725183	2.268973	5.392576	0.002223	0.098402	-0.91054
MSTRG.7233.1	-1.28571	2.710019	-5.3918	0.002224	0.098402	-0.91123
lnc-JAG1-3:3	1.617906	1.855782	5.389564	0.002228	0.098428	-0.91324
lnc-USP6NL-7:12	1.034016	6.288398	5.386106	0.002235	0.098503	-0.91635
NONHSAT187637.1	2.308022	4.285567	5.385292	0.002237	0.098503	-0.91708
lnc-FZD7-1:1	1.115032	4.459599	5.383568	0.00224	0.098503	-0.91863
lnc-AAGAB-3:4	-1.6623	2.460448	-5.38343	0.002241	0.098503	-0.91875
lnc-TRMT61B-7:2	1.393078	3.786248	5.381673	0.002244	0.098503	-0.92033
lnc-PATE4-3:1	-1.67982	3.239178	-5.37834	0.002251	0.098503	-0.92333
ENST00000645656.1	1.702208	2.080986	5.378212	0.002251	0.098503	-0.92345
ZNF582-AS1:39	1.29152	1.999087	5.373174	0.002261	0.098693	-0.92798
NONHSAT202366.1	-2.03995	2.257488	-5.37287	0.002261	0.098693	-0.92826
lnc-ZC3H15-4:1	1.120726	2.280638	5.368452	0.00227	0.098964	-0.93224
lnc-PTPN2-10:1	2.496604	2.833197	5.364529	0.002278	0.099107	-0.93577
MSTRG.29606.1	-1.1764	1.952717	-5.36115	0.002285	0.099241	-0.93882
NONHSAT206254.1	1.358274	3.3804	5.357729	0.002292	0.099341	-0.94191
lnc-BST2-1:1	-1.61091	6.50252	-5.35403	0.002299	0.099426	-0.94525
lnc-CAPRIN2-2:8	-1.11651	2.414466	-5.3534	0.0023	0.099426	-0.94582
NONHSAT205448.1	1.62834	2.280282	5.350971	0.002305	0.09945	-0.94802
lnc-TANC1-6:2	-1.3055	1.93005	-5.34988	0.002308	0.09945	-0.949
MSTRG.32658.1	1.329731	1.723176	5.347119	0.002313	0.099459	-0.9515
ENST00000611741.1	-1.52211	2.66977	-5.34047	0.002327	0.099607	-0.95751
NONHSAT186381.1	-1.1522	6.260011	-5.33673	0.002335	0.099829	-0.9609
ENST00000527443.1	1.031047	2.852988	5.333635	0.002341	0.099997	-0.96371
ENST00000512730.1	1.25251	1.688965	5.333393	0.002341	0.099997	-0.96392
lnc-ERP29-2:1	-1.07924	5.296946	-5.33131	0.002346	0.100092	-0.96581
NONHSAT213288.1	-1.68822	2.82462	-5.33088	0.002347	0.100092	-0.9662

T279189	1.032437	1.616723	5.329555	0.002349	0.100104	-0.96741
MSTRG.58940.1	-1.85264	2.896028	-5.32709	0.002355	0.100224	-0.96964
lnc-ECHDC3-2:2	1.012087	5.382458	5.321896	0.002365	0.100312	-0.97436
lnc-OR11A1-1:1	-1.10597	3.263607	-5.32161	0.002366	0.100312	-0.97462
NONHSAT170971.1	-1.0888	1.643176	-5.31792	0.002374	0.100312	-0.97797
NONHSAT201110.1	1.242175	3.95625	5.31738	0.002375	0.100312	-0.97846
lnc-SEPSECS-4:2	-1.3624	5.165703	-5.31683	0.002376	0.100312	-0.97896
lnc-CD177-1:2	-1.23503	2.270821	-5.31385	0.002382	0.100423	-0.98167
ENST00000655326.1	1.358168	1.947457	5.309396	0.002392	0.100457	-0.98572
NONHSAT170875.1	-1.92082	2.517415	-5.30635	0.002398	0.100457	-0.98849
NR_120606	-1.11869	1.978012	-5.30619	0.002399	0.100457	-0.98864
NONHSAT174502.1	-1.33489	3.24009	-5.30509	0.002401	0.100457	-0.98964
NR_110944	1.097211	3.323838	5.304782	0.002402	0.100457	-0.98992
NONHSAT191219.1	1.270867	4.890177	5.304083	0.002403	0.100457	-0.99055
NONHSAT175877.1	-1.23903	2.355854	-5.30372	0.002404	0.100457	-0.99088
lnc-SETX-2:1	1.220884	1.962709	5.303005	0.002405	0.100457	-0.99154
NONHSAT169483.1	-1.18196	4.543063	-5.30291	0.002406	0.100457	-0.99163
NONHSAT193414.1	1.268898	1.975327	5.301934	0.002408	0.100457	-0.99251
NONHSAT197428.1	1.887645	2.579136	5.298952	0.002414	0.100457	-0.99523
lnc-CHST8-5:3	-1.1762	7.775483	-5.29841	0.002415	0.100457	-0.99573
NONHSAT197463.1	-1.17756	2.53075	-5.29804	0.002416	0.100457	-0.99606
lnc-HSPB9-1:1	-1.165	1.89664	-5.29726	0.002418	0.100457	-0.99677
MSTRG.28141.1	1.0278	4.020468	5.295885	0.002421	0.100457	-0.99803
NONHSAT167997.1	2.475354	2.619418	5.294679	0.002423	0.100457	-0.99913
MSTRG.40495.1	1.731368	2.078363	5.294315	0.002424	0.100457	-0.99946
lnc-MDH1-2:1	-1.19487	6.597455	-5.29307	0.002427	0.100486	-1.00059
T313558	2.797876	2.946431	5.292393	0.002428	0.100493	-1.00121
NONHSAT177925.1	1.05749	2.353765	5.289953	0.002433	0.100587	-1.00344
ENST00000659499.1	-1.98523	2.539562	-5.28733	0.002439	0.100597	-1.00583
lnc-CA6-5:1	-1.45075	5.334817	-5.2869	0.00244	0.100597	-1.00622
MSTRG.59388.1	-1.62832	2.184154	-5.28321	0.002448	0.100722	-1.0096
NONHSAT205898.1	1.397433	2.027909	5.279536	0.002456	0.100876	-1.01295
lnc-ZNF286A-2:1	-1.57807	6.376017	-5.27641	0.002463	0.101032	-1.01581
lnc-SZRD1-5:2	1.279616	3.577579	5.274984	0.002466	0.101078	-1.01712
ENST00000509194.1	1.218618	5.876646	5.273296	0.00247	0.101189	-1.01866
lnc-SMKR1-2:2	-1.2892	10.83093	-5.27126	0.002474	0.101279	-1.02053
MSTRG.25480.3	-1.03252	8.152747	-5.27107	0.002475	0.101279	-1.0207
ENST00000616774.1	1.070603	4.848866	5.270545	0.002476	0.101279	-1.02118
lnc-ACMSD-3:1	-1.30851	1.827277	-5.26907	0.002479	0.101301	-1.02253
lnc-SLC17A5-2:1	-1.20503	4.561686	-5.26889	0.002479	0.101301	-1.0227
lnc-IFT74-5:1	-1.02357	12.01963	-5.26644	0.002485	0.101439	-1.02494
ENST00000567624.1	-1.55545	2.780899	-5.26076	0.002498	0.101789	-1.03015
T273562	-1.18008	3.514714	-5.25823	0.002503	0.101836	-1.03247
NONHSAT197159.1	-1.59534	5.314224	-5.25779	0.002504	0.101836	-1.03287
lnc-ARPC1A-7:3	1.093313	1.654654	5.257728	0.002504	0.101836	-1.03293
lnc-SLC2A9-7:1	-1.02477	6.457562	-5.25762	0.002505	0.101836	-1.03303
ENST00000598561.1	2.180246	2.925413	5.257535	0.002505	0.101836	-1.03311
lnc-ZNF219-1:1	2.02304	2.430803	5.255939	0.002508	0.101899	-1.03457

lnc-MGAT4C-1:4	-1.24609	6.135576	-5.25417	0.002512	0.101978	-1.03619
T267303	1.210564	4.329407	5.249734	0.002522	0.102136	-1.04027
lnc-CYYR1-1:2	-1.3114	5.673273	-5.24882	0.002524	0.102178	-1.04111
NR_027003	1.814207	2.312825	5.246205	0.00253	0.102198	-1.04351
lnc-RBMS1-9:1	1.009244	1.728351	5.244021	0.002535	0.102198	-1.04552
NONHSAT154155.1	1.613236	2.385142	5.242546	0.002538	0.10222	-1.04688
NONHSAT224156.1	1.041646	5.080318	5.23976	0.002545	0.102394	-1.04944
ENST00000456771.1	-1.70823	2.656594	-5.23273	0.002561	0.102743	-1.05591
lnc-EYA4-3:1	-1.13912	8.342967	-5.23216	0.002562	0.102743	-1.05644
lnc-ATP10B-2:1	-1.13394	2.989985	-5.22768	0.002573	0.102835	-1.06056
ENST00000655097.1	1.577292	1.895972	5.226969	0.002574	0.102835	-1.06122
lnc-HNRNPA1P48-15:1	-1.01908	2.264028	-5.22434	0.00258	0.102868	-1.06365
ENST00000538041.1	2.021763	2.183863	5.223293	0.002583	0.102868	-1.06461
MSTRG.6432.1	1.526168	1.84007	5.222819	0.002584	0.102868	-1.06505
lnc-SPATA19-9:3	1.451327	2.071006	5.221254	0.002587	0.102868	-1.0665
NONHSAT160364.1	-1.1106	3.76047	-5.21868	0.002593	0.102868	-1.06888
lnc-PAQR9-5:1	2.225129	2.245757	5.20662	0.002622	0.103623	-1.08002
lnc-LYPD6-10:1	-1.60013	9.808474	-5.2036	0.002629	0.103824	-1.08282
NR_033840	1.185692	3.602611	5.202757	0.002631	0.103861	-1.08359
NONHSAT180357.1	1.218786	2.683521	5.200526	0.002636	0.103989	-1.08566
NONHSAT154619.1	-1.35865	3.946813	-5.19833	0.002641	0.104154	-1.08769
NONHSAT205068.1	1.694155	2.70686	5.196204	0.002646	0.104274	-1.08966
NONHSAT209092.1	-1.13784	7.388634	-5.19582	0.002647	0.104274	-1.09002
lnc-FXYD4-8:2	1.316357	1.753789	5.191098	0.002659	0.104426	-1.0944
ENST00000648514.1	-1.6553	2.66041	-5.1903	0.00266	0.10443	-1.09514
lnc-OLFM4-4:4	-1.30319	3.291599	-5.18598	0.002671	0.104664	-1.09914
ENST00000455257.2	1.133018	3.803108	5.184215	0.002675	0.104772	-1.10078
NONHSAT201480.1	-1.64743	2.645362	-5.18391	0.002676	0.104772	-1.10107
lnc-KLHL42-4:1	-1.21656	5.853425	-5.18312	0.002678	0.104772	-1.1018
NONHSAT201901.1	1.139213	5.064228	5.182633	0.002679	0.104777	-1.10225
NONHSAT224090.1	1.733501	3.489873	5.180665	0.002684	0.104801	-1.10408
NONHSAT164680.1	1.266134	2.675699	5.176577	0.002694	0.104985	-1.10788
lnc-CDK19-5:1	-1.77144	2.350983	-5.17402	0.0027	0.105105	-1.11025
NONHSAT212318.1	-1.23273	1.989927	-5.17123	0.002707	0.105192	-1.11285
lnc-FGD2-2:1	-1.02891	4.821834	-5.17074	0.002708	0.105192	-1.11331
NONHSAT170362.1	1.257249	1.648348	5.166967	0.002717	0.105299	-1.11682
T113219	1.379944	3.553191	5.166609	0.002718	0.105299	-1.11716
MSTRG.56903.1	-1.45518	3.060525	-5.16645	0.002718	0.105299	-1.11731
MSTRG.10019.1	1.771577	2.690774	5.164494	0.002723	0.10542	-1.11913
lnc-CYTH4-2:1	1.310626	2.07564	5.158477	0.002738	0.105747	-1.12473
NONHSAT224461.1	-1.33537	1.784378	-5.15645	0.002743	0.105814	-1.12662
lnc-C5orf30-7:1	1.188377	3.364295	5.155876	0.002744	0.105814	-1.12716
LINC02542:7	-1.52753	2.874869	-5.15095	0.002757	0.106004	-1.13176
ENST00000531379.1	-1.59515	2.178789	-5.14686	0.002767	0.106123	-1.13558
MSTRG.71225.1	-1.48817	2.451862	-5.14017	0.002784	0.106409	-1.14183
lnc-TMC6-11:1	1.374701	2.190846	5.140122	0.002784	0.106409	-1.14187
NONHSAT154364.1	1.248189	2.937298	5.133306	0.002801	0.106603	-1.14825
lnc-COMTD1-1:1	-1.76218	2.956315	-5.13282	0.002802	0.106603	-1.14871

NONHSAT188097.1	1.559118	3.246964	5.132287	0.002804	0.106603	-1.1492
MSTRG.52490.1	-1.51835	6.194658	-5.1308	0.002808	0.106627	-1.1506
NONHSAT222138.1	-1.39911	2.155213	-5.12846	0.002814	0.106726	-1.15279
NONHSAT157805.1	1.166205	1.602809	5.127953	0.002815	0.106726	-1.15326
lnc-AMELY-24:1	1.30634	4.23588	5.124697	0.002823	0.106726	-1.15632
lnc-PLCB1-2:1	-1.17657	2.486736	-5.12441	0.002824	0.106726	-1.15659
NONHSAT205701.1	-1.3906	2.011577	-5.12297	0.002828	0.106746	-1.15793
NONHSAT202035.1	-1.54886	8.090324	-5.12096	0.002833	0.10678	-1.15982
ENST00000534076.2	1.934648	2.083823	5.116162	0.002845	0.106971	-1.16432
NONHSAT193183.1	-1.26911	5.207187	-5.11613	0.002845	0.106971	-1.16435
NONHSAT199698.1	-1.97405	3.719306	-5.11548	0.002847	0.106977	-1.16496
NONHSAT220584.1	-1.28257	1.727119	-5.11371	0.002852	0.107008	-1.16663
ENST00000587412.1	1.099462	5.6722	5.111391	0.002858	0.10716	-1.1688
NONHSAT211118.1	1.400763	1.944291	5.110924	0.002859	0.10716	-1.16924
MSTRG.18758.1	-1.241	1.930469	-5.10985	0.002862	0.107226	-1.17026
NONHSAT164705.1	2.072213	2.055787	5.108671	0.002865	0.107254	-1.17136
lnc-SEC24C-7:1	-1.0668	3.896549	-5.10728	0.002868	0.107317	-1.17267
NONHSAT200121.1	1.321392	2.914023	5.105137	0.002874	0.107447	-1.17468
NONHSAT212525.1	1.457433	2.172612	5.104171	0.002877	0.107502	-1.17559
NONHSAT197711.1	-1.43011	2.693798	-5.10181	0.002883	0.107534	-1.17781
NONHSAT189659.1	1.030906	4.054568	5.097131	0.002895	0.107796	-1.18222
lnc-CCDC190-1:2	1.349569	2.854745	5.096883	0.002896	0.107796	-1.18245
MSTRG.40703.1	1.516086	2.107404	5.095907	0.002898	0.107836	-1.18337
MSTRG.62694.1	1.502196	3.092956	5.094765	0.002901	0.107869	-1.18444
lnc-BTBD18-1:1	-1.04334	6.970771	-5.09385	0.002904	0.107883	-1.1853
NONHSAT214360.1	-1.06771	1.862173	-5.09265	0.002907	0.107924	-1.18644
MSTRG.7129.1	1.434153	1.761176	5.092599	0.002907	0.107924	-1.18649
NONHSAT157973.1	1.86139	3.669581	5.09176	0.002909	0.107937	-1.18728
NONHSAT184723.1	1.139661	2.679126	5.089971	0.002914	0.107945	-1.18896
lnc-C11orf54-3:1	-1.0577	4.063986	-5.08932	0.002916	0.107969	-1.18957
lnc-CDH6-137:1	1.020684	4.579593	5.08813	0.002919	0.107991	-1.1907
T152287	-1.13105	5.084232	-5.08582	0.002925	0.108112	-1.19288
NONHSAT163317.1	-1.11642	9.413419	-5.08546	0.002926	0.108112	-1.19322
NONHSAT200951.1	-1.17863	8.654492	-5.08462	0.002928	0.108155	-1.19401
NONHSAT178775.1	1.127252	1.845415	5.083076	0.002932	0.108159	-1.19547
ENST00000431019.1	-1.4657	2.517366	-5.08204	0.002935	0.108174	-1.19644
MSTRG.45502.1	-1.68529	1.976819	-5.0804	0.00294	0.108174	-1.198
NONHSAT218810.1	1.056344	1.631946	5.07957	0.002942	0.108174	-1.19878
NONHSAT214741.1	-1.2087	1.904357	-5.07887	0.002944	0.108174	-1.19943
lnc-AHSP-5:1	-1.02221	7.662781	-5.07839	0.002945	0.108174	-1.19989
lnc-MTNR1B-3:1	1.31899	2.847579	5.078323	0.002945	0.108174	-1.19995
NONHSAT189657.1	1.11028	3.060801	5.077925	0.002946	0.108174	-1.20033
ENST00000642235.1	1.153201	1.849066	5.076744	0.00295	0.108185	-1.20145
lnc-NFASC-1:1	1.12936	5.021401	5.07495	0.002954	0.10828	-1.20314
T011580	-1.54952	3.183216	-5.07459	0.002955	0.10828	-1.20348
lnc-SAXO1-1:1	-1.10682	4.346815	-5.07277	0.00296	0.10828	-1.2052
NONHSAT210563.1	1.20998	4.200681	5.070133	0.002967	0.108408	-1.20769
lnc-EZR-2:1	1.717592	1.944243	5.059252	0.002997	0.109157	-1.21799

NONHSAT154683.1	-1.18603	3.540273	-5.05251	0.003016	0.10939	-1.22439
lnc-ETFBKMT-2:1	-1.15916	6.760664	-5.05242	0.003016	0.10939	-1.22447
NR_038292	2.183107	3.385862	5.050712	0.003021	0.109414	-1.22609
lnc-ELOA-2:2	1.221652	5.236538	5.049293	0.003025	0.109431	-1.22744
NONHSAT173654.1	-1.07699	8.201881	-5.04654	0.003032	0.109507	-1.23005
ENST00000657275.1	-1.22722	1.741152	-5.04427	0.003039	0.109558	-1.23221
lnc-TGIF1-12:2	-1.26641	3.847936	-5.04378	0.00304	0.109558	-1.23267
ENST00000412298.5	-1.23476	5.889262	-5.04368	0.00304	0.109558	-1.23276
NR_110248	1.077717	1.925962	5.034291	0.003067	0.110004	-1.24169
lnc-ZNF706-9:1	-1.61327	2.934637	-5.0326	0.003072	0.110051	-1.2433
ENST00000618027.1	1.085674	4.839773	5.032533	0.003072	0.110051	-1.24336
ENST00000561816.1	-2.18421	3.031142	-5.0319	0.003073	0.110051	-1.24396
ENST00000441613.1	-1.29201	1.89639	-5.03088	0.003076	0.110076	-1.24493
lnc-NPY5R-5:1	-1.158	9.081305	-5.0242	0.003095	0.110382	-1.2513
lnc-CCT6B-6:2	1.515768	1.870484	5.024052	0.003096	0.110382	-1.25144
lnc-TLE3-15:3	-1.38572	1.86154	-5.02267	0.0031	0.110481	-1.25275
NONHSAT153844.1	1.268399	2.166658	5.021907	0.003102	0.11052	-1.25348
MSTRG.22526.3	-1.01134	2.20192	-5.02104	0.003104	0.11053	-1.25431
lnc-EGFL6-8:4	1.321502	4.32008	5.019031	0.00311	0.110581	-1.25623
T318529	-1.53209	2.619754	-5.01812	0.003113	0.110581	-1.25709
MSTRG.18563.1	1.662814	1.995542	5.016686	0.003117	0.110581	-1.25846
NONHSAT170160.1	-1.68053	2.822815	-5.01473	0.003123	0.110636	-1.26033
lnc-CISH-4:1	-2.08638	6.189131	-5.01349	0.003126	0.110643	-1.26151
NONHSAT193289.1	-1.36499	2.346088	-5.01311	0.003127	0.110643	-1.26188
lnc-SH3BP4-8:1	1.303562	2.0081	5.012218	0.00313	0.110653	-1.26273
ENST00000521188.1	1.29212	1.757755	5.011639	0.003131	0.110657	-1.26328
NONHSAT218166.1	1.394739	1.738403	5.01067	0.003134	0.110695	-1.2642
lnc-LAMA1-7:1	1.498867	1.949905	5.007333	0.003144	0.110765	-1.26739
NONHSAT182696.1	1.812044	2.012525	5.006912	0.003145	0.110767	-1.26779
T129768	1.201768	4.207314	5.004775	0.003151	0.110872	-1.26984
NONHSAT187701.1	1.014969	1.705887	5.004199	0.003153	0.110892	-1.27039
NONHSAT172134.1	1.506583	2.170083	5.002849	0.003157	0.110954	-1.27168
MSTRG.45333.1	-1.60232	4.295824	-5.00196	0.00316	0.110968	-1.27253
lnc-ZIC1-21:1	1.671221	4.58213	4.998952	0.003168	0.111177	-1.2754
NONHSAT191848.1	1.098591	3.932836	4.996163	0.003177	0.111254	-1.27807
ENST00000510433.1	1.259079	1.677816	4.995221	0.003179	0.111313	-1.27898
NONHSAT218126.1	2.041409	2.423294	4.992543	0.003187	0.111439	-1.28154
MSTRG.64707.2	2.020527	2.435855	4.990975	0.003192	0.111439	-1.28304
MSTRG.8348.1	1.216597	1.708025	4.990079	0.003195	0.111439	-1.2839
T165321	-1.60472	2.55355	-4.98984	0.003195	0.111439	-1.28413
lnc-PIGC-5:1	1.319709	2.200109	4.989821	0.003195	0.111439	-1.28415
NONHSAT211686.1	-1.40686	4.277808	-4.98695	0.003204	0.111439	-1.2869
lnc-AMIGO2-8:2	-1.12609	10.91368	-4.98539	0.003208	0.111439	-1.2884
NONHSAT170464.1	2.086096	2.400768	4.984368	0.003212	0.111439	-1.28937
lnc-TMEM250-3:2	1.674626	2.286447	4.982163	0.003218	0.111439	-1.29149
ENST00000548010.1	-1.32272	1.916612	-4.98171	0.003219	0.111439	-1.29193
NONHSAT187289.1	-1.23122	2.918398	-4.9817	0.003219	0.111439	-1.29193
MSTRG.27104.1	-1.47822	2.537311	-4.97832	0.00323	0.111583	-1.29518

lnc-GPR39-2:6	1.124984	3.190547	4.978066	0.00323	0.111583	-1.29542
ENST00000662052.1	-1.0535	3.735677	-4.9747	0.00324	0.111583	-1.29866
lnc-NTRK2-1:1	-2.30855	3.65008	-4.97458	0.003241	0.111583	-1.29877
lnc-DDX58-3:1	-1.38783	3.023775	-4.97421	0.003242	0.111583	-1.29912
NONHSAT222229.1	1.070831	4.550694	4.971662	0.00325	0.111583	-1.30157
MSTRG.795.1	1.684245	1.869407	4.970596	0.003253	0.111609	-1.30259
lnc-SLC19A3-2:1	-1.03125	6.481438	-4.96683	0.003264	0.111644	-1.30622
lnc-ATAD2-2:1	2.128675	3.936035	4.963374	0.003275	0.111644	-1.30954
NONHSAT158127.1	1.020792	4.797856	4.963128	0.003275	0.111644	-1.30978
NONHSAT164501.1	-1.01874	9.119863	-4.9609	0.003282	0.111644	-1.31192
ENST00000454622.2	1.256983	4.882323	4.960554	0.003283	0.111644	-1.31225
NONHSAT160858.1	1.144948	1.656745	4.960149	0.003285	0.111644	-1.31264
NONHSAT168816.1	-1.64486	2.491837	-4.9601	0.003285	0.111644	-1.31269
lnc-SPTSSB-4:1	-1.59318	3.022456	-4.9595	0.003287	0.111644	-1.31327
lnc-KIDINS220-20:1	1.066154	1.618762	4.958787	0.003289	0.111644	-1.31396
NONHSAT186349.1	2.010148	2.555728	4.958726	0.003289	0.111644	-1.31401
T302913	1.154427	1.80669	4.958384	0.00329	0.111644	-1.31434
lnc-FGF9-15:2	1.053605	2.913309	4.957414	0.003293	0.111644	-1.31528
NR_027420	-1.35857	4.550863	-4.95629	0.003296	0.111644	-1.31636
MSTRG.70000.1	1.406327	4.926672	4.955258	0.0033	0.111644	-1.31735
ENST00000657876.1	-1.17208	3.653191	-4.95165	0.003311	0.111816	-1.32083
lnc-SSX5-4:1	-1.94585	2.688859	-4.95144	0.003311	0.111816	-1.32103
lnc-MROH2B-2:1	-1.04776	10.29122	-4.94385	0.003335	0.112224	-1.32836
MSTRG.35265.1	1.982344	2.252011	4.94359	0.003336	0.112224	-1.32861
MSTRG.35113.1	1.495613	3.581254	4.94294	0.003338	0.112254	-1.32923
NONHSAT184293.1	2.834646	3.777321	4.940739	0.003345	0.11241	-1.33136
lnc-UCHL5-7:1	-1.07741	3.819402	-4.93358	0.003367	0.11277	-1.33828
lnc-PERP-1:4	-1.52975	2.814739	-4.93119	0.003374	0.11277	-1.34059
NONHSAT167812.1	-1.1701	1.926245	-4.93091	0.003375	0.11277	-1.34086
NONHSAT176082.1	-2.05453	2.60419	-4.92813	0.003384	0.112899	-1.34355
lnc-BLID-6:1	-1.51591	2.66805	-4.92737	0.003387	0.112899	-1.34428
lnc-PKD2L1-1:3	1.224698	4.417572	4.926537	0.003389	0.112899	-1.34509
ENST00000670949.1	1.653211	2.40516	4.926468	0.003389	0.112899	-1.34516
NONHSAT177137.1	-1.12632	2.669573	-4.92318	0.0034	0.113127	-1.34834
ENST00000587527.1	-1.22649	4.649248	-4.92068	0.003408	0.113215	-1.35076
lnc-CEP128-7:1	-1.45666	1.931453	-4.91895	0.003413	0.113354	-1.35244
MSTRG.1888.1	1.466947	1.880207	4.918354	0.003415	0.113354	-1.35302
lnc-HRASLS2-1:1	-1.7101	2.143685	-4.918	0.003416	0.113354	-1.35336
ENST00000611513.1	1.17675	5.491184	4.917966	0.003416	0.113354	-1.35339
lnc-EPPIN-WFDC6-1:1	-1.0644	1.900706	-4.91703	0.003419	0.113363	-1.3543
lnc-MAP3K8-16:1	1.076182	3.031452	4.91506	0.003426	0.113364	-1.35621
ENST00000663471.1	-1.22806	3.048605	-4.91224	0.003435	0.113549	-1.35894
lnc-MET-1:1	-1.83491	2.669663	-4.91111	0.003438	0.113549	-1.36004
lnc-CYP7B1-3:1	1.258247	1.977819	4.907448	0.00345	0.113659	-1.36359
lnc-VIRMA-1:3	1.000269	1.597093	4.907283	0.003451	0.113659	-1.36375
lnc-LARP1-3:1	-1.04778	8.72461	-4.90675	0.003453	0.113659	-1.36427
lnc-NUDCD2-12:1	1.463754	3.633378	4.906701	0.003453	0.113659	-1.36432
lnc-TPGS2-2:1	1.112237	3.707413	4.904625	0.003459	0.113744	-1.36634

ENST00000511875.1	1.451117	1.810954	4.903872	0.003462	0.113744	-1.36707
ENST00000659521.1	-1.07459	6.737657	-4.90337	0.003463	0.113744	-1.36755
ENST00000508179.1	1.139641	5.781679	4.90288	0.003465	0.113744	-1.36803
lnc-ZBBX-3:1	1.586683	1.829633	4.901641	0.003469	0.113744	-1.36924
T377599	1.241302	1.992271	4.90127	0.00347	0.113744	-1.3696
NONHSAT198699.1	-1.07139	7.204145	-4.9008	0.003472	0.113744	-1.37005
NONHSAT198425.1	1.247512	5.38697	4.900026	0.003474	0.113744	-1.37081
lnc-NAALADL2-14:1	1.224031	3.601229	4.898955	0.003478	0.113744	-1.37185
lnc-GCNT1-4:6	1.121543	1.752465	4.893596	0.003495	0.114055	-1.37706
lnc-TFDP3-1:1	-1.4407	8.123646	-4.89199	0.003501	0.114055	-1.37862
NONHSAT177192.1	-1.28541	3.581281	-4.89175	0.003501	0.114055	-1.37885
ENST00000521984.1	-1.84665	2.574113	-4.89157	0.003502	0.114055	-1.37903
MSTRG.32925.1	1.589356	2.240598	4.889158	0.00351	0.114112	-1.38138
lnc-TSPY4-8:1	1.767557	2.485028	4.888426	0.003512	0.114112	-1.38209
lnc-TAS2R1-13:1	2.09692	2.149513	4.885849	0.003521	0.114235	-1.3846
ENST00000508713.1	-1.38456	5.325459	-4.88462	0.003525	0.114286	-1.3858
lnc-POU4F2-1:1	1.685495	2.290693	4.882197	0.003533	0.114397	-1.38816
NONHSAT149085.1	2.358831	3.126099	4.880329	0.003539	0.114397	-1.38998
NONHSAT189312.1	1.376384	2.120642	4.879722	0.003541	0.114408	-1.39057
lnc-PRDM6-2:1	-1.41695	2.800626	-4.87738	0.003549	0.114527	-1.39285
lnc-C3orf30-11:1	1.724009	3.105536	4.875627	0.003555	0.114556	-1.39457
NR_110807	-1.15993	2.706813	-4.87518	0.003556	0.114568	-1.39501
ENST00000478759.2	-1.06647	6.312511	-4.87399	0.00356	0.11466	-1.39616
MSTRG.36858.1	1.161825	2.74882	4.869868	0.003574	0.114735	-1.40019
MSTRG.6619.1	-1.10692	2.961009	-4.86906	0.003577	0.114735	-1.40098
NONHSAT215045.1	-1.55023	2.087583	-4.86715	0.003583	0.114841	-1.40284
lnc-TENM1-4:1	1.112605	1.728447	4.865142	0.00359	0.11487	-1.40481
lnc-WNT2-3:1	-1.00923	2.089842	-4.86139	0.003603	0.115001	-1.40848
MSTRG.67257.46	1.508694	1.840872	4.860463	0.003606	0.115034	-1.40938
lnc-TMEM184C-7:1	1.273548	2.456874	4.859118	0.003611	0.115066	-1.4107
lnc-RFESD-1:1	-1.37603	1.860103	-4.85838	0.003613	0.115073	-1.41142
lnc-ASCL1-1:1	-1.08037	1.845427	-4.85785	0.003615	0.115094	-1.41194
MSTRG.22271.3	-1.42182	2.664111	-4.85567	0.003622	0.115106	-1.41407
T127511	-1.45584	2.051382	-4.85499	0.003625	0.115106	-1.41474
ENST00000443897.1	1.425629	1.831089	4.852918	0.003632	0.115106	-1.41677
lnc-ZFAT-11:1	-1.30002	2.111862	-4.85221	0.003634	0.115106	-1.41746
lnc-URB1-2:1	1.324019	2.139978	4.852068	0.003635	0.115106	-1.4176
NONHSAT205859.1	1.563241	1.890681	4.849764	0.003643	0.115165	-1.41986
lnc-TFPI2-2:1	1.168617	4.455551	4.849303	0.003644	0.115165	-1.42031
NONHSAT197629.1	1.467494	2.131939	4.84919	0.003645	0.115165	-1.42042
lnc-EEF2-3:1	1.216576	1.623476	4.848876	0.003646	0.115165	-1.42073
ENST00000588469.1	1.930259	2.582268	4.84487	0.00366	0.115282	-1.42466
T367518	1.596698	2.061854	4.844697	0.00366	0.115282	-1.42483
ENST00000665335.1	-1.81474	3.386763	-4.84135	0.003672	0.115373	-1.42811
lnc-RXRA-3:1	-1.21676	1.794011	-4.84134	0.003672	0.115373	-1.42812
lnc-PARVB-3:1	-1.10393	7.501766	-4.83885	0.00368	0.115537	-1.43056
NONHSAT190976.1	-1.2298	3.422827	-4.83647	0.003689	0.115727	-1.43291
ENST00000550309.1	-1.13794	1.729599	-4.83052	0.00371	0.116019	-1.43875

NONHSAT192184.1	1.034464	1.715155	4.82998	0.003712	0.116019	-1.43928
NONHSAT204997.1	-1.34206	2.651804	-4.82954	0.003713	0.116019	-1.43971
MSTRG.26388.1	1.218068	1.736865	4.828075	0.003718	0.116144	-1.44115
ENST00000518031.1	1.056607	2.422975	4.826657	0.003723	0.116264	-1.44255
MSTRG.15382.1	1.153998	4.80962	4.824696	0.00373	0.116336	-1.44447
NONHSAT221171.1	-1.30844	2.435924	-4.82242	0.003738	0.116365	-1.44671
NONHSAT189294.1	-1.01996	7.719239	-4.82058	0.003745	0.116365	-1.44852
MSTRG.31549.1	1.350251	1.704165	4.815436	0.003763	0.11656	-1.45359
lnc-F7-2:1	-1.61401	2.253868	-4.81524	0.003764	0.11656	-1.45378
NONHSAT202874.1	2.40272	3.209332	4.814304	0.003767	0.116598	-1.45471
lnc-FAM13B-3:1	-1.01494	6.793683	-4.81026	0.003782	0.116642	-1.4587
lnc-HPCAL1-2:1	1.244654	3.813765	4.809639	0.003784	0.116642	-1.45931
lnc-PITRM1-5:4	1.117653	7.282766	4.809453	0.003785	0.116642	-1.45949
lnc-BRF1-73:1	1.023505	3.811774	4.808472	0.003788	0.116642	-1.46046
MSTRG.13790.1	-1.36275	2.092638	-4.80834	0.003789	0.116642	-1.46058
NONHSAT208879.1	1.295787	1.709767	4.808336	0.003789	0.116642	-1.46059
lnc-RBM46-1:1	-1.00494	8.771265	-4.80597	0.003797	0.1167	-1.46293
lnc-ICA1-1:9	-1.05726	3.052135	-4.80557	0.003799	0.1167	-1.46332
NONHSAT190397.1	-1.01735	8.97663	-4.80121	0.003814	0.116829	-1.46763
lnc-PLEC-4:1	1.275319	1.976784	4.799466	0.003821	0.116916	-1.46935
lnc-ABCA8-3:1	-1.72745	2.935849	-4.79621	0.003832	0.117172	-1.47257
NONHSAT190483.1	-1.14915	1.984329	-4.79512	0.003836	0.117224	-1.47364
MSTRG.55394.1	-1.02727	5.931586	-4.7941	0.00384	0.117224	-1.47465
NONHSAT221818.1	1.342645	1.862509	4.793285	0.003843	0.117224	-1.47546
lnc-LIMS4-1:3	1.010048	9.100538	4.790889	0.003852	0.117268	-1.47783
MSTRG.33755.2	-1.31498	2.311846	-4.79027	0.003854	0.117268	-1.47844
lnc-MED4-7:1	-1.0509	10.41991	-4.78908	0.003859	0.117317	-1.47962
NONHSAT192231.1	1.527182	1.817026	4.788117	0.003862	0.117367	-1.48057
ENST00000671359.1	2.059097	3.973365	4.786122	0.003869	0.117406	-1.48254
NR_104061	1.705812	2.626644	4.782383	0.003883	0.117406	-1.48625
lnc-HAAO-5:2	-1.2963	7.325922	-4.7815	0.003887	0.117406	-1.48713
MSTRG.32996.1	1.681443	2.289296	4.781198	0.003888	0.117406	-1.48742
NR_125885	1.014607	5.481399	4.780834	0.003889	0.117406	-1.48778
lnc-DEPDC1-3:1	-1.00696	7.461792	-4.77972	0.003893	0.117496	-1.48888
NONHSAT161385.1	1.04295	4.122138	4.777958	0.0039	0.11753	-1.49063
ENST00000418927.2	1.00758	4.504336	4.777504	0.003901	0.11753	-1.49108
T309277	1.582983	2.145432	4.777225	0.003902	0.11753	-1.49136
ENST00000455848.1	1.227139	3.365522	4.775661	0.003908	0.117565	-1.49291
lnc-SLC36A2-1:1	1.165412	3.731918	4.774897	0.003911	0.117615	-1.49367
lnc-S1PR1-6:1	-1.13365	11.2794	-4.77301	0.003918	0.117651	-1.49553
lnc-PLXDC2-9:1	-1.33769	4.935517	-4.77135	0.003924	0.117736	-1.49719
lnc-CEP57-3:1	-1.13486	9.442405	-4.77002	0.003929	0.117738	-1.49851
NONHSAT187230.1	1.178594	1.689073	4.769755	0.00393	0.117738	-1.49877
ENST00000592440.1	1.166512	4.190192	4.768263	0.003936	0.117871	-1.50025
NONHSAT209188.1	1.492427	3.873043	4.766467	0.003943	0.117946	-1.50203
lnc-OSBPL8-1:1	1.309327	4.48427	4.7654	0.003947	0.117946	-1.50309
lnc-NYAP2-7:1	2.429425	2.844508	4.765203	0.003948	0.117946	-1.50329
T265256	-2.18636	2.606315	-4.76512	0.003948	0.117946	-1.50337

lnc-PARM1-2:1	-1.24137	4.896087	-4.76254	0.003958	0.118014	-1.50593
NONHSAT168350.1	-1.4752	2.563921	-4.76234	0.003958	0.118014	-1.50613
lnc-NTSR2-2:5	1.479673	4.183997	4.760949	0.003964	0.118032	-1.50752
NONHSAT182462.1	-1.24765	1.969453	-4.75976	0.003968	0.118131	-1.50869
ENST00000558463.1	1.585951	1.846749	4.751088	0.004001	0.118521	-1.51733
T050385	-1.01095	5.94631	-4.74923	0.004008	0.118662	-1.51917
NONHSAT187411.1	-1.61056	4.3376	-4.74685	0.004017	0.118722	-1.52154
T013215	-1.0636	1.725102	-4.74657	0.004019	0.118722	-1.52183
lnc-FOXG1-12:2	-1.44955	2.35252	-4.74577	0.004022	0.118771	-1.52262
ENST00000638312.1	1.776989	2.026059	4.744094	0.004028	0.118795	-1.52429
ENST00000415202.1	-1.42946	2.128943	-4.74243	0.004035	0.118844	-1.52595
T363976	-1.361	7.61812	-4.7422	0.004035	0.118844	-1.52618
MSTRG.28246.1	-1.03967	5.094294	-4.74183	0.004037	0.118844	-1.52655
NONHSAT180730.1	-1.08822	9.015676	-4.74091	0.00404	0.118845	-1.52746
NONHSAT193687.1	1.005543	6.48338	4.735991	0.00406	0.119199	-1.53237
lnc-PRR29-5:1	-1.31978	1.868501	-4.73516	0.004063	0.119259	-1.5332
T282056	-1.14406	3.219769	-4.7319	0.004076	0.11939	-1.53645
NONHSAT201516.1	2.14348	4.503702	4.728964	0.004087	0.119484	-1.53939
MSTRG.33538.1	1.223759	3.375292	4.7269	0.004095	0.119499	-1.54145
NONHSAT184740.1	-1.10627	3.452924	-4.72555	0.0041	0.119499	-1.5428
MSTRG.17877.2	-1.61201	2.201216	-4.72211	0.004114	0.119669	-1.54624
MSTRG.5890.1	-1.91316	2.084856	-4.72176	0.004115	0.119669	-1.54659
NONHSAT210367.1	1.59489	2.358541	4.720703	0.00412	0.119691	-1.54765
MSTRG.53837.1	1.814599	2.332173	4.720077	0.004122	0.119694	-1.54827
lnc-TNFAIP8L1-3:1	-1.00056	11.25064	-4.71937	0.004125	0.119694	-1.54898
ENST00000419944.1	2.21333	2.591496	4.715126	0.004142	0.119938	-1.55323
NONHSAT207890.1	1.229732	3.983156	4.712037	0.004154	0.120094	-1.55632
NONHSAT176558.1	2.060944	2.923277	4.711505	0.004156	0.120094	-1.55686
ENST00000669532.1	-1.16534	3.007011	-4.71088	0.004159	0.120094	-1.55748
NONHSAT190511.1	2.156777	2.425446	4.710464	0.00416	0.120094	-1.5579
NONHSAT149915.1	1.266629	4.165632	4.710067	0.004162	0.120094	-1.5583
lnc-TSHZ2-7:1	-1.04031	5.030633	-4.70975	0.004163	0.120094	-1.55862
CYP4A22-AS1:1	-1.07203	3.10359	-4.70931	0.004165	0.120094	-1.55905
ENST00000420828.1	-1.43465	1.952667	-4.70754	0.004172	0.120096	-1.56083
ENST00000549291.1	1.097809	1.656913	4.706801	0.004175	0.120096	-1.56157
lnc-EXTL3-8:1	-1.03062	9.994665	-4.70666	0.004176	0.120096	-1.56171
lnc-ENKUR-5:1	1.752944	1.905005	4.705572	0.00418	0.120096	-1.5628
NONHSAT186932.1	1.735011	2.195341	4.703372	0.004189	0.120199	-1.56501
lnc-RTCA-2:1	1.357067	1.745732	4.700558	0.0042	0.120272	-1.56783
ENST00000669860.1	-1.2205	2.169282	-4.69978	0.004204	0.120272	-1.56861
ENST00000653126.1	-1.57456	4.93136	-4.69689	0.004215	0.120505	-1.57152
ENST00000534827.5	-1.36985	7.997369	-4.69662	0.004216	0.120505	-1.57179
lnc-RXFP4-5:1	-1.23849	2.787405	-4.69616	0.004218	0.120522	-1.57225
NONHSAT204799.1	1.522772	2.925041	4.695826	0.00422	0.120522	-1.57258
NONHSAT215556.1	1.385328	1.987492	4.695331	0.004222	0.120522	-1.57308
NONHSAT217428.1	1.752106	3.793622	4.695301	0.004222	0.120522	-1.57311
lnc-TAT-6:1	2.067868	3.01149	4.694776	0.004224	0.120526	-1.57364
lnc-DNAH8-1:1	1.601978	2.356224	4.694673	0.004224	0.120526	-1.57374

lnc-PEX16-1:1	1.03691	3.931338	4.691567	0.004237	0.120677	-1.57686
NONHSAT178542.1	1.58632	2.075577	4.690819	0.00424	0.120696	-1.57761
NONHSAT221748.1	1.784337	2.585859	4.690192	0.004243	0.120696	-1.57824
NONHSAT164695.1	-1.22413	4.100361	-4.68972	0.004245	0.120696	-1.57872
NONHSAT189667.1	1.103913	4.176266	4.689645	0.004245	0.120696	-1.57879
lnc-BOD1-6:1	-1.00106	8.841307	-4.68936	0.004246	0.120696	-1.57908
lnc-KIDINS220-12:2	1.237265	4.373216	4.687986	0.004252	0.120736	-1.58046
lnc-CCDC122-4:1	-1.44221	2.842666	-4.68713	0.004255	0.120736	-1.58133
ENST00000579752.1	1.15634	1.662422	4.686429	0.004258	0.120738	-1.58203
lnc-SFTA3-1:3	-1.0738	8.465377	-4.68575	0.004261	0.120758	-1.58272
NONHSAT216558.1	-1.2129	9.202753	-4.68558	0.004262	0.120758	-1.58288
NONHSAT167206.1	-1.20341	1.939925	-4.68539	0.004262	0.120758	-1.58308
NONHSAT223997.1	-1.3753	5.847673	-4.68481	0.004265	0.120785	-1.58365
lnc-VIRMA-3:1	-1.04192	3.284062	-4.68448	0.004266	0.120785	-1.58399
MSTRG.29187.1	1.38847	2.119944	4.683476	0.00427	0.12081	-1.585
MSTRG.64863.1	-1.59408	4.448543	-4.6823	0.004275	0.12081	-1.58619
ENST00000432706.1	1.311556	3.665835	4.681753	0.004277	0.12081	-1.58673
T119058	-1.30847	2.834977	-4.68108	0.00428	0.120817	-1.58741
ENST00000652083.2	-1.03065	1.670872	-4.67899	0.004289	0.120897	-1.58952
NONHSAT221766.1	-1.35814	5.798307	-4.67456	0.004307	0.12106	-1.59398
lnc-SF3B1-4:1	1.372367	2.02129	4.674058	0.004309	0.12106	-1.59448
NONHSAT212214.1	1.420911	7.346703	4.673365	0.004312	0.121083	-1.59518
lnc-MPPE1-15:1	-1.19931	6.536727	-4.67282	0.004315	0.121103	-1.59573
lnc-SH3RF2-6:1	-1.05821	6.812527	-4.67261	0.004315	0.121103	-1.59594
NONHSAT176788.1	1.471209	2.051763	4.668476	0.004333	0.121421	-1.60011
lnc-HTR3D-2:1	2.916101	6.007367	4.666142	0.004343	0.121431	-1.60247
MSTRG.9325.1	1.863788	2.460505	4.665725	0.004344	0.121431	-1.60289
lnc-CAPN13-3:1	-1.38556	2.924783	-4.66484	0.004348	0.121431	-1.60378
lnc-CIPC-4:1	1.641066	1.881941	4.664799	0.004348	0.121431	-1.60382
NONHSAT205586.1	1.734437	3.642231	4.664572	0.004349	0.121431	-1.60405
ENST00000519550.1	1.75946	2.243079	4.663772	0.004353	0.121436	-1.60486
NONHSAT150648.1	-1.19213	2.665705	-4.65946	0.004371	0.121698	-1.60922
MSTRG.8317.1	-1.05441	1.989826	-4.65898	0.004373	0.121698	-1.6097
MSTRG.17828.1	1.626562	2.023929	4.655418	0.004388	0.122053	-1.6133
lnc-FAM184A-2:1	-1.68136	2.846853	-4.65073	0.004408	0.122323	-1.61804
lnc-RAB6C-9:1	-1.68609	2.441103	-4.6501	0.004411	0.122323	-1.61867
ENST00000671647.1	-1.5123	2.118298	-4.65002	0.004411	0.122323	-1.61876
NONHSAT166694.1	-1.26154	2.081641	-4.64497	0.004433	0.122561	-1.62387
lnc-EDN2-2:7	2.566697	2.370652	4.644864	0.004433	0.122561	-1.62398
ENST00000623207.1	-1.37252	3.240645	-4.6416	0.004447	0.122712	-1.62728
ENST00000533812.6	1.556677	2.29901	4.63672	0.004468	0.122952	-1.63223
MSTRG.23692.1	1.904161	2.409225	4.633389	0.004483	0.123133	-1.63561
lnc-UCHL5-9:2	1.202073	2.023729	4.63266	0.004486	0.123135	-1.63635
NONHSAT153720.1	1.259293	4.120266	4.632528	0.004487	0.123135	-1.63648
lnc-SLC35G4-3:2	1.993536	3.003082	4.630608	0.004495	0.123299	-1.63843
NONHSAT174565.1	1.306417	2.343198	4.626316	0.004514	0.123446	-1.64278
NONHSAT201319.1	1.551876	1.894058	4.626261	0.004514	0.123446	-1.64284
NONHSAT188193.1	1.175059	2.351744	4.624585	0.004522	0.123522	-1.64454

NONHSAT176708.1	1.170419	2.02773	4.62276	0.00453	0.123674	-1.6464
lnc-CD163-3:1	1.179668	4.415744	4.621194	0.004537	0.123762	-1.64799
NONHSAT210513.1	-1.50531	3.107273	-4.62082	0.004538	0.123774	-1.64837
NONHSAT170176.1	-1.04752	4.881485	-4.62025	0.004541	0.12381	-1.64895
ENST00000501886.2	-1.60581	1.973448	-4.61984	0.004543	0.123825	-1.64936
lnc-CDKN1B-1:1	1.042631	2.531226	4.618785	0.004547	0.123886	-1.65044
lnc-CALM3-2:1	-1.2773	2.063928	-4.61777	0.004552	0.12389	-1.65147
lnc-ZNF704-2:1	-1.00091	5.382348	-4.61734	0.004554	0.12389	-1.65191
lnc-TMEM132B-19:1	-1.00113	1.652007	-4.61709	0.004555	0.12389	-1.65216
lnc-ACP5-3:1	1.028097	1.753102	4.616358	0.004558	0.123945	-1.65291
lnc-RIMBP2-7:1	-1.10689	10.46464	-4.61578	0.004561	0.12398	-1.65349
lnc-NRG2-4:1	-1.94557	3.205654	-4.61331	0.004572	0.12398	-1.65601
lnc-MBL2-1:1	-1.55851	2.049779	-4.61304	0.004573	0.12398	-1.65628
NONHSAT186640.1	1.506105	1.861056	4.612702	0.004574	0.123987	-1.65663
ENST00000524252.1	1.036131	5.471206	4.612427	0.004576	0.123987	-1.65691
ENST00000622160.1	1.136201	3.784302	4.608994	0.004591	0.124257	-1.6604
ENST00000667208.1	-1.28738	2.063503	-4.60537	0.004607	0.124545	-1.66409
NONHSAT190488.1	-1.31869	2.146242	-4.6034	0.004616	0.124636	-1.66609
ENST00000422042.1	-1.02438	4.095565	-4.60204	0.004622	0.124672	-1.66749
MSTRG.56866.1	-1.01341	4.285943	-4.60152	0.004625	0.12468	-1.66802
NONHSAT221788.1	1.193626	2.932719	4.599799	0.004632	0.124733	-1.66977
lnc-CDH3-3:1	1.815566	2.340352	4.599711	0.004633	0.124733	-1.66986
NONHSAT187149.1	1.139183	5.359278	4.598745	0.004637	0.124733	-1.67085
MSTRG.11503.1	1.246257	2.173861	4.597681	0.004642	0.124733	-1.67193
MSTRG.49105.1	-1.40055	3.088696	-4.59745	0.004643	0.124733	-1.67217
NONHSAT172982.1	1.05661	1.727722	4.595791	0.004651	0.124733	-1.67386
lnc-SCUBE3-2:1	-1.35988	1.994479	-4.59173	0.004669	0.12498	-1.678
NONHSAT167611.1	-2.2587	2.463156	-4.59084	0.004673	0.125007	-1.67892
T191967	-1.45113	1.91278	-4.59069	0.004674	0.125007	-1.67907
NONHSAT207916.1	1.525514	2.78656	4.590188	0.004676	0.125009	-1.67958
lnc-ZKSCAN2-7:1	-1.27794	10.44386	-4.59012	0.004676	0.125009	-1.67964
T276829	1.659606	2.881426	4.587124	0.00469	0.125164	-1.68271
lnc-HOOK1-6:1	1.485606	1.763723	4.586543	0.004693	0.125183	-1.6833
MSTRG.27914.1	2.285002	2.222483	4.584965	0.0047	0.125297	-1.68492
ENST00000428485.1	-1.25867	2.008998	-4.58399	0.004705	0.125331	-1.68591
NR_026755	-1.46901	3.596913	-4.58122	0.004718	0.125498	-1.68875
NONHSAT210569.1	1.066302	3.080041	4.580622	0.00472	0.125504	-1.68936
MSTRG.6336.1	-1.18276	3.77205	-4.58043	0.004721	0.125504	-1.68955
MSTRG.63542.1	1.345263	1.847778	4.578807	0.004729	0.125532	-1.69121
lnc-KLF4-15:2	1.005928	3.981177	4.576177	0.004741	0.125665	-1.69391
MSTRG.50194.7	2.308354	2.360553	4.575503	0.004744	0.125682	-1.6946
NONHSAT194231.1	-1.08059	4.502372	-4.57404	0.004751	0.125784	-1.69609
ENST00000419211.2	1.250767	3.719177	4.573878	0.004752	0.125784	-1.69626
ENST00000453866.1	-1.58698	2.418653	-4.57264	0.004757	0.125876	-1.69752
NONHSAT174775.1	1.106991	3.651923	4.571526	0.004763	0.125876	-1.69867
lnc-TMSB15A-6:1	-1.3355	2.66217	-4.56802	0.004779	0.125981	-1.70226
MSTRG.40922.1	1.121015	1.66474	4.56791	0.00478	0.125981	-1.70237
lnc-CCT5-14:1	-1.76528	3.013514	-4.56719	0.004783	0.125981	-1.70311

lnc-SERTM2-5:1	-1.06636	3.210309	-4.56679	0.004785	0.125981	-1.70352
NONHSAT156824.1	1.655726	1.94814	4.56603	0.004788	0.126003	-1.7043
lnc-FAM81B-1:1	1.293838	4.976912	4.559183	0.004821	0.126326	-1.71133
ENST00000647271.1	1.372653	3.069073	4.556998	0.004831	0.126494	-1.71357
lnc-UGT1A1-2:1	-1.80713	2.206133	-4.55558	0.004838	0.126522	-1.71503
ENST00000666655.1	1.914312	2.049681	4.55439	0.004844	0.126553	-1.71625
lnc-SBF2-1:2	1.106109	3.944852	4.553362	0.004848	0.126553	-1.7173
NONHSAT169054.1	1.025545	4.814912	4.549827	0.004865	0.126634	-1.72094
lnc-ZNF257-4:1	-1.05631	1.915735	-4.54939	0.004867	0.126654	-1.72139
MSTRG.16488.1	-1.7253	3.698303	-4.54634	0.004882	0.126654	-1.72452
ENST00000671045.1	-1.21648	2.025947	-4.54536	0.004887	0.126654	-1.72552
MSTRG.52951.1	1.255799	1.93003	4.544351	0.004892	0.126677	-1.72657
lnc-GKN2-1:1	1.184833	1.713591	4.541457	0.004906	0.126817	-1.72955
lnc-RAB1A-9:1	-1.42566	2.552774	-4.54108	0.004908	0.126817	-1.72993
ENST00000470427.1	-1.27917	5.157939	-4.53977	0.004914	0.126817	-1.73128
ENST00000431656.1	-1.39266	2.70403	-4.5395	0.004915	0.126817	-1.73156
lnc-SLC35C1-83:12	-1.25996	2.964843	-4.53926	0.004916	0.126817	-1.7318
MSTRG.45455.10	-1.30303	5.468396	-4.53899	0.004918	0.126817	-1.73209
ENST00000319682.2	1.898218	2.82668	4.535927	0.004933	0.126929	-1.73524
lnc-PP2D1-1:1	1.229279	4.044216	4.535463	0.004935	0.126929	-1.73572
lnc-CCNK-5:1	-1.26733	2.223974	-4.53499	0.004937	0.126929	-1.73621
ENST00000526131.1	1.703666	3.527517	4.534295	0.004941	0.126945	-1.73692
NONHSAT205868.1	1.349446	1.950568	4.533571	0.004944	0.126945	-1.73767
lnc-EGR3-7:1	-1.68191	4.535721	-4.53312	0.004946	0.126969	-1.73813
NONHSAT167621.1	1.810981	2.420432	4.529261	0.004965	0.127172	-1.74211
NONHSAT201203.1	-1.15602	2.656147	-4.52909	0.004966	0.127172	-1.74229
NR_038399	1.303884	2.835435	4.528948	0.004967	0.127172	-1.74243
ENST00000435111.1	-1.01761	9.273557	-4.52811	0.004971	0.127172	-1.7433
lnc-ORAOV1-3:3	1.693198	2.515112	4.527969	0.004971	0.127172	-1.74344
lnc-CXADR-7:1	2.029146	3.024064	4.526985	0.004976	0.127255	-1.74446
ENST00000654027.1	-1.2876	3.961029	-4.52556	0.004983	0.127403	-1.74593
NONHSAT211073.1	1.098509	4.383591	4.524056	0.004991	0.127448	-1.74748
NONHSAT177069.1	2.325938	3.05134	4.523257	0.004995	0.127448	-1.74831
NONHSAT208139.1	2.272467	2.836097	4.523151	0.004995	0.127448	-1.74842
MSTRG.31289.6	-1.01665	3.028163	-4.52147	0.005004	0.127566	-1.75015
lnc-PLCL2-11:1	1.947943	2.451543	4.520057	0.005011	0.127645	-1.75161
NONHSAT220956.1	-1.36142	3.036589	-4.51858	0.005018	0.127731	-1.75313
T190093	1.491649	2.084939	4.518285	0.005019	0.127731	-1.75344
NONHSAT191803.1	1.248904	3.639518	4.51809	0.00502	0.127731	-1.75364
lnc-CLEC19A-3:1	-1.09733	5.517658	-4.51782	0.005022	0.127731	-1.75392
lnc-DNAH5-4:1	1.179786	2.349297	4.516808	0.005027	0.127765	-1.75497
LINC00347:6	1.040392	1.96166	4.516283	0.005029	0.127789	-1.75551
lnc-MAGI1-2:1	1.168173	1.672941	4.515115	0.005035	0.127789	-1.75672
NONHSAT157329.1	1.153283	2.218856	4.514173	0.00504	0.127798	-1.75769
NONHSAT210220.1	1.277214	4.33016	4.508584	0.005068	0.12808	-1.76347
ENST00000320372.9	-1.57671	2.517364	-4.50702	0.005076	0.128171	-1.76509
lnc-ANKRD55-8:1	2.21295	2.705121	4.506624	0.005078	0.128189	-1.7655
lnc-PTBP2-12:2	-1.13314	7.724015	-4.50327	0.005095	0.128284	-1.76897

lnc-TIMM21-9:1	1.283527	1.791058	4.50194	0.005101	0.128284	-1.77035
lnc-MAGEB2-1:1	-1.35598	7.669247	-4.50098	0.005106	0.128284	-1.77134
lnc-RPL21-6:1	-1.056	3.743599	-4.4984	0.005119	0.128335	-1.77402
NONHSAT210587.1	-1.29435	2.2631	-4.49803	0.005121	0.128335	-1.7744
lnc-TMC6-7:1	3.328001	4.081536	4.497414	0.005124	0.128335	-1.77504
ENST00000561123.2	1.18241	2.668883	4.496949	0.005127	0.128335	-1.77552
T073551	1.313362	2.217932	4.496844	0.005127	0.128335	-1.77563
MSTRG.25122.1	-1.97227	2.675608	-4.49629	0.00513	0.128348	-1.77621
NONHSAT196277.1	1.498272	3.836394	4.492724	0.005148	0.128491	-1.7799
lnc-SGMS2-1:1	-1.58111	2.844533	-4.49111	0.005156	0.128491	-1.78157
lnc-UNC93A-3:1	-1.03393	2.827746	-4.49067	0.005159	0.128491	-1.78203
LINC01503:4	-1.13479	2.319491	-4.4904	0.00516	0.128491	-1.78231
T049511	1.011246	2.137849	4.48883	0.005168	0.128491	-1.78394
lnc-CD300C-1:1	-1.42637	7.576867	-4.4864	0.005181	0.128561	-1.78646
lnc-CCKAR-6:1	1.209552	4.170615	4.486132	0.005182	0.128561	-1.78674
lnc-SEL1L3-13:1	1.522029	2.099116	4.483135	0.005198	0.128827	-1.78985
NONHSAT157860.1	-1.17233	3.806332	-4.48165	0.005205	0.128891	-1.7914
lnc-PATE2-1:1	-1.01804	2.873187	-4.48057	0.005211	0.128935	-1.79252
ENST00000657726.1	-1.07243	1.669371	-4.47929	0.005217	0.128977	-1.79385
NONHSAT190182.1	1.018371	1.607254	4.479008	0.005219	0.128977	-1.79414
ENST00000663983.1	1.273615	2.77163	4.477282	0.005228	0.129096	-1.79594
NONHSAT179917.1	-1.30076	2.913714	-4.47584	0.005235	0.129217	-1.79743
ENST00000664386.1	1.749173	1.978049	4.47263	0.005252	0.129323	-1.80077
lnc-FAM3B-7:1	1.266794	6.41231	4.469997	0.005266	0.129505	-1.80352
MSTRG.7360.1	1.494002	2.861688	4.46804	0.005276	0.129566	-1.80555
ENST00000548488.1	-2.61903	5.80381	-4.46738	0.00528	0.129566	-1.80624
ENST00000607296.1	1.082594	4.934749	4.467327	0.00528	0.129566	-1.80629
MSTRG.12629.1	-1.68034	2.220256	-4.4665	0.005284	0.129586	-1.80715
lnc-ULBP2-25:1	-1.24877	2.08802	-4.46644	0.005285	0.129586	-1.80722
lnc-PGAP1-4:1	1.026847	5.155881	4.465988	0.005287	0.129613	-1.80769
NONHSAT215815.1	1.113771	2.074298	4.464027	0.005297	0.129772	-1.80973
NONHSAT153688.1	1.237517	1.794371	4.461182	0.005312	0.129983	-1.8127
NONHSAT173633.1	1.094092	2.322344	4.460925	0.005314	0.129985	-1.81297
ENST00000505567.1	-1.06499	1.969006	-4.45963	0.005321	0.130059	-1.81432
lnc-C4orf33-7:1	-1.05737	2.822167	-4.45735	0.005333	0.130292	-1.8167
lnc-ADAMTS5-6:1	1.776424	2.731878	4.456025	0.00534	0.130422	-1.81808
lnc-CLCC1-1:1	-1.01151	7.654671	-4.45434	0.005349	0.13055	-1.81984
lnc-AMELY-10:1	-1.34448	5.817304	-4.45415	0.00535	0.13055	-1.82003
NONHSAT169428.1	1.073915	1.661158	4.452613	0.005358	0.130589	-1.82164
NONHSAT213998.1	1.588192	2.163522	4.452184	0.00536	0.130589	-1.82208
NONHSAT187843.1	1.000034	1.907711	4.452116	0.005361	0.130589	-1.82216
lnc-NEMF-5:1	1.360062	2.109967	4.451717	0.005363	0.130589	-1.82257
NONHSAT195870.1	1.042876	3.042735	4.451211	0.005366	0.130589	-1.8231
NONHSAT191907.1	1.269978	3.7234	4.446121	0.005393	0.130966	-1.82842
NR_109894	1.316125	3.79687	4.445273	0.005398	0.130966	-1.8293
NONHSAT215675.1	1.355816	2.036939	4.445116	0.005398	0.130966	-1.82947
MSTRG.5976.1	1.985059	2.649515	4.441694	0.005417	0.131233	-1.83304
G2E3-AS1:12	-1.52988	2.30479	-4.44027	0.005425	0.131295	-1.83453

MSTRG.62878.1	-1.56575	2.648334	-4.43834	0.005435	0.131349	-1.83655
ENST00000557025.1	1.162942	2.204005	4.438286	0.005435	0.131349	-1.83661
NONHSAT218745.1	1.314286	3.555847	4.438163	0.005436	0.131349	-1.83674
ENST00000601280.1	-1.45544	2.145091	-4.43764	0.005439	0.131357	-1.83728
lnc-RAD23B-11:1	1.196865	1.933677	4.436997	0.005442	0.131357	-1.83796
lnc-OLFM1-2:1	1.772418	1.910252	4.432238	0.005468	0.131641	-1.84294
T236072	1.308062	1.979286	4.430413	0.005478	0.131707	-1.84485
ENST00000663385.1	-1.12137	1.753497	-4.43021	0.00548	0.131707	-1.84506
lnc-GDE1-4:1	-1.63137	3.432861	-4.42927	0.005485	0.131726	-1.84605
MSTRG.49773.1	-1.57619	2.826213	-4.42885	0.005487	0.131729	-1.84648
NONHSAT201899.1	1.490602	4.363607	4.427196	0.005496	0.131845	-1.84822
lnc-LIPC-4:1	-1.04525	7.118168	-4.42629	0.005501	0.131845	-1.84917
T080916	-1.37113	1.870656	-4.42622	0.005502	0.131845	-1.84924
NONHSAT175857.1	1.302423	1.918039	4.42583	0.005504	0.131845	-1.84965
lnc-TARSL2-3:1	1.173507	3.756832	4.420797	0.005532	0.131845	-1.85493
NONHSAT218609.1	1.105618	4.484562	4.420065	0.005536	0.131845	-1.8557
MSTRG.60768.1	1.600451	2.4734	4.418786	0.005543	0.131873	-1.85704
NONHSAT156781.1	1.052893	4.622002	4.416589	0.005555	0.131978	-1.85935
NONHSAT222661.1	1.689985	3.027067	4.415815	0.005559	0.132049	-1.86016
lnc-FER-18:2	1.317202	3.223739	4.41413	0.005569	0.132149	-1.86193
lnc-NXPH1-3:1	-1.07593	1.965582	-4.41346	0.005572	0.132151	-1.86263
lnc-UQCRFS1-6:1	1.409416	2.441485	4.412864	0.005576	0.132151	-1.86326
lnc-RLF-4:1	-1.08857	5.509698	-4.41188	0.005581	0.132151	-1.8643
NONHSAT164739.1	1.327198	1.755273	4.409923	0.005592	0.132343	-1.86635
NONHSAT211997.1	-1.63469	2.243881	-4.40906	0.005597	0.132343	-1.86725
lnc-CYTL1-5:1	-1.48477	2.430601	-4.4087	0.005599	0.132343	-1.86763
lnc-MYT1L-1:4	2.096179	2.894152	4.405993	0.005614	0.132393	-1.87048
lnc-NR3C1-3:1	-1.52286	2.365461	-4.40506	0.00562	0.132455	-1.87146
lnc-OSBP-1:3	1.10471	4.495019	4.402687	0.005633	0.132614	-1.87395
NONHSAT214428.1	1.529467	5.286694	4.402155	0.005636	0.132626	-1.87451
MSTRG.18274.1	-1.19053	2.422994	-4.40044	0.005646	0.132678	-1.87632
NONHSAT201028.1	1.267219	1.691284	4.39976	0.00565	0.132678	-1.87703
NONHSAT196765.1	-1.09874	1.977965	-4.39941	0.005652	0.132678	-1.8774
ENST00000521572.1	1.150561	2.489362	4.398702	0.005656	0.132685	-1.87815
lnc-SLC7A3-4:1	-1.3837	4.250001	-4.3976	0.005662	0.132765	-1.87931
NONHSAT174534.1	-1.86448	1.924292	-4.39741	0.005663	0.132765	-1.87951
ENST00000456031.1	-1.51083	2.289877	-4.39573	0.005673	0.132905	-1.88128
T183635	-1.55907	2.59503	-4.39355	0.005685	0.133092	-1.88357
lnc-CCDC171-7:1	1.51924	2.022377	4.393352	0.005686	0.133092	-1.88378
NONHSAT191854.1	1.587388	2.262223	4.391811	0.005695	0.133144	-1.88541
NONHSAT205357.1	1.264095	3.274459	4.391339	0.005698	0.133163	-1.8859
NONHSAT196754.1	1.691639	2.367794	4.390985	0.0057	0.133163	-1.88628
lnc-ARF6-8:1	1.028492	3.515228	4.389567	0.005708	0.133207	-1.88777
NONHSAT177060.1	-1.34993	2.792546	-4.38953	0.005708	0.133207	-1.88781
NONHSAT214301.1	-1.17617	5.509047	-4.38903	0.005711	0.13324	-1.88834
NONHSAT221962.1	-1.07391	3.821684	-4.38825	0.005716	0.133283	-1.88916
MSTRG.54393.1	-1.03353	3.484866	-4.38491	0.005735	0.133423	-1.89269
lnc-RBBP6-4:4	1.13235	4.035058	4.383793	0.005741	0.133423	-1.89386

T047815	-1.15537	2.016114	-4.38332	0.005744	0.133423	-1.89436
MSTRG.18373.1	1.33551	2.394534	4.383153	0.005745	0.133423	-1.89454
NONHSAT173615.1	-1.28432	3.534785	-4.38298	0.005746	0.133423	-1.89471
ENST00000659759.1	-2.03201	2.411207	-4.38268	0.005748	0.133423	-1.89503
NONHSAT196209.1	-1.5935	2.463917	-4.38043	0.005761	0.133524	-1.89741
MSTRG.38746.2	-1.47114	2.040109	-4.37966	0.005765	0.133524	-1.89822
lnc-CHRNA5-3:3	1.167592	1.899548	4.379054	0.005769	0.133524	-1.89886
lnc-EVX2-4:2	1.072633	3.591688	4.377933	0.005775	0.133524	-1.90005
NONHSAT205359.1	-1.67263	3.830549	-4.375	0.005793	0.133686	-1.90315
NR_110156	-1.16244	2.245189	-4.37291	0.005805	0.133846	-1.90535
NONHSAT195823.1	1.48784	2.145305	4.371126	0.005815	0.133935	-1.90724
lnc-DIAPH3-12:1	-1.11992	8.346002	-4.36782	0.005835	0.134053	-1.91074
NONHSAT198648.1	-1.48735	4.171234	-4.36777	0.005835	0.134053	-1.91079
lnc-EEA1-6:1	1.327451	3.531279	4.367417	0.005837	0.134054	-1.91116
lnc-SEC61G-9:1	1.165145	1.641694	4.365691	0.005847	0.134054	-1.91299
NONHSAT160182.1	1.087421	3.098012	4.361685	0.005871	0.134304	-1.91723
ENST00000661878.1	1.362557	2.345623	4.361651	0.005871	0.134304	-1.91726
NONHSAT164786.1	1.178783	1.969541	4.360403	0.005879	0.134313	-1.91859
NONHSAT161669.1	1.023844	2.149934	4.359868	0.005882	0.134313	-1.91915
ENST00000670381.1	-1.49383	2.81597	-4.35983	0.005882	0.134313	-1.91919
lnc-BTK-1:1	-1.55601	4.972267	-4.35816	0.005892	0.134313	-1.92096
lnc-CLEC4M-3:1	-1.31388	5.401292	-4.35763	0.005895	0.134313	-1.92152
lnc-VRK1-10:3	1.171649	1.664279	4.357267	0.005897	0.134313	-1.92191
MSTRG.47511.1	-1.60236	2.193122	-4.35691	0.0059	0.134313	-1.92229
NONHSAT213854.1	1.259511	4.120228	4.356398	0.005903	0.134313	-1.92283
MSTRG.36348.1	1.11651	1.728198	4.355804	0.005906	0.134313	-1.92346
lnc-KCNB2-9:1	-1.6731	3.415249	-4.35468	0.005913	0.134313	-1.92465
lnc-CDH13-3:1	-1.0831	5.466379	-4.3536	0.005919	0.134395	-1.9258
NONHSAT224265.1	-1.11568	4.157873	-4.35251	0.005926	0.134455	-1.92695
lnc-TTLL4-2:1	1.318833	2.197774	4.351009	0.005935	0.134599	-1.92854
lnc-DDX60-2:1	-1.5234	2.661576	-4.35051	0.005938	0.134604	-1.92907
lnc-CA2-2:1	-1.3277	8.036047	-4.34944	0.005944	0.134604	-1.9302
ENST00000615570.1	-1.20387	3.728256	-4.34612	0.005964	0.134753	-1.93373
ENST00000506068.1	-1.10161	3.114467	-4.34484	0.005972	0.134855	-1.93509
lnc-PIK3R1-10:1	1.136145	3.38891	4.34471	0.005973	0.134855	-1.93523
MSTRG.46731.1	1.026115	3.554577	4.34377	0.005979	0.134893	-1.93622
lnc-SHISA5-3:1	1.641407	2.559435	4.343422	0.005981	0.134911	-1.93659
NONHSAT186627.1	1.474544	2.151226	4.342587	0.005986	0.134972	-1.93748
ENST00000668689.1	-1.71769	3.082343	-4.34216	0.005988	0.134972	-1.93793
ENST00000661265.1	-1.16428	1.817652	-4.3415	0.005992	0.134972	-1.93863
lnc-FBXO21-1:1	-1.37297	8.353805	-4.34108	0.005995	0.134972	-1.93908
NONHSAT186613.1	-1.32823	1.976465	-4.34099	0.005996	0.134972	-1.93917
NONHSAT154864.1	-1.63539	2.677161	-4.34097	0.005996	0.134972	-1.93919
NONHSAT197136.1	-1.52199	2.488746	-4.3398	0.006003	0.135075	-1.94044
MSTRG.53344.1	1.324107	3.427324	4.339332	0.006006	0.135109	-1.94094
NONHSAT210132.1	-1.2226	3.161941	-4.33908	0.006007	0.135114	-1.94121
MSTRG.29874.1	-1.19666	2.038033	-4.33696	0.00602	0.135124	-1.94346
lnc-AGAP2-1:4	1.585043	3.975694	4.336178	0.006025	0.135124	-1.94429

lnc-LUZP2-5:1	1.432469	1.808084	4.33616	0.006025	0.135124	-1.94431
lnc-MBOAT1-15:1	-1.32069	3.643275	-4.33559	0.006029	0.135124	-1.94491
NR_110139	1.008806	4.247814	4.331436	0.006054	0.135244	-1.94933
NONHSAT223699.1	1.014241	2.589248	4.329397	0.006067	0.135393	-1.9515
MSTRG.54890.1	1.016772	4.828959	4.328809	0.00607	0.135444	-1.95213
T270004	-1.37325	2.046455	-4.32725	0.00608	0.135489	-1.95379
ENST00000567305.1	-2.11678	3.984451	-4.32708	0.006081	0.135489	-1.95396
NONHSAT217260.1	-1.08358	3.361014	-4.32687	0.006082	0.135489	-1.95418
NONHSAT193970.1	-2.30198	2.999178	-4.32617	0.006087	0.135529	-1.95494
lnc-PLA1A-1:1	-1.34278	2.16879	-4.32569	0.00609	0.13554	-1.95545
lnc-ADAM17-1:1	-1.32533	4.051992	-4.3255	0.006091	0.13554	-1.95565
NONHSAT201596.1	-1.52616	9.132845	-4.32291	0.006107	0.135616	-1.95841
NONHSAT149371.1	1.096037	1.661646	4.322249	0.006111	0.135616	-1.95911
lnc-SIPA1L2-8:1	1.813868	4.247175	4.321577	0.006115	0.135616	-1.95983
NONHSAT155892.1	1.575985	2.287685	4.319413	0.006129	0.135736	-1.96213
NONHSAT160975.1	1.137999	4.105395	4.319383	0.006129	0.135736	-1.96216
lnc-OR2V2-2:1	1.409756	2.494133	4.319108	0.00613	0.135736	-1.96246
lnc-LGALS9B-12:1	-1.03408	2.582603	-4.31711	0.006143	0.135864	-1.96459
MSTRG.39322.1	1.743604	1.919147	4.313639	0.006165	0.136165	-1.96829
NONHSAT163442.1	-1.31587	2.921852	-4.31307	0.006168	0.13617	-1.9689
ZBED3-AS1:53	1.400882	4.115029	4.312084	0.006175	0.136175	-1.96995
ENST00000568730.1	-1.25947	2.187163	-4.31107	0.006181	0.13622	-1.97103
NONHSAT200395.1	1.189775	3.083518	4.310361	0.006185	0.13622	-1.97179
NONHSAT198305.1	2.377718	2.215323	4.308473	0.006197	0.136311	-1.9738
MSTRG.15357.1	-1.11182	1.708842	-4.30639	0.00621	0.136449	-1.97603
NONHSAT206240.1	-1.01921	3.500674	-4.30614	0.006212	0.136449	-1.97629
NONHSAT219485.1	-1.12138	6.354388	-4.30572	0.006215	0.136449	-1.97675
NONHSAT161592.1	-1.93899	3.808713	-4.30421	0.006224	0.136505	-1.97835
NONHSAT175658.1	-1.21044	1.931713	-4.29862	0.00626	0.136857	-1.98433
NONHSAT159195.1	1.716204	2.231518	4.29829	0.006262	0.136857	-1.98468
lnc-RGMB-4:1	-1.61571	2.815673	-4.29714	0.006269	0.136857	-1.98591
lnc-SDHAF4-8:1	-1.06472	10.34121	-4.29697	0.00627	0.136857	-1.9861
NONHSAT204515.1	1.046151	2.140784	4.296445	0.006274	0.136857	-1.98666
NONHSAT153990.1	-1.01669	1.646578	-4.29581	0.006278	0.136892	-1.98734
lnc-RNF20-5:1	-1.43101	2.921393	-4.29563	0.006279	0.136892	-1.98753
NONHSAT197499.1	-1.19236	4.962113	-4.29362	0.006292	0.136952	-1.98968
NONHSAT184025.1	1.962929	2.457914	4.292454	0.006299	0.13704	-1.99093
NONHSAT201959.1	1.525404	1.795661	4.291589	0.006305	0.137132	-1.99185
NONHSAT201004.1	-1.38434	2.358063	-4.29125	0.006307	0.13715	-1.99222
lnc-MPLKIP-5:4	1.270838	2.953507	4.289927	0.006316	0.137217	-1.99363
NONHSAT205228.1	1.944797	7.055808	4.286724	0.006336	0.13743	-1.99706
lnc-NR3C2-7:2	1.454779	1.802104	4.286297	0.006339	0.137461	-1.99752
NONHSAT148328.1	1.763306	1.982852	4.28433	0.006352	0.137505	-1.99963
lnc-CNTNAP2-7:1	1.609737	1.936531	4.283465	0.006358	0.137556	-2.00055
lnc-FSHB-9:1	1.186726	3.593284	4.283312	0.006359	0.137556	-2.00072
lnc-FAM178B-1:1	-1.36112	2.133791	-4.28297	0.006361	0.137575	-2.00108
MSTRG.54670.1	-1.14149	6.630232	-4.28014	0.006379	0.137915	-2.00412
lnc-PKDCC-1:1	1.287455	2.044293	4.279539	0.006383	0.137948	-2.00476

lnc-TAS2R1-12:1	-1.53962	3.171872	-4.27796	0.006394	0.137948	-2.00645
NONHSAT212977.1	1.526861	2.432911	4.277213	0.006398	0.137948	-2.00726
ENST00000512516.1	-1.36719	2.358179	-4.27694	0.0064	0.137948	-2.00755
NONHSAT153933.1	-1.59019	1.948981	-4.2766	0.006403	0.137948	-2.00792
NONHSAT176310.1	1.093135	4.333108	4.275725	0.006408	0.138007	-2.00885
ENST00000667942.1	2.337566	2.606098	4.275175	0.006412	0.138026	-2.00944
ENST00000648601.1	1.949116	3.650122	4.272563	0.006429	0.138219	-2.01225
ENST00000414377.1	1.539974	6.086554	4.271919	0.006433	0.138257	-2.01294
MSTRG.5523.2	1.434904	2.979321	4.270645	0.006442	0.138314	-2.01431
MSTRG.17485.6	-1.17288	2.80016	-4.27006	0.006446	0.138338	-2.01494
NONHSAT204618.1	1.45431	2.385149	4.268332	0.006457	0.138465	-2.01679
lnc-TARDBP-8:1	-1.22634	7.791071	-4.26738	0.006463	0.138529	-2.01781
T049236	1.261246	2.225946	4.266283	0.006471	0.138529	-2.01899
NONHSAT180294.1	1.879526	2.414752	4.265324	0.006477	0.138529	-2.02002
MSTRG.3292.1	1.003169	4.52849	4.262578	0.006495	0.138552	-2.02297
NR_125817	1.509721	2.895933	4.262055	0.006499	0.138552	-2.02354
lnc-TTPA-6:1	-1.00007	7.373822	-4.2619	0.0065	0.138552	-2.0237
lnc-MRPS22-1:13	-1.51735	2.363207	-4.26183	0.0065	0.138552	-2.02378
lnc-SLC25A36-1:1	-1.02424	3.01429	-4.26023	0.006511	0.13859	-2.0255
MSTRG.61221.1	2.198332	5.1531	4.259936	0.006513	0.138603	-2.02582
lnc-STX4-2:1	1.476519	2.032118	4.259719	0.006514	0.138604	-2.02605
NONHSAT211962.1	2.073017	2.351429	4.258822	0.00652	0.138637	-2.02701
NONHSAT182546.1	1.576358	4.168075	4.256141	0.006538	0.138822	-2.0299
T163079	1.056729	2.015209	4.25485	0.006547	0.13886	-2.03129
lnc-ITGAL-5:1	-1.69644	2.734821	-4.25484	0.006547	0.13886	-2.03129
NONHSAT221794.1	1.402373	1.816575	4.25426	0.006551	0.138904	-2.03192
lnc-CD180-7:1	-1.10425	2.477347	-4.25393	0.006553	0.138904	-2.03228
lnc-HIST1H2AI-1:8	-1.26176	4.852437	-4.25194	0.006567	0.13903	-2.03442
lnc-HTR5A-1:1	-1.281	2.124031	-4.25135	0.006571	0.13904	-2.03506
lnc-BLCAP-5:1	-1.00368	5.108508	-4.25134	0.006571	0.13904	-2.03507
lnc-TMEM135-9:1	1.46401	1.846843	4.247902	0.006594	0.139097	-2.03877
MSTRG.45968.1	1.083634	2.945438	4.247487	0.006597	0.139097	-2.03922
lnc-MFSD9-13:1	-1.07871	1.915808	-4.24726	0.006598	0.139097	-2.03946
ENST00000548779.1	-1.44561	4.97103	-4.24495	0.006614	0.139198	-2.04196
lnc-TTC5-2:2	-1.08986	3.034296	-4.24468	0.006616	0.139207	-2.04225
T284675	1.532493	2.107891	4.244455	0.006617	0.13921	-2.04249
NONHSAT190040.1	-1.28597	5.803015	-4.24392	0.006621	0.139228	-2.04306
MSTRG.28871.3	-1.60129	3.274991	-4.24308	0.006627	0.139262	-2.04397
T104965	1.391983	2.928214	4.242601	0.00663	0.139302	-2.04449
T360844	1.482468	2.069937	4.240651	0.006643	0.139509	-2.04659
lnc-RGS5-2:3	1.141619	1.674543	4.239069	0.006654	0.139566	-2.0483
NONHSAT195538.1	1.43063	3.506912	4.238826	0.006656	0.139566	-2.04856
lnc-REL-6:3	-1.18419	3.206483	-4.23832	0.006659	0.139566	-2.04911
lnc-RALB-7:1	-1.27139	11.24407	-4.2376	0.006664	0.139583	-2.04988
lnc-CAPS2-4:2	-1.85227	2.84795	-4.23552	0.006678	0.13968	-2.05213
MSTRG.26065.2	-1.15994	2.653573	-4.23532	0.00668	0.13968	-2.05234
ENST00000561538.1	1.423089	2.060689	4.234017	0.006689	0.139731	-2.05375
lnc-COL9A2-1:4	-1.14811	1.871072	-4.23253	0.006699	0.139827	-2.05535

NONHSAT167631.1	1.962941	3.327458	4.229854	0.006718	0.139995	-2.05825
lnc-TEX10-4:1	1.248788	3.182783	4.227528	0.006734	0.140132	-2.06076
NONHSAT210164.1	1.004318	1.53089	4.225129	0.00675	0.140247	-2.06336
T047559	1.837094	1.988805	4.22458	0.006754	0.140247	-2.06395
NONHSAT148748.1	1.413203	2.694635	4.224033	0.006758	0.140269	-2.06454
NONHSAT152603.1	1.452976	2.142565	4.222868	0.006766	0.140341	-2.0658
MSTRG.44930.1	-1.26223	3.125659	-4.22273	0.006767	0.140341	-2.06595
ENST00000668724.1	1.57132	4.244832	4.221275	0.006777	0.140354	-2.06753
NONHSAT187427.1	-1.68872	2.626136	-4.21995	0.006787	0.140362	-2.06896
NONHSAT163254.1	-1.1783	4.260642	-4.21981	0.006788	0.140362	-2.06911
MSTRG.60669.1	-1.16938	1.806461	-4.21816	0.006799	0.140457	-2.0709
NONHSAT202146.1	-1.0224	3.367955	-4.21664	0.00681	0.140563	-2.07255
ENST00000654316.1	1.636532	2.791475	4.216213	0.006813	0.140596	-2.07301
lnc-LRMDA-14:1	1.72525	2.347939	4.215528	0.006818	0.140603	-2.07375
lnc-SPACA3-1:2	-1.88498	3.1081	-4.21474	0.006823	0.140603	-2.0746
NONHSAT190073.1	-1.75363	2.958726	-4.21463	0.006824	0.140603	-2.07472
MSTRG.59562.1	1.395644	1.8764	4.214291	0.006826	0.140603	-2.07509
ENST00000607322.1	-1.09509	3.980767	-4.21266	0.006838	0.140603	-2.07685
NONHSAT197730.1	-1.04069	1.774271	-4.21201	0.006843	0.140603	-2.07756
NONHSAT216038.1	1.352362	1.768557	4.211257	0.006848	0.140603	-2.07838
NONHSAT202743.1	-1.58305	2.075548	-4.211	0.00685	0.140603	-2.07866
NONHSAT218904.1	2.01809	4.805467	4.210619	0.006852	0.140606	-2.07907
lnc-PLPP3-4:1	-1.20561	4.281711	-4.20822	0.006869	0.140759	-2.08167
MSTRG.23145.1	-1.41158	2.486862	-4.20806	0.006871	0.140759	-2.08184
MSTRG.202.2	-1.52354	9.092033	-4.20734	0.006876	0.140764	-2.08263
T187106	-1.08759	3.646505	-4.20716	0.006877	0.140764	-2.08282
lnc-STON1-GTF2A1L-2:1	-1.20674	2.577724	-4.20343	0.006904	0.141081	-2.08686
NONHSAT210315.1	1.009141	1.580968	4.20258	0.00691	0.141148	-2.08779
ENST00000653118.1	1.110104	5.567122	4.200245	0.006926	0.141289	-2.09032
MSTRG.37935.1	1.418252	4.070616	4.199656	0.006931	0.141318	-2.09096
MSTRG.36086.1	-1.03991	4.121084	-4.1977	0.006945	0.14152	-2.09309
NONHSAT179711.1	1.009221	1.627955	4.197486	0.006946	0.141522	-2.09332
ENST00000439299.1	-1.20109	2.003793	-4.1956	0.00696	0.141709	-2.09537
NONHSAT154949.1	1.535063	2.946382	4.195054	0.006964	0.141709	-2.09596
lnc-ANXA2R-6:1	2.075301	4.849982	4.193672	0.006974	0.141709	-2.09746
lnc-FRG2C-6:1	-1.10833	2.670306	-4.19338	0.006976	0.141709	-2.09777
ENST00000414790.6	1.233394	6.418453	4.192943	0.006979	0.141732	-2.09825
ENST00000426275.1	-1.43229	2.464331	-4.18715	0.007021	0.142156	-2.10455
lnc-FAT3-6:1	-1.32904	2.905226	-4.18605	0.007029	0.142226	-2.10574
MSTRG.46298.2	-1.07018	5.451954	-4.18549	0.007033	0.142226	-2.10636
NONHSAT169947.1	-1.28817	2.906233	-4.18522	0.007035	0.142226	-2.10665
T103396	1.367379	1.963513	4.185128	0.007036	0.142226	-2.10675
MSTRG.45766.1	1.655994	2.762885	4.184319	0.007042	0.142274	-2.10763
lnc-PRKX-7:1	-1.09862	1.904082	-4.18281	0.007053	0.142379	-2.10927
lnc-P2RY2-11:1	-1.50057	4.948472	-4.18274	0.007053	0.142379	-2.10934
NONHSAT171316.1	1.346697	3.157021	4.177605	0.007091	0.142511	-2.11494
lnc-MC3R-2:1	1.145759	3.278347	4.176266	0.007101	0.142541	-2.1164

ENST00000670510.1	1.89442	2.437181	4.175341	0.007108	0.142621	-2.11741
ENST00000595059.1	-1.07621	2.058494	-4.17469	0.007113	0.142662	-2.11812
MSTRG.58082.1	-1.21239	4.243423	-4.17436	0.007115	0.142682	-2.11848
NONHSAT153655.1	1.144047	1.873476	4.174147	0.007116	0.142685	-2.11871
lnc-ODF1-4:1	-1.14711	5.764951	-4.17236	0.00713	0.142735	-2.12066
NONHSAT154567.1	1.266581	2.021437	4.172299	0.00713	0.142735	-2.12072
lnc-TRMT13-2:1	-1.67576	2.852751	-4.17228	0.00713	0.142735	-2.12074
lnc-FBXO45-1:1	-1.55034	6.121621	-4.17115	0.007139	0.142786	-2.12197
NONHSAT171875.1	2.25326	3.285918	4.170549	0.007143	0.142786	-2.12263
NONHSAT209022.1	-1.00006	4.251147	-4.17019	0.007146	0.142786	-2.12302
ENST00000662862.1	-1.07509	3.517642	-4.17014	0.007146	0.142786	-2.12308
NONHSAT171291.1	1.135244	1.654484	4.166547	0.007173	0.143134	-2.127
ENST00000423838.1	1.505744	2.766615	4.165368	0.007182	0.143207	-2.12828
NONHSAT161597.1	1.277464	2.539125	4.164261	0.00719	0.143277	-2.12949
MSTRG.19480.1	-1.30665	3.791249	-4.16403	0.007192	0.143277	-2.12974
lnc-DNAJC6-3:1	1.321498	4.355149	4.160583	0.007218	0.143459	-2.13351
NONHSAT189670.1	-1.13973	3.185071	-4.15997	0.007222	0.143472	-2.13418
lnc-DRD5-29:8	1.081426	2.038028	4.159561	0.007225	0.143472	-2.13462
MSTRG.7814.1	1.43921	1.820479	4.159542	0.007225	0.143472	-2.13465
lnc-MFSD8-9:1	2.085244	2.337278	4.158801	0.007231	0.143498	-2.13545
NONHSAT164920.1	-1.52548	2.615908	-4.15849	0.007233	0.143516	-2.1358
NONHSAT212969.1	1.502165	1.77617	4.157984	0.007237	0.143535	-2.13635
MSTRG.42808.1	-1.04991	3.262092	-4.15615	0.007251	0.143664	-2.13836
MSTRG.69484.1	-2.19383	2.663725	-4.15604	0.007252	0.143664	-2.13847
MSTRG.43113.1	2.305336	2.976898	4.155063	0.007259	0.143664	-2.13954
NONHSAT198001.1	1.747447	4.724914	4.153555	0.007271	0.143755	-2.14119
lnc-BCL7A-3:1	1.470309	5.217086	4.153501	0.007271	0.143755	-2.14125
lnc-NDST3-10:1	-1.52772	2.077232	-4.15242	0.007279	0.143832	-2.14242
NONHSAT218255.1	-1.19994	2.038123	-4.15183	0.007284	0.143868	-2.14308
lnc-CPT1A-3:1	1.832077	3.826238	4.1489	0.007306	0.143967	-2.14628
MSTRG.61317.1	1.057779	1.553354	4.148608	0.007308	0.143983	-2.1466
ENST00000504874.1	-1.31324	2.482806	-4.14753	0.007317	0.144004	-2.14778
MSTRG.37964.1	1.486282	4.765693	4.146301	0.007326	0.144011	-2.14912
MSTRG.21378.2	1.502777	2.347397	4.143354	0.007348	0.144267	-2.15235
NONHSAT164410.1	-1.04236	1.674707	-4.1416	0.007362	0.144335	-2.15428
NONHSAT172127.1	1.458802	1.885006	4.14014	0.007373	0.144428	-2.15587
lnc-FGF10-4:1	1.152879	2.412392	4.14005	0.007374	0.144428	-2.15597
ENST00000609581.1	-1.0345	9.790566	-4.13848	0.007386	0.144496	-2.15769
NONHSAT164819.1	1.318393	1.942979	4.134498	0.007417	0.144699	-2.16205
NONHSAT212095.1	2.113678	2.303769	4.133887	0.007422	0.144718	-2.16273
NONHSAT200324.1	1.248611	7.025876	4.132932	0.007429	0.144751	-2.16377
ENST00000569778.1	1.413832	3.573185	4.132637	0.007431	0.144751	-2.1641
lnc-CDY1-19:1	1.271409	1.738712	4.132351	0.007433	0.144751	-2.16441
lnc-NSMCE4A-2:1	-1.02464	9.061708	-4.13004	0.007451	0.14492	-2.16694
ENST00000537346.1	1.046284	3.572849	4.13003	0.007452	0.14492	-2.16696
NONHSAT187835.1	1.733328	4.128408	4.129842	0.007453	0.14492	-2.16716
NONHSAT206025.1	-1.46056	1.909707	-4.12959	0.007455	0.14492	-2.16744
lnc-CACYBP-3:1	-1.30775	2.337181	-4.12897	0.00746	0.144956	-2.16811

MSTRG.65491.2	-1.17846	2.260814	-4.12804	0.007467	0.145017	-2.16915
ENST00000574471.1	-1.13728	3.955385	-4.12488	0.007492	0.145135	-2.17261
MSTRG.12265.1	1.385816	2.128166	4.124483	0.007495	0.145135	-2.17305
lnc-PAQR9-7:1	-1.45495	4.636059	-4.12304	0.007506	0.14519	-2.17463
NONHSAT222629.1	1.687346	2.642021	4.121612	0.007517	0.145293	-2.1762
NONHSAT171343.1	-1.38839	2.839716	-4.1181	0.007545	0.14554	-2.18006
lnc-OR6B1-1:1	1.97299	2.845341	4.116005	0.007562	0.145674	-2.18236
NONHSAT159043.1	-1.16621	6.184128	-4.1157	0.007564	0.145687	-2.18269
NONHSAT219425.1	1.132337	1.827007	4.114973	0.00757	0.145693	-2.1835
NONHSAT185890.1	-1.62174	5.403617	-4.10976	0.007611	0.145943	-2.18923
NONHSAT180797.1	1.0948	1.651226	4.109504	0.007613	0.145943	-2.18951
ENST00000457975.2	1.184587	1.64115	4.109478	0.007614	0.145943	-2.18954
lnc-GTDC1-21:1	-1.03291	4.417669	-4.10754	0.007629	0.146093	-2.19168
NONHSAT219711.1	1.197004	3.899484	4.107458	0.00763	0.146093	-2.19177
ENST00000655237.1	-1.14635	2.844274	-4.10114	0.007681	0.146591	-2.19873
ENST00000428646.1	1.081101	6.977787	4.09725	0.007712	0.146744	-2.20302
lnc-NTSR1-1:2	-1.28935	3.624061	-4.09712	0.007713	0.146744	-2.20316
T157393	1.714965	2.840146	4.097029	0.007714	0.146744	-2.20326
NONHSAT200032.1	-1.49236	2.028036	-4.09679	0.007716	0.146744	-2.20352
NONHSAT167071.1	-1.5115	2.090477	-4.09599	0.007722	0.146744	-2.2044
NONHSAT209003.1	1.753307	2.276355	4.095046	0.00773	0.146821	-2.20545
lnc-BOLA3-5:2	1.308731	2.202485	4.09482	0.007732	0.146821	-2.2057
ENST00000578977.1	-1.18023	1.808682	-4.0938	0.00774	0.146865	-2.20682
lnc-STRADB-2:1	-1.22096	2.251599	-4.0926	0.00775	0.146912	-2.20814
NONHSAT197432.1	1.247057	1.740917	4.089385	0.007776	0.14694	-2.2117
NONHSAT177961.1	1.10519	1.584859	4.088205	0.007786	0.147068	-2.213
NONHSAT163085.1	1.628938	2.375666	4.08797	0.007788	0.147072	-2.21326
lnc-ALCAM-8:7	1.604166	5.501169	4.083932	0.007821	0.147324	-2.21772
NONHSAT220969.1	-1.06846	4.156682	-4.08205	0.007836	0.147443	-2.21979
lnc-ELP3-1:1	-1.11329	4.016344	-4.0809	0.007846	0.147512	-2.22107
NONHSAT224258.1	-1.30044	3.647344	-4.08007	0.007853	0.147532	-2.22199
ENST00000566747.1	1.198907	4.609321	4.076463	0.007883	0.147741	-2.22598
NONHSAT206246.1	-1.09594	1.767006	-4.07446	0.007899	0.147907	-2.22819
lnc-AKR1C2-10:1	-1.01639	5.281159	-4.0744	0.0079	0.147907	-2.22826
lnc-NDST4-1:1	-1.6906	2.416405	-4.0737	0.007906	0.147918	-2.22903
NONHSAT195170.1	1.512668	3.682856	4.072951	0.007912	0.14796	-2.22986
lnc-HELQ-1:1	-1.83427	3.733135	-4.07273	0.007914	0.14796	-2.2301
ENST00000658561.1	1.037768	1.538718	4.068898	0.007946	0.148346	-2.23435
NONHSAT157123.1	-1.3817	3.414475	-4.06621	0.007968	0.148368	-2.23732
T080141	-1.22094	3.956803	-4.06582	0.007972	0.148368	-2.23776
lnc-SLC20A1-3:1	-1.05698	2.016048	-4.06568	0.007973	0.148368	-2.23792
lnc-RINT1-4:1	1.22295	3.943493	4.065005	0.007979	0.148368	-2.23866
lnc-THBD-1:5	-1.01654	3.77518	-4.06442	0.007983	0.148368	-2.23931
NONHSAT223999.1	1.691232	3.225536	4.064145	0.007986	0.148368	-2.23962
lnc-OGT-9:1	1.217137	3.02465	4.063643	0.00799	0.148368	-2.24017
T127833	-1.076	1.655858	-4.06341	0.007992	0.148368	-2.24043
ENST00000508815.1	-1.5284	4.115972	-4.06329	0.007993	0.148368	-2.24056
ENST00000451697.1	1.548964	3.13967	4.06298	0.007996	0.148368	-2.24091

ENST00000419190.2	1.311832	3.188634	4.062785	0.007997	0.148368	-2.24112
lnc-MPPE1-1:6	-1.18617	5.021481	-4.06248	0.008	0.148368	-2.24146
NONHSAT162508.1	1.273793	5.03706	4.062127	0.008003	0.148387	-2.24185
T063599	-1.10297	4.149517	-4.06124	0.00801	0.148459	-2.24284
ENST00000442072.1	1.021165	3.966899	4.061149	0.008011	0.148459	-2.24294
NR_125920	1.398388	5.305905	4.060398	0.008017	0.148471	-2.24377
lnc-TRIM38-6:1	-1.26289	2.522142	-4.0599	0.008022	0.148479	-2.24432
MSTRG.5858.1	-1.12489	4.130036	-4.05871	0.008032	0.148524	-2.24565
NONHSAT178389.1	1.300594	1.794533	4.058245	0.008036	0.14856	-2.24616
NONHSAT202409.1	1.226512	3.289815	4.057571	0.008041	0.148613	-2.24691
ENST00000443018.2	1.160058	1.968314	4.052907	0.008081	0.148849	-2.25208
MSTRG.62784.1	-1.87438	2.784357	-4.05092	0.008098	0.148954	-2.25429
MSTRG.1287.1	-1.45489	2.589965	-4.05044	0.008102	0.148954	-2.25482
lnc-C9orf50-1:2	2.086806	3.106692	4.050141	0.008105	0.148954	-2.25515
ENST00000665400.1	-1.19605	4.9828	-4.04992	0.008107	0.148954	-2.2554
MSTRG.42584.1	1.513561	1.999785	4.049828	0.008108	0.148954	-2.2555
lnc-MAP10-1:1	1.976392	2.904531	4.049346	0.008112	0.148954	-2.25604
lnc-ARMCX1-1:4	1.029218	4.513344	4.048886	0.008116	0.148999	-2.25655
NONHSAT218072.1	-1.01184	1.896317	-4.04674	0.008134	0.14917	-2.25893
lnc-RTN2-2:1	1.014948	3.041539	4.046091	0.00814	0.14917	-2.25965
MSTRG.46411.2	1.307797	1.812276	4.045659	0.008143	0.149182	-2.26013
lnc-IRX3-80:23	-1.35799	7.93427	-4.04194	0.008176	0.149393	-2.26427
ENST00000427471.1	-1.264	2.452527	-4.04006	0.008192	0.149393	-2.26636
lnc-DTL-2:7	1.377204	1.873016	4.039779	0.008194	0.149393	-2.26667
ENST00000439207.1	-1.27745	6.074744	-4.03911	0.0082	0.149393	-2.26741
lnc-HNRNPA2B1-16:5	-1.42643	2.84122	-4.03899	0.008201	0.149393	-2.26755
NONHSAT221735.1	2.01676	2.664347	4.037978	0.00821	0.149437	-2.26868
NONHSAT205559.1	-1.70701	2.527081	-4.0379	0.008211	0.149437	-2.26876
NONHSAT197283.1	-1.53443	2.455795	-4.03481	0.008238	0.149496	-2.27221
lnc-CHIC1-9:1	1.659198	2.91855	4.03449	0.00824	0.149496	-2.27256
lnc-SCP2D1-3:1	-2.05538	2.617216	-4.0343	0.008242	0.149496	-2.27277
lnc-LSM3-4:1	1.5124	2.966097	4.03356	0.008248	0.149559	-2.27359
lnc-CWC27-7:1	1.360196	2.445891	4.032043	0.008262	0.149719	-2.27528
NONHSAT201540.1	1.139708	5.118718	4.0318	0.008264	0.149731	-2.27555
NONHSAT180789.1	1.327045	2.085549	4.030674	0.008274	0.149775	-2.27681
NONHSAT195388.1	1.166083	3.794516	4.029745	0.008282	0.149796	-2.27784
NONHSAT223918.1	-1.24966	3.260903	-4.02946	0.008284	0.149796	-2.27816
lnc-CALB2-1:3	-1.67553	2.477367	-4.02825	0.008295	0.149796	-2.27951
MSTRG.42772.3	-1.65669	2.8209	-4.02752	0.008301	0.149835	-2.28032
NONHSAT197039.1	-1.41553	2.353622	-4.02705	0.008306	0.149835	-2.28085
NONHSAT170342.1	-1.54128	2.168561	-4.02625	0.008313	0.149835	-2.28174
NONHSAT189955.1	2.098666	2.693077	4.02578	0.008317	0.149883	-2.28226
ENST00000666543.1	-1.83879	2.072961	-4.02355	0.008337	0.150103	-2.28474
NONHSAT192777.1	1.373273	1.905385	4.023187	0.00834	0.150135	-2.28515
NONHSAT181208.1	1.555146	1.88525	4.022929	0.008342	0.150149	-2.28544
NONHSAT154399.1	-1.22822	12.41958	-4.0209	0.00836	0.150339	-2.2877
MSTRG.43901.1	-1.01436	1.617814	-4.01915	0.008376	0.150403	-2.28965
lnc-NAA35-9:1	1.887553	2.649925	4.018659	0.00838	0.150429	-2.2902

MSTRG.49698.1	1.230527	2.311535	4.017153	0.008393	0.150536	-2.29188
lnc-HLX-2:1	-1.03167	8.063066	-4.01673	0.008397	0.150574	-2.29236
ENST00000660399.1	-1.09907	3.408883	-4.01616	0.008402	0.150616	-2.293
ENST00000546770.1	1.857	4.535884	4.014927	0.008413	0.15065	-2.29437
NONHSAT204047.1	1.362929	2.765824	4.013699	0.008424	0.15076	-2.29574
lnc-CXCL12-5:1	-1.39171	3.526734	-4.01326	0.008428	0.15076	-2.29623
lnc-ETS2-11:1	-1.09186	5.659194	-4.00986	0.008459	0.151016	-2.30003
MSTRG.48174.1	1.033832	4.256608	4.008799	0.008468	0.1511	-2.30121
NONHSAT215265.1	-1.60391	4.333242	-4.00714	0.008483	0.151134	-2.30307
ENST00000670258.1	1.164194	2.174089	4.00509	0.008502	0.151151	-2.30536
T314050	1.344213	2.607261	4.004354	0.008508	0.151228	-2.30619
lnc-GOT1-1:1	-1.09118	3.61128	-4.0024	0.008526	0.151317	-2.30837
lnc-SIX4-2:1	1.023046	3.579857	4.001826	0.008531	0.151317	-2.30901
lnc-UBAC2-5:1	-1.65614	4.574726	-3.99982	0.00855	0.151577	-2.31125
NONHSAT163657.1	-1.22681	4.68726	-3.99777	0.008568	0.151731	-2.31355
NONHSAT208702.1	-1.07296	6.440464	-3.99736	0.008572	0.151755	-2.31401
NONHSAT167452.1	1.331426	2.953906	3.993482	0.008608	0.152052	-2.31835
NONHSAT160094.1	-1.07135	1.695203	-3.9931	0.008611	0.15206	-2.31878
NONHSAT168638.1	-1.16759	1.695369	-3.98863	0.008652	0.152438	-2.32379
NONHSAT158174.1	-1.65079	4.460992	-3.98816	0.008657	0.152438	-2.32432
lnc-CCT5-8:1	1.846289	3.245079	3.987473	0.008663	0.1525	-2.32509
NONHSAT173439.1	2.066742	2.264817	3.987384	0.008664	0.1525	-2.32519
lnc-C2orf91-5:1	1.043327	1.598315	3.987284	0.008665	0.1525	-2.3253
NONHSAT220051.1	-1.47665	2.150245	-3.98535	0.008683	0.152589	-2.32747
lnc-PLLP-2:1	-1.03012	11.03062	-3.98527	0.008683	0.152589	-2.32756
NONHSAT170414.1	1.863219	2.446155	3.984469	0.008691	0.152642	-2.32846
MSTRG.6455.1	1.740406	2.936687	3.984293	0.008692	0.152642	-2.32865
MSTRG.23202.1	1.003991	2.933227	3.984032	0.008695	0.152646	-2.32895
ENST00000670156.1	1.212381	3.528174	3.98272	0.008707	0.152754	-2.33042
NONHSAT208007.1	-1.09088	1.739769	-3.98148	0.008719	0.152887	-2.33181
MSTRG.37623.1	-1.10843	4.61739	-3.97991	0.008733	0.152887	-2.33357
T188397	-1.1267	2.133402	-3.97919	0.00874	0.152887	-2.33438
NONHSAT164534.1	-1.33224	4.262676	-3.97891	0.008743	0.152887	-2.3347
ENST00000575767.1	-1.04733	4.106166	-3.97819	0.008749	0.152895	-2.3355
ENST00000524085.2	-1.1358	1.883463	-3.97761	0.008755	0.152963	-2.33615
ENST00000488190.1	1.692095	2.318334	3.975907	0.008771	0.153109	-2.33807
ENST00000479822.2	1.458502	3.456537	3.974588	0.008783	0.15317	-2.33955
NONHSAT178297.1	-1.0254	1.760109	-3.97095	0.008817	0.153525	-2.34364
T244137	1.697366	2.669738	3.970451	0.008822	0.153547	-2.3442
NONHSAT224236.1	1.173189	2.35979	3.970007	0.008826	0.153547	-2.3447
lnc-ZNF729-1:1	-1.08371	3.73904	-3.96794	0.008846	0.153594	-2.34702
lnc-UGGT1-8:1	1.421769	2.945839	3.967842	0.008847	0.153594	-2.34713
lnc-SENP2-11:1	-1.01548	4.257255	-3.96779	0.008847	0.153594	-2.34719
NONHSAT153470.1	1.294119	5.185985	3.965502	0.008869	0.153678	-2.34976
MSTRG.26293.8	-1.06539	2.971488	-3.96494	0.008874	0.153678	-2.35039
ENST00000668682.1	1.524635	2.479326	3.963983	0.008883	0.153678	-2.35147
ENST00000668117.1	1.673722	3.482055	3.963173	0.008891	0.153678	-2.35238
NONHSAT172187.1	1.06794	2.428367	3.961501	0.008907	0.153758	-2.35426

NR_110114	1.213996	1.946884	3.959343	0.008928	0.153956	-2.35669
NONHSAT191345.1	-1.08181	2.382397	-3.95903	0.008931	0.15398	-2.35704
NONHSAT161414.1	1.101367	3.17394	3.957368	0.008947	0.154036	-2.35892
lnc-OR4C13-7:3	1.411661	2.398739	3.957355	0.008947	0.154036	-2.35893
ENST00000568587.1	-1.44157	3.71023	-3.95694	0.008951	0.154036	-2.3594
ENST00000554595.2	-1.37473	2.560582	-3.95592	0.00896	0.154039	-2.36055
lnc-HPRT1-7:2	1.124851	3.746177	3.953778	0.008981	0.154139	-2.36296
lnc-STX18-2:1	1.107601	3.4331	3.953272	0.008986	0.154144	-2.36353
ENST00000664918.1	-1.55328	3.791643	-3.95241	0.008994	0.154208	-2.3645
ENST00000658970.1	-1.12645	4.85211	-3.95126	0.009005	0.15432	-2.3658
NONHSAT197134.1	-1.00105	6.594207	-3.94982	0.009019	0.154466	-2.36742
NONHSAT218416.1	1.460239	1.855834	3.949747	0.00902	0.154466	-2.3675
NONHSAT171468.1	-1.11091	3.852513	-3.94965	0.009021	0.154466	-2.36761
ENST00000644669.1	-1.02235	2.822454	-3.94886	0.009029	0.154466	-2.3685
lnc-GOT2-6:1	-1.44566	5.048729	-3.94865	0.009031	0.154466	-2.36874
lnc-RUNX1T1-8:1	-1.07136	9.79414	-3.9482	0.009035	0.154485	-2.36925
PDZRN3-AS1:8	1.325781	2.582104	3.947863	0.009038	0.154489	-2.36963
NONHSAT161842.1	1.028754	1.843263	3.947326	0.009043	0.154543	-2.37023
NONHSAT205159.1	1.490115	2.240128	3.945912	0.009057	0.154543	-2.37183
NONHSAT158307.1	2.092066	2.903831	3.945153	0.009065	0.154543	-2.37269
lnc-CLEC4C-1:1	1.45362	2.041924	3.945131	0.009065	0.154543	-2.37271
NONHSAT199491.1	1.958236	2.385641	3.944397	0.009072	0.154543	-2.37354
lnc-XRN2-7:1	1.196531	2.398804	3.943778	0.009078	0.154618	-2.37424
lnc-RTP4-7:3	1.070483	4.194909	3.942328	0.009092	0.154702	-2.37587
NONHSAT211434.1	1.506358	2.630611	3.940902	0.009106	0.154861	-2.37748
ENST00000625090.1	1.506439	3.033125	3.938944	0.009125	0.154906	-2.37969
MSTRG.26937.1	1.365068	1.899177	3.938763	0.009127	0.154906	-2.3799
MSTRG.25126.1	-1.74359	3.669122	-3.93849	0.00913	0.154906	-2.38021
NONHSAT187308.1	-1.33359	5.025584	-3.93844	0.00913	0.154906	-2.38027
lnc-NPR3-2:5	2.392694	2.552424	3.937496	0.009139	0.15501	-2.38133
NONHSAT207110.1	1.256066	1.681147	3.937289	0.009141	0.15501	-2.38156
NONHSAT219017.1	-1.11163	1.850064	-3.93497	0.009164	0.155193	-2.38418
NONHSAT159456.1	1.539947	1.856434	3.934725	0.009167	0.155193	-2.38446
lnc-RAB23-1:5	1.037091	4.555529	3.934068	0.009173	0.155193	-2.3852
lnc-FZD1-4:1	1.431302	5.060232	3.933996	0.009174	0.155193	-2.38528
T217782	-1.0634	3.428893	-3.93387	0.009175	0.155193	-2.38543
lnc-DRD1-2:1	-1.36458	2.783441	-3.93341	0.00918	0.155193	-2.38594
lnc-AMPD3-3:1	1.383329	2.95986	3.932886	0.009185	0.155193	-2.38654
MSTRG.2479.1	1.262648	2.91068	3.93276	0.009186	0.155193	-2.38668
ENST00000563464.1	-1.70553	2.292639	-3.93276	0.009186	0.155193	-2.38668
lnc-COL6A5-4:5	-1.06829	3.340166	-3.93226	0.009191	0.155193	-2.38724
lnc-ACTR3B-6:1	1.326046	3.203694	3.93203	0.009193	0.155193	-2.38751
NONHSAT185309.1	-1.14749	1.705917	-3.93185	0.009195	0.155193	-2.38772
NR_038385	1.289991	1.750494	3.931722	0.009196	0.155193	-2.38785
lnc-ITGB3BP-10:1	1.223791	1.841813	3.931008	0.009203	0.155286	-2.38866
lnc-TADA1-2:3	-1.06751	3.136693	-3.9304	0.009209	0.15531	-2.38935
NONHSAT161581.1	1.006423	3.308005	3.929738	0.009216	0.155382	-2.3901
NONHSAT210334.1	2.338969	3.308812	3.929393	0.009219	0.155382	-2.39049

ENST00000659877.1	-1.26358	3.258703	-3.92861	0.009227	0.155428	-2.39137
NONHSAT189736.1	-1.2495	1.748098	-3.92794	0.009234	0.155463	-2.39213
lnc-DHX37-4:1	-1.30121	6.005454	-3.92787	0.009235	0.155463	-2.39221
NONHSAT169549.1	-1.21947	4.064597	-3.92707	0.009243	0.155485	-2.39312
ENST00000639687.1	1.53596	2.398213	3.925498	0.009258	0.155492	-2.39489
lnc-TBP-10:1	-1.50717	2.855282	-3.92481	0.009265	0.155534	-2.39567
MSTRG.64374.1	-1.49789	2.203846	-3.92464	0.009267	0.155534	-2.39586
ENST00000650740.1	-1.24616	2.18675	-3.9215	0.009298	0.155789	-2.39942
NONHSAT197689.1	1.394564	2.366803	3.921133	0.009302	0.155825	-2.39983
lnc-IPCEF1-4:1	-1.13666	8.391266	-3.919	0.009323	0.156075	-2.40225
lnc-TFAM-7:1	1.183117	3.035399	3.91817	0.009332	0.156075	-2.40319
ENST00000438247.1	1.21257	1.824383	3.917924	0.009334	0.156075	-2.40347
NONHSAT184695.1	-1.10516	1.690608	-3.91717	0.009342	0.156129	-2.40432
lnc-SNW1-8:1	-1.73318	3.336588	-3.91649	0.009348	0.156159	-2.40509
lnc-TNS3-4:2	-1.17368	2.035481	-3.91635	0.00935	0.156159	-2.40526
lnc-ADCY8-3:1	1.804636	3.955502	3.916239	0.009351	0.156159	-2.40538
NONHSAT169353.1	1.193249	2.424378	3.916061	0.009353	0.15616	-2.40558
T232938	-1.28851	4.795225	-3.9153	0.00936	0.156198	-2.40644
T008430	-1.34291	4.144825	-3.91258	0.009388	0.156359	-2.40953
lnc-CD207-3:1	1.065126	1.638295	3.91188	0.009395	0.156359	-2.41032
NONHSAT197139.1	-1.71966	2.65486	-3.91079	0.009406	0.156416	-2.41155
MSTRG.47672.1	1.716009	2.382925	3.910259	0.009412	0.156416	-2.41216
lnc-SLC18A1-1:1	-1.00541	5.246795	-3.90631	0.009452	0.156766	-2.41664
lnc-SLC10A6-3:1	1.342605	2.491429	3.906111	0.009454	0.156766	-2.41686
NONHSAT188541.1	2.056654	2.70397	3.903541	0.00948	0.156922	-2.41978
MSTRG.43658.5	1.353571	2.684242	3.903379	0.009482	0.156922	-2.41996
lnc-CDH10-11:1	-1.13744	3.929037	-3.90336	0.009482	0.156922	-2.41999
lnc-AREG-1:6	-1.57214	1.932315	-3.9027	0.009489	0.156922	-2.42074
NONHSAT214781.1	1.418014	2.449166	3.901187	0.009504	0.157069	-2.42245
NONHSAT163888.1	1.530547	4.952304	3.900471	0.009512	0.157069	-2.42327
NONHSAT214748.1	1.212672	1.700209	3.900463	0.009512	0.157069	-2.42327
ENST00000504245.6	1.020369	4.081558	3.89946	0.009522	0.15709	-2.42441
NONHSAT189814.1	1.391363	1.794244	3.898909	0.009528	0.15709	-2.42504
ENST00000654321.1	-1.16959	1.995139	-3.8988	0.009529	0.15709	-2.42517
NONHSAT158448.1	1.051419	3.42464	3.898642	0.00953	0.15709	-2.42534
lnc-BOC-2:1	1.079834	2.064495	3.898524	0.009532	0.15709	-2.42548
lnc-CTTNBP2NL-3:1	-1.16983	8.320584	-3.89627	0.009555	0.157223	-2.42804
SAMMSON:10	1.154729	2.47348	3.895525	0.009563	0.157236	-2.42888
ENST00000574098.1	1.144943	1.651718	3.894961	0.009568	0.15726	-2.42953
NONHSAT187950.1	-1.10148	2.029465	-3.89291	0.00959	0.157306	-2.43186
lnc-TEX10-1:1	1.903384	3.714464	3.892454	0.009594	0.157306	-2.43238
NONHSAT154561.1	1.408261	1.868453	3.892323	0.009596	0.157306	-2.43252
lnc-GPR137B-8:1	-1.02504	5.344954	-3.89098	0.00961	0.157348	-2.43405
ENST00000657842.1	-1.22668	2.654444	-3.88764	0.009644	0.157729	-2.43785
lnc-UGT1A8-1:1	1.223731	1.71426	3.887137	0.00965	0.157729	-2.43842
ENST00000656956.1	-1.73159	3.199496	-3.88707	0.00965	0.157729	-2.4385
ENST00000546804.1	2.263743	2.900534	3.886092	0.009661	0.157763	-2.43961
NONHSAT200253.1	-1.21506	2.041617	-3.88264	0.009697	0.157942	-2.44355

lnc-SMAD5-8:1	1.106063	1.638187	3.882612	0.009697	0.157942	-2.44358
ENST00000540392.1	-1.18798	2.301003	-3.88257	0.009698	0.157942	-2.44363
LINC00689:9	2.070407	2.329281	3.881817	0.009705	0.157982	-2.44448
ENST00000649143.1	1.467682	1.778385	3.880839	0.009716	0.157983	-2.44559
lnc-MYNN-9:1	-1.00114	3.571291	-3.8804	0.00972	0.158006	-2.44609
lnc-MUC20-4:3	1.600058	3.727501	3.880075	0.009724	0.158037	-2.44646
MSTRG.62727.7	1.221582	2.648393	3.879902	0.009726	0.158041	-2.44666
lnc-ANGPT2-3:2	-1.4095	3.584473	-3.8793	0.009732	0.158052	-2.44734
NONHSAT183557.1	2.34693	3.601404	3.878743	0.009738	0.158062	-2.44798
ENST00000641075.1	1.535627	2.477803	3.878141	0.009744	0.158089	-2.44867
T223393	1.03555	1.678094	3.877788	0.009748	0.158098	-2.44907
lnc-ABHD4-6:1	-1.45502	3.011901	-3.87738	0.009752	0.158117	-2.44953
lnc-SERPINB2-1:1	1.514963	2.470771	3.876439	0.009762	0.158119	-2.45061
NONHSAT161364.1	1.168048	5.315073	3.876439	0.009762	0.158119	-2.45061
NONHSAT158496.1	-1.05904	2.834919	-3.87618	0.009765	0.15812	-2.4509
NONHSAT173254.1	1.811105	2.915045	3.875682	0.00977	0.158129	-2.45147
lnc-CYBRD1-1:1	-1.10259	2.496009	-3.87539	0.009773	0.158153	-2.4518
NONHSAT186734.1	1.367059	2.244974	3.875047	0.009777	0.158162	-2.45219
NONHSAT166167.1	-1.04759	1.735349	-3.87424	0.009786	0.158196	-2.45312
lnc-LGALSL-5:1	-1.45275	3.289817	-3.87241	0.009805	0.158253	-2.4552
NONHSAT197583.1	-1.97568	4.651031	-3.87186	0.009811	0.158253	-2.45583
NONHSAT193303.1	-1.02414	1.930026	-3.87165	0.009813	0.158263	-2.45606
lnc-NAP1L3-7:1	1.394161	3.844733	3.871347	0.009816	0.158288	-2.45641
lnc-RAP2B-2:1	-1.34147	8.95786	-3.87094	0.009821	0.158288	-2.45688
lnc-DUSP26-13:1	1.488198	2.045811	3.870696	0.009823	0.158288	-2.45716
T279692	1.150652	2.13176	3.869677	0.009834	0.158375	-2.45832
MSTRG.831.1	1.370232	4.033243	3.869033	0.009841	0.158409	-2.45905
T148754	1.319183	3.469128	3.868726	0.009844	0.158409	-2.4594
lnc-ZNF479-12:1	-1.20443	3.028823	-3.86767	0.009856	0.158481	-2.4606
ENST00000669053.1	1.261258	2.357839	3.866015	0.009873	0.15855	-2.4625
NONHSAT223715.1	3.269224	3.381287	3.865568	0.009878	0.158576	-2.46301
lnc-ISX-5:1	1.313894	2.165868	3.864075	0.009894	0.158734	-2.46471
NONHSAT174436.1	2.062192	2.473942	3.863979	0.009895	0.158734	-2.46482
NONHSAT214652.1	1.178488	3.389958	3.863697	0.009898	0.158734	-2.46514
ENST00000442815.1	1.605455	2.856363	3.863536	0.0099	0.158734	-2.46533
MSTRG.44436.21	1.735736	2.379579	3.862901	0.009907	0.158734	-2.46605
ENST00000414418.1	1.029894	5.639478	3.862537	0.009911	0.158734	-2.46647
T342925	-1.05984	3.61683	-3.86231	0.009913	0.158734	-2.46673
lnc-IFT80-3:1	1.429279	2.7681	3.860826	0.009929	0.158805	-2.46842
NONHSAT171050.1	1.870864	3.000713	3.860189	0.009936	0.158809	-2.46915
lnc-CIB2-1:1	1.259225	3.326322	3.860127	0.009937	0.158809	-2.46922
NONHSAT210686.1	1.196769	2.563056	3.860126	0.009937	0.158809	-2.46922
lnc-ZNF781-2:2	1.540773	2.05371	3.857716	0.009963	0.159092	-2.47198
lnc-PITPNB-3:1	1.933435	2.52199	3.85749	0.009965	0.159092	-2.47224
lnc-POLR2J2-2:2	-1.63603	7.298314	-3.85681	0.009973	0.159092	-2.47301
lnc-SEL1L3-9:1	-1.26496	8.796072	-3.85677	0.009973	0.159092	-2.47306
NONHSAT213534.1	-1.04086	6.299462	-3.85516	0.00999	0.15929	-2.4749
ENST00000621389.1	2.432896	6.829352	3.853101	0.010013	0.159395	-2.47725

NR_073407	-1.01504	3.652289	-3.85102	0.010036	0.159604	-2.47963
MSTRG.5623.1	1.674764	7.862293	3.850294	0.010043	0.159706	-2.48046
MSTRG.44407.1	-1.41726	2.574424	-3.8482	0.010066	0.159792	-2.48285
lnc-FOXQ1-14:1	-1.55853	3.11311	-3.84393	0.010113	0.160015	-2.48775
NONHSAT216535.1	-1.00344	1.730933	-3.8422	0.010132	0.160164	-2.48973
NONHSAT178204.1	-1.01603	4.117065	-3.83965	0.010161	0.160279	-2.49265
lnc-DAGLA-3:2	1.728303	2.288176	3.839436	0.010163	0.160292	-2.4929
NONHSAT211759.1	1.760923	4.095951	3.838277	0.010176	0.160394	-2.49422
lnc-C15orf41-15:1	-1.20288	4.911465	-3.83677	0.010193	0.160498	-2.49595
NONHSAT219305.1	-1.33297	2.035644	-3.83663	0.010194	0.160498	-2.49612
MSTRG.24918.1	1.1155	1.672692	3.836023	0.010201	0.160513	-2.49681
ENST00000624459.2	1.306324	1.879696	3.835763	0.010204	0.160533	-2.49711
MSTRG.60246.1	2.095468	2.189073	3.835563	0.010206	0.160543	-2.49733
lnc-CEP63-3:1	1.140671	4.462824	3.835018	0.010212	0.160613	-2.49796
MSTRG.63467.1	1.542148	2.243673	3.83349	0.010229	0.160683	-2.49971
ENST00000505575.1	1.557698	6.361727	3.832761	0.010237	0.160759	-2.50055
MSTRG.49027.1	1.165088	2.296292	3.831576	0.01025	0.160766	-2.50191
lnc-KCMF1-1:2	1.289491	3.590309	3.831372	0.010253	0.160777	-2.50214
NONHSAT188113.1	-1.16683	2.65281	-3.83113	0.010255	0.160794	-2.50242
ENST00000625127.3	-1.13452	3.612295	-3.82985	0.01027	0.160868	-2.50388
NONHSAT224049.1	-1.33429	3.435612	-3.82902	0.010279	0.160915	-2.50484
ENST00000412809.1	1.276756	5.248793	3.825765	0.010316	0.161135	-2.50858
lnc-ACTRT2-1:3	1.287633	3.877471	3.822741	0.01035	0.161296	-2.51205
MSTRG.38433.1	1.70397	2.173021	3.821384	0.010365	0.161346	-2.51361
NR_027024	-1.78577	2.39695	-3.82104	0.010369	0.161346	-2.514
lnc-OSBPL1A-7:1	-1.18887	2.555215	-3.82065	0.010373	0.161365	-2.51445
lnc-VGLL4-8:1	-1.32906	7.266264	-3.81862	0.010397	0.161422	-2.51678
NONHSAT163380.1	-1.12404	1.910172	-3.81859	0.010397	0.161422	-2.51682
MSTRG.49184.1	-1.05627	8.272356	-3.81837	0.010399	0.161422	-2.51708
MSTRG.5852.1	1.607121	2.218097	3.816582	0.01042	0.16165	-2.51913
lnc-SCGN-6:1	-1.32149	2.201071	-3.81546	0.010433	0.161736	-2.52041
NONHSAT206219.1	1.920637	5.399823	3.815326	0.010434	0.161736	-2.52057
NONHSAT154700.1	1.143468	2.016418	3.815323	0.010434	0.161736	-2.52058
NONHSAT208204.1	-1.06743	4.48429	-3.81522	0.010435	0.161736	-2.5207
ENST00000437461.1	1.002336	6.146848	3.815107	0.010437	0.161736	-2.52082
NR_110702	-1.15492	3.321443	-3.81478	0.01044	0.161736	-2.52121
lnc-PJA1-4:2	-1.30787	4.792384	-3.81322	0.010458	0.161746	-2.523
T290423	-1.38607	5.946177	-3.81252	0.010466	0.161746	-2.5238
lnc-MKKS-8:1	-1.1733	3.021715	-3.81233	0.010468	0.161746	-2.52402
NONHSAT224060.1	1.521917	3.66023	3.811499	0.010478	0.161762	-2.52497
NONHSAT160770.1	1.504432	2.617258	3.810282	0.010492	0.161795	-2.52638
ENST00000452911.1	-1.35837	1.764724	-3.80984	0.010497	0.161795	-2.52689
lnc-CLEC1B-1:2	1.203891	5.32643	3.809815	0.010497	0.161795	-2.52691
NONHSAT207417.1	1.274333	3.485297	3.809727	0.010498	0.161795	-2.52701
lnc-ZPBP-8:1	-1.31087	3.900319	-3.80963	0.010499	0.161795	-2.52712
NONHSAT180303.1	1.285904	2.023428	3.808394	0.010514	0.161795	-2.52855
lnc-HMGCR-3:1	1.726346	2.025635	3.806299	0.010538	0.161946	-2.53096
lnc-MLLT3-2:1	-1.19023	2.611466	-3.80531	0.010549	0.161992	-2.5321

ENST00000567386.1	-1.12711	2.865773	-3.80436	0.01056	0.162043	-2.53319
MSTRG.62109.1	1.048406	2.436801	3.803823	0.010566	0.162114	-2.53381
lnc-SLC25A12-8:1	-1.71747	2.865156	-3.80256	0.010581	0.162229	-2.53527
NONHSAT154610.1	1.532632	2.191768	3.802085	0.010586	0.16225	-2.53581
lnc-PAX1-10:1	-1.00868	5.08822	-3.80129	0.010596	0.162293	-2.53673
lnc-STRADB-4:1	-2.04507	4.399131	-3.80076	0.010602	0.162293	-2.53734
lnc-TBL1XR1-12:1	-1.44295	2.776757	-3.80035	0.010607	0.162293	-2.53781
NONHSAT176709.1	1.892655	1.99184	3.799939	0.010611	0.162293	-2.53829
MSTRG.8952.1	1.406263	1.810908	3.79985	0.010612	0.162293	-2.53839
NONHSAT188189.1	1.206804	2.28797	3.799677	0.010614	0.162293	-2.53859
MSTRG.52747.10	-1.03938	2.07055	-3.79963	0.010615	0.162293	-2.53864
LINC00887:17	-1.82517	9.894107	-3.79737	0.010641	0.162503	-2.54125
NONHSAT179776.1	-1.74159	3.622118	-3.79624	0.010654	0.162584	-2.54255
NONHSAT171928.1	1.387179	2.29581	3.796198	0.010655	0.162584	-2.5426
MSTRG.37738.2	-1.37791	2.899795	-3.79567	0.010661	0.162654	-2.54321
lnc-NYAP2-6:1	-1.05405	3.664289	-3.79421	0.010678	0.16275	-2.5449
ENST00000509750.1	-1.54798	2.139217	-3.79416	0.010679	0.16275	-2.54495
lnc-MPP7-8:2	-1.50096	3.081834	-3.79323	0.01069	0.162763	-2.54602
NONHSAT217154.1	-1.92846	2.85368	-3.79315	0.010691	0.162763	-2.54611
lnc-FCN1-5:1	1.41259	2.032495	3.792986	0.010693	0.162763	-2.5463
NONHSAT180875.1	1.359757	3.875619	3.791318	0.010712	0.162914	-2.54823
ENST00000416343.2	-1.79894	2.372123	-3.78915	0.010738	0.16309	-2.55074
NONHSAT211083.1	1.014012	5.25247	3.788963	0.01074	0.16309	-2.55095
MSTRG.45548.1	-1.00026	3.396087	-3.78868	0.010743	0.163091	-2.55127
lnc-PRR32-8:1	-1.40569	3.396557	-3.78544	0.010782	0.163279	-2.55501
NONHSAT156091.1	-1.07593	1.9822	-3.78378	0.010801	0.16344	-2.55694
lnc-MRPL19-14:1	-1.19702	1.729163	-3.78198	0.010823	0.163488	-2.55901
lnc-FSHR-7:1	1.828305	1.946259	3.78168	0.010826	0.163488	-2.55936
NONHSAT218650.1	-1.2188	2.021001	-3.78101	0.010834	0.163525	-2.56013
ENST00000664367.1	1.046617	3.703711	3.780521	0.01084	0.16355	-2.5607
ENST00000600074.1	1.05713	3.905488	3.779674	0.01085	0.16358	-2.56168
MSTRG.2214.4	1.066409	1.596368	3.779183	0.010856	0.163603	-2.56224
lnc-ZNF66-3:1	1.145024	2.849791	3.777999	0.01087	0.163623	-2.56361
NONHSAT217657.1	1.207054	4.306874	3.776053	0.010893	0.163792	-2.56586
NONHSAT170181.1	-1.15569	2.477915	-3.77582	0.010896	0.16381	-2.56613
lnc-HOXA13-4:1	1.038216	1.919486	3.773808	0.01092	0.163918	-2.56846
lnc-ASMT-5:1	-1.43981	1.966695	-3.77333	0.010926	0.163918	-2.56901
lnc-IRF7-1:1	1.36509	3.595979	3.772558	0.010935	0.163958	-2.56991
lnc-MIER3-4:1	-1.11224	7.391184	-3.76863	0.010983	0.164195	-2.57445
ENST00000527318.1	1.437163	4.321933	3.768036	0.01099	0.164195	-2.57514
NONHSAT187141.1	1.575904	3.912001	3.767741	0.010993	0.164195	-2.57548
MSTRG.52730.1	1.083798	3.053622	3.766661	0.011007	0.164317	-2.57673
ENST00000660105.1	-1.12338	2.200085	-3.76564	0.011019	0.164428	-2.57791
NONHSAT187340.1	-1.13469	2.270353	-3.76562	0.011019	0.164428	-2.57793
NONHSAT178607.1	-1.03455	6.278283	-3.76551	0.01102	0.164428	-2.57806
NONHSAT153653.1	-1.2553	3.557659	-3.76471	0.01103	0.16444	-2.57899
NONHSAT190185.1	-1.3297	1.779483	-3.764	0.011039	0.164458	-2.57981
NONHSAT164072.1	-1.59318	2.297971	-3.76246	0.011058	0.164519	-2.58159

NONHSAT197747.1	1.893944	3.993585	3.761892	0.011065	0.164554	-2.58226
lnc-RGMB-6:1	-1.3977	3.135823	-3.76174	0.011066	0.164554	-2.58244
T072173	1.519119	2.000639	3.760557	0.011081	0.164673	-2.5838
NONHSAT217935.1	1.695784	2.601805	3.760483	0.011082	0.164673	-2.58389
NONHSAT222083.1	-1.93419	3.681531	-3.75986	0.011089	0.164673	-2.58461
NONHSAT224314.1	1.22941	4.176277	3.75806	0.011111	0.164829	-2.5867
lnc-ALPK2-4:2	1.004006	3.392581	3.757183	0.011122	0.164895	-2.58771
ENST00000663358.1	1.505533	2.247059	3.75655	0.01113	0.164986	-2.58845
MSTRG.53624.1	-1.3584	4.983905	-3.75568	0.011141	0.165073	-2.58946
lnc-TECRL-10:1	-1.19189	12.81817	-3.75541	0.011144	0.165097	-2.58977
ENST00000424252.2	-1.08395	1.920247	-3.75488	0.01115	0.165169	-2.59039
NONHSAT217700.1	1.830627	5.838113	3.754291	0.011158	0.165231	-2.59107
NONHSAT217927.1	-1.59228	2.102791	-3.754	0.011161	0.165231	-2.59141
MSTRG.67637.2	-1.50909	3.351723	-3.75347	0.011168	0.165231	-2.59202
lnc-SLC35G3-2:1	1.190138	3.877956	3.753337	0.011169	0.165232	-2.59218
lnc-VANGL1-3:1	-1.1184	11.19648	-3.75186	0.011188	0.165337	-2.59389
NONHSAT209549.1	1.094752	2.188125	3.751499	0.011192	0.165337	-2.59431
ENST00000614612.1	-1.12362	6.030515	-3.75148	0.011192	0.165337	-2.59433
ENST00000325660.3	-1.26351	2.978711	-3.75074	0.011201	0.165389	-2.59519
MSTRG.15917.1	-1.34535	2.419201	-3.75057	0.011204	0.165389	-2.59539
ENST00000651165.1	1.653978	2.522564	3.750219	0.011208	0.165389	-2.59579
ENST00000446321.1	1.295576	2.846444	3.748813	0.011225	0.165536	-2.59743
MSTRG.10927.13	-1.12443	5.596434	-3.74755	0.011241	0.165586	-2.59889
MSTRG.12377.1	-1.24253	2.085056	-3.74732	0.011244	0.165604	-2.59916
T145026	-1.02947	5.858834	-3.74656	0.011253	0.16567	-2.60004
MSTRG.26636.1	-1.3079	2.992252	-3.74522	0.01127	0.165753	-2.6016
LINC01467:1	-1.12392	2.001528	-3.74452	0.011279	0.165753	-2.60241
lnc-PHKA1-3:1	-1.38191	4.820993	-3.7445	0.011279	0.165753	-2.60244
lnc-SNX7-7:1	1.66484	4.191386	3.74427	0.011282	0.165753	-2.6027
lnc-AWAT2-2:1	-1.19681	2.034013	-3.74351	0.011291	0.165839	-2.60359
NONHSAT182773.1	-1.52164	2.682036	-3.74238	0.011305	0.165946	-2.60489
NONHSAT223177.1	-1.45316	2.537082	-3.74146	0.011317	0.16598	-2.60596
lnc-OR10AD1-1:1	1.32165	2.487558	3.740586	0.011328	0.166075	-2.60698
lnc-ENTPD7-2:1	1.065331	3.325283	3.740405	0.01133	0.166075	-2.60719
lnc-GPRC5D-1:1	1.589201	2.419904	3.739243	0.011345	0.166214	-2.60854
NONHSAT158631.1	-1.52985	2.379764	-3.73833	0.011356	0.166281	-2.60961
NONHSAT184394.1	1.521081	2.627366	3.736957	0.011373	0.166393	-2.6112
MSTRG.30056.3	-1.05914	1.87069	-3.73612	0.011384	0.166409	-2.61217
MSTRG.37338.1	-1.03634	2.940804	-3.73379	0.011413	0.166564	-2.61488
NONHSAT204519.1	1.386943	3.820781	3.733024	0.011423	0.166608	-2.61578
MSTRG.68367.3	1.857148	3.296581	3.732754	0.011426	0.166608	-2.61609
MSTRG.35149.1	1.147589	3.243373	3.732067	0.011435	0.166667	-2.61689
ENST00000661436.1	1.665868	2.0494	3.729765	0.011464	0.166947	-2.61957
MSTRG.26957.1	2.059043	2.15028	3.728653	0.011478	0.167032	-2.62087
MSTRG.2840.1	1.093286	1.660543	3.728594	0.011479	0.167032	-2.62093
lnc-KMT2B-1:1	-1.44981	2.127059	-3.72807	0.011486	0.167036	-2.62155
T083618	1.423899	2.074955	3.728046	0.011486	0.167036	-2.62157
lnc-E2F8-3:1	1.354949	1.960267	3.727591	0.011492	0.16704	-2.6221

ENST00000453951.1	1.860343	2.670291	3.725209	0.011522	0.167062	-2.62488
NONHSAT187014.1	-1.19335	8.284327	-3.72494	0.011526	0.167062	-2.62518
lnc-CACNA1I-1:1	-1.26889	3.562998	-3.72486	0.011527	0.167062	-2.62528
lnc-NRG2-8:1	1.022901	1.889395	3.724012	0.011537	0.167062	-2.62627
T286199	1.165211	3.838613	3.723939	0.011538	0.167062	-2.62636
NONHSAT217991.1	1.694827	3.701988	3.723292	0.011547	0.167087	-2.62711
LINC01829:27	1.237226	3.358172	3.719447	0.011596	0.16752	-2.63159
MSTRG.7430.1	-1.29801	2.229426	-3.71879	0.011604	0.167538	-2.63236
MAGI1-IT1:2	1.09508	4.865433	3.718503	0.011608	0.167548	-2.63269
NONHSAT154311.1	-1.21376	3.096301	-3.71787	0.011616	0.167613	-2.63343
lnc-LOX-4:12	1.005786	4.316368	3.713511	0.011673	0.167969	-2.63851
NONHSAT196935.1	-1.2163	3.992794	-3.71275	0.011682	0.168013	-2.6394
lnc-MAGEA1-2:1	1.895637	2.135952	3.712063	0.011691	0.16805	-2.6402
NONHSAT154716.1	1.634144	2.637213	3.711588	0.011698	0.16805	-2.64076
lnc-MEX3C-7:1	1.051365	1.907672	3.711527	0.011698	0.16805	-2.64083
NONHSAT153419.1	1.337736	2.384837	3.709942	0.011719	0.168179	-2.64268
ENST00000571506.1	-1.58079	9.056842	-3.70851	0.011737	0.16835	-2.64435
NONHSAT205624.1	-1.23548	2.108444	-3.70793	0.011745	0.168388	-2.64503
NONHSAT157750.1	-1.0217	1.702735	-3.70751	0.011751	0.16842	-2.64552
MSTRG.53367.1	1.754222	3.073916	3.707115	0.011756	0.168442	-2.64598
lnc-ADGRF5-2:1	1.252822	4.419241	3.706724	0.011761	0.168469	-2.64644
NONHSAT216354.1	-1.15736	3.997911	-3.70642	0.011765	0.168502	-2.64679
ENST00000520426.1	1.869441	2.543382	3.706167	0.011768	0.168526	-2.64709
T136954	-1.56494	4.635443	-3.703	0.01181	0.168899	-2.65078
lnc-NPIPA7-1:1	-1.37037	2.639488	-3.70233	0.011818	0.168899	-2.65157
lnc-ZC3H11B-10:1	-1.27064	2.084304	-3.70076	0.011839	0.169109	-2.6534
MSTRG.46430.6	1.53209	2.627988	3.70058	0.011841	0.169119	-2.65362
lnc-RARS2-2:1	-1.23439	5.111256	-3.69982	0.011851	0.169225	-2.65451
lnc-SPAG16-11:2	-1.03353	2.387735	-3.69976	0.011852	0.169225	-2.65457
lnc-ZNF490-3:1	1.051592	3.965338	3.698861	0.011864	0.169273	-2.65563
lnc-ZNF674-5:1	-1.30291	4.663508	-3.69678	0.011892	0.169523	-2.65806
NONHSAT217040.1	1.914608	2.580659	3.695188	0.011913	0.169679	-2.65992
NR_027711	1.871859	2.525547	3.692834	0.011944	0.169842	-2.66268
MSTRG.8860.2	2.418525	3.427377	3.692804	0.011944	0.169842	-2.66271
MSTRG.15381.1	1.189484	1.683918	3.688991	0.011995	0.170204	-2.66717
NONHSAT168663.1	1.142616	1.763121	3.688419	0.012003	0.170215	-2.66784
MSTRG.25860.1	1.229817	6.295297	3.688361	0.012003	0.170215	-2.66791
ENST00000435832.1	1.107895	2.023512	3.688302	0.012004	0.170215	-2.66798
NONHSAT217663.1	-1.0585	4.366459	-3.68677	0.012025	0.170245	-2.66978
T057656	1.580559	2.335623	3.685519	0.012042	0.170343	-2.67124
lnc-PEPD-2:1	1.701534	2.313721	3.685487	0.012042	0.170343	-2.67128
ENST00000653501.1	1.963996	2.769607	3.684397	0.012057	0.170343	-2.67256
MSTRG.6431.1	1.345674	8.504623	3.684345	0.012057	0.170343	-2.67262
NONHSAT175233.1	-1.23594	2.436757	-3.6841	0.012061	0.170343	-2.67291
ENST00000570167.1	-1.37066	3.040352	-3.68409	0.012061	0.170343	-2.67292
NONHSAT153483.1	1.125057	5.790892	3.682239	0.012086	0.170555	-2.67508
lnc-CCDC177-2:1	-1.20574	4.897746	-3.68148	0.012096	0.17065	-2.67598
NONHSAT210699.1	1.194661	2.05177	3.68114	0.0121	0.170689	-2.67637

lnc-ODF1-3:2	-1.11904	8.349093	-3.68052	0.012109	0.170752	-2.6771
NONHSAT155020.1	1.489446	4.565512	3.679809	0.012118	0.17078	-2.67793
ENST00000427398.1	-1.69905	7.479965	-3.67954	0.012122	0.170783	-2.67825
lnc-FBXL3-2:1	-1.71183	4.23589	-3.67952	0.012122	0.170783	-2.67827
NONHSAT184469.1	1.245199	1.712468	3.678946	0.01213	0.170808	-2.67894
NONHSAT172299.1	1.002819	1.603203	3.678691	0.012133	0.170823	-2.67924
NONHSAT162819.1	2.167894	2.762083	3.678368	0.012138	0.170837	-2.67962
MSTRG.38860.1	1.692877	2.475849	3.678055	0.012142	0.170848	-2.67999
NONHSAT216967.1	-1.1883	2.732648	-3.6772	0.012154	0.170886	-2.68099
ENST00000458316.1	-1.02605	1.997592	-3.67711	0.012155	0.170886	-2.6811
NONHSAT200930.1	1.364244	5.070775	3.676209	0.012167	0.171033	-2.68215
NONHSAT155835.1	1.312417	3.023475	3.675445	0.012177	0.171106	-2.68305
NONHSAT152995.1	1.28654	3.212007	3.674101	0.012196	0.171183	-2.68463
NONHSAT202245.1	-1.46378	1.971568	-3.67306	0.01221	0.1713	-2.68585
NR_121565	-1.08787	2.028689	-3.67225	0.012221	0.171384	-2.6868
ENST00000411862.1	1.141555	3.730665	3.672116	0.012223	0.171386	-2.68696
NONHSAT168602.1	1.315282	3.314586	3.671929	0.012225	0.171393	-2.68718
lnc-ZNF514-2:1	1.679764	2.440993	3.670986	0.012238	0.171412	-2.68828
NONHSAT168020.1	1.540686	2.599768	3.670446	0.012246	0.171457	-2.68892
lnc-NPEPPS-1:1	1.523836	2.783172	3.669926	0.012253	0.171493	-2.68953
lnc-METTL6-3:1	1.189079	2.610905	3.66951	0.012258	0.171493	-2.69001
ENST00000668975.1	1.976846	2.696269	3.66944	0.012259	0.171493	-2.6901
LINC02100:18	2.045657	2.413592	3.668488	0.012272	0.171586	-2.69122
ENST00000563991.1	1.464473	3.436429	3.667494	0.012286	0.171664	-2.69238
NONHSAT157991.1	1.106684	2.19651	3.667434	0.012287	0.171664	-2.69245
NONHSAT183140.1	1.081369	3.961588	3.666477	0.0123	0.171778	-2.69358
NONHSAT206175.1	1.524017	2.08841	3.666463	0.0123	0.171778	-2.69359
T013641	-1.24946	2.315205	-3.66544	0.012314	0.171856	-2.6948
MSTRG.34703.1	2.02759	2.825224	3.665046	0.01232	0.171856	-2.69526
MSTRG.54468.1	1.908861	2.864844	3.664945	0.012321	0.171856	-2.69538
lnc-THBS2-8:1	1.078944	3.414749	3.663988	0.012334	0.171864	-2.6965
lnc-EDNRB-6:1	-1.20524	4.157278	-3.66391	0.012335	0.171864	-2.69659
NONHSAT216874.1	-1.48188	6.462444	-3.66281	0.01235	0.171932	-2.69788
lnc-SNX11-9:1	-1.50622	2.410436	-3.66022	0.012386	0.172114	-2.70093
lnc-APOL2-5:1	1.023739	3.85227	3.658857	0.012405	0.172231	-2.70253
lnc-ROCK1-2:2	-1.50914	2.297176	-3.65843	0.012411	0.172231	-2.70303
T306347	-1.06527	9.198923	-3.6582	0.012414	0.172231	-2.7033
ENST00000664888.1	1.134229	1.90498	3.658177	0.012415	0.172231	-2.70333
NONHSAT184521.1	1.106568	1.800871	3.657432	0.012425	0.172333	-2.70421
NONHSAT163191.1	-1.46471	3.914067	-3.65741	0.012425	0.172333	-2.70424
lnc-TTLL7-11:1	1.119207	3.430483	3.656409	0.012439	0.172359	-2.70541
lnc-FAM210A-7:1	1.831618	2.008274	3.655687	0.012449	0.172427	-2.70626
NONHSAT163663.1	1.107175	1.905002	3.655489	0.012452	0.172432	-2.70649
NONHSAT193636.1	-1.25082	2.136738	-3.65288	0.012488	0.172732	-2.70956
MSTRG.59825.1	1.385045	3.962054	3.652381	0.012495	0.172757	-2.71015
MSTRG.36424.3	1.438714	4.214514	3.649791	0.012532	0.172959	-2.7132
NONHSAT180304.1	2.010831	2.726591	3.649054	0.012542	0.173003	-2.71407
ENST00000419745.1	1.08738	2.937393	3.648892	0.012544	0.173003	-2.71426

NONHSAT167604.1	-1.15583	2.063332	-3.64735	0.012566	0.173092	-2.71607
MSTRG.40712.1	1.376296	1.802948	3.646877	0.012573	0.173137	-2.71663
lnc-CRB1-2:1	-1.4378	3.064803	-3.64628	0.012581	0.173181	-2.71733
lnc-BMP6-16:1	1.307463	2.118622	3.645349	0.012594	0.173259	-2.71843
MSTRG.21431.1	-1.05601	3.0969	-3.64479	0.012602	0.173259	-2.71909
ENST00000498967.3	1.396547	2.156281	3.644253	0.01261	0.173261	-2.71972
NONHSAT187841.1	1.486279	4.025111	3.642296	0.012637	0.173412	-2.72203
MSTRG.37092.5	1.319293	3.241691	3.639916	0.012671	0.173563	-2.72483
lnc-DCBLD1-2:1	-1.91036	4.150235	-3.63963	0.012675	0.173563	-2.72517
ENST00000606010.1	-1.50415	2.15255	-3.63957	0.012676	0.173563	-2.72524
ENST00000515522.1	1.975574	2.890412	3.639122	0.012682	0.173563	-2.72577
ENST00000649558.1	-1.65471	2.597932	-3.6391	0.012683	0.173563	-2.7258
NONHSAT171233.1	1.297111	2.339817	3.63856	0.01269	0.173596	-2.72643
NR_024338	-1.08339	4.968921	-3.6372	0.01271	0.173768	-2.72804
lnc-VWA8-7:1	-1.03274	2.464248	-3.63613	0.012725	0.173819	-2.72929
ENST00000503757.1	-1.0642	2.351925	-3.63607	0.012726	0.173819	-2.72936
NONHSAT198994.1	1.307392	3.145279	3.636062	0.012726	0.173819	-2.72938
NONHSAT180745.1	1.372019	2.285654	3.635843	0.012729	0.173819	-2.72964
lnc-IL1RL1-3:1	-1.58662	2.764905	-3.63452	0.012748	0.173936	-2.7312
MSTRG.59558.1	1.317947	2.500428	3.634297	0.012751	0.173956	-2.73146
NONHSAT217976.1	-1.09641	4.490974	-3.63212	0.012782	0.174246	-2.73403
MSTRG.28053.1	1.074087	1.632048	3.63174	0.012788	0.174246	-2.73448
lnc-ARF6-14:1	-1.68189	2.756668	-3.63033	0.012808	0.1743	-2.73614
lnc-TMEM155-2:1	-1.64886	5.939865	-3.63013	0.012811	0.1743	-2.73638
T203653	1.713136	3.035482	3.62899	0.012827	0.174452	-2.73772
MSTRG.52953.1	1.849993	2.694115	3.628818	0.01283	0.174462	-2.73793
ENST00000557426.1	1.332722	4.982013	3.628236	0.012838	0.174553	-2.73861
NONHSAT160239.1	-1.14534	2.316355	-3.6281	0.01284	0.174556	-2.73877
ENST00000411616.1	1.214491	2.153878	3.627203	0.012853	0.174627	-2.73983
ENST00000669320.1	-1.0191	2.591312	-3.62714	0.012854	0.174627	-2.73991
NONHSAT195570.1	1.352639	2.174815	3.626845	0.012858	0.174658	-2.74026
NONHSAT180674.1	1.042602	3.15518	3.626742	0.01286	0.174658	-2.74038
MSTRG.34112.1	1.596687	1.888633	3.625515	0.012877	0.174828	-2.74183
lnc-CSMD2-2:1	-1.30849	2.777458	-3.62484	0.012887	0.174876	-2.74263
MSTRG.44184.1	-1.25291	2.527827	-3.62467	0.01289	0.174876	-2.74283
NONHSAT219667.1	1.69838	2.401601	3.623973	0.0129	0.174919	-2.74365
NONHSAT160531.1	1.537088	3.41393	3.622927	0.012915	0.17496	-2.74489
NONHSAT211735.1	-1.07048	3.729882	-3.62124	0.012939	0.175046	-2.74688
NONHSAT167072.1	1.089097	3.509387	3.619577	0.012964	0.175196	-2.74884
NONHSAT158272.1	1.034911	3.668101	3.617089	0.013	0.175452	-2.75179
NONHSAT185048.1	1.083887	2.148621	3.616567	0.013008	0.175503	-2.7524
NONHSAT203791.1	-1.47866	1.880194	-3.61579	0.013019	0.175503	-2.75332
ENST00000653239.1	-1.33719	2.038091	-3.61572	0.01302	0.175503	-2.75341
ENST00000439849.1	1.32891	1.722222	3.615044	0.01303	0.175503	-2.75421
NONHSAT202843.1	1.176528	2.006156	3.614846	0.013033	0.175503	-2.75444
MSTRG.13659.1	-1.07959	1.738589	-3.61469	0.013035	0.175503	-2.75462
ENST00000531347.1	1.23495	4.089484	3.613933	0.013046	0.175522	-2.75552
NONHSAT187821.1	-1.01353	1.876114	-3.61392	0.013046	0.175522	-2.75553

MSTRG.50048.2	-1.41768	2.483384	-3.61279	0.013063	0.175667	-2.75687
NONHSAT158528.1	-1.47939	2.606889	-3.61233	0.01307	0.175689	-2.75742
lnc-MS4A8-17:5	-1.28064	2.235777	-3.61044	0.013098	0.175899	-2.75966
NONHSAT201104.1	1.991449	2.886985	3.610405	0.013098	0.175899	-2.7597
ENST00000537032.1	1.311324	3.417778	3.609739	0.013108	0.175943	-2.76048
NONHSAT169238.1	1.218423	3.874772	3.609485	0.013112	0.175947	-2.76078
NR_125852	1.596038	2.355132	3.609105	0.013117	0.175975	-2.76123
lnc-HSDL2-3:1	-1.649	3.030719	-3.60723	0.013145	0.17616	-2.76345
lnc-TBC1D32-4:1	-1.08395	7.055313	-3.60699	0.013149	0.17616	-2.76373
ENST00000656923.1	1.141826	1.934758	3.60656	0.013155	0.176222	-2.76425
NONHSAT173961.1	1.072785	1.631861	3.604402	0.013187	0.176428	-2.7668
ENST00000512245.1	-1.0939	2.269235	-3.60409	0.013192	0.176428	-2.76718
T038690	-1.06119	1.703876	-3.60358	0.013199	0.176428	-2.76777
NONHSAT187390.1	-1.0715	2.228325	-3.60334	0.013203	0.176428	-2.76806
NONHSAT161166.1	-1.67414	2.461854	-3.60332	0.013203	0.176428	-2.76809
NONHSAT170145.1	1.628511	2.397436	3.603287	0.013204	0.176428	-2.76813
NONHSAT201834.1	1.169307	1.981735	3.60248	0.013216	0.176471	-2.76908
ENST00000648279.1	-1.75364	3.050153	-3.60102	0.013237	0.17658	-2.77081
NONHSAT177713.1	1.583513	2.249927	3.600714	0.013242	0.17658	-2.77117
lnc-CTNNA1-4:2	1.195708	6.558992	3.600555	0.013244	0.17658	-2.77136
NONHSAT211356.1	-1.19686	8.920102	-3.60035	0.013247	0.17658	-2.77161
lnc-ANKRD45-3:3	1.42814	3.63372	3.59796	0.013283	0.176748	-2.77444
T296652	-1.69269	2.442106	-3.59793	0.013284	0.176748	-2.77448
NONHSAT196317.1	1.116787	1.694347	3.597053	0.013297	0.176864	-2.77552
lnc-PLPP3-2:5	1.245791	3.273054	3.596925	0.013299	0.176864	-2.77567
NONHSAT217987.1	-1.06486	2.489814	-3.59684	0.0133	0.176864	-2.77577
T236780	-1.12403	5.465605	-3.59582	0.013315	0.176942	-2.77698
NONHSAT177087.1	1.192343	1.98419	3.595665	0.013317	0.176942	-2.77716
MSTRG.59620.9	1.103142	1.764337	3.594521	0.013335	0.176947	-2.77852
NONHSAT220102.1	-1.70308	2.806585	-3.59447	0.013335	0.176947	-2.77857
lnc-SLC15A4-23:1	-1.05052	9.554245	-3.59355	0.013349	0.176976	-2.77967
lnc-ZCCHC17-12:1	-1.16367	1.841073	-3.59344	0.013351	0.176976	-2.7798
NONHSAT177603.1	-1.41379	8.586375	-3.59319	0.013355	0.176976	-2.78009
NONHSAT154326.1	-1.01314	2.075656	-3.59292	0.013359	0.176976	-2.78042
lnc-DAZ1-166:2	-1.54335	2.801445	-3.59186	0.013375	0.17702	-2.78167
lnc-SLC39A11-6:1	1.029532	2.170983	3.591295	0.013383	0.17702	-2.78235
NONHSAT205713.1	2.206102	3.343844	3.591111	0.013386	0.17702	-2.78256
lnc-THAP3-4:1	-1.09816	2.100132	-3.58895	0.013419	0.177232	-2.78513
lnc-FOXN4-2:1	1.34384	4.052838	3.588627	0.013424	0.177232	-2.78551
lnc-DISC1-1:2	-1.06918	6.668332	-3.58834	0.013428	0.177244	-2.78586
ENST00000437837.1	-1.57109	2.050124	-3.58622	0.01346	0.177396	-2.78838
NONHSAT208060.1	-1.12197	1.901528	-3.58379	0.013497	0.17765	-2.79125
NONHSAT218363.1	-1.46768	2.588999	-3.5819	0.013526	0.177908	-2.79351
GPC6-AS2:1	1.299926	2.166324	3.580487	0.013548	0.1781	-2.79518
NONHSAT224442.1	1.159022	1.745809	3.578643	0.013576	0.178285	-2.79738
ENST00000414776.1	1.115689	1.921937	3.578244	0.013582	0.178305	-2.79785
lnc-ZC3H12B-2:4	1.297084	2.597348	3.577444	0.013594	0.178393	-2.7988
lnc-INAVA-1:1	-1.0048	4.143854	-3.57681	0.013604	0.178424	-2.79956

lnc-FAM50B-4:2	1.529011	2.189644	3.574316	0.013643	0.178556	-2.80252
MSTRG.623.1	1.844599	3.364683	3.573687	0.013652	0.178556	-2.80327
NR_027713	-1.56518	3.082906	-3.57364	0.013653	0.178556	-2.80333
NONHSAT218169.1	1.80256	1.943846	3.572469	0.013671	0.178611	-2.80472
NONHSAT153503.1	1.51365	2.796028	3.57221	0.013675	0.178611	-2.80503
NONHSAT211452.1	-1.2536	2.287592	-3.57133	0.013689	0.178616	-2.80607
lnc-LRRTM1-2:2	-1.47568	3.056266	-3.57125	0.01369	0.178616	-2.80617
NR_026835	1.134214	3.749899	3.570784	0.013697	0.178616	-2.80672
lnc-SLC6A15-2:1	1.833227	2.550342	3.56916	0.013722	0.178742	-2.80866
NONHSAT197807.1	1.521467	1.779432	3.568497	0.013733	0.178758	-2.80945
MSTRG.68181.1	-1.3944	3.058545	-3.56848	0.013733	0.178758	-2.80946
NONHSAT158418.1	1.635548	2.017842	3.567836	0.013743	0.178836	-2.81023
NONHSAT164332.1	-1.08208	1.821517	-3.56732	0.013751	0.178836	-2.81085
NONHSAT206493.1	-1.2039	5.989072	-3.56721	0.013753	0.178836	-2.81098
NONHSAT210319.1	-1.3932	3.205395	-3.56685	0.013758	0.178869	-2.8114
NONHSAT206392.1	-2.64211	2.342377	-3.56217	0.013831	0.179351	-2.81698
NONHSAT192321.1	-1.4595	3.333334	-3.56118	0.013847	0.179411	-2.81817
ENST00000429450.1	-1.45844	2.706131	-3.56085	0.013852	0.179411	-2.81856
NONHSAT174611.1	-1.08745	4.245273	-3.56081	0.013853	0.179411	-2.8186
ENST00000451230.1	1.554676	3.273254	3.560403	0.013859	0.179437	-2.81909
lnc-KLHL1-10:1	-1.81296	3.424641	-3.56032	0.01386	0.179437	-2.81919
lnc-GADD45B-1:1	2.073678	2.616241	3.560246	0.013862	0.179437	-2.81927
MSTRG.41122.1	1.86764	3.306225	3.560163	0.013863	0.179437	-2.81937
ENST00000547084.1	1.976816	4.136076	3.559796	0.013869	0.179465	-2.81981
NONHSAT205486.1	-1.49714	2.968549	-3.55949	0.013874	0.179465	-2.82018
MSTRG.38772.2	-1.13042	1.740647	-3.55753	0.013904	0.179484	-2.82251
lnc-OR5H1-1:1	-1.08837	2.179077	-3.55743	0.013906	0.179484	-2.82263
lnc-VASP-1:1	1.43202	3.066536	3.557338	0.013907	0.179484	-2.82274
lnc-FAM49A-1:1	-1.19034	2.215342	-3.55718	0.01391	0.179484	-2.82294
lnc-RPL21-4:1	1.337073	3.499824	3.555272	0.01394	0.179674	-2.82521
NONHSAT157701.1	1.270132	1.735848	3.554319	0.013955	0.179761	-2.82634
lnc-HMGCLL1-2:1	1.310348	3.03237	3.55426	0.013956	0.179761	-2.82641
ENST00000623971.3	1.279497	2.329604	3.55301	0.013976	0.179859	-2.8279
T258078	-1.19329	4.158373	-3.55044	0.014017	0.1802	-2.83097
NONHSAT170849.1	-1.03495	4.869949	-3.55	0.014024	0.180228	-2.8315
lnc-ACSBG2-2:3	1.65355	4.805025	3.54957	0.01403	0.180262	-2.83201
lnc-ZNF736-2:1	1.225164	1.740517	3.549467	0.014032	0.180262	-2.83213
ENST00000613388.2	1.030106	5.219996	3.54926	0.014035	0.180281	-2.83238
MSTRG.56542.1	1.036923	3.848715	3.548686	0.014045	0.180284	-2.83307
MSTRG.31863.1	1.267012	3.901611	3.547894	0.014057	0.180375	-2.83401
LINC00640:8	1.109052	3.44731	3.547317	0.014066	0.180375	-2.8347
lnc-NR2F2-9:1	1.069157	4.206313	3.547253	0.014067	0.180375	-2.83478
lnc-PEX10-6:3	-1.11542	5.763554	-3.54675	0.014076	0.180375	-2.83538
lnc-CYSLTR2-2:2	1.366835	3.848162	3.546675	0.014077	0.180375	-2.83547
MSTRG.70570.2	-1.01768	1.826406	-3.54638	0.014081	0.180411	-2.83582
NR_110558	1.737554	2.383129	3.545675	0.014093	0.180411	-2.83666
T095343	1.264555	1.804362	3.545575	0.014094	0.180411	-2.83678
MSTRG.23904.1	2.080279	3.341355	3.545409	0.014097	0.180411	-2.83698

NONHSAT206059.1	1.089739	1.959453	3.544442	0.014112	0.18043	-2.83813
NONHSAT187409.1	1.28983	5.002933	3.544392	0.014113	0.18043	-2.83819
lnc-TADA2B-6:1	1.404138	2.791769	3.544199	0.014116	0.180447	-2.83842
ENST00000630100.1	1.337651	1.850087	3.544078	0.014118	0.180449	-2.83857
lnc-WDPCP-4:1	1.004179	6.579133	3.543777	0.014123	0.180464	-2.83893
MSTRG.47262.1	1.081951	1.617591	3.543668	0.014125	0.180464	-2.83906
NONHSAT155354.1	1.670655	2.201654	3.542014	0.014151	0.180575	-2.84103
MSTRG.47445.1	-1.06252	3.299376	-3.54071	0.014172	0.180662	-2.84259
ENST00000412918.1	1.276367	1.963781	3.539925	0.014185	0.180662	-2.84353
NONHSAT171171.1	1.670974	2.68341	3.538095	0.014215	0.180832	-2.84572
NONHSAT158563.1	1.46909	3.07531	3.537242	0.014228	0.180984	-2.84674
NONHSAT190143.1	1.214784	2.875414	3.535172	0.014262	0.181074	-2.84921
T348942	-1.0835	2.205191	-3.53492	0.014266	0.181076	-2.84952
NONHSAT153293.1	1.253945	3.213809	3.533605	0.014287	0.181245	-2.85109
ENST00000449928.1	-1.26151	3.882756	-3.53354	0.014288	0.181245	-2.85116
NONHSAT161030.1	1.619211	4.297819	3.53309	0.014296	0.181254	-2.8517
MSTRG.69884.1	1.546547	2.488121	3.532883	0.014299	0.181254	-2.85195
MSTRG.57291.1	-1.4223	3.309461	-3.53283	0.0143	0.181254	-2.85201
ENST00000454526.1	1.08938	2.942045	3.532276	0.014309	0.181266	-2.85268
ENST00000654462.1	-1.06442	2.101837	-3.53171	0.014318	0.181327	-2.85336
NONHSAT186984.1	1.309175	2.90095	3.530822	0.014333	0.181375	-2.85442
MSTRG.41214.11	-1.07066	4.36861	-3.5301	0.014345	0.181428	-2.85528
lnc-SCIN-4:1	-1.36173	2.273913	-3.5289	0.014364	0.181524	-2.85672
lnc-YEATS4-3:1	-1.15448	4.298928	-3.52888	0.014364	0.181524	-2.85674
lnc-TUBB2B-3:1	1.376562	2.938843	3.525137	0.014426	0.181987	-2.86122
lnc-C6orf118-6:1	1.242115	1.699336	3.524266	0.01444	0.182092	-2.86226
NONHSAT158492.1	1.240703	1.869593	3.523725	0.014449	0.182121	-2.86291
MSTRG.46060.1	-1.11009	3.836886	-3.52173	0.014482	0.182253	-2.86531
NONHSAT193401.1	-1.08615	2.977001	-3.52127	0.01449	0.182253	-2.86586
NONHSAT190154.1	1.290112	1.945069	3.521054	0.014493	0.182253	-2.86611
lnc-SP3-8:1	-1.28386	2.21354	-3.52054	0.014501	0.182253	-2.86673
NONHSAT148274.1	-1.27912	1.979429	-3.51986	0.014513	0.182369	-2.86754
NONHSAT164867.1	-1.46927	2.449225	-3.51884	0.01453	0.182452	-2.86876
lnc-ZNF487-7:1	-1.40465	3.03381	-3.51854	0.014535	0.182486	-2.86913
NONHSAT214759.1	-1.02479	3.310062	-3.51723	0.014556	0.18263	-2.8707
MSTRG.38157.1	1.52445	2.285345	3.51595	0.014578	0.182775	-2.87223
lnc-GATA3-2:1	1.828011	2.318595	3.514295	0.014605	0.182807	-2.87421
NONHSAT164821.1	1.070179	3.212605	3.514155	0.014607	0.182807	-2.87438
NR_038974	-1.6204	4.092433	-3.51361	0.014617	0.182844	-2.87504
NONHSAT214838.1	1.290217	1.712786	3.513549	0.014618	0.182844	-2.87511
LINC02290:25	-1.66735	2.243612	-3.5129	0.014628	0.182912	-2.87588
lnc-IL2RA-7:1	-1.62818	2.487602	-3.51254	0.014634	0.182929	-2.87632
lnc-DLGAP1-7:1	-1.38318	4.883901	-3.51184	0.014646	0.182987	-2.87717
ENST00000424355.1	-1.1372	2.379338	-3.51109	0.014659	0.182987	-2.87806
NONHSAT161171.1	-1.15899	2.242428	-3.51103	0.01466	0.182987	-2.87814
NONHSAT187073.1	-1.44796	3.104999	-3.51102	0.01466	0.182987	-2.87815
MSTRG.10077.1	-1.12825	2.988524	-3.5087	0.014699	0.18329	-2.88093
NONHSAT164555.1	1.385991	2.61709	3.508163	0.014708	0.18338	-2.88157

lnc-SYCP2-3:1	-1.36097	3.020062	-3.5051	0.014759	0.183667	-2.88525
MSTRG.58741.1	1.323632	1.722422	3.505016	0.014761	0.183667	-2.88535
MSTRG.47492.1	1.143893	5.359982	3.504642	0.014767	0.183667	-2.8858
ENST00000438789.1	-1.23896	2.421328	-3.5043	0.014773	0.183667	-2.88621
NONHSAT186941.1	-1.12276	4.519798	-3.50398	0.014778	0.183689	-2.88659
lnc-SOCS6-7:1	-1.23652	4.455044	-3.50384	0.01478	0.183695	-2.88676
NONHSAT167442.1	-1.02843	4.418896	-3.50294	0.014796	0.183817	-2.88784
ENST00000665121.1	1.042797	3.010697	3.502388	0.014805	0.183888	-2.88851
NONHSAT205764.1	-1.05862	3.007635	-3.50115	0.014826	0.18408	-2.88999
lnc-CXorf51A-14:1	-1.15031	2.083641	-3.5006	0.014835	0.184128	-2.89065
NONHSAT167478.1	1.098077	1.916299	3.497706	0.014884	0.184355	-2.89413
lnc-PRELID2-2:2	1.221181	3.300166	3.497402	0.01489	0.184355	-2.8945
NONHSAT190308.1	1.194407	2.011502	3.497348	0.014891	0.184355	-2.89456
NONHSAT193133.1	1.157645	2.773996	3.497196	0.014893	0.184355	-2.89474
ENST00000614509.1	-1.26442	2.386309	-3.4949	0.014932	0.184663	-2.89751
NONHSAT176802.1	1.512483	2.42111	3.491474	0.014991	0.185035	-2.90163
lnc-ATP6V1E1-3:1	-1.62876	2.480102	-3.49067	0.015005	0.185093	-2.90259
lnc-ATF7IP2-6:1	1.075895	1.699811	3.490425	0.015009	0.185093	-2.90289
ENST00000560337.1	-1.75786	2.715035	-3.49013	0.015014	0.185093	-2.90325
T351259	1.239449	1.722997	3.488897	0.015035	0.185157	-2.90473
lnc-CBR1-1:3	1.094037	1.784838	3.487774	0.015055	0.185208	-2.90608
lnc-IRS4-1:1	1.535149	2.698342	3.486802	0.015071	0.185301	-2.90725
NONHSAT174416.1	-1.04546	3.927917	-3.48644	0.015078	0.185333	-2.90769
ENST00000658686.1	-1.26024	2.178965	-3.48582	0.015088	0.185442	-2.90844
lnc-RNF113B-5:1	1.853999	2.953942	3.485586	0.015092	0.185469	-2.90871
E2F3-IT1:1	-1.17724	4.489231	-3.48543	0.015095	0.18548	-2.9089
NONHSAT205672.1	-1.06637	2.903776	-3.48496	0.015103	0.185521	-2.90947
lnc-TUBGCP3-6:4	1.506457	7.502348	3.4843	0.015115	0.185584	-2.91026
NR_110699	1.689119	2.756239	3.483149	0.015134	0.185607	-2.91165
NONHSAT202311.1	1.47122	1.789676	3.482608	0.015144	0.185627	-2.9123
MSTRG.26789.29	-1.17725	2.471576	-3.4817	0.01516	0.185686	-2.91339
MSTRG.6595.1	-1.36542	1.854238	-3.48105	0.015171	0.185729	-2.91417
NONHSAT216550.1	1.383393	3.341553	3.480936	0.015173	0.185729	-2.91431
NONHSAT161150.1	-1.77593	3.036673	-3.48078	0.015176	0.185729	-2.9145
lnc-ARID1B-4:1	-1.58524	4.638487	-3.47865	0.015213	0.185933	-2.91706
NONHSAT160698.1	1.076451	4.529705	3.478407	0.015217	0.185933	-2.91736
lnc-RAX-2:1	-1.10247	1.861888	-3.47775	0.015228	0.185934	-2.91815
ENST00000648611.1	-1.13991	2.006914	-3.47739	0.015235	0.185948	-2.91858
MSTRG.8157.30	1.173062	2.092586	3.476615	0.015248	0.18601	-2.91952
MSTRG.17146.1	1.18115	2.008326	3.475867	0.015261	0.18601	-2.92042
NONHSAT214459.1	-1.32142	2.4791	-3.4757	0.015264	0.186018	-2.92062
lnc-SHPRH-6:1	1.235667	2.307241	3.474623	0.015283	0.186093	-2.92192
NONHSAT157869.1	-1.07545	1.786264	-3.47445	0.015286	0.186093	-2.92213
NONHSAT178924.1	1.321689	2.167728	3.474354	0.015288	0.186093	-2.92224
NONHSAT191702.1	1.21283	1.832807	3.474202	0.01529	0.186093	-2.92243
MSTRG.47239.1	1.942273	2.607016	3.474185	0.015291	0.186093	-2.92245
MSTRG.54968.2	-1.33936	2.437402	-3.47141	0.015339	0.186419	-2.92579
LINC01226:10	1.378644	3.925322	3.471128	0.015344	0.186432	-2.92613

NONHSAT186686.1	-1.47637	2.688269	-3.47089	0.015349	0.186439	-2.92643
lnc-LRRC49-16:1	-1.26599	2.32835	-3.47025	0.01536	0.186477	-2.92719
lnc-MAD2L1BP-1:1	-1.14896	3.140677	-3.46826	0.015395	0.186562	-2.9296
ENST00000658605.1	1.947087	2.956788	3.467683	0.015405	0.186562	-2.93029
NONHSAT154487.1	1.030538	1.572736	3.467428	0.01541	0.186562	-2.9306
NONHSAT176814.1	1.065947	2.516628	3.46678	0.015421	0.186645	-2.93138
lnc-KLHL25-1:5	1.078627	3.106216	3.464973	0.015453	0.186716	-2.93356
lnc-ALDH8A1-4:1	1.126359	1.585744	3.463846	0.015473	0.186716	-2.93492
T309954	1.364235	2.291763	3.463585	0.015478	0.186716	-2.93524
NONHSAT194939.1	1.310906	2.137651	3.463529	0.015479	0.186716	-2.9353
lnc-GTSF1L-8:1	1.751762	1.977212	3.463424	0.015481	0.186716	-2.93543
NONHSAT212826.1	1.555694	2.143142	3.463055	0.015487	0.186773	-2.93588
NONHSAT220287.1	1.018364	4.14392	3.462805	0.015492	0.186787	-2.93618
MSTRG.53962.1	1.066822	2.016951	3.462446	0.015498	0.186836	-2.93661
lnc-SHROOM1-2:1	1.43175	2.439781	3.460798	0.015528	0.186876	-2.9386
lnc-GNL1-3:2	-1.14201	1.978478	-3.45968	0.015547	0.186963	-2.93995
LINC01387:7	1.091628	1.642668	3.459311	0.015554	0.186974	-2.9404
NONHSAT218665.1	1.20397	1.648432	3.45847	0.015569	0.187043	-2.94141
lnc-PRKCQ-7:1	1.155289	4.686243	3.458351	0.015571	0.187047	-2.94156
NR_024591	1.724638	2.301329	3.458071	0.015576	0.187085	-2.9419
T342810	1.131136	4.853084	3.457562	0.015585	0.18712	-2.94251
NONHSAT223796.1	1.001981	2.2198	3.457332	0.01559	0.18712	-2.94279
NONHSAT163852.1	1.486777	2.239703	3.456485	0.015605	0.187158	-2.94381
NONHSAT187508.1	-1.00486	3.381618	-3.45614	0.015611	0.187188	-2.94423
lnc-CDCP1-1:1	1.485277	4.451599	3.455815	0.015617	0.187214	-2.94462
ENST00000515623.1	-1.44566	2.102184	-3.45526	0.015627	0.187263	-2.94529
ENST00000440413.1	1.20395	4.301825	3.454288	0.015644	0.187431	-2.94647
MSTRG.62115.2	1.18803	1.648544	3.454048	0.015649	0.187438	-2.94676
ENST00000436340.2	1.16636	2.924418	3.453876	0.015652	0.187453	-2.94697
NONHSAT192899.1	-1.53542	2.555502	-3.4521	0.015684	0.187609	-2.94911
lnc-PANK3-17:1	-1.31461	7.283283	-3.45203	0.015685	0.187609	-2.9492
NONHSAT196990.1	1.141829	1.71786	3.451903	0.015687	0.187609	-2.94935
NONHSAT179394.1	1.607394	2.738991	3.451813	0.015689	0.187609	-2.94946
ENST00000621571.1	-1.1674	2.996559	-3.45119	0.0157	0.187624	-2.95021
ENST00000582578.2	1.128386	3.558194	3.451127	0.015701	0.187624	-2.95029
ENST00000423637.1	1.417346	3.399601	3.450706	0.015709	0.187651	-2.9508
NONHSAT177853.1	1.443955	4.01657	3.450178	0.015718	0.187651	-2.95144
ENST00000671190.1	1.162661	3.49544	3.449495	0.015731	0.187685	-2.95226
lnc-TUBE1-3:1	1.20202	4.687814	3.448803	0.015743	0.187767	-2.9531
NONHSAT186529.1	1.018922	2.634127	3.448655	0.015746	0.187767	-2.95328
lnc-FAM193B-4:1	-2.19695	3.505321	-3.44839	0.015751	0.187792	-2.9536
lnc-LINS1-5:1	-1.02954	2.713365	-3.44602	0.015794	0.188018	-2.95647
NONHSAT210396.1	1.103145	4.307495	3.443856	0.015833	0.188128	-2.95909
ENST00000558370.1	1.169874	2.228783	3.443186	0.015845	0.188128	-2.9599
NONHSAT156336.1	1.065358	1.819654	3.443165	0.015846	0.188128	-2.95992
T191042	1.584561	1.867103	3.442566	0.015857	0.188144	-2.96065
NONHSAT193336.1	1.224125	1.989191	3.44181	0.015871	0.188174	-2.96157
MSTRG.65759.1	1.446094	1.985206	3.441686	0.015873	0.188174	-2.96172

lnc-ACTL7A-3:1	-1.05656	9.796143	-3.44052	0.015894	0.188289	-2.96313
MSTRG.29239.7	-1.63887	2.646771	-3.44009	0.015902	0.188289	-2.96365
NONHSAT171077.1	1.08409	1.57664	3.440069	0.015902	0.188289	-2.96367
MSTRG.37172.1	1.095685	3.298486	3.438267	0.015935	0.18844	-2.96586
NONHSAT206952.1	1.26972	2.051446	3.437903	0.015942	0.18844	-2.9663
MSTRG.68674.1	1.051395	3.03571	3.437901	0.015942	0.18844	-2.9663
lnc-TIMM8A-2:1	-1.31856	5.051019	-3.43699	0.015959	0.188493	-2.96741
NONHSAT191821.1	-1.10728	4.07917	-3.43532	0.01599	0.188698	-2.96943
ENST00000414544.1	-1.07928	3.382399	-3.4347	0.016001	0.188698	-2.97018
NONHSAT166072.1	1.213488	2.599254	3.433996	0.016014	0.18879	-2.97103
NONHSAT154722.1	-1.00284	1.773636	-3.43272	0.016038	0.188894	-2.97258
ENST00000570443.2	1.082049	1.968586	3.432647	0.016039	0.188894	-2.97267
lnc-HMGXB4-1:1	-1.10612	2.764302	-3.43105	0.016069	0.189054	-2.97461
NONHSAT157713.1	-1.41013	3.70442	-3.43096	0.01607	0.189054	-2.97471
NONHSAT224347.1	1.59801	3.221907	3.430227	0.016084	0.189123	-2.9756
NONHSAT214995.1	-1.50228	2.751076	-3.43014	0.016086	0.189123	-2.97571
lnc-GPBP1-9:2	1.086543	5.689626	3.430001	0.016088	0.189123	-2.97587
NONHSAT210413.1	1.454915	2.76062	3.428917	0.016108	0.189147	-2.97719
NONHSAT203008.1	1.940238	2.010788	3.428636	0.016113	0.189147	-2.97753
lnc-EPB41L1-1:4	1.343835	2.288343	3.428573	0.016115	0.189147	-2.97761
NONHSAT174101.1	-1.22334	9.056842	-3.42801	0.016125	0.189197	-2.97829
NONHSAT205755.1	1.546919	2.137897	3.427983	0.016126	0.189197	-2.97832
lnc-KCNV2-6:1	1.087748	2.296249	3.426439	0.016154	0.189341	-2.9802
NONHSAT183014.1	1.152373	3.193484	3.425562	0.016171	0.189378	-2.98126
NONHSAT190467.1	-1.33078	4.889004	-3.42532	0.016175	0.189405	-2.98155
NONHSAT215067.1	-1.26565	2.162467	-3.42448	0.016191	0.189545	-2.98257
MSTRG.11344.1	-1.09889	2.651774	-3.42417	0.016197	0.189545	-2.98295
lnc-PPP1R2C-10:1	1.106207	3.558062	3.423779	0.016204	0.189564	-2.98342
NR_125388	1.718068	2.789915	3.423559	0.016208	0.189564	-2.98369
lnc-GCNT4-3:1	-1.0071	8.145247	-3.42343	0.016211	0.189564	-2.98385
NONHSAT202285.1	-1.02144	1.767202	-3.42318	0.016215	0.189564	-2.98415
ENST00000601906.2	-1.25572	1.852851	-3.42283	0.016222	0.189598	-2.98458
MSTRG.69846.1	-1.24817	5.805568	-3.42154	0.016246	0.189773	-2.98614
lnc-C11orf91-4:1	1.672136	1.962812	3.421313	0.01625	0.189773	-2.98642
lnc-ZNF518B-4:1	-1.5496	2.226157	-3.42092	0.016258	0.189773	-2.98689
T269639	-1.61725	3.216258	-3.42037	0.016268	0.189808	-2.98756
MSTRG.13601.1	1.381844	4.86776	3.419129	0.016291	0.189843	-2.98907
lnc-TNKS-2:1	1.857722	2.391296	3.418995	0.016294	0.189843	-2.98923
NONHSAT153879.1	-1.84554	3.758515	-3.4186	0.016301	0.189843	-2.98971
ENST00000433378.1	1.036395	4.659995	3.41841	0.016305	0.189843	-2.98994
ENST00000669105.1	-1.87404	3.210929	-3.4184	0.016305	0.189843	-2.98996
T285662	-1.57133	3.20356	-3.41768	0.016319	0.189936	-2.99083
ENST00000502437.1	1.065895	4.417285	3.416356	0.016344	0.190112	-2.99244
NONHSAT188095.1	1.195783	5.501572	3.415971	0.016351	0.190137	-2.9929
NONHSAT197278.1	1.06139	2.028339	3.41444	0.01638	0.190305	-2.99476
ENST00000654642.1	1.002854	4.852354	3.413514	0.016397	0.190392	-2.99589
ENST00000655406.1	-1.1528	1.890479	-3.412	0.016426	0.190528	-2.99773
MSTRG.21490.1	-1.09125	12.31114	-3.41169	0.016432	0.19054	-2.99811

ENST00000509215.1	1.240653	5.028472	3.411617	0.016433	0.19054	-2.9982
LINC02116:4	-1.1803	5.508675	-3.41083	0.016448	0.190648	-2.99915
NONHSAT195324.1	1.235799	1.893571	3.410464	0.016455	0.190692	-2.9996
lnc-S1PR1-7:1	1.082996	4.174554	3.410038	0.016463	0.190726	-3.00012
MSTRG.38541.1	-1.76373	5.879141	-3.40845	0.016494	0.190954	-3.00204
lnc-FAM13C-4:1	-1.70782	3.177997	-3.40838	0.016495	0.190954	-3.00213
lnc-LY6E-6:1	1.368239	2.041381	3.407583	0.01651	0.191021	-3.0031
NONHSAT170255.1	1.026657	3.664062	3.406643	0.016528	0.191185	-3.00424
NONHSAT221212.1	2.177825	2.816578	3.406153	0.016538	0.191272	-3.00484
lnc-SLC16A9-6:1	1.173803	4.145158	3.40521	0.016556	0.191371	-3.00599
lnc-FAM120B-3:4	1.115812	4.038942	3.405087	0.016558	0.191373	-3.00614
lnc-BTBD3-1:1	1.192398	2.758272	3.405003	0.01656	0.191373	-3.00624
lnc-CMKLR1-2:1	1.113316	3.469108	3.404233	0.016574	0.191456	-3.00718
NONHSAT205205.1	1.430683	4.838213	3.403722	0.016584	0.191503	-3.0078
ENST00000511422.1	-1.12244	1.888129	-3.40357	0.016587	0.191514	-3.00798
ENST00000615692.1	1.46373	2.549995	3.403277	0.016593	0.191558	-3.00834
NONHSAT177786.1	1.152381	1.657242	3.401478	0.016627	0.191815	-3.01053
NR_003347	1.332007	1.761294	3.401332	0.01663	0.191815	-3.01071
MSTRG.35345.7	2.004878	2.784795	3.400861	0.016639	0.191875	-3.01128
NONHSAT161591.1	-1.52394	2.665879	-3.40047	0.016647	0.191918	-3.01176
NONHSAT196199.1	1.461982	3.345854	3.399982	0.016656	0.192005	-3.01235
lnc-GNG4-2:1	-1.06441	8.796449	-3.39825	0.01669	0.192162	-3.01446
ENST00000549710.1	1.424679	2.472357	3.39809	0.016693	0.192162	-3.01465
NONHSAT214929.1	-1.73484	2.581539	-3.39684	0.016717	0.192216	-3.01618
lnc-FAM47B-2:1	-1.12774	7.001593	-3.39656	0.016722	0.192216	-3.01652
lnc-MAL2-1:1	1.301	3.05041	3.394016	0.016772	0.192395	-3.01962
NONHSAT211045.1	1.180753	2.22056	3.38993	0.016852	0.192809	-3.0246
T155545	-1.76353	3.649642	-3.38902	0.016869	0.192983	-3.02571
T114303	-1.36645	3.916284	-3.38856	0.016878	0.193014	-3.02627
NONHSAT199339.1	-1.29038	2.254137	-3.38431	0.016962	0.193535	-3.03144
T057556	1.64966	2.849763	3.383794	0.016972	0.193542	-3.03208
lnc-DTYMK-5:1	1.41741	1.768531	3.383423	0.016979	0.193546	-3.03253
NONHSAT180648.1	1.470039	2.226956	3.383173	0.016984	0.193546	-3.03284
NONHSAT188135.1	1.087201	2.204719	3.383095	0.016986	0.193546	-3.03293
lnc-RRM2B-1:1	-1.78468	2.994729	-3.38026	0.017042	0.193848	-3.03639
lnc-PHLDA3-2:1	1.206273	3.93082	3.38019	0.017043	0.193848	-3.03648
lnc-ERG-4:1	1.515103	1.853143	3.379735	0.017052	0.193848	-3.03703
lnc-WASHC4-3:2	1.200876	2.04243	3.378984	0.017067	0.193848	-3.03795
T219085	1.717106	2.331916	3.378506	0.017076	0.193848	-3.03853
NONHSAT210262.1	1.271496	1.658717	3.377862	0.017089	0.193937	-3.03932
NONHSAT187404.1	1.658099	2.073793	3.377346	0.0171	0.193963	-3.03995
MSTRG.54868.1	1.606417	3.07082	3.375911	0.017128	0.194047	-3.0417
NONHSAT190573.1	-1.20191	4.80187	-3.37454	0.017155	0.194271	-3.04337
MSTRG.61489.1	-1.45753	1.959862	-3.37423	0.017161	0.194284	-3.04375
lnc-AGBL1-5:1	1.005166	5.730754	3.374002	0.017166	0.194284	-3.04403
NONHSAT191738.1	1.182777	1.66879	3.373856	0.017169	0.19429	-3.04421
NONHSAT196874.1	1.024593	2.318726	3.373661	0.017173	0.19429	-3.04445
lnc-CRYBA4-12:1	-1.04968	4.814501	-3.37356	0.017175	0.19429	-3.04457

NONHSAT221538.1	1.776556	3.075028	3.373258	0.017181	0.194299	-3.04494
NONHSAT201822.1	1.078217	2.165656	3.373163	0.017183	0.194299	-3.04506
lnc-ACP4-1:2	1.548836	2.590464	3.372624	0.017194	0.194306	-3.04571
lnc-ANKRD30BL-7:7	1.304906	2.097295	3.372612	0.017194	0.194306	-3.04573
NONHSAT174237.1	-1.57678	2.841193	-3.37175	0.017211	0.194314	-3.04678
ENST00000454380.1	1.034081	1.570005	3.371197	0.017222	0.194417	-3.04746
NONHSAT210401.1	-1.19068	8.722252	-3.37036	0.017239	0.194472	-3.04848
ENST00000609969.1	1.942869	2.048752	3.369408	0.017258	0.194475	-3.04964
NONHSAT197073.1	1.047411	2.145772	3.369308	0.01726	0.194475	-3.04977
NONHSAT172699.1	-1.13052	5.025858	-3.36916	0.017263	0.194475	-3.04995
MSTRG.45522.1	2.104096	5.565859	3.368998	0.017266	0.194475	-3.05014
MSTRG.60557.86	-1.0157	2.431156	-3.36834	0.01728	0.194519	-3.05095
NONHSAT206599.1	-1.30805	1.990024	-3.36594	0.017328	0.194847	-3.05389
NONHSAT218832.1	-1.30142	2.213179	-3.36558	0.017335	0.194863	-3.05433
lnc-CNOT9-1:1	1.671042	2.491947	3.365368	0.017339	0.194888	-3.05458
NONHSAT164049.1	1.122127	1.621197	3.364896	0.017349	0.194888	-3.05516
NONHSAT216824.1	-1.13661	1.787308	-3.36434	0.01736	0.194939	-3.05584
lnc-PEPD-7:1	1.043053	1.60971	3.362595	0.017395	0.195215	-3.05797
lnc-GMDS-14:1	1.407273	1.789831	3.360924	0.017429	0.195421	-3.06002
MSTRG.57019.1	-1.32201	4.244187	-3.36074	0.017433	0.195442	-3.06025
NONHSAT148320.1	1.364364	3.94527	3.359932	0.01745	0.195453	-3.06123
NONHSAT181502.1	1.947415	2.471341	3.3599	0.01745	0.195453	-3.06127
lnc-CYB5R2-3:19	1.202883	3.445543	3.35969	0.017454	0.195453	-3.06153
lnc-STK25-2:1	1.24332	8.63101	3.359247	0.017463	0.195453	-3.06207
ENST00000558572.1	1.433125	2.224651	3.357904	0.017491	0.195628	-3.06371
NONHSAT177890.1	2.240896	2.164613	3.357714	0.017495	0.195628	-3.06395
ENST00000650712.1	-1.32999	3.528741	-3.35735	0.017502	0.195628	-3.0644
lnc-CREBL2-3:1	-1.191	2.735567	-3.35596	0.017531	0.195686	-3.0661
NONHSAT205336.1	-1.10532	6.952081	-3.35531	0.017544	0.195767	-3.06689
NONHSAT224078.1	-1.46719	2.085037	-3.35476	0.017555	0.195827	-3.06756
lnc-RIT2-8:1	-1.33577	3.098673	-3.35282	0.017595	0.196039	-3.06994
NONHSAT184560.1	-1.34573	3.740093	-3.35271	0.017597	0.196039	-3.07008
MSTRG.56321.1	1.104453	1.97897	3.352667	0.017598	0.196039	-3.07013
ENST00000668860.1	1.223291	1.944125	3.352532	0.017601	0.196039	-3.07029
NONHSAT173969.1	1.087048	3.405761	3.35224	0.017607	0.196079	-3.07065
STXBP5-AS1:28	1.675721	2.495865	3.351861	0.017614	0.196079	-3.07111
T337792	-1.26442	8.922402	-3.35169	0.017618	0.196079	-3.07132
NONHSAT182965.1	-1.23632	2.03653	-3.35098	0.017633	0.196129	-3.0722
NONHSAT216334.1	1.250467	3.258274	3.347204	0.01771	0.196626	-3.07682
NONHSAT160068.1	1.281157	1.749046	3.346153	0.017732	0.19678	-3.07811
NONHSAT160949.1	1.157131	1.677538	3.345157	0.017753	0.19686	-3.07933
lnc-KIDINS220-16:1	1.484228	3.504883	3.344818	0.01776	0.196876	-3.07974
T161830	-1.10269	1.821513	-3.3432	0.017793	0.196939	-3.08172
NONHSAT160451.1	1.485054	2.47507	3.34257	0.017806	0.197025	-3.0825
lnc-CTU1-2:5	1.094985	2.217325	3.342545	0.017807	0.197025	-3.08253
lnc-TRPV6-3:1	1.228397	5.834305	3.342157	0.017815	0.197059	-3.08301
MSTRG.44989.1	-1.55754	2.615937	-3.3415	0.017829	0.197136	-3.08381
ENST00000412932.1	-1.39753	1.842618	-3.34073	0.017845	0.197205	-3.08475

MSTRG.17773.1	1.041855	1.608651	3.340086	0.017858	0.197267	-3.08555
ENST00000650410.1	1.239595	2.445869	3.339506	0.01787	0.197371	-3.08626
NONHSAT160717.1	-1.66981	3.076725	-3.33836	0.017894	0.19743	-3.08766
ENST00000512329.2	-1.3856	3.615118	-3.33835	0.017894	0.19743	-3.08768
lnc-AIG1-9:1	1.440569	2.564951	3.336721	0.017928	0.197698	-3.08967
lnc-WNT7B-4:1	1.919615	6.173257	3.33667	0.01793	0.197698	-3.08974
T056970	1.04485	1.947665	3.336253	0.017938	0.197698	-3.09025
lnc-JMJD1C-8:1	-1.19075	2.155691	-3.33576	0.017949	0.197785	-3.09085
NONHSAT160662.1	1.316233	3.176508	3.33393	0.017987	0.198035	-3.0931
lnc-SLC6A14-1:1	1.366729	3.023584	3.33335	0.017999	0.198042	-3.09381
ENST00000609497.5	1.357504	2.076054	3.332999	0.018007	0.198042	-3.09424
NONHSAT158216.1	1.631499	3.017795	3.331853	0.018031	0.198176	-3.09565
lnc-PRR16-3:1	-1.02552	5.150623	-3.33069	0.018055	0.198268	-3.09708
NONHSAT189500.1	1.07133	2.075712	3.329994	0.01807	0.198294	-3.09793
NONHSAT215014.1	1.543263	2.502589	3.329943	0.018071	0.198294	-3.098
NONHSAT150546.1	-1.98085	2.454175	-3.32956	0.018079	0.198319	-3.09847
lnc-PPP1R14C-1:5	1.251009	2.072316	3.328048	0.018111	0.19854	-3.10032
ENST00000601409.1	-1.20574	5.656339	-3.32728	0.018128	0.198613	-3.10127
NONHSAT210310.1	-1.04364	3.468127	-3.32722	0.018129	0.198613	-3.10134
NONHSAT172323.1	-1.47096	2.586939	-3.32717	0.01813	0.198613	-3.1014
MSTRG.19505.10	-1.13566	4.043744	-3.32705	0.018132	0.198613	-3.10155
NONHSAT197607.1	-1.05716	3.819345	-3.32691	0.018135	0.198613	-3.10172
NONHSAT221550.1	-1.31801	1.964826	-3.32646	0.018145	0.198671	-3.10228
NONHSAT153512.1	-1.04019	9.948409	-3.32637	0.018147	0.198671	-3.10239
ENST00000616116.1	-1.26606	3.174095	-3.32556	0.018164	0.198733	-3.10338
NONHSAT167607.1	-1.12894	1.674925	-3.32528	0.01817	0.198752	-3.10373
ENST00000321214.2	1.307828	3.029206	3.324451	0.018188	0.198923	-3.10474
ENST00000597346.1	1.696703	2.432011	3.323801	0.018201	0.198987	-3.10554
MSTRG.42451.1	1.67291	2.26284	3.323262	0.018213	0.19907	-3.10621
ENST00000591110.1	-1.09005	2.193098	-3.32289	0.018221	0.199115	-3.10667
lnc-ZNF37A-15:1	1.627492	8.584102	3.322555	0.018228	0.199145	-3.10707
NONHSAT186713.1	-1.34296	1.947606	-3.32234	0.018233	0.199145	-3.10734
NONHSAT191817.1	-1.43891	2.505249	-3.32219	0.018236	0.199145	-3.10753
lnc-JPH3-3:15	1.552933	2.305493	3.321161	0.018258	0.199176	-3.10879
lnc-PHF20L1-2:1	-1.0891	2.036232	-3.32079	0.018266	0.199176	-3.10925
NONHSAT202881.1	1.451931	1.983658	3.320386	0.018274	0.199176	-3.10974
ENST00000434401.1	1.256608	2.824024	3.320093	0.018281	0.199176	-3.1101
lnc-AKR1C2-6:1	1.388659	2.630826	3.31964	0.01829	0.199184	-3.11066
NONHSAT202891.1	-1.11888	4.115392	-3.31811	0.018323	0.199412	-3.11254
ENST00000653445.1	1.231855	1.74644	3.317952	0.018327	0.199412	-3.11274
NONHSAT166978.1	1.734085	2.753156	3.317387	0.018339	0.199429	-3.11343
MSTRG.1808.16	-1.38488	2.091234	-3.31688	0.01835	0.199453	-3.11405
NONHSAT205763.1	1.34841	1.695625	3.316801	0.018351	0.199453	-3.11415
MSTRG.19554.1	-1.06564	10.47605	-3.31664	0.018355	0.199453	-3.11435
ENST00000669000.1	-1.40824	4.00636	-3.31491	0.018392	0.199608	-3.11648
T114658	1.506774	4.945598	3.314785	0.018395	0.199608	-3.11663
ENST00000429328.2	-1.72267	2.91385	-3.31348	0.018423	0.199702	-3.11823
NONHSAT179060.1	-1.26528	1.98827	-3.31222	0.01845	0.199823	-3.11978

ENST00000652524.1	-1.66519	8.349558	-3.31204	0.018454	0.199823	-3.12001
lnc-TMEM268-4:1	-1.18912	3.047004	-3.31203	0.018454	0.199823	-3.12003
lnc-SMPD2-3:1	-1.42954	2.584624	-3.31142	0.018467	0.199858	-3.12077
lnc-POLE3-7:1	1.034055	3.591848	3.308957	0.018521	0.200139	-3.12381
lnc-SP3-14:1	-2.17099	3.545931	-3.30882	0.018524	0.20014	-3.12397
lnc-NEFL-1:2	1.362945	5.198321	3.308237	0.018536	0.200187	-3.12469
ENST00000428903.1	1.167039	2.43105	3.306963	0.018564	0.20029	-3.12626
ENST00000544195.1	1.1019	2.647225	3.306225	0.01858	0.200349	-3.12717
lnc-POLRMT-6:15	-1.58727	7.169336	-3.30534	0.0186	0.200376	-3.12826
NONHSAT179030.1	1.471141	4.523022	3.305283	0.018601	0.200376	-3.12833
T084652	1.435482	2.845602	3.304958	0.018608	0.200376	-3.12873
lnc-C6orf48-1:1	1.223698	3.165805	3.304911	0.018609	0.200376	-3.12879
ENST00000451362.1	-1.27498	4.258313	-3.30399	0.018629	0.200376	-3.12993
NONHSAT191898.1	-1.08972	8.326665	-3.30385	0.018632	0.200376	-3.1301
NONHSAT199953.1	1.041029	1.728028	3.303711	0.018635	0.200376	-3.13027
ENST00000670964.1	1.140316	1.640037	3.303464	0.01864	0.200376	-3.13057
LINC00239:3	1.179459	1.662014	3.303378	0.018642	0.200376	-3.13068
lnc-IRX5-5:1	1.210306	1.83662	3.30326	0.018645	0.200376	-3.13082
lnc-FOXO4-4:1	1.019079	2.294312	3.30201	0.018672	0.200424	-3.13236
NONHSAT219302.1	1.059491	2.891012	3.301903	0.018675	0.200424	-3.1325
lnc-TRIB1-8:1	-1.07713	4.209178	-3.30139	0.018686	0.20046	-3.13313
MSTRG.9158.2	-1.26592	3.349043	-3.30066	0.018702	0.200491	-3.13403
lnc-SLC25A27-3:1	-1.04328	3.358126	-3.30038	0.018708	0.200506	-3.13438
lnc-IQCH-1:1	-1.10846	1.979175	-3.29988	0.018719	0.20055	-3.13499
MSTRG.48741.1	-1.18274	2.472607	-3.29974	0.018722	0.20055	-3.13517
lnc-G2E3-1:1	-1.64751	2.893245	-3.29941	0.018729	0.200579	-3.13557
lnc-HECA-12:2	1.410288	3.33956	3.299342	0.018731	0.200579	-3.13565
NONHSAT184287.1	-1.05658	1.987576	-3.29863	0.018747	0.200579	-3.13653
NONHSAT170323.1	1.112732	2.830851	3.298474	0.01875	0.200579	-3.13672
NONHSAT176725.1	-1.07496	3.54335	-3.29823	0.018755	0.200606	-3.13702
NONHSAT217056.1	1.609868	2.95442	3.297588	0.01877	0.200651	-3.13782
lnc-OR1K1-1:1	-1.35289	2.423998	-3.29682	0.018787	0.200651	-3.13877
ENST00000651680.1	-1.33026	3.216382	-3.29345	0.018861	0.201139	-3.14292
NONHSAT179102.1	1.230385	4.566259	3.292778	0.018876	0.201197	-3.14375
ENST00000426017.1	-1.05431	4.394014	-3.29276	0.018876	0.201197	-3.14378
lnc-GAPDH-4:2	1.501597	1.805106	3.290906	0.018917	0.201474	-3.14606
NONHSAT215747.1	1.491113	2.617046	3.290869	0.018918	0.201474	-3.1461
ENST00000562031.2	1.400945	3.32244	3.290629	0.018924	0.201487	-3.1464
MSTRG.23106.1	1.018881	4.022326	3.288668	0.018967	0.201592	-3.14882
NONHSAT156703.1	-1.1895	2.164218	-3.28858	0.018969	0.201592	-3.14893
NONHSAT175691.1	1.732129	2.618895	3.287526	0.018993	0.201677	-3.15023
lnc-CDR2L-2:3	-1.1786	2.70111	-3.28677	0.01901	0.201735	-3.15116
lnc-EMP2-2:1	-1.21966	2.347859	-3.28641	0.019018	0.201735	-3.15162
NONHSAT193205.1	-1.11025	1.970448	-3.28612	0.019024	0.201735	-3.15197
NONHSAT192665.1	1.059421	6.141332	3.285891	0.019029	0.201735	-3.15225
MSTRG.56003.1	-1.5022	4.940016	-3.28581	0.019031	0.201735	-3.15235
NONHSAT219637.1	1.457124	1.754708	3.2853	0.019043	0.201786	-3.15298
lnc-MBTPS2-1:1	1.168057	4.818056	3.284273	0.019066	0.201858	-3.15425

NONHSAT198358.1	2.391464	2.959343	3.281841	0.01912	0.202279	-3.15725
NONHSAT179075.1	1.299482	2.191557	3.281519	0.019128	0.202334	-3.15765
lnc-FANCL-6:1	-1.34794	2.375044	-3.2812	0.019135	0.202346	-3.15804
lnc-HRH4-15:1	1.31131	1.784551	3.280514	0.01915	0.202467	-3.15889
MSTRG.30074.1	-1.11314	4.313352	-3.28028	0.019155	0.202502	-3.15918
lnc-SLC15A4-16:1	-1.17625	2.943066	-3.28011	0.019159	0.202522	-3.15939
lnc-KLHL36-2:3	1.292104	3.486698	3.279862	0.019165	0.202559	-3.1597
NR_110816	-1.09758	4.331874	-3.27911	0.019182	0.202654	-3.16063
T070327	1.052509	2.616273	3.278916	0.019186	0.202679	-3.16087
NR_120571	1.208538	2.515216	3.278458	0.019197	0.202746	-3.16143
NONHSAT221602.1	1.408436	1.753496	3.278206	0.019202	0.202784	-3.16175
NONHSAT203446.1	-1.04244	1.928574	-3.27756	0.019217	0.202853	-3.16254
ENST00000649345.1	1.817816	3.083037	3.276047	0.019251	0.203037	-3.16442
NONHSAT202455.1	1.16598	2.099418	3.275302	0.019268	0.203075	-3.16534
T357649	-1.1516	4.146576	-3.27507	0.019273	0.203086	-3.16562
MSTRG.57243.4	1.058503	2.825392	3.275036	0.019274	0.203086	-3.16566
NONHSAT218131.1	1.851353	2.753013	3.273768	0.019303	0.203124	-3.16723
lnc-CALCRL-3:1	1.517493	2.62556	3.273482	0.019309	0.203171	-3.16759
MSTRG.46888.2	1.327684	2.980361	3.27308	0.019319	0.203225	-3.16808
lnc-OR56A3-1:1	1.652806	3.054981	3.271864	0.019346	0.203334	-3.16959
NONHSAT169761.1	1.130252	3.89391	3.271713	0.01935	0.203335	-3.16977
T002036	1.169389	2.131865	3.270266	0.019383	0.2035	-3.17157
lnc-MAP3K8-5:2	1.086919	1.626443	3.270232	0.019384	0.2035	-3.17161
NONHSAT175557.1	-1.32149	3.699518	-3.27007	0.019387	0.2035	-3.17181
ENST00000671143.1	-1.59748	2.577604	-3.26985	0.019392	0.2035	-3.17208
T363691	1.146993	2.039115	3.269656	0.019397	0.2035	-3.17232
NONHSAT215803.1	-1.27098	2.159871	-3.26954	0.019399	0.2035	-3.17246
T086049	1.405127	3.161878	3.268959	0.019413	0.2035	-3.17318
NONHSAT177484.1	-1.13927	1.799295	-3.26892	0.019413	0.2035	-3.17323
NONHSAT155026.1	1.293052	6.461722	3.26888	0.019414	0.2035	-3.17328
ENST00000505018.1	1.709372	2.256014	3.268567	0.019422	0.2035	-3.17367
T379777	2.015834	3.185562	3.268249	0.019429	0.2035	-3.17406
NONHSAT222296.1	-1.35437	2.742954	-3.26759	0.019444	0.203513	-3.17488
MSTRG.60712.1	1.509937	2.629441	3.26715	0.019454	0.203513	-3.17542
NONHSAT150120.1	-1.31696	6.475971	-3.26705	0.019457	0.203513	-3.17555
NONHSAT171906.1	1.228936	2.544989	3.266186	0.019476	0.203635	-3.17661
lnc-CYB5R4-2:1	1.015038	2.910283	3.266033	0.01948	0.203646	-3.1768
lnc-ODF1-2:1	-1.49644	2.090062	-3.26533	0.019496	0.203773	-3.17768
T271557	1.100437	2.658156	3.265176	0.019499	0.203788	-3.17787
NONHSAT183921.1	-1.29795	1.741045	-3.26382	0.019531	0.204008	-3.17954
NONHSAT190400.1	1.350622	2.646376	3.262954	0.019551	0.204091	-3.18062
NONHSAT176550.1	1.048511	5.229739	3.261836	0.019576	0.204235	-3.182
lnc-SMNDC1-9:1	1.004233	1.551725	3.26117	0.019592	0.204286	-3.18283
NONHSAT160965.1	1.234013	2.063761	3.260905	0.019598	0.204286	-3.18315
NONHSAT153789.1	-1.92955	2.807905	-3.26054	0.019606	0.204292	-3.1836
NONHSAT169912.1	1.167433	3.062697	3.260033	0.019618	0.204338	-3.18423
lnc-OR1S1-1:1	1.045225	4.337224	3.259659	0.019627	0.204352	-3.1847
NONHSAT204829.1	1.098621	1.918502	3.257986	0.019666	0.204501	-3.18677

lnc-ALX4-17:1	1.192028	1.787259	3.257897	0.019668	0.204501	-3.18688
lnc-POC1B-GALNT4-2:1	1.323247	2.196931	3.256344	0.019704	0.20463	-3.18881
ENST00000424177.1	1.435305	2.079266	3.25613	0.019709	0.20463	-3.18907
NONHSAT208159.1	1.815036	5.246436	3.254247	0.019753	0.204716	-3.1914
ENST00000506392.1	1.123066	1.826515	3.253935	0.01976	0.204728	-3.19179
NONHSAT153870.1	-1.2477	2.184624	-3.25375	0.019764	0.204752	-3.19202
lnc-C1D-15:1	-1.09778	3.999701	-3.25324	0.019776	0.204771	-3.19266
NONHSAT181849.1	1.799267	2.403801	3.252751	0.019788	0.204785	-3.19326
NONHSAT200023.1	-1.4891	2.833802	-3.25097	0.019829	0.205026	-3.19547
NONHSAT156352.1	1.175834	1.904753	3.250326	0.019844	0.205118	-3.19627
lnc-MRPS33-4:1	-1.06781	3.092683	-3.24982	0.019856	0.205169	-3.19689
lnc-MBP-6:1	1.30297	1.932838	3.249772	0.019857	0.205169	-3.19695
NONHSAT198103.1	1.361286	2.183095	3.249441	0.019865	0.205228	-3.19736
ENST00000655873.1	1.573994	2.394287	3.248516	0.019887	0.205264	-3.19851
NONHSAT205333.1	1.289337	3.239397	3.248511	0.019887	0.205264	-3.19852
ENST00000591038.1	1.226706	2.916103	3.247516	0.01991	0.20543	-3.19975
NONHSAT162932.1	-1.05252	2.847531	-3.24705	0.019921	0.205472	-3.20033
MSTRG.22299.1	1.00164	6.213324	3.245157	0.019966	0.205743	-3.20268
lnc-TFB2M-4:1	-1.37074	5.350806	-3.24334	0.020009	0.205997	-3.20494
lnc-HS3ST3B1-12:1	-2.23895	2.490624	-3.2427	0.020024	0.206061	-3.20572
MSTRG.45384.1	1.727456	2.694013	3.242694	0.020024	0.206061	-3.20574
lnc-C6orf201-9:1	1.201682	5.993648	3.241237	0.020059	0.206251	-3.20755
NONHSAT177182.1	-1.28685	9.073172	-3.24016	0.020084	0.206393	-3.20888
lnc-LYPD6-12:1	1.056825	4.378674	3.239449	0.020101	0.206428	-3.20976
T043258	1.350338	3.256486	3.238797	0.020117	0.206465	-3.21058
lnc-SRSF6-6:1	-1.05611	4.241337	-3.23858	0.020122	0.206465	-3.21085
ENST00000664643.1	1.140615	2.992767	3.238157	0.020132	0.206484	-3.21137
lnc-FBN1-1:1	-1.03502	2.713554	-3.23717	0.020156	0.20664	-3.21259
MSTRG.12493.1	-1.32718	2.441719	-3.23625	0.020178	0.206755	-3.21373
MSTRG.30757.1	-1.23106	4.3682	-3.23556	0.020194	0.206805	-3.2146
T302404	-1.09773	2.116712	-3.23542	0.020197	0.206809	-3.21477
lnc-SUMF1-12:4	1.843765	2.614892	3.234699	0.020215	0.206868	-3.21567
ENST00000662988.1	1.063831	1.605997	3.232831	0.020259	0.20699	-3.21799
NONHSAT203444.1	1.668871	2.137151	3.232388	0.02027	0.206996	-3.21854
NONHSAT216502.1	-1.2003	3.422126	-3.23232	0.020272	0.206996	-3.21862
NONHSAT169915.1	1.314953	2.078051	3.23154	0.020291	0.207076	-3.21959
lnc-MYC-18:1	-1.16562	11.02894	-3.23089	0.020306	0.207214	-3.2204
NONHSAT175098.1	1.43103	2.226242	3.23031	0.02032	0.207262	-3.22112
NONHSAT214984.1	1.064836	1.67847	3.229701	0.020335	0.207269	-3.22188
MSTRG.48747.1	-1.54647	2.868762	-3.22729	0.020393	0.207499	-3.22487
T120764	1.115481	3.326232	3.226841	0.020404	0.207499	-3.22543
NONHSAT167179.1	1.637975	4.653405	3.22678	0.020405	0.207499	-3.22551
lnc-TMPRSS15-25:2	1.249683	3.277817	3.226577	0.02041	0.207499	-3.22576
ENST00000638002.1	-1.23346	2.984949	-3.22526	0.020442	0.207677	-3.2274
lnc-ANKRD60-1:3	2.000938	2.565191	3.225033	0.020448	0.20768	-3.22768
NONHSAT161485.1	1.15614	3.404839	3.223888	0.020475	0.207848	-3.22911
NONHSAT224516.1	-1.0541	3.19112	-3.22231	0.020514	0.208102	-3.23107

lnc-CAMSAP1-1:1	1.582384	4.478784	3.221396	0.020536	0.208102	-3.23221
MSTRG.22627.1	1.191443	1.922111	3.221378	0.020536	0.208102	-3.23223
lnc-HLA-DQA1-10:1	1.243424	2.15801	3.221259	0.020539	0.208102	-3.23238
NONHSAT169582.1	1.523333	1.821552	3.220311	0.020562	0.208102	-3.23356
lnc-EIF5-1:2	1.588772	2.557817	3.220297	0.020563	0.208102	-3.23358
lnc-ZFYVE28-2:1	-1.04595	3.534908	-3.22021	0.020565	0.208102	-3.23369
NONHSAT177413.1	-1.22966	3.40046	-3.21996	0.020571	0.208102	-3.234
ENST00000421041.1	1.099344	4.06774	3.219863	0.020573	0.208102	-3.23412
MSTRG.37535.1	-1.01657	4.328585	-3.21972	0.020577	0.208102	-3.23429
NONHSAT212456.1	-1.10552	2.349216	-3.21927	0.020588	0.208102	-3.23485
NONHSAT178822.1	1.290825	2.115326	3.218425	0.020609	0.208194	-3.23591
NONHSAT179237.1	-1.25479	2.384073	-3.21802	0.020618	0.208194	-3.23641
NONHSAT158557.1	1.098135	3.598438	3.217915	0.020621	0.208194	-3.23654
lnc-EFCAB1-5:3	1.038675	1.60436	3.215531	0.020679	0.208472	-3.23951
ENST00000524011.1	1.010446	3.957638	3.21536	0.020684	0.208472	-3.23972
ENST00000653211.1	-1.28159	2.42422	-3.21441	0.020707	0.208588	-3.24091
lnc-SLC45A4-5:1	-1.51318	10.16708	-3.2142	0.020712	0.208588	-3.24117
MSTRG.57559.6	-1.38242	7.956592	-3.21413	0.020714	0.208588	-3.24125
lnc-AKR1E2-10:1	-1.42644	3.040431	-3.21355	0.020728	0.208649	-3.24198
NONHSAT201158.1	1.362281	3.701562	3.213039	0.020741	0.208693	-3.24262
ENST00000657048.1	-1.05186	3.448634	-3.21248	0.020754	0.208726	-3.24331
lnc-F13B-2:1	1.168775	2.962665	3.212162	0.020762	0.208753	-3.24371
NONHSAT194365.1	1.132962	1.667756	3.211964	0.020767	0.208753	-3.24395
NONHSAT214981.1	1.392361	1.850281	3.211957	0.020767	0.208753	-3.24396
lnc-LPP-5:1	1.281544	1.726777	3.210399	0.020806	0.208806	-3.2459
NONHSAT197714.1	1.518639	3.566945	3.210228	0.02081	0.208809	-3.24612
ENST00000510176.1	-1.44487	2.40484	-3.2101	0.020813	0.208809	-3.24628
MSTRG.51281.1	-1.03223	1.858809	-3.21005	0.020814	0.208809	-3.24634
ENST00000648410.1	-1.29816	2.292148	-3.20975	0.020822	0.208822	-3.24671
NONHSAT161481.1	1.094651	5.165569	3.209102	0.020838	0.208913	-3.24752
ENST00000428920.2	1.391058	5.178665	3.20905	0.020839	0.208913	-3.24759
lnc-VSTM5-3:1	1.204784	1.687798	3.2087	0.020848	0.20894	-3.24802
ENST00000429980.6	-1.44835	3.064513	-3.208	0.020865	0.208958	-3.24889
MSTRG.39548.1	-1.00654	2.823316	-3.2077	0.020873	0.208958	-3.24926
ENST00000440516.1	-1.9845	2.073655	-3.20734	0.020882	0.208958	-3.24972
NONHSAT196979.1	1.314733	2.001147	3.207112	0.020887	0.208958	-3.25
LINC00879:4	-1.0262	1.985754	-3.20671	0.020897	0.208973	-3.2505
NONHSAT190409.1	1.588059	2.986712	3.206492	0.020903	0.208973	-3.25077
T197635	-1.03219	1.992923	-3.2062	0.02091	0.208973	-3.25114
lnc-DDIT4L-4:1	1.049085	2.04088	3.205807	0.02092	0.209002	-3.25163
NONHSAT163867.1	1.109263	4.576065	3.205538	0.020926	0.209002	-3.25196
MSTRG.52731.1	-1.17188	2.679265	-3.2053	0.020932	0.209015	-3.25226
ENST00000554614.2	1.074716	4.674396	3.204541	0.020951	0.20908	-3.25321
lnc-PRL-9:1	1.866893	2.674804	3.204456	0.020953	0.20908	-3.25331
NONHSAT190142.1	1.348239	3.94163	3.203836	0.020969	0.209174	-3.25409
ENST00000653358.1	1.386161	2.550514	3.202921	0.020992	0.209254	-3.25523
lnc-ZNF77-3:1	-1.09452	4.864544	-3.2023	0.021007	0.209325	-3.256
lnc-IL2RG-2:1	1.345409	3.033653	3.201706	0.021022	0.209412	-3.25674

MSTRG.608.1	-1.55514	6.699756	-3.2	0.021065	0.209616	-3.25887
MSTRG.67272.1	1.663898	2.402912	3.199769	0.021071	0.209616	-3.25916
lnc-CD38-12:3	1.077307	5.856068	3.199683	0.021073	0.209616	-3.25927
NONHSAT176413.1	-1.24819	3.444563	-3.19939	0.02108	0.209634	-3.25963
NONHSAT167354.1	1.157024	1.933929	3.199323	0.021082	0.209634	-3.25972
NONHSAT150645.1	-1.24568	3.163586	-3.1989	0.021092	0.20972	-3.26025
lnc-TCF24-4:1	-1.17147	2.342996	-3.19805	0.021114	0.209868	-3.2613
NONHSAT197729.1	1.420809	2.508872	3.197807	0.02112	0.209888	-3.26161
NONHSAT203890.1	1.011457	5.397593	3.196884	0.021143	0.209994	-3.26276
NONHSAT157986.1	-1.63804	3.651581	-3.19666	0.021149	0.21001	-3.26304
ENST00000456337.1	-1.15229	2.213597	-3.19587	0.021168	0.210122	-3.26402
lnc-TM2D3-2:3	-1.033	3.152977	-3.19568	0.021173	0.210122	-3.26426
MSTRG.63532.1	1.412402	2.278737	3.195632	0.021174	0.210122	-3.26432
MSTRG.40451.1	-1.25496	2.906266	-3.19472	0.021197	0.210246	-3.26546
MSTRG.55816.1	1.462511	2.878926	3.1924	0.021256	0.210537	-3.26836
lnc-VPS26C-14:1	1.455988	2.339118	3.19143	0.021281	0.2106	-3.26957
NONHSAT201975.1	1.128227	2.952437	3.191384	0.021282	0.2106	-3.26963
MSTRG.25518.1	-1.77174	1.99131	-3.18899	0.021343	0.21096	-3.27262
MSTRG.68174.1	1.44593	1.891923	3.187841	0.021372	0.211147	-3.27405
lnc-ARAP2-11:1	1.061886	3.84041	3.187561	0.021379	0.211167	-3.2744
lnc-LSG1-2:1	-1.31618	2.524479	-3.1868	0.021398	0.2112	-3.27535
lnc-POTEB3-2:1	-1.36689	2.366748	-3.18548	0.021432	0.21133	-3.277
MSTRG.47110.2	-1.31497	4.260578	-3.18545	0.021433	0.21133	-3.27704
lnc-NTF3-4:1	1.637291	2.8951	3.185124	0.021441	0.211354	-3.27745
NONHSAT179936.1	1.151129	1.692532	3.184682	0.021453	0.211424	-3.278
lnc-BCAS1-6:1	-1.36959	5.729601	-3.18313	0.021492	0.211609	-3.27994
NONHSAT186288.1	-1.02899	2.350068	-3.18227	0.021514	0.211704	-3.28102
ENST00000379677.2	1.335115	3.252197	3.182211	0.021516	0.211704	-3.28109
MSTRG.15583.2	-1.28642	2.430114	-3.18189	0.021524	0.211732	-3.28149
MSTRG.61020.3	-1.1808	2.416934	-3.18151	0.021534	0.211753	-3.28196
NONHSAT149101.1	-1.65879	2.986218	-3.18039	0.021563	0.211908	-3.28337
NONHSAT218083.1	1.600334	2.700469	3.179496	0.021586	0.212008	-3.28448
lnc-TNFSF15-3:1	1.123332	2.900481	3.178613	0.021608	0.212123	-3.28559
lnc-FGF16-1:1	-1.5243	4.151101	-3.17849	0.021611	0.212123	-3.28574
lnc-NR3C2-6:1	-1.77778	4.384344	-3.17848	0.021612	0.212123	-3.28576
NONHSAT162938.1	1.269498	2.459346	3.177268	0.021643	0.212224	-3.28727
lnc-ITGA6-3:1	2.102599	5.129147	3.176572	0.021661	0.212312	-3.28814
ENST00000624882.1	1.559958	2.629383	3.175584	0.021687	0.212381	-3.28938
NONHSAT209661.1	1.843552	2.738594	3.175258	0.021695	0.212381	-3.28979
lnc-CPS1-5:2	1.622664	3.014677	3.175245	0.021695	0.212381	-3.2898
NONHSAT151052.1	1.450249	2.477903	3.174449	0.021716	0.212443	-3.2908
lnc-ESM1-4:1	1.577606	4.950301	3.173852	0.021731	0.212532	-3.29154
lnc-ANXA10-4:1	1.360596	4.26673	3.173201	0.021748	0.212569	-3.29236
lnc-FAR2-1:1	1.264545	3.323464	3.172675	0.021762	0.212569	-3.29302
NONHSAT166826.1	1.364696	3.278386	3.172652	0.021763	0.212569	-3.29305
MSTRG.5963.1	1.398849	2.480229	3.172021	0.021779	0.212667	-3.29384
lnc-CLIC6-5:1	1.043021	1.714734	3.17164	0.021789	0.212723	-3.29431
lnc-BIN3-7:1	1.238012	2.726618	3.167267	0.021903	0.213411	-3.29979

ENST00000456036.2	-1.1888	3.93756	-3.16722	0.021904	0.213411	-3.29985
lnc-CENPN-1:1	1.240091	2.77728	3.167168	0.021906	0.213411	-3.29991
ENST00000504409.3	1.416873	2.773753	3.166207	0.021931	0.213438	-3.30112
ENST00000450669.2	1.246922	1.675695	3.165671	0.021945	0.213501	-3.30179
MSTRG.46264.1	1.288698	2.112975	3.164058	0.021987	0.21368	-3.30381
LINC01684:29	-1.03264	2.262749	-3.16387	0.021992	0.21368	-3.30405
ENST00000542577.2	1.424347	2.60303	3.163719	0.021996	0.21368	-3.30423
NONHSAT167643.1	1.070527	1.977502	3.16368	0.021997	0.21368	-3.30428
NONHSAT199449.1	1.399113	2.591753	3.163626	0.021999	0.21368	-3.30435
ENST00000645557.1	1.735718	3.011321	3.163463	0.022003	0.213687	-3.30455
LINC00402:2	1.47239	1.788549	3.161466	0.022055	0.214002	-3.30706
NONHSAT153835.1	-1.18145	4.244898	-3.16111	0.022065	0.214048	-3.3075
NONHSAT218574.1	1.096459	2.777353	3.160896	0.02207	0.214051	-3.30777
lnc-ZC3H12B-8:1	-1.43024	2.607711	-3.1603	0.022086	0.214051	-3.30852
NONHSAT182095.1	1.543542	2.353813	3.159962	0.022095	0.214095	-3.30894
lnc-SCARB1-6:1	-1.19895	8.185319	-3.15972	0.022102	0.214116	-3.30924
NONHSAT154348.1	1.037156	3.816979	3.158738	0.022128	0.214289	-3.31047
NONHSAT197088.1	-1.14164	2.983215	-3.15759	0.022158	0.214477	-3.31192
NONHSAT214447.1	1.317298	2.603437	3.156509	0.022187	0.214615	-3.31327
lnc-KMT5A-20:1	1.162125	6.555596	3.155569	0.022212	0.214732	-3.31445
lnc-PRR26-5:1	1.709007	2.986456	3.155395	0.022216	0.214732	-3.31467
NONHSAT190512.1	1.372749	3.642226	3.155193	0.022222	0.214763	-3.31492
NONHSAT167016.1	-1.50908	6.386891	-3.1539	0.022256	0.214856	-3.31654
ENST00000434730.1	1.432721	1.875012	3.153303	0.022272	0.214858	-3.31729
NONHSAT158004.1	-1.84261	3.143412	-3.1527	0.022288	0.214889	-3.31804
ENST00000661809.1	1.129579	3.564066	3.152406	0.022296	0.214895	-3.31842
ENST00000503695.4	-1.61319	4.093979	-3.15236	0.022297	0.214895	-3.31847
NONHSAT197999.1	-1.46942	3.470553	-3.15137	0.022323	0.214971	-3.31971
ENST00000663658.1	1.323349	2.420355	3.151347	0.022324	0.214971	-3.31975
NONHSAT188020.1	-1.52912	3.743384	-3.15108	0.022331	0.215007	-3.32008
NONHSAT180828.1	1.136891	5.301602	3.151048	0.022332	0.215007	-3.32012
NONHSAT200629.1	-1.30548	8.061352	-3.15016	0.022356	0.215152	-3.32123
lnc-LIG4-2:1	-1.0023	5.916636	-3.14927	0.02238	0.215265	-3.32235
NONHSAT187334.1	1.495686	2.009412	3.147241	0.022434	0.215434	-3.3249
MSTRG.36572.18	1.673355	3.038907	3.147019	0.02244	0.21545	-3.32518
lnc-ZNF267-5:1	1.215887	3.150776	3.146829	0.022445	0.215461	-3.32541
NONHSAT162129.1	1.482039	2.792398	3.146801	0.022446	0.215461	-3.32545
lnc-OPHN1-3:1	-1.13854	3.437094	-3.1467	0.022449	0.215461	-3.32558
lnc-UTP18-2:2	1.801267	1.93232	3.145778	0.022473	0.215484	-3.32673
ENST00000588548.1	-1.00819	5.649489	-3.14551	0.022481	0.215512	-3.32707
lnc-CXorf38-6:1	1.326982	2.578384	3.145099	0.022492	0.215518	-3.32759
NONHSAT224192.1	1.134133	4.289238	3.144713	0.022502	0.215554	-3.32807
MSTRG.20203.1	1.541719	3.26869	3.142867	0.022552	0.215748	-3.33039
MSTRG.840.1	1.919775	2.406244	3.14276	0.022555	0.215748	-3.33052
NONHSAT195156.1	1.097994	1.651241	3.142581	0.02256	0.215748	-3.33075
lnc-TSPY10-8:1	1.920304	4.008568	3.141958	0.022577	0.215795	-3.33153
ENST00000620563.1	-1.19238	5.522617	-3.14094	0.022604	0.215894	-3.3328
NR_126010	1.609171	2.41213	3.140564	0.022614	0.215951	-3.33328

NONHSAT158138.1	1.118819	1.620058	3.140337	0.022621	0.215989	-3.33357
ENST00000562900.1	1.381328	2.751031	3.138921	0.022659	0.216111	-3.33535
ENST00000671197.1	1.172867	2.630536	3.138592	0.022668	0.216135	-3.33576
MSTRG.29873.1	1.481894	3.470423	3.138432	0.022672	0.216135	-3.33596
lnc-UBXN6-2:4	1.45064	3.419176	3.138063	0.022682	0.216135	-3.33642
ENST00000540906.1	1.055279	3.69601	3.137925	0.022686	0.216144	-3.3366
lnc-GDPD4-3:3	1.672586	2.335476	3.135595	0.02275	0.216572	-3.33953
ENST00000509711.2	-1.49709	2.608599	-3.13528	0.022758	0.216626	-3.33992
NR_126564	1.708904	2.771904	3.134665	0.022775	0.216705	-3.34069
NONHSAT160448.1	-1.00874	2.818388	-3.1345	0.022779	0.216726	-3.3409
ENST00000661222.1	1.769237	3.13677	3.133554	0.022805	0.216891	-3.34209
NONHSAT170011.1	-1.1388	2.094086	-3.13019	0.022897	0.217317	-3.34632
lnc-HNRNPA1P48-17:1	1.198743	2.045181	3.130192	0.022898	0.217317	-3.34632
MSTRG.65994.1	1.180795	4.501825	3.12978	0.022909	0.217337	-3.34684
lnc-SYT5-1:2	1.023496	2.168323	3.129392	0.02292	0.217402	-3.34733
T280150	-1.48328	1.85344	-3.1284	0.022947	0.217498	-3.34857
MSTRG.26213.1	1.541568	2.531752	3.127007	0.022985	0.217707	-3.35033
NONHSAT178788.1	-1.19116	2.479284	-3.12678	0.022991	0.217716	-3.35061
lnc-PLCD1-5:1	-1.01573	7.368604	-3.12655	0.022998	0.217716	-3.3509
NONHSAT202399.1	-1.0254	1.829727	-3.1256	0.023024	0.217761	-3.3521
lnc-SLITRK5-3:2	1.044487	1.723908	3.124625	0.023051	0.217893	-3.35332
NONHSAT209770.1	1.796365	2.325164	3.124456	0.023056	0.217917	-3.35353
NONHSAT177868.1	1.049664	3.785112	3.123471	0.023083	0.218073	-3.35477
MSTRG.15651.1	-1.25277	2.310342	-3.12276	0.023103	0.21817	-3.35568
NONHSAT224511.1	1.187088	4.363922	3.122712	0.023104	0.21817	-3.35573
MSTRG.12746.1	-1.30735	2.223014	-3.12237	0.023114	0.218222	-3.35616
NONHSAT153830.1	2.120868	3.72732	3.122351	0.023114	0.218222	-3.35618
NONHSAT207830.1	-1.28148	2.775229	-3.12152	0.023137	0.218318	-3.35723
lnc-NAIF1-1:4	-1.11044	1.953946	-3.12139	0.023141	0.218332	-3.3574
NONHSAT167961.1	-1.41562	1.972724	-3.12103	0.023151	0.218332	-3.35785
lnc-RHBDL2-2:1	1.728764	2.857411	3.120711	0.02316	0.218332	-3.35825
NONHSAT154672.1	-1.21468	6.530623	-3.1207	0.02316	0.218332	-3.35826
MSTRG.67610.1	1.195124	1.669598	3.12069	0.02316	0.218332	-3.35828
MSTRG.72589.1	1.866363	2.53022	3.120013	0.023179	0.218449	-3.35913
T258398	-2.02602	3.71366	-3.11891	0.02321	0.218576	-3.36052
NONHSAT205824.1	1.41348	4.650181	3.118614	0.023218	0.218591	-3.36089
LINC01714:22	1.533011	2.857196	3.118135	0.023232	0.218628	-3.36149
lnc-LBH-4:1	1.264623	1.905296	3.118096	0.023233	0.218628	-3.36154
lnc-LMOD3-4:1	-1.03896	3.599666	-3.11809	0.023233	0.218628	-3.36155
NONHSAT170016.1	1.717864	2.306547	3.117519	0.023249	0.218655	-3.36227
lnc-OFD1-3:1	1.298824	1.705476	3.116991	0.023264	0.218713	-3.36293
lnc-HTR2C-5:1	-1.23002	2.825812	-3.11634	0.023282	0.218822	-3.36375
ENST00000568430.1	-1.01104	4.809228	-3.11603	0.023291	0.218865	-3.36415
NR_125983	-1.25111	2.017237	-3.11582	0.023296	0.218878	-3.36441
ENST00000450681.1	1.637636	2.529003	3.115196	0.023314	0.21891	-3.36519
NONHSAT186781.1	1.1379	4.37352	3.11482	0.023324	0.218932	-3.36567
NONHSAT218677.1	-1.08053	3.697918	-3.11409	0.023345	0.218973	-3.36658
NONHSAT209857.1	-1.30347	3.67931	-3.11258	0.023387	0.219106	-3.36849

T153361	1.327718	3.345921	3.111997	0.023404	0.219134	-3.36923
NONHSAT222813.1	2.091216	3.064131	3.111655	0.023413	0.219142	-3.36966
lnc-KCNJ12-7:1	-1.12227	2.403653	-3.11141	0.02342	0.219142	-3.36996
MSTRG.10987.2	-1.00608	1.895376	-3.1111	0.023429	0.219185	-3.37036
NONHSAT167367.1	-1.44682	3.420337	-3.11099	0.023432	0.21919	-3.3705
lnc-PRR32-9:1	-1.48479	2.385764	-3.11064	0.023442	0.219208	-3.37094
ENST00000442936.1	-1.05092	3.462946	-3.11034	0.023451	0.219208	-3.37132
ENST00000424678.1	1.226621	1.701454	3.109762	0.023467	0.219281	-3.37204
ENST00000635852.1	1.403969	3.022932	3.109011	0.023488	0.219414	-3.37299
lnc-PROKR2-2:3	-1.18744	2.916837	-3.1083	0.023508	0.219451	-3.37389
NR_033423	-1.31965	3.865559	-3.10773	0.023524	0.219503	-3.3746
lnc-SP4-4:1	1.109783	2.498153	3.107317	0.023536	0.21952	-3.37512
NONHSAT171819.1	-1.52883	2.209	-3.10631	0.023565	0.219598	-3.3764
lnc-SLC39A10-12:1	1.325759	1.713799	3.106251	0.023566	0.219598	-3.37647
ENST00000453953.6	-1.13372	10.82272	-3.10552	0.023587	0.219649	-3.37739
NONHSAT210748.1	-1.07245	3.319033	-3.1055	0.023588	0.219649	-3.37742
lnc-MOCS2-5:1	1.135487	1.912576	3.10495	0.023603	0.21974	-3.37811
MSTRG.57193.38	-1.69633	3.101688	-3.10492	0.023604	0.21974	-3.37814
T314610	1.643245	3.115424	3.104685	0.023611	0.219769	-3.37844
NONHSAT195487.1	-1.09119	2.541237	-3.10466	0.023611	0.219769	-3.37847
T346986	1.736484	3.957241	3.10389	0.023633	0.219839	-3.37945
lnc-MAP3K7-4:1	-1.17062	2.026706	-3.10368	0.023639	0.219845	-3.37971
NONHSAT223944.1	1.69463	3.020646	3.102453	0.023674	0.219977	-3.38126
NR_122112	1.021531	3.355021	3.102244	0.02368	0.219977	-3.38152
lnc-CCDC7-11:1	1.516329	2.546016	3.102114	0.023684	0.219977	-3.38169
NONHSAT166707.1	-1.21804	2.707994	-3.10078	0.023722	0.220257	-3.38337
ENST00000615168.4	-1.22194	2.76441	-3.10075	0.023723	0.220257	-3.3834
lnc-P3H2-5:1	1.02557	1.720198	3.099917	0.023747	0.220401	-3.38446
NONHSAT180689.1	1.077631	1.951022	3.099792	0.02375	0.220401	-3.38462
ENST00000438619.1	1.806395	2.387339	3.099737	0.023752	0.220401	-3.38469
MSTRG.59350.1	-1.26353	1.916077	-3.09966	0.023754	0.220401	-3.38479
ENST00000609657.1	-1.23363	3.406983	-3.09903	0.023772	0.220473	-3.38558
NONHSAT183290.1	1.199292	5.244644	3.098378	0.023791	0.220564	-3.3864
NONHSAT167788.1	-1.17525	1.832124	-3.09655	0.023843	0.220866	-3.3887
lnc-CTXND2-7:2	-1.57885	3.298004	-3.09469	0.023897	0.221039	-3.39106
lnc-DTWD1-4:1	-1.40568	2.240845	-3.09454	0.023901	0.221039	-3.39125
ENST00000655341.1	1.528193	1.827585	3.094087	0.023914	0.221098	-3.39182
MSTRG.29184.1	-1.15027	2.515377	-3.0938	0.023923	0.221154	-3.39218
lnc-CCDC167-2:2	1.803187	4.548831	3.09365	0.023927	0.221174	-3.39237
NONHSAT187667.1	-1.03108	2.4415	-3.09319	0.02394	0.221226	-3.39295
ENST00000493033.1	-1.0588	5.754823	-3.09288	0.023949	0.221257	-3.39334
lnc-NCOR2-6:1	1.111264	2.783563	3.091793	0.02398	0.221365	-3.39472
MSTRG.71557.1	-1.01589	7.232705	-3.08966	0.024042	0.221543	-3.39741
MSTRG.48020.1	1.251892	3.724297	3.089514	0.024047	0.221549	-3.39759
lnc-F8-2:1	-1.13423	2.860259	-3.089	0.024061	0.221656	-3.39824
ENST00000523935.1	1.082516	2.571631	3.087591	0.024102	0.221881	-3.40002
NONHSAT215205.1	1.750163	2.659792	3.087228	0.024113	0.221918	-3.40048
NONHSAT195407.1	2.084672	3.402459	3.086876	0.024123	0.221955	-3.40093

NONHSAT159764.1	1.105795	2.316403	3.085771	0.024155	0.22214	-3.40232
MSTRG.2812.2	-1.01977	12.1697	-3.08572	0.024157	0.22214	-3.40239
T160319	1.024956	2.133688	3.085333	0.024168	0.222154	-3.40288
lnc-PPIL1-1:1	-1.34772	2.65294	-3.08524	0.024171	0.222154	-3.40299
NONHSAT170153.1	-1.29145	2.751654	-3.08461	0.024189	0.222235	-3.4038
ENST00000636059.1	1.421654	2.260705	3.083955	0.024208	0.222348	-3.40462
NONHSAT177097.1	-1.07222	2.901956	-3.08353	0.024221	0.222358	-3.40516
ENST00000542197.1	1.726674	2.927562	3.083334	0.024227	0.222375	-3.4054
NONHSAT185574.1	1.032008	3.234996	3.081482	0.024281	0.22261	-3.40775
lnc-C3orf80-1:1	1.040389	1.599003	3.080671	0.024305	0.222734	-3.40877
lnc-DIO1-1:1	-1.34081	4.154424	-3.07855	0.024367	0.22301	-3.41145
MSTRG.2195.1	1.689141	2.851705	3.078403	0.024371	0.22301	-3.41164
MSTRG.16160.1	1.150535	2.208349	3.078352	0.024373	0.22301	-3.4117
ENST00000536835.2	1.708313	3.081153	3.078048	0.024382	0.223036	-3.41209
lnc-TMEM200B-2:1	1.231168	1.69088	3.076013	0.024442	0.223334	-3.41466
NONHSAT187478.1	-1.22431	2.475606	-3.07554	0.024456	0.223377	-3.41526
lnc-ZBTB32-4:1	1.236103	2.248062	3.074838	0.024477	0.223383	-3.41615
NONHSAT182897.1	1.261235	2.159162	3.074635	0.024483	0.223383	-3.41641
NONHSAT220513.1	-1.246	1.796764	-3.07425	0.024494	0.223426	-3.41689
MSTRG.49527.9	-1.12815	3.210764	-3.07358	0.024514	0.223508	-3.41775
lnc-INHBA-1:2	1.430204	2.433251	3.073574	0.024514	0.223508	-3.41775
LINC01792:5	1.905309	3.447781	3.072316	0.024551	0.223706	-3.41934
lnc-ALKBH2-3:1	-1.06523	2.292264	-3.07154	0.024574	0.223814	-3.42032
NONHSAT178955.1	1.260306	2.160097	3.070941	0.024592	0.223916	-3.42108
NONHSAT219358.1	1.023788	5.463872	3.070181	0.024615	0.224001	-3.42204
lnc-MYO3A-2:1	1.336022	2.214955	3.069916	0.024623	0.224033	-3.42238
NONHSAT161596.1	1.317969	1.742376	3.066985	0.02471	0.224277	-3.42609
NONHSAT175577.1	1.847184	2.047324	3.066562	0.024723	0.22428	-3.42663
lnc-MS4A4A-1:1	1.748168	2.614423	3.065792	0.024746	0.224371	-3.4276
ENST00000662897.1	-1.08908	2.313275	-3.06543	0.024757	0.224371	-3.42806
ENST00000621331.1	1.562656	4.217254	3.065425	0.024757	0.224371	-3.42807
lnc-DHRS2-5:1	1.116114	2.099328	3.064046	0.024798	0.22441	-3.42981
NONHSAT198022.1	1.243103	2.159446	3.06358	0.024812	0.224419	-3.4304
NONHSAT172098.1	1.147342	3.291993	3.063478	0.024815	0.224419	-3.43053
NONHSAT217880.1	1.022571	1.613753	3.063436	0.024817	0.224419	-3.43059
lnc-ZNF25-2:1	1.988395	2.849062	3.063197	0.024824	0.224464	-3.43089
MSTRG.56045.1	-1.0678	1.916045	-3.06291	0.024832	0.224523	-3.43126
NONHSAT196125.1	1.096499	4.646613	3.062444	0.024846	0.224568	-3.43184
ENST00000553732.1	-1.0603	5.583684	-3.06209	0.024857	0.224582	-3.43229
MSTRG.55327.1	-1.0631	3.015343	-3.06078	0.024896	0.224762	-3.43395
ENST00000526041.1	1.882793	3.755808	3.060637	0.024901	0.224762	-3.43413
lnc-SIX1-3:1	1.309718	2.38863	3.060623	0.024901	0.224762	-3.43415
MSTRG.60097.1	1.700666	3.489791	3.059234	0.024943	0.22502	-3.43591
lnc-GPATCH2-8:1	1.462368	2.914988	3.05767	0.02499	0.225224	-3.43789
lnc-CROCC-1:6	1.413758	2.318414	3.056911	0.025013	0.225371	-3.43885
NONHSAT158335.1	1.084584	4.307076	3.056568	0.025024	0.225425	-3.43929
NONHSAT195177.1	1.898149	2.990877	3.055504	0.025056	0.225426	-3.44064
lnc-ZNF550-2:1	-1.1974	2.69157	-3.05472	0.02508	0.225567	-3.44163

lnc-DAD1-4:1	1.457871	2.822519	3.053412	0.02512	0.225664	-3.44329
ENST00000648788.1	1.32841	2.587364	3.052509	0.025147	0.22585	-3.44444
NONHSAT167948.1	-1.70763	3.240131	-3.052	0.025163	0.22591	-3.44509
POC1B-AS1:29	1.120089	3.822664	3.051579	0.025176	0.225959	-3.44562
MSTRG.13494.1	-1.18168	2.711228	-3.05017	0.025219	0.226008	-3.4474
NONHSAT218909.1	2.255819	5.029496	3.049294	0.025245	0.226096	-3.44852
lnc-AKIRIN1-1:19	-1.26412	3.830162	-3.0491	0.025252	0.226096	-3.44877
FAM99B:5	-1.28365	2.112018	-3.04835	0.025274	0.226096	-3.44971
NR_125936	-1.37705	1.977331	-3.04825	0.025277	0.226096	-3.44984
NONHSAT170374.1	-1.06996	3.825964	-3.04818	0.025279	0.226096	-3.44992
NONHSAT186990.1	1.143508	1.638401	3.048124	0.025281	0.226096	-3.45
NONHSAT193268.1	-1.08132	1.901504	-3.04748	0.025301	0.226163	-3.45081
MSTRG.13328.1	-1.34189	3.078655	-3.04522	0.025371	0.226446	-3.45368
MSTRG.52476.1	1.519205	2.510614	3.045141	0.025373	0.226446	-3.45379
T345203	1.569966	5.68922	3.04444	0.025395	0.226453	-3.45468
NONHSAT177252.1	-1.02377	4.945152	-3.04439	0.025396	0.226453	-3.45474
ENST00000507304.1	1.089977	1.743039	3.044198	0.025402	0.226485	-3.45498
lnc-MYO6-9:1	1.611037	2.982643	3.043135	0.025435	0.226702	-3.45633
lnc-LRRC9-5:1	1.081314	3.176767	3.042582	0.025452	0.226829	-3.45704
T284013	1.221557	1.715675	3.042005	0.02547	0.226948	-3.45777
lnc-ZGRF1-9:1	1.060693	3.278615	3.041748	0.025478	0.226991	-3.45809
lnc-ZIC1-1:3	1.111171	1.650833	3.040104	0.025529	0.227291	-3.46018
lnc-PRPF18-13:1	1.470964	2.829087	3.038544	0.025577	0.227513	-3.46216
lnc-CCNH-6:1	-1.07637	3.557079	-3.03832	0.025584	0.227513	-3.46245
ENST00000484703.1	1.100272	1.58749	3.037286	0.025616	0.227648	-3.46376
lnc-TLE4-12:4	1.18494	3.179342	3.03652	0.02564	0.227701	-3.46473
lnc-IFNAR1-1:1	1.141388	2.73347	3.036491	0.025641	0.227701	-3.46477
lnc-SLC35G2-1:9	1.032462	3.939156	3.036432	0.025643	0.227701	-3.46485
MSTRG.14197.1	1.010147	5.716472	3.036157	0.025651	0.22774	-3.46519
lnc-LRRTM1-14:1	-1.45309	3.466685	-3.03363	0.02573	0.227931	-3.46841
NONHSAT155153.1	1.399357	2.788996	3.033533	0.025733	0.227931	-3.46853
lnc-COX11-3:1	-1.22792	3.452296	-3.03342	0.025737	0.227931	-3.46868
NONHSAT221961.1	1.91721	2.054844	3.030996	0.025813	0.22826	-3.47175
NONHSAT210535.1	2.027965	3.499275	3.030939	0.025814	0.22826	-3.47183
NONHSAT168402.1	1.317761	1.788385	3.030011	0.025844	0.228422	-3.47301
ENST00000563063.1	1.560733	2.932413	3.029804	0.02585	0.228423	-3.47327
lnc-CDHR3-1:6	1.330056	2.295725	3.029036	0.025874	0.228546	-3.47425
lnc-ATP6AP2-5:1	-2.51244	3.904095	-3.02899	0.025876	0.228546	-3.47431
lnc-SLITRK6-2:1	1.241782	1.901629	3.028767	0.025883	0.228587	-3.47459
lnc-FGF8-2:1	-1.71075	2.94239	-3.02737	0.025927	0.228745	-3.47637
ENST00000391437.2	1.03077	2.141897	3.027253	0.02593	0.228745	-3.47651
lnc-FAM84B-3:1	-1.44289	3.016115	-3.02702	0.025938	0.228773	-3.47681
NONHSAT215024.1	-1.22218	2.255056	-3.02617	0.025964	0.228849	-3.47788
ENST00000580311.1	1.113148	1.99358	3.02607	0.025968	0.228849	-3.47802
NONHSAT194730.1	-1.10571	4.754583	-3.02523	0.025994	0.229008	-3.47908
MSTRG.55287.1	-1.3783	2.21789	-3.02489	0.026005	0.229013	-3.47952
ENST00000655075.1	1.174403	2.649452	3.02364	0.026045	0.229204	-3.48111
ENST00000609313.1	1.897721	2.862723	3.023595	0.026046	0.229204	-3.48117

ENST00000382488.2	-1.34445	3.024434	-3.02259	0.026078	0.229347	-3.48245
lnc-SLC46A3-1:1	1.011951	4.48432	3.021736	0.026105	0.229497	-3.48353
NONHSAT205054.1	-1.29277	2.819913	-3.02162	0.026109	0.229497	-3.48367
NONHSAT177810.1	-1.2833	2.523503	-3.02115	0.026124	0.229498	-3.48428
NONHSAT217255.1	1.80565	2.423591	3.021111	0.026125	0.229498	-3.48433
NR_037694	1.754118	2.792291	3.020922	0.026131	0.229513	-3.48457
NONHSAT197652.1	1.181598	2.006853	3.019396	0.026179	0.229779	-3.48651
NONHSAT154316.1	-1.16575	3.253054	-3.01796	0.026225	0.229894	-3.48833
lnc-TP53TG3-64:1	-1.04433	4.229807	-3.01786	0.026228	0.229894	-3.48846
NONHSAT197956.1	1.373351	2.621664	3.017817	0.02623	0.229894	-3.48852
lnc-CHST4-1:5	1.236004	2.124675	3.017095	0.026253	0.229978	-3.48944
NONHSAT178503.1	-1.1986	2.583913	-3.01682	0.026262	0.229978	-3.48979
NR_125955	-1.08787	2.097065	-3.01657	0.02627	0.229978	-3.4901
MSTRG.53117.1	1.13437	3.109315	3.01599	0.026288	0.229988	-3.49085
lnc-NFAT5-5:1	1.161115	2.075618	3.015939	0.02629	0.229988	-3.49091
T341075	-1.68121	3.012832	-3.01546	0.026305	0.230036	-3.49152
NONHSAT214270.1	-1.09552	6.967484	-3.01448	0.026337	0.230154	-3.49277
ENST00000612739.1	-1.28281	4.489452	-3.0141	0.026349	0.230169	-3.49326
lnc-MAP9-8:1	-1.05369	14.1134	-3.01382	0.026358	0.230203	-3.49361
lnc-PGPEP1L-19:1	-1.15861	2.682324	-3.01238	0.026404	0.230421	-3.49544
MSTRG.50652.2	-1.22175	2.341433	-3.01099	0.026449	0.230645	-3.49722
lnc-PAX9-7:1	1.184335	1.621499	3.010773	0.026456	0.230645	-3.49749
T158189	1.921063	2.368075	3.008798	0.02652	0.230881	-3.50001
lnc-COL28A1-1:18	1.173081	2.247846	3.00842	0.026532	0.230926	-3.50049
NONHSAT194244.1	1.303479	2.134505	3.008176	0.02654	0.23093	-3.5008
lnc-MAS1L-1:1	1.141704	2.366409	3.00726	0.026569	0.231092	-3.50197
NONHSAT212341.1	1.066074	4.327207	3.007047	0.026576	0.23111	-3.50224
NONHSAT153852.1	-1.37689	1.947693	-3.00681	0.026584	0.231116	-3.50254
lnc-PFKP-36:2	-1.25978	2.469906	-3.00639	0.026597	0.23116	-3.50307
ENST00000657137.1	1.798912	3.364999	3.00575	0.026618	0.231236	-3.50389
NONHSAT179044.1	1.074416	2.399817	3.005164	0.026637	0.231312	-3.50464
NONHSAT187600.1	-1.00316	2.121071	-3.005	0.026643	0.231329	-3.50485
NONHSAT160508.1	-1.15167	2.345303	-3.0046	0.026656	0.231411	-3.50536
NONHSAT187608.1	1.063338	6.294171	3.004212	0.026668	0.231411	-3.50585
ENST00000417838.1	-1.1518	2.066357	-3.00321	0.026701	0.231544	-3.50712
T262078	-1.2165	3.703953	-3.00321	0.026701	0.231544	-3.50713
NONHSAT171951.1	-1.44697	2.170928	-3.00318	0.026702	0.231544	-3.50717
MSTRG.27546.1	-1.15532	2.660203	-3.00248	0.026725	0.231663	-3.50806
LINC01473:10	1.307804	2.630471	3.002452	0.026726	0.231663	-3.50809
MSTRG.67367.1	-1.19398	9.585859	-3.00047	0.02679	0.231952	-3.51062
NONHSAT159000.1	-1.03399	2.78485	-2.99859	0.026852	0.232145	-3.51302
MSTRG.19205.1	-1.36946	2.718959	-2.99831	0.026861	0.232164	-3.51337
NONHSAT204340.1	1.047728	1.952424	2.997572	0.026885	0.232239	-3.51432
NONHSAT198795.1	-1.40314	1.936458	-2.99694	0.026906	0.23237	-3.51513
NONHSAT188044.1	1.532255	1.847367	2.996892	0.026908	0.23237	-3.51519
SATB2-AS1:11	1.015467	2.936626	2.996655	0.026915	0.232395	-3.51549
lnc-CDY1B-4:1	-1.20671	5.90254	-2.99614	0.026932	0.232423	-3.51615
NONHSAT167574.1	1.315873	2.553926	2.994745	0.026978	0.232569	-3.51792

MSTRG.21381.1	1.370498	1.756007	2.993662	0.027014	0.232627	-3.51931
ENST00000668609.1	1.480298	1.878601	2.993487	0.02702	0.232627	-3.51953
ENST00000354425.4	-1.18957	4.378343	-2.9931	0.027032	0.232627	-3.52002
lnc-RTL4-4:1	1.024235	2.256809	2.993058	0.027034	0.232627	-3.52008
lnc-FH-1:1	1.434955	3.748322	2.992942	0.027038	0.232627	-3.52023
ENST00000477392.1	-1.25923	2.707931	-2.99198	0.027069	0.232745	-3.52145
ENST00000644259.1	1.696372	2.375363	2.991747	0.027077	0.232775	-3.52175
T305520	-1.33844	2.662234	-2.99149	0.027086	0.232828	-3.52208
NONHSAT189424.1	-1.40902	2.354673	-2.98959	0.027149	0.233085	-3.52451
NONHSAT157022.1	-1.21592	2.110433	-2.98895	0.02717	0.233096	-3.52533
lnc-SCGB1C2-11:1	1.206504	1.710823	2.988807	0.027175	0.233096	-3.5255
MSTRG.2071.1	1.032574	1.571218	2.988672	0.027179	0.233096	-3.52568
MSTRG.59421.1	1.498548	2.366086	2.987726	0.027211	0.233239	-3.52688
NONHSAT165018.1	1.064286	1.874771	2.987537	0.027217	0.23324	-3.52713
lnc-MTA3-11:1	-1.67257	5.065262	-2.98619	0.027262	0.233466	-3.52885
lnc-COPS8-4:4	-1.34581	2.337332	-2.98599	0.027268	0.233483	-3.5291
NONHSAT201221.1	1.575089	1.852255	2.98545	0.027286	0.233545	-3.52979
lnc-SP8-2:1	1.18415	3.845213	2.985425	0.027287	0.233545	-3.52982
ENST00000609599.1	-1.15002	4.526627	-2.98451	0.027318	0.233667	-3.53099
MSTRG.46638.1	-1.39126	2.336543	-2.98362	0.027347	0.23379	-3.53213
NONHSAT199003.1	1.059962	2.765027	2.983157	0.027363	0.233839	-3.53272
lnc-CYP7B1-10:1	1.004552	4.661844	2.981483	0.027419	0.234197	-3.53486
NONHSAT205257.1	-1.66411	2.506722	-2.98083	0.027441	0.234246	-3.5357
lnc-CNTNAP2-3:2	1.138587	4.299724	2.979874	0.027473	0.23442	-3.53691
MSTRG.53632.17	1.198009	2.100729	2.976951	0.027572	0.23472	-3.54065
ENST00000582692.2	-1.28095	1.775743	-2.97684	0.027575	0.23472	-3.54079
ENST00000509924.3	1.187298	7.114083	2.976369	0.027591	0.234791	-3.54139
NONHSAT223713.1	-1.19406	3.36514	-2.9759	0.027607	0.234885	-3.54199
MSTRG.48590.1	1.337697	2.305409	2.975656	0.027615	0.234937	-3.54231
NONHSAT154209.1	1.183464	2.737711	2.975506	0.02762	0.23494	-3.5425
NONHSAT198775.1	1.148866	4.42981	2.975024	0.027637	0.235026	-3.54311
lnc-ADAMTS10-1:1	-1.27697	2.216714	-2.97487	0.027642	0.235026	-3.54331
MSTRG.4065.1	-1.08876	1.705699	-2.9748	0.027644	0.235026	-3.5434
lnc-ZNF649-3:6	-1.4704	2.356868	-2.97411	0.027668	0.235164	-3.54428
NONHSAT214840.1	1.640191	3.69502	2.973589	0.027685	0.235232	-3.54495
NR_028471	-1.53792	5.083374	-2.97339	0.027692	0.235232	-3.5452
MSTRG.71368.1	1.709289	3.119367	2.972021	0.027738	0.23538	-3.54695
MSTRG.49845.3	1.459117	3.676321	2.97179	0.027746	0.23538	-3.54725
lnc-PRKCH-1:1	1.569848	2.12774	2.971665	0.02775	0.23538	-3.54741
lnc-EPHA6-4:1	-1.59925	2.346018	-2.97103	0.027772	0.235384	-3.54822
NONHSAT193956.1	1.123348	1.94375	2.97087	0.027778	0.235384	-3.54843
NONHSAT206072.1	1.847879	2.615978	2.970247	0.027799	0.235441	-3.54922
MSTRG.18248.1	1.428464	3.596039	2.970191	0.027801	0.235441	-3.54929
ENST00000625073.1	1.101837	1.639427	2.969425	0.027827	0.235507	-3.55027
NONHSAT184962.1	-1.32624	3.300978	-2.96843	0.027861	0.23552	-3.55155
NONHSAT185528.1	1.080543	3.109733	2.967814	0.027882	0.23552	-3.55233
ENST00000560446.1	1.552971	2.428141	2.967502	0.027892	0.23552	-3.55273
T285360	1.874684	3.323869	2.966872	0.027914	0.23552	-3.55354

ENST00000567718.1	1.343584	2.108761	2.966867	0.027914	0.23552	-3.55355
ENST00000419766.5	1.138766	3.275234	2.966338	0.027932	0.23552	-3.55422
lnc-SH3BGR-5:1	1.206234	2.108012	2.966318	0.027933	0.23552	-3.55425
NONHSAT221816.1	1.448404	3.192867	2.965876	0.027948	0.235566	-3.55481
ENST00000649638.1	1.604241	1.881597	2.965062	0.027976	0.235571	-3.55586
lnc-INHBB-2:1	1.012142	3.018522	2.964932	0.02798	0.235571	-3.55602
lnc-XRCC2-12:2	1.273635	2.662246	2.9648	0.027985	0.235571	-3.55619
NONHSAT173891.1	2.116531	3.376902	2.963561	0.028027	0.235742	-3.55778
lnc-IFIH1-1:1	-1.10821	2.480821	-2.96344	0.028032	0.235742	-3.55794
lnc-SUCO-1:1	-1.02209	4.378235	-2.9632	0.02804	0.235785	-3.55825
NONHSAT154642.1	1.046034	2.500025	2.962607	0.02806	0.235837	-3.559
ENST00000645181.1	1.262565	1.703298	2.961122	0.028111	0.236014	-3.5609
lnc-NEURL1B-3:7	1.648498	7.174581	2.961067	0.028113	0.236014	-3.56097
NONHSAT197937.1	-1.51067	4.33487	-2.9607	0.028126	0.236077	-3.56144
ENST00000657981.1	2.03651	3.88345	2.96047	0.028134	0.236101	-3.56173
lnc-TLE3-11:1	1.50018	2.685506	2.957931	0.028222	0.236357	-3.56499
NONHSAT148743.1	-1.11032	4.002347	-2.95777	0.028227	0.236357	-3.56519
lnc-TC2N-1:2	-1.49636	4.471618	-2.95751	0.028236	0.236357	-3.56552
CEBPA-DT:14	-1.06608	2.471837	-2.95672	0.028264	0.236464	-3.56654
NONHSAT215077.1	-1.36198	2.389994	-2.95625	0.02828	0.236532	-3.56714
NONHSAT168514.1	1.088606	2.00959	2.955482	0.028306	0.236591	-3.56812
lnc-FOPNL-3:1	1.4144	3.252676	2.955329	0.028312	0.236591	-3.56832
lnc-UXS1-7:1	-1.08214	1.669494	-2.95515	0.028318	0.236604	-3.56855
ENST00000426218.1	2.193178	4.061578	2.954905	0.028326	0.236616	-3.56886
lnc-RTP5-5:4	1.044772	4.322482	2.953919	0.028361	0.236744	-3.57012
lnc-GCG-3:1	1.049541	3.561587	2.95355	0.028374	0.236744	-3.5706
lnc-SPRTN-2:1	1.651571	3.437357	2.95338	0.02838	0.236745	-3.57082
NONHSAT191639.1	1.474754	2.354558	2.952535	0.028409	0.23689	-3.5719
NONHSAT201300.1	-1.55053	3.889728	-2.95221	0.02842	0.23689	-3.57232
MSTRG.43224.4	1.93934	3.175362	2.950461	0.028481	0.237091	-3.57456
NONHSAT161323.1	1.465171	2.537989	2.949661	0.028509	0.237116	-3.57558
lnc-RIPK4-4:1	1.147671	4.507645	2.948602	0.028546	0.237176	-3.57694
T162607	-1.09849	3.894555	-2.94811	0.028564	0.237243	-3.57758
NONHSAT205439.1	-1.07734	2.413719	-2.94568	0.028649	0.237509	-3.58069
NONHSAT162135.1	1.55987	2.079101	2.945068	0.028671	0.237562	-3.58147
NONHSAT193073.1	1.388078	2.99261	2.944623	0.028686	0.237672	-3.58204
NONHSAT150141.1	1.334521	2.95968	2.94413	0.028704	0.237735	-3.58267
ENST00000614815.1	-1.25585	2.88727	-2.94396	0.02871	0.237735	-3.58289
NONHSAT172924.1	-1.30623	1.927995	-2.94365	0.028721	0.237735	-3.58329
MSTRG.19434.1	-1.39879	2.345964	-2.94338	0.02873	0.237753	-3.58364
MSTRG.34381.11	1.411352	3.403291	2.943272	0.028734	0.237755	-3.58377
lnc-UBN2-1:1	-1.43495	2.070919	-2.94294	0.028746	0.237812	-3.5842
MSTRG.3006.1	1.111876	1.756814	2.942379	0.028766	0.237918	-3.58492
NONHSAT208397.1	1.391879	2.129696	2.941889	0.028783	0.237983	-3.58555
NONHSAT178033.1	-1.58461	3.466666	-2.94119	0.028808	0.238099	-3.58645
T315345	2.302575	4.123938	2.941059	0.028812	0.238109	-3.58661
MSTRG.45326.1	-1.10423	3.218433	-2.94078	0.028822	0.238151	-3.58697
NONHSAT211514.1	-1.11974	2.68628	-2.94064	0.028827	0.238151	-3.58715

ENST00000413943.1	1.663782	2.278028	2.939868	0.028854	0.238223	-3.58814
lnc-PPP4R3C-2:2	-1.40835	2.687964	-2.93886	0.02889	0.238338	-3.58944
NONHSAT185039.1	-1.72228	3.228635	-2.93868	0.028897	0.238346	-3.58966
NONHSAT197709.1	-1.34944	1.788993	-2.93843	0.028905	0.238391	-3.58998
NONHSAT190492.1	1.365907	3.31744	2.937924	0.028923	0.238471	-3.59063
ENST00000655303.1	1.126996	5.306275	2.937267	0.028947	0.238537	-3.59148
MSTRG.69815.1	1.063451	4.679113	2.936935	0.028959	0.238609	-3.5919
NONHSAT214691.1	-1.31145	3.913191	-2.93646	0.028976	0.238609	-3.59251
MSTRG.2395.1	1.380966	1.811045	2.936433	0.028977	0.238609	-3.59255
NONHSAT162615.1	-1.0292	3.958922	-2.93582	0.028998	0.238697	-3.59333
NONHSAT149602.1	-1.56762	7.442177	-2.93536	0.029015	0.238765	-3.59392
NONHSAT215392.1	-1.04132	2.681555	-2.93515	0.029022	0.238766	-3.59419
NONHSAT154432.1	1.241671	3.832698	2.93441	0.029049	0.238886	-3.59514
NONHSAT161631.1	-1.22874	3.0413	-2.93376	0.029072	0.238978	-3.59597
NONHSAT219896.1	-1.24674	2.376528	-2.93325	0.02909	0.238978	-3.59664
ENST00000658071.1	1.302598	2.923482	2.93318	0.029093	0.238978	-3.59672
MSTRG.14101.1	1.015771	3.632526	2.932886	0.029103	0.239022	-3.5971
ENST00000602530.1	1.073422	4.16472	2.932469	0.029118	0.239065	-3.59764
NONHSAT215133.1	1.223738	3.141784	2.932422	0.02912	0.239065	-3.5977
ENST00000529314.2	1.03195	3.541401	2.93242	0.02912	0.239065	-3.5977
lnc-BMP6-15:1	-1.23977	2.101031	-2.93177	0.029143	0.239123	-3.59853
lnc-NT5C2-2:1	1.716552	2.368581	2.931252	0.029162	0.239214	-3.5992
lnc-GRM4-2:2	-1.01418	2.281127	-2.93057	0.029186	0.239278	-3.60007
lnc-ROPN1B-5:1	-1.2546	2.566648	-2.92673	0.029325	0.239747	-3.605
ENST00000418282.2	1.594229	1.8894	2.926551	0.029331	0.239747	-3.60524
NONHSAT199836.1	1.34004	1.730299	2.925351	0.029374	0.239938	-3.60678
lnc-RPS13-2:1	-1.16771	2.612402	-2.92454	0.029404	0.240036	-3.60782
NONHSAT182664.1	-1.16983	2.879602	-2.92451	0.029405	0.240036	-3.60786
NONHSAT222896.1	1.848008	2.924878	2.922615	0.029474	0.240345	-3.61029
NONHSAT152675.1	1.624952	2.489224	2.920392	0.029554	0.24062	-3.61315
MSTRG.50712.1	-1.02732	3.214962	-2.91852	0.029623	0.240802	-3.61556
NONHSAT169420.1	1.479341	2.551332	2.917904	0.029645	0.240823	-3.61635
MSTRG.19790.6	-1.40623	2.248004	-2.91766	0.029654	0.240823	-3.61666
NONHSAT199666.1	-1.36923	2.434358	-2.91662	0.029692	0.240989	-3.618
lnc-SIRT1-7:1	-1.25409	3.182005	-2.91589	0.029719	0.241049	-3.61894
NONHSAT219291.1	1.583221	2.473402	2.915433	0.029736	0.241057	-3.61953
T264682	1.885861	2.397444	2.914896	0.029755	0.241174	-3.62021
MSTRG.35264.1	1.208182	3.729415	2.913355	0.029812	0.241385	-3.6222
MSTRG.51958.1	1.260499	2.512106	2.912882	0.029829	0.241483	-3.62281
NONHSAT156649.1	1.630444	2.505234	2.911737	0.029871	0.241653	-3.62428
ENST00000501718.2	-1.11514	1.998637	-2.91167	0.029874	0.241653	-3.62436
MSTRG.51564.1	1.500492	1.843877	2.910911	0.029902	0.241783	-3.62534
NONHSAT163896.1	-1.04292	2.150228	-2.9092	0.029965	0.241984	-3.62755
NONHSAT187082.1	-1.04959	3.553819	-2.9089	0.029976	0.242021	-3.62792
lnc-SRPK2-10:1	1.492126	2.928439	2.908232	0.030001	0.242099	-3.62879
MSTRG.8490.1	-1.01872	3.632668	-2.90655	0.030063	0.242369	-3.63095
MSTRG.33899.1	1.00615	5.067882	2.905895	0.030088	0.242506	-3.6318
lnc-DENND1A-6:1	1.078322	1.623688	2.905231	0.030112	0.242552	-3.63265

NONHSAT211981.1	1.323607	4.340739	2.90484	0.030127	0.24259	-3.63315
lnc-PROX1-3:1	1.131569	3.10997	2.904333	0.030146	0.242677	-3.63381
lnc-PMAIP1-8:1	1.060437	3.568138	2.90415	0.030152	0.242677	-3.63404
ENST00000515414.1	-1.22921	2.06285	-2.90382	0.030165	0.242677	-3.63447
NONHSAT205745.1	-1.22633	5.968484	-2.90373	0.030168	0.242677	-3.63458
lnc-RNF113B-6:1	-1.54518	2.155015	-2.90372	0.030168	0.242677	-3.63459
T376261	-1.12844	2.32587	-2.90334	0.030183	0.242677	-3.63508
NONHSAT222013.1	1.493463	2.271201	2.903262	0.030185	0.242677	-3.63518
lnc-TMEM35A-1:1	1.477262	2.676129	2.903259	0.030186	0.242677	-3.63519
MSTRG.28361.3	1.211409	2.31748	2.903018	0.030195	0.242703	-3.6355
NR_125985	1.404478	2.650932	2.902452	0.030216	0.242802	-3.63623
NONHSAT157929.1	1.289805	2.942755	2.90055	0.030287	0.243082	-3.63868
lnc-KLF15-3:1	-1.18757	2.05045	-2.89956	0.030324	0.243161	-3.63995
NONHSAT222062.1	1.165142	2.066195	2.899513	0.030326	0.243161	-3.64001
lnc-MSR1-7:1	1.660771	2.471337	2.898836	0.030351	0.243225	-3.64088
NONHSAT200732.1	1.254798	1.780861	2.897715	0.030393	0.243408	-3.64233
NONHSAT170265.1	1.251232	2.248547	2.897593	0.030398	0.243408	-3.64249
NONHSAT205340.1	1.115821	2.11801	2.897161	0.030414	0.243443	-3.64304
ENST00000568714.2	-1.49525	2.734293	-2.89539	0.03048	0.243674	-3.64532
MSTRG.7456.1	-1.21266	2.348777	-2.89304	0.030569	0.244026	-3.64835
lnc-CCND1-2:1	1.192667	3.349943	2.892467	0.030591	0.244142	-3.64909
NONHSAT196746.1	1.240702	4.022576	2.892243	0.030599	0.244191	-3.64938
ENST00000417612.1	-1.53235	3.60225	-2.89154	0.030626	0.244201	-3.65028
MSTRG.62695.1	1.602872	2.337332	2.891345	0.030633	0.244201	-3.65054
lnc-KLF9-4:1	-1.25443	3.506657	-2.89064	0.03066	0.244245	-3.65145
NONHSAT169336.1	-1.24315	2.351379	-2.89053	0.030664	0.244245	-3.65159
NONHSAT183216.1	-1.10792	3.427801	-2.88961	0.030699	0.24433	-3.65277
lnc-GOLGA8S-2:1	-1.16994	2.221045	-2.8891	0.030719	0.244388	-3.65343
lnc-FECH-4:2	-1.40054	2.813275	-2.88779	0.030768	0.244571	-3.65512
IL10RB-DT:15	-1.16603	4.65373	-2.88747	0.03078	0.244592	-3.65553
NONHSAT204516.1	-1.63838	3.070057	-2.88711	0.030794	0.244626	-3.656
ENST00000500525.1	-1.21098	3.194088	-2.88675	0.030808	0.244673	-3.65646
MSTRG.34252.1	1.366564	1.747551	2.886467	0.030819	0.244721	-3.65682
lnc-SH2D1B-5:2	1.085926	1.718273	2.885654	0.03085	0.244832	-3.65787
ENST00000253848.3	-1.11684	2.070831	-2.88548	0.030856	0.244859	-3.6581
ENST00000656662.1	-1.19889	8.000008	-2.88414	0.030907	0.244967	-3.65982
NONHSAT162403.1	1.614045	2.490711	2.883552	0.03093	0.244992	-3.66058
lnc-EXD1-2:1	1.019186	2.135673	2.881584	0.031005	0.245283	-3.66312
MSTRG.14835.2	-1.08485	2.071127	-2.8811	0.031024	0.245283	-3.66374
lnc-HAAO-7:2	1.248637	2.060268	2.88074	0.031038	0.245323	-3.66421
MSTRG.29561.1	1.204814	1.710529	2.880081	0.031063	0.245464	-3.66506
NONHSAT177657.1	1.019714	1.873235	2.879447	0.031087	0.245531	-3.66588
ENST00000659930.1	1.201959	2.941703	2.879328	0.031092	0.245531	-3.66603
lnc-ADGRL2-5:2	1.730233	2.875787	2.879083	0.031101	0.245556	-3.66635
lnc-TSEN15-5:1	1.239298	1.920297	2.878692	0.031117	0.245636	-3.66685
lnc-C4orf47-1:1	1.033876	2.070823	2.878338	0.03113	0.245719	-3.66731
lnc-ZNF100-8:1	1.38706	4.051272	2.878224	0.031135	0.245719	-3.66746
NONHSAT169394.1	1.469196	2.284825	2.877858	0.031149	0.245736	-3.66793

lnc-RGS18-9:1	1.136496	3.359552	2.877679	0.031156	0.245771	-3.66816
MSTRG.12641.2	1.117371	2.440173	2.877545	0.031161	0.245771	-3.66833
MSTRG.16812.3	1.036795	1.75094	2.87724	0.031173	0.245809	-3.66873
ENST00000664102.1	1.075747	2.662806	2.876144	0.031215	0.245899	-3.67014
lnc-RAD23B-12:1	2.57588	4.044157	2.875339	0.031246	0.246071	-3.67118
lnc-EPB41L4B-1:1	1.287021	2.090675	2.875305	0.031247	0.246071	-3.67122
lnc-SSH1-2:1	1.199622	4.40244	2.873586	0.031314	0.246173	-3.67344
MSTRG.2211.6	1.239951	4.138045	2.87315	0.031331	0.246287	-3.67401
lnc-KLF12-6:1	-1.43516	3.334815	-2.8723	0.031364	0.246422	-3.6751
ENST00000564485.1	-1.24598	3.090444	-2.87193	0.031378	0.246439	-3.67559
NONHSAT202694.1	1.575708	4.230784	2.871899	0.03138	0.246439	-3.67562
NONHSAT179036.1	-1.11693	1.865705	-2.87176	0.031385	0.246451	-3.6758
lnc-ZNF787-1:1	-1.21391	4.546632	-2.87149	0.031395	0.246451	-3.67614
lnc-ELOVL4-3:1	-1.18224	1.704542	-2.86881	0.0315	0.246979	-3.67961
T116321	1.298727	2.216448	2.867953	0.031533	0.247031	-3.68072
lnc-RREB1-7:1	1.247586	2.283661	2.867713	0.031543	0.247063	-3.68103
MSTRG.56586.1	-1.0474	2.816002	-2.86737	0.031556	0.247112	-3.68146
NONHSAT153263.1	1.275672	2.024159	2.86681	0.031578	0.247159	-3.68219
lnc-SFXN1-1:1	-1.07532	1.743878	-2.86603	0.031609	0.247264	-3.68321
NONHSAT157985.1	1.168047	1.869835	2.865867	0.031615	0.247287	-3.68341
lnc-NUCB2-10:1	-1.33506	2.722766	-2.86523	0.03164	0.247385	-3.68423
NONHSAT190879.1	1.226799	1.704641	2.864643	0.031663	0.247432	-3.68499
ENST00000662137.1	-1.79972	3.657693	-2.86464	0.031663	0.247432	-3.68499
NONHSAT214384.1	-1.24235	3.172887	-2.86323	0.031719	0.247674	-3.68682
lnc-FCGBP-2:1	-1.01645	1.74743	-2.86276	0.031737	0.24773	-3.68742
lnc-CCDC28A-3:1	1.546573	3.955088	2.862525	0.031746	0.247735	-3.68773
lnc-SIRT4-4:1	1.518805	3.012177	2.861999	0.031767	0.247765	-3.68841
lnc-SELENOV-4:1	1.741702	3.133065	2.861653	0.031781	0.247795	-3.68885
NONHSAT168416.1	1.35031	3.731128	2.86141	0.03179	0.247816	-3.68917
MSTRG.48253.2	1.134435	4.60616	2.860485	0.031827	0.248039	-3.69036
lnc-CCDC6-2:1	1.485303	2.13939	2.859768	0.031855	0.248133	-3.69129
T251873	1.402995	2.81701	2.859661	0.031859	0.248133	-3.69143
MSTRG.65259.1	1.587846	2.651319	2.858393	0.03191	0.248242	-3.69307
NONHSAT208016.1	-1.15136	3.892914	-2.8582	0.031917	0.248269	-3.69332
NONHSAT177017.1	1.139366	1.862695	2.857333	0.031952	0.248435	-3.69444
T278895	1.943263	2.048393	2.856195	0.031997	0.248684	-3.69591
NONHSAT201843.1	-1.18224	2.271857	-2.85578	0.032013	0.248731	-3.69645
lnc-RAB11A-4:1	1.197296	2.0776	2.855553	0.032022	0.24875	-3.69674
NONHSAT201093.1	-1.38868	3.217812	-2.85545	0.032026	0.24875	-3.69688
NONHSAT206231.1	-1.11638	1.95334	-2.85389	0.032089	0.248982	-3.6989
NONHSAT174433.1	1.279297	2.089297	2.853385	0.032109	0.24903	-3.69954
NONHSAT180573.1	1.389337	4.983534	2.852819	0.032131	0.249034	-3.70028
lnc-C9orf57-3:1	1.044444	3.63049	2.85225	0.032154	0.249034	-3.70101
lnc-ALCAM-11:1	-1.45805	2.419004	-2.85164	0.032178	0.249084	-3.7018
ENST00000426213.1	-1.40207	2.344763	-2.85085	0.03221	0.249236	-3.70282
ENST00000559394.2	-1.04896	1.978355	-2.85059	0.03222	0.249236	-3.70316
NONHSAT217968.1	1.320535	1.755865	2.84967	0.032257	0.249346	-3.70435
ENST00000570186.1	1.437893	3.452268	2.849593	0.03226	0.249346	-3.70445

MSTRG.13842.2	1.186126	2.243366	2.849308	0.032272	0.249351	-3.70482
NONHSAT210183.1	-1.33767	2.3275	-2.84879	0.032293	0.249392	-3.70549
NONHSAT182459.1	1.38566	2.182811	2.847931	0.032327	0.249527	-3.7066
MSTRG.26929.1	1.052983	3.722146	2.847815	0.032331	0.249542	-3.70675
lnc-DUXA-2:1	1.040234	1.605851	2.846568	0.032382	0.249724	-3.70836
lnc-PCDH9-11:1	-1.1123	2.689702	-2.84639	0.032389	0.249734	-3.70859
NONHSAT153477.1	1.154599	1.627444	2.845694	0.032417	0.249837	-3.70949
NONHSAT185301.1	1.315529	4.747089	2.845567	0.032422	0.249858	-3.70966
NONHSAT209189.1	1.159655	3.538382	2.844318	0.032472	0.249959	-3.71127
lnc-FBXO9-6:1	-1.06654	5.550516	-2.84386	0.032491	0.250027	-3.71187
NONHSAT177435.1	-1.40431	3.418804	-2.84338	0.03251	0.250083	-3.71249
lnc-QSER1-1:1	1.136071	3.031374	2.842274	0.032555	0.250188	-3.71392
lnc-CAND1-6:1	-1.11385	4.34491	-2.84144	0.032589	0.250262	-3.715
NONHSAT161304.1	-1.25822	2.504281	-2.84116	0.0326	0.250293	-3.71536
NONHSAT173165.1	-1.05281	2.290702	-2.84019	0.03264	0.250497	-3.71662
MSTRG.10278.1	1.227306	3.739438	2.839257	0.032677	0.250573	-3.71783
ENST00000425185.2	-1.17836	2.486914	-2.83908	0.032685	0.250573	-3.71806
ENST00000511361.1	1.019004	1.85701	2.839049	0.032686	0.250573	-3.71809
NONHSAT223926.1	-1.04277	4.988137	-2.8389	0.032692	0.250579	-3.71829
NONHSAT155779.1	1.051924	4.033024	2.838549	0.032706	0.250579	-3.71874
NONHSAT206006.1	1.524114	1.869553	2.83626	0.032799	0.25084	-3.72171
MSTRG.50711.1	-1.11236	2.991245	-2.83623	0.032801	0.25084	-3.72175
ENST00000649257.1	-1.06104	3.334265	-2.83568	0.032823	0.250936	-3.72246
NONHSAT162008.1	1.33075	2.695514	2.835298	0.032839	0.250939	-3.72295
lnc-PPEF1-2:1	1.163234	2.319365	2.835097	0.032847	0.250939	-3.72321
ENST00000581727.1	-1.16426	2.813382	-2.83503	0.03285	0.250939	-3.7233
NONHSAT191190.1	1.036361	4.473246	2.834494	0.032872	0.250969	-3.724
MSTRG.70708.1	1.225161	2.425866	2.833269	0.032922	0.251097	-3.72558
NONHSAT192126.1	1.449614	4.272595	2.832926	0.032936	0.251158	-3.72603
MSTRG.62501.1	1.219627	3.377442	2.832877	0.032938	0.251158	-3.72609
NONHSAT178510.1	-1.08919	2.634119	-2.83284	0.032939	0.251158	-3.72614
NONHSAT201341.1	1.05106	2.591833	2.83232	0.032961	0.251206	-3.72681
NONHSAT194361.1	1.160276	3.203865	2.832003	0.032974	0.251267	-3.72722
NONHSAT183398.1	-1.28234	2.265042	-2.83184	0.03298	0.25129	-3.72743
NR_040080	1.149041	3.493079	2.831635	0.032989	0.25129	-3.7277
lnc-PLCG2-1:20	1.178513	2.82801	2.831428	0.032997	0.251295	-3.72797
NONHSAT193771.1	1.305798	2.724094	2.831427	0.032997	0.251295	-3.72797
lnc-DOC2B-2:2	1.176118	3.462103	2.83106	0.033012	0.251372	-3.72844
lnc-TP53TG3F-11:1	1.073868	4.530656	2.830778	0.033024	0.251394	-3.72881
lnc-CCL2-13:1	1.04352	1.722134	2.830581	0.033032	0.251394	-3.72906
NONHSAT194932.1	-1.00947	5.377592	-2.82995	0.033058	0.251453	-3.72988
lnc-ANKRD10-7:1	-1.06027	1.877594	-2.82989	0.03306	0.251453	-3.72995
MSTRG.65428.2	-1.21249	2.276501	-2.82961	0.033072	0.251486	-3.73033
T350762	1.085853	4.86113	2.829451	0.033079	0.251486	-3.73053
lnc-AKTIP-7:1	1.246709	2.794027	2.829225	0.033088	0.251486	-3.73082
ENST00000418426.1	1.246928	1.759363	2.82888	0.033102	0.251505	-3.73127
ENST00000413406.1	-1.14969	2.776855	-2.82814	0.033133	0.25154	-3.73223
NONHSAT159681.1	1.441033	1.868065	2.828101	0.033134	0.25154	-3.73228

NR_120650	1.843561	2.729048	2.827139	0.033174	0.25167	-3.73353
NONHSAT170054.1	-1.52425	3.192896	-2.82661	0.033196	0.251742	-3.73421
lnc-GBP5-3:4	1.647317	1.954279	2.826387	0.033205	0.251745	-3.7345
NR_103835	-1.01915	5.058092	-2.82473	0.033274	0.251997	-3.73664
lnc-PATE4-1:1	1.487909	2.307263	2.824679	0.033276	0.251997	-3.73671
lnc-MUC20-67:148	1.448661	1.912549	2.824334	0.03329	0.251997	-3.73716
ENST00000555303.1	-1.07558	1.662335	-2.82431	0.033291	0.251997	-3.7372
MSTRG.30622.1	1.490369	2.204807	2.823809	0.033312	0.252062	-3.73784
ENST00000658933.1	-1.0991	2.424166	-2.82231	0.033374	0.252291	-3.73979
lnc-KLHL29-4:1	1.435914	2.899578	2.822272	0.033376	0.252291	-3.73983
lnc-GCM1-2:1	1.455803	1.786033	2.822156	0.033381	0.252309	-3.73999
NONHSAT169160.1	1.033951	3.285378	2.821946	0.033389	0.252337	-3.74026
lnc-MTDH-1:1	-1.07333	3.870022	-2.82165	0.033402	0.252374	-3.74064
T187593	-1.0092	2.233629	-2.82109	0.033425	0.252374	-3.74136
NONHSAT197417.1	1.155646	2.137945	2.820805	0.033437	0.252374	-3.74174
NONHSAT218454.1	-1.15071	6.203711	-2.82051	0.033449	0.252374	-3.74212
ENST00000661858.1	1.12024	4.037058	2.820395	0.033454	0.25239	-3.74227
lnc-IFT20-11:1	1.272345	2.734871	2.81983	0.033478	0.252489	-3.743
NONHSAT198401.1	-1.54245	2.621865	-2.81937	0.033497	0.252506	-3.7436
NONHSAT148322.1	1.03585	3.09203	2.819202	0.033504	0.25252	-3.74382
NONHSAT158167.1	-1.2826	3.13973	-2.81889	0.033517	0.25258	-3.74422
NONHSAT216339.1	1.436261	2.252644	2.818552	0.033531	0.252589	-3.74466
lnc-ACHE-3:1	-1.39666	5.051009	-2.81839	0.033538	0.252589	-3.74488
lnc-CHODL-3:2	1.480893	4.271273	2.816801	0.033604	0.252975	-3.74693
lnc-MEI4-12:1	1.219799	1.705882	2.816507	0.033617	0.253017	-3.74731
lnc-KIAA1755-12:1	-1.15147	2.882778	-2.81646	0.033618	0.253017	-3.74737
lnc-COX10-7:1	-1.12477	2.028566	-2.8157	0.03365	0.253115	-3.74836
NONHSAT174627.1	-1.27714	4.458207	-2.81534	0.033666	0.253154	-3.74883
NONHSAT193280.1	-1.27029	2.169717	-2.8126	0.033781	0.253471	-3.75238
lnc-WNT10A-2:1	1.285762	2.801285	2.8124	0.033789	0.253516	-3.75264
NONHSAT190356.1	-1.01335	1.619513	-2.8117	0.033819	0.253662	-3.75355
lnc-TTPA-1:8	-1.40766	5.674085	-2.81162	0.033822	0.253669	-3.75366
NONHSAT222282.1	-1.02031	1.817209	-2.81116	0.033842	0.253708	-3.75425
MSTRG.1679.1	-1.2649	2.030101	-2.81036	0.033875	0.25384	-3.75529
lnc-HS1BP3-4:1	-1.98264	2.865192	-2.80998	0.033892	0.253925	-3.75579
lnc-CXCR3-14:1	1.288191	2.618658	2.809788	0.0339	0.253963	-3.75603
NONHSAT202594.1	1.324877	2.444504	2.809677	0.033904	0.253963	-3.75618
lnc-MRPL39-34:1	1.979709	3.197054	2.809044	0.033931	0.254088	-3.757
ENST00000433344.1	-1.48734	3.134937	-2.80895	0.033935	0.254098	-3.75712
lnc-OCA2-6:1	1.209952	2.248017	2.80876	0.033943	0.254103	-3.75737
NONHSAT187945.1	1.314156	2.611027	2.808608	0.03395	0.254128	-3.75756
MSTRG.50782.1	1.174262	1.637236	2.808114	0.03397	0.254232	-3.75821
NONHSAT178959.1	1.366975	2.565924	2.807614	0.033992	0.254321	-3.75885
lnc-FKBPL-1:1	-1.36305	2.356036	-2.80708	0.034014	0.254399	-3.75955
NONHSAT193633.1	-1.40457	3.13765	-2.80693	0.034021	0.254399	-3.75974
lnc-CNTNAP3B-14:1	1.905482	3.328694	2.806398	0.034043	0.254489	-3.76043
lnc-FH-2:1	1.025756	2.257765	2.806356	0.034045	0.254489	-3.76049
NONHSAT210723.1	-1.05474	3.840025	-2.80611	0.034056	0.254525	-3.76081

lnc-HLTF-4:1	-1.22137	3.397828	-2.806	0.03406	0.254525	-3.76095
lnc-RASA3-8:1	-1.03468	3.776348	-2.80574	0.034071	0.254537	-3.76129
lnc-EBF1-1:1	-1.01087	1.714962	-2.80473	0.034114	0.254591	-3.7626
lnc-CDK5RAP1-2:1	-1.19897	2.202573	-2.80427	0.034134	0.254682	-3.7632
lnc-SEC11A-3:1	-1.215	3.280223	-2.80411	0.034141	0.254714	-3.76341
MSTRG.69717.3	1.055473	3.132197	2.803133	0.034182	0.254797	-3.76467
ENST00000606998.1	1.046617	1.713296	2.802572	0.034206	0.254821	-3.7654
LINC01504:1	1.548244	3.272666	2.80244	0.034212	0.254821	-3.76557
MSTRG.55011.9	-1.41475	2.790137	-2.80192	0.034234	0.254821	-3.76625
NONHSAT156407.1	1.687177	3.191262	2.80123	0.034264	0.254863	-3.76715
NONHSAT182761.1	1.326354	2.137536	2.801166	0.034266	0.254863	-3.76723
NONHSAT205845.1	1.267847	2.509396	2.801086	0.03427	0.254863	-3.76733
lnc-RNF187-3:3	-1.09667	2.955955	-2.80101	0.034273	0.254863	-3.76743
NONHSAT169946.1	-1.26292	3.026484	-2.80098	0.034275	0.254863	-3.76748
NONHSAT168915.1	1.765676	3.489816	2.797584	0.03442	0.255456	-3.77188
NONHSAT218493.1	1.342046	3.057707	2.796735	0.034457	0.255577	-3.77299
MSTRG.43948.1	1.098441	3.136894	2.79628	0.034476	0.255594	-3.77358
MSTRG.69795.1	1.162602	2.531472	2.796027	0.034487	0.255634	-3.77391
NONHSAT211342.1	-1.32748	2.242502	-2.79553	0.034509	0.255697	-3.77455
NR_104672	1.853855	3.158549	2.795275	0.03452	0.255729	-3.77488
ENST00000590241.1	1.34513	2.680621	2.794107	0.03457	0.255777	-3.7764
NONHSAT210356.1	1.048008	2.808183	2.793732	0.034586	0.255777	-3.77689
lnc-TRIM49B-1:1	1.891706	2.518467	2.79318	0.03461	0.255793	-3.77761
lnc-HOXC13-5:1	1.558047	2.944359	2.792882	0.034623	0.255851	-3.77799
MSTRG.55324.1	1.349644	2.357973	2.79249	0.03464	0.255901	-3.7785
NONHSAT161115.1	-1.5244	3.270017	-2.79217	0.034654	0.255967	-3.77892
lnc-GLRX3-2:6	1.580712	2.547085	2.791822	0.034669	0.255984	-3.77937
NONHSAT163794.1	-1.28811	2.178659	-2.79097	0.034706	0.256164	-3.78048
NONHSAT211802.1	1.527757	2.420375	2.789536	0.034768	0.256375	-3.78234
NONHSAT156184.1	1.316909	2.985568	2.789012	0.034791	0.256493	-3.78303
NONHSAT206354.1	-1.1433	3.073966	-2.78852	0.034813	0.256493	-3.78366
lnc-TXNDC2-8:1	-1.00627	3.807134	-2.78846	0.034815	0.256493	-3.78374
MSTRG.67348.1	1.160548	8.296154	2.788427	0.034817	0.256493	-3.78379
ENST00000418004.5	-1.14728	2.390142	-2.78784	0.034842	0.256549	-3.78455
lnc-BAG3-4:5	-1.07341	4.246168	-2.78766	0.03485	0.256586	-3.78478
MSTRG.44410.1	1.156809	1.740471	2.786282	0.03491	0.256774	-3.78658
lnc-COBLL1-3:1	-1.04966	2.748611	-2.78507	0.034963	0.256972	-3.78816
lnc-ZNF433-2:1	-1.84401	4.126916	-2.78488	0.034972	0.257013	-3.7884
lnc-INHBC-1:3	-1.15799	3.500403	-2.78398	0.035011	0.257227	-3.78957
NONHSAT187660.1	-1.16419	3.489498	-2.78368	0.035024	0.257227	-3.78996
NONHSAT178270.1	-1.25693	3.02481	-2.78306	0.035051	0.257291	-3.79076
NONHSAT204530.1	1.077327	2.926278	2.782079	0.035094	0.25739	-3.79204
lnc-ZNF704-8:1	1.170988	1.677383	2.781673	0.035112	0.25745	-3.79257
lnc-SFTPB-2:1	1.231061	3.63416	2.780309	0.035172	0.257717	-3.79435
lnc-EXD2-9:1	-1.20495	3.175012	-2.77942	0.035211	0.257886	-3.7955
lnc-ESPN-5:4	1.190363	2.102971	2.778607	0.035247	0.257941	-3.79656
NONHSAT208124.1	-1.18084	1.986241	-2.77814	0.035268	0.258024	-3.79716
NONHSAT171942.1	-1.15966	2.325873	-2.778	0.035274	0.25804	-3.79735

NONHSAT215876.1	1.323197	3.065995	2.777975	0.035275	0.25804	-3.79738
ENST00000434195.1	1.093604	4.003045	2.777647	0.03529	0.258091	-3.79781
MSTRG.15843.1	1.745654	6.761313	2.777161	0.035311	0.258116	-3.79844
MSTRG.54666.1	1.364201	2.042657	2.776739	0.03533	0.258148	-3.79899
NONHSAT203976.1	-1.23971	2.002419	-2.77667	0.035333	0.258148	-3.79908
lnc-ATP6V1C1-2:1	-1.22763	1.822711	-2.77626	0.035351	0.258179	-3.79961
NONHSAT168770.1	1.376398	2.723071	2.775986	0.035363	0.258197	-3.79997
lnc-WWC2-6:1	-1.06345	7.05932	-2.77592	0.035366	0.258197	-3.80006
NONHSAT218726.1	-1.47545	3.437419	-2.77542	0.035388	0.258197	-3.80071
MSTRG.58292.5	1.243255	1.640921	2.775386	0.03539	0.258197	-3.80075
NONHSAT156420.1	1.654669	3.124644	2.77535	0.035391	0.258197	-3.8008
ENST00000453972.1	1.319155	2.772421	2.773961	0.035453	0.258498	-3.80261
NONHSAT201101.1	1.574947	3.040584	2.773488	0.035474	0.258576	-3.80322
NONHSAT200428.1	-1.72425	3.293098	-2.77304	0.035494	0.258611	-3.80381
NONHSAT219902.1	-1.02926	3.790814	-2.77294	0.035498	0.258618	-3.80394
MSTRG.63708.1	1.112244	3.92112	2.772755	0.035507	0.258627	-3.80418
lnc-ATP2A2-1:1	1.095621	1.880606	2.771816	0.035548	0.25873	-3.8054
NONHSAT197097.1	-1.03311	2.098777	-2.77143	0.035566	0.258796	-3.80591
ENST00000647804.1	1.257437	2.319381	2.770999	0.035585	0.258823	-3.80647
NONHSAT163791.1	1.388113	2.770191	2.76958	0.035648	0.258989	-3.80831
NONHSAT194018.1	2.705415	2.434185	2.769579	0.035648	0.258989	-3.80831
NONHSAT168988.1	-1.38321	3.497365	-2.76921	0.035665	0.258992	-3.8088
NONHSAT175992.1	1.477178	2.15686	2.769099	0.03567	0.258992	-3.80894
lnc-NDST1-3:1	1.596503	3.093085	2.76852	0.035696	0.259049	-3.80969
T372664	-1.14975	8.130303	-2.76824	0.035708	0.259053	-3.81005
NONHSAT205246.1	-1.46767	1.968621	-2.76759	0.035737	0.259143	-3.81091
MSTRG.2379.1	-1.12935	2.765653	-2.76754	0.035739	0.259143	-3.81096
ENST00000567907.1	1.358082	2.749707	2.766692	0.035778	0.259271	-3.81207
lnc-LAD1-1:2	1.507541	2.37394	2.766143	0.035802	0.259314	-3.81279
lnc-HNRNPM-6:1	1.169198	2.316467	2.765985	0.035809	0.259314	-3.81299
LINC00955:6	1.046049	1.665883	2.765736	0.035821	0.259336	-3.81332
MSTRG.19529.5	1.289165	1.740166	2.765239	0.035843	0.259342	-3.81397
NONHSAT188336.1	1.355476	2.502059	2.764598	0.035872	0.259466	-3.8148
NONHSAT164531.1	1.700853	2.196702	2.763878	0.035904	0.259609	-3.81574
ENST00000481312.1	1.042961	3.693963	2.763055	0.035941	0.259728	-3.81681
NONHSAT197953.1	1.125329	3.781405	2.762153	0.035982	0.25978	-3.81799
NONHSAT172238.1	-1.0833	2.84551	-2.7616	0.036007	0.25983	-3.81871
ENST00000646143.2	1.946612	5.292915	2.760021	0.036078	0.260104	-3.82077
NONHSAT202423.1	1.05487	3.537984	2.759635	0.036096	0.260166	-3.82127
MSTRG.45727.2	1.052067	2.138328	2.756794	0.036225	0.260749	-3.82497
lnc-CHRDL2-4:1	1.292524	5.437803	2.756277	0.036248	0.260782	-3.82565
ENST00000453924.1	-1.21268	2.839241	-2.75613	0.036255	0.260782	-3.82584
NONHSAT206320.1	1.000498	2.440145	2.756086	0.036257	0.260782	-3.8259
NONHSAT185781.1	1.301672	2.181712	2.756038	0.036259	0.260782	-3.82596
NONHSAT183119.1	-1.21636	1.691506	-2.75593	0.036264	0.260791	-3.8261
NONHSAT176492.1	-1.34599	2.870657	-2.75582	0.036269	0.260809	-3.82625
MSTRG.10705.1	1.492189	2.756323	2.75567	0.036276	0.260833	-3.82644
MSTRG.36139.1	1.087199	4.5249	2.755166	0.036299	0.260847	-3.8271

lnc-TMEM53-1:1	1.576502	3.161884	2.755057	0.036304	0.260847	-3.82724
LINC01284:4	1.302479	1.805388	2.754787	0.036316	0.260847	-3.82759
NONHSAT209233.1	1.456861	2.266073	2.754648	0.036322	0.260847	-3.82777
lnc-SLC25A28-1:2	1.512616	2.327686	2.75152	0.036465	0.261525	-3.83185
NONHSAT188153.1	-1.2561	2.057236	-2.75076	0.0365	0.261593	-3.83284
lnc-CYP3A7-2:7	1.074925	4.34212	2.749227	0.036571	0.261805	-3.83484
NONHSAT181210.1	1.83543	2.750317	2.748805	0.03659	0.261847	-3.83539
MSTRG.63581.1	1.23707	4.002816	2.748463	0.036606	0.261898	-3.83584
lnc-TRNP1-4:1	1.555611	2.966786	2.748149	0.03662	0.261935	-3.83625
NONHSAT207682.1	-1.46713	5.794474	-2.74799	0.036628	0.261948	-3.83646
NONHSAT156216.1	1.02846	2.372896	2.747886	0.036632	0.261963	-3.83659
NONHSAT218898.1	1.178366	3.574502	2.747509	0.03665	0.262033	-3.83708
lnc-TMED10-4:1	-1.24069	2.350351	-2.74721	0.036663	0.262092	-3.83747
lnc-TRIM13-7:1	-1.03313	2.187099	-2.74704	0.036671	0.262093	-3.8377
MSTRG.45201.1	1.363464	4.772671	2.747002	0.036673	0.262093	-3.83774
lnc-STK26-11:1	1.323403	2.87595	2.746818	0.036682	0.262103	-3.83798
NONHSAT151609.1	-1.50376	4.983954	-2.74612	0.036714	0.262172	-3.8389
T102435	1.684199	3.391764	2.745643	0.036736	0.262238	-3.83952
ENST00000422600.1	1.127816	3.144822	2.745376	0.036748	0.262283	-3.83987
MSTRG.12580.1	-1.1221	2.348802	-2.74533	0.03675	0.262283	-3.83992
MSTRG.49474.1	-1.00403	3.918826	-2.74426	0.0368	0.262381	-3.84132
lnc-TMTC3-18:2	-1.28142	5.440329	-2.74382	0.03682	0.262404	-3.84189
NONHSAT181420.1	-1.26336	5.40145	-2.74339	0.03684	0.262404	-3.84246
NONHSAT224206.1	1.490997	3.035231	2.74332	0.036843	0.262404	-3.84255
lnc-RFX8-2:1	-1.08671	2.059884	-2.74313	0.036852	0.262417	-3.8428
NONHSAT157935.1	1.371743	1.76324	2.742939	0.036861	0.26244	-3.84305
MSTRG.16892.1	1.457971	3.070369	2.742861	0.036865	0.26244	-3.84315
NONHSAT180343.1	1.709601	3.099968	2.742839	0.036866	0.26244	-3.84318
NONHSAT172990.1	1.551223	3.509034	2.742632	0.036875	0.26244	-3.84345
MSTRG.35465.1	1.009772	2.289324	2.742611	0.036876	0.26244	-3.84347
lnc-BMP5-3:1	-1.02956	3.297167	-2.74248	0.036882	0.262449	-3.84365
MSTRG.14113.1	1.189386	3.60145	2.741985	0.036905	0.262573	-3.84429
MSTRG.1479.1	1.549809	2.406442	2.740747	0.036963	0.262685	-3.84591
MSTRG.23277.1	-1.01644	9.257805	-2.7406	0.036969	0.262685	-3.8461
NONHSAT167081.1	1.409788	3.301732	2.740157	0.03699	0.262714	-3.84668
NONHSAT184132.1	-1.23275	3.535441	-2.73909	0.03704	0.262881	-3.84807
MSTRG.22132.2	1.06937	1.644503	2.738694	0.037058	0.262898	-3.84859
NONHSAT152586.1	-1.1069	1.962477	-2.73815	0.037084	0.262994	-3.84929
ENST00000560153.2	-1.08839	4.416493	-2.73755	0.037112	0.263051	-3.85008
NONHSAT169431.1	1.074241	1.602633	2.736548	0.037158	0.263224	-3.85139
NONHSAT204454.1	1.732361	4.038334	2.73595	0.037186	0.263377	-3.85217
NONHSAT166089.1	1.147636	3.054766	2.735824	0.037192	0.263383	-3.85233
lnc-ZDHHC17-7:1	-1.046	4.992958	-2.73561	0.037202	0.263405	-3.85261
MSTRG.48996.1	-1.30762	2.582217	-2.7341	0.037273	0.263643	-3.85458
NONHSAT160987.1	1.314556	2.619202	2.73394	0.037281	0.263663	-3.85479
NONHSAT210660.1	1.427516	2.949462	2.732351	0.037355	0.264054	-3.85687
NONHSAT197414.1	1.180927	2.612861	2.731531	0.037394	0.26413	-3.85794
NONHSAT183693.1	1.024881	1.691683	2.731176	0.037411	0.26414	-3.8584

lnc-PRPF40B-1:3	-1.33317	2.544275	-2.73023	0.037455	0.264356	-3.85964
lnc-RREB1-9:4	-1.41657	2.760264	-2.72959	0.037486	0.264482	-3.86048
lnc-SH3RF3-2:1	1.448983	2.913117	2.729376	0.037495	0.264496	-3.86075
ENST00000548738.1	1.257808	2.484726	2.7293	0.037499	0.264502	-3.86085
lnc-LRIG1-5:1	1.270369	4.679466	2.729192	0.037504	0.264502	-3.861
ENST00000630472.1	1.112063	2.367457	2.72876	0.037525	0.264553	-3.86156
NONHSAT210756.1	-1.4176	2.549029	-2.72753	0.037583	0.264778	-3.86316
lnc-ZNF681-5:1	1.147673	1.607355	2.727276	0.037595	0.264808	-3.8635
ENST00000520543.1	1.658338	5.464359	2.726185	0.037647	0.265017	-3.86493
ENST00000563347.1	1.012159	1.604392	2.725875	0.037661	0.265045	-3.86533
NONHSAT175661.1	-1.2647	1.780315	-2.72563	0.037673	0.265045	-3.86565
T191068	1.135115	5.066245	2.725062	0.0377	0.265138	-3.86639
MSTRG.66289.1	1.099491	3.954599	2.724771	0.037714	0.26514	-3.86677
lnc-PCDH7-16:1	-1.25402	2.380003	-2.7246	0.037722	0.26514	-3.86699
NONHSAT179294.1	1.105395	1.963271	2.723746	0.037762	0.265227	-3.86811
NONHSAT170348.1	1.053234	5.345052	2.723103	0.037793	0.265314	-3.86895
lnc-TRABD2A-6:1	-1.32085	4.258181	-2.72258	0.037818	0.265362	-3.86964
lnc-NXF2B-1:1	1.258728	2.587398	2.722522	0.037821	0.265362	-3.86971
NONHSAT196989.1	-1.02361	1.685277	-2.7224	0.037826	0.265362	-3.86987
NONHSAT190106.1	1.280764	2.154152	2.722351	0.037829	0.265362	-3.86994
ENST00000499137.6	-1.17859	2.493032	-2.72184	0.037853	0.265459	-3.8706
NONHSAT188130.1	1.719108	3.544561	2.720888	0.037899	0.265645	-3.87185
lnc-SLC13A1-1:2	-1.23096	2.74175	-2.72078	0.037904	0.265645	-3.87199
NONHSAT164390.1	1.40102	3.143843	2.720569	0.037914	0.265645	-3.87226
T239058	-1.46247	2.317165	-2.71912	0.037983	0.265809	-3.87416
lnc-TAF5L-4:2	-1.34899	3.362631	-2.71909	0.037985	0.265809	-3.8742
lnc-TMEM17-6:1	-1.01669	1.818894	-2.71723	0.038074	0.266143	-3.87663
NR_024236	-1.05568	7.486792	-2.71723	0.038074	0.266143	-3.87663
MSTRG.37775.1	1.041783	4.14238	2.717076	0.038081	0.266143	-3.87683
NONHSAT161080.1	1.781446	2.844504	2.717017	0.038084	0.266143	-3.87691
lnc-BRF1-65:1	1.546937	2.980594	2.716881	0.038091	0.266143	-3.87709
lnc-RAP1A-2:1	1.25935	1.730995	2.716543	0.038107	0.266164	-3.87753
MSTRG.41652.30	1.017798	3.400804	2.715837	0.038141	0.266254	-3.87845
lnc-SUMO1-1:3	-1.53437	3.149327	-2.71488	0.038187	0.266367	-3.8797
lnc-PPP1R3D-5:1	1.382101	2.033282	2.714571	0.038202	0.266367	-3.88011
NONHSAT186983.1	1.182124	2.918619	2.713844	0.038237	0.266401	-3.88106
NONHSAT190586.1	1.41716	7.202784	2.713719	0.038243	0.266401	-3.88122
NONHSAT201462.1	1.603556	2.936206	2.71299	0.038278	0.266492	-3.88217
NONHSAT177084.1	-1.10115	3.521711	-2.71282	0.038286	0.26653	-3.88239
NONHSAT154009.1	1.317751	1.749947	2.712596	0.038297	0.266534	-3.88269
NONHSAT187827.1	1.246226	2.358428	2.712286	0.038312	0.266564	-3.8831
lnc-RASGRP3-7:1	-1.1141	2.104809	-2.71186	0.038333	0.266634	-3.88366
T285145	1.01259	2.491151	2.71172	0.038339	0.266648	-3.88384
T050176	1.221219	2.08586	2.711595	0.038345	0.266663	-3.884
lnc-JPH3-6:2	1.023993	3.428689	2.711304	0.03836	0.266692	-3.88438
MSTRG.33107.1	-1.10797	2.422267	-2.71014	0.038416	0.266809	-3.88591
NONHSAT153363.1	-1.3319	1.967391	-2.70994	0.038425	0.266826	-3.88616
NONHSAT206343.1	1.169647	2.709392	2.708936	0.038474	0.267029	-3.88748

lnc-FOXG1-9:1	-1.30227	3.006912	-2.70877	0.038482	0.267048	-3.88769
LINC01010:9	-1.16196	2.501258	-2.70714	0.038562	0.267341	-3.88983
lnc-NIPSNAP3B-4:1	1.267789	2.51289	2.706931	0.038572	0.267346	-3.8901
T105475	2.059865	2.443086	2.706746	0.038581	0.267346	-3.89034
NONHSAT171724.1	1.2125	1.973384	2.706325	0.038601	0.267368	-3.89089
MSTRG.55435.1	-1.36726	3.127686	-2.70537	0.038648	0.267458	-3.89214
lnc-HNRNPU-9:1	1.018513	2.215522	2.704942	0.038669	0.267458	-3.89271
NONHSAT201647.1	-1.49253	2.829215	-2.70486	0.038673	0.267458	-3.89282
NONHSAT187452.1	1.721281	3.054235	2.704851	0.038673	0.267458	-3.89282
lnc-GPR65-17:1	-1.57976	6.791746	-2.70217	0.038804	0.26779	-3.89633
ENST00000427132.1	-1.32562	2.312255	-2.70165	0.03883	0.267838	-3.89701
NONHSAT161188.1	1.126103	2.880368	2.700642	0.038879	0.267978	-3.89833
ENST00000563151.1	-1.42689	2.672108	-2.70029	0.038897	0.268043	-3.8988
lnc-MARCKS-15:1	1.43963	1.827964	2.699692	0.038926	0.268135	-3.89958
MSTRG.53678.1	1.15335	2.163927	2.699557	0.038933	0.268148	-3.89975
NONHSAT185708.1	1.571254	1.89411	2.699408	0.03894	0.268148	-3.89995
NONHSAT207345.1	-1.19346	2.541249	-2.69938	0.038941	0.268148	-3.89998
NONHSAT221730.1	-1.60121	3.127354	-2.69889	0.038965	0.268222	-3.90062
T345100	1.080584	1.838571	2.69809	0.039005	0.268227	-3.90167
ENST00000661314.1	1.004726	3.87282	2.697458	0.039036	0.268236	-3.9025
NONHSAT158553.1	-1.36542	3.084423	-2.69725	0.039046	0.268236	-3.90278
lnc-ACVR1-4:1	1.392255	2.482299	2.696877	0.039065	0.268236	-3.90326
NONHSAT171502.1	1.190622	2.275367	2.696651	0.039076	0.268236	-3.90356
MSTRG.57595.1	1.365601	1.783551	2.695769	0.03912	0.268396	-3.90471
ENST00000648110.1	1.36159	2.220073	2.694978	0.039159	0.268544	-3.90575
NONHSAT199573.1	1.28522	2.639137	2.694439	0.039185	0.268618	-3.90646
MSTRG.50656.1	-1.54902	3.056489	-2.69417	0.039199	0.268672	-3.90681
lnc-ZFP42-8:1	-1.11312	1.900609	-2.6941	0.039202	0.268678	-3.9069
NONHSAT169853.1	1.065801	2.533724	2.693841	0.039215	0.268698	-3.90724
lnc-OFD1-2:1	1.103414	1.638942	2.692868	0.039263	0.26875	-3.90851
lnc-PTCD2-7:1	-1.2129	3.128426	-2.69195	0.039309	0.268972	-3.90972
ENST00000668076.1	1.055594	4.173331	2.690878	0.039362	0.269148	-3.91112
NONHSAT207179.1	-1.2233	2.011782	-2.69052	0.03938	0.269166	-3.91159
MSTRG.55418.1	-1.12882	3.137771	-2.69038	0.039387	0.269175	-3.91177
lnc-RACGAP1-1:13	-1.92347	3.756213	-2.68892	0.03946	0.269434	-3.91369
NONHSAT210258.1	-1.05728	1.852013	-2.68887	0.039462	0.269434	-3.91375
ENST00000448643.1	-1.09439	2.013215	-2.68846	0.039483	0.269503	-3.91429
ENST00000668740.1	1.435859	1.826365	2.687993	0.039506	0.269606	-3.9149
lnc-OR52H1-1:1	1.107353	1.797151	2.687845	0.039513	0.26962	-3.91509
lnc-DENND3-1:2	1.064151	2.305316	2.687383	0.039537	0.269645	-3.9157
NONHSAT192443.1	-1.22122	2.19353	-2.68657	0.039577	0.269796	-3.91676
ENST00000653425.1	-1.34134	4.178081	-2.68655	0.039578	0.269796	-3.9168
LINC01180:2	1.169955	3.586079	2.685576	0.039627	0.270003	-3.91807
NONHSAT187591.1	1.263944	1.724811	2.685309	0.03964	0.270003	-3.91842
lnc-IGFBP7-2:1	1.054803	2.007526	2.685272	0.039642	0.270003	-3.91847
T344722	-1.77235	4.637997	-2.68512	0.03965	0.270005	-3.91867
NONHSAT210236.1	1.386747	2.358818	2.684946	0.039659	0.270027	-3.91889
lnc-DDX25-1:2	-1.0625	1.756039	-2.68441	0.039686	0.270119	-3.9196

NONHSAT206458.1	-1.28445	2.61425	-2.684	0.039706	0.270204	-3.92013
MSTRG.52989.1	1.182012	4.603647	2.683692	0.039722	0.270265	-3.92054
ENST00000655799.1	1.496698	2.854996	2.682791	0.039767	0.270435	-3.92172
lnc-HLA-DRB1-7:1	1.718115	3.325061	2.68235	0.039789	0.27052	-3.9223
lnc-CPO-1:5	-1.04176	4.821923	-2.68201	0.039806	0.27056	-3.92274
lnc-NF2-2:1	-1.05171	2.834369	-2.682	0.039807	0.27056	-3.92276
NONHSAT174630.1	1.253187	2.154948	2.681629	0.039825	0.270653	-3.92324
NONHSAT149097.1	-1.01115	1.82064	-2.68139	0.039837	0.270653	-3.92355
ENST00000649077.1	1.460084	2.058424	2.681369	0.039839	0.270653	-3.92358
MSTRG.32971.1	-1.35363	2.903483	-2.68066	0.039874	0.270783	-3.92451
NONHSAT201918.1	1.172494	2.480313	2.680027	0.039906	0.270803	-3.92534
MSTRG.61915.1	-1.0606	1.767528	-2.67951	0.039933	0.270803	-3.92602
NONHSAT222334.1	1.324581	2.36897	2.679491	0.039933	0.270803	-3.92604
MSTRG.34127.1	1.108796	5.95317	2.678554	0.039981	0.270994	-3.92727
MSTRG.38899.7	-1.14005	2.213661	-2.67782	0.040018	0.2711	-3.92823
NONHSAT176078.1	-1.09726	3.109527	-2.6773	0.040044	0.27117	-3.92892
lnc-STOM-7:3	-1.322	3.466053	-2.67719	0.04005	0.271175	-3.92906
lnc-ZFAND5-1:1	-1.00867	2.413634	-2.67654	0.040083	0.271286	-3.92992
lnc-CDRT15-5:1	-1.17059	5.413398	-2.67594	0.040113	0.271381	-3.9307
NONHSAT193262.1	-1.19329	5.261257	-2.67586	0.040118	0.271381	-3.93081
lnc-RAB30-6:1	-1.32516	2.881928	-2.67579	0.040121	0.271381	-3.93089
NONHSAT176466.1	-1.40234	7.889981	-2.67579	0.040121	0.271381	-3.9309
lnc-GRIA3-3:1	-1.88701	3.332285	-2.67522	0.04015	0.271503	-3.93165
lnc-LMBR1-8:2	-1.29696	3.238561	-2.67464	0.04018	0.271657	-3.93241
NONHSAT160653.1	1.691734	3.312168	2.674336	0.040195	0.271698	-3.9328
ENST00000426771.1	-1.2384	3.039756	-2.6742	0.040202	0.271698	-3.93298
ENST00000420237.1	1.288823	2.816692	2.673773	0.040224	0.271731	-3.93354
ENST00000558888.2	-1.38591	1.99849	-2.67366	0.040229	0.271731	-3.93368
lnc-SLC35C1-1:2	-1.55544	6.90573	-2.67354	0.040236	0.271753	-3.93385
MSTRG.31144.1	-1.48234	2.641271	-2.6734	0.040243	0.271766	-3.93404
NONHSAT209420.1	1.65587	3.113538	2.672668	0.04028	0.271871	-3.93499
ENST00000661000.1	-1.02002	2.722312	-2.6723	0.040299	0.271877	-3.93547
ENST00000531609.1	-1.15937	2.71686	-2.67227	0.0403	0.271877	-3.93551
NONHSAT218649.1	1.057976	1.963062	2.672251	0.040301	0.271877	-3.93554
MSTRG.53588.1	-1.02956	2.064242	-2.67223	0.040303	0.271877	-3.93557
ENST00000663935.1	1.274724	1.685949	2.672075	0.04031	0.271893	-3.93577
lnc-ART3-2:2	1.18971	3.415682	2.671497	0.04034	0.271962	-3.93653
NONHSAT179473.1	1.382529	2.852457	2.671414	0.040344	0.271962	-3.93664
NONHSAT213855.1	1.733471	2.136162	2.670176	0.040407	0.27222	-3.93826
NONHSAT196798.1	-1.20231	2.973018	-2.66894	0.040471	0.272466	-3.93988
NONHSAT205496.1	-1.24689	2.09023	-2.66873	0.040482	0.27252	-3.94016
NONHSAT179288.1	1.91676	3.144993	2.668658	0.040485	0.272526	-3.94025
ENST00000549251.1	1.376284	1.764943	2.667934	0.040522	0.272722	-3.9412
NONHSAT214082.1	1.014006	2.12833	2.667011	0.04057	0.272932	-3.94241
lnc-POFUT1-2:1	1.1279	1.990723	2.666707	0.040586	0.272996	-3.94281
lnc-SLC39A10-2:2	1.726436	2.897926	2.6655	0.040648	0.273127	-3.9444
NONHSAT186764.1	-1.3842	2.761524	-2.66544	0.040651	0.273131	-3.94448
NONHSAT203873.1	1.349643	2.235047	2.665145	0.040666	0.273188	-3.94486

ENST00000606194.1	1.490923	2.817569	2.664944	0.040676	0.273229	-3.94513
T226026	1.489422	2.237107	2.664486	0.0407	0.273326	-3.94573
lnc-PIP-1:1	-1.26403	2.301284	-2.66389	0.040731	0.273364	-3.94651
NONHSAT209663.1	1.149693	2.268671	2.663855	0.040733	0.273364	-3.94655
ENST00000662662.1	-1.18843	2.383025	-2.66289	0.040783	0.273559	-3.94782
NONHSAT169965.1	1.22649	1.680743	2.662646	0.040795	0.273607	-3.94814
MSTRG.57865.1	1.237496	2.077373	2.661745	0.040842	0.273724	-3.94932
lnc-ADAM18-2:2	1.101308	2.554707	2.661688	0.040845	0.273724	-3.9494
NONHSAT191694.1	1.038756	3.134024	2.66039	0.040912	0.273881	-3.9511
ENST00000602510.1	1.044516	4.392873	2.660203	0.040922	0.273892	-3.95135
NONHSAT161193.1	1.076924	4.815213	2.659184	0.040975	0.274049	-3.95269
lnc-PDK3-8:1	-1.23805	3.542563	-2.65913	0.040977	0.274049	-3.95275
ENST00000580326.1	1.284183	1.675428	2.659088	0.04098	0.274049	-3.95281
lnc-CXCL3-2:1	-1.18038	3.920653	-2.65867	0.041001	0.274052	-3.95336
lnc-USP20-1:1	-1.60779	3.09337	-2.65821	0.041026	0.27415	-3.95397
ENST00000606959.1	-1.19432	3.249049	-2.65743	0.041066	0.274286	-3.95499
lnc-BCL9L-2:1	1.131428	2.17782	2.657355	0.04107	0.274286	-3.95509
NONHSAT199680.1	1.335686	2.575095	2.657311	0.041072	0.274286	-3.95515
NONHSAT217982.1	-1.05585	7.00261	-2.6567	0.041104	0.274329	-3.95595
MSTRG.12436.2	-1.1823	2.400011	-2.65593	0.041144	0.274487	-3.95696
lnc-MTRNR2L13-11:1	-1.02464	2.154793	-2.65588	0.041147	0.274487	-3.95702
MSTRG.68534.1	1.306813	3.494746	2.655391	0.041172	0.27458	-3.95767
ENST00000572067.1	-1.3723	2.827606	-2.65509	0.041188	0.27458	-3.95806
lnc-KCTD15-2:3	-1.07748	7.445015	-2.65346	0.041274	0.274701	-3.96021
lnc-STX2-1:1	-1.07711	4.238033	-2.65274	0.041311	0.274767	-3.96115
MSTRG.35403.1	1.576276	2.20062	2.652546	0.041321	0.274797	-3.9614
NONHSAT189810.1	1.560831	2.665404	2.652032	0.041348	0.274832	-3.96208
NONHSAT156175.1	-1.0096	2.563106	-2.65172	0.041365	0.274891	-3.9625
NONHSAT177539.1	1.160751	2.053343	2.651233	0.04139	0.274911	-3.96313
lnc-RAB23-5:2	1.25655	2.707963	2.650676	0.04142	0.274998	-3.96386
lnc-ITGB8-4:1	1.543463	2.848419	2.649637	0.041474	0.275123	-3.96522
lnc-TUBB-17:1	1.200724	1.716578	2.649613	0.041476	0.275123	-3.96526
NONHSAT221027.1	-1.09611	2.128142	-2.64886	0.041515	0.275234	-3.96625
NONHSAT200153.1	-1.05658	2.190258	-2.6486	0.041529	0.275256	-3.96658
LINC00637:3	1.687081	3.408378	2.648538	0.041532	0.275256	-3.96667
NONHSAT171066.1	1.040229	1.657426	2.648236	0.041548	0.275294	-3.96707
ENST00000659612.1	-1.06225	1.638099	-2.64817	0.041551	0.275294	-3.96715
ENST00000653190.1	-1.02447	4.04058	-2.648	0.041561	0.275329	-3.96738
MSTRG.71226.1	1.489481	2.582426	2.647801	0.041571	0.275329	-3.96764
ENST00000420225.1	1.188602	2.522729	2.647726	0.041575	0.275329	-3.96774
MSTRG.20119.1	1.426843	1.80023	2.6476	0.041582	0.275329	-3.9679
NONHSAT201658.1	1.113828	2.479071	2.647582	0.041583	0.275329	-3.96793
NONHSAT187998.1	-1.03015	2.626197	-2.64723	0.041602	0.275371	-3.96839
lnc-MIA3-1:1	1.484625	3.049243	2.646841	0.041622	0.275433	-3.9689
NONHSAT161343.1	-1.15389	2.893402	-2.64585	0.041674	0.275617	-3.9702
ENST00000550506.2	1.334234	1.71659	2.645469	0.041694	0.275733	-3.9707
NONHSAT153049.1	-1.17054	2.062006	-2.64488	0.041726	0.275822	-3.97148
ENST00000513899.1	1.17406	2.271794	2.644721	0.041734	0.275822	-3.97168

lnc-SCGB2B2-10:2	1.038457	4.543396	2.643815	0.041782	0.276041	-3.97288
NR_125941	1.464851	2.616574	2.6437	0.041788	0.276042	-3.97303
NONHSAT158260.1	1.077627	2.015927	2.64345	0.041802	0.276042	-3.97335
lnc-MSX2-9:1	-1.33346	2.672395	-2.6431	0.04182	0.276111	-3.97381
lnc-KCNS2-5:3	-1.37567	4.053162	-2.64287	0.041833	0.276143	-3.97412
ENST00000579673.1	1.296172	2.772118	2.642607	0.041846	0.276143	-3.97446
NONHSAT167251.1	-1.04149	5.188887	-2.64255	0.04185	0.276143	-3.97454
lnc-PLAGL2-6:1	-1.09104	5.478688	-2.64176	0.041891	0.276256	-3.97557
NONHSAT220255.1	1.574896	2.910787	2.641673	0.041896	0.276266	-3.97569
ENST00000416842.1	-1.01676	2.792589	-2.64153	0.041904	0.276266	-3.97587
NONHSAT218822.1	1.292394	2.754222	2.640826	0.041941	0.276314	-3.9768
lnc-RNMT-2:2	1.212943	2.141573	2.640446	0.041962	0.276352	-3.9773
NONHSAT182881.1	-1.19804	1.897676	-2.6399	0.041991	0.276352	-3.97803
NONHSAT154407.1	1.164964	6.474619	2.639569	0.042008	0.27643	-3.97846
MSTRG.66984.1	1.106956	1.624223	2.638516	0.042065	0.276674	-3.97984
NONHSAT156173.1	1.275861	2.405395	2.637533	0.042117	0.276912	-3.98113
lnc-EAF1-3:1	1.049604	3.464729	2.637326	0.042128	0.276934	-3.9814
MSTRG.46435.2	1.107778	2.467128	2.63721	0.042135	0.276935	-3.98156
NONHSAT182887.1	1.188183	1.693053	2.637161	0.042137	0.276935	-3.98162
NONHSAT185920.1	1.105917	1.951092	2.634996	0.042253	0.277533	-3.98447
ENST00000554096.2	1.58719	2.82089	2.634952	0.042256	0.277533	-3.98453
MSTRG.53213.2	1.070946	2.203292	2.634348	0.042288	0.277566	-3.98532
ENST00000660197.1	1.236716	2.170229	2.634288	0.042291	0.277569	-3.9854
T070526	1.137068	4.696781	2.63381	0.042317	0.277631	-3.98603
lnc-PSD2-2:2	-1.00595	2.824426	-2.63381	0.042317	0.277631	-3.98603
lnc-HOXC13-1:2	-1.07611	2.944896	-2.63375	0.042321	0.277634	-3.98611
NONHSAT211225.1	1.253011	2.364388	2.633683	0.042324	0.277638	-3.98619
NONHSAT193294.1	-1.14465	2.375554	-2.63161	0.042436	0.27806	-3.98892
NONHSAT179024.1	1.117174	2.216621	2.631417	0.042446	0.27806	-3.98918
NONHSAT212092.1	1.054447	4.365404	2.631159	0.04246	0.278097	-3.98951
lnc-PRKACG-1:1	1.231966	2.768842	2.630723	0.042484	0.278124	-3.99009
T053049	1.25346	2.933988	2.629766	0.042535	0.278247	-3.99135
NONHSAT172339.1	-1.12725	2.159146	-2.62969	0.04254	0.278255	-3.99145
lnc-PRPS1-4:2	1.316453	2.866283	2.628259	0.042617	0.278492	-3.99333
MSTRG.65661.1	1.15294	2.469642	2.627535	0.042656	0.278622	-3.99428
lnc-ARHGAP21-4:1	1.061759	1.93495	2.626237	0.042727	0.27892	-3.99599
NONHSAT164902.1	-1.30459	2.104956	-2.6259	0.042745	0.278964	-3.99643
lnc-MAP2K4-3:4	1.290155	2.631395	2.625663	0.042758	0.278987	-3.99674
NONHSAT178466.1	-1.07714	2.321756	-2.62564	0.042759	0.278987	-3.99677
ENST00000423499.3	-1.15902	3.13482	-2.62553	0.042766	0.279005	-3.99692
NONHSAT167675.1	1.162873	4.297949	2.625297	0.042778	0.279005	-3.99723
ENST00000454135.1	1.298885	4.167173	2.624836	0.042803	0.279022	-3.99783
ENST00000652155.1	-1.0099	3.862902	-2.62472	0.04281	0.279022	-3.99799
NONHSAT211096.1	1.227131	2.881165	2.624694	0.042811	0.279022	-3.99802
NONHSAT199981.1	1.062162	3.44707	2.623842	0.042857	0.279176	-3.99914
lnc-SLC10A7-3:1	-1.32026	2.989814	-2.62251	0.04293	0.27949	-4.00089
NONHSAT174685.1	-1.34085	3.139881	-2.62237	0.042938	0.279521	-4.00108
NONHSAT153462.1	-1.29312	2.880457	-2.6214	0.042991	0.279723	-4.00235

MSTRG.39277.5	-1.1946	4.16282	-2.62102	0.043012	0.279764	-4.00285
lnc-TICRR-4:1	-1.02565	3.453985	-2.62014	0.04306	0.27995	-4.00401
MSTRG.24402.1	-1.24272	2.156721	-2.62	0.043068	0.27995	-4.00419
MSTRG.2824.1	1.005833	1.553416	2.619806	0.043078	0.28	-4.00445
lnc-CCT6B-2:1	-1.10169	3.215536	-2.61955	0.043093	0.280051	-4.00479
lnc-API5-10:1	2.071117	2.19688	2.618782	0.043135	0.280149	-4.0058
NONHSAT173378.1	-1.08634	1.903572	-2.61799	0.043178	0.280202	-4.00684
lnc-FBXO15-6:3	-1.54856	2.990175	-2.61754	0.043203	0.280234	-4.00743
lnc-PIGF-2:1	-1.15367	2.200449	-2.61682	0.043243	0.28032	-4.00839
NONHSAT156806.1	1.27802	2.568011	2.616547	0.043258	0.28032	-4.00874
NONHSAT171149.1	1.115969	2.494241	2.616159	0.043279	0.280366	-4.00925
lnc-CEP19-1:2	-1.15199	4.125503	-2.6148	0.043354	0.28059	-4.01104
NONHSAT200067.1	1.438038	1.796361	2.614141	0.043391	0.28069	-4.01191
NONHSAT151410.1	1.546547	3.130674	2.61392	0.043403	0.280751	-4.0122
MSTRG.38875.1	1.021104	4.385829	2.613755	0.043412	0.28078	-4.01242
lnc-TMEM177-3:3	1.049919	4.080402	2.613738	0.043413	0.28078	-4.01244
NONHSAT173237.1	1.035127	1.647281	2.61309	0.043449	0.28094	-4.01329
NONHSAT214113.1	1.001888	3.456827	2.612815	0.043464	0.281003	-4.01366
lnc-KIAA0319L-3:1	1.477429	2.54871	2.612441	0.043485	0.281073	-4.01415
ENST00000670548.1	1.318986	3.593724	2.611801	0.04352	0.281204	-4.01499
NONHSAT186445.1	-1.0239	2.198302	-2.61046	0.043594	0.281539	-4.01675
lnc-GLDC-8:2	1.026739	2.251352	2.609923	0.043625	0.281656	-4.01746
lnc-RCAN2-2:1	-1.19027	2.526934	-2.60982	0.04363	0.281656	-4.0176
NONHSAT161146.1	2.157714	3.99352	2.609616	0.043642	0.281656	-4.01787
NONHSAT181685.1	-1.37187	3.449442	-2.60954	0.043646	0.281656	-4.01797
lnc-LRCH2-3:1	1.001061	3.820461	2.608786	0.043688	0.281792	-4.01896
T081420	-1.05774	2.153619	-2.6087	0.043693	0.281792	-4.01908
lnc-NUB1-5:1	-1.13219	3.41387	-2.60834	0.043713	0.281814	-4.01954
NONHSAT154528.1	-1.11562	2.496945	-2.60705	0.043785	0.281901	-4.02125
lnc-WTAP-4:1	1.407911	2.335003	2.606731	0.043803	0.281928	-4.02167
NONHSAT217711.1	-1.55508	2.551564	-2.60523	0.043886	0.282083	-4.02364
NONHSAT177997.1	1.019447	2.631127	2.605072	0.043895	0.282083	-4.02385
NONHSAT221234.1	1.822991	2.645808	2.604941	0.043903	0.282083	-4.02403
NONHSAT190296.1	1.564404	3.643875	2.603868	0.043963	0.282291	-4.02544
NONHSAT180779.1	1.328264	1.741236	2.603731	0.04397	0.28232	-4.02562
NONHSAT206003.1	1.556771	2.47617	2.603577	0.043979	0.28232	-4.02582
NONHSAT205599.1	1.230458	3.41299	2.60291	0.044017	0.282412	-4.0267
MSTRG.45732.1	1.536414	2.305648	2.601404	0.044101	0.282712	-4.02869
ENST00000439928.2	-1.58823	3.14073	-2.60098	0.044125	0.282829	-4.02924
NONHSAT190171.1	-1.05873	3.705127	-2.60026	0.044166	0.282981	-4.03019
MSTRG.38905.1	1.126547	2.576624	2.599664	0.044199	0.283088	-4.03098
NONHSAT194118.1	1.237582	2.165607	2.599443	0.044212	0.283114	-4.03127
lnc-C5orf63-2:1	1.068957	2.060981	2.599094	0.044232	0.283123	-4.03173
NONHSAT154801.1	1.191772	1.75087	2.598636	0.044257	0.283173	-4.03233
lnc-PEPD-6:1	-1.07126	6.676965	-2.59786	0.044301	0.283232	-4.03335
NONHSAT158628.1	-1.01828	5.306172	-2.5978	0.044305	0.283232	-4.03344
NONHSAT150723.1	1.332053	2.625828	2.597776	0.044306	0.283232	-4.03347
NONHSAT196540.1	-1.07896	3.629761	-2.5977	0.04431	0.283232	-4.03356

MSTRG.26588.1	-1.23401	3.860595	-2.59702	0.044349	0.283368	-4.03447
ENST00000671430.1	-1.08458	2.429648	-2.597	0.04435	0.283368	-4.03448
MSTRG.32588.5	1.468988	2.64466	2.595961	0.044409	0.283441	-4.03586
lnc-SOD3-11:1	1.114333	2.054186	2.595906	0.044412	0.283441	-4.03593
NONHSAT219786.1	1.068817	3.461414	2.59586	0.044415	0.283441	-4.03599
MSTRG.33022.1	1.159184	4.204815	2.595445	0.044438	0.283483	-4.03654
NONHSAT154441.1	-1.29392	3.282577	-2.59493	0.044468	0.283564	-4.03722
NONHSAT189680.1	-1.37635	3.455634	-2.59324	0.044563	0.28389	-4.03944
lnc-MANEA-5:2	1.434802	2.319241	2.593181	0.044567	0.28389	-4.03952
NONHSAT194176.1	1.038592	3.560579	2.592611	0.044599	0.28399	-4.04027
NONHSAT217900.1	-1.68732	3.431533	-2.59251	0.044605	0.283992	-4.04041
ENST00000454735.1	-1.37931	5.675957	-2.59243	0.04461	0.284003	-4.04052
NONHSAT203336.1	-1.4358	2.546849	-2.59126	0.044676	0.284272	-4.04205
lnc-C12orf74-5:5	1.444993	2.596108	2.591113	0.044685	0.284272	-4.04225
MSTRG.51756.1	1.473243	2.661726	2.590385	0.044726	0.284304	-4.04321
MSTRG.4614.16	1.202428	3.974792	2.590346	0.044729	0.284304	-4.04326
lnc-ARL4C-11:1	1.447744	2.924306	2.58946	0.044779	0.284398	-4.04443
lnc-TMEM123-2:1	1.018388	1.596696	2.589259	0.044791	0.284398	-4.04469
lnc-RPAP1-1:5	1.464719	2.97077	2.589221	0.044793	0.284398	-4.04474
NONHSAT191604.1	1.070046	1.804411	2.589022	0.044804	0.284416	-4.04501
ENST00000521245.2	1.094159	2.554436	2.588741	0.04482	0.284446	-4.04538
lnc-HECTD2-1:1	-1.69753	3.263433	-2.58748	0.044892	0.284581	-4.04703
NONHSAT157898.1	1.51728	2.917563	2.587054	0.044917	0.284632	-4.0476
NONHSAT206627.1	1.099126	1.729065	2.587028	0.044919	0.284632	-4.04764
ENST00000657385.1	1.686718	4.272502	2.5869	0.044926	0.284632	-4.0478
lnc-ZNF469-3:1	1.325595	3.838501	2.58571	0.044994	0.284967	-4.04937
NONHSAT163322.1	1.342289	2.006974	2.584443	0.045067	0.285205	-4.05105
MSTRG.50166.3	1.536699	2.969233	2.583882	0.0451	0.285317	-4.05178
MSTRG.11972.1	1.532665	2.312951	2.58353	0.04512	0.285359	-4.05225
T230105	1.059782	1.573305	2.581132	0.045259	0.285658	-4.05541
MSTRG.43200.1	1.069258	1.746351	2.581096	0.045261	0.285658	-4.05546
NONHSAT174554.1	-1.03204	3.054439	-2.57828	0.045424	0.286204	-4.05917
lnc-C5orf46-1:1	-1.2645	2.699425	-2.57782	0.04545	0.286247	-4.05978
ENST00000615088.1	-1.00015	2.451268	-2.5761	0.045551	0.286469	-4.06206
lnc-ANO3-5:1	1.801013	2.780386	2.575429	0.04559	0.286588	-4.06294
NONHSAT222036.1	-1.20863	2.350355	-2.57522	0.045602	0.286614	-4.06322
T056769	-1.40072	2.995793	-2.57462	0.045637	0.286743	-4.06401
NONHSAT165415.1	1.302181	4.278623	2.574474	0.045646	0.286744	-4.0642
NONHSAT148744.1	1.800023	2.79405	2.573515	0.045702	0.286923	-4.06546
MSTRG.61503.1	-1.51388	2.738522	-2.5735	0.045703	0.286923	-4.06549
NONHSAT172294.1	1.383498	1.792052	2.573203	0.04572	0.286941	-4.06587
NONHSAT208255.1	-1.12314	2.456044	-2.57319	0.045721	0.286941	-4.06589
lnc-CLRN2-5:2	1.27968	3.107576	2.573144	0.045723	0.286941	-4.06595
ENST00000561800.1	-1.58119	2.605015	-2.57276	0.045746	0.286997	-4.06646
NONHSAT215341.1	1.262617	1.919081	2.572462	0.045763	0.287053	-4.06685
NONHSAT162470.1	1.275813	2.625749	2.572073	0.045786	0.287128	-4.06737
NONHSAT154620.1	1.261624	3.349342	2.572062	0.045787	0.287128	-4.06738
NONHSAT161335.1	-1.09218	2.08313	-2.57123	0.045836	0.287339	-4.06848

lnc-ZDHHC17-11:1	1.450357	1.834248	2.570201	0.045896	0.287584	-4.06984
T113305	-1.11551	4.213103	-2.56941	0.045942	0.287707	-4.07088
NONHSAT204247.1	1.306988	3.068375	2.569373	0.045945	0.287707	-4.07093
NONHSAT167068.1	-1.12969	3.010932	-2.56833	0.046006	0.287888	-4.0723
NONHSAT210794.1	-1.57323	2.338139	-2.56818	0.046015	0.287888	-4.0725
NONHSAT203953.1	1.244317	1.650404	2.568155	0.046016	0.287888	-4.07254
ENST00000653696.1	1.060905	4.419595	2.566688	0.046103	0.288196	-4.07447
NR_033977	1.206539	2.567611	2.56593	0.046148	0.288405	-4.07548
NONHSAT206367.1	1.029863	5.291648	2.565486	0.046174	0.288469	-4.07606
lnc-RPS6KA2-2:1	1.0998	2.11699	2.565257	0.046188	0.288493	-4.07636
lnc-FOXF2-4:1	-1.37897	3.399802	-2.56452	0.046231	0.28864	-4.07734
ENST00000554737.2	-1.21936	2.416388	-2.56422	0.046249	0.288698	-4.07773
lnc-ATP6AP2-11:1	1.446185	1.80688	2.563755	0.046277	0.28878	-4.07835
lnc-KDM4B-1:1	1.790053	3.230453	2.563333	0.046302	0.288856	-4.0789
NR_110201	1.358662	2.918756	2.562942	0.046325	0.288951	-4.07942
NONHSAT165224.1	-1.14591	2.31608	-2.5624	0.046357	0.289069	-4.08014
lnc-DENND1B-6:2	1.367654	2.850624	2.561872	0.046388	0.289156	-4.08083
NONHSAT164372.1	-1.28246	2.20803	-2.5615	0.04641	0.289194	-4.08132
MSTRG.51542.1	1.583785	2.47035	2.561142	0.046432	0.289194	-4.0818
lnc-ECSCR-3:1	-1.28693	2.570178	-2.5608	0.046452	0.289257	-4.08224
MIR4527HG:18	1.450378	2.912625	2.560528	0.046468	0.289278	-4.08261
ENST00000301683.7	-1.06186	4.115471	-2.5589	0.046565	0.289562	-4.08476
lnc-CCT5-28:1	1.837696	2.208138	2.558156	0.04661	0.289639	-4.08574
ENST00000653016.1	1.640136	2.571323	2.557852	0.046628	0.289648	-4.08614
NR_125864	1.303347	2.907951	2.557645	0.04664	0.289706	-4.08642
NONHSAT219739.1	1.395389	2.612376	2.557546	0.046646	0.289724	-4.08655
lnc-NOL11-4:2	1.928817	2.372539	2.556521	0.046708	0.289802	-4.0879
lnc-RTP5-3:2	1.550284	3.208105	2.554356	0.046837	0.290304	-4.09076
NONHSAT163641.1	-1.04693	1.881478	-2.55398	0.04686	0.290316	-4.09125
NONHSAT166893.1	1.129228	2.283082	2.553495	0.046889	0.290428	-4.0919
lnc-SNAP47-1:1	-1.14697	6.340727	-2.55303	0.046917	0.290483	-4.09251
NONHSAT152050.1	1.5843	1.872412	2.552853	0.046928	0.290507	-4.09275
lnc-EXT1-5:1	-1.0469	2.382766	-2.55234	0.046959	0.290553	-4.09343
lnc-CDC42EP3-2:1	1.69868	2.888088	2.552071	0.046975	0.290553	-4.09378
NONHSAT156616.1	-1.16723	2.160343	-2.55201	0.046978	0.290553	-4.09386
MSTRG.24872.1	-1.15108	2.773648	-2.55169	0.046998	0.290553	-4.09429
NONHSAT210357.1	1.109004	3.964496	2.551652	0.047	0.290553	-4.09433
lnc-BZW1-3:1	1.299933	2.580319	2.55152	0.047008	0.290553	-4.09451
ENST00000584078.1	-1.1296	2.245038	-2.55137	0.047017	0.290553	-4.09471
NR_103827	1.081043	5.581633	2.551132	0.047031	0.29056	-4.09502
MSTRG.46666.1	1.898249	2.695077	2.551084	0.047034	0.29056	-4.09508
NONHSAT204827.1	1.127735	3.413638	2.550621	0.047062	0.290679	-4.09569
NONHSAT197162.1	-1.08233	1.742727	-2.55033	0.04708	0.290741	-4.09608
NONHSAT177691.1	1.5015	1.805101	2.549811	0.047111	0.290821	-4.09677
NONHSAT153829.1	1.450163	2.475599	2.549707	0.047118	0.290837	-4.0969
MSTRG.26270.2	1.615197	2.989577	2.549626	0.047122	0.290837	-4.09701
NONHSAT182289.1	-1.09722	1.702712	-2.54929	0.047143	0.290928	-4.09746
NONHSAT219906.1	1.180096	2.660372	2.549098	0.047154	0.290959	-4.09771

NONHSAT193405.1	1.327967	3.098535	2.548424	0.047195	0.29108	-4.0986
ENST00000609583.1	1.261589	2.071678	2.548313	0.047202	0.29108	-4.09874
lnc-PACRGL-10:1	1.43156	2.518445	2.547976	0.047222	0.291159	-4.09919
NONHSAT165148.1	1.003115	3.708496	2.547905	0.047227	0.291159	-4.09928
ENST00000523881.1	-1.2228	6.007009	-2.54782	0.047232	0.291174	-4.0994
lnc-BMPER-4:1	1.101099	2.414015	2.546179	0.047331	0.291502	-4.10157
NONHSAT204962.1	-1.53009	3.53487	-2.5461	0.047336	0.291504	-4.10167
lnc-RSPH14-7:1	-1.06632	1.931701	-2.54538	0.04738	0.291653	-4.10263
NONHSAT161144.1	1.615845	2.589491	2.545342	0.047382	0.291653	-4.10267
T034024	-1.0357	2.30332	-2.54443	0.047438	0.291793	-4.10387
lnc-TECRL-12:1	-1.31342	4.680054	-2.54348	0.047495	0.291995	-4.10513
NONHSAT193018.1	-1.00262	4.299152	-2.54257	0.047551	0.29222	-4.10633
MSTRG.19174.1	1.351506	2.939481	2.541737	0.047602	0.292439	-4.10744
NONHSAT167414.1	-1.4743	3.165817	-2.54173	0.047602	0.292439	-4.10745
NONHSAT153966.1	1.220336	2.660393	2.541063	0.047643	0.292582	-4.10833
NONHSAT181993.1	1.537635	2.936283	2.539192	0.047758	0.292937	-4.1108
MSTRG.61389.2	1.351624	3.787082	2.538526	0.047799	0.293079	-4.11168
NONHSAT197496.1	1.230202	2.127049	2.538397	0.047807	0.293079	-4.11185
lnc-COX19-1:1	1.072284	2.744998	2.53801	0.04783	0.29312	-4.11236
NONHSAT223967.1	1.327654	5.396898	2.537806	0.047843	0.293148	-4.11263
MSTRG.35288.1	-1.05871	3.262377	-2.53772	0.047848	0.293164	-4.11275
NONHSAT206905.1	1.041275	2.457623	2.536929	0.047897	0.293267	-4.11379
MSTRG.69589.1	1.199631	2.576477	2.536534	0.047921	0.293281	-4.11431
NONHSAT199196.1	-1.2529	2.053638	-2.53643	0.047927	0.293281	-4.11445
NONHSAT224494.1	1.22547	2.115785	2.536398	0.047929	0.293281	-4.11449
NONHSAT193028.1	1.282535	2.048561	2.535727	0.047971	0.293412	-4.11538
lnc-CCKAR-1:13	-1.01968	4.379405	-2.53433	0.048057	0.293588	-4.11723
MSTRG.29057.151	1.145716	4.743882	2.533244	0.048124	0.293729	-4.11867
lnc-POU6F2-1:1	1.318109	2.951417	2.532893	0.048146	0.293754	-4.11913
NONHSAT213668.1	-1.1356	2.577141	-2.53287	0.048147	0.293754	-4.11916
NONHSAT173171.1	2.576782	3.798042	2.532724	0.048156	0.293754	-4.11935
ENST00000671426.1	1.20374	2.425474	2.53269	0.048158	0.293754	-4.1194
MSTRG.30060.1	1.261982	2.163183	2.531828	0.048212	0.293912	-4.12054
NR_125422	-1.27202	2.374316	-2.53172	0.048218	0.293912	-4.12068
ENST00000427173.1	1.189051	2.331253	2.531159	0.048253	0.293947	-4.12142
NONHSAT158403.1	1.330865	7.665768	2.530902	0.048269	0.293974	-4.12176
MSTRG.56289.1	1.198023	1.671977	2.529631	0.048348	0.294238	-4.12344
NONHSAT210575.1	1.184258	2.386519	2.529554	0.048353	0.294238	-4.12355
NONHSAT164735.1	-1.46233	4.897545	-2.52955	0.048353	0.294238	-4.12355
NONHSAT194894.1	-1.09826	2.315921	-2.52914	0.048378	0.294301	-4.12409
MSTRG.34306.1	1.426302	2.680975	2.528837	0.048397	0.294346	-4.12449
NONHSAT193350.1	1.113338	2.012922	2.528224	0.048436	0.294507	-4.12531
CNTFR-AS1:14	1.324033	2.373969	2.527392	0.048487	0.294702	-4.12641
ENST00000622466.1	1.045136	3.859721	2.525779	0.048588	0.294998	-4.12854
NONHSAT198089.1	1.8887	2.47344	2.525463	0.048608	0.294998	-4.12896
lnc-CNOT1-2:1	-1.46727	2.898419	-2.52544	0.048609	0.294998	-4.12899
NONHSAT171467.1	1.260571	2.901358	2.525187	0.048625	0.295055	-4.12932
lnc-BBIP1-8:1	-1.02305	5.79999	-2.525	0.048637	0.295076	-4.12957

NONHSAT174094.1	1.505862	2.269757	2.523276	0.048745	0.29547	-4.13185
lnc-KCTD17-3:1	-1.08836	8.255036	-2.52256	0.04879	0.295529	-4.1328
NONHSAT198406.1	-1.30017	2.953658	-2.5221	0.048819	0.295543	-4.13341
ENST00000455498.1	1.116402	2.439289	2.521683	0.048845	0.29559	-4.13396
NONHSAT153635.1	-1.33149	4.802719	-2.52033	0.04893	0.295825	-4.13575
ENST00000514877.2	1.084666	1.744512	2.520311	0.048931	0.295825	-4.13577
NONHSAT167424.1	-1.15438	2.144759	-2.52016	0.048941	0.295843	-4.13598
MSTRG.20397.1	1.690188	1.902877	2.519843	0.048961	0.295845	-4.13639
NONHSAT164713.1	-1.25151	4.795266	-2.51969	0.04897	0.295862	-4.1366
lnc-GPR132-1:1	1.834406	2.689211	2.518204	0.049064	0.296113	-4.13856
NONHSAT201797.1	1.280656	2.578479	2.517958	0.049079	0.296136	-4.13889
NONHSAT210826.1	1.346935	1.706044	2.517162	0.04913	0.29628	-4.13994
MSTRG.47000.33	-1.04566	5.853752	-2.51703	0.049138	0.296313	-4.14012
lnc-EPB41L2-2:1	-1.23846	2.760836	-2.51662	0.049164	0.296396	-4.14065
T302058	1.112578	2.490976	2.516143	0.049194	0.296527	-4.14129
ENST00000412654.1	1.100493	4.016644	2.515754	0.049219	0.296534	-4.14181
lnc-SATB1-4:1	1.356305	3.756436	2.515706	0.049222	0.296534	-4.14187
ENST00000652589.1	-1.49271	7.087964	-2.51537	0.049243	0.296575	-4.14232
MSTRG.8945.1	1.719879	3.222437	2.515099	0.04926	0.296575	-4.14267
NONHSAT153716.1	1.627791	2.767142	2.514728	0.049284	0.296624	-4.14316
MSTRG.64535.1	-1.03703	2.53222	-2.51351	0.049361	0.296911	-4.14477
lnc-GPATCH1-1:1	1.146861	1.835167	2.513199	0.049381	0.296953	-4.14519
NONHSAT166070.1	-1.13359	5.600107	-2.51317	0.049383	0.296953	-4.14522
T314979	1.384394	2.339773	2.512951	0.049397	0.296971	-4.14552
lnc-CLIC5-2:1	-1.30508	3.00244	-2.51193	0.049461	0.297144	-4.14686
lnc-EIPR1-8:1	1.118087	2.64385	2.511923	0.049462	0.297144	-4.14688
ENST00000661978.1	-1.05551	1.941254	-2.51188	0.049465	0.297144	-4.14693
lnc-AKR1E2-16:1	1.966242	3.29493	2.511776	0.049472	0.297156	-4.14707
MSTRG.54854.17	1.120431	2.381218	2.511685	0.049477	0.29716	-4.14719
NONHSAT206427.1	1.149939	2.133856	2.511134	0.049512	0.29724	-4.14792
NONHSAT161187.1	1.625733	1.838518	2.511029	0.049519	0.29724	-4.14806
NONHSAT158640.1	-1.22027	2.430294	-2.51099	0.049522	0.29724	-4.14812
lnc-RASEF-2:1	1.213267	1.67855	2.510758	0.049536	0.297275	-4.14842
MSTRG.21016.1	-1.04869	3.702913	-2.51043	0.049557	0.29731	-4.14885
lnc-SLC25A29-2:1	-1.11541	1.673693	-2.50895	0.049652	0.297527	-4.15081
NR_026760	1.46379	2.311755	2.508947	0.049652	0.297527	-4.15082
MSTRG.8195.1	-1.2389	3.137478	-2.50868	0.049669	0.297576	-4.15117
NONHSAT154087.1	1.309509	2.162105	2.508532	0.049679	0.297597	-4.15137
lnc-SMIM33-1:1	-1.03329	2.275862	-2.50766	0.049734	0.297767	-4.15252
lnc-CTH-14:1	1.189557	2.395604	2.506935	0.049781	0.297855	-4.15348
NONHSAT173575.1	-1.00598	1.810289	-2.50651	0.049808	0.297914	-4.15405
lnc-SCTR-2:4	-1.3385	3.219368	-2.50572	0.049859	0.298078	-4.15509
NONHSAT205238.1	1.267357	2.657079	2.505711	0.04986	0.298078	-4.1551
lnc-KLF4-11:1	-1.11814	2.216664	-2.50559	0.049867	0.29809	-4.15526
NONHSAT201690.1	-1.10374	3.169325	-2.50529	0.049886	0.298098	-4.15565
MSTRG.65885.2	1.442445	2.830689	2.504889	0.049912	0.298109	-4.15619
lnc-PLET1-7:1	1.422137	5.142744	2.504353	0.049947	0.298193	-4.1569
NONHSAT206486.1	1.292436	2.950502	2.504071	0.049965	0.298201	-4.15727

MSTRG.62230.27	1.022482	3.550632	2.504	0.04997	0.298201	-4.15737
NONHSAT168621.1	-1.40573	2.835039	-2.50374	0.049986	0.298233	-4.15771

Table S4. significant different expression of lncRNA after glucose deprivation compared to

controls.

LncRNA	logFC	AveExpr	t	P.Value	adj.P.Val	B
lnc-TEX2-1:1	2.983421	2.554823	24.32173	7.56E-07	0.025848	5.434501
lnc-HTR3D-2:1	3.514912	6.306772	24.12824	7.90E-07	0.025848	5.415234
NONHSAT168904.1	2.443664	2.243122	21.8448	1.36E-06	0.025848	5.159426
lnc-THBS1-5:1	-3.04458	3.759083	-21.8027	1.38E-06	0.025848	5.154168
ENST00000564240.1	-2.83514	2.544331	-21.5022	1.49E-06	0.025848	5.115993
NONHSAT156824.1	2.275324	2.257939	20.30389	2.04E-06	0.025848	4.951989
NONHSAT196599.1	2.026559	2.109175	20.09438	2.16E-06	0.025848	4.921242
NONHSAT182696.1	2.675269	2.444138	19.37668	2.63E-06	0.025848	4.810808
NONHSAT168006.1	2.478441	2.380344	19.29177	2.69E-06	0.025848	4.797198
ENST00000657119.1	2.700027	2.410614	19.27067	2.71E-06	0.025848	4.793797
MSTRG.5623.1	2.042337	8.04608	19.13271	2.82E-06	0.025848	4.771377
lnc-GADD45A-4:1	2.048874	8.812479	18.09158	3.83E-06	0.028213	4.59135
NONHSAT214950.1	1.892876	1.999034	17.42857	4.70E-06	0.028213	4.465904
MSTRG.16199.1	2.122871	2.15423	17.41101	4.72E-06	0.028213	4.462459
lnc-CERK-17:1	2.58725	2.441959	17.20089	5.04E-06	0.028213	4.420717
lnc-TRIM27-18:1	2.382683	2.263464	17.12432	5.17E-06	0.028213	4.405267
NR_103827	2.180638	6.131431	16.74291	5.84E-06	0.028213	4.326355
MSTRG.55066.1	2.024208	2.102626	16.73339	5.86E-06	0.028213	4.324343
MSTRG.16713.1	1.713966	5.905734	16.51682	6.29E-06	0.028213	4.277995
NONHSAT221845.1	-1.75717	1.999598	-16.4361	6.46E-06	0.028213	4.260423
NONHSAT153565.1	2.400845	2.479404	16.34239	6.67E-06	0.028213	4.239848
NONHSAT218416.1	1.819418	2.035424	16.19926	7.00E-06	0.028213	4.20799
ENST00000601618.1	-1.74057	2.053329	-15.9788	7.54E-06	0.028213	4.157911
NONHSAT199539.1	2.209574	2.425781	15.9615	7.58E-06	0.028213	4.153932
ENST00000621389.1	2.904388	7.065098	15.89342	7.76E-06	0.028213	4.138187
lnc-MPLKIP-13:1	1.654209	3.561919	15.72276	8.23E-06	0.028213	4.098179
NONHSAT156259.1	2.457714	2.311418	15.62842	8.50E-06	0.028213	4.075733
NONHSAT197062.1	1.67482	1.927577	15.58865	8.62E-06	0.028213	4.066195
T345203	2.55442	6.181447	15.54243	8.76E-06	0.028213	4.055061
NONHSAT172926.1	1.79272	2.698218	15.41428	9.17E-06	0.028213	4.023878
NONHSAT212969.1	2.510794	2.280484	15.24094	9.75E-06	0.028213	3.98097
MSTRG.37340.2	-1.86706	2.109082	-15.0581	1.04E-05	0.028213	3.934788
MSTRG.59871.1	2.25393	2.298128	14.96224	1.08E-05	0.028213	3.910171
ENST00000579752.1	2.287983	2.228243	14.9618	1.08E-05	0.028213	3.910056
NONHSAT204602.1	1.971149	2.046468	14.94989	1.08E-05	0.028213	3.906981
lnc-ABCG5-3:1	2.174992	2.197504	14.80061	1.14E-05	0.028213	3.868059
NR_004383	-1.90709	9.613983	-14.7813	1.15E-05	0.028213	3.862986
ENST00000423942.1	2.122581	2.23806	14.76373	1.16E-05	0.028213	3.85834
lnc-NUDT2-5:1	2.06774	2.114217	14.69548	1.19E-05	0.028213	3.840243
ENST00000520588.1	-1.88823	3.881836	-14.4728	1.29E-05	0.028213	3.7802
MSTRG.19613.1	-2.35613	2.315169	-14.4403	1.31E-05	0.028213	3.771305
NR_029487	-1.66568	1.912265	-14.4179	1.32E-05	0.028213	3.765142
NONHSAT205228.1	2.152009	7.159414	14.39563	1.33E-05	0.028213	3.759014
MSTRG.61229.1	-1.72394	3.609143	-14.3882	1.33E-05	0.028213	3.756963
lnc-MRPL33-2:1	-2.01513	3.041181	-14.3572	1.35E-05	0.028213	3.748394
ENST00000670395.1	2.001442	2.102346	14.34036	1.36E-05	0.028213	3.743728

lnc-ZC3H12D-2:2	1.71846	1.974844	14.32013	1.37E-05	0.028213	3.738108
lnc-PHF24-1:1	1.548532	1.887667	14.30073	1.38E-05	0.028213	3.732707
NONHSAT156871.1	2.284337	2.436341	14.26652	1.39E-05	0.028213	3.723153
MSTRG.48086.1	1.986315	2.040705	14.17845	1.44E-05	0.028213	3.698376
NONHSAT167997.1	2.238702	2.501092	14.1275	1.47E-05	0.028213	3.683926
NONHSAT208879.1	2.258051	2.190899	14.1214	1.47E-05	0.028213	3.682191
lnc-ACAD11-2:1	1.8249	2.001203	14.05676	1.51E-05	0.028213	3.663718
NR_125940	1.674915	1.851487	13.99587	1.55E-05	0.028213	3.646189
lnc-NDUFB4-5:1	-2.22519	2.35712	-13.8864	1.61E-05	0.028213	3.614361
MSTRG.52989.1	1.850701	4.937992	13.79138	1.68E-05	0.028213	3.586387
NONHSAT156002.1	-1.45553	6.078459	-13.7811	1.68E-05	0.028213	3.583347
NONHSAT217334.1	1.64067	1.915789	13.74976	1.70E-05	0.028213	3.574035
NONHSAT177733.1	2.232657	3.121165	13.72792	1.72E-05	0.028213	3.567529
LINC01020:9	1.44747	1.774878	13.69132	1.74E-05	0.028213	3.556589
MSTRG.16372.1	1.989457	2.140678	13.66575	1.76E-05	0.028213	3.548919
NONHSAT195823.1	2.204197	2.503484	13.66229	1.76E-05	0.028213	3.547879
NONHSAT156470.1	-1.71621	4.161339	-13.6398	1.78E-05	0.028213	3.541098
lnc-DUSP14-1:1	1.772244	6.001942	13.62282	1.79E-05	0.028213	3.535986
NONHSAT199836.1	1.392515	1.756537	13.45237	1.92E-05	0.029734	3.483978
MSTRG.19670.1	1.722097	1.901365	13.36524	1.99E-05	0.030032	3.456982
ENST00000577171.1	1.416777	6.218748	13.33739	2.01E-05	0.030032	3.448293
NONHSAT164535.1	1.426866	1.797382	13.2929	2.04E-05	0.030032	3.434349
lnc-BEGAIN-3:1	2.308653	2.171961	13.22185	2.10E-05	0.030032	3.41193
NONHSAT170348.1	1.524814	5.580842	13.17459	2.15E-05	0.030032	3.396909
NONHSAT158403.1	1.608475	7.804573	13.106	2.21E-05	0.030032	3.374957
MSTRG.26960.1	1.521047	1.859795	13.09039	2.22E-05	0.030032	3.369935
NONHSAT221322.1	-2.03094	2.356043	-13.0561	2.25E-05	0.030032	3.358878
NONHSAT206540.1	2.517469	2.854961	13.05576	2.25E-05	0.030032	3.35876
NR_028328	-2.06206	7.218157	-13.0291	2.28E-05	0.030032	3.35013
NONHSAT171768.1	2.774117	2.41287	12.96225	2.34E-05	0.030032	3.328352
lnc-STMN2-2:1	2.442787	2.382045	12.95201	2.35E-05	0.030032	3.325002
lnc-EMB-10:1	-2.27835	2.494412	-12.9411	2.36E-05	0.030032	3.321427
NONHSAT190586.1	2.308184	7.648295	12.93892	2.36E-05	0.030032	3.32071
lnc-PCDH19-7:1	1.827667	2.052441	12.89216	2.41E-05	0.030032	3.305332
lnc-SPTBN4-3:3	1.76667	1.907046	12.80083	2.51E-05	0.030032	3.27504
ENST00000655097.1	1.911369	2.06301	12.78491	2.52E-05	0.030032	3.269725
NR_004384	-2.03591	12.93042	-12.7079	2.61E-05	0.030032	3.24386
NONHSAT210632.1	1.615607	1.894463	12.69485	2.62E-05	0.030032	3.239461
NONHSAT207426.1	1.883442	2.040152	12.68937	2.63E-05	0.030032	3.237607
lnc-FILIP1L-10:1	-2.42335	2.728802	-12.687	2.63E-05	0.030032	3.2368
lnc-TRIM32-8:1	1.53137	1.814022	12.66003	2.66E-05	0.030032	3.227669
ENST00000444629.5	1.806241	1.981245	12.62872	2.69E-05	0.030032	3.217023
NONHSAT179896.1	1.625338	1.843193	12.58828	2.74E-05	0.030032	3.203214
NONHSAT192231.1	1.783232	1.945052	12.58223	2.75E-05	0.030032	3.20114
ENST00000453806.1	-1.73951	4.981738	-12.5822	2.75E-05	0.030032	3.201139
MSTRG.37964.1	1.961934	5.003519	12.57875	2.75E-05	0.030032	3.19995
MSTRG.22299.1	1.59627	6.51064	12.56439	2.77E-05	0.030032	3.195023
NONHSAT161378.1	-1.31707	6.620257	-12.4996	2.85E-05	0.030345	3.172696

lnc-CENPK-4:1	-2.36988	2.399202	-12.4355	2.93E-05	0.030345	3.150399
ENST00000659906.1	1.643062	2.888929	12.40863	2.96E-05	0.030345	3.141024
MSTRG.37919.1	-1.72785	2.415477	-12.3835	2.99E-05	0.030345	3.132233
MSTRG.17580.1	-1.60629	4.013538	-12.3646	3.02E-05	0.030345	3.125559
MSTRG.5986.15	-1.95553	2.322986	-12.3645	3.02E-05	0.030345	3.125535
NR_024243	-1.78246	14.97312	-12.3561	3.03E-05	0.030345	3.122596
lnc-CD38-12:3	1.608031	6.12143	12.34926	3.04E-05	0.030345	3.120167
lnc-FGF18-4:2	1.916591	2.074234	12.3252	3.07E-05	0.030364	3.111673
lnc-LY96-2:6	1.8704	1.999404	12.28208	3.13E-05	0.030536	3.096384
MSTRG.50194.7	2.702237	2.557495	12.23519	3.19E-05	0.030536	3.079663
NONHSAT208155.1	1.515851	1.806768	12.22473	3.21E-05	0.030536	3.075919
ENST00000660183.1	1.293124	1.7076	12.22445	3.21E-05	0.030536	3.07582
lnc-SLC15A4-25:1	2.054019	2.132461	12.19223	3.26E-05	0.03054	3.064258
ENST00000645181.1	2.192491	2.168261	12.18164	3.27E-05	0.03054	3.060448
NONHSAT218663.1	-1.77481	2.893535	-12.1285	3.35E-05	0.03082	3.041236
ENST00000520543.1	2.599612	5.934996	12.10817	3.38E-05	0.03082	3.033878
T103396	1.622455	2.091051	12.09908	3.39E-05	0.03082	3.030573
lnc-CNDP1-5:1	-1.98891	3.608239	-12.0597	3.45E-05	0.030857	3.016212
lnc-TAS2R1-13:1	2.017526	2.109815	12.05622	3.46E-05	0.030857	3.014943
NONHSAT160698.1	1.566289	4.774624	12.01339	3.52E-05	0.030881	2.999237
NONHSAT176819.1	1.295689	4.784806	11.99345	3.56E-05	0.030881	2.991897
MSTRG.70356.1	-2.21797	2.218894	-11.9798	3.58E-05	0.030881	2.986877
lnc-NEURL1B-3:7	2.42793	7.564297	11.97554	3.58E-05	0.030881	2.985291
T357613	1.854258	2.26499	11.95389	3.62E-05	0.030881	2.977283
lnc-ALKBH8-1:1	1.676366	3.459973	11.93881	3.64E-05	0.030881	2.971693
MSTRG.12894.1	-2.47695	2.571708	-11.9009	3.71E-05	0.03115	2.957588
lnc-TP53TG3F-17:1	2.914872	2.473404	11.88185	3.74E-05	0.031159	2.950479
ENST00000614738.1	-1.67273	3.828404	-11.827	3.83E-05	0.031679	2.929922
lnc-UBASH3A-8:4	2.128789	2.123338	11.80907	3.86E-05	0.031679	2.923151
NONHSAT193259.1	1.959183	2.085765	11.7736	3.93E-05	0.03187	2.909745
ENST00000416909.1	-1.8832	2.238159	-11.7567	3.96E-05	0.03187	2.903335
lnc-CDH11-7:1	1.993597	7.199593	11.70839	4.05E-05	0.03187	2.884937
NONHSAT173296.1	1.971497	4.451678	11.70553	4.05E-05	0.03187	2.883841
NONHSAT186855.1	1.813458	3.526295	11.69763	4.07E-05	0.03187	2.880823
ENST00000592429.1	1.41948	6.263818	11.6741	4.11E-05	0.03187	2.871809
NONHSAT201540.1	1.845892	5.47181	11.65798	4.14E-05	0.03187	2.865616
T113219	2.081205	3.903822	11.65018	4.16E-05	0.03187	2.862616
NONHSAT197178.1	2.401851	2.298012	11.62606	4.20E-05	0.03187	2.853319
NONHSAT163750.1	1.241502	1.746776	11.55921	4.33E-05	0.03187	2.82741
NONHSAT217700.1	2.471741	6.15867	11.55836	4.34E-05	0.03187	2.827076
MSTRG.25860.1	1.930347	6.645562	11.5216	4.41E-05	0.03187	2.812732
lnc-MFSD8-9:1	2.608096	2.598704	11.50427	4.45E-05	0.03187	2.805945
lnc-CREB1-5:1	1.503712	1.834215	11.5009	4.45E-05	0.03187	2.804624
lnc-ANKDD1B-1:1	-1.45461	2.106355	-11.4739	4.51E-05	0.03187	2.794032
NONHSAT163617.1	-1.37336	8.373008	-11.469	4.52E-05	0.03187	2.792093
lnc-ZBBX-3:1	2.275267	2.173925	11.46642	4.52E-05	0.03187	2.791072
NONHSAT212535.1	1.99983	2.061519	11.45021	4.56E-05	0.03187	2.784682
NONHSAT153599.1	1.285866	1.676755	11.44825	4.56E-05	0.03187	2.783906

NR_024244	-1.84125	14.08783	-11.4445	4.57E-05	0.03187	2.782419
MSTRG.47169.1	-1.67415	4.42932	-11.422	4.62E-05	0.03187	2.773546
NONHSAT221831.1	2.013748	2.881964	11.40359	4.66E-05	0.03187	2.766228
MSTRG.30163.1	1.560716	4.64761	11.38046	4.71E-05	0.03187	2.757029
NONHSAT196765.1	-1.35952	1.847575	-11.3769	4.72E-05	0.03187	2.755612
lnc-CBR1-1:3	1.775462	2.125551	11.37619	4.72E-05	0.03187	2.755328
lnc-NDUFB4-3:1	-2.15503	2.259663	-11.3574	4.76E-05	0.03187	2.747816
lnc-DDX60-2:1	-2.26439	2.291083	-11.3565	4.76E-05	0.03187	2.747483
MSTRG.11715.1	-1.84451	2.20979	-11.3525	4.77E-05	0.03187	2.745861
MSTRG.56903.1	-2.57518	2.500527	-11.2969	4.90E-05	0.032073	2.723569
ENST00000418282.2	2.745191	2.464881	11.29049	4.91E-05	0.032073	2.720993
NR_024230	-1.72068	15.41639	-11.2863	4.92E-05	0.032073	2.719301
NONHSAT181816.1	-1.64966	4.046395	-11.2837	4.93E-05	0.032073	2.718246
T187569	-1.65327	2.537995	-11.2638	4.98E-05	0.032125	2.710214
lnc-SERPINA12-2:1	1.191829	1.6652	11.25317	5.00E-05	0.032125	2.705923
NR_024214	-1.67369	15.63723	-11.1997	5.13E-05	0.032717	2.684192
ENST00000551372.1	1.449139	1.819269	11.17002	5.20E-05	0.032717	2.672089
ENST00000669434.1	1.27369	8.523354	11.16423	5.22E-05	0.032717	2.669718
lnc-EDAR-9:1	2.34901	2.230446	11.1621	5.22E-05	0.032717	2.668846
NONHSAT222524.1	2.434382	2.282588	11.13194	5.30E-05	0.032988	2.656475
lnc-COL8A1-3:2	-1.47897	7.351048	-11.0644	5.47E-05	0.033867	2.628602
NONHSAT219358.1	2.005663	5.95481	10.99239	5.67E-05	0.034854	2.598588
NONHSAT211005.1	1.242172	1.769483	10.95574	5.77E-05	0.035036	2.583211
lnc-SIPA1L2-8:1	1.611674	4.146078	10.95466	5.77E-05	0.035036	2.582754
ENST00000648601.1	1.879528	3.615328	10.93807	5.82E-05	0.035036	2.57577
lnc-PRKCD-3:5	-1.48326	3.044768	-10.9322	5.84E-05	0.035036	2.57328
MSTRG.48889.1	-2.21465	2.306191	-10.9179	5.88E-05	0.035072	2.567249
T349820	-1.69423	3.250475	-10.873	6.01E-05	0.035107	2.548217
ENST00000542076.1	1.410372	1.926554	10.87047	6.02E-05	0.035107	2.547153
NONHSAT202201.1	1.724329	6.539039	10.86683	6.03E-05	0.035107	2.545605
MSTRG.63591.1	-1.2052	4.147708	-10.8337	6.13E-05	0.035107	2.53149
NONHSAT182196.1	-1.98644	2.359883	-10.8227	6.16E-05	0.035107	2.52678
lnc-BAZ2B-1:7	-1.36126	4.374617	-10.8123	6.19E-05	0.035107	2.522327
MSTRG.53837.1	2.138697	2.494222	10.80693	6.21E-05	0.035107	2.520024
lnc-P2RY1-2:1	1.275788	2.506398	10.79532	6.24E-05	0.035107	2.515042
ENST00000664488.1	-1.49734	4.444411	-10.772	6.31E-05	0.035107	2.505037
T160707	3.571891	2.829317	10.77134	6.32E-05	0.035107	2.504733
NONHSAT202194.1	1.659218	6.322596	10.77102	6.32E-05	0.035107	2.504593
lnc-CD46-3:1	2.221579	2.238131	10.76582	6.33E-05	0.035107	2.502355
NONHSAT173171.1	4.20993	4.614616	10.75079	6.38E-05	0.035107	2.495867
T315546	1.470364	1.842186	10.72253	6.47E-05	0.035107	2.483644
NONHSAT165196.1	1.601659	1.854466	10.69487	6.56E-05	0.035107	2.471632
ENST00000519803.1	-1.74887	2.170074	-10.6917	6.57E-05	0.035107	2.470247
lnc-HTR1B-1:5	-1.30736	6.071777	-10.6811	6.61E-05	0.035107	2.465656
lnc-CCDC144NL-4:1	1.162458	11.89958	10.68081	6.61E-05	0.035107	2.465509
lnc-EEF2-3:1	1.425262	1.727819	10.67749	6.62E-05	0.035107	2.464064
lnc-SPATA6L-4:1	1.119662	2.414252	10.66336	6.67E-05	0.035107	2.457896
MSTRG.67348.1	1.525061	8.478411	10.66118	6.67E-05	0.035107	2.456944

T262691	-1.89555	2.22917	-10.651	6.71E-05	0.035107	2.452513
NONHSAT221960.1	1.321745	3.63003	10.62619	6.79E-05	0.035107	2.441622
lnc-CTNNA1-4:2	1.961328	6.941802	10.62268	6.80E-05	0.035107	2.44008
MSTRG.19441.1	-1.41867	3.341912	-10.6025	6.87E-05	0.035107	2.43119
NONHSAT155120.1	2.043752	6.39348	10.60156	6.87E-05	0.035107	2.430791
NONHSAT199491.1	1.900883	2.356964	10.59773	6.89E-05	0.035107	2.429105
lnc-HIST1H2AH-5:1	-1.62818	8.153641	-10.5977	6.89E-05	0.035107	2.429096
NONHSAT173365.1	1.556803	3.319931	10.59525	6.90E-05	0.035107	2.428013
MSTRG.58292.5	1.617663	1.828125	10.57899	6.95E-05	0.035107	2.420838
lnc-CNDP1-6:1	-1.2989	4.490371	-10.5759	6.96E-05	0.035107	2.41949
MSTRG.12643.4	-1.193	2.158738	-10.5603	7.02E-05	0.035208	2.412563
NONHSAT211083.1	1.894737	5.692833	10.54555	7.07E-05	0.035295	2.406041
NONHSAT172031.1	1.271532	5.325505	10.53167	7.12E-05	0.035368	2.399876
lnc-LSMEM1-2:1	2.944719	2.649474	10.5129	7.19E-05	0.03553	2.391526
NONHSAT198051.1	1.664121	1.936057	10.4979	7.24E-05	0.035626	2.384835
ENST00000609827.1	-1.8649	2.2096	-10.4749	7.33E-05	0.035771	2.374576
lnc-OR10K1-1:1	-1.37628	4.732928	-10.4627	7.37E-05	0.035771	2.369067
MSTRG.23637.1	1.831857	1.97281	10.46122	7.38E-05	0.035771	2.368426
NONHSAT158418.1	1.933411	2.166774	10.4441	7.44E-05	0.035866	2.360738
NONHSAT149668.1	1.708242	3.986556	10.42128	7.53E-05	0.035866	2.350463
lnc-MARCH4-2:10	-1.10867	5.806953	-10.4117	7.57E-05	0.035866	2.346155
NR_024342	-1.64693	14.21585	-10.3964	7.63E-05	0.035866	2.339216
MSTRG.8708.2	1.347104	9.196872	10.36755	7.74E-05	0.035866	2.326153
NR_002976	-1.46987	8.191472	-10.3571	7.78E-05	0.035866	2.321426
lnc-SOHLH2-3:1	-1.22982	8.309375	-10.3536	7.80E-05	0.035866	2.319793
NONHSAT183290.1	1.567611	5.428803	10.3458	7.83E-05	0.035866	2.316263
lnc-WRNIP1-34:2	1.108825	1.622461	10.31419	7.95E-05	0.035866	2.301838
NONHSAT149439.1	-1.0557	5.92883	-10.3138	7.96E-05	0.035866	2.301639
NONHSAT206047.1	-1.72676	3.298869	-10.313	7.96E-05	0.035866	2.301307
lnc-FRMD4A-6:1	-1.32452	11.27414	-10.3117	7.96E-05	0.035866	2.300719
MSTRG.34252.1	1.648324	1.888431	10.30276	8.00E-05	0.035866	2.296608
lnc-CDY2A-11:1	1.634703	5.527621	10.29692	8.03E-05	0.035866	2.293935
lnc-VRK2-14:1	1.646123	1.891569	10.29514	8.03E-05	0.035866	2.293119
MSTRG.24373.1	-1.26619	4.844265	-10.272	8.13E-05	0.035866	2.282506
lnc-NT5E-3:1	-1.73212	4.194145	-10.2718	8.13E-05	0.035866	2.282392
MSTRG.70999.1	-1.31851	6.785662	-10.2528	8.21E-05	0.035866	2.273651
ENST00000445418.1	1.867445	4.444669	10.23329	8.29E-05	0.035866	2.264648
lnc-TDRD1-1:1	-1.14586	9.175468	-10.2216	8.34E-05	0.035866	2.259244
lnc-RXFP3-2:1	-1.26424	7.597213	-10.2212	8.35E-05	0.035866	2.259062
ENST00000618303.1	2.201269	2.210011	10.21697	8.36E-05	0.035866	2.2571
NONHSAT218581.1	1.333163	4.178059	10.21673	8.37E-05	0.035866	2.256984
NONHSAT154087.1	1.738151	2.376426	10.21482	8.37E-05	0.035866	2.2561
NONHSAT223976.1	1.485573	4.127132	10.21178	8.39E-05	0.035866	2.254693
lnc-CRACR2A-8:1	1.158921	6.209449	10.21161	8.39E-05	0.035866	2.254614
NONHSAT206058.1	1.283887	1.713389	10.18436	8.51E-05	0.035866	2.241962
MSTRG.14267.1	1.908893	7.946334	10.1822	8.52E-05	0.035866	2.240957
NONHSAT220127.1	1.097996	6.435377	10.17937	8.53E-05	0.035866	2.239638
ENST00000428191.1	1.799919	4.310902	10.16353	8.60E-05	0.035866	2.232258

lnc-OR8G5-5:1	2.059076	2.099282	10.15966	8.62E-05	0.035866	2.230453
lnc-E2F6-1:1	-1.90292	3.603758	-10.1539	8.64E-05	0.035866	2.227762
ENST00000637583.1	1.971367	3.566366	10.15258	8.65E-05	0.035866	2.227145
NONHSAT198001.1	2.408993	5.055687	10.1471	8.67E-05	0.035866	2.224584
MSTRG.18011.9	-1.11778	7.596469	-10.143	8.69E-05	0.035866	2.222686
NONHSAT174850.1	-1.43504	3.224837	-10.1401	8.71E-05	0.035866	2.2213
lnc-AGBL1-5:1	1.32797	5.892156	10.08965	8.94E-05	0.035866	2.197625
lnc-MTRF1-1:1	1.574575	8.03103	10.08369	8.97E-05	0.035866	2.194816
lnc-TSR2-1:1	1.652311	2.989515	10.07671	9.00E-05	0.035866	2.191523
lnc-VWF-2:8	-1.29591	5.084476	-10.0611	9.07E-05	0.035866	2.184133
T187106	-1.70002	3.340289	-10.058	9.09E-05	0.035866	2.182662
NONHSAT185724.1	1.699086	2.048545	10.04393	9.16E-05	0.035866	2.176021
MSTRG.2665.1	-1.68618	2.581504	-10.0292	9.23E-05	0.035866	2.169043
lnc-CCDC82-9:1	1.475958	1.829763	10.02256	9.26E-05	0.035866	2.165882
MSTRG.5852.1	1.555522	2.192297	10.00513	9.35E-05	0.035866	2.157585
lnc-PIK3R1-10:1	2.011294	3.826484	10.00161	9.36E-05	0.035866	2.155908
NONHSAT155125.1	1.506764	1.833986	9.992463	9.41E-05	0.035866	2.151546
ENST00000449805.2	1.282768	6.004819	9.985274	9.45E-05	0.035866	2.148114
MSTRG.15081.1	-1.41791	5.847756	-9.97392	9.50E-05	0.035866	2.142688
lnc-FZD2-3:1	-1.57346	2.94601	-9.97217	9.51E-05	0.035866	2.141849
NONHSAT191702.1	1.626765	2.039775	9.969486	9.52E-05	0.035866	2.140565
NONHSAT161126.1	1.844564	2.146205	9.968049	9.53E-05	0.035866	2.139877
NONHSAT216808.1	1.438863	4.981948	9.967471	9.53E-05	0.035866	2.139601
NR_027112	1.420047	1.816234	9.932356	9.71E-05	0.035866	2.122748
lnc-ACTRT1-3:1	-1.44994	3.683432	-9.93201	9.72E-05	0.035866	2.122582
lnc-STX2-12:1	1.366709	5.420311	9.929257	9.73E-05	0.035866	2.121257
lnc-PGAP1-4:1	1.268468	5.276692	9.921472	9.77E-05	0.035866	2.117508
NONHSAT197807.1	2.210308	2.123853	9.921053	9.77E-05	0.035866	2.117307
MSTRG.27714.1	-1.389	4.094142	-9.91923	9.78E-05	0.035866	2.116429
lnc-FLRT2-10:2	-1.35548	2.130883	-9.91871	9.79E-05	0.035866	2.116178
NONHSAT185643.1	1.921854	2.191864	9.914484	9.81E-05	0.035866	2.114141
lnc-WASHC5-11:3	2.127146	2.473796	9.91371	9.81E-05	0.035866	2.113767
lnc-TSPY10-8:1	1.571883	3.834357	9.912116	9.82E-05	0.035866	2.112998
lnc-RABL3-5:1	-1.00549	9.664778	-9.91056	9.83E-05	0.035866	2.112247
lnc-MVD-3:3	1.070911	7.718214	9.907218	9.85E-05	0.035866	2.110635
lnc-FAM184A-2:1	-2.35522	2.509924	-9.90103	9.88E-05	0.035866	2.107648
NONHSAT161348.1	-1.65988	2.021154	-9.89513	9.91E-05	0.035866	2.104798
ENST00000607893.1	1.128931	11.12537	9.888704	9.94E-05	0.035866	2.101688
LINC01539:9	1.042939	6.205708	9.885366	9.96E-05	0.035866	2.100073
lnc-RDX-9:1	-1.03119	4.839398	-9.88514	9.96E-05	0.035866	2.099965
NONHSAT186640.1	2.046146	2.131077	9.883873	9.97E-05	0.035866	2.09935
ENST00000514339.1	1.357888	3.588621	9.878725	1.00E-04	0.035866	2.096856
MSTRG.23214.5	2.280648	2.622794	9.875934	0.0001	0.035866	2.095504
MSTRG.17975.21	2.528747	3.537119	9.872297	0.0001	0.035866	2.093741
NONHSAT202972.1	-1.65952	2.308633	-9.85568	0.000101	0.036059	2.085674
lnc-QRSL1-4:1	-1.63875	2.98064	-9.84278	0.000102	0.036135	2.079399
lnc-RWDD1-1:1	-1.1438	5.612629	-9.83863	0.000102	0.036135	2.07738
NONHSAT177397.1	1.779613	5.09756	9.818688	0.000103	0.036169	2.067651

NONHSAT173967.1	1.208807	1.707904	9.816154	0.000103	0.036169	2.066413
NONHSAT192665.1	1.737923	6.480583	9.813714	0.000104	0.036169	2.065221
ENST00000558967.1	1.243376	6.071809	9.810962	0.000104	0.036169	2.063875
ENST00000655217.1	1.501969	2.301255	9.780849	0.000105	0.036634	2.049123
lnc-TBC1D32-1:1	1.715763	1.956474	9.762999	0.000106	0.036862	2.04035
NONHSAT153806.1	2.019114	2.087397	9.729109	0.000108	0.037366	2.023635
lnc-CYTH4-2:1	2.165053	2.502853	9.725322	0.000109	0.037366	2.021762
NONHSAT149238.1	1.088966	1.712454	9.718764	0.000109	0.037371	2.018517
lnc-ZNF366-9:1	-1.3957	5.017043	-9.70953	0.00011	0.037432	2.013946
lnc-NUTM1-1:2	1.900288	3.574473	9.691948	0.000111	0.037577	2.005219
lnc-PTPN14-8:1	-1.59755	2.481014	-9.6829	0.000111	0.037577	2.000719
MSTRG.51008.21	2.411834	2.394019	9.678017	0.000111	0.037577	1.998291
NONHSAT183428.1	1.275943	1.742699	9.676931	0.000111	0.037577	1.997751
NONHSAT150719.1	-1.17071	3.974673	-9.66562	0.000112	0.037577	1.992117
NONHSAT164400.1	1.680123	5.13397	9.66546	0.000112	0.037577	1.992036
ENST00000426859.1	1.106587	7.136497	9.659404	0.000113	0.037577	1.989015
NONHSAT175159.1	1.663346	2.095434	9.638201	0.000114	0.037772	1.97842
NONHSAT205181.1	1.490717	1.823894	9.623251	0.000115	0.037772	1.970931
NR_002324	-1.50006	10.2388	-9.59914	0.000116	0.037772	1.958823
NONHSAT181150.1	1.348715	2.840471	9.581593	0.000117	0.037772	1.949983
ENST00000534076.2	1.848036	2.040517	9.580002	0.000118	0.037772	1.949181
ENST00000457033.5	-1.28161	12.75784	-9.57717	0.000118	0.037772	1.947754
lnc-KIAA0825-2:1	-1.09331	5.173582	-9.57646	0.000118	0.037772	1.947396
NONHSAT153597.1	1.734349	2.024337	9.574867	0.000118	0.037772	1.94659
lnc-MRPL39-31:1	-1.04675	2.711256	-9.56974	0.000118	0.037772	1.944001
ENST00000656703.1	-1.67577	2.447012	-9.56881	0.000118	0.037772	1.94353
lnc-MAL2-1:1	1.572168	3.185994	9.565892	0.000118	0.037772	1.942057
lnc-TMEM135-9:1	2.172456	2.201066	9.565518	0.000118	0.037772	1.941869
ENST00000640491.1	1.478222	7.496851	9.56523	0.000119	0.037772	1.941723
ENST00000417299.2	-1.17361	4.725215	-9.56241	0.000119	0.037772	1.940298
MSTRG.32762.2	1.548526	5.941202	9.561594	0.000119	0.037772	1.939885
T058954	1.325085	5.828587	9.530867	0.000121	0.037814	1.924316
lnc-FZD7-1:1	1.683365	4.743766	9.521817	0.000121	0.037814	1.919718
ENST00000414418.1	1.388173	5.818618	9.521303	0.000121	0.037814	1.919457
MSTRG.58741.1	1.754759	1.937986	9.519493	0.000122	0.037814	1.918536
lnc-MED12L-3:1	1.180772	4.260134	9.497471	0.000123	0.037814	1.907321
NONHSAT153975.1	1.521082	1.848157	9.496555	0.000123	0.037814	1.906854
NONHSAT219425.1	1.613632	2.067654	9.487788	0.000124	0.037814	1.902379
ENST00000422510.1	-2.09589	2.352787	-9.48679	0.000124	0.037814	1.901869
MSTRG.51667.1	1.463796	1.773546	9.484247	0.000124	0.037814	1.90057
ENST00000670759.1	-1.43395	2.095138	-9.48057	0.000124	0.037814	1.898689
lnc-RHEX-1:5	1.190096	4.281831	9.480419	0.000124	0.037814	1.898614
NONHSAT205570.1	1.092561	4.231719	9.476213	0.000124	0.037814	1.896463
MSTRG.5495.2	1.339729	3.61807	9.473413	0.000125	0.037814	1.895031
lnc-SMARCA4-4:1	1.005047	11.70612	9.473006	0.000125	0.037814	1.894822
NONHSAT199557.1	1.395526	5.836465	9.470588	0.000125	0.037814	1.893584
lnc-MX1-5:1	-1.06842	6.084558	-9.47058	0.000125	0.037814	1.893579
NONHSAT216263.1	-2.04437	2.190355	-9.46444	0.000125	0.03783	1.890438

lnc-CCDC18-2:1	-2.01373	3.283779	-9.44862	0.000126	0.038037	1.88232
T339380	-1.5981	2.647264	-9.44124	0.000127	0.038037	1.878532
T320443	1.665411	5.869047	9.430251	0.000128	0.038037	1.872879
MSTRG.33672.1	-1.49043	2.147304	-9.429	0.000128	0.038037	1.872236
lnc-MPDZ-4:1	-1.34849	5.880457	-9.42414	0.000128	0.038037	1.869734
NONHSAT174964.1	1.441809	5.839663	9.413712	0.000129	0.038037	1.864357
MSTRG.26947.1	2.182912	2.166538	9.409605	0.000129	0.038037	1.862237
MSTRG.21348.20	-2.22182	2.203268	-9.40931	0.000129	0.038037	1.862087
LINC01010:9	-1.64981	2.257336	-9.40063	0.00013	0.038037	1.857602
NR_033423	-1.45739	3.796688	-9.39286	0.00013	0.038037	1.853585
NONHSAT206852.1	2.850725	2.562076	9.389237	0.000131	0.038037	1.85171
NONHSAT150153.1	2.840038	2.513147	9.382942	0.000131	0.038037	1.84845
NR_002966	-1.88142	7.802454	-9.38112	0.000131	0.038037	1.847508
lnc-NUP107-1:1	-1.13051	6.617373	-9.37048	0.000132	0.038131	1.841986
NONHSAT189667.1	1.423678	4.336148	9.366523	0.000132	0.038131	1.839934
NONHSAT219215.1	1.395782	1.872188	9.340064	0.000134	0.038265	1.826172
NONHSAT170302.1	1.371489	1.708263	9.335168	0.000135	0.038265	1.82362
ENST00000647189.1	1.356897	4.105439	9.334946	0.000135	0.038265	1.823505
lnc-ARHGAP27-1:5	-1.5083	2.526578	-9.32863	0.000135	0.038265	1.820208
lnc-METTL15-11:1	-1.89812	3.63122	-9.32663	0.000135	0.038265	1.819167
NONHSAT196509.1	-1.75608	3.162196	-9.32145	0.000136	0.038265	1.816462
lnc-TRMU-3:1	-1.61312	5.006592	-9.31589	0.000136	0.038265	1.813553
NONHSAT182911.1	1.551545	3.784772	9.305213	0.000137	0.038265	1.807969
NONHSAT201980.1	-1.61751	4.717644	-9.29441	0.000138	0.038265	1.802309
lnc-HELQ-1:1	-1.49415	3.903194	-9.28039	0.000139	0.038265	1.794951
ENST00000544067.1	1.426078	4.067434	9.280337	0.000139	0.038265	1.794922
lnc-ITGA1-2:2	1.676656	2.002082	9.279086	0.000139	0.038265	1.794265
NONHSAT200324.1	1.232132	7.017636	9.277584	0.000139	0.038265	1.793476
MSTRG.22526.3	-1.48084	1.967171	-9.27215	0.00014	0.038265	1.790619
NONHSAT196550.1	-1.18495	6.930575	-9.27109	0.00014	0.038265	1.790062
lnc-ARL14EPL-3:1	-1.40152	4.167072	-9.26613	0.00014	0.038265	1.787453
lnc-EDC3-2:7	1.944086	2.022639	9.261939	0.00014	0.038265	1.785245
lnc-CXorf66-4:1	-1.39211	4.137513	-9.25893	0.000141	0.038265	1.783658
lnc-RRP1-3:1	-1.39139	4.492068	-9.25704	0.000141	0.038265	1.782663
MSTRG.31549.1	1.944997	2.001539	9.249252	0.000141	0.038331	1.778558
ENST00000558940.1	1.817011	3.751884	9.242591	0.000142	0.038373	1.775042
lnc-TRAFD1-2:1	-1.04761	7.454051	-9.22587	0.000143	0.038636	1.766202
NONHSAT183359.1	2.199822	2.65157	9.214614	0.000144	0.0387	1.76024
NONHSAT202039.1	1.961885	2.231906	9.213636	0.000144	0.0387	1.759722
NONHSAT218990.1	1.644381	4.162767	9.191802	0.000146	0.039081	1.748128
T319903	-1.18486	7.351671	-9.17092	0.000148	0.039158	1.737011
lnc-ARL5C-1:1	1.287213	7.070081	9.170477	0.000148	0.039158	1.736773
NONHSAT157107.1	-1.13169	4.54964	-9.16257	0.000149	0.039158	1.732553
lnc-RPS6KA6-1:1	-1.13937	7.258334	-9.16115	0.000149	0.039158	1.731796
NONHSAT201031.1	-1.0336	1.586268	-9.16071	0.000149	0.039158	1.731558
MSTRG.63783.1	-1.64486	3.052948	-9.14998	0.00015	0.039209	1.725824
ENST00000507932.1	1.300679	7.873176	9.137301	0.000151	0.039209	1.719039
NONHSAT196558.1	-2.19713	3.374286	-9.13692	0.000151	0.039209	1.718836

NR_002921	-1.4956	6.537812	-9.12967	0.000151	0.039209	1.714948
CERS3-AS1:5	-1.37579	1.740811	-9.12826	0.000152	0.039209	1.714194
NONHSAT181214.1	1.380926	4.462099	9.128006	0.000152	0.039209	1.714056
NONHSAT224442.1	1.711657	2.022126	9.115179	0.000153	0.039209	1.70717
lnc-UNC5CL-4:1	-1.00575	1.606089	-9.10461	0.000154	0.039209	1.701486
ENST00000433406.6	-1.29396	6.545807	-9.09688	0.000154	0.039209	1.697325
MSTRG.25532.3	-1.60232	2.564293	-9.0967	0.000154	0.039209	1.697224
T095343	1.430262	1.887215	9.092132	0.000155	0.039209	1.694765
lnc-PATE4-3:1	-1.91626	3.120959	-9.08439	0.000155	0.039209	1.690589
NR_109893	2.52893	2.330006	9.078693	0.000156	0.039209	1.687514
ENST00000607675.1	1.397969	1.883686	9.075302	0.000156	0.039209	1.685682
lnc-CPT2-4:2	1.090577	3.880197	9.070125	0.000157	0.039209	1.682883
lnc-HUS1B-3:2	-1.0378	8.93736	-9.0633	0.000157	0.039209	1.679193
LINC02387:5	-1.69425	3.063042	-9.05305	0.000158	0.039209	1.673639
NONHSAT193062.1	-1.11557	6.836788	-9.05279	0.000158	0.039209	1.673497
NONHSAT206636.1	1.032153	5.100432	9.048039	0.000159	0.039209	1.670923
NR_027046	-1.12021	6.053628	-9.03828	0.00016	0.039209	1.665628
NONHSAT171612.1	1.216854	4.18652	9.036256	0.00016	0.039209	1.664527
lnc-CCDC68-2:1	-1.07854	8.491209	-9.03412	0.00016	0.039209	1.663369
NONHSAT193770.1	1.15899	6.143934	9.03179	0.00016	0.039209	1.662101
MSTRG.34673.1	-1.92332	5.990653	-9.03004	0.00016	0.039209	1.661151
MSTRG.56604.1	2.585331	2.393693	9.023618	0.000161	0.039209	1.657656
lnc-CNOT1-2:1	-2.20416	2.529973	-9.02233	0.000161	0.039209	1.656956
ENST00000253848.3	-1.45165	1.903425	-9.02075	0.000161	0.039209	1.656095
ENST00000612725.1	-1.48011	3.232559	-9.01931	0.000161	0.039209	1.655312
lnc-RNLS-3:1	1.487535	1.856407	9.012438	0.000162	0.039271	1.651568
ENST00000669110.1	2.574213	2.400218	9.004863	0.000163	0.039292	1.647437
NONHSAT170266.1	1.258418	1.683942	9.003287	0.000163	0.039292	1.646577
ENST00000624919.1	-1.28426	3.674949	-8.98802	0.000164	0.039327	1.638238
T306349	1.68165	6.467399	8.973151	0.000166	0.039327	1.630097
lnc-PRDM14-4:1	-2.36354	2.451186	-8.95606	0.000167	0.039327	1.620722
NONHSAT169160.1	1.878291	3.707548	8.951532	0.000168	0.039327	1.618232
MSTRG.30364.1	1.861755	1.976856	8.951209	0.000168	0.039327	1.618054
ENST00000590604.1	1.325285	4.40086	8.950678	0.000168	0.039327	1.617763
MSTRG.22925.2	1.325348	4.231236	8.942916	0.000169	0.039327	1.613494
ENST00000612584.1	2.029361	2.118511	8.942114	0.000169	0.039327	1.613052
NONHSAT207851.1	2.071397	2.091368	8.941184	0.000169	0.039327	1.61254
NONHSAT187784.1	1.303881	4.492906	8.937147	0.000169	0.039327	1.610317
NONHSAT200630.1	1.02281	4.377473	8.935894	0.000169	0.039327	1.609628
NONHSAT186218.1	1.10391	1.592973	8.92802	0.00017	0.039327	1.605288
NONHSAT200308.1	-1.91478	3.538937	-8.92699	0.00017	0.039327	1.604721
ENST00000445785.6	-1.37295	1.943864	-8.92542	0.00017	0.039327	1.603853
NONHSAT176220.1	-1.32108	4.948961	-8.92516	0.00017	0.039327	1.60371
T269433	1.460152	1.764098	8.913614	0.000172	0.039327	1.597336
NONHSAT161191.1	2.532525	2.646512	8.908743	0.000172	0.039327	1.594644
lnc-TMEM17-1:1	1.459854	6.073343	8.907114	0.000172	0.039327	1.593743
MSTRG.35826.1	1.139259	1.864557	8.906205	0.000172	0.039327	1.593241
ENST00000510211.1	2.017119	2.105238	8.897438	0.000173	0.039441	1.588389

NONHSAT178728.1	-1.25641	1.741882	-8.88907	0.000174	0.039468	1.583753
NONHSAT152008.1	2.002671	3.300857	8.888616	0.000174	0.039468	1.583501
NONHSAT224511.1	1.712896	4.626826	8.868713	0.000176	0.039768	1.57245
NONHSAT203440.1	1.373046	4.63511	8.864348	0.000177	0.039768	1.570023
lnc-KIAA0408-4:2	-1.40062	3.598488	-8.85698	0.000177	0.039852	1.565924
ENST00000653118.1	1.395792	5.709966	8.84737	0.000178	0.039933	1.560567
NONHSAT209334.1	-1.72972	4.257581	-8.84604	0.000179	0.039933	1.559824
ENST00000520572.2	-1.2193	3.14639	-8.83028	0.00018	0.040128	1.551024
lnc-JUN-5:16	-1.96483	2.769819	-8.8012	0.000183	0.040664	1.534737
NONHSAT165393.1	-1.0295	5.775125	-8.80048	0.000183	0.040664	1.534332
NONHSAT207754.1	1.338084	1.699827	8.788627	0.000185	0.04083	1.527676
lnc-SLC35F5-10:7	1.712296	6.119105	8.782873	0.000185	0.04083	1.524441
ENST00000624760.1	-1.03672	6.994149	-8.77512	0.000186	0.04083	1.520076
lnc-SMPX-6:1	2.30749	2.243113	8.771571	0.000187	0.04083	1.518077
MSTRG.50587.1	2.315093	2.178731	8.755536	0.000188	0.040871	1.509031
NONHSAT214255.1	1.362708	5.538865	8.755206	0.000189	0.040871	1.508844
MSTRG.6431.1	1.534111	8.598841	8.75216	0.000189	0.040871	1.507123
ENST00000444244.1	-1.06915	10.30884	-8.74947	0.000189	0.040871	1.505601
lnc-SP3-14:1	-1.6232	3.819826	-8.74062	0.00019	0.040871	1.500595
ENST00000437080.1	1.451957	4.964309	8.740256	0.00019	0.040871	1.500391
ENST00000649691.1	1.13162	6.945768	8.738518	0.00019	0.040871	1.499408
ENST00000430078.1	2.552123	4.085521	8.732169	0.000191	0.040871	1.495812
MSTRG.16982.1	1.998967	2.188778	8.722516	0.000192	0.040871	1.490339
ENST00000456535.1	-1.21395	1.65	-8.71925	0.000193	0.040871	1.488486
lnc-C1orf43-1:1	-1.07581	1.685648	-8.71723	0.000193	0.040871	1.48734
lnc-ATP5F1A-4:1	-1.44979	1.814816	-8.71131	0.000194	0.040871	1.483974
lnc-C1QTNF9B-3:1	2.303896	2.864693	8.709539	0.000194	0.040871	1.482969
NONHSAT206529.1	1.217316	3.978597	8.709109	0.000194	0.040871	1.482724
lnc-CCL25-1:3	-1.55762	3.708999	-8.70642	0.000194	0.040871	1.481195
NONHSAT159921.1	2.315327	2.566713	8.70193	0.000195	0.040871	1.478642
MSTRG.61572.1	1.702531	4.563924	8.692067	0.000196	0.040871	1.473026
lnc-MC5R-1:16	-1.25346	4.527937	-8.69191	0.000196	0.040871	1.472938
lnc-MEIS2-8:1	-2.20086	2.727421	-8.68842	0.000196	0.040871	1.470948
NONHSAT190227.1	1.6275	2.601173	8.686338	0.000196	0.040871	1.46976
NONHSAT165231.1	1.754269	2.245301	8.676398	0.000198	0.040871	1.464087
lnc-RNF150-7:1	-1.36438	1.944326	-8.67333	0.000198	0.040871	1.462337
lnc-NPM2-3:1	-1.33395	4.944619	-8.67064	0.000198	0.040871	1.460796
lnc-RNF149-4:1	-1.73488	2.02752	-8.66431	0.000199	0.040871	1.45718
NONHSAT177001.1	1.799213	3.535622	8.660942	0.000199	0.040871	1.455252
NONHSAT222775.1	-1.23764	6.355663	-8.65891	0.0002	0.040871	1.454086
MSTRG.32923.1	1.132432	7.197451	8.653236	0.0002	0.040871	1.450839
NONHSAT167866.1	1.699686	5.795102	8.651226	0.000201	0.040871	1.449688
NONHSAT178987.1	1.745192	4.252033	8.649377	0.000201	0.040871	1.448628
NONHSAT169170.1	-1.34726	3.907015	-8.64143	0.000202	0.040871	1.444068
T064024	1.153461	3.786674	8.641187	0.000202	0.040871	1.443931
lnc-CLGN-2:1	-1.61595	4.84391	-8.63728	0.000202	0.040871	1.441686
T278895	2.295902	2.224713	8.631795	0.000203	0.040871	1.438538
NONHSAT188031.1	1.539533	1.79909	8.630098	0.000203	0.040871	1.437562

MSTRG.35862.1	-1.18578	4.074371	-8.63005	0.000203	0.040871	1.437536
lnc-YWHAZ-4:1	1.403748	3.863128	8.62587	0.000204	0.040871	1.435131
lnc-ANKRD55-8:1	2.644144	2.920718	8.623878	0.000204	0.040871	1.433986
lnc-MVB12B-7:1	-1.02295	5.188478	-8.62292	0.000204	0.040871	1.433433
lnc-RNASET2-1:8	-1.62975	3.840155	-8.62243	0.000204	0.040871	1.433151
NONHSAT193617.1	-1.1133	5.201597	-8.62153	0.000204	0.040871	1.432637
ENST00000561538.1	1.384387	2.041338	8.615801	0.000205	0.040871	1.429337
ENST00000610840.1	-1.86143	4.543845	-8.61065	0.000206	0.040871	1.426367
lnc-KCNA4-8:1	-2.15193	2.557776	-8.60823	0.000206	0.040871	1.424977
ENST00000658633.1	2.864674	2.621217	8.60794	0.000206	0.040871	1.424807
lnc-KIAA1328-12:1	-1.08267	5.191679	-8.605	0.000206	0.040871	1.423114
LINC02143:6	1.203382	1.817177	8.601028	0.000207	0.040871	1.42082
lnc-CCDC82-11:1	1.495536	1.830058	8.599417	0.000207	0.040871	1.41989
MSTRG.47614.1	-1.34022	1.762904	-8.59889	0.000207	0.040871	1.419585
NONHSAT177159.1	1.259085	1.749837	8.59548	0.000207	0.040871	1.417617
MSTRG.1368.5	1.749792	1.97606	8.595178	0.000208	0.040871	1.417443
MSTRG.48011.1	1.025312	6.735701	8.581655	0.000209	0.040962	1.409625
NONHSAT217314.1	1.395834	3.843102	8.579645	0.000209	0.040962	1.408461
ENST00000374014.3	2.45864	2.344321	8.578035	0.00021	0.040962	1.40753
lnc-BDH2-5:1	-2.03639	3.523205	-8.5771	0.00021	0.040962	1.406986
lnc-GKN2-1:1	1.794567	2.018457	8.575481	0.00021	0.040962	1.406051
ENST00000666584.1	1.298586	4.666554	8.564827	0.000211	0.041097	1.399876
lnc-CD46-5:3	-1.07189	4.922591	-8.56205	0.000212	0.041097	1.398266
ENST00000474444.1	1.17169	6.465654	8.560047	0.000212	0.041097	1.397102
POC1B-AS1:29	-1.90614	2.30955	-8.54736	0.000214	0.041097	1.38973
NONHSAT196460.1	-1.34127	5.490091	-8.54632	0.000214	0.041097	1.389128
NONHSAT201513.1	1.313912	5.665675	8.545868	0.000214	0.041097	1.388864
lnc-ATP6AP2-11:1	1.332872	1.750223	8.542759	0.000214	0.041097	1.387056
CSTF3-DT:16	1.166832	8.260982	8.541706	0.000214	0.041097	1.386443
MSTRG.60394.1	-1.68805	2.530237	-8.52877	0.000216	0.041318	1.378909
ENST00000508713.1	-1.74934	5.143066	-8.52608	0.000216	0.041318	1.377339
NONHSAT196746.1	1.859862	4.332155	8.522196	0.000217	0.041318	1.375074
LINC01117:14	-2.00319	2.873461	-8.52048	0.000217	0.041318	1.374072
T346136	-1.8346	3.430349	-8.50729	0.000219	0.041339	1.366367
NONHSAT164997.1	-1.72748	3.217988	-8.50503	0.000219	0.041339	1.365045
lnc-CRP-2:3	-1.31784	4.900193	-8.49908	0.00022	0.041339	1.361567
lnc-CLDN5-2:1	-1.05629	5.580592	-8.49765	0.00022	0.041339	1.360726
NONHSAT162037.1	-1.34308	4.334347	-8.49739	0.00022	0.041339	1.360576
lnc-RIPK4-4:1	1.605229	4.736425	8.49387	0.000221	0.041339	1.358513
NONHSAT191166.1	2.472275	2.264059	8.491519	0.000221	0.041339	1.357135
ENST00000414452.1	-1.2044	6.62827	-8.49061	0.000221	0.041339	1.356601
ENST00000617439.1	-1.06707	5.767462	-8.48909	0.000221	0.041339	1.355714
NR_110607	-1.24062	3.644163	-8.47984	0.000223	0.04142	1.350287
lnc-PAPOLA-3:1	1.19883	3.546814	8.472611	0.000224	0.041528	1.34604
ENST00000598561.1	2.112423	2.891501	8.458373	0.000226	0.041642	1.337665
lnc-DAGLA-3:2	1.988947	2.418498	8.446611	0.000227	0.041642	1.330735
MSTRG.18857.1	1.205955	6.129226	8.437564	0.000229	0.041642	1.325396
NONHSAT198305.1	1.622942	1.837935	8.436663	0.000229	0.041642	1.324863

ENST00000621404.1	-1.44198	3.380983	-8.43503	0.000229	0.041642	1.3239
NONHSAT195938.1	-1.07398	1.575678	-8.43457	0.000229	0.041642	1.323625
ENST00000412485.1	1.420847	8.727924	8.433148	0.000229	0.041642	1.322787
lnc-ATP6V1C1-3:1	1.198655	1.640702	8.431933	0.000229	0.041642	1.322069
lnc-ALDH1B1-8:1	1.462687	1.794874	8.429636	0.00023	0.041642	1.320712
MSTRG.60768.1	3.27893	3.31264	8.426958	0.00023	0.041642	1.319128
PGM5P4-AS1:11	-1.86558	2.29576	-8.42686	0.00023	0.041642	1.31907
NONHSAT162888.1	-1.33625	4.128107	-8.42161	0.000231	0.041642	1.315966
NR_002325	-1.38963	10.22261	-8.42014	0.000231	0.041642	1.315095
lnc-TMPRSS15-22:1	1.892923	2.617826	8.418328	0.000231	0.041642	1.314022
ENST00000452326.1	-2.11073	2.763658	-8.41695	0.000231	0.041642	1.313205
NONHSAT192126.1	2.245696	4.670636	8.415152	0.000232	0.041642	1.31214
ENST00000511875.1	1.929404	2.050098	8.409611	0.000232	0.041655	1.308857
NONHSAT185214.1	2.114985	2.147525	8.402761	0.000233	0.041655	1.304794
ENST00000588342.1	-1.02339	1.571576	-8.39905	0.000234	0.041655	1.302591
NONHSAT214838.1	1.581158	1.858257	8.397458	0.000234	0.041655	1.301646
NR_002918	-1.30338	11.69478	-8.39527	0.000235	0.041655	1.300348
lnc-RAB1A-9:1	-1.05464	2.738286	-8.3946	0.000235	0.041655	1.299951
NONHSAT216598.1	1.272134	5.3928	8.389733	0.000235	0.041701	1.297056
lnc-MTNR1A-66:1	-2.35882	2.915562	-8.38712	0.000236	0.041701	1.295503
ENST00000423187.1	-1.46109	4.169704	-8.37363	0.000238	0.041801	1.287473
NONHSAT212907.1	1.855105	2.187277	8.370163	0.000238	0.041801	1.285406
NONHSAT209795.1	1.589764	2.015844	8.361268	0.00024	0.041801	1.2801
NR_003016	-1.17223	9.976708	-8.35873	0.00024	0.041801	1.278582
MSTRG.40703.1	2.381296	2.540009	8.358275	0.00024	0.041801	1.278313
ENST00000608684.1	-1.13984	6.643662	-8.35224	0.000241	0.041801	1.274708
NONHSAT216570.1	1.007103	1.573373	8.351545	0.000241	0.041801	1.274292
lnc-BST2-3:2	-1.89638	4.847789	-8.34977	0.000241	0.041801	1.273232
ENST00000435108.1	1.886581	7.277521	8.34976	0.000241	0.041801	1.273225
MSTRG.44130.1	2.032894	2.051051	8.349528	0.000241	0.041801	1.273086
lnc-GOLGA8A-2:3	-1.2444	4.35588	-8.34467	0.000242	0.041801	1.27018
NONHSAT156336.1	1.843567	2.208758	8.343011	0.000242	0.041801	1.269188
lnc-RNF152-7:1	-1.42547	4.116807	-8.34282	0.000242	0.041801	1.269073
ENST00000503052.3	-1.18205	6.789388	-8.34141	0.000243	0.041801	1.268228
ENST00000541723.5	1.154837	4.100981	8.325024	0.000245	0.042158	1.258411
lnc-MFSD14B-12:1	1.504022	4.284963	8.316138	0.000246	0.042214	1.253076
MSTRG.13905.1	-1.89203	2.793258	-8.31329	0.000247	0.042214	1.251366
MSTRG.29979.1	-2.41191	2.942262	-8.31197	0.000247	0.042214	1.250572
lnc-GABRE-6:1	1.202744	5.871738	8.305431	0.000248	0.042243	1.24664
T109200	1.019609	1.609793	8.301177	0.000249	0.042284	1.24408
ENST00000417927.1	1.100935	1.771769	8.297798	0.000249	0.042302	1.242045
NONHSAT196125.1	1.275329	4.736028	8.293941	0.00025	0.04233	1.239721
lnc-ALCAM-8:7	2.058636	5.728404	8.288812	0.000251	0.04233	1.23663
T193510	-1.02639	4.399018	-8.28868	0.000251	0.04233	1.236553
MSTRG.59388.1	-1.79741	2.099612	-8.28339	0.000251	0.0424	1.233357
ENST00000500989.2	1.774666	3.747184	8.279462	0.000252	0.042433	1.230988
MSTRG.20402.1	-1.05474	10.71802	-8.26745	0.000254	0.042612	1.223726
NR_104999	-1.44652	7.621356	-8.25775	0.000256	0.042773	1.217856

NONHSAT215966.1	1.109197	4.108092	8.250845	0.000257	0.042773	1.213673
MSTRG.56042.1	1.204899	7.524096	8.245581	0.000257	0.042844	1.21048
lnc-LRRC40-5:1	-1.19727	5.423859	-8.23856	0.000259	0.042963	1.206217
lnc-PTMA-5:1	1.476409	3.468828	8.232296	0.00026	0.043061	1.202412
NONHSAT197913.1	-1.18372	5.431021	-8.22314	0.000261	0.043237	1.196841
ENST00000462801.2	1.099928	4.7199	8.215489	0.000262	0.043306	1.192183
MSTRG.4872.1	-1.1057	5.601638	-8.20341	0.000264	0.043528	1.184818
NONHSAT209413.1	1.162584	3.927966	8.201767	0.000265	0.043528	1.183814
NR_030732	1.009827	6.7421	8.199612	0.000265	0.043528	1.182498
NONHSAT168672.1	1.398791	1.726058	8.191964	0.000266	0.043666	1.177825
lnc-CTAGE1-5:1	-1.20131	2.07489	-8.18857	0.000267	0.043666	1.175749
lnc-GABRA5-5:1	-1.75687	3.013266	-8.18687	0.000267	0.043666	1.174711
ENST00000671050.1	-1.83677	2.663702	-8.18395	0.000268	0.043676	1.172924
NONHSAT177579.1	2.120505	2.084854	8.177568	0.000269	0.043729	1.169014
ENST00000566876.1	1.178373	1.650095	8.176958	0.000269	0.043729	1.16864
ENST00000513871.1	1.903865	2.133623	8.166914	0.000271	0.043937	1.162482
lnc-CD163-3:1	-2.3071	2.672362	-8.15744	0.000272	0.04413	1.156667
lnc-WNT7B-4:1	1.855847	6.141373	8.152312	0.000273	0.044203	1.153514
NONHSAT220592.1	-1.02234	3.569515	-8.13365	0.000276	0.044529	1.142027
lnc-XRN2-7:1	1.62235	2.611713	8.128138	0.000277	0.044529	1.138625
NONHSAT170341.1	1.776784	2.146507	8.128073	0.000277	0.044529	1.138585
lnc-DNAJC24-8:1	1.896399	2.313363	8.126953	0.000278	0.044529	1.137894
NONHSAT186705.1	1.274966	4.469577	8.125184	0.000278	0.044529	1.136803
NONHSAT200337.1	1.658458	3.450632	8.120109	0.000279	0.044545	1.133669
lnc-PSG2-3:1	1.629913	5.192049	8.113526	0.00028	0.044609	1.129601
ENST00000653036.1	1.224672	5.017372	8.112169	0.00028	0.044609	1.128762
NONHSAT221857.1	-1.1406	4.997469	-8.11042	0.00028	0.044609	1.127678
lnc-CCKAR-6:1	1.472034	4.301856	8.107849	0.000281	0.044612	1.12609
lnc-FOXO4-5:1	-1.35234	5.292348	-8.10105	0.000282	0.044735	1.12188
MSTRG.61221.1	2.705937	5.406902	8.096558	0.000283	0.044793	1.119098
LINC01571:14	1.045231	7.808107	8.093004	0.000284	0.044825	1.116895
NONHSAT149227.1	-1.11346	5.798928	-8.08856	0.000284	0.044882	1.11414
NONHSAT218748.1	1.267307	3.869358	8.069126	0.000288	0.045271	1.102064
lnc-SH3GL2-1:1	-1.14746	4.727226	-8.06513	0.000289	0.045271	1.099579
MSTRG.6762.4	1.625967	1.828394	8.063109	0.000289	0.045271	1.098319
lnc-CHST15-1:2	2.073082	2.057458	8.061733	0.000289	0.045271	1.097462
RBMS3-AS3:6	-1.0786	7.146954	-8.05964	0.00029	0.045271	1.09616
NONHSAT167733.1	2.093273	2.158603	8.053481	0.000291	0.045271	1.09232
lnc-RAB3C-4:1	1.512509	2.889116	8.0516	0.000291	0.045271	1.091146
T118756	1.693044	4.138788	8.040578	0.000293	0.045409	1.084266
lnc-SMC6-2:1	-1.32354	1.99526	-8.0365	0.000294	0.045409	1.08172
ENST00000454526.1	1.559904	3.177307	8.032909	0.000295	0.045444	1.079473
MSTRG.29537.16	-1.89281	3.742442	-8.02816	0.000296	0.045444	1.076502
lnc-HRH3-2:1	-1.04017	2.232144	-8.01912	0.000297	0.04557	1.070841
NONHSAT200647.1	1.497893	2.269999	8.006193	0.0003	0.04568	1.062733
NONHSAT203154.1	1.048824	6.322527	8.004816	0.0003	0.04568	1.061868
NONHSAT157973.1	1.77799	3.627881	8.003841	0.0003	0.04568	1.061256
NONHSAT158772.1	-1.16078	1.676319	-8.00206	0.000301	0.04568	1.060137

T165321	-1.78409	2.463869	-8.00198	0.000301	0.04568	1.060084
ENST00000665254.1	-1.68637	2.607213	-8.00077	0.000301	0.04568	1.059325
ENST00000570409.1	-1.32708	5.833431	-7.99759	0.000302	0.04568	1.057327
lnc-FBXW11-4:1	-1.87588	2.655812	-7.99663	0.000302	0.04568	1.056728
MSTRG.45337.1	1.285145	7.031486	7.986982	0.000304	0.045758	1.050655
NONHSAT153483.1	2.045295	6.251012	7.985368	0.000304	0.045758	1.049639
lnc-TLX3-3:1	-2.06879	2.375139	-7.97407	0.000306	0.045806	1.042518
lnc-NAV1-2:5	-1.87862	2.352401	-7.97389	0.000306	0.045806	1.042403
NR_028348	1.246986	3.378498	7.970506	0.000307	0.045839	1.040271
ENST00000602789.1	-2.46977	2.975522	-7.9634	0.000308	0.045982	1.035784
NONHSAT166887.1	-1.50843	2.023391	-7.95227	0.000311	0.046246	1.028749
ENST00000504082.1	-1.35627	4.262399	-7.94282	0.000312	0.046264	1.02277
MSTRG.48865.1	-1.02413	5.841162	-7.94115	0.000313	0.046264	1.021708
NONHSAT150534.1	1.347448	7.073616	7.940337	0.000313	0.046264	1.021193
MSTRG.18207.1	1.51052	2.105618	7.930714	0.000315	0.046418	1.015091
MSTRG.27914.1	2.065916	2.11294	7.924219	0.000316	0.046546	1.010967
MSTRG.48292.1	-1.09719	3.786598	-7.91608	0.000318	0.046642	1.005794
lnc-CNIH1-3:1	-1.19567	7.340357	-7.91436	0.000318	0.046642	1.004698
NR_002952	-1.01669	9.613039	-7.90958	0.000319	0.046719	1.001661
T285662	-1.45182	3.263318	-7.89738	0.000322	0.046939	0.993889
lnc-POC5-3:1	-1.18526	8.422536	-7.89582	0.000322	0.046939	0.992891
lnc-RTP2-1:2	-2.03389	2.177128	-7.89408	0.000322	0.046939	0.99178
T188397	-1.59694	1.89828	-7.89249	0.000323	0.046939	0.990767
lnc-METTL15-5:3	-1.21819	5.255282	-7.88804	0.000324	0.046971	0.987929
MSTRG.30609.2	-1.09547	5.458468	-7.88047	0.000325	0.047068	0.983093
NONHSAT218594.1	1.344518	5.742994	7.869925	0.000328	0.047258	0.976345
lnc-NAA16-1:1	1.846039	3.201593	7.861769	0.000329	0.047259	0.97112
lnc-TUBE1-3:1	2.156436	5.165022	7.861253	0.000329	0.047259	0.970789
ENST00000618697.1	-1.24084	6.308909	-7.86066	0.00033	0.047259	0.97041
NONHSAT174829.1	-1.47582	10.72344	-7.85946	0.00033	0.047259	0.969638
NONHSAT201469.1	1.036832	6.751156	7.856532	0.00033	0.047259	0.967763
lnc-CDY2B-13:1	-1.04594	5.783458	-7.85558	0.000331	0.047259	0.967153
ENST00000526186.1	1.003385	3.869252	7.852548	0.000331	0.047259	0.965206
lnc-ARL4A-3:1	-1.01297	4.08093	-7.84786	0.000332	0.047259	0.962199
NONHSAT165582.1	1.551765	4.614453	7.845999	0.000333	0.047259	0.961001
NONHSAT172234.1	1.373844	3.735783	7.844061	0.000333	0.047259	0.959756
lnc-CSNK1G1-1:3	-1.03177	6.480356	-7.84364	0.000333	0.047259	0.959486
lnc-GINS2-2:4	2.497015	3.320882	7.841216	0.000334	0.047259	0.957928
NONHSAT212461.1	-1.31616	4.600724	-7.83295	0.000336	0.047259	0.952608
NONHSAT204473.1	-1.18762	4.420491	-7.83207	0.000336	0.047259	0.952042
NONHSAT201745.1	-1.14487	1.718083	-7.82679	0.000337	0.047259	0.948646
T194191	1.904356	2.377764	7.825596	0.000337	0.047259	0.947876
NONHSAT197657.1	-2.15909	2.199195	-7.82474	0.000337	0.047259	0.947323
lnc-IGSF10-4:1	1.519852	5.658953	7.824635	0.000337	0.047259	0.947256
lnc-TTC13-3:1	1.599905	1.910879	7.822455	0.000338	0.047259	0.945851
NONHSAT217271.1	-1.49165	4.29915	-7.81457	0.00034	0.047259	0.940766
NONHSAT201383.1	1.069629	1.733646	7.812246	0.00034	0.047259	0.939266
T184755	1.102442	5.040789	7.811771	0.00034	0.047259	0.938959

MSTRG.11313.4	1.564064	5.259843	7.810455	0.000341	0.047259	0.93811
ENST00000412996.1	-1.1949	3.746944	-7.80905	0.000341	0.047259	0.937199
lnc-INHBC-1:3	-1.69008	3.234356	-7.80798	0.000341	0.047259	0.936509
NONHSAT197998.1	1.088531	3.251824	7.800152	0.000343	0.047266	0.931453
NONHSAT154345.1	-1.17384	5.08166	-7.79971	0.000343	0.047266	0.931165
NONHSAT218479.1	1.327516	1.882879	7.797781	0.000344	0.047266	0.929919
NONHSAT191848.1	1.690371	4.228726	7.797596	0.000344	0.047266	0.929799
MSTRG.51115.1	1.544974	2.086995	7.793439	0.000344	0.047266	0.92711
NONHSAT167810.1	1.551352	1.815783	7.787578	0.000346	0.047266	0.923314
MSTRG.5185.1	-1.07204	4.336494	-7.78657	0.000346	0.047266	0.922659
NONHSAT154027.1	-1.24688	3.336218	-7.78402	0.000347	0.047266	0.921009
NONHSAT206219.1	2.022332	5.45067	7.782981	0.000347	0.047266	0.920335
ENST00000547084.1	2.526733	4.411034	7.782739	0.000347	0.047266	0.920178
lnc-DEGS2-2:7	-1.45923	2.868504	-7.7797	0.000348	0.047266	0.918205
lnc-ECHDC1-3:2	-1.51827	4.8759	-7.77678	0.000348	0.047266	0.916316
ENST00000658275.1	1.26395	5.850726	7.774794	0.000349	0.047266	0.915025
ENST00000614966.1	-1.0385	7.047244	-7.76835	0.00035	0.04739	0.910841
MSTRG.31522.1	-1.17439	5.222265	-7.76455	0.000351	0.04739	0.908373
ENST00000421206.1	-1.27184	5.201183	-7.76424	0.000351	0.04739	0.908169
NR_002912	-1.1643	12.99009	-7.76273	0.000352	0.04739	0.90719
ENST00000325042.2	-1.05097	7.553376	-7.75839	0.000353	0.0474	0.904363
lnc-ZCCHC24-9:1	1.803175	3.875228	7.755179	0.000353	0.047407	0.902276
lnc-ARL4C-13:1	-1.96049	2.319495	-7.75415	0.000354	0.047407	0.901604
MSTRG.44762.38	-1.60015	2.822381	-7.74451	0.000356	0.047596	0.895327
lnc-SNX8-3:1	1.542839	6.312031	7.740712	0.000357	0.047596	0.892851
NONHSAT183346.1	1.512273	4.570662	7.737799	0.000357	0.047596	0.89095
FTX:24	-1.01573	5.848188	-7.73708	0.000358	0.047596	0.890479
NONHSAT156338.1	2.330512	3.761886	7.734752	0.000358	0.047596	0.888962
MSTRG.56204.1	-2.00975	3.049424	-7.73394	0.000358	0.047596	0.888431
lnc-DAW1-5:1	-1.53191	4.854847	-7.7276	0.00036	0.047596	0.884291
lnc-CREBL2-3:1	-1.71224	2.474949	-7.72393	0.000361	0.047596	0.88189
NONHSAT201972.1	1.368203	6.624553	7.721727	0.000361	0.047596	0.880451
NONHSAT153476.1	1.119883	6.076709	7.718606	0.000362	0.047596	0.87841
ENST00000520426.1	2.580473	2.898899	7.716911	0.000362	0.047596	0.877301
NONHSAT199158.1	-1.12434	1.812943	-7.71232	0.000364	0.047596	0.874294
T146532	1.211405	6.645283	7.707324	0.000365	0.047596	0.871022
lnc-SNTB1-3:1	-1.06679	6.13742	-7.70328	0.000366	0.047596	0.868371
ENST00000471921.2	1.806611	2.017889	7.703174	0.000366	0.047596	0.868302
NONHSAT184521.1	1.694734	2.094954	7.696482	0.000367	0.047596	0.863911
NONHSAT175386.1	-1.87282	3.522598	-7.69253	0.000368	0.047596	0.861315
ENST00000622740.3	1.026738	1.558911	7.690643	0.000369	0.047596	0.860076
lnc-C9orf92-2:1	-1.12847	4.365236	-7.68752	0.00037	0.047596	0.858024
lnc-PIEZO2-9:1	1.324888	7.023692	7.687256	0.00037	0.047596	0.857851
lnc-BATF-1:1	-1.92426	6.376442	-7.68323	0.000371	0.047596	0.855207
ENST00000658676.1	1.265513	2.706643	7.682441	0.000371	0.047596	0.854685
NONHSAT190544.1	2.263826	3.332199	7.680287	0.000371	0.047596	0.853268
ENST00000609497.5	1.6468	2.220702	7.680084	0.000371	0.047596	0.853134
NONHSAT167487.1	1.038156	5.585088	7.677559	0.000372	0.047596	0.851473

lnc-BTN3A2-1:4	-1.31805	1.975176	-7.67685	0.000372	0.047596	0.851009
MSTRG.19675.1	-1.196	3.059792	-7.67247	0.000373	0.047596	0.848119
NONHSAT155053.1	1.364329	6.153431	7.667793	0.000374	0.047596	0.84504
NONHSAT216432.1	1.775432	6.763035	7.664714	0.000375	0.047596	0.84301
NR_026813	-1.09173	8.568336	-7.66255	0.000376	0.047596	0.841585
ENST00000428646.1	1.103835	6.989154	7.660899	0.000376	0.047596	0.840494
NONHSAT169833.1	1.005527	5.539118	7.65083	0.000379	0.047722	0.833845
lnc-HILPDA-1:6	-1.34288	3.568514	-7.64717	0.00038	0.047722	0.83143
lnc-TOX3-6:1	-1.05428	4.612622	-7.64455	0.00038	0.047722	0.829695
NONHSAT196576.1	1.05763	1.561059	7.644367	0.00038	0.047722	0.829573
lnc-RFX3-4:1	-1.25098	4.83229	-7.64167	0.000381	0.047722	0.827793
lnc-NBPF3-9:1	-1.52411	2.584374	-7.63755	0.000382	0.047722	0.825063
NONHSAT172317.1	1.491802	1.852903	7.627217	0.000385	0.047722	0.818216
ENST00000454135.1	1.262617	4.149039	7.625707	0.000385	0.047722	0.817216
MSTRG.7158.1	-1.19525	3.314362	-7.62548	0.000385	0.047722	0.817064
NONHSAT163834.1	1.353635	5.13375	7.623699	0.000386	0.047722	0.815884
T133891	1.393401	3.929686	7.618619	0.000387	0.047722	0.812512
lnc-EMSY-1:1	1.023673	6.777195	7.612533	0.000389	0.047722	0.80847
NONHSAT196935.1	-1.09017	4.055862	-7.61093	0.000389	0.047722	0.807405
lnc-FRA10AC1-3:1	1.119093	4.879601	7.607559	0.00039	0.047773	0.805164
lnc-IQCA1-3:1	-1.93875	2.500542	-7.60345	0.000391	0.047847	0.802432
NONHSAT214652.1	1.592055	3.596741	7.600648	0.000392	0.04787	0.800567
NONHSAT190511.1	2.743118	2.718617	7.593334	0.000394	0.04787	0.795696
ENST00000554679.1	1.101108	5.041226	7.59309	0.000394	0.04787	0.795534
lnc-JAKMIP2-2:1	-1.86982	4.294525	-7.59218	0.000394	0.04787	0.794927
ENST00000656790.1	-1.64831	4.902792	-7.58866	0.000395	0.047919	0.792581
MSTRG.40328.2	1.845453	6.340686	7.58397	0.000396	0.048014	0.789453
lnc-GLT1D1-2:3	1.643747	6.238476	7.578862	0.000398	0.048122	0.786044
lnc-ROCK1-2:2	-1.70026	2.201615	-7.57526	0.000399	0.048123	0.783638
NONHSAT210220.1	1.317822	4.350464	7.570409	0.0004	0.048195	0.780396
MSTRG.4395.1	-1.06792	3.14937	-7.56868	0.0004	0.048195	0.779239
lnc-CD72-1:1	-1.14431	3.740135	-7.56776	0.000401	0.048195	0.778625
NONHSAT173809.1	1.3542	8.068451	7.557232	0.000403	0.048223	0.771579
T296710	2.028247	2.642023	7.556684	0.000404	0.048223	0.771212
MSTRG.17656.1	-1.59152	2.13307	-7.55635	0.000404	0.048223	0.770986
MSTRG.5674.1	1.709444	3.987488	7.5563	0.000404	0.048223	0.770954
lnc-CCT6B-6:2	1.187488	1.706344	7.554605	0.000404	0.048223	0.769819
MSTRG.61533.1	2.387116	2.589011	7.549999	0.000405	0.04826	0.766732
MSTRG.38875.1	2.097012	4.923783	7.541686	0.000408	0.048382	0.761154
ENST00000664386.1	1.181993	1.694458	7.537882	0.000409	0.048382	0.7586
ENST00000621477.1	-1.64978	4.227164	-7.53527	0.000409	0.048382	0.756846
lnc-ARF6-8:1	1.421669	3.711816	7.531399	0.000411	0.048382	0.754244
NONHSAT188590.1	1.459217	1.939586	7.528449	0.000411	0.048382	0.75226
ENST00000563624.1	-1.44632	4.975431	-7.52696	0.000412	0.048382	0.751258
NONHSAT212456.1	-1.63404	2.084956	-7.52105	0.000413	0.048382	0.747281
lnc-OLFM4-12:1	-1.6682	1.953027	-7.51674	0.000415	0.048382	0.744374
lnc-FAM81B-1:1	1.75104	5.205513	7.513796	0.000415	0.048382	0.742393
lnc-BCL7C-1:2	1.135469	7.577693	7.511538	0.000416	0.048382	0.740871

NONHSAT180081.1	1.199008	6.206769	7.507493	0.000417	0.048382	0.738143
ENST00000644259.1	2.75763	2.905993	7.503284	0.000418	0.048382	0.735303
MSTRG.2407.1	-1.14777	6.581589	-7.499	0.00042	0.048382	0.732407
NR_125909	1.04055	2.613547	7.497858	0.00042	0.048382	0.731638
lnc-RPS12-6:1	-1.68483	2.934245	-7.49575	0.000421	0.048382	0.730217
NONHSAT148297.1	1.390852	1.790825	7.492465	0.000422	0.048382	0.727993
lnc-PRSS16-1:1	-1.06583	5.23094	-7.48264	0.000424	0.048382	0.721348
MSTRG.47492.1	1.099752	5.337912	7.482161	0.000425	0.048382	0.721021
lnc-AMELY-24:1	1.113163	4.139292	7.481195	0.000425	0.048382	0.720367
NONHSAT223809.1	1.048576	5.295182	7.480911	0.000425	0.048382	0.720174
lnc-P3H2-7:1	1.687028	1.880539	7.480601	0.000425	0.048382	0.719965
NONHSAT160087.1	-1.81239	5.51807	-7.48054	0.000425	0.048382	0.719926
T108433	1.684823	2.352857	7.480489	0.000425	0.048382	0.719889
NONHSAT186528.1	1.699649	1.942952	7.474162	0.000427	0.048382	0.715601
MSTRG.9065.1	1.307484	2.035964	7.474061	0.000427	0.048382	0.715533
ENST00000558175.1	-1.09664	3.435739	-7.47013	0.000428	0.048382	0.712864
MSTRG.56561.1	-1.01024	5.983419	-7.46897	0.000428	0.048382	0.712082
NONHSAT169054.1	1.337024	4.970651	7.467522	0.000429	0.048382	0.711097
lnc-TMEM170B-5:3	-1.06269	5.035224	-7.46681	0.000429	0.048382	0.710613
MSTRG.44418.1	1.163425	1.625151	7.465925	0.000429	0.048382	0.710014
NONHSAT199468.1	-1.12418	5.646777	-7.46513	0.00043	0.048382	0.709471
NONHSAT205252.1	1.822943	1.933818	7.460635	0.000431	0.048382	0.706421
ENST00000607625.1	-1.10127	6.52185	-7.4546	0.000433	0.048382	0.702319
MSTRG.35113.1	1.824848	3.745871	7.451513	0.000434	0.048382	0.700221
NONHSAT203890.1	1.638796	5.711262	7.448182	0.000435	0.048382	0.697955
NONHSAT153878.1	1.275711	5.091232	7.440858	0.000437	0.048382	0.692968
lnc-GAPVD1-6:1	-1.19659	6.626308	-7.43515	0.000438	0.048382	0.689081
lnc-GCM1-2:1	1.685706	1.900984	7.432638	0.000439	0.048382	0.687365
ENST00000668724.1	1.812845	4.365594	7.431392	0.00044	0.048382	0.686515
lnc-CSN2-1:1	2.455854	3.089587	7.431147	0.00044	0.048382	0.686348
NONHSAT164348.1	1.455537	1.808004	7.429831	0.00044	0.048382	0.685449
NR_002981	-1.26552	7.903821	-7.42674	0.000441	0.048382	0.683338
NONHSAT164531.1	2.294935	2.493743	7.424097	0.000442	0.048382	0.681536
NONHSAT217822.1	1.362711	4.698202	7.42358	0.000442	0.048382	0.681182
T096031	1.178175	4.090819	7.420384	0.000443	0.048383	0.678999
NONHSAT223796.1	1.462409	2.450013	7.412731	0.000445	0.048437	0.673767
MSTRG.29811.1	-1.54568	2.451454	-7.41249	0.000445	0.048437	0.673604
MSTRG.42434.19	1.966932	2.416744	7.395714	0.00045	0.048829	0.662112
NONHSAT201516.1	2.592659	4.728291	7.394381	0.000451	0.048829	0.661198
NONHSAT174350.1	1.538238	5.715617	7.384186	0.000454	0.048969	0.6542
MSTRG.49244.1	-1.91892	3.141794	-7.37377	0.000457	0.049126	0.647037
MSTRG.17010.45	-1.86929	2.937116	-7.37038	0.000458	0.049126	0.644708
NONHSAT153862.1	1.095191	1.785067	7.368762	0.000459	0.049126	0.643593
NONHSAT179831.1	-1.28296	3.939829	-7.36722	0.000459	0.049126	0.642533
MSTRG.54710.1	1.961488	2.064042	7.359736	0.000462	0.04913	0.637374
MSTRG.42584.1	2.327437	2.406724	7.358197	0.000462	0.04913	0.636313
NONHSAT169815.1	-1.05724	6.879252	-7.3571	0.000463	0.04913	0.635554
T262630	1.087302	5.810605	7.354978	0.000463	0.04913	0.634093

NONHSAT154432.1	1.775016	4.09937	7.35335	0.000464	0.04913	0.63297
MSTRG.35536.1	-1.50207	4.552332	-7.35299	0.000464	0.04913	0.632721
NONHSAT187793.1	1.082568	5.032359	7.349205	0.000465	0.04913	0.630109
NR_003521	-1.12621	4.64655	-7.34499	0.000467	0.04913	0.6272
NONHSAT205166.1	2.031891	2.091617	7.344921	0.000467	0.04913	0.627151
NONHSAT188190.1	1.001598	5.81271	7.344669	0.000467	0.04913	0.626976
NONHSAT169886.1	1.286891	1.724493	7.338907	0.000469	0.049208	0.622994
MSTRG.25437.1	1.331533	2.101717	7.335969	0.00047	0.049208	0.620961
MSTRG.51109.1	1.293617	10.07664	7.335603	0.00047	0.049208	0.620708
T066611	1.123582	5.292977	7.333459	0.00047	0.049208	0.619225
lnc-GUSB-1:1	-1.12069	3.59462	-7.33312	0.00047	0.049208	0.618993
NONHSAT171732.1	1.493633	1.765077	7.332813	0.000471	0.049208	0.618778
T200023	-1.52665	2.162433	-7.32649	0.000473	0.049225	0.614396
ENST00000620192.1	-1.00275	5.926493	-7.32644	0.000473	0.049225	0.614366
MSTRG.29239.7	-1.93464	2.498884	-7.32602	0.000473	0.049225	0.614072
MSTRG.52104.1	-1.26264	2.038448	-7.32511	0.000473	0.049225	0.61344
lnc-RNF20-5:1	-2.19789	2.53795	-7.31734	0.000476	0.049441	0.608054
ENST00000431376.1	-1.2171	3.905671	-7.31164	0.000478	0.049474	0.6041
NONHSAT199381.1	-1.50415	1.812852	-7.30839	0.000479	0.049474	0.601844
NR_026874	1.263914	5.654085	7.305781	0.000479	0.049474	0.600031
NONHSAT213855.1	2.538166	2.53851	7.303122	0.00048	0.049474	0.598184
lnc-SLC3A1-3:1	-1.05452	6.328057	-7.29837	0.000482	0.049576	0.594877
ENST00000572573.2	1.029584	1.589019	7.293103	0.000484	0.049576	0.591214
lnc-ZNRF4-3:1	1.775096	4.983931	7.290932	0.000484	0.049576	0.589702
NONHSAT181782.1	1.079214	1.632673	7.289504	0.000485	0.049576	0.588707
MSTRG.11604.15	-1.04674	6.809829	-7.28716	0.000486	0.049576	0.587078
ENST00000608871.2	-1.21359	6.720447	-7.28485	0.000487	0.049576	0.585463
LINC00920:1	-1.57495	4.67159	-7.2842	0.000487	0.049576	0.58501
NONHSAT218360.1	1.014109	5.653701	7.28371	0.000487	0.049576	0.58467
lnc-EPSTI1-5:2	2.022318	2.385086	7.2827	0.000487	0.049576	0.583966
NR_105061	1.545064	5.236231	7.278798	0.000489	0.049615	0.581245
ENST00000581489.1	-1.48636	2.042452	-7.27869	0.000489	0.049615	0.581172
NONHSAT209967.1	1.095251	3.26298	7.272334	0.000491	0.049738	0.576733
NR_002731	1.381948	5.66454	7.270741	0.000491	0.049738	0.575621
NONHSAT174759.1	-1.03696	7.968947	-7.26676	0.000493	0.049738	0.572841
ENST00000606330.1	1.68118	4.431784	7.26495	0.000493	0.049738	0.571574
NONHSAT153890.1	1.615731	6.554441	7.260823	0.000495	0.049738	0.568689
lnc-QDPR-5:1	-1.80443	4.5539	-7.25197	0.000498	0.049948	0.562496
NONHSAT201275.1	1.5321	1.968579	7.246577	0.0005	0.050088	0.558714
NONHSAT201948.1	1.34936	4.669789	7.243518	0.000501	0.050113	0.556569
MSTRG.27104.1	-1.83783	2.357507	-7.23481	0.000504	0.050113	0.550456
NONHSAT154326.1	-1.45044	1.857006	-7.23203	0.000505	0.050113	0.548504
lnc-SHARPIN-4:5	-1.47229	3.333184	-7.23138	0.000505	0.050113	0.54805
lnc-KCNJ6-16:1	-1.27225	5.051044	-7.23037	0.000505	0.050113	0.547344
lnc-RAP1B-6:1	-1.22091	7.746886	-7.22382	0.000508	0.05012	0.542734
MSTRG.7871.1	-1.03029	5.381579	-7.22351	0.000508	0.05012	0.542521
NONHSAT161585.1	1.211149	5.718715	7.223211	0.000508	0.05012	0.542308
lnc-ARRDC3-10:1	-1.04351	6.161979	-7.21319	0.000512	0.050318	0.535253

NONHSAT172454.1	1.731996	2.215067	7.212136	0.000512	0.050318	0.534512
ENST00000550941.1	1.803268	4.364067	7.21061	0.000513	0.050318	0.533437
lnc-SLC23A3-3:1	-1.7374	2.300656	-7.20929	0.000513	0.050318	0.532509
ENST00000659614.1	1.199438	4.272205	7.204435	0.000515	0.050349	0.529083
NONHSAT223229.1	1.020408	2.09906	7.201133	0.000516	0.050349	0.526754
lnc-ARHGAP29-2:1	-1.22314	4.543609	-7.20072	0.000516	0.050349	0.526463
NONHSAT208028.1	1.102651	1.627117	7.198928	0.000517	0.050349	0.525198
lnc-VSTM5-3:1	1.11245	1.641631	7.19879	0.000517	0.050349	0.5251
lnc-CLEC1B-1:2	1.617418	5.533193	7.189602	0.00052	0.050534	0.518611
NONHSAT161255.1	1.742363	2.205395	7.189488	0.00052	0.050534	0.51853
NONHSAT203155.1	-1.07954	1.754892	-7.1824	0.000523	0.050615	0.513518
NONHSAT199763.1	1.550588	1.809601	7.181458	0.000523	0.050615	0.512851
NONHSAT170235.1	-1.32667	5.458908	-7.18102	0.000523	0.050615	0.512542
ENST00000642132.1	1.517942	1.92025	7.17947	0.000524	0.050615	0.511444
NONHSAT169336.1	-1.87047	2.037719	-7.17727	0.000525	0.050624	0.509887
NONHSAT185364.1	1.349739	5.065978	7.173379	0.000526	0.050624	0.50713
MSTRG.29057.151	1.447151	4.894599	7.173114	0.000526	0.050624	0.506942
MSTRG.23836.4	-1.70272	3.771436	-7.16927	0.000528	0.050624	0.504218
MSTRG.56680.1	-1.0652	2.280312	-7.16318	0.00053	0.050624	0.499899
lnc-ZNF680-15:1	1.122324	1.801769	7.162871	0.00053	0.050624	0.499679
NONHSAT149538.1	1.803918	2.333956	7.161612	0.000531	0.050624	0.498785
lnc-ADSL-2:1	-2.12162	3.102262	-7.16132	0.000531	0.050624	0.498579
lnc-ADAM2-3:1	1.647422	3.629175	7.156015	0.000533	0.050734	0.494811
NONHSAT183140.1	1.547742	4.194775	7.152905	0.000534	0.050744	0.492602
ENST00000504175.2	-1.14264	4.502033	-7.15265	0.000534	0.050744	0.492418
lnc-SUMF2-3:1	1.718712	2.285162	7.148477	0.000536	0.050847	0.489454
NONHSAT205360.1	1.450955	3.287064	7.143346	0.000537	0.050946	0.485804
NONHSAT202963.1	1.020389	6.758665	7.142225	0.000538	0.050946	0.485006
NONHSAT150648.1	-1.30235	2.610596	-7.13744	0.00054	0.050946	0.481602
ENST00000439451.1	1.491799	1.968585	7.132915	0.000541	0.051024	0.478375
lnc-ZPBP-8:1	-1.01463	4.04844	-7.12894	0.000543	0.051024	0.475537
MSTRG.8974.1	1.832343	2.246605	7.128451	0.000543	0.051024	0.475192
NONHSAT194239.1	1.023004	11.59057	7.127765	0.000543	0.051024	0.474702
lnc-FAM72D-3:2	-1.01546	5.522374	-7.11123	0.00055	0.05128	0.462894
MSTRG.26626.1	-1.15773	5.707534	-7.1054	0.000552	0.051346	0.458721
lnc-RTCA-2:1	1.982181	2.058289	7.101286	0.000554	0.051346	0.455778
SAMMSON:10	1.583095	2.687663	7.100825	0.000554	0.051346	0.455448
NONHSAT187555.1	2.226116	5.146787	7.096653	0.000556	0.051359	0.452459
NONHSAT210239.1	1.30779	7.855635	7.096534	0.000556	0.051359	0.452374
NONHSAT207192.1	-1.84164	2.174392	-7.08999	0.000558	0.051443	0.447679
NONHSAT211691.1	1.101673	3.389232	7.08929	0.000559	0.051443	0.44718
NONHSAT187835.1	1.334007	3.928747	7.089177	0.000559	0.051443	0.447099
MSTRG.65701.1	1.136418	3.155322	7.077275	0.000563	0.051443	0.438552
lnc-HMGCLL1-2:1	2.360118	3.557255	7.076678	0.000564	0.051443	0.438123
lnc-CENPP-14:1	-1.02115	6.011663	-7.07258	0.000565	0.051443	0.435175
NONHSAT197562.1	1.073761	4.656128	7.071674	0.000566	0.051443	0.434526
NONHSAT211250.1	1.119946	4.196865	7.070099	0.000566	0.051443	0.433393
NONHSAT150854.1	1.470812	2.022946	7.067693	0.000567	0.051443	0.431662

NONHSAT214841.1	1.799253	2.653933	7.067543	0.000567	0.051443	0.431554
lnc-LY86-4:2	1.328313	1.963401	7.067146	0.000568	0.051443	0.431268
lnc-NXPE3-1:1	1.200954	5.677776	7.065316	0.000568	0.051443	0.429951
MSTRG.26513.93	1.592246	4.637459	7.064847	0.000568	0.051443	0.429613
T314099	1.266497	1.754677	7.063992	0.000569	0.051443	0.428998
MSTRG.13154.1	1.421656	4.1987	7.063953	0.000569	0.051443	0.428969
lnc-CAT-1:1	-1.33807	4.099285	-7.06301	0.000569	0.051443	0.428289
NONHSAT205640.1	-1.2556	2.392792	-7.05577	0.000572	0.051599	0.423072
NONHSAT214741.1	-1.41061	1.8034	-7.05338	0.000573	0.051599	0.421348
T241186	1.064083	4.321655	7.051843	0.000574	0.05161	0.420241
lnc-CTXND2-5:2	1.060625	4.4103	7.049264	0.000575	0.051642	0.418381
NONHSAT221951.1	1.171468	7.799437	7.046223	0.000576	0.051642	0.416186
NONHSAT177069.1	3.058342	3.417542	7.035219	0.000581	0.051642	0.408235
NONHSAT206364.1	-1.12512	4.576794	-7.03437	0.000581	0.051642	0.40762
NONHSAT175033.1	-1.42561	4.341286	-7.03349	0.000581	0.051642	0.406988
lnc-PDIA4-3:1	-1.41567	4.025307	-7.03303	0.000582	0.051642	0.406648
ENST00000660627.1	-1.50489	4.285831	-7.03184	0.000582	0.051642	0.405793
lnc-WAC-1:1	1.042184	5.229757	7.031331	0.000582	0.051642	0.405422
T309027	1.494964	5.875645	7.023858	0.000585	0.051642	0.400012
lnc-DPYS-1:2	1.543598	3.548938	7.016216	0.000589	0.051642	0.394473
lnc-ANXA2R-6:1	2.810563	5.217614	7.00847	0.000592	0.051642	0.388853
NONHSAT180753.1	1.441274	6.108274	7.006811	0.000593	0.051642	0.387648
lnc-ADGRD2-5:1	-1.67319	2.504339	-7.00448	0.000594	0.051642	0.385956
lnc-DCDC1-4:1	-1.0771	3.141013	-7.00292	0.000594	0.051642	0.384821
lnc-BST2-1:1	-1.07614	6.769907	-7.0023	0.000595	0.051642	0.38437
MSTRG.66910.17	-1.19331	6.128242	-7.00118	0.000595	0.051642	0.383555
ENST00000599498.2	-1.79347	2.465943	-7.00112	0.000595	0.051642	0.383516
ENST00000548900.1	-1.29169	1.876276	-7.00057	0.000595	0.051642	0.383113
lnc-E2F6-2:1	-1.79551	3.88886	-7.00001	0.000596	0.051642	0.382706
lnc-PXDC1-12:2	-1.47074	2.166642	-6.99857	0.000596	0.051642	0.381658
NONHSAT165093.1	1.044627	1.618701	6.998565	0.000596	0.051642	0.381657
T152287	-1.34681	4.976351	-6.99739	0.000597	0.051642	0.380802
ENST00000648410.1	-1.85191	2.015273	-6.99705	0.000597	0.051642	0.380554
lnc-HSDL2-5:1	-1.12279	3.887029	-6.99587	0.000597	0.051642	0.379695
NONHSAT177705.1	-1.18329	1.65286	-6.99553	0.000598	0.051642	0.379453
lnc-COL6A5-3:1	1.214724	1.793432	6.993505	0.000598	0.051642	0.377976
ENST00000540392.1	-1.50501	2.142487	-6.99266	0.000599	0.051642	0.377363
MSTRG.57936.1	2.022027	2.041376	6.991817	0.000599	0.051642	0.376748
lnc-RXYLT1-3:2	-1.36973	3.273257	-6.99145	0.000599	0.051642	0.376481
T372787	1.332232	3.972329	6.990373	0.0006	0.051642	0.375697
MSTRG.72001.13	-1.09354	6.321996	-6.98633	0.000602	0.051747	0.37275
lnc-SCHIP1-2:1	-1.21306	4.933657	-6.97802	0.000605	0.051929	0.366697
MSTRG.24168.1	1.275352	4.30627	6.976741	0.000606	0.051932	0.365763
lnc-OR13J1-2:1	-1.18106	4.916485	-6.97329	0.000607	0.051974	0.363245
ENST00000670874.1	1.362182	2.02073	6.970188	0.000609	0.051985	0.360981
MSTRG.44086.1	1.683851	5.590312	6.968482	0.000609	0.051985	0.359735
lnc-ZNF705D-5:1	-1.09936	1.809489	-6.96832	0.000609	0.051985	0.359619
lnc-PTPRZ1-11:1	1.117664	4.767447	6.958654	0.000614	0.052174	0.352552

NONHSAT219644.1	1.252183	4.365415	6.956795	0.000615	0.0522	0.351192
MSTRG.64924.1	1.373961	5.906594	6.952483	0.000616	0.05225	0.348037
lnc-TMEM161B-10:3	-1.09857	5.078432	-6.94753	0.000619	0.05225	0.344409
lnc-TSPY4-8:1	1.743163	2.472831	6.947156	0.000619	0.05225	0.344135
NONHSAT159699.1	1.890709	2.076876	6.945522	0.00062	0.05225	0.342938
ENST00000609279.1	-1.73994	2.241633	-6.94394	0.00062	0.05225	0.341779
ENST00000567428.2	-1.18486	4.071682	-6.93789	0.000623	0.052437	0.337339
NONHSAT185502.1	2.626794	2.937134	6.935468	0.000624	0.052485	0.335564
lnc-STK25-2:1	1.200153	8.609427	6.918368	0.000632	0.052878	0.322998
NONHSAT218859.1	1.619928	2.174116	6.915813	0.000633	0.052889	0.321118
NONHSAT178852.1	1.085482	7.374425	6.912626	0.000635	0.052968	0.318772
NONHSAT193328.1	-1.27363	1.723518	-6.90972	0.000636	0.053015	0.316633
NONHSAT199844.1	1.347431	3.909066	6.909149	0.000636	0.053015	0.31621
NONHSAT206387.1	1.040113	5.550902	6.903096	0.000639	0.053119	0.311748
lnc-CAMSAP2-5:1	-1.2477	5.943498	-6.89867	0.000641	0.053119	0.308481
NR_026993	-1.57829	3.966637	-6.89779	0.000641	0.053119	0.307832
lnc-NEIL3-1:1	2.170515	2.17812	6.895686	0.000642	0.053119	0.306281
lnc-SLC25A10-4:1	-1.54763	5.135234	-6.89518	0.000643	0.053119	0.305907
NONHSAT157966.1	-1.68033	2.143169	-6.89174	0.000644	0.053119	0.303364
NONHSAT216241.1	1.026503	1.530808	6.891256	0.000645	0.053119	0.303009
NONHSAT202591.1	-1.4924	2.319229	-6.89084	0.000645	0.053119	0.302701
lnc-ID2-8:2	1.202195	1.692554	6.888704	0.000646	0.053119	0.301123
MSTRG.27546.1	-1.56257	2.456579	-6.88153	0.000649	0.053119	0.295817
ENST00000439027.1	1.080165	2.563786	6.881096	0.000649	0.053119	0.295497
NONHSAT186733.1	2.338527	6.529731	6.879176	0.00065	0.053119	0.294077
PCAT6:6	1.293404	5.673987	6.877179	0.000651	0.053119	0.292599
lnc-RABEP1-5:1	-1.55351	3.433793	-6.87524	0.000652	0.053119	0.291164
lnc-ATP12A-1:11	1.473952	2.216177	6.87452	0.000653	0.053119	0.290629
NONHSAT212341.1	1.249762	4.419051	6.87358	0.000653	0.053119	0.289933
lnc-ELF1-1:2	-1.49002	3.403996	-6.87173	0.000654	0.053119	0.288563
NONHSAT187985.1	-1.65379	2.141417	-6.87165	0.000654	0.053119	0.288507
NONHSAT214601.1	1.347195	4.909687	6.868894	0.000655	0.053119	0.286461
NONHSAT168416.1	1.631643	3.871794	6.86838	0.000655	0.053119	0.28608
T224283	2.038292	3.510036	6.866746	0.000656	0.053119	0.284869
NR_024046	-1.62609	5.239959	-6.8649	0.000657	0.053119	0.283499
NONHSAT191672.1	-1.74832	2.784791	-6.86477	0.000657	0.053119	0.283404
ENST00000589715.1	-1.12936	4.305044	-6.86424	0.000657	0.053119	0.283007
ENST00000581727.1	-2.10627	2.342375	-6.86313	0.000658	0.053119	0.282187
MSTRG.21378.2	1.831572	2.511794	6.849191	0.000665	0.053261	0.271835
lnc-PFKP-27:3	1.029301	4.525896	6.848519	0.000665	0.053261	0.271336
lnc-CDH2-3:1	-1.069	3.337885	-6.84755	0.000666	0.053261	0.270613
MSTRG.56046.1	1.339078	3.244771	6.845681	0.000666	0.053261	0.269225
MSTRG.60557.86	-1.53627	2.170868	-6.84431	0.000667	0.053261	0.268204
MSTRG.50714.19	-1.46149	4.950789	-6.83816	0.00067	0.053391	0.263627
NONHSAT223747.1	2.972548	3.089457	6.837525	0.00067	0.053391	0.263155
MSTRG.6137.1	-1.09164	4.123294	-6.835	0.000672	0.053391	0.261274
lnc-CRACR2A-5:2	-1.80671	2.188557	-6.83412	0.000672	0.053391	0.260622
ENST00000485055.5	1.033467	4.30421	6.82902	0.000675	0.053391	0.256817

T122536	2.045203	2.103255	6.824778	0.000677	0.053391	0.253653
MSTRG.4524.4	-2.32347	2.726766	-6.8245	0.000677	0.053391	0.253444
NONHSAT206884.1	-1.31803	4.686512	-6.8229	0.000678	0.053391	0.252255
NONHSAT178438.1	2.654847	2.514367	6.821658	0.000678	0.053391	0.251325
NR_039984	1.023797	5.097057	6.821049	0.000679	0.053391	0.25087
MSTRG.39603.1	1.039758	4.131819	6.817637	0.00068	0.053471	0.248322
NONHSAT187660.1	-1.14464	3.499269	-6.81587	0.000681	0.053474	0.247005
MSTRG.20397.1	1.582748	1.849157	6.813311	0.000683	0.053474	0.24509
MSTRG.65759.1	1.843455	2.183886	6.810475	0.000684	0.053474	0.24297
NONHSAT187940.1	1.254438	4.045906	6.808359	0.000685	0.053474	0.241388
ENST00000655803.1	-1.15335	4.444149	-6.80818	0.000685	0.053474	0.241253
lnc-YEATS4-3:1	-1.19871	4.276811	-6.80789	0.000685	0.053474	0.241036
lnc-NEFL-1:2	1.506613	5.270155	6.805573	0.000686	0.053474	0.239304
lnc-TXNDC5-5:1	1.275044	11.53744	6.804924	0.000687	0.053474	0.238817
NONHSAT199064.1	1.219181	4.834977	6.803797	0.000687	0.053474	0.237974
MSTRG.67360.12	-1.27232	2.091389	-6.80287	0.000688	0.053474	0.237278
T255263	-1.33894	4.562679	-6.80135	0.000689	0.05348	0.236146
lnc-PEPD-7:1	1.408847	1.792607	6.791825	0.000693	0.053548	0.229005
lnc-ODC1-2:4	-1.52097	5.900565	-6.79067	0.000694	0.053548	0.228143
MSTRG.15382.1	1.663488	5.064365	6.788078	0.000695	0.053548	0.226195
NONHSAT176879.1	1.429067	3.604117	6.786452	0.000696	0.053548	0.224976
MSTRG.33740.1	1.057464	4.125353	6.785453	0.000697	0.053548	0.224226
NONHSAT187158.1	1.005181	1.97843	6.784223	0.000697	0.053556	0.223302
T321579	1.126943	5.19967	6.783053	0.000698	0.053562	0.222424
NONHSAT178902.1	1.400589	1.906829	6.780269	0.000699	0.053568	0.220334
MSTRG.60769.1	-1.38743	5.216621	-6.77983	0.0007	0.053568	0.220005
MSTRG.37312.1	1.242239	5.366247	6.777123	0.000701	0.05363	0.21797
MSTRG.32785.1	-1.7319	2.255614	-6.77236	0.000704	0.05363	0.214392
lnc-KIF18A-9:1	-1.01593	5.352756	-6.77158	0.000704	0.05363	0.213801
ENST00000616950.1	1.437676	6.320191	6.768094	0.000706	0.05363	0.21118
lnc-DCBLD1-2:1	-1.32838	4.441224	-6.76713	0.000706	0.05363	0.210459
lnc-PTCHD4-2:1	-1.12385	7.01468	-6.76616	0.000707	0.05363	0.209724
NONHSAT153767.1	-1.24384	4.559856	-6.7644	0.000708	0.05363	0.208401
MSTRG.52731.1	-1.93356	2.298429	-6.76403	0.000708	0.05363	0.208121
NONHSAT178466.1	-1.6313	2.044676	-6.75975	0.00071	0.05363	0.204901
MSTRG.63640.1	1.062664	3.693942	6.757169	0.000712	0.05363	0.202953
NONHSAT158334.1	-1.66718	4.652809	-6.75644	0.000712	0.05363	0.2024
MSTRG.2660.1	-1.61732	5.30469	-6.75624	0.000712	0.05363	0.202256
ENST00000541885.1	2.720091	3.105184	6.755937	0.000712	0.05363	0.202024
NONHSAT200732.1	2.142039	2.224481	6.753963	0.000713	0.05363	0.200536
NONHSAT152765.1	1.492773	3.021596	6.753482	0.000713	0.05363	0.200174
lnc-PRR4-1:13	-1.06064	5.483623	-6.75	0.000715	0.05363	0.197545
lnc-VEZF1-1:7	-1.0402	5.328271	-6.74948	0.000716	0.05363	0.197151
MSTRG.25435.1	-1.25445	3.593991	-6.74875	0.000716	0.05363	0.1966
NONHSAT210404.1	1.184477	3.167359	6.747944	0.000716	0.05363	0.195995
NONHSAT166826.1	1.937662	3.564869	6.743344	0.000719	0.053664	0.192522
NONHSAT149286.1	1.419922	3.147758	6.742697	0.000719	0.053664	0.192034
NONHSAT213782.1	1.415373	1.797475	6.741259	0.00072	0.053664	0.190947

NONHSAT217845.1	1.321509	1.691077	6.737889	0.000722	0.053683	0.1884
lnc-ZNF131-11:1	-1.1909	3.190999	-6.73768	0.000722	0.053683	0.188242
lnc-EMCN-2:1	-1.97284	5.245069	-6.73382	0.000724	0.053714	0.185321
NONHSAT158061.1	-1.43253	2.334148	-6.73303	0.000724	0.053714	0.184728
ENST00000668181.1	1.243656	4.843622	6.732715	0.000725	0.053714	0.184488
NONHSAT208810.1	1.684524	1.887201	6.7325	0.000725	0.053714	0.184325
lnc-VKORC1L1-2:1	-1.14961	3.356565	-6.72675	0.000728	0.053883	0.179977
lnc-SLITRK6-28:2	1.034359	4.131783	6.716209	0.000734	0.054016	0.171987
lnc-FCN1-5:1	1.922771	2.287586	6.71456	0.000734	0.054016	0.170736
lnc-APOD-6:1	-1.02005	8.523607	-6.71445	0.000735	0.054016	0.17065
lnc-ID3-4:1	1.159228	3.348641	6.713447	0.000735	0.054016	0.169892
T182488	-1.45713	2.004525	-6.71327	0.000735	0.054016	0.169756
ENST00000502437.1	1.188387	4.478531	6.712319	0.000736	0.054016	0.169036
NONHSAT217478.1	2.095274	2.334527	6.710031	0.000737	0.054016	0.1673
lnc-TNFSF15-3:1	1.555714	3.116672	6.709801	0.000737	0.054016	0.167125
lnc-KHNYN-8:1	1.037217	5.080677	6.707337	0.000738	0.054016	0.165254
lnc-TAF5L-4:2	-1.42622	3.324013	-6.70684	0.000739	0.054016	0.164879
NONHSAT187820.1	-1.33091	4.834157	-6.70192	0.000741	0.054109	0.161141
ENST00000439455.1	-1.77219	3.670341	-6.70164	0.000742	0.054109	0.160924
NONHSAT164347.1	1.020988	5.489494	6.697232	0.000744	0.054137	0.157575
MSTRG.8860.2	2.761215	3.598722	6.690873	0.000748	0.054181	0.152737
NONHSAT160345.1	1.073876	5.022499	6.686816	0.00075	0.054202	0.149648
MSTRG.51939.1	-1.08095	1.635286	-6.68507	0.000751	0.054202	0.14832
lnc-PTGS2-10:1	1.351725	1.73675	6.684868	0.000751	0.054202	0.148164
MSTRG.29862.1	-1.03826	6.216453	-6.68278	0.000752	0.054202	0.146574
NONHSAT171285.1	1.60055	6.924888	6.681851	0.000753	0.054202	0.145865
lnc-ANKRD29-2:1	1.011144	9.367901	6.681713	0.000753	0.054202	0.145759
MSTRG.50049.1	1.264967	4.548924	6.680526	0.000753	0.054202	0.144854
MSTRG.59826.1	-1.03837	6.194511	-6.68017	0.000754	0.054202	0.144582
NONHSAT164446.1	1.400385	1.877418	6.677829	0.000755	0.054259	0.142798
NONHSAT170161.1	-1.09004	4.734948	-6.6763	0.000756	0.054282	0.141635
T187936	1.100863	2.073741	6.672022	0.000758	0.054418	0.138367
ENST00000453716.1	1.040063	4.678935	6.66617	0.000762	0.05458	0.133898
lnc-ARHGAP6-2:1	-1.1332	4.154684	-6.66505	0.000762	0.054587	0.133045
lnc-MYLK4-12:1	1.22348	4.559123	6.661482	0.000764	0.054656	0.130315
lnc-ICE2-2:2	-1.16102	5.822646	-6.65638	0.000767	0.054668	0.126412
NONHSAT156588.1	1.166174	4.285892	6.655859	0.000768	0.054668	0.126013
NONHSAT183144.1	1.407131	4.454012	6.655551	0.000768	0.054668	0.125778
lnc-FUCA2-2:1	-1.4419	2.178256	-6.65446	0.000768	0.054674	0.124946
NONHSAT178936.1	-1.31848	1.933587	-6.65102	0.00077	0.054698	0.122311
lnc-SCTR-2:4	-1.47342	3.151907	-6.65097	0.00077	0.054698	0.122274
ENST00000587696.2	-1.38771	2.348364	-6.65013	0.000771	0.054698	0.12163
NONHSAT186416.1	1.279632	5.149463	6.640496	0.000776	0.054781	0.114244
NONHSAT201926.1	1.896664	2.030651	6.637515	0.000778	0.054781	0.111957
NONHSAT167559.1	-1.22561	4.48152	-6.63412	0.00078	0.054781	0.109355
NONHSAT155778.1	1.010395	4.077935	6.633494	0.000781	0.054781	0.10887
NONHSAT197985.1	1.724139	7.755332	6.632606	0.000781	0.054781	0.108189
NONHSAT214222.1	1.313778	1.685011	6.631059	0.000782	0.054781	0.107001

lnc-C6orf201-9:1	1.533055	6.159334	6.62874	0.000783	0.054781	0.10522
NONHSAT198109.1	-1.0069	4.406604	-6.62491	0.000786	0.054781	0.102272
MSTRG.7840.3	-1.39148	4.467517	-6.62491	0.000786	0.054781	0.102272
ENST00000663697.1	1.449022	3.72187	6.624361	0.000786	0.054781	0.101853
ENST00000666655.1	1.94387	2.064459	6.623268	0.000787	0.054781	0.101013
ENST00000449581.1	2.04857	2.546601	6.621814	0.000787	0.054781	0.099895
NFIA-AS1:12	-1.62925	5.001628	-6.62154	0.000788	0.054781	0.099683
lnc-EFCAB6-1:1	1.136132	4.286808	6.620026	0.000789	0.054781	0.09852
T227254	1.954002	2.036085	6.618819	0.000789	0.054781	0.097591
lnc-KLHL18-6:1	-1.00183	5.210678	-6.61879	0.000789	0.054781	0.09757
lnc-SLC46A3-3:1	-1.35028	2.033441	-6.6186	0.000789	0.054781	0.097423
NONHSAT181808.1	1.15926	4.66767	6.613968	0.000792	0.054781	0.093857
ENST00000549291.1	1.970693	2.093355	6.611052	0.000794	0.054781	0.091611
NONHSAT176767.1	2.294787	2.248682	6.610294	0.000794	0.054781	0.091027
NONHSAT177961.1	1.268219	1.666374	6.609095	0.000795	0.054781	0.090103
NONHSAT170875.1	-2.07548	2.440088	-6.60773	0.000796	0.054781	0.089054
lnc-SLCO4C1-5:1	-2.09151	3.220852	-6.60605	0.000797	0.054781	0.087759
NONHSAT217968.1	1.07247	1.631832	6.605827	0.000797	0.054781	0.087584
lnc-CD226-3:4	-1.00056	6.141834	-6.60567	0.000797	0.054781	0.087464
T342810	1.717045	5.146039	6.604995	0.000798	0.054781	0.086942
ENST00000522005.1	-1.23948	2.224534	-6.60378	0.000798	0.054781	0.086003
ENST00000518552.2	-1.82494	3.889278	-6.59872	0.000801	0.054781	0.0821
NONHSAT179061.1	1.502757	5.871137	6.598693	0.000801	0.054781	0.082081
NONHSAT186698.1	1.417368	3.186919	6.597253	0.000802	0.054781	0.08097
NONHSAT166052.1	1.508534	2.348859	6.596571	0.000803	0.054781	0.080443
NONHSAT170083.1	1.284733	1.860433	6.595968	0.000803	0.054781	0.079977
ENST00000585189.1	1.182092	3.913011	6.59457	0.000804	0.054781	0.078898
LINC00602:3	1.116326	4.799168	6.593625	0.000804	0.054781	0.078168
NONHSAT158864.1	-1.13289	5.185595	-6.59156	0.000806	0.054781	0.076575
T116318	1.306986	1.976864	6.590239	0.000807	0.054781	0.075552
lnc-HS3ST5-2:5	1.786382	1.945877	6.589659	0.000807	0.054781	0.075104
NONHSAT197190.1	1.084815	5.437569	6.589322	0.000807	0.054781	0.074843
NONHSAT201928.1	-1.00002	1.60723	-6.58907	0.000807	0.054781	0.074648
ENST00000510433.1	2.722143	2.409348	6.588894	0.000807	0.054781	0.074512
MSTRG.48273.2	1.004731	4.927729	6.585367	0.00081	0.054782	0.071785
lnc-SDHAF4-4:1	-1.34866	4.045462	-6.58334	0.000811	0.05483	0.07022
lnc-BCL7A-3:1	1.963602	5.463733	6.58235	0.000811	0.054834	0.069452
NONHSAT216230.1	-1.63806	4.067893	-6.57818	0.000814	0.054852	0.066221
ENST00000655714.1	-1.09623	5.988291	-6.57769	0.000814	0.054852	0.065843
lnc-ACTR3B-4:1	-1.03772	4.775828	-6.57752	0.000814	0.054852	0.065711
NONHSAT174436.1	2.541795	2.713744	6.576236	0.000815	0.054869	0.064718
lnc-DAP-12:1	1.160958	1.662326	6.573571	0.000817	0.054915	0.062655
NONHSAT186809.1	-1.12224	4.375531	-6.57338	0.000817	0.054915	0.062509
T015742	1.350924	5.633918	6.568315	0.00082	0.055018	0.058581
lnc-ZNF669-1:1	1.890266	2.038316	6.566239	0.000821	0.055036	0.056971
lnc-SYPL1-2:1	-1.26738	4.962142	-6.56101	0.000825	0.055093	0.05291
ENST00000616097.1	-1.20819	3.935909	-6.55451	0.000829	0.055093	0.047866
lnc-NAALADL2-12:1	-1.15482	5.594147	-6.55423	0.000829	0.055093	0.04765

ENST00000554540.1	1.136283	4.396126	6.553906	0.000829	0.055093	0.047397
NONHSAT175396.1	1.014796	5.77309	6.551951	0.00083	0.055093	0.045877
NONHSAT186860.1	-1.68788	2.005136	-6.55088	0.000831	0.055093	0.045046
lnc-UNC5D-5:1	1.22737	4.951275	6.548549	0.000833	0.055093	0.043232
NONHSAT224335.1	1.867507	5.109776	6.546679	0.000834	0.055093	0.041778
NONHSAT205368.1	-1.03795	1.850343	-6.54542	0.000835	0.055093	0.040799
lnc-MKKS-8:1	-1.76475	2.725993	-6.54352	0.000836	0.055093	0.039322
NONHSAT197527.1	-1.52089	2.107934	-6.54339	0.000836	0.055093	0.039219
NONHSAT158009.1	1.479369	3.413146	6.542047	0.000837	0.055093	0.038174
lnc-C14orf180-3:1	1.274113	4.106366	6.538934	0.000839	0.055093	0.03575
ENST00000546770.1	2.148021	4.681394	6.536321	0.00084	0.055093	0.033714
lnc-TAS2R1-12:1	-2.02725	2.928057	-6.5362	0.00084	0.055093	0.03362
MSTRG.22622.1	1.00426	4.743102	6.535931	0.000841	0.055093	0.03341
MSTRG.55402.1	-2.61831	3.663217	-6.53495	0.000841	0.055093	0.032646
LINC01697:42	1.195023	5.256212	6.534576	0.000841	0.055093	0.032354
NONHSAT187230.1	1.86299	2.031271	6.530604	0.000844	0.055093	0.029258
ENST00000527318.1	1.947587	4.577145	6.529837	0.000844	0.055093	0.02866
NONHSAT220581.1	1.718687	2.13276	6.529004	0.000845	0.055093	0.028011
MSTRG.35419.1	1.655683	4.297546	6.527392	0.000846	0.055093	0.026753
NONHSAT149210.1	1.072004	2.381219	6.52446	0.000848	0.055093	0.024465
LINC01762:27	1.358077	4.282526	6.524134	0.000848	0.055093	0.024211
ENST00000583687.1	1.135848	1.876673	6.523451	0.000849	0.055093	0.023677
NONHSAT188541.1	2.786956	3.069121	6.520712	0.00085	0.055093	0.021539
lnc-C15orf41-2:4	1.248226	1.9097	6.517378	0.000853	0.055093	0.018935
lnc-BAIAP2-3:1	-1.10159	4.336739	-6.51708	0.000853	0.055093	0.018703
lnc-BECN2-2:2	1.43737	4.444632	6.514619	0.000854	0.055093	0.016778
ENST00000619354.1	1.08872	5.890739	6.513838	0.000855	0.055093	0.016168
lnc-SAMD12-4:1	-1.05744	6.705736	-6.51347	0.000855	0.055093	0.015883
ENST00000668509.1	1.835316	2.374709	6.512692	0.000856	0.055093	0.015272
NONHSAT221485.1	1.05201	7.911935	6.502238	0.000863	0.055438	0.007092
NONHSAT211123.1	-1.04845	1.608344	-6.50205	0.000863	0.055438	0.006946
MSTRG.49273.1	1.370734	1.774377	6.497471	0.000866	0.0555	0.003357
ENST00000503307.2	1.180848	3.448053	6.497267	0.000866	0.0555	0.003198
MSTRG.32943.1	-1.13688	5.184215	-6.4957	0.000867	0.05551	0.001972
NONHSAT169246.1	1.094579	1.922344	6.494159	0.000868	0.05551	0.000762
lnc-SLC2A12-11:1	-1.13985	3.061055	-6.49371	0.000868	0.05551	0.000413
ENST00000608730.1	-1.10676	1.806507	-6.49233	0.000869	0.055528	-0.00067
NONHSAT173571.1	1.16816	1.931094	6.49165	0.00087	0.055528	-0.00121
ENST00000666788.1	1.364138	4.028486	6.484436	0.000874	0.055655	-0.00687
NONHSAT193652.1	-1.36546	1.944949	-6.48296	0.000875	0.055655	-0.00802
lnc-MORC2-3:2	-1.94975	2.770005	-6.48291	0.000875	0.055655	-0.00807
NONHSAT189915.1	1.597591	4.004613	6.48195	0.000876	0.055661	-0.00882
ENST00000505389.1	1.06234	5.53447	6.480528	0.000877	0.055687	-0.00994
lnc-SPATA31A7-8:1	1.211714	5.157779	6.474863	0.000881	0.055826	-0.01439
lnc-FAM50B-4:2	2.362646	2.606462	6.474253	0.000881	0.055826	-0.01487
NONHSAT174480.1	1.149344	3.798772	6.472127	0.000883	0.055846	-0.01654
lnc-STX11-5:1	-1.14748	6.401408	-6.46677	0.000886	0.055946	-0.02076
lnc-NYAP2-7:1	2.866942	3.063267	6.466346	0.000887	0.055946	-0.02109

NONHSAT182983.1	1.526923	2.225626	6.459394	0.000891	0.056176	-0.02657
NONHSAT197714.1	1.910523	3.762886	6.456401	0.000893	0.05624	-0.02893
lnc-ZNF621-1:4	1.442918	5.498228	6.455526	0.000894	0.05624	-0.02962
lnc-RNF212-1:3	1.43705	1.992134	6.45449	0.000895	0.05624	-0.03043
T314711	1.997123	2.69066	6.452631	0.000896	0.05624	-0.0319
T047559	2.152873	2.146694	6.451893	0.000897	0.05624	-0.03248
MSTRG.47530.1	1.153599	4.540557	6.450892	0.000897	0.05624	-0.03327
MSTRG.60219.1	1.404361	4.693838	6.450645	0.000897	0.05624	-0.03347
NONHSAT214360.1	-1.24802	1.772018	-6.44868	0.000899	0.056255	-0.03502
NONHSAT164281.1	1.070408	4.097036	6.443928	0.000902	0.056369	-0.03877
lnc-HOXC13-1:2	-2.14629	2.409805	-6.44364	0.000902	0.056369	-0.039
NONHSAT198401.1	-2.14645	2.319862	-6.44132	0.000904	0.056399	-0.04083
NONHSAT158984.1	1.394533	3.632592	6.441312	0.000904	0.056399	-0.04084
ENST00000506902.1	2.09783	2.456047	6.427525	0.000914	0.056549	-0.05175
lnc-FAM172A-3:4	-1.83634	3.928542	-6.42141	0.000918	0.056549	-0.0566
MSTRG.45444.1	-1.64941	3.045636	-6.41802	0.00092	0.056549	-0.05928
NONHSAT193396.1	1.17561	3.803309	6.417664	0.000921	0.056549	-0.05956
lnc-NENF-4:2	-1.12908	2.430596	-6.41524	0.000922	0.056549	-0.06149
lnc-OCIAD2-5:1	-1.17571	1.752612	-6.41486	0.000923	0.056549	-0.06178
ENST00000659392.1	1.693528	2.337192	6.414111	0.000923	0.056549	-0.06238
NONHSAT210605.1	1.726924	4.070195	6.412541	0.000924	0.056549	-0.06363
NONHSAT196990.1	1.319005	1.806448	6.41227	0.000924	0.056549	-0.06384
NONHSAT201449.1	1.43797	5.431103	6.410267	0.000926	0.056549	-0.06543
NONHSAT204524.1	1.433	3.874452	6.406613	0.000929	0.056549	-0.06834
T120764	1.006853	3.271918	6.406238	0.000929	0.056549	-0.06863
lnc-APPBP2-2:1	-1.14551	4.723553	-6.40562	0.000929	0.056549	-0.06913
NONHSAT210357.1	1.828999	4.324494	6.405167	0.00093	0.056549	-0.06948
T171443	-1.60813	3.424917	-6.40062	0.000933	0.056664	-0.0731
NONHSAT172139.1	1.314058	4.488959	6.397069	0.000935	0.056721	-0.07592
ENST00000530479.1	1.074681	6.848581	6.393094	0.000938	0.056721	-0.07909
lnc-HMGN3-6:1	-1.07345	4.483235	-6.39229	0.000939	0.056721	-0.07973
ENST00000617191.1	1.041133	7.538551	6.390579	0.00094	0.056721	-0.08109
NONHSAT158174.1	-1.62535	4.473712	-6.39038	0.00094	0.056721	-0.08125
ENST00000668682.1	1.650171	2.542094	6.389289	0.000941	0.056721	-0.08212
lnc-HSPB9-1:1	-1.31438	1.821951	-6.38827	0.000942	0.056721	-0.08293
ENST00000552426.1	1.230536	4.214097	6.387467	0.000942	0.056721	-0.08357
NONHSAT158239.1	-1.43725	6.25609	-6.3864	0.000943	0.056721	-0.08442
NONHSAT219249.1	-1.83049	2.275667	-6.38564	0.000944	0.056721	-0.08502
MSTRG.66691.10	-2.27595	2.345974	-6.38394	0.000945	0.056721	-0.08638
ENST00000655747.1	-1.93848	2.50912	-6.38041	0.000948	0.056721	-0.08919
lnc-KLF12-4:1	-1.01717	6.323981	-6.37993	0.000948	0.056721	-0.08958
lnc-SELENOF-11:1	1.822538	1.957716	6.376265	0.000951	0.056755	-0.0925
NONHSAT218976.1	1.585892	4.078422	6.375147	0.000952	0.056771	-0.09339
NONHSAT157886.1	2.252661	2.552681	6.371961	0.000954	0.056811	-0.09594
NR_026938	-1.57805	4.492885	-6.36799	0.000957	0.056853	-0.09911
lnc-FH-4:1	-1.07264	2.110702	-6.36353	0.00096	0.056883	-0.10267
ENST00000594688.1	1.460286	2.603767	6.36224	0.000961	0.056895	-0.10371
ENST00000602530.1	1.52533	4.390674	6.36129	0.000962	0.056895	-0.10447

NONHSAT199820.1	-1.0707	3.034631	-6.35952	0.000963	0.056895	-0.10589
NONHSAT191190.1	1.387608	4.64887	6.356233	0.000966	0.056947	-0.10852
lnc-SENP8-1:2	-1.18955	1.844968	-6.35609	0.000966	0.056947	-0.10863
NONHSAT208897.1	1.075057	1.894969	6.355051	0.000967	0.05696	-0.10946
lnc-ZDHHC11-3:1	1.412879	5.518939	6.351893	0.000969	0.056992	-0.11199
MSTRG.50353.1	1.670747	3.352302	6.351339	0.000969	0.056992	-0.11243
MSTRG.50782.1	1.402451	1.75133	6.348698	0.000971	0.057037	-0.11455
MSTRG.4114.1	-1.10106	4.284426	-6.34721	0.000972	0.057037	-0.11575
lnc-UCK2-5:1	1.115807	2.037321	6.34445	0.000975	0.057037	-0.11796
ENST00000605334.1	-2.15467	3.653454	-6.3432	0.000976	0.057037	-0.11896
ENST00000563503.1	-1.64515	2.471074	-6.34317	0.000976	0.057037	-0.11898
NONHSAT159744.1	-1.63674	2.152736	-6.3421	0.000976	0.057037	-0.11984
NONHSAT178955.1	2.937914	2.998901	6.336252	0.000981	0.057083	-0.12454
NONHSAT172209.1	-1.22114	5.001047	-6.33433	0.000982	0.057083	-0.12608
NONHSAT165025.1	1.06916	5.371152	6.333449	0.000983	0.057083	-0.12679
MSTRG.49058.1	-1.31846	1.872384	-6.33263	0.000984	0.057083	-0.12745
lnc-ZNF574-1:6	1.574853	4.79122	6.332265	0.000984	0.057083	-0.12774
NONHSAT173961.1	1.308836	1.749887	6.330516	0.000985	0.057096	-0.12914
ENST00000668921.1	1.090251	4.305045	6.323005	0.000991	0.057306	-0.13518
lnc-ANKRD46-5:1	2.015787	2.491278	6.321242	0.000992	0.057306	-0.1366
MSTRG.44436.21	2.435663	2.729542	6.320823	0.000993	0.057306	-0.13694
NONHSAT185514.1	1.012563	5.240521	6.318511	0.000995	0.057306	-0.1388
ENST00000616998.1	-1.16847	6.726636	-6.31595	0.000997	0.057306	-0.14086
ENST00000576365.1	-1.05746	8.394088	-6.31421	0.000998	0.057306	-0.14226
lnc-RPS14-2:1	-1.11819	4.8497	-6.31332	0.000999	0.057306	-0.14298
lnc-C10orf143-5:1	1.098581	2.95459	6.312997	0.000999	0.057306	-0.14324
NONHSAT204887.1	1.305736	2.17805	6.312703	0.000999	0.057306	-0.14348
NONHSAT210187.1	1.259286	4.122936	6.311779	0.001	0.057306	-0.14422
NONHSAT223272.1	-1.64894	2.305617	-6.31153	0.001	0.057306	-0.14442
ENST00000501440.1	1.12689	6.235317	6.310614	0.001001	0.057306	-0.14516
NONHSAT190412.1	1.332424	4.395585	6.310261	0.001001	0.057306	-0.14545
MSTRG.68158.1	-1.53609	3.570135	-6.30907	0.001002	0.057306	-0.1464
ENST00000669014.1	-2.3681	3.608127	-6.30671	0.001004	0.05738	-0.14831
lnc-TSPAN8-1:3	1.033489	1.797022	6.302298	0.001007	0.057482	-0.15187
lnc-VWA8-10:1	-1.86959	2.842972	-6.30079	0.001009	0.057507	-0.15309
ENST00000671297.1	-1.85174	3.155137	-6.29867	0.00101	0.057507	-0.1548
lnc-TREM2-1:1	-1.5052	3.386202	-6.29802	0.001011	0.057507	-0.15532
NONHSAT167179.1	1.953247	4.811041	6.295462	0.001013	0.057507	-0.15739
ENST00000521188.1	1.02524	1.624315	6.294812	0.001013	0.057507	-0.15792
ENST00000649504.1	1.641982	1.891874	6.294668	0.001013	0.057507	-0.15803
NONHSAT217657.1	1.542132	4.474413	6.293309	0.001014	0.057507	-0.15913
LINC02251:7	1.129884	5.140899	6.292251	0.001015	0.057507	-0.15999
lnc-PMFBP1-3:6	-1.21348	4.933963	-6.28926	0.001018	0.057507	-0.16241
NONHSAT204158.1	-1.8492	2.718584	-6.28907	0.001018	0.057507	-0.16256
NR_003706	-1.11055	5.897761	-6.28898	0.001018	0.057507	-0.16263
NONHSAT156302.1	1.101318	2.675917	6.288865	0.001018	0.057507	-0.16272
MSTRG.66149.1	-1.22992	1.986102	-6.28811	0.001019	0.057508	-0.16333
lnc-LYPD6-12:1	1.673979	4.68725	6.285322	0.001021	0.057546	-0.16559

NONHSAT218783.1	1.332371	2.578631	6.285171	0.001021	0.057546	-0.16571
NONHSAT216140.1	1.7886	2.418338	6.283988	0.001022	0.057546	-0.16667
ENST00000502344.5	1.089806	1.640146	6.280742	0.001025	0.057585	-0.1693
NONHSAT161580.1	1.172544	7.604423	6.2778	0.001027	0.057623	-0.17168
NONHSAT183450.1	1.791348	2.007285	6.269827	0.001033	0.057672	-0.17814
NONHSAT154599.1	1.661168	3.42241	6.26546	0.001037	0.057672	-0.18169
NONHSAT221751.1	1.327374	1.723969	6.264713	0.001038	0.057672	-0.18229
lnc-ZSCAN30-9:2	1.6063	2.998661	6.261276	0.00104	0.057672	-0.18508
lnc-C2orf73-2:1	-1.70328	2.029669	-6.26084	0.001041	0.057672	-0.18544
MSTRG.47842.1	1.251783	1.650811	6.260484	0.001041	0.057672	-0.18573
NONHSAT161094.1	-1.26201	5.002833	-6.25861	0.001043	0.057672	-0.18725
NONHSAT167388.1	-1.88184	6.107833	-6.25683	0.001044	0.057672	-0.1887
ENST00000452288.1	-1.82001	2.512321	-6.25425	0.001046	0.057672	-0.19079
lnc-HAPLN3-3:1	-1.19136	3.404594	-6.25258	0.001048	0.057672	-0.19215
NONHSAT179300.1	1.209749	5.39619	6.251116	0.001049	0.057672	-0.19334
lnc-FAM149B1-8:1	1.706884	4.39091	6.250753	0.001049	0.057672	-0.19364
lnc-ZXDB-3:1	1.467367	5.691733	6.249007	0.001051	0.057672	-0.19506
MSTRG.1548.1	-1.3559	3.576359	-6.24835	0.001051	0.057672	-0.19559
NONHSAT200992.1	1.26179	2.608701	6.244619	0.001054	0.057771	-0.19863
lnc-HAO2-11:5	1.662999	1.850012	6.242302	0.001056	0.057844	-0.20052
lnc-TMEM200B-2:1	1.644603	1.897597	6.241649	0.001057	0.057844	-0.20105
lnc-CRNKL1-6:1	-1.21227	3.247691	-6.23871	0.001059	0.057916	-0.20344
NONHSAT201694.1	2.826798	2.731189	6.236572	0.001061	0.057939	-0.20519
ENST00000668438.1	1.960504	2.005416	6.236434	0.001061	0.057939	-0.2053
NONHSAT187439.1	1.030584	6.056684	6.23431	0.001063	0.057939	-0.20703
lnc-CEP128-2:1	2.595994	3.179868	6.234021	0.001063	0.057939	-0.20727
lnc-MYC-20:1	1.070588	4.156624	6.232163	0.001065	0.057939	-0.20878
lnc-GGTLC1-6:1	-1.29066	5.624681	-6.23072	0.001066	0.057939	-0.20996
T219111	1.092544	4.365787	6.230709	0.001066	0.057939	-0.20997
NONHSAT184413.1	-1.20402	4.346752	-6.22875	0.001068	0.057955	-0.21157
lnc-LIG1-2:1	1.102543	7.399468	6.226501	0.001069	0.057955	-0.2134
NONHSAT217711.1	-1.72671	2.465746	-6.22428	0.001071	0.058016	-0.21522
ENST00000504610.2	1.048773	6.006767	6.221839	0.001073	0.058066	-0.21721
ENST00000440900.1	1.319138	1.723433	6.219161	0.001076	0.05808	-0.2194
ENST00000423838.1	2.024097	3.025791	6.218698	0.001076	0.05808	-0.21978
NONHSAT204084.1	1.357214	4.241945	6.217405	0.001077	0.05808	-0.22083
T314485	-1.09118	3.145656	-6.21618	0.001078	0.05808	-0.22184
lnc-DNAJC24-6:1	-1.20405	4.693325	-6.21613	0.001078	0.05808	-0.22187
LINC00938:7	-1.66197	2.956466	-6.21514	0.001079	0.058095	-0.22269
lnc-C8orf34-2:1	-1.72405	8.135274	-6.21342	0.001081	0.058118	-0.22409
lnc-IL7-3:1	-1.00314	8.43155	-6.21331	0.001081	0.058118	-0.22418
lnc-ZNF577-1:1	-1.05573	3.256839	-6.21076	0.001083	0.058165	-0.22627
NONHSAT184132.1	-1.43918	3.432224	-6.21041	0.001083	0.058165	-0.22656
NONHSAT224347.1	2.257516	3.55166	6.200047	0.001092	0.058348	-0.23505
MSTRG.22953.2	-1.4481	4.001354	-6.19998	0.001092	0.058348	-0.2351
lnc-ERG-4:1	2.443192	2.317187	6.198587	0.001093	0.058348	-0.23624
MSTRG.64245.1	1.3228	2.917311	6.197785	0.001094	0.058348	-0.2369
lnc-FBXO9-6:1	-1.08535	5.541115	-6.19678	0.001095	0.058348	-0.23772

NONHSAT158087.1	1.052985	1.745359	6.196346	0.001095	0.058348	-0.23808
MSTRG.50048.2	-1.94904	2.217701	-6.19592	0.001096	0.058348	-0.23843
ENST00000538190.2	1.091058	2.086516	6.195667	0.001096	0.058348	-0.23864
lnc-TAOK3-10:1	1.732046	3.300023	6.193579	0.001098	0.058384	-0.24035
ENST00000606988.1	-1.22683	3.095966	-6.18956	0.001101	0.058509	-0.24365
ENST00000653501.1	2.252449	2.913834	6.185331	0.001105	0.058645	-0.24712
ENST00000471537.3	1.080333	6.934386	6.184438	0.001106	0.058656	-0.24786
ENST00000580270.1	-1.44956	2.574198	-6.18218	0.001108	0.0587	-0.24971
MSTRG.60188.1	-1.03372	4.534089	-6.18088	0.001109	0.05873	-0.25078
NONHSAT205068.1	1.921977	2.820771	6.177748	0.001112	0.058846	-0.25336
lnc-HMGN1-2:1	-1.19402	4.30507	-6.17683	0.001113	0.058858	-0.25411
lnc-PRPF18-14:1	-1.15291	4.485523	-6.17244	0.001117	0.058953	-0.25772
ENST00000668271.1	1.216597	4.402889	6.172328	0.001117	0.058953	-0.25782
MSTRG.15843.1	2.053425	6.915198	6.171777	0.001117	0.058953	-0.25827
NONHSAT174328.1	1.647007	1.981726	6.169936	0.001119	0.058953	-0.25979
lnc-COIL-4:1	-1.00497	4.917023	-6.16958	0.001119	0.058953	-0.26008
lnc-SNTG1-9:1	-1.40049	5.159931	-6.16602	0.001122	0.059067	-0.26301
NONHSAT177017.1	1.890017	2.23802	6.165114	0.001123	0.059071	-0.26376
NONHSAT200930.1	1.627638	5.202472	6.163675	0.001124	0.059108	-0.26494
lnc-WDPCP-5:1	1.457108	3.421121	6.159461	0.001128	0.059246	-0.26842
ENST00000671457.1	1.026522	11.79285	6.156819	0.001131	0.059341	-0.2706
MSTRG.42451.1	2.305549	2.57916	6.149715	0.001137	0.059461	-0.27646
NONHSAT205805.1	1.121455	4.87314	6.147614	0.001139	0.059461	-0.2782
LINC01036:28	-1.03259	1.628924	-6.14708	0.001139	0.059461	-0.27864
NONHSAT157985.1	1.397174	1.984399	6.146662	0.00114	0.059461	-0.27899
MSTRG.2445.1	-1.86459	3.422068	-6.14549	0.001141	0.059461	-0.27995
lnc-FGF12-1:2	-1.06247	4.492978	-6.14532	0.001141	0.059461	-0.2801
lnc-NAP1L5-4:1	-1.72267	4.825726	-6.14522	0.001141	0.059461	-0.28018
MSTRG.10921.1	-1.53588	2.969986	-6.13977	0.001146	0.059506	-0.28469
MSTRG.58424.1	1.119691	3.601477	6.137904	0.001148	0.059534	-0.28623
NONHSAT201262.1	2.376696	2.902548	6.133281	0.001152	0.059639	-0.29006
ENST00000604965.1	-1.23979	5.373685	-6.13316	0.001152	0.059639	-0.29016
lnc-ETAA1-14:1	1.071662	6.457593	6.131931	0.001153	0.059667	-0.29117
ENST00000635510.1	1.275921	3.697413	6.130408	0.001155	0.059679	-0.29244
lnc-CASD1-1:1	-1.45549	2.469594	-6.12772	0.001157	0.059746	-0.29466
NONHSAT222006.1	1.659031	2.300107	6.122062	0.001163	0.059839	-0.29936
NR_003693	-1.22646	4.669416	-6.12095	0.001164	0.059856	-0.30028
NONHSAT170538.1	1.069159	4.3965	6.120316	0.001164	0.059856	-0.3008
MSTRG.59196.1	-1.54816	4.549967	-6.11781	0.001166	0.059856	-0.30288
ENST00000653058.1	1.207214	2.897214	6.117706	0.001167	0.059856	-0.30297
MSTRG.16882.1	1.169396	4.337487	6.115556	0.001169	0.059856	-0.30476
NONHSAT174778.1	2.304246	2.321649	6.114275	0.00117	0.059856	-0.30582
LINC01833:24	1.655456	2.168975	6.114124	0.00117	0.059856	-0.30594
lnc-NCBP3-1:1	-1.0254	7.080501	-6.1139	0.00117	0.059856	-0.30613
MSTRG.14375.2	1.427722	5.243441	6.112895	0.001171	0.059856	-0.30697
NONHSAT185192.1	1.247418	4.814099	6.1124	0.001172	0.059856	-0.30738
NONHSAT164039.1	1.103341	1.705162	6.112141	0.001172	0.059856	-0.30759
NONHSAT172154.1	-1.32081	2.326845	-6.10989	0.001174	0.05987	-0.30946

NR_033987	1.665878	5.028549	6.108078	0.001176	0.05987	-0.31097
lnc-DGKH-1:1	-1.00389	3.878267	-6.10593	0.001178	0.059883	-0.31275
NR_110747	-1.71784	3.03365	-6.10156	0.001182	0.059935	-0.31639
MSTRG.62095.1	-1.19777	5.861042	-6.10094	0.001182	0.059935	-0.31691
NONHSAT161611.1	1.654334	4.700669	6.091072	0.001192	0.060174	-0.32512
lnc-STRN3-18:1	-1.48189	3.148585	-6.09025	0.001193	0.060174	-0.32581
lnc-SCN5A-3:1	-1.17979	1.885292	-6.08895	0.001194	0.060174	-0.32689
ENST00000649594.1	2.148481	2.176423	6.088616	0.001194	0.060174	-0.32717
T262091	1.335739	4.224706	6.088094	0.001195	0.060174	-0.32761
T184375	-1.54376	2.732096	-6.08347	0.001199	0.060235	-0.33147
MSTRG.32186.1	1.890789	2.027172	6.083129	0.0012	0.060235	-0.33175
PVT1:79	-1.33526	6.236318	-6.08289	0.0012	0.060235	-0.33195
lnc-ANKRD27-9:1	-1.34813	4.054112	-6.0813	0.001201	0.060235	-0.33327
NONHSAT221876.1	1.110837	5.360727	6.080637	0.001202	0.060235	-0.33383
ENST00000641190.1	1.141356	4.102298	6.080131	0.001202	0.060235	-0.33425
LINC01938:10	1.278632	3.553749	6.0795	0.001203	0.060235	-0.33478
MSTRG.23106.1	1.722256	4.374014	6.079459	0.001203	0.060235	-0.33481
MSTRG.23271.1	-1.74135	2.471852	-6.07785	0.001205	0.060283	-0.33615
NONHSAT210403.1	1.012599	5.596018	6.075357	0.001207	0.060331	-0.33824
MSTRG.56343.1	-1.29334	2.645561	-6.07366	0.001209	0.060331	-0.33965
lnc-MEF2C-8:1	-1.65967	2.57077	-6.07297	0.001209	0.060331	-0.34023
ENST00000667496.1	1.042788	3.92661	6.071496	0.001211	0.060337	-0.34146
ENST00000515882.2	-1.48819	5.236503	-6.06824	0.001214	0.06037	-0.34419
NONHSAT210612.1	-1.45453	1.907298	-6.06636	0.001216	0.060426	-0.34576
NONHSAT222553.1	1.601509	5.057562	6.063774	0.001218	0.060493	-0.34792
NONHSAT197805.1	1.009503	5.784642	6.059173	0.001223	0.060616	-0.35178
NONHSAT158375.1	1.928771	2.847568	6.057169	0.001225	0.06062	-0.35345
lnc-CIB1-5:2	-1.36078	2.449718	-6.05565	0.001227	0.06062	-0.35473
ENST00000658878.1	1.474803	4.173364	6.05545	0.001227	0.06062	-0.35489
ENST00000649682.2	1.172702	3.176313	6.049383	0.001233	0.060785	-0.35998
NONHSAT163651.1	2.207278	2.162845	6.048815	0.001233	0.060785	-0.36046
MSTRG.18858.1	-1.41977	3.295922	-6.04589	0.001236	0.060838	-0.36291
NONHSAT201412.1	1.012023	3.283435	6.042697	0.00124	0.060913	-0.36559
ENST00000379677.2	1.285616	3.227448	6.041976	0.00124	0.060913	-0.3662
lnc-SIK2-3:2	-1.58851	1.893877	-6.03924	0.001243	0.060944	-0.36849
ENST00000614119.1	-1.69843	4.985549	-6.03723	0.001245	0.060944	-0.37018
MSTRG.38136.1	1.404315	7.414897	6.036549	0.001246	0.060944	-0.37076
T127511	-1.44775	2.055429	-6.03626	0.001246	0.060944	-0.371
lnc-ECHDC3-6:1	1.093497	4.013552	6.035579	0.001247	0.060944	-0.37157
NONHSAT191749.1	1.188621	4.706412	6.034722	0.001248	0.060944	-0.37229
lnc-CLEC4C-1:1	2.279237	2.454733	6.034279	0.001248	0.060944	-0.37267
NONHSAT178944.1	2.585864	2.803139	6.033508	0.001249	0.060944	-0.37331
MSTRG.41610.78	2.100046	2.101762	6.029185	0.001253	0.060944	-0.37695
lnc-ARF4-2:1	-1.29251	7.102437	-6.02793	0.001254	0.060944	-0.378
lnc-RASEF-2:1	1.066669	1.605251	6.027733	0.001255	0.060944	-0.37817
NONHSAT196308.1	-1.02006	4.658157	-6.0243	0.001258	0.060944	-0.38106
ENST00000651456.1	-1.80466	3.161663	-6.0206	0.001262	0.060944	-0.38418
T338502	1.316204	3.808476	6.019874	0.001263	0.060944	-0.38479

NONHSAT223967.1	1.801764	5.633953	6.018023	0.001265	0.060944	-0.38635
NONHSAT153263.1	1.49574	2.134194	6.017036	0.001266	0.060944	-0.38718
MSTRG.10798.1	1.555841	2.67431	6.014278	0.001269	0.060953	-0.38951
NONHSAT154807.1	1.068553	1.591922	6.013163	0.00127	0.060953	-0.39045
lnc-FER-18:2	1.544945	3.33761	6.011588	0.001271	0.060953	-0.39178
lnc-SH3BGR-1:1	1.24048	1.644146	6.009701	0.001273	0.060963	-0.39337
lnc-TXNDC5-3:1	-1.17099	4.032166	-6.00962	0.001273	0.060963	-0.39344
NONHSAT164601.1	-1.01758	3.015203	-6.00878	0.001274	0.060963	-0.39415
lnc-NPM3-1:1	-1.17543	6.705812	-6.00852	0.001274	0.060963	-0.39437
T223628	1.45141	5.219496	6.003874	0.001279	0.060974	-0.39829
lnc-TRIO-5:1	1.087242	4.266657	5.996898	0.001287	0.061014	-0.40418
T309712	1.146541	4.052562	5.994922	0.001289	0.061014	-0.40585
LINC01356:1	-1.17955	3.709961	-5.99362	0.00129	0.061014	-0.40696
lnc-MED10-6:3	1.242867	3.607475	5.99356	0.00129	0.061014	-0.40701
lnc-NOP56-1:2	-1.81779	2.355314	-5.99187	0.001292	0.061014	-0.40843
NONHSAT186932.1	1.3773	2.016485	5.984994	0.001299	0.061108	-0.41426
NONHSAT166709.1	1.015589	3.549882	5.984546	0.0013	0.061108	-0.41464
NONHSAT219885.1	1.468659	4.447789	5.981852	0.001303	0.061136	-0.41692
lnc-FDX1-2:1	-1.22589	4.044663	-5.98149	0.001303	0.061136	-0.41723
lnc-SLCO1A2-5:1	1.919783	2.494211	5.981445	0.001303	0.061136	-0.41726
MSTRG.56895.1	1.51512	2.170864	5.980725	0.001304	0.061144	-0.41787
NONHSAT158206.1	1.580868	3.635309	5.977851	0.001307	0.061208	-0.42031
NONHSAT201126.1	1.637538	4.891624	5.977654	0.001307	0.061208	-0.42048
lnc-RFX7-1:1	-1.51102	3.816366	-5.97717	0.001308	0.061208	-0.42089
NR_038958	-1.16915	2.284941	-5.97582	0.001309	0.061243	-0.42204
lnc-SORCS3-7:1	-1.19691	2.441714	-5.97533	0.00131	0.061243	-0.42245
ENST00000415736.1	1.434482	4.970122	5.974693	0.00131	0.061245	-0.42299
NONHSAT161166.1	-2.00578	2.296032	-5.97368	0.001311	0.061245	-0.42385
NONHSAT205746.1	1.041055	4.068986	5.972872	0.001312	0.061245	-0.42454
lnc-ERGIC1-3:1	1.155815	7.102962	5.968745	0.001317	0.061273	-0.42804
MSTRG.52827.1	-2.12526	2.845617	-5.96828	0.001317	0.061273	-0.42843
NONHSAT176375.1	1.454094	3.002496	5.966212	0.001319	0.061332	-0.43019
NONHSAT206490.1	1.00726	5.02344	5.962806	0.001323	0.061393	-0.43308
lnc-ADGRF5-2:1	1.524338	4.554999	5.96272	0.001323	0.061393	-0.43316
lnc-MRGPRX2-1:1	1.212898	4.739891	5.961923	0.001324	0.061393	-0.43384
ITGB1-DT:30	-1.55215	2.087672	-5.96097	0.001325	0.061393	-0.43464
NONHSAT197259.1	1.781571	2.283013	5.960304	0.001326	0.061393	-0.43521
NONHSAT160239.1	-1.13499	2.321533	-5.95974	0.001326	0.061393	-0.43569
lnc-COG2-4:2	-2.21175	2.926791	-5.95959	0.001327	0.061393	-0.43582
ENST00000422799.1	1.076787	1.913136	5.958481	0.001328	0.061393	-0.43676
NR_110745	1.029162	1.687366	5.95782	0.001328	0.061393	-0.43732
NONHSAT171253.1	-1.20667	4.814724	-5.95692	0.001329	0.061406	-0.43809
NONHSAT166964.1	2.366148	2.276061	5.95478	0.001332	0.061458	-0.43991
MSTRG.3780.1	1.451358	6.635818	5.953029	0.001334	0.061463	-0.4414
NONHSAT217284.1	-1.26671	3.852084	-5.94922	0.001338	0.061598	-0.44464
NONHSAT206680.1	-1.31215	4.72347	-5.94825	0.001339	0.061607	-0.44547
lnc-ANKRD45-3:3	1.790512	3.814906	5.944962	0.001343	0.061621	-0.44827
MSTRG.47239.1	1.750252	2.511006	5.944693	0.001343	0.061621	-0.4485

NONHSAT217935.1	2.048048	2.777937	5.942824	0.001345	0.061625	-0.45009
NONHSAT206554.1	1.092737	2.495088	5.941974	0.001346	0.061625	-0.45082
MSTRG.9646.1	1.033041	4.731059	5.941491	0.001346	0.061625	-0.45123
NONHSAT207630.1	1.129737	1.702778	5.938671	0.00135	0.06163	-0.45364
LINC01270:16	-1.59836	1.916735	-5.93755	0.001351	0.06163	-0.4546
MSTRG.6354.1	1.310305	4.149363	5.933352	0.001355	0.061694	-0.45818
ENST00000412602.1	1.436823	1.977539	5.932999	0.001356	0.061694	-0.45848
MSTRG.43659.1	-1.60941	2.525174	-5.93198	0.001357	0.061717	-0.45934
ENST00000513358.3	-1.09208	5.778136	-5.92744	0.001362	0.061865	-0.46323
NONHSAT198023.1	1.746184	2.288766	5.919757	0.001371	0.062075	-0.4698
NONHSAT184025.1	2.216919	2.584909	5.916987	0.001374	0.062075	-0.47217
LINC01358:14	-1.14127	1.849025	-5.91659	0.001374	0.062075	-0.47251
lnc-GUSB-11:1	1.008132	7.224175	5.916163	0.001375	0.062075	-0.47287
lnc-SLC20A1-3:1	-1.1942	1.947435	-5.91528	0.001376	0.062075	-0.47363
NONHSAT223715.1	4.014175	3.753762	5.915166	0.001376	0.062075	-0.47372
NONHSAT164978.1	1.902276	2.181358	5.914875	0.001376	0.062075	-0.47397
lnc-KCTD18-2:1	1.610138	5.660102	5.91349	0.001378	0.062075	-0.47516
ENST00000527012.1	-1.1913	4.297246	-5.91295	0.001378	0.062075	-0.47562
lnc-COMMD6-6:1	-2.02411	2.528887	-5.91249	0.001379	0.062075	-0.47602
NONHSAT210523.1	-1.19451	1.909908	-5.91082	0.001381	0.062077	-0.47745
T334719	-1.3316	3.698634	-5.9089	0.001383	0.06212	-0.47909
lnc-PCBP1-7:1	1.594969	3.983531	5.907655	0.001385	0.062156	-0.48016
NONHSAT177107.1	-1.65538	2.374821	-5.90417	0.001389	0.062159	-0.48315
NONHSAT218126.1	2.417788	2.611483	5.903786	0.001389	0.062159	-0.48348
NR_110159	2.036309	8.409525	5.903199	0.00139	0.062159	-0.48398
NONHSAT186675.1	-1.60797	3.948292	-5.89892	0.001395	0.062199	-0.48766
ENST00000558572.1	1.916829	2.466503	5.898837	0.001395	0.062199	-0.48772
ENST00000498432.6	-1.27977	4.229006	-5.89839	0.001395	0.062199	-0.4881
MSTRG.66881.1	2.07632	2.107201	5.897564	0.001396	0.062199	-0.48881
lnc-C12orf74-13:2	1.377015	2.84135	5.896118	0.001398	0.062199	-0.49006
lnc-MPHOSPH8-11:3	1.115334	1.731842	5.895577	0.001398	0.062199	-0.49052
ENST00000559148.1	-2.11001	2.874445	-5.89421	0.0014	0.062241	-0.49169
MSTRG.35582.1	1.008405	6.130482	5.888858	0.001406	0.06247	-0.49629
ENST00000515153.1	-1.26416	6.855323	-5.88775	0.001408	0.06247	-0.49725
ENST00000655744.1	1.269209	1.775874	5.887256	0.001408	0.06247	-0.49767
MSTRG.34127.1	1.608897	6.20322	5.88672	0.001409	0.06247	-0.49813
NONHSAT165853.1	1.564606	1.805579	5.883492	0.001412	0.062576	-0.50091
ENST00000454781.6	-1.25762	2.093244	-5.87929	0.001417	0.062653	-0.50452
lnc-DHX37-25:1	-1.12895	6.750555	-5.87848	0.001418	0.062653	-0.50522
T313558	2.806537	2.950761	5.877782	0.001419	0.062653	-0.50582
NONHSAT205265.1	1.400657	4.3191	5.877614	0.001419	0.062653	-0.50597
lnc-MEGF10-2:1	1.666127	2.481581	5.873973	0.001424	0.062653	-0.5091
lnc-ARMCX4-3:1	-1.44437	1.856895	-5.87233	0.001426	0.06269	-0.51052
NONHSAT197795.1	1.080529	7.640756	5.870548	0.001428	0.062746	-0.51206
NONHSAT212523.1	-1.62525	2.083541	-5.8702	0.001428	0.062746	-0.51235
NONHSAT160640.1	2.06409	2.587756	5.86656	0.001432	0.062826	-0.51549
NONHSAT182546.1	2.036521	4.398157	5.864672	0.001435	0.062849	-0.51712
lnc-COMMD10-6:1	-1.05583	5.2017	-5.86427	0.001435	0.062849	-0.51747

NONHSAT195170.1	1.414542	3.633793	5.863579	0.001436	0.062849	-0.51807
lnc-MAP3K3-1:1	-1.22908	3.726769	-5.86352	0.001436	0.062849	-0.51812
NONHSAT198634.1	-1.37358	2.394009	-5.86237	0.001437	0.062881	-0.51911
MSTRG.9960.1	-1.69513	5.191942	-5.85973	0.001441	0.062988	-0.52139
MSTRG.38433.1	1.151991	1.897031	5.853049	0.001449	0.063122	-0.52716
lnc-TLX3-8:1	-1.13201	4.636765	-5.85219	0.00145	0.063122	-0.52791
ENST00000509194.1	-1.37199	4.581345	-5.85008	0.001452	0.063175	-0.52973
ENST00000503323.1	1.500911	2.452632	5.849939	0.001452	0.063175	-0.52985
MSTRG.70548.1	1.143767	3.768913	5.848569	0.001454	0.063196	-0.53103
NONHSAT188007.1	-2.0372	2.560734	-5.84608	0.001457	0.063246	-0.53318
MSTRG.44987.1	1.774352	4.63425	5.845278	0.001458	0.063261	-0.53388
NONHSAT170226.1	1.371553	5.527558	5.838537	0.001466	0.063479	-0.53972
MSTRG.31129.1	1.389232	2.852823	5.834673	0.001471	0.063561	-0.54307
ENST00000653247.1	1.322558	3.278748	5.8342	0.001472	0.063561	-0.54348
NONHSAT165209.1	1.406627	4.101259	5.833495	0.001472	0.063561	-0.54409
ENST00000635449.1	-1.50302	1.985814	-5.83113	0.001475	0.063561	-0.54614
lnc-CLMP-8:1	1.021836	2.717776	5.829392	0.001478	0.063561	-0.54765
lnc-NKAIN1-1:2	-1.39716	5.81509	-5.82932	0.001478	0.063561	-0.54771
T210371	1.31899	4.057502	5.826535	0.001481	0.063628	-0.55013
lnc-PIGP-1:1	2.190132	2.560643	5.824706	0.001483	0.063698	-0.55171
T083348	1.124033	1.976113	5.822358	0.001486	0.063705	-0.55375
MSTRG.50700.1	1.369824	4.499484	5.81949	0.00149	0.063705	-0.55624
lnc-GRIK3-2:1	1.617577	2.283577	5.816218	0.001494	0.063798	-0.55909
lnc-BRF1-65:1	2.701613	3.557931	5.814677	0.001496	0.063854	-0.56043
lnc-TMC8-4:1	-1.16	4.538452	-5.81092	0.001501	0.063999	-0.56369
ENST00000443284.1	-1.22816	3.152925	-5.81044	0.001501	0.063999	-0.56411
lnc-HECA-4:1	-1.00692	1.670125	-5.8083	0.001504	0.064038	-0.56597
lnc-FAM210A-7:1	1.404404	1.794667	5.805994	0.001507	0.064048	-0.56798
NONHSAT166773.1	1.138963	4.494335	5.802673	0.001511	0.064136	-0.57087
NR_033652	1.36898	5.404259	5.798874	0.001516	0.064136	-0.57418
T114599	1.376848	3.815981	5.797861	0.001517	0.064136	-0.57507
ENST00000577309.1	1.134279	4.146452	5.795185	0.00152	0.064136	-0.5774
lnc-NOB1-2:1	-1.26485	5.902487	-5.79478	0.001521	0.064136	-0.57775
lnc-COL26A1-3:2	1.398474	4.718989	5.79478	0.001521	0.064136	-0.57775
lnc-ADGRV1-11:1	1.147037	4.920539	5.790861	0.001526	0.064136	-0.58117
ENST00000671287.1	-1.16108	1.654051	-5.79015	0.001527	0.064136	-0.58179
ENST00000605480.1	-1.19841	2.891688	-5.7893	0.001528	0.064136	-0.58253
lnc-IFNA1-5:1	-1.01861	5.736886	-5.78909	0.001528	0.064136	-0.58272
NONHSAT201845.1	1.214781	5.33026	5.788927	0.001528	0.064136	-0.58286
MSTRG.40845.1	2.111365	2.56634	5.788435	0.001529	0.064136	-0.58329
lnc-NRP1-7:1	2.056066	2.575913	5.788384	0.001529	0.064136	-0.58333
NONHSAT150381.1	-1.19862	5.721252	-5.78644	0.001532	0.064164	-0.58503
lnc-CHD2-9:1	-1.45695	6.063533	-5.78637	0.001532	0.064164	-0.58509
NONHSAT150139.1	-1.36394	2.734544	-5.78555	0.001533	0.064182	-0.58581
NONHSAT188135.1	1.56139	2.441814	5.7839	0.001535	0.064185	-0.58725
NONHSAT210761.1	1.162402	5.174648	5.780915	0.001539	0.064272	-0.58986
lnc-USP9Y-10:1	-1.09689	1.788563	-5.77811	0.001542	0.064345	-0.59231
lnc-GPR75-3:1	-1.11377	1.804951	-5.77747	0.001543	0.064345	-0.59287

NONHSAT214501.1	1.252919	2.321014	5.776912	0.001544	0.064345	-0.59336
MSTRG.51632.1	1.339416	3.483341	5.776613	0.001544	0.064345	-0.59362
ENST00000568911.5	-1.99404	3.302844	-5.77271	0.001549	0.064487	-0.59703
MSTRG.17241.3	1.415524	1.777728	5.772523	0.00155	0.064487	-0.5972
NONHSAT189382.1	-1.20052	3.884512	-5.77184	0.001551	0.064497	-0.59779
lnc-FILIP1L-6:1	-2.31212	4.40003	-5.76994	0.001553	0.064573	-0.59945
NONHSAT205253.1	1.080069	3.033821	5.769171	0.001554	0.064589	-0.60013
lnc-ZFP90-2:1	1.722089	4.690135	5.764298	0.00156	0.064719	-0.6044
NONHSAT208159.1	2.247341	5.462588	5.764083	0.001561	0.064719	-0.60459
lnc-CST8-5:1	2.033967	2.161879	5.763847	0.001561	0.064719	-0.60479
lnc-NEK11-4:1	-1.16285	4.655378	-5.76156	0.001564	0.06479	-0.60679
lnc-MPZL3-4:1	-1.13101	9.781642	-5.75882	0.001568	0.06486	-0.6092
lnc-MARF1-1:2	-1.01574	5.917673	-5.75569	0.001572	0.064967	-0.61194
ENST00000533859.1	1.159431	4.286763	5.752603	0.001576	0.065031	-0.61465
NONHSAT213668.1	-1.31238	2.488752	-5.75206	0.001577	0.065031	-0.61513
ENST00000449034.1	-1.50755	3.434253	-5.75098	0.001578	0.06505	-0.61608
lnc-PCMTD2-2:3	1.084303	3.542205	5.749453	0.00158	0.065081	-0.61742
lnc-HS3ST3B1-10:1	-1.19599	6.331648	-5.74713	0.001583	0.065181	-0.61946
ENST00000434112.1	-1.6576	3.557078	-5.74602	0.001585	0.065197	-0.62043
NONHSAT165013.1	1.361313	3.412379	5.745224	0.001586	0.065207	-0.62114
NONHSAT180304.1	2.496201	2.969276	5.743691	0.001588	0.065255	-0.62248
MSTRG.19197.1	2.037867	2.648474	5.743335	0.001588	0.065255	-0.62279
lnc-RPL7-3:2	-1.4281	4.178927	-5.74061	0.001592	0.06527	-0.62519
NONHSAT206487.1	1.200628	6.412009	5.740561	0.001592	0.06527	-0.62523
MSTRG.27268.7	1.11116	4.635278	5.737142	0.001597	0.065274	-0.62824
lnc-CDH3-3:1	2.390093	2.627616	5.736992	0.001597	0.065274	-0.62837
lnc-GLIS1-2:3	-1.39005	1.989431	-5.73292	0.001602	0.065408	-0.63195
NONHSAT184782.1	1.70134	4.705363	5.72881	0.001608	0.06548	-0.63558
MSTRG.18373.1	1.878573	2.666065	5.728017	0.001609	0.06548	-0.63628
lnc-APMAP-2:1	1.423876	3.062257	5.72752	0.00161	0.06548	-0.63671
ENST00000424355.1	-1.63492	2.13048	-5.72719	0.00161	0.06548	-0.637
lnc-GK3P-6:1	1.411953	4.828528	5.726841	0.001611	0.06548	-0.63731
ENST00000440413.1	1.433784	4.416742	5.725099	0.001613	0.065503	-0.63885
ENST00000509924.3	1.639917	7.340392	5.724518	0.001614	0.065503	-0.63936
MSTRG.42620.1	1.631553	1.861681	5.722549	0.001616	0.065521	-0.6411
ENST00000663734.1	-1.91577	2.541502	-5.72088	0.001619	0.065573	-0.64257
lnc-ARFGEF2-4:1	1.516601	2.120542	5.719225	0.001621	0.065632	-0.64403
lnc-NR3C2-7:2	1.562875	1.856152	5.717643	0.001623	0.065632	-0.64542
NONHSAT193412.1	-2.04159	2.384737	-5.717	0.001624	0.065632	-0.646
NONHSAT193115.1	1.540605	5.034968	5.716403	0.001625	0.065632	-0.64652
ENST00000587412.1	1.039301	5.64212	5.716035	0.001625	0.065632	-0.64684
lnc-KCNS3-1:1	-1.95648	4.632682	-5.71518	0.001627	0.065653	-0.6476
T234182	1.325448	1.762269	5.709448	0.001634	0.065819	-0.65266
NONHSAT191647.1	1.132984	4.3381	5.706397	0.001639	0.065853	-0.65536
NONHSAT214396.1	-1.57924	2.087815	-5.70638	0.001639	0.065853	-0.65537
NR_024627	2.165337	5.429499	5.705241	0.00164	0.065853	-0.65638
lnc-RTN4R-1:5	1.408006	1.823628	5.705047	0.001641	0.065853	-0.65655
MSTRG.67087.6	1.694484	1.959918	5.704538	0.001641	0.065853	-0.657

lnc-CFAP99-7:1	1.353452	3.159637	5.70328	0.001643	0.065871	-0.65811
ENST00000412918.1	1.879421	2.265308	5.701068	0.001646	0.065937	-0.66007
ENST00000549710.1	2.278735	2.899385	5.699814	0.001648	0.06596	-0.66118
MSTRG.36159.1	1.238704	1.654675	5.698815	0.001649	0.06599	-0.66206
NONHSAT154697.1	1.506141	2.548028	5.69764	0.001651	0.06603	-0.6631
ENST00000420705.2	1.437479	2.220522	5.69644	0.001653	0.066071	-0.66417
lnc-RXFP4-2:2	1.026656	5.075736	5.69251	0.001658	0.066132	-0.66765
lnc-DHX15-1:2	-1.46221	4.651895	-5.69224	0.001658	0.066132	-0.66789
lnc-TMEM168-4:1	-1.28975	7.173495	-5.69174	0.001659	0.066132	-0.66833
NONHSAT172266.1	-1.01817	3.438888	-5.68999	0.001662	0.066171	-0.66988
NONHSAT206525.1	1.83381	3.215451	5.689269	0.001663	0.066186	-0.67052
MSTRG.55088.1	-1.85873	2.985125	-5.68583	0.001668	0.066249	-0.67357
ENST00000559041.1	-1.38713	3.932545	-5.68265	0.001672	0.066298	-0.67639
lnc-SPERT-3:1	1.256522	4.648442	5.681438	0.001674	0.06632	-0.67746
NONHSAT155187.1	1.367859	6.213416	5.68134	0.001674	0.06632	-0.67755
lnc-SASS6-5:2	-1.25885	3.554967	-5.67798	0.001679	0.066458	-0.68054
MSTRG.43574.1	1.554335	4.068985	5.674559	0.001684	0.066573	-0.68357
ENST00000658044.1	-1.04684	5.29121	-5.67357	0.001685	0.066603	-0.68445
lnc-NIFK-1:3	1.157223	3.600358	5.667017	0.001694	0.066824	-0.69028
NONHSAT198031.1	1.033041	5.043112	5.666474	0.001695	0.066824	-0.69076
lnc-GNRH2-1:1	-1.41923	4.211335	-5.66265	0.001701	0.066911	-0.69416
lnc-FAT3-6:1	-1.85179	2.643854	-5.66123	0.001703	0.066911	-0.69543
NONHSAT173947.1	1.142843	1.90593	5.659089	0.001706	0.066911	-0.69733
lnc-MEST-6:1	-1.25481	4.516338	-5.65771	0.001708	0.066911	-0.69856
lnc-CTNND2-1:3	-1.25048	2.187058	-5.65717	0.001709	0.066911	-0.69904
T213659	-1.06612	5.667049	-5.65701	0.001709	0.066911	-0.69918
NONHSAT166690.1	1.788717	7.36978	5.656176	0.00171	0.066911	-0.69993
ENST00000475981.6	1.347734	2.028936	5.65453	0.001713	0.066911	-0.70139
MSTRG.36424.3	1.634018	4.312167	5.654226	0.001713	0.066911	-0.70166
ENST00000456087.1	-1.19904	4.215416	-5.654	0.001713	0.066911	-0.70186
NONHSAT163663.1	1.735529	2.219179	5.651967	0.001716	0.067002	-0.70368
KDM4A-AS1:10	-1.35602	5.8922	-5.6507	0.001718	0.067048	-0.70481
MSTRG.17479.1	1.174495	2.058886	5.646375	0.001725	0.06708	-0.70866
NONHSAT210333.1	1.475714	1.821066	5.645898	0.001725	0.06708	-0.70909
MSTRG.8286.1	-1.56984	4.120119	-5.64561	0.001726	0.06708	-0.70934
NONHSAT158962.1	1.487513	4.372461	5.645506	0.001726	0.06708	-0.70944
NONHSAT205289.1	-1.56086	3.132958	-5.64466	0.001727	0.06708	-0.71019
NONHSAT172153.1	-1.68742	3.695069	-5.6446	0.001727	0.06708	-0.71025
lnc-SLC39A11-10:48	1.065107	4.939352	5.644494	0.001727	0.06708	-0.71034
ENST00000663783.1	1.140268	4.208624	5.644049	0.001728	0.06708	-0.71074
lnc-FANCL-6:1	-1.47755	2.31024	-5.64381	0.001728	0.06708	-0.71095
MSTRG.51837.1	2.155672	2.910109	5.643231	0.001729	0.06708	-0.71147
ENST00000558258.1	-1.11904	4.606123	-5.64311	0.001729	0.06708	-0.71158
ENST00000600074.1	1.607192	4.180519	5.642925	0.00173	0.06708	-0.71174
lnc-MREG-3:1	-1.13043	6.752142	-5.6414	0.001732	0.067116	-0.7131
lnc-WDR37-5:3	1.198823	4.022348	5.63975	0.001734	0.067134	-0.71458
MSTRG.63393.9	1.993713	9.704844	5.637804	0.001737	0.067136	-0.71631
NONHSAT193978.1	-1.11287	2.308705	-5.63704	0.001738	0.067136	-0.717

NONHSAT175668.1	1.619074	1.930502	5.636811	0.001739	0.067136	-0.7172
MSTRG.7706.1	1.032861	3.214663	5.636262	0.00174	0.067136	-0.71769
NONHSAT218418.1	2.43553	2.519382	5.635767	0.00174	0.067136	-0.71814
lnc-P2RY1-3:5	1.83759	2.723115	5.635529	0.001741	0.067136	-0.71835
ENST00000526388.2	1.023332	5.724458	5.633755	0.001743	0.067146	-0.71993
lnc-RPGR-5:1	1.12199	4.820512	5.629486	0.00175	0.067262	-0.72375
NONHSAT184739.1	-1.15412	4.034112	-5.62687	0.001754	0.067315	-0.72609
NONHSAT162689.1	-1.00839	5.628387	-5.62679	0.001754	0.067315	-0.72616
NONHSAT156796.1	-1.85507	3.257949	-5.6262	0.001755	0.067323	-0.72669
NONHSAT191037.1	2.073375	2.49803	5.625513	0.001756	0.067337	-0.72731
MSTRG.52091.5	1.6598	2.404044	5.620846	0.001763	0.067535	-0.73149
T363705	-1.22214	3.762251	-5.62076	0.001763	0.067535	-0.73156
NONHSAT185771.1	-1.18189	5.31843	-5.61803	0.001767	0.067553	-0.73401
ENST00000607296.1	1.198062	4.992483	5.617966	0.001767	0.067553	-0.73407
NONHSAT193503.1	1.380997	4.24927	5.615534	0.001771	0.067553	-0.73624
NONHSAT193124.1	-1.04162	6.07629	-5.61546	0.001771	0.067553	-0.73631
NONHSAT203604.1	-1.01581	1.874832	-5.61503	0.001772	0.067553	-0.73669
lnc-CMPK2-7:1	1.234366	5.086249	5.612959	0.001775	0.067553	-0.73855
lnc-ZNF438-1:4	-1.41541	4.55932	-5.61242	0.001776	0.067559	-0.73904
NONHSAT219291.1	2.854655	3.109118	5.611838	0.001776	0.067567	-0.73956
MSTRG.42522.11	-1.45838	3.204567	-5.61054	0.001778	0.067591	-0.74072
lnc-GATA3-2:1	2.358218	2.583698	5.60885	0.001781	0.067613	-0.74224
NONHSAT177387.1	1.593688	1.849336	5.608206	0.001782	0.067625	-0.74282
MSTRG.44081.1	-1.37673	2.110538	-5.60563	0.001786	0.067661	-0.74513
NONHSAT205357.1	1.523718	3.40427	5.605364	0.001786	0.067661	-0.74537
NONHSAT152324.1	1.345875	5.018448	5.605164	0.001787	0.067661	-0.74555
MSTRG.19527.1	-1.63817	2.766602	-5.605	0.001787	0.067661	-0.74569
MSTRG.15821.1	1.127285	7.336254	5.604027	0.001788	0.067661	-0.74657
MSTRG.62922.1	-1.00596	5.591048	-5.60282	0.00179	0.067661	-0.74765
MSTRG.67250.1	1.00509	3.301915	5.602349	0.001791	0.067661	-0.74808
NR_027246	-1.33982	4.780321	-5.60071	0.001794	0.06768	-0.74955
NONHSAT173136.1	-1.04094	9.679707	-5.59856	0.001797	0.0677	-0.75148
MSTRG.795.1	1.646157	1.850363	5.598279	0.001797	0.0677	-0.75173
NONHSAT178084.1	1.075031	1.749998	5.597504	0.001798	0.0677	-0.75243
lnc-SLC9A8-3:1	-1.67902	2.829167	-5.59575	0.001801	0.0677	-0.75401
MSTRG.60047.1	-1.36423	3.138394	-5.59329	0.001805	0.067759	-0.75622
lnc-MED27-3:1	-1.21122	4.209437	-5.58963	0.001811	0.06789	-0.75951
ENST00000606008.1	-1.07513	5.489799	-5.58822	0.001813	0.067908	-0.76078
ENST00000615709.1	-1.58335	3.681055	-5.58753	0.001814	0.067919	-0.76141
lnc-GTF2H2-9:1	1.231362	5.886963	5.585505	0.001817	0.067941	-0.76323
MSTRG.14197.1	1.385557	5.904177	5.582613	0.001822	0.068006	-0.76583
NONHSAT219924.1	-1.36794	1.802177	-5.57817	0.001829	0.068158	-0.76984
NONHSAT212351.1	1.146175	3.62001	5.577062	0.00183	0.06816	-0.77084
NR_110826	1.965374	2.01149	5.57483	0.001834	0.068172	-0.77285
ENST00000430905.1	1.350307	5.312504	5.574652	0.001834	0.068172	-0.77301
lnc-SPATA6L-5:1	-1.43214	3.805198	-5.57443	0.001835	0.068172	-0.77321
NONHSAT160218.1	1.251019	3.156256	5.573556	0.001836	0.068172	-0.774
NONHSAT187637.1	2.534892	4.399002	5.572242	0.001838	0.068172	-0.77518

NONHSAT198737.1	-1.35062	4.132114	-5.57209	0.001838	0.068172	-0.77532
MSTRG.13138.1	1.013039	4.613679	5.571256	0.00184	0.068177	-0.77607
ENST00000670221.1	1.090891	4.349452	5.56914	0.001843	0.068195	-0.77798
NONHSAT164853.1	-1.30446	2.618047	-5.56668	0.001847	0.068203	-0.78021
lnc-PDK1-2:1	-1.0428	3.648677	-5.56646	0.001847	0.068203	-0.78041
NONHSAT168857.1	2.619369	2.932415	5.564383	0.001851	0.06828	-0.78228
ENST00000579033.1	1.460587	1.807602	5.56372	0.001852	0.06828	-0.78288
MSTRG.44407.1	-1.07479	2.745659	-5.56305	0.001853	0.06828	-0.78348
NONHSAT167728.1	2.457459	2.338339	5.562835	0.001853	0.06828	-0.78368
ENST00000453199.2	1.648042	5.010903	5.562613	0.001853	0.06828	-0.78388
NONHSAT181377.1	1.50853	3.873226	5.560366	0.001857	0.068347	-0.78591
MSTRG.56279.1	1.321593	3.914726	5.557562	0.001862	0.068429	-0.78845
lnc-ARHGAP15-13:1	-1.82674	3.595972	-5.55597	0.001864	0.068474	-0.78989
lnc-ZC3H8-7:1	1.089923	6.66708	5.555464	0.001865	0.068479	-0.79034
ENST00000442328.1	-1.67701	3.150176	-5.55288	0.001869	0.068532	-0.79269
NR_121618	1.019913	3.357453	5.551903	0.001871	0.068555	-0.79357
lnc-RBBP6-4:4	1.046543	3.992155	5.55068	0.001873	0.068555	-0.79468
lnc-DTL-7:1	-1.26489	3.835987	-5.54768	0.001878	0.068555	-0.79739
NONHSAT215508.1	1.003066	5.705215	5.547425	0.001878	0.068555	-0.79762
MSTRG.15343.32	-1.04362	3.629921	-5.54538	0.001881	0.068555	-0.79948
NONHSAT223500.1	1.211911	3.07086	5.538385	0.001893	0.068555	-0.80582
NONHSAT163037.1	1.720458	3.906993	5.537713	0.001894	0.068555	-0.80643
ENST00000671278.1	1.327648	6.258146	5.537442	0.001894	0.068555	-0.80667
lnc-TEX10-4:1	1.38296	3.24987	5.535527	0.001897	0.068555	-0.80841
lnc-UBXN4-9:1	1.089002	6.04666	5.535326	0.001898	0.068555	-0.80859
NONHSAT176321.1	1.274014	5.995927	5.535176	0.001898	0.068555	-0.80873
NONHSAT184942.1	1.161798	4.96883	5.534104	0.0019	0.068555	-0.8097
NONHSAT186977.1	1.570996	5.641415	5.532797	0.001902	0.068555	-0.81089
NONHSAT179188.1	1.241774	4.718468	5.532377	0.001903	0.068555	-0.81127
NONHSAT196039.1	1.249046	2.042094	5.531332	0.001904	0.068555	-0.81222
NR_073512	1.243654	6.932575	5.530497	0.001906	0.068555	-0.81298
T284818	2.020067	2.427038	5.528673	0.001909	0.068555	-0.81463
NONHSAT177938.1	-1.20808	2.107203	-5.52866	0.001909	0.068555	-0.81464
NONHSAT168566.1	2.111635	6.12662	5.528386	0.001909	0.068555	-0.8149
NONHSAT216545.1	2.306428	2.689894	5.527877	0.00191	0.068555	-0.81536
lnc-TMEM106C-9:1	-1.66732	3.468867	-5.52786	0.00191	0.068555	-0.81537
NONHSAT215659.1	-1.2856	1.88267	-5.52598	0.001913	0.068555	-0.81709
ENST00000567905.1	-1.60054	2.403732	-5.52554	0.001914	0.068556	-0.81748
NR_037839	1.556636	1.850283	5.524104	0.001916	0.068569	-0.81879
MSTRG.62727.7	1.722997	2.899101	5.520507	0.001922	0.068606	-0.82206
lnc-ZNF737-2:1	1.215606	1.727938	5.519092	0.001925	0.068606	-0.82335
MSTRG.57145.1	-1.76507	2.290172	-5.51909	0.001925	0.068606	-0.82335
NONHSAT173891.1	2.962982	3.800128	5.516291	0.001929	0.068631	-0.82589
ENST00000605991.1	-1.4538	4.279136	-5.51558	0.001931	0.068631	-0.82654
NONHSAT213694.1	-1.57299	1.956525	-5.51466	0.001932	0.068647	-0.82738
NONHSAT168125.1	1.319409	1.931151	5.513805	0.001934	0.068665	-0.82816
MSTRG.7663.8	1.552964	4.200382	5.512268	0.001936	0.068665	-0.82956
LINC02447:19	1.079962	3.997408	5.5104	0.001939	0.068665	-0.83126

MSTRG.56072.1	1.273613	4.103899	5.509343	0.001941	0.068692	-0.83222
NONHSAT190377.1	1.043906	3.577309	5.507005	0.001945	0.068791	-0.83435
ENST00000573856.1	-1.70761	2.298809	-5.50451	0.001949	0.068893	-0.83663
NONHSAT220509.1	-1.37205	4.348737	-5.49655	0.001963	0.069154	-0.84389
lnc-CDH6-18:1	-1.73681	2.52751	-5.49611	0.001964	0.069156	-0.84429
NONHSAT205205.1	1.479074	4.862408	5.494898	0.001966	0.069182	-0.8454
NONHSAT224156.1	1.130856	5.124922	5.494231	0.001967	0.069198	-0.84601
NONHSAT197565.1	1.528425	1.880364	5.488993	0.001976	0.069323	-0.85079
lnc-KCNJ10-2:2	1.869242	2.335676	5.488964	0.001976	0.069323	-0.85082
NONHSAT190938.1	-1.05617	3.74491	-5.48751	0.001978	0.069328	-0.85215
NONHSAT187187.1	-1.46204	3.36212	-5.48748	0.001978	0.069328	-0.85218
lnc-HAPLN1-2:3	1.828642	6.317409	5.485415	0.001982	0.069328	-0.85407
ENST00000319682.2	2.025309	2.890225	5.484402	0.001984	0.069328	-0.85499
NR_024366	1.083332	4.303341	5.484303	0.001984	0.069328	-0.85508
MSTRG.44943.1	1.114175	4.178451	5.483167	0.001986	0.069328	-0.85612
lnc-ALDH3B2-2:1	-1.2235	4.76049	-5.48314	0.001986	0.069328	-0.85615
lnc-SLC12A7-1:1	1.117885	5.093188	5.482767	0.001987	0.069328	-0.85649
lnc-TENM1-4:1	1.206823	1.775556	5.480901	0.00199	0.069328	-0.8582
lnc-MYH13-2:1	1.312842	1.73544	5.479491	0.001992	0.069328	-0.85949
NONHSAT167935.1	-1.34085	3.645774	-5.47937	0.001993	0.069328	-0.8596
lnc-CTBP2-2:1	-1.54472	2.812419	-5.47668	0.001997	0.069337	-0.86206
T009292	1.93937	2.142989	5.476378	0.001998	0.069337	-0.86234
MSTRG.13153.1	1.937233	2.076623	5.475698	0.001999	0.069337	-0.86296
lnc-RDH13-4:2	-1.69712	2.166167	-5.47448	0.002001	0.069337	-0.86408
DLEU2:45	-1.02366	5.945453	-5.4741	0.002002	0.069337	-0.86443
T047118	-1.99977	2.273644	-5.4722	0.002005	0.069357	-0.86617
lnc-ZFHX2-2:1	1.660952	1.929625	5.471455	0.002006	0.069362	-0.86685
NONHSAT165037.1	-1.51561	2.375283	-5.46899	0.002011	0.069362	-0.86911
lnc-SERTAD2-10:1	-1.51408	2.157046	-5.46777	0.002013	0.069362	-0.87022
NONHSAT179012.1	1.239016	5.160987	5.465447	0.002017	0.069413	-0.87236
NONHSAT179071.1	-1.72011	2.108781	-5.46542	0.002017	0.069413	-0.87238
NONHSAT191823.1	-1.17723	1.683456	-5.46484	0.002018	0.069425	-0.87292
lnc-TEX264-1:1	-1.66688	2.042981	-5.46301	0.002021	0.069431	-0.87459
lnc-STK25-1:1	-1.19582	4.572315	-5.46285	0.002022	0.069431	-0.87474
NONHSAT180346.1	1.202953	2.557542	5.462035	0.002023	0.069431	-0.87549
NONHSAT152602.1	1.184496	2.990486	5.460015	0.002027	0.069431	-0.87734
NONHSAT172091.1	1.524949	5.469832	5.459898	0.002027	0.069431	-0.87745
lnc-ZBTB39-2:2	1.73496	2.154104	5.458548	0.002029	0.069431	-0.87869
NONHSAT177713.1	2.121919	2.51913	5.45827	0.00203	0.069431	-0.87894
lnc-PPIAL4G-4:17	-1.56515	3.033064	-5.45761	0.002031	0.06944	-0.87955
lnc-CHIC1-9:1	1.849612	3.013757	5.456511	0.002033	0.06945	-0.88056
ENST00000668625.1	-1.0546	1.758218	-5.45588	0.002034	0.06945	-0.88114
lnc-ESM1-5:1	-2.05187	2.496701	-5.45393	0.002037	0.06945	-0.88293
lnc-ZFAND5-2:1	2.224027	2.438394	5.452944	0.002039	0.06945	-0.88384
MSTRG.60986.1	1.884921	4.051672	5.4525	0.00204	0.06945	-0.88425
lnc-ZNF66-3:1	1.46658	3.010569	5.452414	0.00204	0.06945	-0.88433
lnc-KLRB1-12:8	-1.43603	3.251249	-5.45149	0.002042	0.06945	-0.88517
lnc-THYN1-5:1	1.014107	6.149449	5.451346	0.002042	0.06945	-0.88531

NONHSAT167081.1	1.527896	3.360786	5.451185	0.002042	0.06945	-0.88546
ENST00000436500.1	1.363588	5.590285	5.45059	0.002043	0.06945	-0.886
ENST00000668466.1	-1.50332	2.758882	-5.4505	0.002044	0.06945	-0.88608
lnc-LRCH1-9:1	1.643832	1.8991	5.449395	0.002046	0.069471	-0.8871
lnc-SLC25A35-2:1	-1.31638	4.462978	-5.44807	0.002048	0.069481	-0.88832
NONHSAT169812.1	-1.19098	6.005096	-5.44613	0.002051	0.069537	-0.89011
ENST00000555771.2	-1.16372	2.937398	-5.44491	0.002054	0.069569	-0.89122
ENST00000455848.1	1.283336	3.393621	5.444723	0.002054	0.069569	-0.8914
ENST00000557965.1	-1.36978	2.308701	-5.44325	0.002057	0.069636	-0.89276
ENST00000421851.1	-1.2565	3.452106	-5.44047	0.002062	0.069696	-0.89532
MSTRG.47961.1	-1.31504	3.816089	-5.4402	0.002062	0.069696	-0.89556
NONHSAT156709.1	1.641993	2.363246	5.43922	0.002064	0.069696	-0.89647
NONHSAT206835.1	1.237679	4.528331	5.439217	0.002064	0.069696	-0.89647
lnc-C14orf119-1:1	-1.16528	3.787053	-5.43811	0.002066	0.069717	-0.89749
NONHSAT187701.1	1.064424	1.730615	5.435967	0.00207	0.069736	-0.89946
ENST00000654444.1	-1.61905	4.041972	-5.4359	0.00207	0.069736	-0.89953
T239450	-1.5997	3.338849	-5.43382	0.002074	0.069839	-0.90145
NONHSAT210848.1	1.435627	2.04902	5.433456	0.002074	0.069839	-0.90178
lnc-ACMSD-3:1	-1.29601	1.833528	-5.43097	0.002079	0.069889	-0.90407
NR_110782	-1.17871	3.942306	-5.43027	0.00208	0.069889	-0.90472
lnc-FKBP5-1:2	1.209628	6.249867	5.429997	0.002081	0.069889	-0.90497
NONHSAT185026.1	1.677584	2.418176	5.428931	0.002083	0.069931	-0.90595
lnc-POMGNT1-2:1	1.680516	2.19796	5.423497	0.002093	0.069992	-0.91097
ENST00000450669.2	1.501629	1.803048	5.423164	0.002093	0.069992	-0.91127
lnc-CYBB-2:1	1.344215	4.211776	5.421193	0.002097	0.069992	-0.91309
ENST00000671681.1	1.140335	3.414826	5.421007	0.002097	0.069992	-0.91327
MSTRG.53632.17	1.885343	2.444396	5.420319	0.002099	0.069992	-0.9139
NONHSAT149293.1	1.16771	1.692104	5.420297	0.002099	0.069992	-0.91392
NONHSAT221753.1	1.762855	2.01116	5.420034	0.002099	0.069992	-0.91416
NONHSAT156287.1	1.557853	1.885098	5.418454	0.002102	0.07002	-0.91562
NONHSAT187468.1	1.317774	1.723476	5.41744	0.002104	0.070059	-0.91656
NONHSAT201104.1	1.863612	2.823067	5.416222	0.002106	0.070059	-0.91769
NONHSAT211192.1	1.075437	4.339545	5.415945	0.002107	0.070059	-0.91794
MSTRG.26213.1	1.818131	2.670033	5.41549	0.002108	0.070059	-0.91836
lnc-POTEB2-5:1	-1.26477	3.552888	-5.41483	0.002109	0.070059	-0.91898
NONHSAT189471.1	1.598773	1.840303	5.412396	0.002113	0.070113	-0.92123
NONHSAT198215.1	-1.07827	3.544624	-5.41182	0.002114	0.070113	-0.92176
NONHSAT182503.1	1.092128	1.605239	5.411526	0.002115	0.070113	-0.92203
NONHSAT212214.1	1.260142	7.266318	5.411333	0.002115	0.070113	-0.92221
MSTRG.40860.1	1.065438	4.87146	5.411159	0.002116	0.070113	-0.92237
MSTRG.42739.1	-1.39865	2.600494	-5.41059	0.002117	0.070114	-0.9229
MSTRG.49439.7	1.371541	3.282135	5.410157	0.002117	0.070117	-0.9233
MSTRG.43258.1	1.034798	4.202853	5.409497	0.002119	0.070135	-0.92391
lnc-RWDD4-5:1	1.62009	1.881734	5.406172	0.002125	0.070285	-0.92698
NONHSAT191743.1	2.09572	3.615095	5.406121	0.002125	0.070285	-0.92703
ENST00000550135.5	-1.46061	3.306672	-5.40495	0.002127	0.070316	-0.92811
lnc-SGCA-4:2	-1.04027	2.25174	-5.40435	0.002128	0.070316	-0.92867
lnc-ZFAT-11:1	-1.04082	2.241459	-5.40256	0.002132	0.070358	-0.93033

NONHSAT202365.1	1.209315	5.848947	5.395721	0.002145	0.070542	-0.93667
lnc-CYP4F22-5:1	-1.06356	6.4468	-5.3957	0.002145	0.070542	-0.93668
MSTRG.42808.1	-1.89809	2.838003	-5.39554	0.002145	0.070542	-0.93683
ENST00000657877.1	1.23893	1.672429	5.39429	0.002147	0.070597	-0.938
lnc-ZSCAN20-1:1	-1.04972	3.845688	-5.39346	0.002149	0.070615	-0.93877
NONHSAT186585.1	1.336079	6.907034	5.391769	0.002152	0.070671	-0.94033
NR_110840	1.157832	6.652268	5.391629	0.002152	0.070671	-0.94046
T204260	1.366961	3.22338	5.390601	0.002154	0.070707	-0.94142
MSTRG.13601.1	1.988599	5.171138	5.389136	0.002157	0.070707	-0.94278
MSTRG.62672.1	1.006683	5.268968	5.388832	0.002158	0.070707	-0.94306
MSTRG.25954.1	-1.1586	4.729587	-5.38865	0.002158	0.070707	-0.94323
NONHSAT197451.1	1.178096	1.705671	5.388477	0.002158	0.070707	-0.94339
lnc-POLR1D-1:2	-1.33549	2.082759	-5.38534	0.002164	0.070808	-0.9463
NONHSAT167452.1	1.628933	3.102659	5.385153	0.002165	0.070808	-0.94648
lnc-HAO2-7:1	1.444257	3.263219	5.382666	0.00217	0.070808	-0.94879
NONHSAT197461.1	1.220997	1.884263	5.382459	0.00217	0.070808	-0.94898
NONHSAT152445.1	2.071869	2.508585	5.381361	0.002172	0.070808	-0.95
lnc-C12orf40-2:1	1.073577	5.934736	5.381121	0.002173	0.070808	-0.95022
NONHSAT156234.1	1.012612	5.907754	5.38023	0.002174	0.070808	-0.95105
lnc-PMP22-4:1	1.350122	5.370982	5.380219	0.002174	0.070808	-0.95106
lnc-LLPH-6:1	-1.04205	5.142409	-5.38021	0.002174	0.070808	-0.95107
MSTRG.37935.1	1.49241	4.107694	5.378225	0.002178	0.070823	-0.95291
MSTRG.17336.1	-1.05755	2.33693	-5.37652	0.002181	0.070907	-0.9545
NONHSAT191694.1	1.222074	3.225683	5.374859	0.002185	0.070989	-0.95604
MSTRG.25713.4	-1.11225	4.240495	-5.37301	0.002188	0.071013	-0.95776
NONHSAT219661.1	-1.15908	8.954959	-5.37263	0.002189	0.071013	-0.95812
lnc-FARSB-7:1	-1.93632	2.551424	-5.36979	0.002194	0.071013	-0.96076
NONHSAT205149.1	1.415788	3.104163	5.369669	0.002195	0.071013	-0.96087
lnc-TTC21B-2:4	-1.43233	3.986838	-5.36957	0.002195	0.071013	-0.96097
NONHSAT158549.1	-1.02499	3.927137	-5.36547	0.002203	0.071013	-0.96478
NONHSAT200276.1	-2.00116	3.767584	-5.36526	0.002203	0.071013	-0.96498
MSTRG.20182.1	1.356069	5.280527	5.364842	0.002204	0.071013	-0.96537
lnc-POU4F3-3:1	-1.07208	4.617007	-5.36468	0.002204	0.071013	-0.96552
lnc-PROSER1-2:1	-1.18361	3.110691	-5.36421	0.002205	0.071013	-0.96596
lnc-MYOT-1:1	1.533249	2.084212	5.363151	0.002207	0.071013	-0.96695
MSTRG.28257.1	1.396845	7.578189	5.362919	0.002208	0.071013	-0.96716
NONHSAT206201.1	1.167664	5.127254	5.360348	0.002213	0.071059	-0.96956
lnc-KLHL31-10:1	1.600741	3.241912	5.357537	0.002218	0.071136	-0.97218
lnc-PATJ-2:1	-1.51034	5.759551	-5.35739	0.002219	0.071136	-0.97232
NONHSAT149315.1	-1.11946	3.58018	-5.3555	0.002223	0.07114	-0.97408
NONHSAT197590.1	1.011229	8.212559	5.354819	0.002224	0.07114	-0.97472
lnc-ALB-7:1	-1.78738	3.075145	-5.35336	0.002227	0.071142	-0.97608
ENST00000420867.1	1.825721	3.115332	5.351867	0.00223	0.071168	-0.97747
NONHSAT172704.1	-1.0042	4.052349	-5.35139	0.002231	0.071168	-0.97791
NONHSAT171316.1	1.167005	3.067175	5.350067	0.002233	0.071169	-0.97915
NONHSAT208849.1	1.393097	5.636644	5.349517	0.002234	0.071169	-0.97967
NONHSAT157193.1	1.460199	1.793478	5.349385	0.002235	0.071169	-0.97979
NR_110039	1.337828	4.508802	5.346521	0.00224	0.071197	-0.98246

ENST00000651492.1	1.036225	4.421793	5.34563	0.002242	0.071197	-0.9833
lnc-IGIP-2:2	-1.1884	4.028887	-5.34552	0.002242	0.071197	-0.9834
ENST00000623971.3	1.781051	2.580381	5.343542	0.002246	0.071225	-0.98525
NONHSAT158029.1	1.253404	3.864006	5.343078	0.002247	0.071229	-0.98568
NONHSAT157172.1	1.327169	4.208934	5.342771	0.002248	0.071229	-0.98597
NONHSAT188971.1	-1.20722	1.75848	-5.34109	0.002251	0.071286	-0.98754
MSTRG.23307.2	1.436886	3.482792	5.340809	0.002252	0.071286	-0.9878
NONHSAT184723.1	1.558463	2.888527	5.340257	0.002253	0.071295	-0.98832
lnc-ANKRD30BL-10:1	1.153567	1.675274	5.338379	0.002257	0.071329	-0.99008
lnc-UHRF1BP1L-4:1	1.432393	2.032094	5.337381	0.002259	0.07137	-0.99101
lnc-ATP6V1E2-10:4	1.390767	4.413165	5.334005	0.002265	0.071428	-0.99417
lnc-TMED10-1:1	-1.87605	3.52859	-5.33254	0.002268	0.071455	-0.99554
NONHSAT185726.1	-1.03755	1.616704	-5.33104	0.002271	0.071511	-0.99695
lnc-CNOT6-6:1	-1.80957	2.769466	-5.33015	0.002273	0.071511	-0.99778
lnc-CDH10-11:1	-1.89182	3.551845	-5.32919	0.002275	0.071512	-0.99868
lnc-VIT-3:1	-1.02526	11.32181	-5.32662	0.00228	0.071521	-1.00109
NONHSAT160182.1	1.26015	3.184377	5.326178	0.002281	0.071521	-1.0015
ENST00000454234.1	1.262684	3.51843	5.322889	0.002288	0.071581	-1.00459
lnc-HBEGF-2:8	2.479981	3.62353	5.321286	0.002291	0.071607	-1.00609
T265427	1.40625	2.006741	5.32101	0.002292	0.071607	-1.00635
lnc-ZEB2-1:14	-1.08068	8.833715	-5.32074	0.002292	0.071607	-1.0066
lnc-GADD45B-1:1	1.72435	2.441577	5.317287	0.0023	0.071719	-1.00984
lnc-ALG10B-3:2	2.422927	2.927926	5.315139	0.002304	0.071833	-1.01186
lnc-NUDCD1-1:1	-1.19479	5.853053	-5.31443	0.002305	0.071856	-1.01252
ENST00000523279.1	1.83547	5.285513	5.31174	0.002311	0.071941	-1.01505
MSTRG.57243.4	1.590069	3.091176	5.309749	0.002315	0.072024	-1.01692
ENST00000607953.1	-1.20525	5.12849	-5.30651	0.002322	0.072071	-1.01996
NONHSAT218260.1	1.44077	2.371826	5.304798	0.002325	0.072071	-1.02157
lnc-ZSCAN5B-2:1	-1.27033	2.074234	-5.30456	0.002326	0.072071	-1.0218
lnc-FCRLB-3:1	-1.33998	3.487613	-5.30323	0.002329	0.072112	-1.02305
lnc-TMEM232-2:1	1.624985	2.153849	5.296786	0.002342	0.072344	-1.02911
T006588	-1.17925	2.483699	-5.29656	0.002343	0.072344	-1.02933
NONHSAT190273.1	1.684213	2.361218	5.295813	0.002344	0.072348	-1.03003
T262074	1.855956	3.490245	5.295428	0.002345	0.072351	-1.03039
lnc-ZMAT3-5:1	-1.95885	3.595078	-5.29504	0.002346	0.072354	-1.03075
NONHSAT198574.1	-1.0683	5.580983	-5.29463	0.002347	0.072359	-1.03115
MSTRG.64748.4	-1.25208	3.30142	-5.29325	0.00235	0.072362	-1.03244
lnc-NPTX1-2:8	-1.26471	3.054982	-5.29318	0.00235	0.072362	-1.03251
ENST00000528607.1	-1.53114	2.65849	-5.29193	0.002352	0.072369	-1.03369
NONHSAT167524.1	-1.22932	2.111291	-5.28822	0.00236	0.072413	-1.03718
ENST00000451929.1	1.542051	2.50155	5.286469	0.002364	0.072466	-1.03883
NONHSAT212981.1	1.089209	1.639585	5.285427	0.002366	0.072466	-1.03982
MSTRG.45749.1	1.137807	3.453764	5.285149	0.002367	0.072466	-1.04008
ENST00000504735.1	1.008739	1.60038	5.284415	0.002368	0.072466	-1.04077
NONHSAT215235.1	1.374522	6.614903	5.284108	0.002369	0.072466	-1.04106
NONHSAT178914.1	1.612194	4.001314	5.280979	0.002376	0.072542	-1.04401
NONHSAT171956.1	-2.27879	3.481912	-5.28071	0.002376	0.072542	-1.04427
NONHSAT218909.1	2.322422	5.062798	5.280582	0.002376	0.072542	-1.04439

ENST00000558429.1	-1.90071	3.009656	-5.28046	0.002377	0.072542	-1.0445
ENST00000606959.1	-1.7364	2.978009	-5.27986	0.002378	0.072542	-1.04507
NONHSAT185733.1	-1.19724	1.89059	-5.27961	0.002378	0.072542	-1.04531
lnc-C12orf40-5:1	-1.2385	2.970877	-5.27832	0.002381	0.072548	-1.04653
NONHSAT207738.1	-1.21984	2.152127	-5.27802	0.002382	0.072548	-1.04681
T081667	2.129425	2.079464	5.277619	0.002383	0.072548	-1.04719
MSTRG.52173.1	-1.05446	1.633336	-5.2775	0.002383	0.072548	-1.0473
lnc-ZNF730-2:1	2.227424	2.347872	5.276604	0.002385	0.072584	-1.04815
NONHSAT194468.1	1.328185	4.183577	5.274471	0.002389	0.072615	-1.05016
lnc-NDE1-1:3	-1.2983	3.390337	-5.27379	0.002391	0.072615	-1.0508
lnc-PKDCC-1:1	1.506201	2.153666	5.271787	0.002395	0.072622	-1.0527
MSTRG.62453.1	1.0666	7.256615	5.271671	0.002395	0.072622	-1.05281
NONHSAT156527.1	1.154833	5.415408	5.268255	0.002403	0.072629	-1.05604
lnc-PCDH20-15:1	1.268437	3.421911	5.267067	0.002405	0.072629	-1.05716
lnc-TRMT2A-3:1	-1.60397	3.289717	-5.26595	0.002408	0.072629	-1.05822
NONHSAT170895.1	-1.22118	5.506798	-5.26567	0.002408	0.072629	-1.05848
NONHSAT189577.1	1.600902	3.534304	5.264637	0.002411	0.072629	-1.05946
lnc-CWF19L2-2:1	-1.02383	4.250671	-5.26304	0.002414	0.072629	-1.06097
NONHSAT155028.1	1.292404	4.93764	5.262986	0.002414	0.072629	-1.06102
NONHSAT193057.1	-1.07846	5.6767	-5.26293	0.002414	0.072629	-1.06107
MSTRG.12580.1	-1.64541	2.087146	-5.26204	0.002416	0.072629	-1.06191
MSTRG.47035.1	-1.26763	1.885148	-5.2609	0.002419	0.072629	-1.063
NONHSAT196564.1	1.004898	4.785159	5.260573	0.00242	0.072629	-1.0633
lnc-FAM174B-1:5	-1.65704	2.828988	-5.25926	0.002422	0.072629	-1.06455
NONHSAT179981.1	1.478674	2.024374	5.259092	0.002423	0.072629	-1.06471
ENST00000561486.1	-1.97825	2.640283	-5.25831	0.002424	0.072629	-1.06544
lnc-MED14-8:1	-1.12876	4.924833	-5.25813	0.002425	0.072629	-1.06562
NONHSAT187141.1	1.758741	4.003419	5.257777	0.002426	0.072629	-1.06595
lnc-SHC3-8:2	1.059629	3.730893	5.257622	0.002426	0.072629	-1.0661
lnc-TTC37-4:2	-1.30904	4.28987	-5.2555	0.002431	0.072704	-1.06811
LINC00477:3	1.10509	4.762297	5.255143	0.002431	0.072705	-1.06845
lnc-LHFPL4-4:1	1.014754	3.744087	5.25385	0.002434	0.072768	-1.06967
NONHSAT200446.1	1.920069	2.445335	5.25163	0.002439	0.072871	-1.07178
lnc-TIMM21-5:10	1.319307	2.03122	5.249819	0.002443	0.072968	-1.07349
ENST00000658651.1	-1.20042	2.445669	-5.2492	0.002444	0.072976	-1.07408
ENST00000421965.1	-1.2409	3.133724	-5.24515	0.002453	0.073028	-1.07793
lnc-SLC9A3R1-2:2	1.232844	8.92614	5.244693	0.002454	0.073028	-1.07836
lnc-GABPA-14:1	-1.0025	6.451322	-5.24376	0.002456	0.073028	-1.07924
lnc-CHRDL1-3:1	-1.75433	3.091258	-5.24374	0.002457	0.073028	-1.07927
MSTRG.58649.5	-1.00939	5.354253	-5.24335	0.002457	0.073028	-1.07963
NONHSAT170185.1	1.060941	5.326387	5.242546	0.002459	0.073059	-1.0804
NONHSAT220183.1	1.861292	2.437993	5.2406	0.002464	0.073074	-1.08224
lnc-COL28A1-1:18	1.513305	2.417958	5.238277	0.002469	0.073074	-1.08445
MSTRG.50749.2	1.538646	2.166574	5.238082	0.002469	0.073074	-1.08464
T035724	-1.23547	1.88934	-5.23687	0.002472	0.073096	-1.08578
lnc-GGTLC2-5:1	1.738805	2.500899	5.235871	0.002474	0.073096	-1.08674
lnc-LRR1-9:1	-1.22963	2.527432	-5.23541	0.002475	0.073096	-1.08718
lnc-ETV5-2:1	1.219124	6.405489	5.234185	0.002478	0.073096	-1.08834

NONHSAT159015.1	-1.29084	2.673125	-5.23294	0.002481	0.073115	-1.08953
NONHSAT182960.1	1.078336	1.588944	5.230441	0.002486	0.073198	-1.0919
MSTRG.60069.4	1.341048	4.285036	5.229734	0.002488	0.073198	-1.09257
NONHSAT224166.1	1.372041	3.690844	5.227201	0.002494	0.073249	-1.09498
T231552	1.048091	7.749944	5.227178	0.002494	0.073249	-1.095
MSTRG.33988.1	1.831849	2.393966	5.227005	0.002494	0.073249	-1.09517
NR_002950	-1.40661	6.151251	-5.2267	0.002495	0.073249	-1.09546
NONHSAT214429.1	1.07784	5.987084	5.225248	0.002498	0.073303	-1.09684
lnc-TGFBR2-3:3	1.265951	3.620896	5.223123	0.002503	0.07338	-1.09886
lnc-FAM43A-12:1	1.588046	4.38957	5.222399	0.002504	0.073388	-1.09955
lnc-DIP2B-1:1	-1.86033	2.48539	-5.22236	0.002504	0.073388	-1.09959
NONHSAT172975.1	-1.22485	2.303081	-5.21903	0.002512	0.073482	-1.10276
lnc-PKD2L1-1:3	1.187901	4.399174	5.217585	0.002515	0.073523	-1.10414
MSTRG.35845.53	-1.70026	2.803331	-5.21751	0.002516	0.073523	-1.10421
MSTRG.28178.1	1.818035	3.793029	5.213677	0.002524	0.073647	-1.10787
lnc-ANGPT4-1:1	1.2478	4.247341	5.212344	0.002527	0.073672	-1.10914
NONHSAT224461.1	-1.35021	1.77696	-5.21097	0.00253	0.073705	-1.11045
NONHSAT196155.1	2.538194	2.780639	5.210657	0.002531	0.073705	-1.11075
NONHSAT211129.1	1.26747	3.605606	5.210417	0.002532	0.073705	-1.11098
lnc-MNX1-6:1	-1.16664	3.094703	-5.21025	0.002532	0.073705	-1.11114
ENST00000598116.1	1.019817	2.993062	5.208274	0.002537	0.073782	-1.11302
NONHSAT210678.1	-1.61994	3.295351	-5.20782	0.002538	0.073783	-1.11345
lnc-TOP2B-5:1	-1.24449	4.780215	-5.20618	0.002541	0.073829	-1.11502
MSTRG.21380.10	-1.62086	5.904168	-5.20442	0.002546	0.073841	-1.1167
T069672	1.058395	4.316002	5.203462	0.002548	0.073883	-1.11762
lnc-ASPH-4:2	1.960511	5.750025	5.20254	0.00255	0.073924	-1.1185
NONHSAT157234.1	1.595632	2.669032	5.201411	0.002552	0.073939	-1.11958
ENST00000664643.1	1.199809	3.022364	5.201159	0.002553	0.073939	-1.11982
NONHSAT219233.1	1.242937	5.067866	5.200626	0.002554	0.073939	-1.12033
lnc-LRP12-10:1	-1.54362	1.855452	-5.20048	0.002555	0.073939	-1.12047
NONHSAT162834.1	1.457924	1.813329	5.200039	0.002556	0.073939	-1.12089
NONHSAT149319.1	-1.61858	3.502423	-5.19978	0.002556	0.073939	-1.12114
lnc-CYP7B1-7:1	1.043772	5.494751	5.198074	0.00256	0.073948	-1.12276
MSTRG.54805.1	1.689049	2.220814	5.195624	0.002566	0.074058	-1.12511
lnc-LY6E-2:2	-1.07262	5.412761	-5.19422	0.002569	0.074068	-1.12645
lnc-KLF12-5:1	-1.92937	3.296461	-5.19099	0.002577	0.074068	-1.12954
lnc-ANKRD42-5:1	-1.51938	4.716404	-5.18953	0.00258	0.074068	-1.13094
NONHSAT161601.1	1.94104	4.260673	5.189485	0.00258	0.074068	-1.13098
NONHSAT153736.1	1.122463	1.654189	5.188384	0.002583	0.074068	-1.13203
lnc-PTPN13-2:1	-1.58954	4.134387	-5.18816	0.002583	0.074068	-1.13225
NONHSAT161637.1	1.318534	1.764997	5.185647	0.002589	0.074068	-1.13466
ENST00000657349.1	1.567516	2.685299	5.1854	0.00259	0.074068	-1.13489
lnc-OR6B1-1:1	1.93627	2.826981	5.185088	0.002591	0.074068	-1.13519
NONHSAT215444.1	1.877526	4.208444	5.18229	0.002597	0.074089	-1.13787
NR_110036	1.473214	2.132161	5.181588	0.002599	0.074089	-1.13854
lnc-CCR7-2:1	1.226882	1.883901	5.180528	0.002601	0.074097	-1.13956
NONHSAT154946.1	2.031588	2.421341	5.177713	0.002608	0.074138	-1.14226
NONHSAT160754.1	-1.09426	2.34074	-5.17685	0.00261	0.074138	-1.14308

MSTRG.40553.1	1.040493	3.967716	5.17662	0.002611	0.074138	-1.14331
lnc-RCBTB1-2:1	-1.4284	4.321184	-5.17632	0.002611	0.074138	-1.14359
NONHSAT205781.1	1.822655	2.997019	5.174618	0.002615	0.074183	-1.14523
NONHSAT177004.1	1.785384	4.377352	5.173754	0.002618	0.074183	-1.14606
MSTRG.18807.6	1.105439	5.947712	5.173382	0.002618	0.074183	-1.14641
ENST00000657738.1	-1.37293	4.548631	-5.17273	0.00262	0.074184	-1.14704
lnc-ZNF648-6:1	1.246459	2.650602	5.171922	0.002622	0.074184	-1.14782
NR_110139	1.296885	4.391853	5.169388	0.002628	0.074194	-1.15025
NONHSAT221630.1	1.417355	1.75903	5.166276	0.002635	0.074289	-1.15324
lnc-LRRC49-16:1	-1.61339	2.154649	-5.16485	0.002639	0.074318	-1.15461
ENST00000513041.1	1.077702	9.055863	5.164623	0.002639	0.074318	-1.15483
BANCR:2	-1.35224	2.220108	-5.1617	0.002646	0.074363	-1.15764
lnc-OR6A2-1:1	-1.10107	4.753472	-5.16061	0.002649	0.074363	-1.15869
NONHSAT176818.1	1.057169	4.57932	5.159758	0.002651	0.074363	-1.1595
lnc-IL22-1:3	-1.1488	4.390858	-5.15938	0.002652	0.074363	-1.15986
NONHSAT158651.1	1.300516	1.751503	5.157627	0.002656	0.074391	-1.16155
lnc-PMAIP1-8:1	1.57052	3.82318	5.15743	0.002657	0.074391	-1.16174
ENST00000435832.1	2.060379	2.499753	5.156048	0.00266	0.074391	-1.16307
lnc-BRF1-47:1	-1.41306	1.811089	-5.15398	0.002665	0.074391	-1.16506
MSTRG.16968.1	1.193213	2.00472	5.153804	0.002666	0.074391	-1.16523
NONHSAT185509.1	-1.27399	1.731304	-5.15191	0.00267	0.074428	-1.16706
T281976	1.173912	4.622567	5.150785	0.002673	0.074428	-1.16814
MSTRG.39508.1	1.03749	5.319536	5.149187	0.002677	0.074428	-1.16967
lnc-MGARP-1:2	-1.17931	6.371954	-5.14759	0.002681	0.074428	-1.17121
lnc-CHRNA10-2:1	-1.00864	5.143162	-5.14745	0.002681	0.074428	-1.17135
ENST00000651844.1	-1.11646	2.739438	-5.14688	0.002683	0.074428	-1.1719
NONHSAT221241.1	1.573621	2.353656	5.146378	0.002684	0.074428	-1.17238
lnc-EML4-10:1	1.076603	3.471193	5.146288	0.002684	0.074428	-1.17247
NONHSAT205548.1	1.548286	1.881876	5.14504	0.002687	0.074434	-1.17367
NONHSAT167523.1	-1.1269	2.015671	-5.14504	0.002687	0.074434	-1.17367
MSTRG.19911.1	-1.26734	2.825517	-5.14221	0.002694	0.074545	-1.1764
lnc-MYCT1-1:1	1.01845	1.556245	5.13884	0.002702	0.074655	-1.17965
NONHSAT209233.1	2.097561	2.586424	5.133271	0.002716	0.074892	-1.18502
NONHSAT175052.1	-1.00044	4.056947	-5.13318	0.002716	0.074892	-1.18511
ENST00000658548.1	1.884452	7.122717	5.132005	0.002719	0.074892	-1.18625
MSTRG.61863.1	1.156611	1.658856	5.131929	0.00272	0.074892	-1.18632
lnc-CCT5-8:1	1.866734	3.255301	5.131182	0.002721	0.074892	-1.18704
ENST00000437621.6	1.2212	5.638095	5.127967	0.002729	0.074957	-1.19015
ENST00000655281.1	1.507838	5.841286	5.127782	0.00273	0.074957	-1.19033
lnc-GRHL2-8:3	1.28623	8.415763	5.127542	0.00273	0.074957	-1.19056
MSTRG.28406.1	1.045858	2.749855	5.126261	0.002734	0.074972	-1.1918
lnc-ANTXR2-1:5	1.551752	2.201159	5.125167	0.002736	0.074972	-1.19285
ENST00000603160.5	-1.48796	3.438491	-5.12503	0.002737	0.074972	-1.19299
NONHSAT192342.1	2.040877	2.845434	5.124651	0.002738	0.074972	-1.19335
ENST00000649638.1	1.328359	1.743656	5.12068	0.002748	0.075103	-1.19719
NONHSAT209176.1	1.468391	1.944729	5.119115	0.002752	0.075128	-1.19871
NONHSAT190525.1	-1.17433	1.949662	-5.11883	0.002752	0.075128	-1.19899
MSTRG.7329.1	-2.47049	3.071023	-5.11855	0.002753	0.075128	-1.19926

NONHSAT209959.1	1.352553	6.733649	5.116116	0.002759	0.075176	-1.20161
NONHSAT170668.1	-1.13425	5.129356	-5.11589	0.00276	0.075176	-1.20183
ENST00000541892.1	-1.72897	2.516511	-5.11487	0.002762	0.075176	-1.20281
NONHSAT170265.1	1.71804	2.481951	5.114323	0.002764	0.075176	-1.20335
NONHSAT218255.1	-1.27279	2.001699	-5.1132	0.002767	0.075205	-1.20444
lnc-ISG20L2-1:1	-1.7453	3.214414	-5.11315	0.002767	0.075205	-1.20448
NONHSAT196366.1	1.174693	4.689501	5.112706	0.002768	0.075207	-1.20491
NONHSAT214440.1	1.367226	1.879906	5.112012	0.00277	0.075212	-1.20558
ENST00000648995.1	-1.33987	2.697824	-5.11132	0.002771	0.075212	-1.20625
lnc-IL13RA2-7:1	-1.29174	1.964366	-5.10854	0.002779	0.075259	-1.20895
NONHSAT206246.1	-1.20823	1.710857	-5.10708	0.002782	0.075291	-1.21036
MSTRG.5963.1	1.748166	2.654888	5.106733	0.002783	0.075291	-1.2107
NONHSAT180349.1	-1.09353	1.636301	-5.10651	0.002784	0.075291	-1.21092
NONHSAT207712.1	1.083467	4.950808	5.104646	0.002789	0.075293	-1.21272
MSTRG.2479.1	1.882648	3.22068	5.103733	0.002791	0.075293	-1.21361
lnc-CCNH-6:1	-1.47239	3.359066	-5.10243	0.002794	0.075293	-1.21488
MSTRG.30374.1	1.232389	1.962099	5.102401	0.002794	0.075293	-1.2149
lnc-MANEA-6:1	1.561316	4.019856	5.102084	0.002795	0.075293	-1.21521
NONHSAT186409.1	-1.57405	2.081012	-5.10089	0.002798	0.075318	-1.21637
T138549	2.21113	3.249416	5.099801	0.002801	0.075353	-1.21742
ENST00000435271.1	-1.17075	4.983095	-5.09925	0.002802	0.075371	-1.21796
ENST00000448901.1	1.252433	1.695561	5.097579	0.002807	0.075407	-1.21958
NONHSAT184089.1	1.392881	3.535299	5.097144	0.002808	0.075417	-1.22
NONHSAT212180.1	-1.14782	3.560771	-5.09646	0.00281	0.075444	-1.22067
lnc-SIMC1-5:1	1.163895	4.854149	5.096069	0.002811	0.075452	-1.22105
NONHSAT154238.1	1.440469	2.930413	5.094191	0.002815	0.075481	-1.22287
NONHSAT198374.1	2.081724	2.14889	5.09355	0.002817	0.075481	-1.22349
NONHSAT210318.1	2.226662	3.164563	5.093324	0.002818	0.075481	-1.22371
lnc-SLC35F5-4:1	1.342301	5.746686	5.092614	0.00282	0.075481	-1.2244
NONHSAT209939.1	-1.10273	6.243183	-5.09099	0.002824	0.075481	-1.22598
NONHSAT179207.1	1.646181	5.753693	5.09067	0.002825	0.075481	-1.22629
LINC01782:9	1.46094	2.765476	5.090209	0.002826	0.075481	-1.22674
lnc-ANKRD60-1:3	2.280826	2.705135	5.090109	0.002826	0.075481	-1.22684
ENST00000434296.2	-1.6823	3.980772	-5.08912	0.002829	0.075508	-1.22779
NONHSAT153494.1	1.013117	3.61078	5.085096	0.002839	0.075695	-1.23171
lnc-LRMP-4:1	1.038782	7.126684	5.084068	0.002842	0.075705	-1.23271
lnc-CHRM5-4:1	1.533983	2.344601	5.082988	0.002845	0.075707	-1.23376
lnc-ZNF469-3:1	1.782106	4.066757	5.081485	0.002849	0.075707	-1.23523
NONHSAT161596.1	1.39041	1.778597	5.080322	0.002852	0.075707	-1.23636
NONHSAT167892.1	1.647346	3.100163	5.079972	0.002853	0.075707	-1.2367
NONHSAT197871.1	1.157644	2.500462	5.078549	0.002856	0.075707	-1.23808
NONHSAT196083.1	1.150009	1.619221	5.076184	0.002863	0.075752	-1.24039
lnc-NPBWR1-12:3	-1.25506	2.196147	-5.07477	0.002866	0.075781	-1.24177
lnc-SPATA16-5:1	-1.12528	3.744136	-5.07175	0.002874	0.075781	-1.2447
T017018	1.69377	2.494724	5.071416	0.002875	0.075781	-1.24503
lnc-IL17D-3:1	1.021477	5.772279	5.071395	0.002875	0.075781	-1.24505
SNHG17:24	-1.56388	3.088725	-5.07033	0.002878	0.075781	-1.24609
lnc-SERHL2-1:6	-1.37173	3.829393	-5.07017	0.002879	0.075781	-1.24625

ENST00000587011.1	-1.17686	2.204917	-5.07001	0.002879	0.075781	-1.2464
NONHSAT155289.1	1.355379	3.932457	5.06887	0.002882	0.075817	-1.24752
NONHSAT210011.1	1.421722	2.224494	5.067207	0.002886	0.075863	-1.24914
ENST00000483218.1	-1.62553	3.945337	-5.06547	0.002891	0.075863	-1.25083
NONHSAT178451.1	1.23738	2.043394	5.065361	0.002891	0.075863	-1.25094
NONHSAT151645.1	1.129095	2.261645	5.063489	0.002896	0.075943	-1.25277
NONHSAT166413.1	-1.01837	4.495346	-5.06299	0.002898	0.075943	-1.25326
lnc-ZNF708-8:1	1.398973	3.506979	5.062816	0.002898	0.075943	-1.25342
ENST00000660486.1	-2.39078	3.387132	-5.06218	0.0029	0.075949	-1.25404
NONHSAT222504.1	1.8708	3.784345	5.059896	0.002906	0.07605	-1.25627
NONHSAT161485.1	1.402739	3.528138	5.057992	0.002911	0.076105	-1.25813
NONHSAT172148.1	1.568084	4.251411	5.04911	0.002935	0.076452	-1.26681
MSTRG.2664.1	-1.1362	3.283097	-5.04781	0.002939	0.076484	-1.26809
NONHSAT224004.1	-1.32784	1.896075	-5.04604	0.002944	0.076484	-1.26982
ENST00000668332.1	-1.07042	1.656437	-5.04535	0.002946	0.076484	-1.2705
NONHSAT215405.1	-1.43903	4.322542	-5.04519	0.002946	0.076484	-1.27065
NONHSAT193103.1	1.189615	4.463535	5.044618	0.002947	0.076484	-1.27121
ENST00000613652.1	-1.28517	4.743419	-5.04283	0.002952	0.076535	-1.27296
NONHSAT167880.1	1.431181	3.48333	5.04248	0.002953	0.076535	-1.2733
lnc-MPZL3-2:1	-1.16809	5.274406	-5.04226	0.002954	0.076535	-1.27352
MSTRG.62298.4	1.489589	9.409909	5.038733	0.002964	0.076577	-1.27697
NONHSAT211759.1	1.960377	4.195678	5.03852	0.002964	0.076577	-1.27718
lnc-ETV5-1:1	1.037627	4.671096	5.038512	0.002964	0.076577	-1.27719
NONHSAT204799.1	2.084567	3.205938	5.038117	0.002965	0.076577	-1.27758
ENST00000504874.1	-1.61202	2.333416	-5.03558	0.002972	0.076585	-1.28006
MSTRG.2427.1	-1.41917	3.010272	-5.03535	0.002973	0.076585	-1.28029
lnc-FOXG1-20:1	1.53083	2.748658	5.03495	0.002974	0.076585	-1.28068
lnc-IRF8-1:2	1.190758	9.039412	5.03452	0.002975	0.076585	-1.2811
lnc-MCFD2-3:1	-1.09914	2.090515	-5.03353	0.002978	0.076585	-1.28207
lnc-SEC24C-7:1	-1.81436	3.522766	-5.03276	0.00298	0.076585	-1.28282
NONHSAT178104.1	1.378182	5.090809	5.032595	0.002981	0.076585	-1.28299
T061741	-1.75715	2.505673	-5.03172	0.002983	0.076585	-1.28384
lnc-GIMAP5-2:1	-1.69116	5.772695	-5.03135	0.002984	0.076585	-1.28421
NONHSAT213914.1	1.191187	3.901291	5.031114	0.002985	0.076585	-1.28444
MSTRG.42064.1	1.406891	3.367515	5.029097	0.00299	0.076662	-1.28642
ENST00000657713.1	-1.21211	3.120291	-5.0275	0.002995	0.076691	-1.28798
lnc-ALB-3:1	-1.53357	4.625289	-5.02735	0.002995	0.076691	-1.28813
NR_038965	1.047271	5.67279	5.027126	0.002996	0.076691	-1.28835
NONHSAT166899.1	1.827154	2.154565	5.027065	0.002996	0.076691	-1.28841
NONHSAT178948.1	1.631896	8.029268	5.026518	0.002997	0.07671	-1.28895
ENST00000657048.1	-1.89245	3.028337	-5.02542	0.003001	0.076722	-1.29003
lnc-OR9Q1-1:1	-1.61626	2.982217	-5.02445	0.003003	0.076722	-1.29098
lnc-PEPD-2:1	1.69094	2.308425	5.02444	0.003003	0.076722	-1.29099
lnc-PXDN-9:2	1.107873	3.331049	5.022746	0.003008	0.076734	-1.29265
ENST00000655813.1	-1.22847	4.191468	-5.02183	0.003011	0.076734	-1.29356
lnc-GRIK2-2:11	1.194331	3.535777	5.021086	0.003013	0.076734	-1.29428
lnc-SPANXB1-7:7	1.039271	4.943596	5.020437	0.003014	0.076734	-1.29492
ENST00000671641.1	-1.1729	4.746251	-5.02042	0.003015	0.076734	-1.29494

ENST00000515522.1	2.899727	3.352488	5.020198	0.003015	0.076734	-1.29516
lnc-ARID1B-5:1	-1.31416	4.16891	-5.01861	0.00302	0.076828	-1.29672
PDZRN3-AS1:8	1.297755	2.568091	5.017788	0.003022	0.076867	-1.29752
ENST00000670510.1	2.015868	2.497905	5.017135	0.003024	0.076895	-1.29817
NONHSAT206827.1	1.354613	7.333978	5.015968	0.003027	0.076944	-1.29931
MSTRG.11503.1	1.314811	2.208138	5.015903	0.003027	0.076944	-1.29938
lnc-C11orf91-4:1	2.429966	2.341727	5.014277	0.003032	0.076982	-1.30098
NONHSAT210699.1	1.500951	2.204915	5.014254	0.003032	0.076982	-1.301
lnc-SUPT6H-5:2	-1.64746	2.458232	-5.01409	0.003032	0.076982	-1.30116
ENST00000606809.1	1.069658	2.392427	5.014025	0.003033	0.076982	-1.30122
lnc-CSMD1-11:1	1.266906	3.9882	5.012801	0.003036	0.07705	-1.30243
lnc-PATE4-1:1	2.35276	2.739689	5.011715	0.003039	0.077089	-1.3035
lnc-HSFY1-4:1	-1.34476	2.7933	-5.00953	0.003045	0.077227	-1.30564
MSTRG.59825.1	2.195169	4.367116	5.006868	0.003053	0.077347	-1.30827
lnc-ECI2-11:1	-1.36281	3.274538	-5.00652	0.003054	0.077347	-1.30861
NONHSAT165224.1	-1.17999	2.299039	-5.00524	0.003057	0.077359	-1.30987
NONHSAT166245.1	1.34851	1.926739	5.003477	0.003062	0.077359	-1.31161
lnc-FAM53A-1:4	1.33646	4.834536	5.003152	0.003063	0.077359	-1.31193
ENST00000668417.1	-1.43209	3.414452	-5.00306	0.003064	0.077359	-1.31202
lnc-PLA1A-1:1	-1.02173	2.329316	-5.00254	0.003065	0.077359	-1.31253
lnc-CCNL1-5:1	-1.04764	6.759109	-5.00007	0.003072	0.077359	-1.31496
lnc-ENKUR-5:1	1.804215	1.93064	4.999938	0.003073	0.077359	-1.31509
lnc-SLC25A51-3:2	-1.25074	2.067957	-4.99977	0.003073	0.077359	-1.31526
lnc-SLC35G4-3:2	2.220455	3.116542	4.999591	0.003074	0.077359	-1.31543
lnc-EDDM13-1:2	1.649082	2.210775	4.999431	0.003074	0.077359	-1.31559
NONHSAT154407.1	2.115447	6.94986	4.997808	0.003079	0.077381	-1.31719
lnc-FBXO30-2:1	-1.08676	4.782496	-4.99773	0.003079	0.077381	-1.31727
ENST00000472514.1	-1.15651	1.914999	-4.99699	0.003081	0.077416	-1.318
lnc-PDE4D-4:1	-1.54463	3.978722	-4.99645	0.003083	0.077429	-1.31853
MSTRG.43658.5	2.005806	3.01036	4.995997	0.003084	0.077429	-1.31898
ENST00000592016.1	-1.00856	4.073068	-4.9946	0.003088	0.077474	-1.32036
NONHSAT200657.1	1.004145	4.722406	4.993302	0.003092	0.077528	-1.32164
ENST00000602618.1	-1.02395	4.514892	-4.99216	0.003095	0.077569	-1.32277
lnc-LYZL1-9:1	-1.03505	4.397669	-4.98974	0.003102	0.077632	-1.32515
lnc-C5orf46-1:1	-1.71016	2.476593	-4.98804	0.003107	0.07766	-1.32683
ENST00000653821.1	1.360376	4.13632	4.988039	0.003107	0.07766	-1.32683
ENST00000583067.1	-1.02046	5.708197	-4.98795	0.003107	0.07766	-1.32692
NONHSAT197136.1	-1.36421	2.567639	-4.98702	0.00311	0.077694	-1.32783
NONHSAT162078.1	1.023995	3.926114	4.986764	0.003111	0.077694	-1.32809
NONHSAT218131.1	2.384102	3.019388	4.985312	0.003115	0.077777	-1.32952
NONHSAT223999.1	1.867111	3.313475	4.984354	0.003118	0.077811	-1.33047
MSTRG.52068.11	-1.2051	1.895717	-4.98233	0.003124	0.077844	-1.33247
NR_015446	1.405289	5.506865	4.982317	0.003124	0.077844	-1.33248
lnc-DERA-6:1	-1.012	5.008321	-4.98132	0.003127	0.077853	-1.33347
NONHSAT194526.1	1.554633	6.599123	4.97976	0.003131	0.077915	-1.33501
lnc-GPR39-2:6	1.187072	3.221591	4.978551	0.003135	0.077966	-1.3362
NONHSAT161335.1	-1.54557	1.856435	-4.97651	0.003141	0.077979	-1.33822
ENST00000625026.1	-1.71098	2.59957	-4.975	0.003145	0.078004	-1.33972

NONHSAT164022.1	1.238459	4.343915	4.974329	0.003147	0.078004	-1.34038
ENST00000524017.1	-1.24186	4.161102	-4.97169	0.003155	0.078098	-1.34299
lnc-SNX14-6:1	-1.29688	6.56201	-4.97157	0.003155	0.078098	-1.34312
lnc-ENPP6-3:1	-1.304	2.458232	-4.97065	0.003158	0.078103	-1.34402
MSTRG.65187.1	-1.24534	5.249539	-4.96969	0.003161	0.078103	-1.34498
NONHSAT172727.1	1.795455	4.962494	4.969312	0.003162	0.078103	-1.34535
MSTRG.66845.1	-1.91627	3.012531	-4.9646	0.003176	0.078235	-1.35001
lnc-PDGFB-2:5	-1.48332	4.59795	-4.96446	0.003176	0.078235	-1.35015
LINC01239:6	1.599876	4.134261	4.964352	0.003177	0.078235	-1.35026
ENST00000607861.1	-1.20684	4.404285	-4.96285	0.003181	0.078235	-1.35175
NONHSAT202743.1	-1.75256	1.990792	-4.9628	0.003181	0.078235	-1.3518
MSTRG.65216.1	-1.80746	2.628899	-4.96257	0.003182	0.078235	-1.35203
MSTRG.65081.1	1.171935	6.15547	4.962557	0.003182	0.078235	-1.35204
lnc-GFPT1-2:1	1.758349	2.817136	4.9609	0.003187	0.07829	-1.35368
MSTRG.35445.1	-1.29709	4.306143	-4.9567	0.0032	0.078323	-1.35785
ENST00000657889.1	1.254774	3.567448	4.956372	0.003201	0.078323	-1.35817
NONHSAT161086.1	-1.55789	2.678122	-4.95573	0.003202	0.078323	-1.35881
ENST00000629214.1	1.830355	2.730738	4.954536	0.003206	0.078323	-1.35999
lnc-ADCY8-3:1	1.881789	3.994079	4.953426	0.003209	0.078323	-1.3611
lnc-THSD7A-8:1	-1.77845	2.719413	-4.95297	0.003211	0.078323	-1.36155
lnc-TXNDC12-3:1	-1.18253	4.983448	-4.95242	0.003212	0.078323	-1.36209
NONHSAT190089.1	1.03366	4.910331	4.951679	0.003215	0.078323	-1.36283
T359291	-1.66265	2.080604	-4.95087	0.003217	0.078323	-1.36363
NONHSAT188004.1	-1.62638	3.214115	-4.95078	0.003217	0.078323	-1.36372
NONHSAT181534.1	-1.09466	1.762848	-4.95003	0.00322	0.078323	-1.36447
ENST00000524252.1	-1.27567	4.315303	-4.94917	0.003222	0.078323	-1.36533
NONHSAT157332.1	1.686944	3.115472	4.948962	0.003223	0.078323	-1.36553
NONHSAT158307.1	2.394907	3.055252	4.948341	0.003225	0.078323	-1.36615
lnc-GEMIN8-2:1	1.030925	4.134752	4.948269	0.003225	0.078323	-1.36622
lnc-HS6ST3-3:1	1.64035	3.055454	4.947895	0.003226	0.078323	-1.36659
lnc-OLFM1-2:1	1.437019	1.742553	4.947645	0.003227	0.078323	-1.36684
NONHSAT188144.1	2.529162	3.527319	4.946649	0.00323	0.078323	-1.36783
MSTRG.52995.1	-1.285	1.853847	-4.94621	0.003231	0.078323	-1.36827
NONHSAT153470.1	1.095568	5.08671	4.946158	0.003231	0.078323	-1.36831
T064777	1.302552	4.903746	4.945939	0.003232	0.078323	-1.36853
ENST00000427471.1	-1.67717	2.245945	-4.94541	0.003234	0.078323	-1.36906
NONHSAT214949.1	1.227931	3.862433	4.945288	0.003234	0.078323	-1.36918
NONHSAT174543.1	-1.60491	2.790739	-4.94513	0.003234	0.078323	-1.36933
lnc-METTL14-1:3	1.015784	1.777962	4.943823	0.003238	0.078345	-1.37064
ENST00000664348.1	1.454346	3.426498	4.943491	0.003239	0.078348	-1.37097
lnc-PLEKHG7-7:2	1.094209	4.003155	4.940345	0.003249	0.078499	-1.37409
lnc-EXOC2-188:2	1.234019	4.105541	4.940201	0.00325	0.078499	-1.37424
MSTRG.48172.1	1.027897	5.032118	4.937373	0.003258	0.078614	-1.37705
NONHSAT201797.1	2.153016	3.014659	4.93588	0.003263	0.078696	-1.37854
MSTRG.38157.1	1.956372	2.501306	4.935541	0.003264	0.078696	-1.37887
lnc-CHIC1-8:1	-1.83128	3.664181	-4.93549	0.003264	0.078696	-1.37892
NONHSAT155238.1	1.377093	2.073199	4.93429	0.003268	0.07873	-1.38012
ENST00000421411.1	1.382121	4.860431	4.933598	0.00327	0.078743	-1.38081

NONHSAT222723.1	-1.00697	5.883825	-4.93041	0.00328	0.078835	-1.38399
lnc-ITGA6-3:1	2.833204	5.494449	4.93013	0.00328	0.078835	-1.38426
T203331	1.50536	1.870183	4.92951	0.003282	0.078835	-1.38488
MSTRG.3006.1	1.523213	1.962483	4.929384	0.003283	0.078835	-1.38501
LINC01128:89	-1.01139	4.001146	-4.92932	0.003283	0.078835	-1.38507
ENST00000596563.5	1.03156	3.27386	4.927536	0.003289	0.07888	-1.38685
ENST00000517623.1	1.195353	2.013041	4.926817	0.003291	0.078887	-1.38756
lnc-FGD4-1:1	-1.03654	4.462595	-4.92635	0.003292	0.078887	-1.38803
ENST00000666326.1	-1.74054	3.109996	-4.92578	0.003294	0.078892	-1.3886
ENST00000543475.1	-1.09887	1.870845	-4.92534	0.003295	0.078892	-1.38904
NONHSAT170356.1	-1.14671	1.957199	-4.92296	0.003303	0.078936	-1.39141
NONHSAT168808.1	1.794376	2.713419	4.9227	0.003304	0.078936	-1.39167
NR_110225	-1.41465	3.808853	-4.9226	0.003304	0.078936	-1.39177
ENST00000657719.1	1.159058	3.66265	4.921847	0.003306	0.078936	-1.39252
MSTRG.31439.1	1.384737	4.73729	4.920424	0.003311	0.078952	-1.39394
ENST00000521572.1	1.257543	2.542853	4.917747	0.003319	0.079041	-1.39661
lnc-CSPG5-1:1	-1.30644	3.371357	-4.91573	0.003325	0.079084	-1.39862
NONHSAT152483.1	1.067521	5.580595	4.912983	0.003334	0.079211	-1.40137
MSTRG.14624.1	1.547214	1.977313	4.910777	0.003341	0.079283	-1.40357
lnc-CDH12-5:1	1.245199	1.65678	4.909815	0.003344	0.079299	-1.40453
lnc-ACMSD-6:1	-1.78018	4.581437	-4.90611	0.003356	0.079456	-1.40824
ENST00000657024.1	1.584903	2.19882	4.905983	0.003356	0.079456	-1.40836
NONHSAT215714.1	1.393107	3.923287	4.90523	0.003359	0.079486	-1.40911
lnc-TUBGCP3-6:4	1.050992	7.274616	4.904771	0.00336	0.079486	-1.40957
MSTRG.70008.1	-1.05181	4.806513	-4.90433	0.003361	0.079486	-1.41001
ENST00000500381.2	-1.241	1.814634	-4.90412	0.003362	0.079486	-1.41022
lnc-DIRAS3-2:1	-1.03013	5.418632	-4.90327	0.003365	0.079507	-1.41108
NONHSAT169362.1	1.422275	10.76161	4.899768	0.003376	0.079606	-1.41458
NONHSAT192777.1	1.078722	1.75811	4.897836	0.003382	0.079696	-1.41651
lnc-IFT80-3:1	1.932763	3.019842	4.897192	0.003384	0.079726	-1.41716
NONHSAT199442.1	-1.12951	1.899464	-4.89683	0.003385	0.079735	-1.41752
ENST00000435984.1	1.269068	1.745827	4.89476	0.003392	0.079765	-1.41959
ENST00000549373.1	-1.09637	7.609791	-4.89472	0.003392	0.079765	-1.41963
ENST00000420268.1	-1.53513	2.981199	-4.89443	0.003393	0.079765	-1.41992
lnc-ANKRD20A2-19:1	-1.14974	4.810158	-4.89421	0.003394	0.079765	-1.42014
lnc-ZNF395-2:4	-1.40855	6.390385	-4.89363	0.003396	0.079782	-1.42072
lnc-TEX15-1:3	-2.17698	3.527348	-4.89277	0.003398	0.079782	-1.42159
ENST00000638795.1	-1.18539	2.12621	-4.89253	0.003399	0.079782	-1.42182
MSTRG.4181.1	-2.26336	2.25549	-4.89109	0.003404	0.079848	-1.42327
lnc-PTPN23-2:1	1.588057	1.83844	4.88981	0.003408	0.079878	-1.42455
MSTRG.37026.1	-1.03396	4.402563	-4.88928	0.00341	0.079878	-1.42508
NONHSAT201690.1	-1.79272	2.824833	-4.8887	0.003411	0.079903	-1.42566
LINC01638:16	2.025929	3.467438	4.888299	0.003413	0.079915	-1.42607
lnc-CLDN16-7:1	-1.7874	6.777002	-4.88751	0.003415	0.079938	-1.42685
ENST00000649562.1	1.412243	2.071724	4.886531	0.003419	0.079956	-1.42784
NONHSAT224060.1	1.779681	3.789111	4.885741	0.003421	0.079998	-1.42863
ENST00000668426.1	-1.26015	2.306327	-4.88337	0.003429	0.080104	-1.43101
ENST00000539305.1	1.263991	5.441673	4.883269	0.003429	0.080104	-1.43111

NONHSAT190244.1	-1.07137	4.661744	-4.88309	0.00343	0.080104	-1.43129
lnc-MZT2A-1:1	-1.18161	4.47376	-4.88216	0.003433	0.080121	-1.43222
NONHSAT158372.1	1.229478	6.173581	4.878816	0.003444	0.080175	-1.43558
NONHSAT189657.1	1.101863	3.056593	4.878345	0.003445	0.080175	-1.43605
lnc-PDHA1-3:1	1.056634	4.611896	4.878283	0.003445	0.080175	-1.43612
MSTRG.60057.2	2.263393	2.991886	4.877997	0.003446	0.080178	-1.4364
NONHSAT206436.1	1.593403	2.932847	4.876757	0.00345	0.080214	-1.43765
NONHSAT218967.1	1.298357	3.891788	4.876036	0.003453	0.080226	-1.43837
NONHSAT190308.1	1.411089	2.119842	4.875366	0.003455	0.080249	-1.43905
lnc-RIPPLY3-3:1	1.324872	1.771561	4.873401	0.003461	0.080308	-1.44102
lnc-COG6-5:1	2.276809	3.31052	4.872259	0.003465	0.080324	-1.44217
MSTRG.35265.1	1.608405	2.065041	4.872213	0.003465	0.080324	-1.44221
lnc-AASS-1:1	-1.40485	4.238351	-4.87116	0.003469	0.080327	-1.44327
T055857	1.015312	9.310205	4.868148	0.003479	0.080431	-1.4463
lnc-MATN1-1:1	1.167531	4.329786	4.86767	0.00348	0.08044	-1.44678
NONHSAT215169.1	-1.40144	2.854235	-4.86755	0.003481	0.08044	-1.4469
lnc-GALC-15:1	1.299769	1.671753	4.866298	0.003485	0.080443	-1.44816
NONHSAT155835.1	1.828062	3.281298	4.86515	0.003489	0.08049	-1.44932
lnc-NFASC-1:1	1.009173	4.961307	4.864299	0.003491	0.080495	-1.45017
lnc-ESM1-4:1	1.766451	5.044724	4.864186	0.003492	0.080495	-1.45029
ENST00000642657.1	-1.05836	2.442009	-4.86302	0.003496	0.080541	-1.45146
NONHSAT218061.1	1.205884	1.749855	4.862192	0.003498	0.080541	-1.45229
MSTRG.14663.1	1.074911	4.839676	4.857911	0.003513	0.080626	-1.4566
MSTRG.54072.1	1.436878	2.062884	4.856784	0.003516	0.080675	-1.45774
MSTRG.54225.2	1.183857	3.634497	4.856143	0.003519	0.080675	-1.45838
lnc-GRXCR2-1:1	-1.24952	4.218677	-4.8558	0.00352	0.080675	-1.45873
lnc-PGPEP1-4:1	1.319703	4.579146	4.854888	0.003523	0.080675	-1.45965
NONHSAT161481.1	1.400153	5.31832	4.854404	0.003524	0.080681	-1.46014
ENST00000431019.1	-1.18631	2.657061	-4.85367	0.003527	0.080693	-1.46087
NONHSAT167937.1	1.242547	6.092191	4.853219	0.003528	0.080693	-1.46133
NONHSAT188334.1	1.108518	5.527583	4.851685	0.003534	0.08072	-1.46288
NONHSAT191845.1	-1.2371	2.978588	-4.85085	0.003536	0.080733	-1.46372
ENST00000564193.1	1.340131	3.223721	4.848624	0.003544	0.080835	-1.46596
NONHSAT210143.1	1.266327	3.526271	4.84845	0.003545	0.080835	-1.46614
lnc-PTPN2-10:1	2.176617	2.673204	4.846987	0.003549	0.080863	-1.46762
lnc-B4GALT7-1:2	-1.30714	1.971248	-4.84569	0.003554	0.080871	-1.46892
ENST00000663213.1	1.598052	1.878547	4.84527	0.003555	0.080885	-1.46935
NONHSAT201829.1	1.001848	4.99173	4.844858	0.003557	0.080899	-1.46976
NONHSAT157099.1	-1.20112	4.937662	-4.8441	0.003559	0.080917	-1.47053
MSTRG.43953.1	1.537672	5.23995	4.844014	0.00356	0.080917	-1.47062
NONHSAT151324.1	1.326136	1.719933	4.84252	0.003565	0.080982	-1.47212
ENST00000638312.1	1.502083	1.888606	4.842356	0.003565	0.080982	-1.47229
lnc-CAMK1G-6:1	1.532754	2.0777	4.841139	0.003569	0.080985	-1.47352
NONHSAT154770.1	1.145752	4.872865	4.840618	0.003571	0.080989	-1.47404
NONHSAT171137.1	1.682684	2.421457	4.837698	0.003581	0.081022	-1.47699
NONHSAT205483.1	1.10825	3.198137	4.836163	0.003586	0.081101	-1.47855
lnc-NDNF-1:1	-1.00562	1.696698	-4.83497	0.00359	0.081101	-1.47976
lnc-BRF1-70:1	1.178168	3.596022	4.833726	0.003595	0.081122	-1.48101

lnc-C10orf105-5:1	1.173276	4.063704	4.833069	0.003597	0.081155	-1.48167
NONHSAT164867.1	-1.63613	2.365795	-4.83241	0.003599	0.081169	-1.48234
T359296	-1.58896	2.527018	-4.8313	0.003603	0.08122	-1.48347
T363553	1.695142	3.285445	4.830072	0.003607	0.081276	-1.4847
NONHSAT215158.1	1.251167	1.907232	4.829143	0.00361	0.081314	-1.48564
ENST00000639687.1	1.31789	2.289178	4.828913	0.003611	0.081314	-1.48588
lnc-SHOX2-8:1	-2.04291	3.80987	-4.82632	0.00362	0.081404	-1.4885
NONHSAT179202.1	1.525869	4.424855	4.826128	0.003621	0.081404	-1.48869
NONHSAT173439.1	1.971993	2.217443	4.825034	0.003625	0.081434	-1.4898
lnc-ATP23-8:1	1.01173	1.701963	4.823414	0.00363	0.081439	-1.49144
MSTRG.17540.1	1.044864	4.60182	4.822028	0.003635	0.081451	-1.49285
NONHSAT199139.1	2.409061	3.028706	4.822006	0.003635	0.081451	-1.49287
NONHSAT223695.1	1.365329	1.70608	4.821336	0.003637	0.081451	-1.49355
ENST00000510176.1	-1.79686	2.228841	-4.82119	0.003638	0.081451	-1.49369
NONHSAT157921.1	1.079915	5.656155	4.819938	0.003642	0.081481	-1.49496
NONHSAT169420.1	1.650471	2.636897	4.819504	0.003644	0.081481	-1.4954
NONHSAT218745.1	1.229404	3.513406	4.819444	0.003644	0.081481	-1.49546
NONHSAT192820.1	-1.43531	2.023566	-4.81936	0.003644	0.081481	-1.49555
MSTRG.2492.1	-1.52619	3.692284	-4.81901	0.003646	0.081481	-1.4959
lnc-C12orf42-6:1	1.410062	2.296612	4.81662	0.003654	0.081542	-1.49833
LINC02397:28	1.591984	2.245352	4.816321	0.003655	0.081542	-1.49863
NONHSAT175341.1	1.072834	5.518038	4.815379	0.003658	0.081554	-1.49958
ENST00000442260.1	1.176306	8.503987	4.815058	0.003659	0.081561	-1.49991
NONHSAT181505.1	1.698687	5.02342	4.814197	0.003662	0.081575	-1.50078
NONHSAT162394.1	1.256675	6.539618	4.813531	0.003665	0.081575	-1.50146
NONHSAT214459.1	-1.1575	2.561063	-4.81351	0.003665	0.081575	-1.50148
MSTRG.13586.1	-1.47975	1.877506	-4.81212	0.00367	0.081601	-1.50289
NONHSAT218914.1	-1.14533	1.910554	-4.81202	0.00367	0.081601	-1.50299
lnc-ALDH9A1-3:2	-1.07114	2.788991	-4.81056	0.003675	0.08168	-1.50448
lnc-IGF1R-1:1	1.616907	2.392786	4.810281	0.003676	0.08168	-1.50475
lnc-TBCCD1-2:1	1.986354	2.538045	4.809671	0.003678	0.08168	-1.50537
NONHSAT161281.1	2.076024	2.144033	4.807234	0.003687	0.081764	-1.50785
NONHSAT214868.1	1.706326	3.939823	4.806624	0.003689	0.081764	-1.50847
ENST00000602529.2	1.025051	5.160483	4.805259	0.003694	0.08177	-1.50985
lnc-AGMAT-1:2	1.510987	4.698218	4.80501	0.003695	0.081772	-1.5101
NONHSAT154177.1	1.306538	4.704441	4.80414	0.003698	0.081822	-1.51099
NONHSAT223150.1	-1.07613	3.531751	-4.80216	0.003705	0.08183	-1.513
ENST00000617867.1	-2.01183	3.260047	-4.8017	0.003707	0.08183	-1.51346
NONHSAT196979.1	1.098832	1.893197	4.801283	0.003708	0.08183	-1.51389
ENST00000423873.5	-1.06856	3.688141	-4.80109	0.003709	0.08183	-1.51409
MSTRG.1837.5	-1.48891	4.810335	-4.79952	0.003714	0.081845	-1.51568
NONHSAT156407.1	1.104771	2.900059	4.799412	0.003715	0.081845	-1.51579
ENST00000667077.1	1.578512	4.232556	4.799169	0.003716	0.081845	-1.51604
ENST00000526041.1	1.921446	3.775134	4.798022	0.00372	0.081845	-1.5172
lnc-ASAP3-1:1	1.121089	5.378674	4.79641	0.003725	0.081869	-1.51884
lnc-MCTP2-13:1	1.880411	4.366185	4.796021	0.003727	0.081869	-1.51924
lnc-MAFB-1:1	-1.72932	4.05863	-4.79547	0.003729	0.081895	-1.5198
NONHSAT180848.1	1.881042	2.790776	4.794889	0.003731	0.081903	-1.52039

NONHSAT190313.1	-1.11157	2.623855	-4.79475	0.003731	0.081903	-1.52053
NONHSAT205692.1	1.527013	3.257508	4.788722	0.003753	0.082258	-1.52666
NONHSAT152099.1	1.307233	2.360526	4.788372	0.003754	0.082258	-1.52702
lnc-HIST2H3PS2-8:1	1.103121	5.210053	4.78698	0.003759	0.082314	-1.52844
NR_002971	-1.34761	5.968783	-4.78586	0.003763	0.082364	-1.52957
NR_110093	1.24097	1.991962	4.785816	0.003764	0.082364	-1.52962
lnc-PRKN-9:4	1.165176	3.644553	4.785671	0.003764	0.082364	-1.52977
NONHSAT215675.1	1.156505	1.937283	4.784471	0.003768	0.082402	-1.53099
NONHSAT174859.1	1.284094	2.964624	4.782218	0.003777	0.082488	-1.53329
ENST00000599484.1	-1.02386	3.237005	-4.78206	0.003777	0.082488	-1.53344
lnc-TBX19-1:1	1.41902	7.016142	4.781711	0.003778	0.082488	-1.53381
NR_110764	1.588905	2.449631	4.781175	0.00378	0.082488	-1.53435
T275252	-1.02017	5.04813	-4.77911	0.003788	0.082566	-1.53646
lnc-FAM133B-3:1	-1.22223	3.786508	-4.77908	0.003788	0.082566	-1.53649
lnc-BAAT-3:3	1.202291	2.380249	4.777988	0.003792	0.082605	-1.5376
lnc-USP35-18:2	1.02432	2.842469	4.777378	0.003794	0.082605	-1.53822
NONHSAT177259.1	1.012325	1.545521	4.777148	0.003795	0.082605	-1.53846
ENST00000439186.1	-1.74468	2.591552	-4.7767	0.003797	0.082605	-1.53891
NONHSAT152985.1	1.184304	4.910446	4.775864	0.0038	0.082605	-1.53977
MSTRG.38782.1	1.048161	5.062638	4.774998	0.003803	0.082605	-1.54065
lnc-CCDC167-2:2	2.43381	4.864143	4.774781	0.003804	0.082605	-1.54087
MSTRG.51389.1	-1.25035	4.475566	-4.77471	0.003804	0.082605	-1.54094
NONHSAT208139.1	2.015451	2.707589	4.774551	0.003805	0.082605	-1.54111
ENST00000667988.1	1.011102	3.205617	4.774003	0.003807	0.082614	-1.54166
NONHSAT167914.1	2.268099	2.325946	4.771613	0.003815	0.082696	-1.5441
NONHSAT224354.1	1.720195	2.063596	4.771085	0.003817	0.082696	-1.54464
ENST00000595644.1	1.118849	4.188477	4.770971	0.003818	0.082696	-1.54476
NR_126010	1.83442	2.524755	4.768863	0.003825	0.082827	-1.54691
lnc-BTBD3-1:1	1.091437	2.707791	4.768252	0.003828	0.082832	-1.54753
NONHSAT222995.1	1.049698	6.540285	4.768136	0.003828	0.082832	-1.54765
lnc-SPANXN5-2:2	1.073514	4.399551	4.767768	0.00383	0.082832	-1.54803
NONHSAT155210.1	-1.07361	1.918746	-4.76769	0.00383	0.082832	-1.54811
NONHSAT153443.1	1.296385	7.103749	4.765815	0.003837	0.082858	-1.55002
lnc-JMJD7-PLA2G4B-2:24	-1.31682	4.794208	-4.76489	0.00384	0.082858	-1.55097
NONHSAT161973.1	1.113521	4.812605	4.764828	0.00384	0.082858	-1.55103
NONHSAT210886.1	1.009962	2.864212	4.763431	0.003846	0.082858	-1.55246
NONHSAT217840.1	1.984235	3.083406	4.76268	0.003848	0.082858	-1.55323
NONHSAT187627.1	1.323946	4.273571	4.762236	0.00385	0.082858	-1.55368
NONHSAT150595.1	1.229384	5.294524	4.760572	0.003856	0.082858	-1.55538
lnc-HAAO-7:2	1.794256	2.333077	4.760498	0.003856	0.082858	-1.55546
MSTRG.63786.1	-1.10738	3.225874	-4.76047	0.003857	0.082858	-1.55549
lnc-SPRR2D-2:1	1.2245	6.548972	4.759825	0.003859	0.082858	-1.55615
NONHSAT153462.1	-1.28267	2.885683	-4.75875	0.003863	0.082858	-1.55724
lnc-STBD1-5:2	-1.16573	8.190079	-4.7587	0.003863	0.082858	-1.5573
NONHSAT203765.1	1.380537	5.551612	4.75838	0.003864	0.082858	-1.55762
NONHSAT208330.1	1.286637	4.092295	4.758058	0.003866	0.082858	-1.55795
lnc-SLC25A46-2:1	1.262761	2.030189	4.755066	0.003877	0.083034	-1.56101

NONHSAT197277.1	-1.12568	2.288834	-4.75476	0.003878	0.083034	-1.56132
lnc-RGMB-4:1	-1.87525	2.6859	-4.75248	0.003886	0.083163	-1.56366
T354765	1.196474	3.134772	4.752397	0.003887	0.083163	-1.56374
NONHSAT203293.1	1.055109	5.612728	4.751104	0.003892	0.083187	-1.56507
lnc-KDM4A-1:1	-1.47033	3.819218	-4.74772	0.003904	0.083299	-1.56853
NONHSAT173969.1	1.739781	3.732127	4.747514	0.003905	0.083299	-1.56874
ENST00000554759.1	1.353179	4.746225	4.745771	0.003912	0.083387	-1.57053
MSTRG.49316.1	-1.10179	2.633784	-4.74427	0.003917	0.083448	-1.57206
MSTRG.45920.1	-1.52083	3.370924	-4.74423	0.003918	0.083448	-1.57211
NONHSAT179060.1	-1.50821	1.866808	-4.74247	0.003924	0.08353	-1.57392
NONHSAT205596.1	1.021379	1.890261	4.741489	0.003928	0.083579	-1.57492
NONHSAT161144.1	2.593148	3.078142	4.740719	0.003931	0.083579	-1.57571
NONHSAT196277.1	1.830128	4.002322	4.739468	0.003936	0.083623	-1.57699
lnc-CDH12-7:1	1.14711	1.900485	4.73935	0.003936	0.083623	-1.57711
NONHSAT205421.1	1.509373	2.191594	4.736361	0.003947	0.083701	-1.58018
lnc-DLX5-4:1	-1.23288	6.076387	-4.73628	0.003948	0.083701	-1.58026
lnc-CRIM1-3:1	-1.53853	4.322789	-4.73617	0.003948	0.083701	-1.58037
lnc-SNAI1-5:2	1.451606	6.970732	4.736024	0.003949	0.083701	-1.58052
lnc-KIAA1257-3:1	1.767744	4.743496	4.732751	0.003961	0.083874	-1.58388
ENST00000571113.1	-1.57569	3.505321	-4.73003	0.003972	0.08394	-1.58668
NONHSAT149360.1	-1.20025	3.650301	-4.72809	0.003979	0.084025	-1.58867
ENST00000433876.2	-1.77991	3.267965	-4.7279	0.00398	0.084025	-1.58887
MSTRG.7933.1	-1.4113	3.789448	-4.72647	0.003985	0.084099	-1.59033
NONHSAT221687.1	-1.4038	3.783316	-4.72582	0.003988	0.084106	-1.591
lnc-CHSY1-1:1	-1.1268	5.074297	-4.72552	0.003989	0.084113	-1.59131
lnc-KDM4B-3:1	2.068681	3.068343	4.724992	0.003991	0.084136	-1.59186
lnc-SNX30-2:1	-1.45699	3.913891	-4.72431	0.003994	0.084143	-1.59255
MSTRG.6138.1	-1.44822	2.486072	-4.7234	0.003997	0.084181	-1.5935
NONHSAT154603.1	-1.19747	2.815223	-4.72166	0.004004	0.084192	-1.59528
T262081	2.381022	3.344403	4.721232	0.004006	0.084192	-1.59572
NONHSAT215859.1	1.154256	4.42381	4.721038	0.004007	0.084192	-1.59592
MSTRG.51601.1	1.084705	4.582788	4.720299	0.004009	0.084192	-1.59668
ENST00000548359.1	2.049237	3.096645	4.720148	0.00401	0.084192	-1.59684
lnc-BRF1-37:1	1.610983	4.110723	4.719772	0.004011	0.084192	-1.59722
MSTRG.61914.1	-1.11572	5.013826	-4.71804	0.004018	0.084223	-1.59901
lnc-STK32C-6:1	1.091909	4.992366	4.717776	0.004019	0.084223	-1.59928
MSTRG.52951.1	1.260765	1.932513	4.715644	0.004028	0.084271	-1.60147
lnc-PLA2G4C-1:1	1.057872	3.452957	4.714586	0.004032	0.084288	-1.60256
lnc-HAND1-5:1	1.066428	6.20422	4.713281	0.004037	0.084328	-1.6039
T057556	2.038474	3.04417	4.712596	0.004039	0.084337	-1.60461
NONHSAT186078.1	1.656871	2.327063	4.711513	0.004044	0.084362	-1.60572
NONHSAT167831.1	1.971881	2.103055	4.711334	0.004044	0.084362	-1.60591
lnc-CCNA2-1:1	-2.05355	2.489019	-4.71094	0.004046	0.084377	-1.60632
NONHSAT196897.1	1.099724	3.818143	4.7106	0.004047	0.084387	-1.60667
NONHSAT196937.1	-1.23616	3.51286	-4.71015	0.004049	0.084391	-1.60713
lnc-AMFR-5:1	-1.49978	2.689447	-4.70954	0.004051	0.084422	-1.60775
ENST00000501178.2	1.493486	3.811414	4.709175	0.004053	0.08443	-1.60813
NONHSAT188024.1	1.215604	1.680936	4.708855	0.004054	0.08443	-1.60846

lnc-ABCB11-1:1	-1.45252	5.371286	-4.70825	0.004057	0.08443	-1.60908
lnc-RNF6-5:1	-1.18485	6.186779	-4.70758	0.004059	0.08446	-1.60977
ENST00000649670.1	-1.47616	3.644059	-4.70691	0.004062	0.084461	-1.61047
lnc-SYNDIG1-3:3	1.440474	5.612263	4.705844	0.004066	0.084513	-1.61157
MSTRG.45338.1	1.800327	3.201095	4.705665	0.004067	0.084513	-1.61175
NONHSAT206283.1	-1.39036	2.044726	-4.70413	0.004073	0.084538	-1.61334
MSTRG.20108.1	1.281436	1.772364	4.700398	0.004088	0.084716	-1.61718
lnc-ANKRD36-13:1	1.216415	5.001798	4.700234	0.004088	0.084716	-1.61735
NONHSAT151360.1	1.438985	5.202096	4.699433	0.004092	0.084716	-1.61818
NONHSAT218900.1	-1.2752	3.404272	-4.6994	0.004092	0.084716	-1.61821
lnc-WDR60-9:1	-1.10541	2.494733	-4.69561	0.004107	0.084855	-1.62213
lnc-TSG101-2:1	-1.04994	4.143787	-4.69513	0.004109	0.084855	-1.62262
ENST00000508823.1	1.08743	2.889024	4.694779	0.00411	0.084855	-1.62298
NONHSAT156576.1	1.09127	1.605159	4.693903	0.004114	0.084855	-1.62389
NONHSAT188158.1	1.118609	3.469952	4.6936	0.004115	0.084855	-1.6242
lnc-TPT1-2:1	-1.51485	2.628921	-4.6933	0.004116	0.084855	-1.62451
MSTRG.57993.1	1.393195	1.749552	4.693174	0.004117	0.084855	-1.62464
NONHSAT158551.1	1.016837	5.118524	4.686931	0.004142	0.08508	-1.63109
lnc-IMP4-11:1	-1.89914	2.868413	-4.68681	0.004142	0.08508	-1.63122
lnc-BMP15-1:5	1.257489	4.564103	4.686469	0.004144	0.085091	-1.63157
MSTRG.48177.1	-1.88446	3.115527	-4.68501	0.004149	0.08516	-1.63308
lnc-ARHGAP20-11:2	1.817857	1.920923	4.680536	0.004168	0.085342	-1.63771
MSTRG.54591.1	-1.77714	3.288593	-4.68032	0.004168	0.085342	-1.63793
T226026	1.958442	2.471617	4.679437	0.004172	0.085381	-1.63884
ENST00000429151.2	-1.4299	2.303964	-4.67831	0.004177	0.08544	-1.64001
lnc-GLP1R-4:1	-1.24071	4.968261	-4.67737	0.00418	0.085447	-1.64098
ENST00000644669.1	-1.62187	2.522696	-4.67618	0.004185	0.085508	-1.64221
T268978	1.377435	1.733901	4.674635	0.004192	0.085525	-1.64382
MSTRG.54666.1	1.07294	1.897026	4.674392	0.004193	0.085525	-1.64407
NONHSAT167801.1	1.062538	4.500046	4.674176	0.004194	0.085525	-1.64429
lnc-C8orf76-2:1	-1.57779	2.226078	-4.6734	0.004197	0.08554	-1.6451
lnc-TTC25-4:1	-1.48317	4.165453	-4.67147	0.004205	0.085592	-1.6471
NONHSAT212095.1	2.333899	2.413879	4.671357	0.004205	0.085592	-1.64721
ENST00000501708.1	-1.59518	2.978992	-4.67126	0.004205	0.085592	-1.64731
lnc-DDX18-6:1	1.337787	1.768223	4.669375	0.004213	0.085676	-1.64927
ENST00000550177.2	1.086649	3.721741	4.66906	0.004215	0.085676	-1.64959
ENST00000427387.1	1.026659	4.580442	4.666715	0.004224	0.08572	-1.65203
NONHSAT208894.1	1.623779	7.638886	4.666478	0.004225	0.08572	-1.65227
ENST00000576808.1	1.646889	5.358873	4.665024	0.004231	0.08579	-1.65378
lnc-SNX19-4:1	1.102991	7.602929	4.664189	0.004235	0.085843	-1.65465
NONHSAT187568.1	-1.61655	2.40673	-4.66298	0.00424	0.085893	-1.6559
ENST00000624879.1	-1.08341	4.774768	-4.66229	0.004243	0.085917	-1.65662
NR_110201	2.287798	3.383323	4.661028	0.004248	0.085953	-1.65793
ENST00000534505.1	1.359655	3.684978	4.660439	0.00425	0.085982	-1.65854
MSTRG.72588.1	-1.04597	3.641335	-4.6574	0.004263	0.086121	-1.66169
NONHSAT220915.1	1.170109	1.940319	4.654954	0.004273	0.086258	-1.66423
ENST00000525548.1	-1.23767	3.575054	-4.65454	0.004275	0.086259	-1.66467
NONHSAT158550.1	1.024304	4.746795	4.653778	0.004278	0.086261	-1.66546

lnc-SPC24-1:1	-1.23626	3.838923	-4.65313	0.004281	0.086274	-1.66612
NONHSAT160149.1	1.360574	5.970747	4.651365	0.004288	0.086312	-1.66796
ENST00000531609.1	-1.84035	2.376369	-4.65128	0.004289	0.086312	-1.66806
lnc-NR5A2-6:1	-1.09878	3.051852	-4.65062	0.004291	0.086312	-1.66874
NONHSAT176820.1	1.877204	2.47968	4.649903	0.004294	0.086312	-1.66948
lnc-GNAQ-11:1	-1.56141	2.26161	-4.64922	0.004297	0.086312	-1.67019
NONHSAT161066.1	-1.13492	2.374251	-4.64869	0.004299	0.086323	-1.67075
MSTRG.62465.1	-1.26589	1.674798	-4.64694	0.004307	0.086399	-1.67256
NONHSAT155026.1	1.638217	6.634304	4.645947	0.004311	0.0864	-1.6736
NONHSAT222549.1	1.358868	4.502011	4.645617	0.004312	0.086411	-1.67394
lnc-NUDT4-2:1	-1.07229	2.460502	-4.64298	0.004324	0.086515	-1.67668
T297794	-1.54127	2.716031	-4.64167	0.004329	0.086515	-1.67805
NONHSAT223098.1	1.405418	3.732884	4.641626	0.004329	0.086515	-1.67809
ENST00000522189.1	1.183747	4.053474	4.641411	0.00433	0.086515	-1.67832
NONHSAT221953.1	1.09112	4.775408	4.641021	0.004332	0.086515	-1.67872
ENST00000445589.1	2.350136	5.418035	4.638857	0.004341	0.086573	-1.68098
MSTRG.41122.1	2.39603	3.57042	4.63864	0.004342	0.086574	-1.6812
lnc-MAP3K9-12:1	-1.34169	3.540396	-4.6371	0.004349	0.086671	-1.68281
NONHSAT162129.1	1.425617	2.764188	4.636843	0.00435	0.086674	-1.68308
lnc-PARP8-6:1	-1.05183	2.85905	-4.63502	0.004357	0.08668	-1.68497
MSTRG.13771.4	1.645959	1.909739	4.634956	0.004358	0.08668	-1.68504
lnc-ZNF486-3:1	-1.27233	3.351297	-4.63465	0.004359	0.08668	-1.68536
lnc-LGALS16-1:1	-1.41116	2.439497	-4.63424	0.004361	0.08668	-1.68579
NONHSAT193880.1	1.619716	3.48381	4.634036	0.004362	0.08668	-1.686
MSTRG.63485.1	-1.30281	2.612499	-4.63403	0.004362	0.08668	-1.68601
ENST00000569618.1	-2.01998	2.595637	-4.63398	0.004362	0.08668	-1.68606
lnc-BCL10-1:1	-1.08654	5.326873	-4.63355	0.004364	0.08668	-1.68651
ENST00000651527.1	1.499327	2.233208	4.633486	0.004364	0.08668	-1.68657
ENST00000658344.1	1.236117	2.1213	4.633349	0.004365	0.08668	-1.68672
lnc-GAA-2:1	-1.17573	4.917373	-4.63263	0.004368	0.08671	-1.68747
T189064	-1.17413	1.875672	-4.63217	0.00437	0.086732	-1.68795
lnc-SMPDL3A-2:1	1.797634	2.412248	4.630454	0.004377	0.086827	-1.68973
NONHSAT203517.1	-1.16572	4.277957	-4.62877	0.004384	0.08684	-1.69149
lnc-LEKR1-1:9	-1.29665	2.474816	-4.62875	0.004384	0.08684	-1.69151
ENST00000657726.1	-1.11679	1.647191	-4.62786	0.004388	0.08684	-1.69244
ENST00000531549.1	-1.06943	4.18107	-4.62756	0.004389	0.08684	-1.69275
NONHSAT209573.1	1.17297	1.900358	4.627537	0.00439	0.08684	-1.69278
lnc-SLC6A14-1:1	1.535249	3.107844	4.627534	0.00439	0.08684	-1.69278
T275025	-1.02562	2.707722	-4.62744	0.00439	0.08684	-1.69288
NONHSAT157929.1	2.119658	3.357681	4.625463	0.004399	0.086894	-1.69494
lnc-OR51B4-3:1	-2.34041	5.920381	-4.62388	0.004405	0.086966	-1.6966
NONHSAT187455.1	1.26187	3.752053	4.623834	0.004406	0.086966	-1.69664
LINC00052:6	1.021997	3.686147	4.622357	0.004412	0.087058	-1.69818
T172028	-1.05621	3.833792	-4.62105	0.004418	0.087084	-1.69954
NONHSAT219152.1	1.410606	3.551339	4.618177	0.00443	0.087246	-1.70255
MSTRG.62257.1	1.449139	1.780833	4.616583	0.004437	0.087315	-1.70421
lnc-C16orf95-2:20	-1.09096	3.736407	-4.61555	0.004442	0.087324	-1.70529
NONHSAT165148.1	1.4394	3.926639	4.615486	0.004442	0.087324	-1.70536

NONHSAT170031.1	1.873831	2.754966	4.614154	0.004448	0.087405	-1.70675
MSTRG.32971.1	-1.31691	2.921839	-4.61323	0.004452	0.087446	-1.70772
NONHSAT223760.1	-1.80744	2.716022	-4.61308	0.004452	0.087446	-1.70788
NONHSAT217052.1	-1.25858	1.724297	-4.6126	0.004455	0.087465	-1.70837
MSTRG.43853.1	1.397753	4.207835	4.612237	0.004456	0.087465	-1.70876
ENST00000470739.1	1.410074	2.052502	4.61218	0.004456	0.087465	-1.70882
NONHSAT153104.1	2.300845	2.696999	4.611435	0.00446	0.087465	-1.7096
NONHSAT153524.1	-1.05505	5.788072	-4.61131	0.00446	0.087465	-1.70973
lnc-PPP2R2A-5:1	1.397839	1.722258	4.609401	0.004469	0.087465	-1.71172
lnc-KMT2E-1:9	-2.04621	2.932617	-4.60929	0.004469	0.087465	-1.71184
lnc-TRAPPC12-6:1	1.711798	2.571048	4.608524	0.004473	0.087465	-1.71264
NONHSAT214398.1	-1.17471	3.903101	-4.60758	0.004477	0.087523	-1.71363
NONHSAT185531.1	1.191138	2.693826	4.606237	0.004483	0.087594	-1.71503
NONHSAT210389.1	1.108243	5.977421	4.603917	0.004493	0.087744	-1.71746
lnc-SPAG16-7:5	-1.12663	3.463571	-4.60139	0.004504	0.087898	-1.72011
NONHSAT187525.1	-1.06559	1.629757	-4.59938	0.004513	0.087975	-1.72222
ENST00000663787.1	-1.73294	2.658863	-4.5987	0.004516	0.087975	-1.72292
lnc-REL-6:3	-1.19943	3.198864	-4.59836	0.004518	0.087988	-1.72329
MSTRG.41590.10	1.196889	4.340442	4.596368	0.004526	0.088037	-1.72537
NONHSAT154279.1	1.059693	6.658138	4.594841	0.004533	0.088101	-1.72697
lnc-NGRN-1:1	1.542904	2.443873	4.594594	0.004534	0.088101	-1.72723
T049236	1.555748	2.373197	4.593272	0.00454	0.088141	-1.72862
lnc-RBM15-1:1	1.413717	3.482586	4.591551	0.004548	0.088224	-1.73042
NONHSAT185648.1	-1.05796	4.408109	-4.59134	0.004549	0.088224	-1.73064
NONHSAT200333.1	1.264149	6.856943	4.590763	0.004552	0.088231	-1.73125
lnc-TMEM242-6:1	-1.9649	3.834668	-4.59068	0.004552	0.088231	-1.73134
MSTRG.72460.2	-1.25915	3.285955	-4.58984	0.004556	0.088254	-1.73222
ENST00000448887.1	1.388683	2.099558	4.587604	0.004566	0.088345	-1.73456
lnc-PLGRKT-4:1	1.123288	2.989857	4.587054	0.004568	0.088345	-1.73514
MSTRG.46671.1	1.093205	1.945936	4.586949	0.004569	0.088345	-1.73525
NONHSAT223743.1	1.129732	4.545583	4.586141	0.004572	0.088345	-1.7361
NONHSAT187988.1	-1.52745	2.33051	-4.58569	0.004574	0.088345	-1.73658
NONHSAT200395.1	1.212087	3.094674	4.585426	0.004576	0.088345	-1.73685
lnc-PPP1R3B-7:1	-1.24385	1.817977	-4.58459	0.004579	0.08837	-1.73773
lnc-CDH6-4:1	1.288564	1.692362	4.58438	0.00458	0.08837	-1.73795
NONHSAT183785.1	2.01717	4.399517	4.583438	0.004585	0.088393	-1.73894
lnc-AAGAB-3:4	-1.79515	2.394026	-4.58302	0.004587	0.088393	-1.73938
lnc-SEC61G-9:1	1.260012	1.689127	4.582764	0.004588	0.088393	-1.73965
lnc-TP53TG3-9:2	-1.07161	5.378851	-4.58272	0.004588	0.088393	-1.73969
NONHSAT169877.1	1.078629	4.929836	4.581587	0.004593	0.088393	-1.74088
NONHSAT205713.1	2.761208	3.621397	4.580645	0.004597	0.088414	-1.74187
T271565	-2.29226	2.794415	-4.58042	0.004598	0.088417	-1.74211
NONHSAT162508.1	1.333412	5.06687	4.579523	0.004602	0.088479	-1.74305
ENST00000508038.1	1.470696	2.068432	4.578192	0.004609	0.0885	-1.74445
MSTRG.24013.1	-1.3681	1.855671	-4.57778	0.00461	0.0885	-1.74488
NONHSAT219556.1	-1.09596	3.978683	-4.57755	0.004611	0.0885	-1.74513
lnc-TNKS-2:1	1.924571	2.42472	4.576427	0.004617	0.088562	-1.7463
lnc-KLF4-12:1	-1.03552	2.529343	-4.57316	0.004632	0.088627	-1.74974

lnc-IDH3B-1:2	1.376199	4.148582	4.572974	0.004632	0.088627	-1.74994
NONHSAT185628.1	1.471248	2.098963	4.572742	0.004633	0.088627	-1.75018
lnc-DDX1-8:1	-1.11091	3.059871	-4.57165	0.004638	0.088696	-1.75132
MSTRG.30165.1	1.222723	1.720917	4.57148	0.004639	0.088696	-1.75151
NONHSAT188084.1	-1.00647	4.952533	-4.57043	0.004644	0.088708	-1.75262
NONHSAT196742.1	1.442239	2.019945	4.570269	0.004645	0.088708	-1.75278
ENST00000657275.1	-1.16506	1.772231	-4.57024	0.004645	0.088708	-1.75281
NONHSAT217880.1	1.257555	1.731246	4.570077	0.004646	0.088708	-1.75298
ENST00000668852.1	-1.91764	2.457615	-4.56911	0.00465	0.088753	-1.754
lnc-UBE2L5-5:1	1.25797	4.039001	4.568869	0.004651	0.088753	-1.75426
NONHSAT190035.1	1.116628	3.854715	4.568462	0.004653	0.088753	-1.75468
ENST00000669800.1	1.019907	4.378646	4.567748	0.004656	0.088754	-1.75544
NONHSAT191612.1	-1.54681	3.192262	-4.56757	0.004657	0.088754	-1.75563
NONHSAT211031.1	-1.1121	4.319548	-4.56694	0.00466	0.088762	-1.75629
NONHSAT171233.1	1.687449	2.534986	4.566332	0.004663	0.088762	-1.75693
ENST00000649987.1	-1.89678	3.309726	-4.56519	0.004668	0.088813	-1.75813
lnc-BCOR-12:2	-1.06695	2.081407	-4.56326	0.004677	0.088889	-1.76016
lnc-MSMO1-1:1	-1.32195	3.730716	-4.5616	0.004685	0.088932	-1.76191
lnc-HES1-9:1	1.109271	1.79951	4.55868	0.004699	0.089052	-1.76499
lnc-SHISA6-2:1	-1.18825	3.474236	-4.55521	0.004715	0.089216	-1.76865
lnc-DPPA4-2:1	-1.30729	2.205277	-4.55458	0.004718	0.089216	-1.76932
ENST00000565366.1	1.091365	2.874112	4.554401	0.004719	0.089216	-1.7695
T256976	1.577393	2.167472	4.553997	0.00472	0.089216	-1.76993
ENST00000521501.1	-1.69397	2.874626	-4.55342	0.004723	0.089216	-1.77053
NONHSAT198089.1	1.929277	2.493728	4.553172	0.004724	0.08922	-1.7708
NONHSAT214905.1	1.319083	4.208104	4.552301	0.004728	0.089249	-1.77172
lnc-TMC6-7:1	3.093041	3.964056	4.550272	0.004738	0.089329	-1.77386
NONHSAT198371.1	1.33834	3.916724	4.550142	0.004739	0.089329	-1.77399
MSTRG.56542.1	1.26604	3.963273	4.548356	0.004747	0.089432	-1.77588
lnc-STX4-2:1	1.729484	2.158601	4.547758	0.00475	0.089435	-1.77651
MSTRG.14462.1	-1.39753	3.062264	-4.54624	0.004757	0.089503	-1.77811
lnc-SLITRK1-7:1	1.755944	2.349205	4.545004	0.004763	0.089555	-1.77942
NONHSAT149085.1	2.427589	3.160478	4.544879	0.004763	0.089555	-1.77955
NONHSAT201372.1	-1.45837	3.752081	-4.5444	0.004766	0.089567	-1.78006
NONHSAT218069.1	1.104722	5.425836	4.54212	0.004776	0.089603	-1.78247
NONHSAT205571.1	1.217958	6.799775	4.538632	0.004793	0.089795	-1.78615
ENST00000655828.1	1.013953	5.334018	4.537101	0.0048	0.089868	-1.78777
lnc-MICA-7:1	1.563283	4.786914	4.534453	0.004813	0.089955	-1.79058
NONHSAT159023.1	1.235609	5.590965	4.533173	0.004819	0.090011	-1.79193
lnc-GFRA2-1:1	1.591729	1.80938	4.533112	0.00482	0.090011	-1.79199
ENST00000567234.1	1.115954	4.208698	4.532707	0.004821	0.090011	-1.79242
NONHSAT170016.1	1.589862	2.242546	4.531131	0.004829	0.090011	-1.79409
lnc-UTS2-10:2	1.124514	6.631249	4.531124	0.004829	0.090011	-1.7941
lnc-SAP30-1:2	1.80693	2.24592	4.530483	0.004832	0.090011	-1.79478
NONHSAT216652.1	1.106577	4.541642	4.526616	0.004851	0.090159	-1.79887
ENST00000411427.3	-1.19041	6.508979	-4.52548	0.004856	0.090221	-1.80007
lnc-RAD23B-12:1	3.704826	4.60863	4.525167	0.004858	0.090223	-1.80041
lnc-CIAPIN1-2:1	-1.50388	2.609558	-4.52436	0.004862	0.090228	-1.80127

lnc-LEPROTL1-13:4	1.488494	2.443748	4.523717	0.004865	0.090228	-1.80194
lnc-UBE2F-1:1	1.01482	6.786995	4.523012	0.004868	0.090228	-1.80269
NONHSAT148578.1	1.471388	8.779931	4.522954	0.004869	0.090228	-1.80275
lnc-IRF5-1:1	-1.56383	4.07131	-4.52192	0.004874	0.090266	-1.80385
MSTRG.35898.1	1.428924	1.802269	4.520748	0.004879	0.090322	-1.80509
NONHSAT150821.1	1.05495	3.009315	4.520079	0.004883	0.090324	-1.8058
NONHSAT184394.1	1.870403	2.802027	4.519031	0.004888	0.090324	-1.80691
NONHSAT167631.1	1.935348	3.313661	4.518612	0.00489	0.090324	-1.80736
NONHSAT186670.1	1.893628	2.883575	4.518278	0.004891	0.090324	-1.80771
ENST00000563103.1	1.129269	3.511832	4.518277	0.004891	0.090324	-1.80771
lnc-C10orf82-3:1	-1.21127	2.001014	-4.51816	0.004892	0.090324	-1.80784
NONHSAT195908.1	-1.73431	2.429203	-4.51753	0.004895	0.090337	-1.80851
MSTRG.54720.11	1.180531	2.313027	4.517461	0.004895	0.090337	-1.80858
lnc-ANKRD33-4:1	-1.07586	4.394337	-4.51283	0.004918	0.090431	-1.81349
lnc-REEP3-13:2	-1.09099	1.659042	-4.51245	0.00492	0.090431	-1.8139
NONHSAT220543.1	-1.49792	3.104624	-4.51145	0.004925	0.090431	-1.81495
NONHSAT154331.1	1.193037	4.292352	4.511349	0.004925	0.090431	-1.81506
NONHSAT218404.1	1.322507	1.741072	4.510939	0.004927	0.090431	-1.8155
ENST00000665877.1	1.216565	1.880246	4.510571	0.004929	0.090431	-1.81589
lnc-ACTR2-3:28	2.106732	2.375683	4.509942	0.004932	0.090442	-1.81656
NONHSAT164821.1	1.554481	3.454756	4.509534	0.004934	0.090442	-1.81699
ENST00000427524.1	-1.17689	2.226272	-4.50911	0.004936	0.090464	-1.81744
NONHSAT204222.1	1.278902	3.561349	4.50853	0.004939	0.090486	-1.81806
lnc-MYLIP-1:1	-1.39525	4.753602	-4.50839	0.00494	0.090486	-1.81821
NONHSAT208060.1	-1.21724	1.853894	-4.50826	0.004941	0.090486	-1.81834
lnc-RPGRIP1L-2:1	-1.52502	2.012464	-4.50777	0.004943	0.090486	-1.81887
NONHSAT215010.1	1.156512	2.311938	4.506484	0.004949	0.090488	-1.82023
NONHSAT167431.1	1.318711	1.950259	4.506229	0.004951	0.090495	-1.8205
lnc-SLC1A1-11:1	1.007337	3.083825	4.505042	0.004956	0.090555	-1.82176
MSTRG.14612.1	1.096198	5.065744	4.50337	0.004965	0.090555	-1.82354
lnc-HTR5A-5:2	1.225402	4.452639	4.503295	0.004965	0.090555	-1.82362
NONHSAT183125.1	1.275795	4.108934	4.502549	0.004969	0.090564	-1.82441
MSTRG.5689.1	-1.30024	2.876671	-4.50239	0.00497	0.090564	-1.82458
ENST00000575126.1	1.091303	7.497044	4.502097	0.004971	0.090574	-1.82489
NONHSAT196524.1	1.315406	3.864183	4.500931	0.004977	0.090604	-1.82613
lnc-VTI1A-7:1	-1.73751	2.012305	-4.50093	0.004977	0.090604	-1.82614
NONHSAT166190.1	1.557977	3.552641	4.500027	0.004981	0.090606	-1.82709
NONHSAT222224.1	1.257969	5.386198	4.498514	0.004989	0.090612	-1.8287
lnc-ATXN1-6:1	1.330001	5.703144	4.498437	0.004989	0.090612	-1.82879
NONHSAT211131.1	1.282461	6.626842	4.497296	0.004995	0.090629	-1.83
ENST00000665863.1	-1.30699	2.540658	-4.49659	0.004999	0.090629	-1.83075
lnc-ABR-6:4	-1.1944	3.002631	-4.4951	0.005006	0.090661	-1.83234
NONHSAT199304.1	1.450528	5.75581	4.493992	0.005012	0.090694	-1.83352
MSTRG.24872.1	-1.06391	2.817233	-4.49294	0.005017	0.09072	-1.83463
NONHSAT197953.1	1.545601	3.991541	4.49205	0.005021	0.090748	-1.83558
NONHSAT176880.1	-1.17586	2.81051	-4.49199	0.005022	0.090748	-1.83565
NONHSAT150379.1	-1.03905	6.171033	-4.49191	0.005022	0.090748	-1.83573
NONHSAT157000.1	1.793072	2.024587	4.491192	0.005026	0.090797	-1.8365

lnc-WDR11-2:4	1.891972	2.025047	4.490289	0.00503	0.09083	-1.83746
NONHSAT177925.1	1.004091	2.327066	4.489899	0.005032	0.090834	-1.83787
lnc-SEC22C-2:12	-1.48371	2.37236	-4.48946	0.005034	0.090858	-1.83835
lnc-TOM1L1-4:1	-1.42282	3.351283	-4.48876	0.005038	0.090905	-1.83909
T126419	2.456028	2.913646	4.487205	0.005046	0.090998	-1.84074
MSTRG.21497.5	1.162118	7.408038	4.485652	0.005054	0.091	-1.8424
lnc-CTXND2-7:2	-1.42105	3.376903	-4.48558	0.005054	0.091	-1.84248
NONHSAT190409.1	2.224753	3.305059	4.484947	0.005057	0.091041	-1.84315
NONHSAT161677.1	2.271487	3.425912	4.483501	0.005065	0.091092	-1.84469
NONHSAT154642.1	1.346397	2.650206	4.479357	0.005086	0.091244	-1.84911
lnc-TOP1-6:1	1.205532	7.275787	4.477563	0.005095	0.091294	-1.85103
NONHSAT153499.1	1.165544	6.711288	4.477536	0.005095	0.091294	-1.85105
MSTRG.7563.1	-1.81192	2.667554	-4.47665	0.005099	0.091294	-1.852
lnc-NUDT11-4:1	-1.14832	1.734908	-4.47649	0.0051	0.091294	-1.85217
ENST00000563063.1	1.652959	2.978526	4.476242	0.005102	0.091294	-1.85244
ENST00000667942.1	1.406493	2.140561	4.475079	0.005108	0.091294	-1.85368
NONHSAT203265.1	1.896085	4.077562	4.473164	0.005117	0.091294	-1.85572
MSTRG.45517.1	-2.13091	2.637416	-4.47308	0.005118	0.091294	-1.85582
NR_104178	-1.147	5.870671	-4.47293	0.005119	0.091294	-1.85597
lnc-NEFL-6:11	1.938294	3.04196	4.472848	0.005119	0.091294	-1.85606
NONHSAT220350.1	1.25283	3.826313	4.472641	0.00512	0.091294	-1.85628
NONHSAT211907.1	1.681483	1.948951	4.47215	0.005123	0.091294	-1.8568
NONHSAT194125.1	-1.01041	3.60555	-4.4721	0.005123	0.091294	-1.85686
lnc-TBP-10:1	-1.01132	3.103207	-4.47115	0.005128	0.091301	-1.85787
lnc-RNF20-7:1	1.176862	4.49757	4.471146	0.005128	0.091301	-1.85788
NONHSAT193879.1	2.0459	2.544982	4.470566	0.005131	0.091305	-1.8585
NONHSAT154429.1	1.106564	7.552694	4.470472	0.005131	0.091305	-1.8586
NONHSAT205738.1	1.583772	2.125304	4.469596	0.005136	0.091355	-1.85953
NONHSAT156376.1	1.332147	2.339779	4.468596	0.005141	0.09139	-1.8606
MSTRG.27276.2	1.535995	2.478306	4.467368	0.005147	0.091453	-1.86191
lnc-KMT2C-3:2	1.55722	2.749638	4.46652	0.005152	0.091457	-1.86282
NONHSAT216300.1	-1.0765	1.635155	-4.46646	0.005152	0.091457	-1.86289
NONHSAT196829.1	1.791586	2.965104	4.466107	0.005154	0.091474	-1.86326
lnc-BHLHE22-2:1	1.981475	3.459832	4.464929	0.00516	0.091476	-1.86452
lnc-SCN7A-3:1	1.545578	1.998301	4.464871	0.00516	0.091476	-1.86458
MSTRG.24026.1	-1.48688	3.588755	-4.46432	0.005163	0.091476	-1.86517
NONHSAT202315.1	1.241418	4.044791	4.46416	0.005164	0.091476	-1.86534
NONHSAT154701.1	1.066271	3.381152	4.463786	0.005166	0.091476	-1.86574
NONHSAT153713.1	-1.07248	5.237955	-4.46325	0.005169	0.091476	-1.86632
NONHSAT187860.1	1.395148	4.003085	4.463185	0.005169	0.091476	-1.86639
lnc-GCN1-1:2	-1.1515	8.60571	-4.46309	0.005169	0.091476	-1.86649
lnc-SCAMP1-6:1	1.050153	5.172727	4.462644	0.005172	0.091476	-1.86697
ENST00000550506.2	1.093964	1.596455	4.462207	0.005174	0.091476	-1.86743
NONHSAT219667.1	2.132744	2.618783	4.46177	0.005176	0.091487	-1.8679
NONHSAT176791.1	-1.21557	2.96171	-4.46109	0.00518	0.091501	-1.86862
MSTRG.55324.1	1.76894	2.56762	4.460245	0.005184	0.091547	-1.86953
lnc-ERC1-1:3	1.125484	5.482435	4.45845	0.005194	0.091601	-1.87145
NONHSAT161124.1	-1.31493	2.843963	-4.45827	0.005195	0.091601	-1.87165

NONHSAT170869.1	-1.03983	4.155887	-4.45798	0.005196	0.091601	-1.87196
T100137	-1.08157	2.471217	-4.45764	0.005198	0.091612	-1.87232
ENST00000427319.1	1.046077	4.778706	4.45693	0.005202	0.09165	-1.87308
NONHSAT171966.1	-1.37087	3.132593	-4.45542	0.00521	0.091689	-1.8747
NONHSAT214521.1	-1.06655	1.701021	-4.45302	0.005222	0.091754	-1.87727
ENST00000484222.1	1.750026	7.5632	4.45271	0.005224	0.091754	-1.8776
NONHSAT154545.1	1.231141	4.997447	4.45184	0.005228	0.091754	-1.87853
lnc-ABCA9-2:1	-1.39529	3.570347	-4.44994	0.005238	0.091761	-1.88057
MSTRG.18830.2	1.455146	1.981501	4.449845	0.005239	0.091761	-1.88067
NONHSAT179509.1	-1.39745	4.235073	-4.44923	0.005242	0.091761	-1.88133
lnc-LRFN5-4:1	-1.44833	3.801509	-4.44908	0.005243	0.091761	-1.88149
NONHSAT175830.1	1.063139	5.332653	4.449	0.005243	0.091761	-1.88157
NONHSAT171746.1	1.686745	7.400278	4.448513	0.005246	0.091761	-1.8821
NONHSAT222813.1	2.814904	3.425975	4.448502	0.005246	0.091761	-1.88211
NONHSAT162176.1	1.312358	2.65808	4.447977	0.005249	0.091764	-1.88267
lnc-SIRT4-5:1	1.489137	4.091664	4.447777	0.00525	0.091764	-1.88288
lnc-CCNJL-1:3	1.103386	4.289692	4.447447	0.005252	0.091764	-1.88324
lnc-TP53TG3F-39:1	1.049642	4.717432	4.447006	0.005254	0.091764	-1.88371
lnc-ABI1-11:1	2.463213	3.395167	4.446357	0.005257	0.091788	-1.88441
NONHSAT170272.1	1.868973	2.825604	4.4461	0.005259	0.091788	-1.88468
lnc-CLCN3-10:1	1.136331	2.835654	4.446062	0.005259	0.091788	-1.88472
NONHSAT154421.1	1.741342	2.327491	4.445573	0.005261	0.091807	-1.88525
lnc-SCAF8-4:1	-1.51838	3.681071	-4.44509	0.005264	0.091807	-1.88576
MSTRG.67610.1	1.459124	1.801598	4.444954	0.005265	0.091807	-1.88591
MSTRG.12454.2	2.414435	2.945658	4.44476	0.005266	0.091807	-1.88612
MSTRG.31978.1	-1.52421	2.349243	-4.44465	0.005266	0.091807	-1.88624
MSTRG.45201.1	1.682894	4.932386	4.443434	0.005273	0.091865	-1.88754
lnc-GJE1-4:1	-1.10713	4.147343	-4.442	0.00528	0.091874	-1.88908
NONHSAT181849.1	2.012962	2.510648	4.44181	0.005281	0.091874	-1.88928
T189967	1.092687	8.187009	4.441459	0.005283	0.091874	-1.88966
NONHSAT205770.1	-1.89225	2.178945	-4.44062	0.005288	0.091874	-1.89056
NONHSAT213086.1	1.579231	2.896486	4.439461	0.005294	0.091874	-1.8918
MSTRG.19158.1	1.049291	2.224439	4.439422	0.005294	0.091874	-1.89185
MSTRG.13498.2	-1.8341	3.251653	-4.43915	0.005296	0.091874	-1.89214
NONHSAT165033.1	1.044744	1.727417	4.438588	0.005299	0.091879	-1.89274
NONHSAT221901.1	-1.64188	4.035467	-4.43858	0.005299	0.091879	-1.89275
lnc-GNA12-2:1	-1.11145	4.12701	-4.43545	0.005316	0.092009	-1.89611
NONHSAT161312.1	-1.71248	2.678186	-4.43478	0.005319	0.092009	-1.89683
lnc-CAMSAP1-1:1	1.846041	4.610613	4.434766	0.005319	0.092009	-1.89684
lnc-TTLL2-1:1	1.665767	3.438956	4.434733	0.005319	0.092009	-1.89688
lnc-CORO6-3:1	-1.19732	5.014042	-4.43383	0.005324	0.092012	-1.89785
lnc-KCNJ15-3:2	-1.04767	5.484056	-4.43358	0.005326	0.092012	-1.89812
lnc-CXADR-7:1	1.801202	2.910092	4.433288	0.005327	0.092023	-1.89843
NONHSAT206367.1	1.442713	5.498073	4.432372	0.005332	0.092049	-1.89942
ENST00000583148.1	-1.39698	1.769806	-4.43135	0.005338	0.092097	-1.90052
NONHSAT191778.1	1.021511	5.911768	4.431276	0.005338	0.092097	-1.90059
ENST00000641784.1	2.106526	9.29331	4.43104	0.005339	0.092097	-1.90085
NONHSAT186349.1	1.956363	2.528836	4.428835	0.005351	0.092228	-1.90322

lnc-CCDC198-3:1	-1.04008	4.07542	-4.42782	0.005357	0.092228	-1.90431
NONHSAT210744.1	1.056647	4.933044	4.427233	0.00536	0.092228	-1.90494
NONHSAT154112.1	-1.20353	3.138743	-4.42714	0.00536	0.092228	-1.90504
lnc-ZNF322-6:1	-1.51941	2.545263	-4.42693	0.005361	0.092228	-1.90527
ENST00000496488.1	-1.5011	2.24038	-4.42593	0.005367	0.092228	-1.90634
NONHSAT207493.1	-1.49749	2.037825	-4.42583	0.005367	0.092228	-1.90645
NONHSAT187499.1	1.456696	2.107286	4.42558	0.005369	0.092228	-1.90672
lnc-MYBPC1-1:1	-1.3349	4.048116	-4.42551	0.005369	0.092228	-1.90679
NONHSAT187272.1	1.053555	1.593424	4.42248	0.005386	0.092399	-1.91005
NONHSAT165848.1	-1.4362	3.162328	-4.42234	0.005386	0.092399	-1.9102
NONHSAT202334.1	1.943218	2.341627	4.422251	0.005387	0.092399	-1.9103
lnc-KRTAP20-3-1:1	1.231458	1.716257	4.42205	0.005388	0.092399	-1.91052
LINC02105:7	1.466592	2.622051	4.421651	0.00539	0.092399	-1.91094
NONHSAT201887.1	1.444738	3.471983	4.421193	0.005393	0.092399	-1.91144
NONHSAT178740.1	-1.04588	6.020078	-4.42069	0.005395	0.092399	-1.91198
NONHSAT217991.1	1.658546	3.683848	4.420642	0.005396	0.092399	-1.91203
lnc-ULBP2-4:1	1.218705	3.956246	4.420084	0.005399	0.092399	-1.91263
NONHSAT207110.1	1.058281	1.582255	4.417394	0.005413	0.092399	-1.91553
lnc-SLC7A11-4:1	1.459971	1.841258	4.416871	0.005416	0.092399	-1.91609
NONHSAT187224.1	1.421015	1.819475	4.416071	0.005421	0.092399	-1.91695
lnc-DNAAF1-6:1	1.029585	1.624651	4.415545	0.005424	0.092399	-1.91752
lnc-ACHE-3:1	-1.13758	5.180552	-4.41509	0.005426	0.092399	-1.91801
NONHSAT192321.1	-1.27732	3.424424	-4.41492	0.005427	0.092399	-1.91819
NR_027003	1.508665	2.160054	4.414903	0.005427	0.092399	-1.91821
T213082	-1.20685	2.379063	-4.41475	0.005428	0.092399	-1.91838
MSTRG.67056.1	1.177045	4.390673	4.414197	0.005431	0.092399	-1.91897
NONHSAT196024.1	-2.04195	2.964204	-4.41392	0.005433	0.092399	-1.91927
MSTRG.12621.2	-1.82786	3.080377	-4.41309	0.005437	0.09245	-1.92017
lnc-SLC7A3-4:1	-1.17182	4.35594	-4.41214	0.005442	0.092477	-1.92119
ENST00000438619.1	1.94165	2.454966	4.411689	0.005445	0.092488	-1.92167
ENST00000658340.1	-1.36009	2.35696	-4.41128	0.005447	0.092503	-1.92212
lnc-MRFAP1L1-4:1	-1.14641	1.676302	-4.40979	0.005455	0.092541	-1.92372
MSTRG.58571.1	-1.18583	1.691276	-4.40834	0.005463	0.092599	-1.92528
NONHSAT207259.1	1.110709	3.668876	4.407657	0.005467	0.092648	-1.92602
MSTRG.17905.1	1.178862	1.604749	4.406787	0.005472	0.092699	-1.92696
NR_036575	-1.34822	3.182032	-4.40629	0.005475	0.092708	-1.9275
ENST00000652985.1	-1.00967	4.145112	-4.40518	0.005481	0.092719	-1.9287
MSTRG.24706.61	1.080576	7.779452	4.405062	0.005482	0.092719	-1.92882
lnc-TENM3-1:1	1.036828	4.335502	4.403394	0.005491	0.092719	-1.93062
lnc-CHD2-12:1	-1.6062	3.144324	-4.40306	0.005493	0.092719	-1.93098
NONHSAT185730.1	2.324635	2.796325	4.402777	0.005494	0.092719	-1.93128
ENST00000564696.1	1.196972	3.716513	4.402701	0.005495	0.092719	-1.93137
NONHSAT161321.1	-1.47297	1.830913	-4.40263	0.005495	0.092719	-1.93144
ENST00000662749.1	1.027678	4.11579	4.402269	0.005497	0.092719	-1.93183
lnc-TMEM18-20:1	-1.20363	3.63658	-4.40108	0.005504	0.092778	-1.93311
NONHSAT207865.1	1.82427	2.767157	4.401051	0.005504	0.092778	-1.93315
NONHSAT152939.1	-1.27789	2.275831	-4.40045	0.005507	0.092795	-1.93379
ENST00000668860.1	1.361171	2.013065	4.400311	0.005508	0.092795	-1.93395

NONHSAT219335.1	-2.09178	2.485814	-4.39933	0.005513	0.092837	-1.935
NONHSAT160171.1	-1.10512	2.098882	-4.39912	0.005515	0.092837	-1.93524
NR_024004	1.005443	8.733132	4.399047	0.005515	0.092837	-1.93531
NONHSAT201749.1	1.136258	1.681668	4.396836	0.005527	0.092968	-1.9377
lnc-ENC1-3:1	-2.08481	2.678273	-4.39611	0.005532	0.092982	-1.93848
NONHSAT218317.1	1.722512	2.772186	4.395296	0.005536	0.092999	-1.93936
T190918	1.286082	1.73043	4.394827	0.005539	0.093002	-1.93987
NONHSAT157297.1	-1.30786	3.025951	-4.39415	0.005543	0.093031	-1.9406
MSTRG.15403.1	1.117868	4.012986	4.394032	0.005543	0.093031	-1.94073
NONHSAT161538.1	1.172068	2.998158	4.393	0.005549	0.093081	-1.94184
NONHSAT204076.1	1.430374	2.398985	4.391565	0.005557	0.093119	-1.94339
NONHSAT188001.1	1.265853	4.279885	4.391298	0.005559	0.093119	-1.94368
lnc-FANCL-2:2	1.588722	1.818815	4.390178	0.005565	0.093179	-1.94489
lnc-PHLDA3-2:1	1.281573	3.968469	4.389989	0.005566	0.093179	-1.9451
lnc-APPBP2-6:1	-1.34223	3.325443	-4.38896	0.005572	0.093179	-1.94621
lnc-DDX3X-7:1	-1.17221	3.286983	-4.38826	0.005576	0.093179	-1.94696
lnc-DNAJC24-5:1	-1.34241	3.018471	-4.38769	0.005579	0.093179	-1.94758
NONHSAT210312.1	1.677017	2.814494	4.387562	0.00558	0.093179	-1.94772
NONHSAT205558.1	-1.39346	2.951359	-4.38706	0.005583	0.093179	-1.94827
MSTRG.34381.11	1.748372	3.571801	4.386998	0.005583	0.093179	-1.94833
LINC02357:8	1.625347	2.262533	4.386769	0.005584	0.093179	-1.94858
ENST00000454723.2	-1.91914	2.716092	-4.38675	0.005584	0.093179	-1.9486
NONHSAT201297.1	1.227597	5.827887	4.386597	0.005585	0.093179	-1.94877
lnc-CDYL-14:1	1.114193	6.195834	4.38659	0.005585	0.093179	-1.94877
ENST00000562814.1	1.393235	2.428635	4.385823	0.00559	0.093209	-1.9496
ENST00000504650.2	1.148049	3.880427	4.385458	0.005592	0.093209	-1.95
LINC00114:9	1.184534	1.706028	4.385001	0.005594	0.093209	-1.95049
lnc-RAB11FIP4-8:1	1.087485	3.824228	4.384761	0.005596	0.093209	-1.95075
NONHSAT151203.1	1.093889	1.586259	4.384638	0.005596	0.093209	-1.95089
NONHSAT166131.1	1.280803	3.32616	4.383471	0.005603	0.093209	-1.95215
ENST00000667238.1	1.249823	7.147965	4.382979	0.005606	0.093209	-1.95268
lnc-WRNIP1-28:1	-1.15826	1.690233	-4.38254	0.005608	0.093209	-1.95315
ENST00000663358.1	1.841202	2.414893	4.380679	0.005619	0.09332	-1.95517
MSTRG.21244.1	-1.06512	4.607028	-4.37987	0.005624	0.093355	-1.95605
lnc-SLC39A11-6:1	1.25343	2.282932	4.379702	0.005625	0.093356	-1.95623
lnc-UTY-15:1	1.135769	5.269083	4.377206	0.005639	0.09347	-1.95893
MSTRG.19443.2	1.838491	2.003536	4.375895	0.005646	0.093549	-1.96035
ENST00000567718.1	1.523235	2.198587	4.375355	0.00565	0.09356	-1.96094
NONHSAT173855.1	1.731132	2.338268	4.373908	0.005658	0.093599	-1.96251
MSTRG.62501.1	1.484183	3.50972	4.37291	0.005664	0.093634	-1.96359
lnc-HEXA-1:1	-1.65492	2.766326	-4.37231	0.005667	0.093638	-1.96423
NONHSAT169353.1	1.257247	2.456377	4.370586	0.005677	0.093701	-1.96611
ENST00000443018.2	1.657669	2.217119	4.370137	0.00568	0.093701	-1.9666
NONHSAT155095.1	-1.13947	2.141446	-4.36963	0.005683	0.093701	-1.96714
MSTRG.64377.1	1.023423	4.638305	4.369214	0.005685	0.093709	-1.9676
NONHSAT181450.1	-1.26167	2.336153	-4.36888	0.005687	0.093712	-1.96796
MSTRG.22908.3	-1.02374	3.501768	-4.3682	0.005691	0.093731	-1.9687
lnc-DAZ1-166:2	-1.82312	2.661556	-4.36653	0.005701	0.093821	-1.97051

ENST00000504365.1	1.50576	2.133955	4.365562	0.005706	0.093861	-1.97156
ENST00000663591.1	1.089602	3.64256	4.364692	0.005711	0.093886	-1.9725
lnc-ZC3HAV1-2:1	1.645812	2.147655	4.364658	0.005712	0.093886	-1.97254
NONHSAT164372.1	-1.39937	2.149578	-4.36449	0.005712	0.093887	-1.97272
T341075	-1.41083	3.148023	-4.36422	0.005714	0.093897	-1.97301
NONHSAT170717.1	-1.36999	4.69176	-4.36375	0.005717	0.093912	-1.97353
lnc-CAB39L-8:4	1.451662	2.401701	4.362394	0.005725	0.09398	-1.975
MSTRG.18542.1	-1.41203	2.279345	-4.36114	0.005732	0.094024	-1.97636
lnc-PEX16-4:1	-1.43784	2.411667	-4.36072	0.005734	0.094044	-1.97681
lnc-SLC6A3-1:1	1.378086	4.299295	4.360612	0.005735	0.094044	-1.97693
ENST00000560375.5	1.168936	1.60415	4.359904	0.005739	0.094066	-1.9777
NR_125401	1.391671	4.608786	4.359198	0.005743	0.094104	-1.97847
NONHSAT162721.1	1.238294	2.115169	4.357002	0.005756	0.094254	-1.98085
MSTRG.47596.1	-1.03847	3.342528	-4.3563	0.00576	0.094266	-1.98161
ENST00000670607.1	1.058283	4.455727	4.356189	0.005761	0.094266	-1.98174
NR_015445	-1.17116	5.172299	-4.35518	0.005767	0.094266	-1.98283
MSTRG.49157.1	-1.42112	3.388773	-4.35517	0.005767	0.094266	-1.98285
NONHSAT213696.1	-1.04518	3.927613	-4.35513	0.005767	0.094266	-1.98288
NONHSAT179204.1	1.131139	4.68231	4.354376	0.005772	0.094292	-1.98371
T016619	-1.3062	3.049279	-4.35373	0.005776	0.094317	-1.98441
MSTRG.23228.1	1.369188	2.305662	4.353615	0.005776	0.094317	-1.98453
LINC02535:17	-1.12575	4.389475	-4.35349	0.005777	0.094317	-1.98467
NONHSAT158543.1	-1.28739	3.836601	-4.35248	0.005783	0.094335	-1.98577
lnc-PPM1M-2:5	-1.64477	2.330971	-4.35235	0.005784	0.094335	-1.98591
NONHSAT191418.1	1.439696	4.989363	4.351438	0.005789	0.094377	-1.9869
ENST00000504357.1	-1.27445	2.363905	-4.35031	0.005796	0.094386	-1.98813
lnc-PTGES-2:1	1.323609	4.347956	4.350251	0.005796	0.094386	-1.98819
NONHSAT218194.1	-1.07067	3.154669	-4.35013	0.005797	0.094386	-1.98832
MSTRG.12812.1	1.116809	3.661474	4.350081	0.005797	0.094386	-1.98838
MSTRG.11047.2	1.110424	3.666764	4.349723	0.005799	0.09439	-1.98877
lnc-TMEM243-2:1	-1.08026	3.156655	-4.34865	0.005806	0.094471	-1.98993
ENST00000329015.2	-1.04178	2.887465	-4.3481	0.005809	0.094471	-1.99054
ENST00000661716.1	1.247762	4.532111	4.347552	0.005812	0.094478	-1.99113
MSTRG.41651.1	1.236091	2.278028	4.345828	0.005822	0.094567	-1.99301
lnc-TRIM32-6:1	1.292813	3.844508	4.345215	0.005826	0.094567	-1.99367
NONHSAT175983.1	-1.2839	5.834248	-4.34488	0.005828	0.094584	-1.99404
NONHSAT198952.1	-1.64326	2.505486	-4.3431	0.005839	0.094695	-1.99597
MSTRG.34193.1	1.477262	2.140131	4.34283	0.00584	0.0947	-1.99627
NONHSAT154003.1	1.313486	3.266654	4.340972	0.005851	0.094824	-1.99829
ENST00000420828.1	-1.3866	1.976692	-4.34078	0.005853	0.094824	-1.9985
lnc-ZCCHC17-8:1	-1.76688	2.631214	-4.33991	0.005858	0.094831	-1.99945
ENST00000479822.2	1.711996	3.583284	4.339108	0.005863	0.094832	-2.00032
lnc-SLX4IP-5:2	1.027346	1.635894	4.337494	0.005872	0.09495	-2.00208
ENST00000428485.1	-1.15596	2.060351	-4.33724	0.005874	0.094959	-2.00236
NONHSAT209770.1	2.114313	2.484138	4.336222	0.00588	0.094994	-2.00347
NONHSAT162467.1	1.042691	4.749603	4.335701	0.005883	0.094996	-2.00404
NONHSAT215171.1	1.975471	3.005678	4.335518	0.005884	0.094996	-2.00423
lnc-HNRNPA2B1-12:1	1.047155	5.636306	4.334959	0.005888	0.095004	-2.00484

MSTRG.29893.2	-1.44556	2.964092	-4.33412	0.005893	0.09502	-2.00576
NONHSAT224126.1	-1.27533	3.166924	-4.33335	0.005897	0.095027	-2.00659
lnc-COX10-7:1	-1.18376	1.999072	-4.33327	0.005898	0.095027	-2.00668
lnc-ACTR10-6:1	-1.47979	3.402814	-4.33201	0.005905	0.095078	-2.00806
NR_024604	-1.10045	4.719334	-4.329	0.005924	0.095188	-2.01134
NONHSAT164533.1	-1.79207	2.05604	-4.32824	0.005928	0.095188	-2.01217
lnc-KCNS2-4:3	-1.15708	1.789738	-4.32804	0.005929	0.095188	-2.0124
lnc-TTLL7-11:1	1.266084	3.503922	4.325594	0.005944	0.095275	-2.01506
lnc-SPINT2-8:1	-1.34215	4.453115	-4.32421	0.005953	0.095365	-2.01657
NONHSAT152290.1	-1.37505	1.87647	-4.32286	0.005961	0.095406	-2.01805
ENST00000655634.1	1.010915	1.814345	4.322678	0.005962	0.095409	-2.01825
NONHSAT197747.1	1.837695	3.96546	4.322448	0.005964	0.095413	-2.0185
NONHSAT186107.1	-1.40696	2.673681	-4.32171	0.005968	0.095413	-2.0193
NONHSAT193956.1	1.134186	1.949168	4.321267	0.005971	0.095427	-2.01979
lnc-KCNV2-4:1	-1.31619	3.012708	-4.32058	0.005975	0.095463	-2.02054
MSTRG.49845.3	1.356788	3.625157	4.319761	0.00598	0.095503	-2.02143
NONHSAT216546.1	-1.09139	1.716871	-4.3162	0.006002	0.09572	-2.02532
NONHSAT178542.1	1.42675	1.995792	4.315975	0.006003	0.09572	-2.02557
NONHSAT164680.1	1.316797	2.701031	4.315952	0.006003	0.09572	-2.02559
NONHSAT204898.1	-1.80646	3.603002	-4.31564	0.006005	0.09572	-2.02593
MSTRG.44205.1	-1.07868	3.780819	-4.31546	0.006006	0.09572	-2.02613
lnc-DHTKD1-2:1	1.031721	3.4889	4.314959	0.00601	0.09572	-2.02668
lnc-KCNG3-2:1	-1.6015	3.176078	-4.31476	0.006011	0.09572	-2.02689
lnc-HIST1H4D-2:1	-1.80395	3.521481	-4.3142	0.006014	0.095735	-2.02751
ENST00000572067.1	-1.2136	2.906953	-4.31399	0.006016	0.095735	-2.02774
lnc-GPR135-2:1	-1.19149	2.691243	-4.31386	0.006016	0.095735	-2.02788
NONHSAT183373.1	1.274619	4.785295	4.313127	0.006021	0.095735	-2.02868
lnc-NAA38-2:2	-1.49492	3.041964	-4.31239	0.006025	0.095742	-2.02949
NONHSAT180303.1	1.644538	2.202745	4.31174	0.00603	0.095742	-2.0302
MSTRG.36212.1	1.14347	4.360425	4.311491	0.006031	0.095743	-2.03047
ENST00000670726.1	-1.30317	1.735918	-4.31142	0.006031	0.095743	-2.03055
NONHSAT161153.1	-1.39943	1.888654	-4.31083	0.006035	0.095768	-2.03119
NONHSAT153090.1	-1.08149	3.319539	-4.31063	0.006036	0.095768	-2.03141
NONHSAT221701.1	1.058251	5.923513	4.310375	0.006038	0.095768	-2.03169
lnc-POU6F2-1:1	2.23585	3.410288	4.310141	0.006039	0.095768	-2.03195
NONHSAT148636.1	1.188184	2.961446	4.310101	0.00604	0.095768	-2.03199
ENST00000611525.1	1.57255	2.398345	4.309383	0.006044	0.095779	-2.03278
NONHSAT157970.1	1.181453	3.902246	4.309333	0.006044	0.095779	-2.03283
lnc-CPQ-4:1	-1.80371	2.456629	-4.30915	0.006046	0.095779	-2.03303
NONHSAT164714.1	1.417631	2.931761	4.309067	0.006046	0.095779	-2.03313
NONHSAT210251.1	1.880187	3.213144	4.309065	0.006046	0.095779	-2.03313
ENST00000658843.1	1.581188	3.044421	4.307739	0.006054	0.09585	-2.03458
LINC01384:1	-2.05456	3.820052	-4.30623	0.006064	0.095938	-2.03623
lnc-ZNF491-1:1	-1.07949	4.284413	-4.30506	0.006071	0.096006	-2.03751
NONHSAT177475.1	1.233222	4.756517	4.303853	0.006079	0.09606	-2.03883
MSTRG.60097.1	2.241704	3.76031	4.303609	0.00608	0.09606	-2.0391
T245853	-1.21124	3.729695	-4.30309	0.006083	0.096082	-2.03967
NONHSAT187841.1	1.693758	4.12885	4.30272	0.006086	0.096084	-2.04007

ENST00000528696.3	1.212826	3.696406	4.302171	0.006089	0.096089	-2.04068
lnc-UCP3-6:1	-1.16429	2.539489	-4.30182	0.006091	0.096089	-2.04106
NONHSAT186397.1	1.113239	1.901484	4.30048	0.0061	0.096099	-2.04253
lnc-API5-9:1	-1.29362	3.187334	-4.30004	0.006102	0.096099	-2.04301
ENST00000562989.1	-1.18205	4.879718	-4.29988	0.006103	0.096099	-2.04318
ENST00000657264.1	2.501379	3.719683	4.298658	0.006111	0.096133	-2.04453
NONHSAT187963.1	-1.25736	4.388246	-4.29834	0.006113	0.096133	-2.04487
MSTRG.42851.1	-1.14836	2.406721	-4.29814	0.006114	0.096133	-2.0451
MSTRG.21093.1	1.067741	2.521285	4.298085	0.006115	0.096133	-2.04515
NONHSAT153306.1	-1.08774	2.018774	-4.29808	0.006115	0.096133	-2.04516
ENST00000581636.1	-1.14634	4.935919	-4.29729	0.00612	0.096133	-2.04603
NONHSAT201818.1	1.177168	5.588157	4.29698	0.006122	0.096133	-2.04636
lnc-TRAM1L1-4:1	1.578947	3.011588	4.296978	0.006122	0.096133	-2.04637
NONHSAT214019.1	-1.74103	2.756673	-4.2959	0.006129	0.096209	-2.04755
ENST00000608131.1	-1.15646	3.541648	-4.29427	0.006139	0.096274	-2.04934
NONHSAT171724.1	2.055843	2.395056	4.292932	0.006147	0.096274	-2.0508
lnc-CPEB4-5:2	1.375749	2.006574	4.292682	0.006149	0.096274	-2.05108
ENST00000457647.2	1.01306	5.095513	4.292678	0.006149	0.096274	-2.05108
NONHSAT163966.1	1.625183	5.50738	4.29131	0.006158	0.096386	-2.05258
ENST00000608748.1	-1.10464	5.807349	-4.29007	0.006166	0.096422	-2.05395
NONHSAT168332.1	-1.30533	2.09256	-4.28957	0.006169	0.096422	-2.05449
MSTRG.65628.1	-1.46816	2.293401	-4.28941	0.00617	0.096422	-2.05467
LINC01341:4	1.340095	4.630138	4.289288	0.00617	0.096422	-2.0548
NONHSAT164410.1	-1.07977	1.656004	-4.28853	0.006175	0.09645	-2.05563
lnc-C5orf47-2:1	1.138744	4.314705	4.2885	0.006175	0.09645	-2.05567
T017889	1.274316	1.786199	4.287748	0.00618	0.09651	-2.05649
MSTRG.6310.1	2.302418	3.377347	4.28594	0.006192	0.09663	-2.05848
NONHSAT220918.1	1.742711	2.727097	4.284258	0.006203	0.096708	-2.06033
NONHSAT149352.1	2.084418	2.680586	4.282653	0.006213	0.096794	-2.06209
NONHSAT196291.1	-1.17078	5.302318	-4.2825	0.006214	0.096794	-2.06225
MSTRG.34330.1	1.692132	4.275478	4.280937	0.006224	0.096899	-2.06397
NONHSAT198479.1	-1.31062	4.401543	-4.28055	0.006226	0.096899	-2.0644
NONHSAT194176.1	1.686752	3.884659	4.28026	0.006228	0.096913	-2.06472
ENST00000436429.1	-1.3035	3.248176	-4.27863	0.006239	0.097017	-2.06651
NONHSAT187763.1	-1.10534	1.789776	-4.27827	0.006241	0.097021	-2.0669
NONHSAT222125.1	1.527093	2.06309	4.277731	0.006245	0.097036	-2.0675
lnc-RD3-6:1	1.186122	2.720004	4.277542	0.006246	0.097036	-2.06771
NONHSAT172383.1	-1.04947	3.665917	-4.27722	0.006248	0.097052	-2.06806
RNASEH1-AS1:26	1.342604	2.501246	4.275831	0.006257	0.097128	-2.06959
lnc-HMGB2-8:1	-1.35738	2.594873	-4.27572	0.006258	0.097128	-2.06971
NONHSAT198283.1	2.238436	2.749366	4.274869	0.006263	0.097159	-2.07064
ENST00000379963.1	1.638416	4.337548	4.272794	0.006276	0.097302	-2.07293
NONHSAT189814.1	1.52129	1.859208	4.272652	0.006277	0.097302	-2.07308
lnc-TRPV6-3:1	1.395715	5.917964	4.272449	0.006279	0.097308	-2.07331
NONHSAT165135.1	1.372726	2.436107	4.270206	0.006293	0.097384	-2.07578
ENST00000645861.1	1.169902	4.80016	4.26806	0.006307	0.097387	-2.07814
NONHSAT210654.1	1.03163	1.635644	4.26677	0.006316	0.097454	-2.07956
ENST00000532849.1	1.305716	5.722323	4.266251	0.006319	0.097469	-2.08013

NONHSAT201718.1	1.210649	6.677796	4.265847	0.006322	0.09748	-2.08058
lnc-ABCE1-1:1	-1.09744	5.674425	-4.26566	0.006323	0.097484	-2.08078
MSTRG.53117.1	1.409467	3.246864	4.2652	0.006326	0.097512	-2.08129
ENST00000667961.1	-1.37641	3.38164	-4.26491	0.006328	0.097515	-2.08161
lnc-SP4-4:1	1.476554	2.681538	4.264341	0.006332	0.097533	-2.08223
ENST00000515670.1	-1.19607	4.409316	-4.26429	0.006332	0.097533	-2.08229
NONHSAT162506.1	1.38368	3.929667	4.263195	0.006339	0.097544	-2.0835
NONHSAT154057.1	1.634905	2.191198	4.263064	0.00634	0.097544	-2.08364
lnc-RC3H1-1:9	1.00212	3.794323	4.26289	0.006341	0.097544	-2.08383
NONHSAT158400.1	2.09205	2.583789	4.261915	0.006347	0.097579	-2.08491
NONHSAT170032.1	1.530404	2.600278	4.261664	0.006349	0.097585	-2.08518
MSTRG.25374.4	-1.21826	2.718553	-4.26156	0.00635	0.097585	-2.0853
MSTRG.59236.32	-1.04277	1.638582	-4.26112	0.006353	0.097615	-2.08579
NONHSAT153933.1	-1.59946	1.944347	-4.25929	0.006365	0.097648	-2.0878
NONHSAT153829.1	2.480291	2.990663	4.256934	0.00638	0.097762	-2.0904
lnc-PKLR-6:1	-1.35915	3.785013	-4.25675	0.006381	0.097762	-2.0906
NONHSAT205725.1	1.31323	6.575941	4.256552	0.006383	0.097762	-2.09082
NONHSAT182571.1	1.237488	4.28271	4.255993	0.006386	0.097762	-2.09144
ENST00000635064.1	1.197291	5.622103	4.254574	0.006396	0.097835	-2.093
NONHSAT167737.1	1.693609	2.299129	4.254493	0.006396	0.097835	-2.09309
NR_110112	-1.39941	2.294252	-4.25407	0.006399	0.097835	-2.09356
NONHSAT210377.1	1.386603	2.866236	4.253952	0.0064	0.097835	-2.09369
MSTRG.41826.1	-1.11068	3.139584	-4.25222	0.006412	0.09792	-2.0956
NONHSAT221803.1	1.853446	2.105972	4.251947	0.006413	0.09792	-2.0959
T207405	1.841144	7.11991	4.251489	0.006416	0.097951	-2.09641
lnc-CYP24A1-2:1	1.172985	3.137862	4.250968	0.00642	0.097989	-2.09698
lnc-TOR1AIP2-2:5	1.244527	3.367425	4.250164	0.006425	0.098041	-2.09787
T304770	1.255767	3.777041	4.249339	0.006431	0.09808	-2.09878
NONHSAT224320.1	1.78967	3.8729	4.248003	0.00644	0.098149	-2.10025
NR_110699	2.048155	2.935757	4.247915	0.00644	0.098149	-2.10035
lnc-MUC20-4:3	1.577915	3.71643	4.247648	0.006442	0.098149	-2.10065
lnc-EML4-2:1	1.141851	4.816695	4.247465	0.006443	0.098153	-2.10085
lnc-DZANK1-3:3	-1.69041	3.955347	-4.24679	0.006448	0.098157	-2.10159
NR_004401	-1.18529	1.883866	-4.24669	0.006448	0.098157	-2.1017
NONHSAT166593.1	-1.63574	2.480052	-4.24626	0.006451	0.098186	-2.10218
NONHSAT185478.1	-1.68006	2.552498	-4.24603	0.006453	0.098195	-2.10244
T245856	-1.40982	2.958502	-4.24547	0.006457	0.098208	-2.10305
NONHSAT199376.1	1.629007	2.859839	4.244456	0.006463	0.098292	-2.10417
lnc-ZFP42-4:2	1.310485	4.888124	4.243914	0.006467	0.098296	-2.10477
MSTRG.10985.3	1.724175	2.355252	4.243825	0.006468	0.098296	-2.10487
NONHSAT194161.1	1.012708	3.294119	4.243531	0.00647	0.098296	-2.10519
NONHSAT206432.1	-1.80266	2.40013	-4.24277	0.006475	0.098296	-2.10603
lnc-METAP1-3:9	-1.32128	2.312701	-4.24197	0.00648	0.098296	-2.10692
ENST00000503091.1	-1.54926	2.091654	-4.24061	0.006489	0.098296	-2.10842
ENST00000642235.1	1.044325	1.794628	4.240599	0.006489	0.098296	-2.10844
ENST00000665007.1	-1.45083	3.087687	-4.2404	0.006491	0.098296	-2.10865
ENST00000649237.1	1.357841	10.18434	4.239277	0.006498	0.098371	-2.1099
lnc-BTNL2-2:1	-1.34456	1.768099	-4.23885	0.006501	0.098371	-2.11037

MSTRG.63841.1	-1.79958	3.873303	-4.23781	0.006508	0.098428	-2.11152
ENST00000647804.1	1.919726	2.650525	4.236367	0.006518	0.09847	-2.11312
lnc-TAPT1-4:1	-1.01201	6.882692	-4.23626	0.006519	0.09847	-2.11323
NONHSAT202447.1	1.240642	3.127506	4.236152	0.006519	0.09847	-2.11335
NONHSAT151721.1	1.207118	4.022955	4.235289	0.006525	0.098527	-2.11431
lnc-TFF3-4:1	1.236368	5.53348	4.234399	0.006531	0.09854	-2.11529
NONHSAT157864.1	1.794643	3.187743	4.232327	0.006545	0.098596	-2.11758
MSTRG.1842.8	-1.11182	3.048817	-4.23227	0.006546	0.098596	-2.11765
lnc-RNF227-2:1	-1.19035	4.115453	-4.22937	0.006565	0.098776	-2.12086
NONHSAT181882.1	1.648307	1.981564	4.227544	0.006578	0.098905	-2.12288
lnc-IQCF3-1:4	1.629742	2.412033	4.225651	0.006591	0.09893	-2.12498
MSTRG.12936.12	1.415933	1.792012	4.225576	0.006591	0.09893	-2.12506
lnc-AGBL4-3:1	1.589513	2.329051	4.225289	0.006593	0.09893	-2.12538
lnc-NAIF1-1:4	-1.22348	1.897425	-4.22522	0.006594	0.09893	-2.12545
lnc-ABT1-6:1	1.123935	3.498863	4.225087	0.006595	0.09893	-2.1256
NONHSAT209150.1	1.705399	2.390146	4.225004	0.006595	0.09893	-2.12569
ENST00000586894.1	-1.3277	3.057326	-4.22402	0.006602	0.098996	-2.12678
NONHSAT197646.1	1.874562	3.002015	4.223939	0.006603	0.098996	-2.12687
NONHSAT150433.1	1.107343	4.15328	4.223196	0.006608	0.099014	-2.1277
lnc-COA1-3:1	-1.63079	2.09918	-4.2228	0.00661	0.099014	-2.12814
ENST00000626448.1	-1.57973	2.795345	-4.22262	0.006612	0.099014	-2.12833
NONHSAT214330.1	1.201433	4.115065	4.222623	0.006612	0.099014	-2.12833
lnc-ARL6IP6-3:1	1.158689	1.704881	4.222401	0.006613	0.099022	-2.12858
lnc-SMCO2-4:1	-1.00989	4.537369	-4.22202	0.006616	0.099027	-2.129
lnc-IRF7-1:1	1.273227	3.550048	4.221174	0.006622	0.099027	-2.12994
lnc-CD79B-2:1	-1.28882	4.172942	-4.22107	0.006622	0.099027	-2.13005
NONHSAT175262.1	1.664572	2.004917	4.220606	0.006626	0.099042	-2.13057
T126462	2.15158	2.528526	4.220355	0.006627	0.099042	-2.13085
MSTRG.6455.1	1.949484	3.041225	4.218554	0.00664	0.099164	-2.13285
T006816	1.689003	1.946341	4.218193	0.006642	0.099177	-2.13325
lnc-NUBPL-1:5	1.932833	2.490991	4.217258	0.006649	0.099244	-2.13428
ENST00000567966.1	1.774328	1.980317	4.215041	0.006664	0.099361	-2.13674
NR_104616	1.013907	3.850304	4.214563	0.006667	0.099361	-2.13727
NONHSAT165026.1	1.401916	2.014641	4.214206	0.00667	0.099374	-2.13767
MSTRG.14798.1	1.143738	2.210505	4.213972	0.006671	0.099378	-2.13793
NONHSAT216351.1	-1.47255	3.548411	-4.21377	0.006673	0.099384	-2.13815
ENST00000654007.1	1.692117	5.202183	4.211754	0.006687	0.09952	-2.14039
MSTRG.72423.1	-1.37664	3.967114	-4.21117	0.006691	0.099566	-2.14103
NONHSAT154406.1	1.762919	2.870005	4.209344	0.006704	0.099661	-2.14307
MSTRG.32700.1	1.153126	5.035617	4.206786	0.006722	0.099794	-2.14591
MSTRG.45516.5	1.598368	2.475284	4.204464	0.006738	0.099883	-2.14849
lnc-GLT1D1-4:2	1.139691	3.513408	4.204159	0.00674	0.099883	-2.14883
ENST00000527321.1	1.363221	7.528103	4.202915	0.006749	0.099926	-2.15021
lnc-VCAN-4:1	-1.11472	4.73551	-4.20261	0.006751	0.099942	-2.15055
MSTRG.65635.3	-1.27771	3.815506	-4.20181	0.006757	0.099961	-2.15144
NR_109894	1.185784	3.731699	4.201531	0.006759	0.099961	-2.15175
lnc-GRSF1-1:1	-1.10713	3.399566	-4.20138	0.00676	0.099961	-2.15191
MSTRG.71413.1	-1.58437	3.073818	-4.20112	0.006762	0.099961	-2.15221

NONHSAT215465.1	-1.07021	3.523042	-4.20091	0.006763	0.099961	-2.15244
lnc-ADAM22-7:1	1.417909	4.506566	4.200552	0.006766	0.099961	-2.15284
NONHSAT170849.1	-1.34324	4.715802	-4.20055	0.006766	0.099961	-2.15284
lnc-PLA2G4A-7:5	1.246481	4.138346	4.200473	0.006766	0.099961	-2.15292
MSTRG.32945.20	1.326712	1.704957	4.199517	0.006773	0.099995	-2.15399
MSTRG.50863.1	2.068593	5.04873	4.198594	0.006779	0.100025	-2.15501
lnc-PLCL2-11:1	1.808995	2.382069	4.198233	0.006782	0.100048	-2.15542
lnc-NUDT15-4:1	1.503219	4.510082	4.197754	0.006785	0.100051	-2.15595
NONHSAT154631.1	1.194226	5.894698	4.196481	0.006794	0.100114	-2.15737
lnc-CRX-5:2	1.142864	4.300648	4.196402	0.006795	0.100114	-2.15745
MSTRG.67292.1	-1.63662	2.081064	-4.19615	0.006797	0.10012	-2.15774
T190328	1.035463	5.176546	4.19568	0.0068	0.100154	-2.15826
ENST00000591038.1	1.687846	3.146673	4.195127	0.006804	0.10018	-2.15887
NONHSAT219302.1	1.494123	3.108328	4.194836	0.006806	0.10018	-2.1592
MSTRG.65885.2	1.930105	3.074519	4.194554	0.006808	0.10018	-2.15951
NONHSAT148328.1	1.66884	1.935619	4.194382	0.006809	0.10018	-2.1597
lnc-SLC10A5-1:2	-1.07346	4.780397	-4.19409	0.006811	0.10018	-2.16003
lnc-CCDC74A-7:3	-1.06145	3.876998	-4.19403	0.006812	0.10018	-2.16009
lnc-C12orf75-5:1	-1.03914	10.0937	-4.19298	0.006819	0.100215	-2.16126
NONHSAT190483.1	-1.07163	2.023085	-4.19283	0.00682	0.100215	-2.16143
lnc-GUCD1-1:1	-1.10819	5.720091	-4.19217	0.006825	0.100259	-2.16216
lnc-RRM1-6:1	-1.38871	4.135037	-4.192	0.006826	0.100261	-2.16235
lnc-WASHC4-3:2	1.67656	2.280272	4.190428	0.006837	0.100328	-2.1641
ENST00000420020.5	-1.38278	2.387701	-4.19018	0.006839	0.100328	-2.16438
lnc-ZNF107-6:1	-1.00367	2.944112	-4.19011	0.00684	0.100328	-2.16446
lnc-GTF2B-7:1	-1.94009	2.949412	-4.18942	0.006845	0.100328	-2.16522
NONHSAT190073.1	-1.86014	2.905471	-4.18806	0.006854	0.100347	-2.16674
MSTRG.15295.1	1.261449	2.124962	4.186706	0.006864	0.100347	-2.16825
MSTRG.21468.3	1.196472	2.584165	4.186068	0.006869	0.100347	-2.16896
NONHSAT168514.1	1.371858	2.151215	4.186008	0.006869	0.100347	-2.16903
NONHSAT184635.1	-1.18796	2.086208	-4.18599	0.006869	0.100347	-2.16905
NONHSAT205263.1	1.045726	3.36084	4.18572	0.006871	0.100347	-2.16935
NONHSAT197652.1	1.368533	2.100321	4.185498	0.006873	0.100347	-2.1696
NONHSAT170181.1	-1.38455	2.363483	-4.18472	0.006878	0.100399	-2.17046
ENST00000419362.1	1.334567	1.719016	4.183582	0.006887	0.100446	-2.17173
NR_110877	1.630404	2.134151	4.183244	0.006889	0.100467	-2.17211
lnc-MAP10-1:1	2.110359	2.971515	4.181415	0.006902	0.100572	-2.17415
NONHSAT214547.1	1.50281	3.378078	4.180836	0.006906	0.100575	-2.17479
lnc-ZFHX4-5:1	-2.07263	3.364782	-4.18064	0.006908	0.100582	-2.17502
lnc-CDY1B-13:1	-1.1505	4.717581	-4.17947	0.006916	0.100647	-2.17632
MSTRG.67720.1	-1.13935	4.167229	-4.17833	0.006925	0.100694	-2.17759
lnc-PSG2-5:2	1.257933	4.928158	4.177499	0.006931	0.10072	-2.17852
NONHSAT201637.1	-1.11457	1.795648	-4.17657	0.006937	0.10072	-2.17955
NONHSAT221731.1	1.245748	1.720965	4.176272	0.00694	0.100723	-2.17988
NONHSAT201154.1	1.94903	4.044475	4.173215	0.006962	0.100944	-2.1833
lnc-PTGS2-3:1	-1.29279	3.066595	-4.17189	0.006971	0.100967	-2.18477
lnc-TNFAIP3-5:5	1.174699	1.997115	4.170433	0.006982	0.10105	-2.1864
NONHSAT161440.1	1.896814	3.022158	4.169824	0.006987	0.101071	-2.18708

lnc-TRAF5-6:12	-1.35359	2.277517	-4.16939	0.00699	0.101102	-2.18757
ENST00000515871.1	-1.47919	4.931503	-4.16765	0.007003	0.1012	-2.18952
NONHSAT150546.1	-2.34145	2.273873	-4.16722	0.007006	0.101213	-2.19
NONHSAT207168.1	1.364486	6.94055	4.167117	0.007006	0.101213	-2.19011
NONHSAT190127.1	1.580827	3.40076	4.165482	0.007018	0.10128	-2.19194
ENST00000662406.1	-1.0594	2.988558	-4.16487	0.007023	0.10128	-2.19262
MSTRG.53962.1	1.295213	2.131147	4.164818	0.007023	0.10128	-2.19268
MSTRG.52949.1	1.126248	1.997477	4.164726	0.007024	0.10128	-2.19278
ENST00000577423.2	1.098658	4.807445	4.164415	0.007026	0.10128	-2.19313
NONHSAT167758.1	1.838071	2.452317	4.164377	0.007027	0.10128	-2.19317
lnc-NRDE2-6:1	-1.22732	3.634235	-4.1643	0.007027	0.10128	-2.19325
NONHSAT197032.1	1.018648	1.594534	4.162975	0.007037	0.10135	-2.19474
NONHSAT174917.1	1.692285	2.917414	4.162588	0.00704	0.101376	-2.19517
lnc-ENDOD1-6:1	-1.7443	2.633154	-4.16062	0.007054	0.101484	-2.19737
T331246	-1.35422	3.271998	-4.15984	0.00706	0.101512	-2.19825
ENST00000658639.1	-1.81188	3.424945	-4.15966	0.007061	0.101512	-2.19844
lnc-GARS-8:1	-1.02152	1.790215	-4.15915	0.007065	0.101539	-2.19901
T306301	1.742299	2.438104	4.158115	0.007073	0.101628	-2.20018
lnc-SLC15A4-16:1	-1.72355	2.669418	-4.15733	0.007079	0.101628	-2.20106
ENST00000422600.1	1.401117	3.281473	4.156203	0.007087	0.101633	-2.20232
NONHSAT221833.1	1.008472	2.79045	4.153956	0.007104	0.1017	-2.20483
lnc-CBLN4-1:1	1.661136	2.378745	4.153175	0.00711	0.1017	-2.2057
NONHSAT196346.1	1.432968	1.916322	4.150929	0.007127	0.101738	-2.20822
lnc-COG2-1:1	1.372226	7.191168	4.150073	0.007133	0.101738	-2.20918
NONHSAT159704.1	1.658263	2.497875	4.149997	0.007134	0.101738	-2.20926
lnc-RDH13-2:9	1.358319	1.883767	4.149432	0.007138	0.101738	-2.2099
NONHSAT170145.1	1.346154	2.256258	4.14926	0.007139	0.101738	-2.21009
lnc-TMEM53-1:1	1.486754	3.11701	4.148978	0.007141	0.101738	-2.21041
NONHSAT190552.1	-1.3146	6.916679	-4.14891	0.007142	0.101738	-2.21048
NONHSAT150508.1	1.217832	4.955073	4.148308	0.007146	0.101742	-2.21116
MSTRG.7688.1	1.11328	1.985406	4.14826	0.007147	0.101742	-2.21121
ENST00000431813.1	-1.1497	6.023166	-4.14815	0.007147	0.101742	-2.21133
ENST00000665399.1	1.246438	8.340314	4.148018	0.007148	0.101742	-2.21148
NONHSAT179102.1	1.615437	4.758785	4.146798	0.007158	0.101772	-2.21285
NONHSAT190001.1	1.493296	4.022432	4.146456	0.00716	0.101772	-2.21323
NONHSAT179007.1	1.830438	2.437714	4.145923	0.007164	0.1018	-2.21383
MSTRG.19304.1	1.400023	5.007449	4.144653	0.007174	0.10189	-2.21525
lnc-ZNF615-1:1	-1.17887	3.296997	-4.14316	0.007185	0.101981	-2.21693
NONHSAT220502.1	1.121649	4.231964	4.142672	0.007189	0.102005	-2.21747
lnc-LEFTY1-5:1	1.019418	5.093585	4.142048	0.007193	0.102044	-2.21817
NONHSAT203634.1	1.574296	2.199485	4.140865	0.007202	0.102128	-2.2195
NONHSAT159422.1	1.149757	4.443456	4.139792	0.007211	0.102167	-2.2207
NONHSAT170742.1	1.104244	2.029254	4.139412	0.007213	0.102167	-2.22113
T300637	-1.64951	2.326817	-4.13935	0.007214	0.102167	-2.22121
lnc-DDX58-3:1	-1.73898	2.848203	-4.13842	0.007221	0.102204	-2.22225
NONHSAT165822.1	-1.40036	3.486608	-4.13818	0.007223	0.102216	-2.22252
lnc-IL37-5:1	-1.45896	2.313765	-4.135	0.007247	0.102412	-2.22608
NONHSAT215556.1	1.15758	1.873618	4.134343	0.007252	0.102457	-2.22682

NONHSAT218355.1	1.124528	1.647297	4.133584	0.007258	0.102502	-2.22767
lnc-EVX2-4:2	1.141501	3.626122	4.133291	0.00726	0.102502	-2.228
NONHSAT165146.1	2.067045	2.670648	4.132319	0.007268	0.102541	-2.22909
NONHSAT222021.1	-1.16397	1.706072	-4.1317	0.007272	0.102541	-2.22979
lnc-AGR3-6:1	1.766083	4.242558	4.131544	0.007274	0.102541	-2.22996
NONHSAT158555.1	1.088338	2.695626	4.13104	0.007277	0.102543	-2.23053
ENST00000507849.1	-1.1585	1.684741	-4.13094	0.007278	0.102543	-2.23064
lnc-MAP3K7-4:1	-1.25933	1.98235	-4.13059	0.007281	0.102543	-2.23103
ENST00000612806.1	-1.13232	7.148681	-4.12992	0.007286	0.102543	-2.23179
MSTRG.43268.10	2.339256	2.283973	4.129611	0.007288	0.102543	-2.23214
NONHSAT205340.1	1.81647	2.468334	4.128922	0.007294	0.102556	-2.23291
NONHSAT214589.1	1.709652	2.866782	4.128295	0.007298	0.102587	-2.23361
NONHSAT221914.1	-1.56137	1.875955	-4.12707	0.007308	0.102685	-2.23499
lnc-RRM2-2:1	-1.27734	4.27811	-4.12632	0.007314	0.10271	-2.23583
MSTRG.29347.1	1.120099	4.750836	4.126095	0.007315	0.10271	-2.23609
lnc-ZC3H12A-1:1	-1.11029	4.32188	-4.12588	0.007317	0.10271	-2.23633
MSTRG.61994.1	1.251995	8.242467	4.12585	0.007317	0.10271	-2.23636
lnc-BLOC1S6-2:1	1.153207	3.717176	4.124646	0.007327	0.102774	-2.23772
NONHSAT153152.1	1.780417	1.925401	4.124396	0.007329	0.102774	-2.238
lnc-SON-2:1	-1.00467	5.518391	-4.12278	0.007341	0.102888	-2.23982
NONHSAT174957.1	-1.0055	1.751461	-4.12182	0.007349	0.10291	-2.24089
lnc-HMBOX1-9:1	-1.4558	2.949947	-4.1217	0.007349	0.10291	-2.24103
NONHSAT158034.1	1.917979	2.200226	4.121417	0.007352	0.102923	-2.24135
MSTRG.45502.1	-1.40858	2.115175	-4.12066	0.007358	0.102954	-2.2422
MSTRG.48238.14	1.349865	1.799152	4.119781	0.007364	0.102955	-2.24319
lnc-RASGRP3-15:1	-1.12046	4.992229	-4.11802	0.007378	0.103027	-2.24517
MSTRG.8962.1	1.011342	3.921874	4.117965	0.007379	0.103027	-2.24523
ENST00000431656.1	-1.60826	2.59623	-4.11784	0.007379	0.103027	-2.24537
NONHSAT218026.1	2.457516	3.588595	4.117532	0.007382	0.103035	-2.24572
lnc-UPRT-4:1	1.196107	3.527766	4.116909	0.007387	0.103043	-2.24642
lnc-EXTL3-7:1	-1.06095	2.012153	-4.11677	0.007388	0.103043	-2.24657
ENST00000656456.1	1.034301	1.581741	4.11667	0.007389	0.103043	-2.24669
MSTRG.58001.1	-1.53883	3.351692	-4.11629	0.007392	0.103045	-2.24712
ENST00000654316.1	1.364082	2.65525	4.116067	0.007393	0.103051	-2.24737
NONHSAT199256.1	2.285086	3.338998	4.115931	0.007394	0.103051	-2.24752
NONHSAT203228.1	1.127108	1.629488	4.114432	0.007406	0.103152	-2.24921
NONHSAT215990.1	-1.04177	5.068392	-4.1142	0.007408	0.103152	-2.24947
LINC02539:10	1.686217	4.140709	4.114096	0.007409	0.103152	-2.24959
lnc-UPRT-6:7	-1.23761	2.826584	-4.11355	0.007413	0.103162	-2.25021
lnc-MMACHC-1:1	-1.26908	4.016644	-4.11298	0.007417	0.103189	-2.25085
NONHSAT160725.1	-1.08774	4.977158	-4.11264	0.00742	0.103208	-2.25123
lnc-SRI-1:1	1.167534	2.255699	4.112422	0.007422	0.103208	-2.25147
lnc-COPS2-5:1	-1.03764	9.177026	-4.11195	0.007426	0.103215	-2.252
MSTRG.40830.22	1.398778	2.446701	4.11141	0.00743	0.103248	-2.25261
T171330	1.160376	1.689568	4.110738	0.007435	0.103248	-2.25337
NONHSAT155526.1	1.17531	4.387176	4.110053	0.00744	0.10328	-2.25414
lnc-BAG3-3:1	1.298032	4.560151	4.109692	0.007443	0.10329	-2.25455
lnc-MED10-11:3	1.047635	3.573509	4.108931	0.007449	0.103316	-2.25541

ENST00000502049.3	-1.30709	3.444184	-4.10763	0.00746	0.103352	-2.25688
lnc-MRPL44-1:3	1.430014	1.842961	4.107259	0.007462	0.103352	-2.25729
lnc-POU6F2-3:1	1.867607	2.801611	4.107228	0.007463	0.103352	-2.25733
NONHSAT222287.1	-1.44353	2.13972	-4.10683	0.007466	0.103352	-2.25777
ENST00000602328.1	-1.7483	3.844334	-4.1068	0.007466	0.103352	-2.25781
lnc-TMEM135-10:1	1.739494	2.774743	4.106274	0.00747	0.103369	-2.2584
ENST00000415549.2	1.411948	2.546603	4.10616	0.007471	0.103369	-2.25853
NONHSAT154470.1	1.093295	1.922803	4.105706	0.007475	0.103392	-2.25904
ENST00000564251.1	1.161565	1.935157	4.105309	0.007478	0.103401	-2.25949
NONHSAT216339.1	1.747551	2.408289	4.105019	0.00748	0.103419	-2.25982
lnc-AGAP2-1:4	1.727554	4.04695	4.104098	0.007487	0.103462	-2.26085
ENST00000662737.1	1.577581	4.259934	4.103849	0.007489	0.103462	-2.26114
NONHSAT154487.1	1.236692	1.675813	4.10289	0.007497	0.103508	-2.26222
NONHSAT207325.1	1.400923	6.617803	4.102476	0.0075	0.103513	-2.26268
ENST00000558375.1	-1.24572	2.093105	-4.10199	0.007504	0.10354	-2.26324
NONHSAT203438.1	2.572468	3.036957	4.101971	0.007504	0.10354	-2.26325
lnc-RAB39A-2:1	-1.76318	4.640076	-4.10116	0.007511	0.103567	-2.26417
ENST00000649077.1	1.232377	1.944571	4.100729	0.007514	0.103567	-2.26466
NONHSAT180828.1	1.228518	5.347415	4.100698	0.007514	0.103567	-2.26469
NONHSAT193654.1	1.075178	7.612436	4.100126	0.007519	0.103587	-2.26534
NONHSAT218312.1	1.378117	8.690041	4.099921	0.007521	0.103595	-2.26557
NONHSAT190399.1	1.198349	4.322326	4.099497	0.007524	0.103597	-2.26605
MSTRG.41786.1	1.196747	4.969414	4.099179	0.007527	0.103597	-2.26641
NONHSAT211841.1	-1.2632	4.064023	-4.09906	0.007528	0.103597	-2.26654
NONHSAT221602.1	1.603796	1.851176	4.099057	0.007528	0.103597	-2.26654
lnc-ANXA1-4:2	2.403472	2.897963	4.098172	0.007535	0.10366	-2.26754
ENST00000451066.1	-1.10683	4.292213	-4.09739	0.007541	0.103668	-2.26843
lnc-PRDM1-7:1	1.87406	2.566791	4.097195	0.007542	0.103668	-2.26864
lnc-CYP2C8-3:1	1.644194	2.582773	4.096451	0.007548	0.103683	-2.26948
lnc-RPS19BP1-3:1	1.807195	1.968856	4.095475	0.007556	0.10373	-2.27059
ENST00000562873.1	1.008989	2.153632	4.0952	0.007558	0.103746	-2.2709
LINC00689:9	1.510194	2.049174	4.093877	0.007569	0.103821	-2.27239
ENST00000649143.1	1.120004	1.604546	4.091314	0.00759	0.104008	-2.27529
lnc-NKX3-2-3:1	-1.4235	3.36481	-4.09004	0.0076	0.104102	-2.27672
NR_125983	-1.52645	1.879566	-4.08915	0.007607	0.104144	-2.27773
lnc-C6orf48-1:1	1.264442	3.186177	4.088931	0.007609	0.104154	-2.27798
MSTRG.68491.5	1.099567	1.888365	4.088768	0.00761	0.104158	-2.27816
NONHSAT165056.1	1.137545	5.378434	4.088598	0.007611	0.104163	-2.27836
NONHSAT167889.1	1.000836	6.140853	4.088044	0.007616	0.104171	-2.27898
ENST00000662638.1	-1.68199	2.445077	-4.0878	0.007618	0.104171	-2.27926
ENST00000444488.1	-1.29114	4.724578	-4.08753	0.00762	0.104171	-2.27957
lnc-FAM241B-4:1	1.077007	1.635683	4.087021	0.007624	0.104171	-2.28014
MSTRG.22407.1	1.687512	2.57019	4.086732	0.007627	0.104172	-2.28047
lnc-SAXO1-1:1	-1.51794	4.141254	-4.08657	0.007628	0.104175	-2.28065
lnc-C11orf95-1:3	-1.24431	1.869033	-4.08594	0.007633	0.104216	-2.28136
ENST00000440492.1	-1.67222	5.268169	-4.08567	0.007635	0.104232	-2.28166
NONHSAT204519.1	1.651	3.952809	4.085213	0.007639	0.104248	-2.28218
lnc-WDR72-1:4	1.103173	1.636407	4.085147	0.007639	0.104248	-2.28226

ENST00000669546.1	-1.02338	4.029273	-4.08473	0.007643	0.10425	-2.28272
NONHSAT179548.1	-1.35685	1.900926	-4.08468	0.007643	0.10425	-2.28278
NONHSAT172593.1	-1.18767	2.106568	-4.08461	0.007644	0.10425	-2.28286
NONHSAT195522.1	-1.26163	1.722626	-4.08422	0.007647	0.10428	-2.28331
NONHSAT186573.1	2.025859	3.165169	4.083045	0.007656	0.104311	-2.28463
NR_036512	1.019383	10.40677	4.082732	0.007659	0.104325	-2.28499
NONHSAT215093.1	1.326812	1.721065	4.081806	0.007667	0.104364	-2.28604
lnc-ZNF708-14:3	1.008641	4.028402	4.080651	0.007676	0.104421	-2.28734
MSTRG.14640.1	1.097588	5.890503	4.079668	0.007684	0.104439	-2.28846
MSTRG.10705.1	1.704459	2.862459	4.07961	0.007684	0.104439	-2.28852
lnc-ANKRA2-10:1	-1.08567	3.729491	-4.07872	0.007692	0.104453	-2.28953
NONHSAT192694.1	1.225319	4.68919	4.078274	0.007695	0.10446	-2.29003
lnc-MCL1-5:2	-1.14334	3.283936	-4.0771	0.007705	0.104507	-2.29137
lnc-FYB1-1:2	-1.26964	2.357686	-4.077	0.007706	0.104507	-2.29147
ENST00000428920.2	1.445688	5.20598	4.076948	0.007706	0.104507	-2.29154
MSTRG.1129.1	-1.44119	2.357391	-4.07683	0.007707	0.104507	-2.29166
lnc-SH3TC2-3:1	-1.16563	2.146128	-4.07671	0.007708	0.104507	-2.2918
lnc-BRD7-4:1	-1.0197	4.27742	-4.07639	0.007711	0.104515	-2.29217
NONHSAT178397.1	-1.46905	3.154359	-4.07619	0.007712	0.104522	-2.29239
lnc-REG1B-9:1	1.970323	2.997538	4.074889	0.007723	0.104576	-2.29387
ENST00000425983.1	1.312179	10.615	4.073762	0.007732	0.104622	-2.29514
NONHSAT173144.1	1.090891	5.353925	4.07303	0.007738	0.104663	-2.29597
NONHSAT197536.1	-1.08717	4.097366	-4.07216	0.007746	0.104704	-2.29696
lnc-KHNYN-5:3	-1.54995	2.659125	-4.07171	0.007749	0.104718	-2.29746
lnc-SYTL3-150:2	1.207552	8.739974	4.071651	0.00775	0.104718	-2.29753
MSTRG.33258.20	-1.02562	5.181279	-4.07059	0.007758	0.104795	-2.29874
MSTRG.62109.1	1.27115	2.548174	4.070331	0.007761	0.104797	-2.29903
NONHSAT164334.1	-1.18452	3.12557	-4.07032	0.007761	0.104797	-2.29905
NONHSAT170323.1	1.146978	2.847974	4.070026	0.007763	0.104811	-2.29938
NONHSAT208086.1	1.105818	1.652085	4.067433	0.007785	0.104997	-2.30231
lnc-TEX10-1:1	2.047031	3.786288	4.067393	0.007785	0.104997	-2.30236
NONHSAT193293.1	1.189969	2.596696	4.066973	0.007788	0.105013	-2.30284
lnc-ZNF652-2:1	-1.43659	3.224861	-4.06687	0.007789	0.105013	-2.30296
NONHSAT168663.1	1.399748	1.891687	4.066311	0.007794	0.105047	-2.30359
NONHSAT202399.1	-1.16976	1.757545	-4.06593	0.007797	0.10507	-2.30402
ENST00000485218.1	1.785478	4.51391	4.065515	0.0078	0.10507	-2.30449
lnc-CHPF2-4:1	-1.80618	2.16129	-4.06527	0.007802	0.10507	-2.30477
NONHSAT190142.1	1.297103	3.916063	4.064952	0.007805	0.10507	-2.30513
lnc-EIF1AY-4:1	-1.10972	5.033056	-4.06491	0.007805	0.10507	-2.30518
lnc-MCM10-1:2	1.580321	4.723004	4.06453	0.007809	0.10507	-2.30561
MSTRG.28913.1	-1.42136	3.349498	-4.06409	0.007812	0.105099	-2.3061
NONHSAT205051.1	1.25955	3.696364	4.062606	0.007825	0.105165	-2.30779
NONHSAT215103.1	1.57258	7.470845	4.062492	0.007826	0.105165	-2.30792
NONHSAT178897.1	1.281046	3.352781	4.061997	0.00783	0.105165	-2.30848
NONHSAT164794.1	1.364751	2.341592	4.060382	0.007843	0.105234	-2.31031
lnc-KAT7-2:4	-1.4452	2.702544	-4.06017	0.007845	0.105234	-2.31056
MSTRG.46430.6	1.871249	2.797567	4.057467	0.007868	0.105258	-2.31362
NONHSAT170464.1	1.974531	2.344986	4.057423	0.007868	0.105258	-2.31367

MSTRG.33927.1	-1.29123	5.152142	-4.05721	0.00787	0.105265	-2.31391
ENST00000518217.1	1.788086	1.957686	4.056853	0.007873	0.105265	-2.31432
ENST00000661711.1	1.208531	3.239862	4.056617	0.007875	0.105265	-2.31459
lnc-ZMAT3-8:1	-1.27702	4.073866	-4.05615	0.007879	0.105268	-2.31512
lnc-ABHD17B-6:1	-1.16309	4.746718	-4.05498	0.007888	0.105268	-2.31644
ENST00000641271.1	1.266994	4.584805	4.054763	0.00789	0.105268	-2.31669
NONHSAT172103.1	1.759725	1.900449	4.054744	0.00789	0.105268	-2.31671
LINC01052:1	1.15556	1.996115	4.054553	0.007892	0.105268	-2.31693
MSTRG.49961.1	-1.25277	4.064669	-4.05386	0.007898	0.105268	-2.31771
lnc-SGK1-9:1	1.088219	4.18667	4.053718	0.007899	0.105268	-2.31788
lnc-RASA1-2:5	1.463444	2.721521	4.053647	0.0079	0.105268	-2.31796
NONHSAT201986.1	1.486163	3.183857	4.051281	0.00792	0.105418	-2.32065
lnc-PSMG4-17:1	1.501184	2.332699	4.050911	0.007923	0.105432	-2.32107
NONHSAT184695.1	-1.14277	1.671806	-4.0498	0.007932	0.105452	-2.32234
NONHSAT210396.1	1.281277	4.396561	4.048616	0.007942	0.105526	-2.32368
NONHSAT155110.1	1.43659	1.820912	4.048236	0.007945	0.105526	-2.32411
ENST00000660474.1	-1.03167	5.728888	-4.04822	0.007945	0.105526	-2.32413
NONHSAT193771.1	1.719087	2.930739	4.047532	0.007951	0.105551	-2.32491
ENST00000641463.1	-1.74727	2.588335	-4.04728	0.007954	0.105563	-2.3252
NONHSAT211948.1	1.275835	3.915598	4.047032	0.007956	0.105569	-2.32548
lnc-VIRMA-3:1	-2.05277	2.778637	-4.04687	0.007957	0.105569	-2.32566
MSTRG.56755.1	1.399589	1.785357	4.046401	0.007961	0.105574	-2.3262
ENST00000660617.1	1.228776	4.034593	4.046168	0.007963	0.105576	-2.32646
NONHSAT222639.1	-1.2699	2.657392	-4.04464	0.007976	0.105711	-2.32819
MSTRG.31588.7	1.706544	2.806508	4.043576	0.007985	0.105722	-2.32941
lnc-NDC80-5:1	1.607328	2.257171	4.043399	0.007986	0.105722	-2.32961
MSTRG.23543.1	1.010915	4.627258	4.041699	0.008001	0.105838	-2.33155
ENST00000450936.1	-1.47687	3.969939	-4.03978	0.008017	0.105951	-2.33374
NONHSAT154068.1	1.075413	4.737334	4.039201	0.008022	0.105956	-2.33439
lnc-PIEZO2-6:3	1.051188	3.604745	4.038939	0.008025	0.105956	-2.33469
lnc-C3orf30-11:1	1.501716	2.994389	4.038156	0.008031	0.105996	-2.33558
ENST00000420548.1	-1.65647	2.406502	-4.03555	0.008054	0.106062	-2.33854
NONHSAT206064.1	1.20357	1.710175	4.035188	0.008057	0.106062	-2.33896
NONHSAT219993.1	1.13966	2.467699	4.035041	0.008058	0.106062	-2.33913
lnc-PTGR1-2:1	1.396118	4.876686	4.035004	0.008058	0.106062	-2.33917
NR_120430	-1.25747	5.205317	-4.03432	0.008064	0.106084	-2.33995
NONHSAT157805.1	1.426563	1.732988	4.033502	0.008071	0.106148	-2.34088
MSTRG.49733.1	1.036128	2.773907	4.032745	0.008078	0.106148	-2.34174
lnc-ADAMTS9-5:1	-1.07285	4.307611	-4.03214	0.008083	0.106179	-2.34243
lnc-CROCC-6:2	-1.37691	2.911321	-4.03122	0.008091	0.106227	-2.34348
lnc-GREB1-14:1	1.300332	1.953826	4.030521	0.008097	0.106256	-2.34428
NONHSAT158563.1	1.751866	3.216698	4.030496	0.008097	0.106256	-2.3443
lnc-TMEM114-7:1	-1.06119	5.167236	-4.03012	0.008101	0.106264	-2.34473
lnc-MYLK4-16:1	1.437717	2.857805	4.02649	0.008132	0.10649	-2.34887
ENST00000422042.1	-1.69785	3.758831	-4.02591	0.008137	0.106514	-2.34953
MSTRG.7437.6	1.13929	6.390686	4.025741	0.008139	0.10652	-2.34973
NONHSAT179281.1	1.306051	2.901773	4.025093	0.008144	0.106566	-2.35046
NONHSAT214795.1	1.211733	5.687895	4.024686	0.008148	0.106579	-2.35093

ENST00000430111.1	-1.63732	2.783592	-4.02461	0.008148	0.106579	-2.35101
MSTRG.37165.1	-1	1.591958	-4.02409	0.008153	0.106612	-2.35161
lnc-SLC36A2-1:1	1.185347	3.741885	4.022153	0.00817	0.106686	-2.35382
MSTRG.43901.1	-1.01798	1.616006	-4.02166	0.008174	0.106704	-2.35438
MSTRG.27493.20	-1.16038	3.795959	-4.02051	0.008184	0.106753	-2.35569
ENST00000661265.1	-1.17095	1.81432	-4.0203	0.008186	0.106753	-2.35594
ENST00000671014.1	1.07007	1.748365	4.020027	0.008189	0.106753	-2.35625
NONHSAT186672.1	1.209839	3.365082	4.018837	0.008199	0.106825	-2.35761
lnc-LRP1B-4:7	1.701532	2.397848	4.017259	0.008213	0.106895	-2.35941
NONHSAT157050.1	1.688589	2.0848	4.015679	0.008227	0.106993	-2.36121
MSTRG.292.1	1.362997	2.084601	4.015124	0.008232	0.107021	-2.36184
NONHSAT216320.1	-1.65327	3.393106	-4.01507	0.008232	0.107021	-2.3619
MSTRG.64486.1	1.337186	2.663582	4.014412	0.008238	0.107045	-2.36266
T242581	1.080826	4.380169	4.01405	0.008241	0.107045	-2.36307
NONHSAT172167.1	1.123273	6.542903	4.013923	0.008242	0.107045	-2.36322
NONHSAT200938.1	1.361256	2.649975	4.01366	0.008245	0.107045	-2.36352
lnc-UBAC2-5:1	-1.05331	4.87614	-4.01229	0.008257	0.107123	-2.36508
MSTRG.53793.1	1.065966	1.600813	4.011887	0.00826	0.107126	-2.36554
NONHSAT169482.1	1.1751	1.676008	4.011875	0.00826	0.107126	-2.36556
NR_109771	1.186859	7.438677	4.010516	0.008272	0.107186	-2.36711
ENST00000611726.1	-1.06762	4.365456	-4.00979	0.008279	0.107219	-2.36795
ENST00000565300.2	1.665091	4.650706	4.009042	0.008286	0.107273	-2.3688
lnc-SMIM23-1:1	1.799827	4.558396	4.006912	0.008305	0.107394	-2.37123
NONHSAT215224.1	1.33887	1.773867	4.004235	0.008328	0.107496	-2.37429
lnc-PIGM-4:1	1.188472	2.156534	4.000664	0.00836	0.107813	-2.37838
NONHSAT207883.1	1.077146	3.308324	3.999859	0.008368	0.107818	-2.3793
NONHSAT203390.1	1.351077	2.131781	3.999839	0.008368	0.107818	-2.37932
MSTRG.35148.1	1.226128	1.868291	3.999546	0.00837	0.107818	-2.37966
lnc-DDHD1-1:1	1.195971	3.194185	3.999197	0.008374	0.10782	-2.38006
NONHSAT220293.1	1.202071	3.75877	3.998738	0.008378	0.107839	-2.38058
NONHSAT202618.1	-1.64918	3.047589	-3.99806	0.008384	0.107881	-2.38136
MSTRG.50297.2	1.151166	9.534498	3.997439	0.008389	0.107905	-2.38207
NONHSAT218080.1	1.890859	3.205138	3.997178	0.008392	0.107905	-2.38237
NONHSAT217235.1	1.710368	2.538519	3.997014	0.008393	0.107905	-2.38256
lnc-ZBTB7C-4:1	-1.07751	4.844121	-3.99673	0.008396	0.107911	-2.38288
MSTRG.46476.3	-1.02539	2.311433	-3.99591	0.008403	0.107941	-2.38382
NONHSAT208513.1	-1.25509	1.751364	-3.99587	0.008404	0.107941	-2.38387
NONHSAT199389.1	1.881841	2.533062	3.994861	0.008413	0.108017	-2.38503
NONHSAT162536.1	-1.41139	1.917987	-3.99404	0.00842	0.108086	-2.38597
MSTRG.60712.1	1.71941	2.734177	3.993555	0.008425	0.108114	-2.38652
MSTRG.2969.1	1.418581	1.811549	3.99275	0.008432	0.108138	-2.38744
NONHSAT222139.1	1.512153	2.297814	3.992356	0.008435	0.108138	-2.3879
lnc-PCDH17-1:1	1.312134	1.68536	3.992206	0.008437	0.108138	-2.38807
MSTRG.29873.1	1.616055	3.537503	3.991875	0.00844	0.108138	-2.38845
ENST00000418574.3	-1.37086	2.961508	-3.99181	0.00844	0.108138	-2.38852
NONHSAT205859.1	1.536276	1.877199	3.990628	0.008451	0.108221	-2.38988
ENST00000522896.1	1.52988	2.202327	3.989971	0.008457	0.108242	-2.39063
lnc-CLIC5-2:1	-1.781	2.764477	-3.9896	0.00846	0.108272	-2.39105

NONHSAT208011.1	-1.11397	4.903735	-3.98879	0.008468	0.108338	-2.39198
lnc-YES1-11:1	1.126945	1.759075	3.987688	0.008478	0.108402	-2.39325
NONHSAT154158.1	-1.27174	2.831676	-3.98753	0.008479	0.108402	-2.39343
NONHSAT156519.1	1.229826	3.836478	3.987178	0.008482	0.108403	-2.39383
lnc-DYNC1H1-4:1	-1.39722	2.810277	-3.98701	0.008484	0.108409	-2.39403
NONHSAT157738.1	1.106532	3.214956	3.98669	0.008487	0.108416	-2.39439
NONHSAT154282.1	1.310856	7.590467	3.986551	0.008488	0.108416	-2.39455
lnc-MAGEA1-1:2	2.00141	2.565973	3.986449	0.008489	0.108416	-2.39467
ENST00000453155.1	1.172434	4.566451	3.985735	0.008496	0.108442	-2.39549
NONHSAT200356.1	-1.02778	4.011453	-3.98466	0.008506	0.108449	-2.39672
lnc-CDK13-6:1	1.982354	3.06054	3.984658	0.008506	0.108449	-2.39672
NONHSAT184311.1	-1.22173	1.92906	-3.98455	0.008507	0.108449	-2.39685
lnc-ZC3H11B-10:1	-1.21749	2.110881	-3.98429	0.008509	0.108449	-2.39715
NONHSAT161030.1	1.536397	4.256411	3.984197	0.00851	0.108449	-2.39725
lnc-ALDH1L2-1:5	-1.08681	4.057121	-3.98413	0.00851	0.108449	-2.39733
lnc-RNF43-1:1	1.256697	4.755517	3.983902	0.008512	0.108449	-2.39759
MSTRG.8195.1	-1.06237	3.22574	-3.9838	0.008513	0.108449	-2.39771
ENST00000437515.1	-1.31617	1.894775	-3.9825	0.008525	0.108541	-2.3992
lnc-LARP4-5:1	-1.63461	2.77019	-3.97931	0.008555	0.108722	-2.40286
NONHSAT223731.1	1.318403	1.723371	3.979215	0.008556	0.108722	-2.40297
T080916	-1.26417	1.924134	-3.97884	0.008559	0.108722	-2.4034
NONHSAT154069.1	1.4996	4.482701	3.977886	0.008568	0.108759	-2.4045
NONHSAT186902.1	1.74691	2.096071	3.9769	0.008577	0.108809	-2.40563
NONHSAT189364.1	1.596955	2.367399	3.976366	0.008582	0.108846	-2.40624
NR_037694	2.142766	2.986615	3.975127	0.008593	0.108846	-2.40766
NONHSAT206818.1	1.241578	1.717062	3.974694	0.008597	0.108846	-2.40816
ENST00000656212.1	-1.34569	4.215659	-3.97416	0.008602	0.108846	-2.40877
MSTRG.7002.1	-1.12546	1.694085	-3.97337	0.00861	0.108888	-2.40969
lnc-ENO1-5:1	-1.01183	3.064772	-3.97274	0.008615	0.108928	-2.4104
MSTRG.38906.1	-1.78956	2.281364	-3.97252	0.008618	0.108941	-2.41066
ENST00000584078.1	-1.47051	2.074585	-3.97202	0.008622	0.108958	-2.41123
NONHSAT191786.1	1.445554	1.826353	3.970929	0.008632	0.108984	-2.41249
NONHSAT193536.1	-1.53582	3.590868	-3.97076	0.008634	0.108984	-2.41269
NONHSAT181130.1	1.430171	3.026052	3.97041	0.008637	0.108997	-2.41309
NONHSAT166059.1	2.439399	2.949194	3.969362	0.008647	0.109049	-2.41429
lnc-MINDY2-7:1	-1.00229	4.491932	-3.96932	0.008647	0.109049	-2.41434
MSTRG.10683.2	-1.12188	2.216176	-3.96928	0.008648	0.109049	-2.41439
MSTRG.48286.1	-1.46061	3.071226	-3.96908	0.00865	0.109058	-2.41462
MSTRG.67708.1	1.138747	6.048587	3.968196	0.008658	0.109132	-2.41563
ENST00000450893.1	1.39559	3.671218	3.967905	0.00866	0.109132	-2.41597
lnc-ROPN1L-7:1	-1.04759	4.988568	-3.96607	0.008678	0.109276	-2.41807
NONHSAT194275.1	-1.3724	4.957748	-3.96544	0.008684	0.109297	-2.41881
ENST00000608671.1	-1.02635	3.861947	-3.96465	0.008691	0.109331	-2.41971
lnc-LRMDA-14:1	1.408546	2.189586	3.964649	0.008691	0.109331	-2.41971
NR_109890	1.154124	5.3414	3.964474	0.008693	0.109331	-2.41991
T159314	-1.12889	2.271105	-3.96429	0.008694	0.109331	-2.42013
ENST00000423568.2	1.455472	2.415564	3.963909	0.008698	0.109331	-2.42056
T104965	1.390872	2.927658	3.96376	0.008699	0.109331	-2.42073

NONHSAT161377.1	1.732111	1.983694	3.963217	0.008704	0.10936	-2.42136
T219085	1.442084	2.194406	3.962654	0.00871	0.10936	-2.42201
NONHSAT222168.1	-1.48849	3.567146	-3.96109	0.008724	0.109387	-2.42381
ENST00000562900.1	1.449466	2.7851	3.960774	0.008727	0.109411	-2.42417
NONHSAT150736.1	1.5465	2.319667	3.960282	0.008732	0.109442	-2.42474
lnc-FRRS1-3:1	-1.56799	2.702945	-3.95951	0.008739	0.109506	-2.42563
MSTRG.38386.2	1.500294	4.024423	3.955968	0.008773	0.10983	-2.42971
ENST00000648709.1	1.09639	1.976518	3.955576	0.008777	0.109863	-2.43016
NONHSAT198358.1	2.39998	2.963601	3.954922	0.008783	0.109927	-2.43091
lnc-NUCB2-10:1	-1.05788	2.861358	-3.95427	0.008789	0.109927	-2.43166
NONHSAT179017.1	-1.31392	2.067615	-3.9536	0.008795	0.109949	-2.43243
ENST00000569873.1	-1.62864	2.460994	-3.95346	0.008797	0.109949	-2.4326
lnc-CFH-4:2	1.085837	1.615144	3.952203	0.008809	0.110046	-2.43404
lnc-CCR4-3:1	2.191998	2.830103	3.95074	0.008823	0.110163	-2.43573
ENST00000670424.1	1.2417	2.180638	3.949806	0.008831	0.110189	-2.43681
ENST00000445000.2	-1.58929	2.522361	-3.94968	0.008833	0.110189	-2.43695
lnc-ZMYND19-2:1	-1.21469	2.630306	-3.94926	0.008837	0.110189	-2.43743
lnc-TMEM123-2:1	1.33267	1.753837	3.949122	0.008838	0.110189	-2.4376
lnc-IL12RB2-6:1	1.031296	5.19677	3.948907	0.00884	0.110189	-2.43784
MSTRG.35407.1	1.112065	5.854054	3.948829	0.008841	0.110189	-2.43793
NONHSAT203637.1	1.15076	3.020818	3.948503	0.008844	0.110214	-2.43831
lnc-AKAIN1-9:1	-1.31509	6.25627	-3.9483	0.008846	0.110225	-2.43854
NONHSAT176042.1	1.476152	1.816406	3.948181	0.008847	0.110225	-2.43868
NONHSAT158053.1	-1.00793	4.694194	-3.94737	0.008855	0.110254	-2.43962
MSTRG.45063.1	1.067761	5.965307	3.946873	0.00886	0.1103	-2.44019
NONHSAT224375.1	-1.08775	4.115565	-3.94442	0.008883	0.110429	-2.44302
lnc-UTS2R-1:1	-1.53419	2.542454	-3.94401	0.008887	0.110446	-2.44349
NONHSAT198660.1	1.403132	2.05627	3.944009	0.008887	0.110446	-2.44349
NONHSAT173193.1	1.028237	2.056041	3.943658	0.00889	0.110446	-2.4439
NONHSAT193899.1	-1.627	1.980116	-3.94345	0.008892	0.110446	-2.44415
MSTRG.8150.1	1.158721	3.964822	3.942478	0.008902	0.110446	-2.44526
NONHSAT210262.1	1.153616	1.599777	3.942459	0.008902	0.110446	-2.44528
ENST00000648393.1	2.244665	3.379325	3.941507	0.008911	0.110496	-2.44638
ENST00000453367.1	1.268762	2.52643	3.940622	0.00892	0.110557	-2.4474
MSTRG.45548.1	-1.64006	3.076187	-3.94004	0.008925	0.110587	-2.44808
NONHSAT191779.1	2.016974	2.636867	3.939592	0.00893	0.110613	-2.44859
NONHSAT205159.1	1.550579	2.27036	3.939263	0.008933	0.110635	-2.44897
ENST00000418927.2	-1.3803	3.310393	-3.93823	0.008943	0.11065	-2.45017
MSTRG.34704.1	-1.59029	2.170507	-3.93732	0.008952	0.110661	-2.45122
NONHSAT158592.1	1.186262	2.416644	3.937036	0.008954	0.110675	-2.45154
NONHSAT172942.1	1.147151	3.640621	3.934394	0.00898	0.110817	-2.4546
NONHSAT160092.1	-1.31047	1.924537	-3.93438	0.00898	0.110817	-2.45461
lnc-ZNF585B-2:1	-1.06932	5.062139	-3.9334	0.00899	0.11085	-2.45575
NONHSAT187290.1	1.123112	1.679669	3.933064	0.008993	0.110853	-2.45613
NONHSAT201842.1	1.030849	3.720507	3.932715	0.008996	0.110878	-2.45654
lnc-P2RX7-2:1	1.426509	2.093406	3.930993	0.009013	0.110967	-2.45853
ENST00000667899.1	1.371826	4.342542	3.930388	0.009019	0.110999	-2.45923
NONHSAT180461.1	-1.11412	4.659786	-3.92974	0.009026	0.111025	-2.45997

MSTRG.45766.1	1.445857	2.657816	3.92972	0.009026	0.111025	-2.46
NONHSAT159456.1	1.659042	1.915981	3.929057	0.009032	0.111057	-2.46077
ENST00000533740.1	-1.53089	5.769914	-3.92898	0.009033	0.111057	-2.46085
lnc-CREBZF-6:1	1.368854	1.998786	3.928635	0.009036	0.111057	-2.46125
NONHSAT169623.1	-1.22433	4.182887	-3.92724	0.00905	0.11114	-2.46287
ENST00000412654.1	1.374735	4.153765	3.927193	0.009051	0.11114	-2.46292
ENST00000418747.2	-1.32503	3.436738	-3.92602	0.009062	0.11121	-2.46428
lnc-MAK16-1:6	1.40271	1.751209	3.925818	0.009064	0.11121	-2.46451
lnc-CAB39-1:1	-1.03763	3.880679	-3.92563	0.009066	0.11121	-2.46474
ENST00000636059.1	1.504732	2.302243	3.924442	0.009078	0.11129	-2.4661
ENST00000428903.1	1.511452	2.603256	3.924378	0.009078	0.11129	-2.46618
lnc-ARHGDIB-4:1	1.27629	1.725412	3.923961	0.009082	0.11129	-2.46666
NONHSAT197035.1	-1.00299	4.28401	-3.92299	0.009092	0.111364	-2.46779
lnc-ZNF835-3:1	-1.11233	1.835813	-3.92204	0.009101	0.11141	-2.46889
lnc-PTGS2-7:1	-1.0874	4.120262	-3.92194	0.009102	0.11141	-2.469
NONHSAT218656.1	1.661683	3.682566	3.921732	0.009104	0.111421	-2.46924
NONHSAT164737.1	1.084076	2.834814	3.921442	0.009107	0.111429	-2.46958
lnc-GLI3-5:1	-1.06436	3.946592	-3.92109	0.009111	0.111445	-2.46999
lnc-C11orf53-3:1	1.01792	1.584982	3.920221	0.009119	0.111536	-2.47099
NONHSAT200605.1	-1.26985	2.452886	-3.91972	0.009124	0.111574	-2.47157
MSTRG.33020.1	-1.79427	2.492544	-3.91907	0.009131	0.111622	-2.47232
lnc-NUP35-4:2	-1.50699	2.728742	-3.9188	0.009133	0.11164	-2.47263
MSTRG.70287.1	-1.71705	3.297024	-3.91753	0.009146	0.111727	-2.4741
T360844	1.879024	2.268215	3.916833	0.009153	0.111772	-2.47491
lnc-CHAT-1:1	-2.16896	3.441754	-3.91683	0.009153	0.111772	-2.47492
lnc-C15orf41-17:1	2.087531	2.700947	3.916083	0.00916	0.111795	-2.47578
NONHSAT152664.1	1.007283	6.012201	3.915243	0.009169	0.111836	-2.47676
lnc-ICOSLG-6:12	1.873545	2.102155	3.914066	0.00918	0.111841	-2.47812
lnc-ESRRG-3:1	-1.53055	3.13258	-3.91336	0.009188	0.111841	-2.47894
NONHSAT193339.1	-1.29631	2.590089	-3.91332	0.009188	0.111841	-2.47898
NONHSAT174534.1	-1.72252	1.995273	-3.91127	0.009208	0.111961	-2.48136
NONHSAT179296.1	1.540235	2.247371	3.910855	0.009213	0.111971	-2.48184
lnc-CNTN2-3:1	-1.52905	3.60715	-3.91001	0.009221	0.112007	-2.48282
lnc-SF3B2-1:1	1.448571	4.056828	3.908992	0.009231	0.112051	-2.484
lnc-ANO3-6:1	1.411809	1.88656	3.908948	0.009232	0.112051	-2.48405
ENST00000417957.1	1.331843	3.085509	3.908045	0.009241	0.112107	-2.4851
NONHSAT154935.1	1.017326	4.628587	3.906581	0.009256	0.112215	-2.4868
lnc-VTI1A-8:1	-1.04372	5.696755	-3.90628	0.009259	0.112225	-2.48714
ENST00000671634.1	1.016111	4.139383	3.902693	0.009295	0.112463	-2.49131
NONHSAT172765.1	1.007584	4.693834	3.902408	0.009298	0.112463	-2.49164
lnc-ERI1-8:2	-1.03855	1.924144	-3.9022	0.0093	0.112468	-2.49188
NONHSAT190550.1	1.40744	3.733755	3.902143	0.0093	0.112468	-2.49195
NR_125907	1.085016	2.801997	3.901285	0.009309	0.112498	-2.49294
lnc-RGSL1-7:1	-1.16715	4.980419	-3.90107	0.009311	0.112501	-2.4932
T340968	1.093584	8.150722	3.900995	0.009312	0.112501	-2.49328
ENST00000457477.2	1.112582	1.888453	3.900374	0.009318	0.112535	-2.494
NONHSAT201534.1	1.632176	2.3319	3.899876	0.009323	0.11255	-2.49458
NONHSAT215515.1	1.012069	5.028979	3.899471	0.009327	0.112553	-2.49505

lnc-PFN2-4:1	-1.01993	4.333054	-3.89859	0.009336	0.112581	-2.49607
lnc-MYB-3:3	1.313534	2.079214	3.896046	0.009362	0.11279	-2.49903
MSTRG.54962.1	-1.10318	3.542718	-3.8958	0.009365	0.11279	-2.49932
ENST00000645557.1	1.911836	3.09938	3.895661	0.009366	0.11279	-2.49948
NONHSAT174536.1	1.010237	4.974774	3.894822	0.009375	0.112841	-2.50045
lnc-PLPP4-4:3	1.083611	3.800771	3.894159	0.009382	0.112898	-2.50122
MSTRG.65941.1	-1.22766	3.185904	-3.89413	0.009382	0.112898	-2.50126
ENST00000555316.1	-1.37766	3.637727	-3.89402	0.009383	0.112898	-2.50138
lnc-HEBP1-1:1	1.003762	4.144501	3.893607	0.009387	0.112924	-2.50186
ENST00000423593.1	1.113944	1.605575	3.893224	0.009391	0.112944	-2.50231
NONHSAT204618.1	1.360176	2.338082	3.891388	0.00941	0.113055	-2.50444
lnc-SLC39A10-7:1	1.673749	2.038416	3.888546	0.009439	0.113222	-2.50775
NONHSAT194206.1	1.263937	2.971447	3.888346	0.009441	0.113222	-2.50798
lnc-LUZP2-5:1	1.516702	1.850201	3.888303	0.009442	0.113222	-2.50803
MSTRG.69484.1	-1.92737	2.796952	-3.8849	0.009477	0.11346	-2.51199
NONHSAT218311.1	2.098018	3.228697	3.884778	0.009478	0.113461	-2.51213
ENST00000670832.1	-1.54943	4.407428	-3.88332	0.009493	0.113568	-2.51382
NONHSAT171875.1	1.990598	3.154587	3.883117	0.009495	0.113573	-2.51406
NONHSAT200526.1	1.076285	1.746364	3.882695	0.0095	0.113581	-2.51455
ENST00000512599.1	1.048571	7.509676	3.882436	0.009502	0.113581	-2.51486
T284768	-1.09837	1.752204	-3.88244	0.009502	0.113581	-2.51486
lnc-MAP4-1:1	-1.30887	2.77798	-3.88147	0.009512	0.113589	-2.51598
lnc-BIK-2:1	1.188841	10.5758	3.880101	0.009527	0.113683	-2.51757
MSTRG.21844.1	-1.46488	2.171143	-3.87896	0.009538	0.113779	-2.5189
MSTRG.183.1	-1.06335	3.651128	-3.87841	0.009544	0.11382	-2.51954
lnc-USP6NL-18:1	1.009763	1.616546	3.876773	0.009561	0.113937	-2.52145
T293184	-1.22767	2.903839	-3.87658	0.009563	0.113937	-2.52167
lnc-FUT8-3:1	1.361333	1.773472	3.876533	0.009564	0.113937	-2.52173
NONHSAT172452.1	1.098893	1.629889	3.875988	0.00957	0.113961	-2.52236
NONHSAT199019.1	1.404305	1.778804	3.875806	0.009571	0.11397	-2.52258
ENST00000456585.5	1.00804	3.756071	3.875305	0.009577	0.114005	-2.52316
ENST00000619338.1	-1.62304	2.326061	-3.87448	0.009585	0.114068	-2.52412
ENST00000423515.2	1.384894	1.713784	3.874273	0.009587	0.11408	-2.52436
MSTRG.33765.1	1.511184	2.600181	3.873871	0.009592	0.114087	-2.52483
NONHSAT214975.1	1.184721	1.965558	3.873742	0.009593	0.114087	-2.52498
T014019	-1.46716	2.458617	-3.87363	0.009594	0.114087	-2.52511
NONHSAT178920.1	1.104452	3.663282	3.873628	0.009594	0.114087	-2.52511
lnc-SSC5D-1:1	1.270026	2.309652	3.873604	0.009595	0.114087	-2.52514
NONHSAT167453.1	1.214522	3.720389	3.872995	0.009601	0.114115	-2.52585
lnc-AREG-1:6	-1.56609	1.935339	-3.87294	0.009601	0.114115	-2.52591
lnc-EDARADD-4:1	1.325722	1.754811	3.872917	0.009602	0.114115	-2.52594
MSTRG.2157.1	-1.14839	1.898762	-3.87262	0.009605	0.114138	-2.52628
MSTRG.63542.1	1.194176	1.772235	3.872436	0.009607	0.114145	-2.5265
NONHSAT197504.1	1.343088	3.001192	3.872351	0.009608	0.114145	-2.5266
NONHSAT158046.1	1.751239	8.025779	3.872117	0.00961	0.114161	-2.52688
lnc-CCL4-1:1	-1.0987	5.005704	-3.87184	0.009613	0.114176	-2.5272
MSTRG.46638.1	-1.53416	2.265094	-3.87114	0.00962	0.114176	-2.52802
lnc-ITPR3-9:1	2.083799	3.386434	3.871031	0.009622	0.114176	-2.52814

lnc-STRBP-10:1	-1.27646	2.904614	-3.86995	0.009633	0.114266	-2.5294
NONHSAT204178.1	1.090926	3.488322	3.869572	0.009637	0.114266	-2.52984
ENST00000670736.1	1.203657	3.320309	3.8695	0.009638	0.114266	-2.52993
MSTRG.40406.1	-1.37469	3.69838	-3.86946	0.009638	0.114266	-2.52997
lnc-KIDINS220-16:1	1.444109	3.484823	3.868951	0.009643	0.114266	-2.53057
ENST00000648887.1	1.37987	2.398083	3.868706	0.009646	0.114266	-2.53085
MSTRG.45925.1	-1.70701	2.23429	-3.86827	0.009651	0.114266	-2.53136
lnc-EPHA4-3:1	-1.39858	1.856647	-3.86816	0.009652	0.114266	-2.53149
ENST00000649345.1	2.050453	3.199356	3.867126	0.009663	0.114355	-2.5327
NONHSAT202594.1	1.310462	2.437296	3.866345	0.009671	0.114387	-2.53361
lnc-HPSE-1:1	-1.21626	2.02874	-3.86571	0.009678	0.114438	-2.53435
NONHSAT154722.1	-1.08738	1.731366	-3.86515	0.009684	0.114469	-2.535
NONHSAT205066.1	1.19312	3.435751	3.864915	0.009686	0.114484	-2.53527
LINC01619:10	-1.22056	2.301864	-3.86466	0.009689	0.114502	-2.53557
NONHSAT156472.1	1.193713	3.89596	3.863251	0.009704	0.114545	-2.53722
MSTRG.38860.1	1.666509	2.462665	3.861249	0.009725	0.114676	-2.53955
NONHSAT202036.1	-1.212	2.587208	-3.85948	0.009744	0.114793	-2.54162
MSTRG.5378.1	-1.01134	4.224021	-3.85946	0.009744	0.114793	-2.54164
T078794	1.482129	2.351905	3.858526	0.009754	0.11486	-2.54273
lnc-STK24-2:1	-1.36526	3.299146	-3.8584	0.009756	0.11486	-2.54288
lnc-CBX4-2:1	1.297621	5.453149	3.857942	0.00976	0.114904	-2.54341
T284338	1.12612	4.923433	3.857377	0.009766	0.114935	-2.54407
NONHSAT218592.1	-1.38795	5.433568	-3.85702	0.00977	0.114941	-2.54449
lnc-BAG5-2:1	2.10642	2.717012	3.856194	0.009779	0.11503	-2.54546
NONHSAT200975.1	1.490985	2.657846	3.856087	0.00978	0.11503	-2.54558
NONHSAT204962.1	-2.08886	3.255487	-3.85542	0.009787	0.115053	-2.54637
NONHSAT175850.1	-1.33356	6.238602	-3.8553	0.009789	0.115053	-2.54651
ENST00000438923.1	1.71159	2.548777	3.854392	0.009798	0.11506	-2.54756
UVRAG-DT:22	1.499129	2.538401	3.854253	0.0098	0.11506	-2.54772
ENST00000670409.1	-1.59307	3.399853	-3.85261	0.009818	0.115087	-2.54965
NONHSAT162365.1	1.055334	5.167863	3.852376	0.00982	0.115087	-2.54992
lnc-BEND7-1:2	-1.26246	2.158958	-3.85223	0.009822	0.115087	-2.55008
MSTRG.49024.1	1.885863	2.16268	3.852129	0.009823	0.115087	-2.55021
NONHSAT198912.1	1.511208	2.149705	3.848414	0.009863	0.115343	-2.55455
LINC00518:4	1.271098	1.711345	3.848072	0.009867	0.115359	-2.55495
MSTRG.28690.1	-1.45589	3.643671	-3.84747	0.009873	0.115365	-2.55566
NONHSAT160987.1	1.460253	2.69205	3.84677	0.009881	0.115382	-2.55647
NONHSAT178308.1	1.182562	7.153385	3.845929	0.00989	0.11542	-2.55746
lnc-KCNJ12-7:1	-1.52072	2.204429	-3.8459	0.00989	0.11542	-2.55749
lnc-MPV17L2-1:8	-1.49842	2.421029	-3.84572	0.009892	0.11543	-2.5577
MSTRG.42800.1	-1.58216	2.201864	-3.84545	0.009895	0.11545	-2.55801
NONHSAT222338.1	1.229052	2.692401	3.844911	0.009901	0.115453	-2.55865
lnc-ADAMTS5-5:1	1.291414	3.191295	3.844356	0.009907	0.115453	-2.5593
lnc-DBT-6:1	-1.03187	5.126668	-3.84428	0.009908	0.115453	-2.55938
MSTRG.39196.1	1.567131	2.397094	3.844085	0.00991	0.115453	-2.55961
ENST00000657178.1	1.204832	1.727375	3.841996	0.009933	0.115582	-2.56206
NONHSAT158154.1	-1.89046	2.999011	-3.84022	0.009952	0.115743	-2.56414
lnc-C8A-7:1	-1.0542	3.144263	-3.83943	0.009961	0.115799	-2.56507

lnc-ZNF627-2:15	-1.24991	4.181356	-3.83934	0.009962	0.115799	-2.56517
lnc-LDB1-1:1	-1.0279	1.674757	-3.83915	0.009964	0.1158	-2.56539
lnc-AKR1C2-3:8	-1.15458	3.890085	-3.83784	0.009978	0.115859	-2.56693
ENST00000607434.1	-1.52983	2.739818	-3.83749	0.009982	0.115859	-2.56734
lnc-GGPS1-17:1	-1.20399	2.22272	-3.83666	0.009991	0.115866	-2.56831
lnc-BMP6-106:140	1.266472	1.6525	3.836512	0.009993	0.115866	-2.56848
NONHSAT200585.1	1.536081	3.50507	3.836507	0.009993	0.115866	-2.56849
NONHSAT177853.1	1.174145	3.881665	3.835051	0.010009	0.115939	-2.57019
NONHSAT192503.1	1.144155	5.216529	3.83475	0.010012	0.115939	-2.57055
MSTRG.4610.1	1.391137	2.529659	3.834539	0.010014	0.115939	-2.57079
MSTRG.51461.2	1.120789	5.86919	3.8343	0.010017	0.115939	-2.57107
NONHSAT189584.1	1.160349	4.43456	3.833635	0.010024	0.115939	-2.57185
NONHSAT202837.1	1.167694	3.567003	3.831733	0.010045	0.116089	-2.57408
lnc-KMT2C-6:3	1.427787	5.447492	3.830572	0.010058	0.116103	-2.57544
NONHSAT153523.1	-1.42337	2.082062	-3.83048	0.010059	0.116103	-2.57556
lnc-ADCYAP1-10:1	1.178138	3.617882	3.830382	0.01006	0.116103	-2.57567
lnc-CDKN3-5:1	-1.0411	2.083962	-3.83035	0.010061	0.116103	-2.5757
MSTRG.44815.1	-1.18589	3.684143	-3.83031	0.010061	0.116103	-2.57575
MSTRG.23322.1	1.182935	4.596596	3.829588	0.010069	0.116131	-2.5766
T307750	1.699051	2.925141	3.829235	0.010073	0.116131	-2.57701
lnc-DNM1L-4:1	-1.16225	4.051164	-3.82916	0.010074	0.116131	-2.5771
MSTRG.30757.1	-1.84084	4.06331	-3.82761	0.010091	0.116241	-2.57892
NONHSAT210575.1	1.61731	2.603045	3.827527	0.010092	0.116241	-2.57901
MSTRG.7771.1	2.01954	2.674019	3.827231	0.010095	0.116266	-2.57936
lnc-ACADSB-1:1	1.125354	2.15777	3.827106	0.010097	0.116268	-2.57951
lnc-ZNF284-2:1	1.004221	4.506765	3.826874	0.010099	0.116275	-2.57978
MSTRG.49548.1	-1.11126	4.283781	-3.82674	0.010101	0.116275	-2.57994
ENST00000552413.1	1.81209	2.557665	3.82659	0.010102	0.116281	-2.58011
NONHSAT209304.1	1.10167	1.633954	3.826032	0.010108	0.116313	-2.58077
MSTRG.58753.1	-1.23658	2.033876	-3.82384	0.010133	0.116461	-2.58334
lnc-CALM3-2:1	-1.02953	2.187816	-3.82339	0.010138	0.116492	-2.58387
MSTRG.36192.1	1.205349	6.456327	3.823305	0.010139	0.116492	-2.58397
ENST00000523110.1	1.30497	3.175166	3.821677	0.010157	0.116619	-2.58588
NONHSAT195523.1	1.971808	2.074783	3.821345	0.010161	0.11664	-2.58627
ENST00000499452.2	-1.15609	3.995433	-3.82093	0.010165	0.116648	-2.58675
NONHSAT224041.1	1.348224	2.023047	3.820588	0.010169	0.116653	-2.58716
NONHSAT172098.1	1.533187	3.484916	3.818952	0.010188	0.116757	-2.58908
NONHSAT193401.1	-1.89222	2.573967	-3.81871	0.01019	0.116762	-2.58936
ENST00000429074.1	-1.18365	3.905147	-3.81807	0.010198	0.11683	-2.59011
NONHSAT164545.1	1.007917	1.940907	3.81775	0.010201	0.116851	-2.59049
NONHSAT217964.1	-1.36549	3.012417	-3.8177	0.010202	0.116851	-2.59055
NONHSAT206809.1	-2.56518	3.529879	-3.81703	0.010209	0.116872	-2.59134
lnc-PAXIP1-3:4	-1.46114	4.022328	-3.81614	0.010219	0.116959	-2.59238
ENST00000603191.2	-1.04944	5.775946	-3.81385	0.010245	0.117147	-2.59507
MSTRG.28504.1	1.74256	2.819494	3.812461	0.010261	0.117196	-2.5967
NONHSAT197325.1	1.471333	2.209405	3.811734	0.010269	0.11725	-2.59756
MSTRG.69732.1	-1.7115	3.331916	-3.81142	0.010273	0.117264	-2.59793
ENST00000652367.1	1.333541	2.875964	3.811292	0.010274	0.117267	-2.59808

NR_038334	-1.30861	3.38108	-3.81005	0.010288	0.117352	-2.59954
MSTRG.12030.1	-1.22584	3.345411	-3.80957	0.010294	0.117384	-2.6001
ENST00000433071.2	-1.0845	5.360235	-3.80848	0.010306	0.117485	-2.60139
MSTRG.58982.1	1.341528	1.941014	3.80823	0.010309	0.117504	-2.60168
lnc-CHD2-23:1	1.095676	5.245153	3.807672	0.010315	0.117546	-2.60233
NONHSAT153986.1	1.178836	3.358963	3.806647	0.010327	0.117603	-2.60354
lnc-KLRB1-1:3	1.851585	4.065588	3.806525	0.010328	0.117605	-2.60368
NONHSAT180873.1	1.630655	2.487227	3.806428	0.010329	0.117605	-2.6038
lnc-BBOF1-2:1	-1.37974	5.254366	-3.80507	0.010345	0.117657	-2.6054
lnc-DBT-7:1	-1.21265	3.166137	-3.80465	0.01035	0.117657	-2.60589
ENST00000603154.1	1.02152	3.376057	3.804635	0.01035	0.117657	-2.60591
NONHSAT222206.1	1.116569	1.653911	3.802761	0.010371	0.117803	-2.60811
lnc-BHLHA9-3:1	-1.24264	1.902974	-3.80232	0.010376	0.117834	-2.60864
MSTRG.56458.14	1.756445	2.523301	3.801152	0.01039	0.117946	-2.61001
lnc-TERB2-2:1	1.797094	2.321158	3.800826	0.010393	0.117975	-2.61039
lnc-TPD52L1-3:1	-1.11913	3.254849	-3.79944	0.010409	0.118078	-2.61202
NONHSAT214461.1	-1.04961	1.618612	-3.79922	0.010412	0.118078	-2.61228
lnc-THAP3-4:1	-1.32659	1.985913	-3.79865	0.010418	0.118101	-2.61295
SNHG1:33	-1.21313	5.657737	-3.7981	0.010425	0.118111	-2.6136
NONHSAT218904.1	1.766706	4.679776	3.798057	0.010425	0.118111	-2.61365
NONHSAT174554.1	-1.75895	2.690985	-3.79732	0.010434	0.118141	-2.61452
NONHSAT210587.1	-1.17469	2.322933	-3.79518	0.010458	0.1183	-2.61704
MSTRG.22787.1	1.073558	4.744765	3.794651	0.010465	0.118356	-2.61766
lnc-C8orf86-8:1	1.051143	6.245532	3.793034	0.010483	0.118489	-2.61957
lnc-TCTA-3:1	-1.38019	3.54482	-3.79246	0.01049	0.11851	-2.62024
lnc-TRMT9B-3:1	-1.21609	2.695138	-3.79182	0.010497	0.118528	-2.621
lnc-CROT-1:1	1.089616	3.032588	3.791637	0.010499	0.118528	-2.62121
LIF-AS1:10	1.458044	4.849593	3.791599	0.0105	0.118528	-2.62126
lnc-EFCAB11-3:1	-1.34424	3.230416	-3.79121	0.010504	0.118555	-2.62171
ENST00000594562.1	-1.16225	3.599989	-3.7907	0.01051	0.118582	-2.62232
MSTRG.20055.1	1.647906	2.302267	3.790573	0.010512	0.118583	-2.62247
MSTRG.39481.4	1.140753	4.193543	3.789699	0.010522	0.118583	-2.6235
ENST00000625090.1	1.317988	2.938899	3.789605	0.010523	0.118583	-2.62361
NONHSAT178422.1	1.45988	3.999823	3.789382	0.010526	0.118583	-2.62387
lnc-CLDN11-8:1	-1.45183	1.850214	-3.78895	0.010531	0.118623	-2.62438
NONHSAT186578.1	1.258517	6.457885	3.788131	0.01054	0.118654	-2.62535
NONHSAT157516.1	-1.01421	2.254446	-3.78776	0.010545	0.118689	-2.62579
MSTRG.53542.1	1.206671	3.307766	3.786254	0.010562	0.118799	-2.62756
NONHSAT175431.1	1.130224	3.593206	3.785939	0.010566	0.118823	-2.62793
ENST00000586389.1	-1.04023	5.654627	-3.7858	0.010567	0.118827	-2.62809
NONHSAT154908.1	1.320957	4.782253	3.785363	0.010573	0.118854	-2.62861
lnc-RAB30-6:1	-1.59674	2.746138	-3.78535	0.010573	0.118854	-2.62863
NONHSAT177175.1	1.019908	3.120193	3.785197	0.010575	0.118854	-2.62881
ENST00000653239.1	-1.1091	2.152136	-3.78467	0.010581	0.118897	-2.62943
MSTRG.43948.1	1.432315	3.303831	3.784199	0.010586	0.118897	-2.62999
lnc-HIST1H2BI-3:1	1.709812	2.77589	3.781966	0.010612	0.119036	-2.63262
ENST00000518591.1	-1.80366	3.386293	-3.78194	0.010613	0.119036	-2.63266
NONHSAT212913.1	1.513174	4.395609	3.781608	0.010617	0.119036	-2.63304

lnc-PLEKHA2-4:1	-1.27773	4.43981	-3.78153	0.010618	0.119036	-2.63313
NONHSAT219350.1	-1.65687	2.203309	-3.78087	0.010625	0.119079	-2.63391
NONHSAT195442.1	1.202145	3.15414	3.780581	0.010629	0.119079	-2.63426
LINC02449:15	1.115988	4.64218	3.780559	0.010629	0.119079	-2.63428
lnc-ING2-1:1	-1.43454	2.44521	-3.78048	0.01063	0.119079	-2.63438
ENST00000427132.1	-1.29153	2.329298	-3.78027	0.010632	0.11908	-2.63462
lnc-PRKDC-3:1	1.437399	2.335439	3.778746	0.01065	0.119228	-2.63642
lnc-ZNF500-1:3	1.150316	3.085468	3.778629	0.010652	0.11923	-2.63656
NONHSAT153734.1	1.032318	2.225285	3.777684	0.010663	0.1193	-2.63768
NONHSAT187909.1	-1.40612	1.986258	-3.7776	0.010664	0.1193	-2.63778
lnc-C9orf47-2:1	-1.12927	3.148153	-3.77702	0.010671	0.11935	-2.63846
NONHSAT194118.1	1.04849	2.071061	3.776704	0.010674	0.119365	-2.63883
NONHSAT172237.1	1.937128	2.570552	3.776483	0.010677	0.119378	-2.6391
NONHSAT186770.1	1.280869	1.965825	3.775733	0.010686	0.119438	-2.63998
lnc-FNTB-5:1	-2.06049	2.224538	-3.77533	0.010691	0.119455	-2.64046
ENST00000604491.1	-1.76468	2.320858	-3.77518	0.010692	0.119461	-2.64063
ENST00000616113.1	-1.28257	3.803429	-3.77362	0.010711	0.119615	-2.64248
MSTRG.40358.1	-1.03025	4.166963	-3.77272	0.010722	0.119696	-2.64354
lnc-EIF5-1:2	1.502545	2.514704	3.772625	0.010723	0.119696	-2.64365
NONHSAT218699.1	1.641284	2.736066	3.772575	0.010723	0.119696	-2.64371
NONHSAT162479.1	-1.06787	3.844036	-3.77114	0.010741	0.119746	-2.64541
T302404	-1.03842	2.146369	-3.77079	0.010745	0.119746	-2.64582
MSTRG.831.1	1.154745	3.925499	3.770333	0.01075	0.119746	-2.64636
ENST00000589323.1	2.123056	3.134453	3.770029	0.010754	0.119746	-2.64672
NONHSAT166978.1	1.744797	2.758512	3.769746	0.010757	0.119746	-2.64706
NONHSAT207417.1	1.298428	3.497344	3.769504	0.01076	0.119751	-2.64734
lnc-TMEM250-3:2	1.381526	2.139897	3.769235	0.010763	0.11976	-2.64766
NONHSAT206231.1	-1.38233	1.820366	-3.76726	0.010787	0.11993	-2.64999
lnc-KLHL35-4:2	-1.47812	2.446042	-3.76407	0.010825	0.120184	-2.65377
NONHSAT218591.1	-1.36962	1.844357	-3.76395	0.010827	0.120185	-2.65391
NONHSAT164779.1	1.738336	3.425916	3.763065	0.010837	0.120252	-2.65496
lnc-ELMOD1-1:1	1.412723	4.76067	3.762657	0.010842	0.120264	-2.65544
NONHSAT153502.1	-1.12497	1.973424	-3.76179	0.010853	0.120291	-2.65647
NONHSAT216719.1	1.60329	1.86661	3.761352	0.010858	0.120291	-2.65699
NONHSAT218169.1	1.533854	1.809493	3.761166	0.01086	0.120291	-2.65721
MSTRG.4494.1	1.421776	4.884821	3.760985	0.010862	0.120291	-2.65742
NONHSAT153894.1	1.307969	4.417941	3.760275	0.010871	0.120339	-2.65826
NONHSAT172223.1	-1.43694	2.200209	-3.76023	0.010871	0.120339	-2.65831
ENST00000657287.1	1.038556	4.88357	3.759387	0.010882	0.120426	-2.65931
NONHSAT159827.1	-1.59273	3.33894	-3.75926	0.010883	0.120426	-2.65947
NONHSAT152816.1	1.279167	4.247451	3.758545	0.010892	0.12048	-2.66031
ENST00000430776.2	1.835336	2.689065	3.758107	0.010897	0.120491	-2.66083
NONHSAT220255.1	1.783824	3.015251	3.756904	0.010912	0.120546	-2.66225
NONHSAT206235.1	1.196897	4.709728	3.756765	0.010913	0.120546	-2.66242
lnc-RALGAPA1-3:1	1.542112	1.881172	3.756527	0.010916	0.120558	-2.6627
lnc-CRYBA4-20:1	1.009317	4.239882	3.756085	0.010922	0.120572	-2.66322
NONHSAT177997.1	1.562045	2.902425	3.755711	0.010926	0.120588	-2.66367
MSTRG.3584.1	1.486154	2.22602	3.755027	0.010934	0.120638	-2.66448

lnc-EYA3-3:1	1.038221	4.31874	3.754946	0.010935	0.120638	-2.66457
NONHSAT198103.1	1.456652	2.230778	3.754284	0.010943	0.120684	-2.66536
MSTRG.69496.1	1.136919	1.659002	3.754127	0.010945	0.120684	-2.66554
NONHSAT173070.1	1.030169	2.76306	3.754084	0.010946	0.120684	-2.66559
MSTRG.53344.1	1.547637	3.539089	3.753473	0.010953	0.120711	-2.66632
lnc-PRR32-9:1	-1.60891	2.323708	-3.75335	0.010955	0.120711	-2.66646
lnc-RAB3B-1:1	-1.24424	2.186944	-3.75326	0.010956	0.120711	-2.66657
lnc-PNPLA3-1:11	1.45359	2.101639	3.753125	0.010958	0.120711	-2.66673
NONHSAT204047.1	1.942957	3.055838	3.753117	0.010958	0.120711	-2.66674
MSTRG.14112.1	-1.13789	1.767704	-3.75155	0.010977	0.120766	-2.6686
NONHSAT220467.1	1.248507	4.56741	3.751313	0.01098	0.120766	-2.66888
T046928	1.422454	4.948121	3.751109	0.010982	0.120766	-2.66912
lnc-EZR-2:1	1.565369	1.868131	3.750893	0.010985	0.120766	-2.66937
ENST00000660390.1	1.15332	2.128745	3.750792	0.010986	0.120766	-2.66949
NONHSAT223850.1	1.062556	7.280741	3.749883	0.010997	0.120766	-2.67057
NONHSAT161289.1	1.122128	5.423815	3.749815	0.010998	0.120766	-2.67065
NONHSAT201605.1	1.155262	1.790286	3.749577	0.011001	0.120776	-2.67094
MSTRG.44414.1	-1.09168	5.358508	-3.74932	0.011004	0.120776	-2.67124
lnc-KAT2B-8:1	-1.07129	2.1736	-3.74887	0.01101	0.120808	-2.67178
NONHSAT148263.1	1.174005	3.370703	3.747054	0.011032	0.120947	-2.67393
ENST00000454253.2	1.400472	9.376338	3.746148	0.011043	0.121016	-2.675
NONHSAT185309.1	-1.10331	1.728009	-3.74584	0.011047	0.121032	-2.67537
NONHSAT169982.1	1.766538	1.904222	3.745537	0.01105	0.121059	-2.67573
lnc-CRYM-6:1	1.407826	1.874861	3.744716	0.011061	0.121075	-2.6767
NONHSAT215948.1	1.316053	2.379033	3.744664	0.011061	0.121075	-2.67676
lnc-PRICKLE2-5:1	-1.15007	4.403709	-3.74462	0.011062	0.121075	-2.67682
NONHSAT193280.1	-1.51682	2.046455	-3.74445	0.011064	0.121075	-2.67702
NONHSAT205238.1	1.405395	2.726099	3.744349	0.011065	0.121075	-2.67713
NONHSAT152406.1	1.171741	1.932896	3.742739	0.011085	0.121226	-2.67904
MSTRG.52953.1	1.709671	2.623954	3.742535	0.011087	0.12124	-2.67929
lnc-TPT1-1:1	-1.24542	4.700309	-3.74089	0.011108	0.121396	-2.68124
ENST00000666763.1	1.512574	2.223393	3.740225	0.011116	0.121434	-2.68203
lnc-RAF1-1:1	-1.14773	2.843847	-3.73829	0.01114	0.121601	-2.68433
lnc-TOX3-3:1	-1.08302	1.729663	-3.73703	0.011156	0.121697	-2.68582
NONHSAT183293.1	-1.41294	2.243711	-3.73679	0.011159	0.121697	-2.68611
T157392	-1.02242	5.721889	-3.73675	0.011159	0.121697	-2.68615
NONHSAT189313.1	-1.6578	2.659322	-3.73671	0.01116	0.121697	-2.6862
NONHSAT189955.1	2.096375	2.691931	3.736002	0.011168	0.121756	-2.68704
MSTRG.3136.1	1.303839	1.820501	3.735743	0.011172	0.121771	-2.68735
NONHSAT154053.1	-1.52037	3.046062	-3.7357	0.011172	0.121771	-2.68741
ENST00000649401.1	1.427346	3.784698	3.735489	0.011175	0.121777	-2.68765
lnc-LAMA1-7:1	1.206021	1.803482	3.735174	0.011179	0.121777	-2.68803
MSTRG.10077.1	-1.25304	2.926126	-3.7335	0.0112	0.121882	-2.69001
lnc-OR4N4-1:6	1.242935	3.464541	3.732492	0.011212	0.12197	-2.69121
ENST00000584414.1	1.938888	4.024829	3.732324	0.011214	0.121976	-2.69141
NONHSAT163249.1	1.065841	4.020749	3.732059	0.011218	0.121976	-2.69173
lnc-GTSF1L-10:1	1.491467	1.793118	3.731356	0.011226	0.122026	-2.69256
NONHSAT157034.1	-1.37266	2.848638	-3.7313	0.011227	0.122026	-2.69263

MSTRG.17146.1	1.358199	2.096851	3.730354	0.011239	0.122118	-2.69375
NONHSAT221767.1	1.08155	5.296629	3.730045	0.011243	0.122134	-2.69412
NONHSAT194790.1	1.014523	1.560588	3.72935	0.011252	0.122199	-2.69495
lnc-SPNS2-4:1	2.034144	3.127172	3.729157	0.011254	0.1222	-2.69518
lnc-IRX5-4:1	1.503441	2.872675	3.727132	0.011279	0.122362	-2.69758
NONHSAT223804.1	-1.06846	5.037385	-3.72711	0.01128	0.122362	-2.69762
ENST00000659885.1	-1.14066	3.535703	-3.72649	0.011288	0.122379	-2.69835
NONHSAT148748.1	1.153413	2.56474	3.726487	0.011288	0.122379	-2.69835
T114088	-1.08842	4.672918	-3.7264	0.011289	0.122379	-2.69846
ENST00000582101.1	1.530813	3.929343	3.725128	0.011305	0.122455	-2.69997
NR_125936	-1.5549	1.888406	-3.72449	0.011313	0.122502	-2.70073
MSTRG.6619.1	-1.35569	2.836624	-3.7237	0.011323	0.122564	-2.70167
NONHSAT201110.1	1.03526	3.852792	3.723518	0.011325	0.122576	-2.70188
lnc-POFUT1-1:1	1.137948	4.350693	3.722832	0.011334	0.122604	-2.7027
lnc-NEK11-2:1	-1.53387	2.25538	-3.72262	0.011336	0.122604	-2.70296
NONHSAT168095.1	-1.14117	2.174904	-3.72195	0.011345	0.12266	-2.70375
MSTRG.58979.1	1.22818	3.91711	3.721675	0.011348	0.12267	-2.70408
lnc-PJVK-2:4	-1.28359	3.742485	-3.71997	0.01137	0.122838	-2.7061
NONHSAT211160.1	1.681956	3.207835	3.719855	0.011371	0.122841	-2.70624
MSTRG.24918.1	1.527492	1.878688	3.719098	0.011381	0.122862	-2.70714
NONHSAT218424.1	-1.18629	1.797596	-3.7188	0.011385	0.122862	-2.70749
ENST00000518090.1	-1.64135	2.93268	-3.71831	0.011391	0.122862	-2.70808
NONHSAT178754.1	1.258594	3.362144	3.718207	0.011392	0.122862	-2.7082
MSTRG.21637.2	1.589903	6.093479	3.718126	0.011393	0.122862	-2.7083
ENST00000655507.1	1.001776	1.523682	3.718036	0.011395	0.122862	-2.70841
NONHSAT162883.1	1.887171	2.37905	3.718027	0.011395	0.122862	-2.70842
MSTRG.18125.1	1.8287	2.608988	3.717862	0.011397	0.122862	-2.70862
MSTRG.9301.2	1.210537	4.080408	3.717769	0.011398	0.122862	-2.70873
lnc-GAPVD1-1:4	1.133221	3.719886	3.717381	0.011403	0.122865	-2.70919
NONHSAT174329.1	1.456681	2.355063	3.717124	0.011406	0.122874	-2.70949
lnc-EMC2-1:1	-1.39035	2.644747	-3.71592	0.011422	0.123014	-2.71093
ENST00000658663.1	1.414283	3.201044	3.715679	0.011425	0.12302	-2.71122
lnc-PLET1-7:1	1.803864	5.333607	3.715299	0.01143	0.12302	-2.71167
ENST00000415477.1	-1.34528	2.240614	-3.71516	0.011431	0.123025	-2.71183
NONHSAT151593.1	-1.22778	3.218562	-3.71421	0.011443	0.12313	-2.71296
NONHSAT152574.1	1.457665	1.947784	3.713269	0.011455	0.123154	-2.71409
NONHSAT167897.1	1.956247	3.617491	3.712493	0.011465	0.12321	-2.71501
NONHSAT214995.1	-1.1644	2.920016	-3.71245	0.011466	0.12321	-2.71506
NONHSAT177084.1	-1.27906	3.432756	-3.71221	0.011469	0.12321	-2.71534
lnc-CA13-1:1	-2.07301	2.858281	-3.7116	0.011477	0.123228	-2.71608
LINC02201:13	-1.07278	1.924126	-3.7112	0.011482	0.123261	-2.71655
NONHSAT155354.1	1.508505	2.120579	3.710987	0.011485	0.123271	-2.7168
NONHSAT211225.1	1.272602	2.374183	3.710808	0.011487	0.123271	-2.71702
NONHSAT163940.1	-1.14711	2.659119	-3.71019	0.011495	0.123314	-2.71775
MSTRG.54768.1	-1.5816	3.073121	-3.70944	0.011505	0.123314	-2.71865
NONHSAT167655.1	2.291094	3.237174	3.709196	0.011508	0.123314	-2.71894
lnc-ERICH1-9:3	-1.00343	5.064806	-3.70902	0.01151	0.123314	-2.71915
lnc-UBP1-4:1	-1.22959	3.79304	-3.70901	0.01151	0.123314	-2.71917

MSTRG.53180.1	-1.52117	2.479091	-3.70849	0.011517	0.123333	-2.71978
NR_026674	-1.66741	3.086697	-3.70765	0.011528	0.123336	-2.72079
lnc-FANK1-4:4	1.031226	3.991969	3.707566	0.011529	0.123336	-2.72088
MSTRG.41652.30	1.570356	3.677083	3.707412	0.011531	0.123336	-2.72107
T046081	-1.77467	2.97055	-3.70669	0.01154	0.123397	-2.72192
NONHSAT223325.1	1.076998	4.018869	3.706079	0.011548	0.123427	-2.72266
MSTRG.10616.5	1.29571	4.828178	3.706068	0.011548	0.123427	-2.72267
lnc-SERPINI1-1:1	-1.08748	2.301057	-3.70572	0.011553	0.123437	-2.72308
lnc-PAX9-7:1	1.21889	1.638776	3.70571	0.011553	0.123437	-2.7231
lnc-ENTHD1-1:2	1.044741	1.748437	3.705395	0.011557	0.123458	-2.72347
lnc-FZD10-4:1	1.856965	2.383396	3.70516	0.01156	0.123458	-2.72375
NONHSAT173237.1	1.004126	1.631781	3.705135	0.01156	0.123458	-2.72378
NONHSAT169248.1	1.374016	3.779051	3.705087	0.011561	0.123458	-2.72384
lnc-C9orf50-1:2	1.951927	3.039252	3.704166	0.011573	0.123572	-2.72494
NONHSAT205818.1	1.031396	6.615751	3.704018	0.011575	0.123579	-2.72511
ENST00000643151.1	1.614168	3.994661	3.70296	0.011588	0.123634	-2.72638
NONHSAT211354.1	1.41127	2.101717	3.702421	0.011595	0.12367	-2.72702
ENST00000507398.1	-1.4204	2.180785	-3.69996	0.011627	0.123882	-2.72996
NONHSAT183623.1	1.84513	6.77996	3.699942	0.011628	0.123882	-2.72998
NONHSAT176709.1	1.011918	1.551471	3.698392	0.011648	0.124032	-2.73183
NONHSAT216550.1	1.450451	3.375082	3.697898	0.011654	0.124052	-2.73242
lnc-NRROS-1:1	1.263591	3.815827	3.697727	0.011656	0.124052	-2.73262
NONHSAT194018.1	2.121415	2.142185	3.697686	0.011657	0.124052	-2.73267
ENST00000507770.1	-1.22673	3.650721	-3.69723	0.011663	0.124089	-2.73322
MSTRG.8945.1	2.592529	3.658763	3.695348	0.011688	0.124248	-2.73546
ENST00000529314.2	1.206431	3.628641	3.695333	0.011688	0.124248	-2.73548
lnc-PATE2-1:1	-1.41748	2.673467	-3.6948	0.011695	0.124255	-2.73612
NONHSAT209010.1	-1.03132	3.937941	-3.69472	0.011696	0.124255	-2.73621
NONHSAT205755.1	1.526202	2.127539	3.694711	0.011696	0.124255	-2.73622
NONHSAT164668.1	1.053634	3.040213	3.694452	0.011699	0.124255	-2.73653
lnc-SNCG-1:1	1.068917	5.894202	3.694133	0.011704	0.124271	-2.73691
lnc-BCLAF3-1:1	1.580288	3.079055	3.693673	0.01171	0.124296	-2.73746
lnc-MAGIX-1:2	-1.28324	2.816336	-3.69311	0.011717	0.124311	-2.73813
NONHSAT215339.1	1.179343	1.824158	3.691138	0.011743	0.12448	-2.74049
NONHSAT172294.1	1.371645	1.786125	3.691135	0.011743	0.12448	-2.74049
NONHSAT206059.1	1.238595	2.033881	3.690673	0.011749	0.124518	-2.74105
NONHSAT167612.1	1.412341	2.22669	3.690567	0.01175	0.12452	-2.74117
lnc-ACSM6-1:1	-1.88932	3.490165	-3.69003	0.011758	0.124561	-2.74182
lnc-APIP-3:1	1.528537	3.966174	3.689825	0.01176	0.124561	-2.74206
NONHSAT221794.1	1.66572	1.948248	3.689684	0.011762	0.124561	-2.74223
ENST00000661961.1	1.054687	8.181689	3.68938	0.011766	0.124561	-2.74259
NONHSAT167741.1	1.377242	3.183677	3.689087	0.01177	0.124561	-2.74294
lnc-LMBRD1-6:1	1.092886	3.444836	3.68903	0.011771	0.124561	-2.74301
NONHSAT151820.1	1.355968	4.581178	3.688512	0.011778	0.124598	-2.74363
ENST00000411978.1	1.715878	1.936899	3.688223	0.011781	0.124599	-2.74397
MSTRG.54566.35	-1.12206	1.660017	-3.68793	0.011785	0.124613	-2.74432
NONHSAT203342.1	1.07477	3.329612	3.686483	0.011804	0.124699	-2.74605
NONHSAT170093.1	2.731865	3.476717	3.686177	0.011808	0.124715	-2.74642

lnc-SCUBE3-2:1	-1.32998	2.00943	-3.68608	0.01181	0.124716	-2.74654
MSTRG.9325.1	1.693886	2.375554	3.684524	0.01183	0.124856	-2.7484
NONHSAT196309.1	-1.17654	5.280597	-3.68351	0.011844	0.124942	-2.74962
T074212	1.375315	2.190456	3.682106	0.011863	0.125027	-2.75129
ENST00000418598.1	-1.21293	3.5645	-3.68128	0.011874	0.125092	-2.75227
NONHSAT224516.1	-1.12803	3.154153	-3.68045	0.011885	0.125139	-2.75327
ENST00000657842.1	-1.50074	2.517411	-3.68036	0.011886	0.125139	-2.75338
NONHSAT211360.1	1.085221	4.816261	3.679476	0.011898	0.125238	-2.75444
lnc-TAT-6:1	1.633967	2.794539	3.677411	0.011925	0.125396	-2.75691
ENST00000566529.1	1.206206	2.178659	3.677327	0.011926	0.125396	-2.75701
lnc-ASB4-1:2	-1.65432	2.239495	-3.67646	0.011938	0.125421	-2.75805
lnc-TLE4-12:4	1.128807	3.151275	3.675394	0.011952	0.125486	-2.75932
NONHSAT221562.1	1.432393	2.239301	3.675197	0.011955	0.125488	-2.75956
lnc-ZNF219-1:1	2.235858	2.537212	3.674844	0.01196	0.125498	-2.75998
lnc-GK-8:1	1.457792	2.521821	3.674128	0.011969	0.125521	-2.76084
T272852	1.545612	2.205083	3.67373	0.011975	0.125544	-2.76132
MSTRG.45379.7	-1.74892	2.229646	-3.67369	0.011975	0.125544	-2.76137
MSTRG.45107.23	1.598424	3.348462	3.672316	0.011994	0.125594	-2.76301
ENST00000451230.1	-1.33451	1.82866	-3.67152	0.012004	0.125627	-2.76396
NONHSAT210258.1	-1.26651	1.747399	-3.67066	0.012016	0.125671	-2.76499
NONHSAT154716.1	1.674118	2.6572	3.669764	0.012028	0.125727	-2.76607
lnc-CDH9-7:1	1.793957	2.520238	3.66922	0.012036	0.125748	-2.76672
NONHSAT187821.1	-1.0102	1.87778	-3.66919	0.012036	0.125748	-2.76675
lnc-KRTAP5-7-1:2	-1.01424	6.067044	-3.66835	0.012047	0.125816	-2.76776
NONHSAT159221.1	1.122552	6.591473	3.667393	0.01206	0.125886	-2.76891
NONHSAT178573.1	-1.39969	2.788374	-3.66663	0.012071	0.125947	-2.76982
ENST00000417794.1	1.431662	1.935973	3.666597	0.012071	0.125947	-2.76986
NONHSAT210892.1	1.058733	3.82681	3.666443	0.012073	0.125956	-2.77005
PKIA-AS1:4	1.737235	2.498787	3.66595	0.01208	0.126	-2.77064
lnc-ZNF675-4:1	-2.18781	3.977852	-3.66556	0.012085	0.126028	-2.77111
NONHSAT207831.1	1.117816	7.588087	3.665472	0.012086	0.126028	-2.77121
MSTRG.51522.1	-1.20401	5.421498	-3.66516	0.012091	0.126033	-2.77158
lnc-FZR1-4:1	-1.32725	3.870364	-3.66482	0.012095	0.126042	-2.77199
lnc-PHF20L1-8:1	-1.24812	5.296508	-3.66442	0.012101	0.126073	-2.77247
ENST00000566143.2	-1.15946	3.035692	-3.66405	0.012106	0.126074	-2.77292
MSTRG.13610.1	1.316118	2.247235	3.663589	0.012112	0.126119	-2.77347
lnc-SH3BGR-5:1	1.598368	2.304079	3.662531	0.012127	0.126185	-2.77474
lnc-ACP4-1:2	2.188586	2.910339	3.662488	0.012127	0.126185	-2.77479
MSTRG.2262.27	1.035848	1.536765	3.662369	0.012129	0.126185	-2.77493
T152001	1.441749	1.783292	3.662307	0.01213	0.126185	-2.77501
NONHSAT204910.1	-1.08194	2.164768	-3.66117	0.012145	0.126209	-2.77637
lnc-RBM45-9:1	1.219816	1.908322	3.661067	0.012147	0.126211	-2.7765
lnc-NTSR2-6:1	1.116792	4.986455	3.660889	0.012149	0.126223	-2.77671
MSTRG.45376.1	1.369596	6.003358	3.660502	0.012154	0.126243	-2.77717
NONHSAT151481.1	-1.54263	3.223697	-3.65928	0.012171	0.126288	-2.77864
ENST00000651074.1	1.473951	5.890637	3.659223	0.012172	0.126288	-2.77871
NONHSAT195860.1	1.075046	1.999788	3.659074	0.012174	0.126288	-2.77889
NONHSAT214840.1	1.398747	3.574299	3.659031	0.012174	0.126288	-2.77894

ZBED3-AS1:53	1.446402	4.137789	3.658597	0.01218	0.126319	-2.77946
lnc-SSH1-2:1	1.25445	4.429854	3.658572	0.012181	0.126319	-2.77949
lnc-NT5C2-2:1	1.709095	2.364853	3.657591	0.012194	0.126406	-2.78067
lnc-NOVA1-3:1	-1.06248	2.083561	-3.65641	0.01221	0.126474	-2.78208
lnc-SMG6-8:1	1.547433	2.825855	3.653991	0.012244	0.126608	-2.78499
MSTRG.34744.1	-1.60472	2.927634	-3.65316	0.012255	0.126646	-2.78599
ENST00000605836.1	-1.1669	4.846786	-3.65309	0.012256	0.126646	-2.78608
lnc-WRNIP1-4:2	1.17284	1.6592	3.651479	0.012279	0.126723	-2.78801
lnc-AHR-1:12	-1.18991	2.04097	-3.65146	0.012279	0.126723	-2.78803
lnc-MPLKIP-8:1	2.133753	4.044595	3.650374	0.012294	0.126827	-2.78933
lnc-PAX5-7:1	-1.79708	4.112182	-3.64879	0.012316	0.126955	-2.79124
NONHSAT221898.1	-1.67388	3.782362	-3.64838	0.012322	0.126955	-2.79173
lnc-AMZ1-7:1	-1.45788	5.199788	-3.64836	0.012322	0.126955	-2.79176
NONHSAT205824.1	1.416376	4.651629	3.648019	0.012327	0.126955	-2.79216
NONHSAT176720.1	1.070504	1.566727	3.64797	0.012327	0.126955	-2.79222
lnc-SEMA5A-10:1	-1.11186	2.127171	-3.647	0.012341	0.127024	-2.79339
ENST00000514187.1	-1.19992	2.329013	-3.64658	0.012347	0.127071	-2.79389
lnc-RPP25L-1:2	-1.44186	3.591001	-3.64647	0.012348	0.127074	-2.79402
ENST00000657992.1	-1.19095	4.250785	-3.64606	0.012354	0.127081	-2.79452
NR_102268	1.199545	2.90057	3.645659	0.012359	0.127088	-2.795
ENST00000669000.1	-1.41767	4.001646	-3.64553	0.012361	0.127088	-2.79515
NONHSAT195901.1	-1.12832	1.779865	-3.64531	0.012364	0.127088	-2.79542
NONHSAT201252.1	-1.01838	4.020338	-3.64513	0.012367	0.127097	-2.79563
ENST00000538041.1	1.402543	1.874253	3.644668	0.012373	0.127099	-2.79619
lnc-ZC3H12D-13:1	1.171159	1.686669	3.644373	0.012377	0.127129	-2.79655
ENST00000606279.1	-1.25164	2.977508	-3.64274	0.0124	0.127261	-2.79851
T009860	1.74848	2.506322	3.641595	0.012416	0.127381	-2.79989
ENST00000563991.1	1.331946	3.370165	3.641341	0.01242	0.127392	-2.80019
lnc-TRIB1-9:1	1.661968	3.51277	3.640526	0.012431	0.127416	-2.80117
lnc-CYB561D2-2:1	-1.19502	2.533	-3.64052	0.012431	0.127416	-2.80118
T090882	-1.26767	2.229856	-3.6384	0.012461	0.127574	-2.80374
MSTRG.71226.1	2.153012	2.914192	3.637659	0.012472	0.127639	-2.80462
lnc-MLN-2:1	-1.33375	1.981303	-3.63711	0.012479	0.127642	-2.80528
NONHSAT160720.1	-1.31429	4.491453	-3.63618	0.012492	0.127712	-2.8064
ENST00000563289.1	1.646355	2.447669	3.63597	0.012495	0.127729	-2.80666
NONHSAT202484.1	1.628927	2.4647	3.635506	0.012502	0.127757	-2.80721
lnc-FAM25C-2:1	1.08851	4.876423	3.635193	0.012506	0.12779	-2.80759
NONHSAT158373.1	-1.09031	3.173392	-3.63471	0.012513	0.127808	-2.80818
lnc-WFDC9-1:1	-1.30392	3.520338	-3.6332	0.012535	0.127961	-2.80999
MSTRG.24499.1	-1.60315	2.802384	-3.63307	0.012536	0.127967	-2.81014
lnc-MBOAT1-2:26	1.910086	2.92013	3.632347	0.012547	0.128009	-2.81102
NONHSAT186514.1	-1.36511	3.165092	-3.63233	0.012547	0.128009	-2.81103
NONHSAT201175.1	1.611258	2.329471	3.631874	0.012553	0.128049	-2.81159
MSTRG.29324.1	1.37079	3.692147	3.630168	0.012578	0.128162	-2.81364
NONHSAT192352.1	1.077053	4.274083	3.630115	0.012578	0.128162	-2.81371
lnc-CYSLTR2-2:2	1.33776	3.833624	3.629712	0.012584	0.128192	-2.81419
lnc-PABPC4L-6:2	1.134504	4.492341	3.628728	0.012598	0.128235	-2.81538
ENST00000502284.1	-1.37734	4.651822	-3.62802	0.012608	0.128297	-2.81623

lnc-ARHGAP21-4:1	1.824657	2.316399	3.627515	0.012615	0.128332	-2.81684
NONHSAT158919.1	1.424776	2.760186	3.62611	0.012635	0.128438	-2.81853
NONHSAT206175.1	1.391388	2.022096	3.625211	0.012648	0.128463	-2.81962
MSTRG.45328.1	1.10319	5.078577	3.62506	0.012651	0.128463	-2.8198
MSTRG.67188.1	1.222344	5.3708	3.624847	0.012654	0.128474	-2.82006
lnc-OCA2-8:1	-1.04967	4.185995	-3.62425	0.012662	0.128521	-2.82077
lnc-PTPRR-3:2	-1.19665	5.101039	-3.62413	0.012664	0.128526	-2.82092
LINC01804:7	1.047825	2.709576	3.623851	0.012668	0.128554	-2.82126
MSTRG.8236.1	-1.16307	3.481213	-3.62341	0.012674	0.128558	-2.82178
NONHSAT164498.1	1.131895	3.756361	3.623375	0.012675	0.128558	-2.82183
NONHSAT188130.1	1.828563	3.599289	3.623175	0.012678	0.128575	-2.82207
lnc-RFC3-7:2	-1.59823	2.52607	-3.62233	0.01269	0.128627	-2.82309
NONHSAT206999.1	-1.39108	3.267164	-3.62193	0.012695	0.128627	-2.82357
NONHSAT164427.1	1.412925	2.160199	3.621304	0.012704	0.128665	-2.82433
ENST00000661767.1	-1.27728	3.699814	-3.62121	0.012706	0.128665	-2.82444
ENST00000420389.1	1.061853	2.453687	3.621071	0.012708	0.128665	-2.82461
NONHSAT219481.1	-1.0756	3.187599	-3.62093	0.01271	0.128665	-2.82478
lnc-HSPG2-3:1	-1.32238	4.180943	-3.62076	0.012712	0.128665	-2.82498
ENST00000575772.1	-1.11824	3.357442	-3.62067	0.012713	0.128665	-2.82509
ENST00000659449.1	-1.4993	3.177732	-3.62064	0.012714	0.128665	-2.82513
lnc-DPH3-3:1	-1.44795	2.638228	-3.62055	0.012715	0.128665	-2.82523
ENST00000450681.1	1.916013	2.668192	3.62018	0.012721	0.128675	-2.82568
ENST00000669105.1	-1.40113	3.447383	-3.61885	0.01274	0.128775	-2.82729
NONHSAT180279.1	-1.56594	2.837838	-3.6188	0.01274	0.128775	-2.82735
NONHSAT177657.1	1.473703	2.10023	3.617392	0.012761	0.128873	-2.82905
lnc-SPRTN-3:1	1.00847	3.907893	3.617159	0.012764	0.128873	-2.82933
lnc-THBS1-3:1	1.002048	5.759424	3.617142	0.012764	0.128873	-2.82935
MSTRG.55923.1	1.017477	7.274848	3.616278	0.012777	0.128883	-2.83039
ZNF337-AS1:17	-1.04564	3.181883	-3.61588	0.012783	0.128929	-2.83088
lnc-ELN-4:4	1.348641	2.306185	3.615498	0.012788	0.128955	-2.83133
ENST00000438738.1	1.523206	2.68214	3.615341	0.012791	0.128955	-2.83152
NONHSAT214867.1	1.337925	2.483221	3.615084	0.012794	0.128959	-2.83183
NONHSAT190400.1	1.398197	2.670164	3.61505	0.012795	0.128959	-2.83187
lnc-RACGAP1-1:13	-1.95988	3.738007	-3.61397	0.01281	0.129079	-2.83318
lnc-PDE9A-2:1	1.253509	1.844821	3.613348	0.012819	0.129106	-2.83393
NONHSAT178510.1	-1.16837	2.594526	-3.61284	0.012827	0.129129	-2.83455
NONHSAT224078.1	-1.49939	2.068936	-3.61271	0.012829	0.129133	-2.83469
lnc-ZCCHC17-7:2	-1.43516	3.142413	-3.61214	0.012837	0.129133	-2.83539
lnc-INTS8-3:1	-1.52748	2.760099	-3.61058	0.01286	0.129265	-2.83727
ENST00000661222.1	1.970759	3.237531	3.610122	0.012866	0.129294	-2.83782
NONHSAT167071.1	-1.13323	2.279615	-3.60988	0.01287	0.129295	-2.83811
NONHSAT153535.1	1.023055	5.77638	3.608031	0.012897	0.12942	-2.84035
NONHSAT206290.1	1.937439	3.018928	3.607913	0.012899	0.129425	-2.84049
NONHSAT150233.1	1.050993	3.054259	3.60589	0.012928	0.129653	-2.84294
NONHSAT167814.1	1.087407	1.595929	3.605834	0.012929	0.129653	-2.84301
lnc-NDST3-10:1	-1.27943	2.201377	-3.60538	0.012936	0.129694	-2.84355
MSTRG.21178.1	1.696509	2.608899	3.605095	0.01294	0.12971	-2.8439
NONHSAT186571.1	1.089243	4.139881	3.604062	0.012955	0.12985	-2.84515

lnc-ATXN7-12:1	-1.20612	5.173007	-3.60293	0.012972	0.129909	-2.84651
ENST00000657311.1	1.588074	2.320336	3.602239	0.012982	0.129964	-2.84735
lnc-RAB35-2:1	-1.07512	5.611366	-3.60152	0.012993	0.130026	-2.84822
ENST00000414377.1	-1.48771	4.572715	-3.60146	0.012993	0.130026	-2.84829
ENST00000426870.1	1.216932	1.668174	3.601327	0.012996	0.130034	-2.84845
NONHSAT158161.1	-1.36408	4.066079	-3.60115	0.012998	0.130034	-2.84867
NONHSAT194522.1	1.233025	3.896881	3.600862	0.013002	0.13006	-2.84902
ENST00000509191.5	1.002447	7.995928	3.600188	0.013012	0.130065	-2.84983
MSTRG.66471.4	-1.24473	4.468843	-3.59982	0.013018	0.130065	-2.85027
lnc-ITGA11-3:1	-1.53115	2.205999	-3.5998	0.013018	0.130065	-2.8503
lnc-PFKP-16:13	1.448722	2.636434	3.599635	0.01302	0.130077	-2.8505
NONHSAT214001.1	1.048269	3.625835	3.598512	0.013037	0.130179	-2.85186
NONHSAT202344.1	-1.03622	2.174006	-3.59829	0.01304	0.130199	-2.85213
lnc-NBAS-14:2	1.218059	5.466926	3.597807	0.013048	0.130227	-2.85271
ENST00000657583.1	-1.04962	5.160046	-3.59773	0.013049	0.130227	-2.8528
ENST00000502766.2	1.625546	1.838377	3.59749	0.013052	0.130227	-2.85309
NONHSAT186946.1	1.494677	2.587465	3.597381	0.013054	0.130227	-2.85323
lnc-PHTF2-4:2	1.2098	3.605044	3.5973	0.013055	0.130227	-2.85332
lnc-ERICH4-1:1	-2.15278	4.432514	-3.597	0.01306	0.130248	-2.85369
NONHSAT213858.1	1.947385	2.028409	3.596812	0.013062	0.130251	-2.85391
MSTRG.66393.12	-1.21898	2.719955	-3.59681	0.013062	0.130251	-2.85392
lnc-PTTG1-6:1	1.298986	3.81405	3.595741	0.013078	0.130357	-2.85521
ENST00000448412.2	-1.25401	2.515565	-3.59306	0.013118	0.130523	-2.85845
NONHSAT170606.1	-1.01417	4.695008	-3.59306	0.013118	0.130523	-2.85846
ENST00000646143.2	2.609741	5.62448	3.592725	0.013123	0.130546	-2.85886
lnc-RARS2-2:1	-1.01172	5.22259	-3.59211	0.013132	0.130599	-2.8596
MSTRG.55327.1	-1.91088	2.591451	-3.59174	0.013138	0.130611	-2.86005
MSTRG.42207.1	1.962053	3.210935	3.591518	0.013141	0.130611	-2.86032
NONHSAT164812.1	-1.41373	2.491956	-3.59151	0.013141	0.130611	-2.86033
MSTRG.40783.1	1.378863	6.335708	3.590331	0.013159	0.130744	-2.86176
lnc-TFAM-7:1	1.098046	2.992863	3.589633	0.013169	0.130761	-2.8626
lnc-PRDX1-2:1	-1.13678	3.916954	-3.58901	0.013179	0.130828	-2.86336
NR_026946	1.688637	3.108661	3.588886	0.013181	0.130834	-2.86351
NONHSAT185900.1	-1.02031	3.515265	-3.58768	0.013199	0.130948	-2.86497
lnc-BRF1-28:1	1.381873	2.178075	3.586992	0.013209	0.131013	-2.8658
NONHSAT156564.1	-1.1423	3.902163	-3.58638	0.013218	0.131087	-2.86655
ENST00000527443.1	1.106323	2.890626	3.586096	0.013222	0.131095	-2.86689
lnc-ZNF680-29:1	-1.52144	2.722668	-3.58591	0.013225	0.131097	-2.86712
ENST00000512036.1	1.544083	2.701671	3.585672	0.013229	0.131106	-2.8674
ENST00000653641.1	1.262451	1.781959	3.585482	0.013232	0.131111	-2.86763
lnc-ZDHHC17-11:1	1.284868	1.751503	3.585212	0.013236	0.131111	-2.86796
ENST00000526566.2	1.257468	5.303214	3.583292	0.013265	0.131243	-2.87029
ENST00000419944.1	2.265182	2.617422	3.582817	0.013272	0.131279	-2.87086
NONHSAT210717.1	1.657117	2.657849	3.582474	0.013277	0.131301	-2.87128
MSTRG.48102.1	1.366094	2.231766	3.581947	0.013285	0.131325	-2.87192
NONHSAT188466.1	1.518766	6.98453	3.581883	0.013286	0.131325	-2.872
NONHSAT215382.1	-1.95635	3.250955	-3.58103	0.013299	0.131348	-2.87303
T043258	1.154847	3.158741	3.580952	0.0133	0.131348	-2.87312

NR_125837	1.07534	3.967603	3.580705	0.013304	0.131359	-2.87342
MSTRG.44118.2	1.122739	2.574192	3.580459	0.013308	0.131383	-2.87372
T356962	1.281436	1.686663	3.58019	0.013312	0.131386	-2.87405
lnc-TM9SF2-6:1	-1.14482	2.476366	-3.57975	0.013318	0.131399	-2.87458
NONHSAT154528.1	-1.09341	2.508046	-3.57868	0.013335	0.131546	-2.87588
NONHSAT206423.1	1.204971	2.110953	3.578491	0.013337	0.131546	-2.87611
MSTRG.56189.1	1.11953	4.422009	3.578375	0.013339	0.131546	-2.87625
ENST00000577621.1	-1.24847	3.011789	-3.57833	0.01334	0.131546	-2.8763
ENST00000505575.1	1.425806	6.295781	3.577997	0.013345	0.131563	-2.87671
ENST00000609839.1	1.07938	3.076878	3.577799	0.013348	0.131563	-2.87695
NONHSAT163917.1	-1.57615	2.180561	-3.57768	0.01335	0.131568	-2.8771
NONHSAT214004.1	-1.56649	2.938803	-3.57745	0.013353	0.131576	-2.87737
NR_047476	1.467699	5.397888	3.577197	0.013357	0.131589	-2.87768
lnc-CREB1-9:1	1.359471	5.37578	3.576969	0.013361	0.131606	-2.87795
lnc-CLDND1-2:1	-1.26865	2.769059	-3.57669	0.013365	0.131626	-2.87829
NONHSAT198406.1	-1.42162	2.892934	-3.57632	0.01337	0.131657	-2.87874
lnc-TUBA3C-19:1	1.197067	6.612237	3.575957	0.013376	0.131668	-2.87918
lnc-JPH3-3:15	2.182016	2.620035	3.575258	0.013387	0.131712	-2.88003
MSTRG.889.1	1.25004	2.25748	3.575168	0.013388	0.131713	-2.88014
NONHSAT164756.1	1.573394	3.950629	3.57461	0.013397	0.131735	-2.88082
lnc-L3MBTL4-3:1	1.191018	7.794674	3.574517	0.013398	0.131735	-2.88093
LINC00960:14	-1.15676	2.891512	-3.57418	0.013403	0.131742	-2.88134
MSTRG.27797.5	1.767603	2.725038	3.573735	0.01341	0.131742	-2.88188
ENST00000655017.1	1.565983	2.967106	3.57286	0.013423	0.131814	-2.88294
NONHSAT167882.1	-1.06461	3.37543	-3.5682	0.013495	0.132171	-2.8886
MSTRG.45522.1	2.392088	5.709855	3.567751	0.013502	0.132187	-2.88914
NONHSAT180875.1	1.212447	3.801964	3.566627	0.013519	0.13226	-2.89051
ENST00000624818.1	-1.24236	2.229769	-3.56659	0.01352	0.13226	-2.89056
ENST00000504409.3	1.334171	2.732402	3.566154	0.013526	0.132312	-2.89108
MSTRG.72479.1	1.455738	3.610804	3.565484	0.013537	0.13233	-2.8919
NONHSAT215205.1	2.043182	2.806302	3.565271	0.01354	0.13233	-2.89216
ENST00000668117.1	1.466573	3.378481	3.564583	0.013551	0.132408	-2.89299
lnc-FBXL3-2:1	-1.33847	4.422572	-3.56336	0.01357	0.132479	-2.89448
lnc-ABCD3-9:1	-1.05711	4.699846	-3.56326	0.013571	0.13248	-2.8946
NONHSAT173142.1	-1.70371	2.607835	-3.56314	0.013573	0.132485	-2.89474
lnc-P4HA3-2:1	-1.60687	3.132644	-3.56183	0.013593	0.132579	-2.89633
lnc-HS3ST3B1-9:2	1.125091	3.550208	3.561759	0.013594	0.132579	-2.89642
MSTRG.840.1	1.433075	2.162894	3.561445	0.013599	0.132583	-2.89681
MSTRG.23099.1	1.479326	4.369088	3.56114	0.013604	0.132583	-2.89718
MSTRG.63581.1	1.403271	4.085917	3.560934	0.013607	0.132583	-2.89743
lnc-TMEM206-4:1	1.471337	2.31716	3.560822	0.013609	0.132583	-2.89756
MSTRG.10862.1	1.155687	1.855483	3.560706	0.013611	0.132583	-2.8977
NONHSAT164565.1	1.088411	1.571471	3.560631	0.013612	0.132583	-2.8978
NONHSAT165160.1	1.36721	1.728834	3.560487	0.013614	0.132592	-2.89797
NONHSAT181568.1	1.425806	5.101137	3.56023	0.013618	0.132605	-2.89828
NONHSAT199573.1	1.677145	2.8351	3.559073	0.013636	0.132649	-2.89969
ENST00000660929.1	-1.23962	1.953872	-3.559	0.013637	0.132649	-2.89978
NONHSAT218083.1	2.205438	3.003021	3.558751	0.013641	0.132663	-2.90008

NONHSAT212826.1	2.123582	2.427086	3.556712	0.013673	0.132844	-2.90256
lnc-DGKI-1:1	-1.15402	3.924897	-3.55585	0.013686	0.132909	-2.90361
ENST00000605407.1	-1.29273	3.693451	-3.55574	0.013688	0.132909	-2.90374
MSTRG.53254.1	1.104255	5.911422	3.555178	0.013697	0.132909	-2.90443
lnc-ZGRF1-2:1	1.025659	4.472536	3.554918	0.013701	0.132909	-2.90474
NONHSAT160040.1	1.056051	3.330532	3.554457	0.013708	0.132909	-2.9053
NONHSAT166303.1	-1.34181	2.420289	-3.55444	0.013709	0.132909	-2.90533
T214992	1.095973	3.502877	3.554238	0.013712	0.132913	-2.90557
ENST00000524008.1	-1.69942	2.797849	-3.55344	0.013724	0.13297	-2.90654
T298512	1.528788	2.790004	3.553205	0.013728	0.132993	-2.90683
NONHSAT163641.1	-1.25957	1.775159	-3.55268	0.013736	0.133009	-2.90747
NONHSAT217056.1	1.630978	2.964975	3.552133	0.013745	0.133017	-2.90813
NONHSAT220317.1	1.063854	3.48397	3.551701	0.013751	0.133056	-2.90866
lnc-CHRDL2-4:1	1.365653	5.474367	3.550776	0.013766	0.133104	-2.90978
MSTRG.62525.1	-1.25917	2.028254	-3.5504	0.013772	0.133104	-2.91024
MSTRG.31863.1	1.26778	3.901995	3.550224	0.013775	0.133104	-2.91046
ENST00000619560.1	1.909218	2.562603	3.550075	0.013777	0.133104	-2.91064
T188803	-1.22512	3.051801	-3.54886	0.013796	0.133224	-2.91211
NONHSAT182669.1	1.229575	1.669282	3.54885	0.013796	0.133224	-2.91213
MSTRG.58015.1	-1.13696	4.587728	-3.547	0.013826	0.133414	-2.91438
NONHSAT176684.1	1.168346	3.777385	3.546898	0.013827	0.133414	-2.9145
NONHSAT207120.1	1.00851	3.451182	3.546017	0.013841	0.133434	-2.91558
MSTRG.51670.1	1.619718	6.748017	3.545037	0.013857	0.133461	-2.91677
NONHSAT178083.1	1.057221	7.744767	3.544932	0.013858	0.133461	-2.9169
ENST00000661314.1	1.211814	3.976365	3.544881	0.013859	0.133461	-2.91696
MSTRG.63976.1	1.621099	1.824173	3.544214	0.01387	0.133496	-2.91777
NONHSAT210826.1	1.073671	1.569411	3.544181	0.01387	0.133496	-2.91781
NONHSAT165521.1	1.833943	2.873012	3.543976	0.013874	0.133496	-2.91806
ENST00000669468.1	1.006527	2.623544	3.543829	0.013876	0.133496	-2.91824
NONHSAT183085.1	1.260548	8.27591	3.54346	0.013882	0.133496	-2.91869
lnc-RTP5-3:2	2.087749	3.476837	3.543165	0.013886	0.133504	-2.91905
MSTRG.23692.1	1.565369	2.23983	3.542845	0.013892	0.133527	-2.91944
NONHSAT190043.1	1.276766	7.349768	3.542475	0.013897	0.133545	-2.91989
NONHSAT166762.1	1.235185	3.934592	3.54214	0.013903	0.133584	-2.9203
NONHSAT206443.1	1.117331	4.158834	3.540466	0.013929	0.133696	-2.92234
lnc-ME2-2:1	-1.57335	4.547277	-3.54046	0.013929	0.133696	-2.92235
lnc-UTS2-5:8	-1.09742	1.815802	-3.54041	0.01393	0.133696	-2.92241
MSTRG.28695.1	1.510586	1.836046	3.539009	0.013953	0.133812	-2.92412
lnc-LSM6-1:3	1.441117	3.657217	3.538881	0.013955	0.133812	-2.92427
MSTRG.47231.10	-1.0701	2.798045	-3.53718	0.013982	0.133962	-2.92635
NONHSAT170728.1	-1.20336	3.256743	-3.53642	0.013994	0.134031	-2.92728
lnc-ZNF680-12:1	1.240208	1.986331	3.536276	0.013996	0.134031	-2.92745
MSTRG.3694.15	1.87256	2.614197	3.536074	0.014	0.134031	-2.9277
MSTRG.8198.1	-1.13886	6.153969	-3.53566	0.014006	0.13408	-2.9282
lnc-HTR1D-1:1	-1.31939	2.820051	-3.53547	0.014009	0.134097	-2.92843
NONHSAT154567.1	1.455067	2.11568	3.5353	0.014012	0.13411	-2.92864
lnc-PDSS1-3:1	-1.05559	4.535601	-3.53393	0.014034	0.134244	-2.93031
MSTRG.10278.1	1.399156	3.825363	3.531939	0.014066	0.13433	-2.93274

ENST00000653445.1	1.891724	2.076375	3.531746	0.014069	0.13433	-2.93297
NONHSAT221550.1	-1.39146	1.928098	-3.53103	0.014081	0.134375	-2.93385
NONHSAT201189.1	1.205413	2.368945	3.530693	0.014086	0.134412	-2.93426
NONHSAT209089.1	-1.81479	3.091576	-3.53011	0.014096	0.134463	-2.93497
lnc-FGF8-1:5	1.421857	3.66828	3.527547	0.014137	0.134728	-2.9381
NONHSAT217040.1	1.85744	2.552075	3.527042	0.014145	0.134766	-2.93872
NONHSAT216332.1	1.593914	5.301463	3.526865	0.014148	0.134766	-2.93893
NONHSAT187426.1	1.08071	3.25767	3.525909	0.014164	0.134822	-2.9401
NONHSAT200358.1	-1.10187	3.61203	-3.52542	0.014172	0.134883	-2.9407
NONHSAT200253.1	-1.19604	2.051125	-3.52489	0.01418	0.134911	-2.94134
ENST00000662757.1	-1.53296	3.566377	-3.52484	0.014181	0.134911	-2.9414
NONHSAT187295.1	1.049119	6.370668	3.523956	0.014196	0.134995	-2.94248
lnc-ABCA5-11:1	1.705766	1.876436	3.523552	0.014202	0.135032	-2.94298
lnc-MAP3K8-5:2	1.450665	1.808316	3.52247	0.01422	0.135076	-2.9443
T055045	1.096702	1.566961	3.522446	0.01422	0.135076	-2.94433
lnc-DHX38-22:1	-1.02465	3.485479	-3.52212	0.014225	0.135082	-2.94473
ENST00000421041.1	1.043354	4.039745	3.52208	0.014226	0.135082	-2.94478
ENST00000412483.1	1.324305	4.04386	3.520804	0.014247	0.135158	-2.94634
NONHSAT160909.1	1.467644	1.849309	3.520643	0.01425	0.135158	-2.94653
lnc-VSIG10-2:1	-1.46279	4.348631	-3.51728	0.014305	0.135433	-2.95064
NONHSAT178883.1	1.280722	2.915956	3.516952	0.01431	0.135462	-2.95104
NONHSAT192124.1	-1.29366	4.079148	-3.51667	0.014315	0.135465	-2.95139
MSTRG.28898.1	1.893616	2.602883	3.51501	0.014342	0.135579	-2.95342
NONHSAT169398.1	1.348715	1.774697	3.514952	0.014343	0.135579	-2.95349
lnc-ST6GALNAC2-1:1	1.907254	2.048136	3.514239	0.014355	0.135641	-2.95436
NONHSAT211028.1	1.457536	2.655827	3.513806	0.014362	0.135669	-2.95489
NONHSAT186845.1	1.265916	2.959214	3.513173	0.014372	0.135704	-2.95566
NONHSAT215014.1	1.369481	2.415697	3.512075	0.014391	0.135824	-2.95701
T092428	-1.65955	2.95027	-3.51107	0.014407	0.13593	-2.95823
NONHSAT197294.1	-1.45476	1.987317	-3.51099	0.014409	0.135931	-2.95834
lnc-RASA1-14:1	-1.65765	3.402969	-3.51055	0.014416	0.135943	-2.95887
NONHSAT169840.1	1.648125	2.397221	3.509961	0.014426	0.135964	-2.95959
lnc-SLC1A3-1:1	-1.40729	4.336281	-3.5096	0.014432	0.135996	-2.96004
NONHSAT205867.1	1.517836	1.880102	3.508685	0.014447	0.13605	-2.96116
NONHSAT157898.1	1.207911	2.762878	3.508357	0.014452	0.136075	-2.96156
ENST00000625073.1	1.303023	1.74002	3.508239	0.014454	0.136075	-2.9617
lnc-PTDSS1-3:1	-1.10222	4.629808	-3.50812	0.014456	0.136075	-2.96185
NONHSAT194438.1	1.554568	3.111451	3.506421	0.014484	0.136159	-2.96393
ENST00000661962.1	1.35446	2.149219	3.505899	0.014493	0.136192	-2.96456
NONHSAT204611.1	1.744266	1.91393	3.505059	0.014507	0.136211	-2.96559
T002036	1.155435	2.124888	3.504343	0.014519	0.136235	-2.96647
LINC00879:4	-1.1604	1.918656	-3.50379	0.014528	0.13625	-2.96715
NONHSAT215297.1	1.038037	1.890687	3.503242	0.014538	0.13625	-2.96782
NONHSAT167152.1	1.85326	2.987989	3.503179	0.014539	0.13625	-2.96789
MSTRG.29578.1	1.103422	4.666099	3.502161	0.014556	0.136311	-2.96914
lnc-TFAM-5:1	-1.42621	4.282335	-3.5015	0.014567	0.136376	-2.96995
NR_110258	1.135962	1.786646	3.501418	0.014568	0.136377	-2.97005
lnc-PPA2-10:1	1.641925	2.558905	3.501079	0.014574	0.136384	-2.97047

MSTRG.61178.1	1.274746	1.727883	3.500409	0.014585	0.136427	-2.97129
lnc-ASPHD2-3:1	-1.01959	4.280891	-3.49935	0.014603	0.136512	-2.97259
T131984	1.006447	4.270102	3.499117	0.014607	0.136523	-2.97287
NONHSAT193814.1	-1.44455	2.519932	-3.49887	0.014611	0.136536	-2.97317
NONHSAT172405.1	1.058648	3.970251	3.498528	0.014617	0.136578	-2.97359
lnc-MARCH3-5:1	-1.41308	2.159238	-3.49796	0.014626	0.136629	-2.97429
MSTRG.49932.1	1.421963	5.421395	3.497748	0.01463	0.136642	-2.97455
NR_003954	1.727858	4.162906	3.497697	0.014631	0.136642	-2.97461
lnc-ARHGAP28-13:1	-1.07031	1.890775	-3.49728	0.014638	0.136672	-2.97512
MSTRG.64775.1	-1.20303	2.526641	-3.49696	0.014643	0.136698	-2.97552
MSTRG.3770.1	1.513386	2.006283	3.49634	0.014653	0.136743	-2.97627
ENST00000425820.1	-1.23316	2.995422	-3.49598	0.01466	0.136743	-2.97672
ENST00000554318.2	1.908491	3.269112	3.49596	0.01466	0.136743	-2.97674
NONHSAT222929.1	1.665571	3.294229	3.495951	0.01466	0.136743	-2.97675
lnc-ARRDC4-9:1	-1.54153	3.952253	-3.49545	0.014669	0.136771	-2.97737
NONHSAT153852.1	-1.52509	1.873592	-3.49457	0.014683	0.136797	-2.97845
MSTRG.29598.1	1.412683	3.461983	3.493469	0.014702	0.136888	-2.97979
lnc-UBAP1L-1:3	-1.10043	2.607621	-3.49344	0.014702	0.136888	-2.97982
lnc-C8orf48-7:1	-1.18049	2.690585	-3.49244	0.01472	0.137006	-2.98106
MSTRG.33394.1	-2.01674	3.376605	-3.49231	0.014722	0.137014	-2.98122
NONHSAT201058.1	-1.11138	2.834464	-3.49188	0.014729	0.137016	-2.98175
MSTRG.51859.1	-1.54148	3.712291	-3.49117	0.014741	0.137067	-2.98261
lnc-RAD23B-1:1	-1.06726	3.727689	-3.49077	0.014748	0.137088	-2.98311
ENST00000577281.2	-1.0802	2.141993	-3.49072	0.014749	0.137088	-2.98317
ENST00000668076.1	1.380653	4.33586	3.490512	0.014752	0.137095	-2.98342
ENST00000429530.1	-1.0056	4.001628	-3.48873	0.014783	0.1373	-2.98561
NONHSAT209420.1	1.809384	3.190295	3.488487	0.014787	0.137302	-2.9859
ENST00000446321.1	1.212814	2.805063	3.488329	0.014789	0.137314	-2.9861
ENST00000442815.1	1.55406	2.830666	3.487895	0.014797	0.137324	-2.98663
NONHSAT218189.1	-1.42403	3.1812	-3.48775	0.014799	0.137324	-2.98681
NR_038891	1.257211	2.842614	3.487458	0.014804	0.137324	-2.98717
ENST00000559246.1	1.08105	3.68078	3.487442	0.014805	0.137324	-2.98719
lnc-CLDN22-6:1	1.788862	2.468013	3.48743	0.014805	0.137324	-2.9872
NONHSAT180812.1	-1.49336	3.266781	-3.48739	0.014805	0.137324	-2.98725
lnc-ITGB8-4:1	1.6376	2.895488	3.486414	0.014822	0.137441	-2.98845
ENST00000650740.1	-1.3824	2.118628	-3.48601	0.014829	0.137457	-2.98894
ENST00000662516.1	1.146509	1.677874	3.485891	0.014831	0.13746	-2.98909
lnc-CDC42EP3-2:1	2.134812	3.106154	3.483364	0.014874	0.137774	-2.99219
NONHSAT214373.1	1.757641	2.402033	3.483277	0.014876	0.137774	-2.9923
NONHSAT164520.1	1.304677	5.422349	3.48256	0.014888	0.13784	-2.99318
ENST00000652062.1	-1.23309	2.542547	-3.48254	0.014889	0.13784	-2.99321
lnc-CTBP1-1:2	1.023186	4.55262	3.482173	0.014895	0.13784	-2.99365
ENST00000430184.1	1.422287	2.531103	3.481505	0.014906	0.137851	-2.99447
T343678	-2.00222	3.906252	-3.47976	0.014936	0.138043	-2.99662
NONHSAT204220.1	1.049541	3.982608	3.479324	0.014944	0.138062	-2.99715
lnc-CSF1-3:1	1.356373	1.777742	3.479283	0.014945	0.138062	-2.9972
lnc-ZNF518B-4:1	-1.35543	2.323241	-3.4788	0.014953	0.138095	-2.9978
ENST00000457706.1	1.110749	2.249155	3.478562	0.014957	0.138108	-2.99809

lnc-IQCH-7:1	-1.27614	3.36994	-3.47806	0.014966	0.138136	-2.99871
MSTRG.15910.2	-1.26814	3.283809	-3.47696	0.014985	0.138202	-3.00006
lnc-DHX38-29:3	-1.40715	2.405418	-3.47694	0.014985	0.138202	-3.00008
NONHSAT176395.1	1.902161	2.655672	3.476579	0.014991	0.138222	-3.00052
NONHSAT153668.1	1.579954	2.762992	3.475333	0.015013	0.138336	-3.00206
T309777	1.689418	3.547772	3.475242	0.015015	0.138336	-3.00217
lnc-SORBS2-3:1	-1.48786	2.871606	-3.47489	0.015021	0.138351	-3.00261
lnc-BMPER-4:1	1.260095	2.493513	3.474829	0.015022	0.138351	-3.00268
NONHSAT149237.1	-1.39645	3.694906	-3.47395	0.015037	0.138357	-3.00376
ENST00000426213.1	-1.69065	2.200474	-3.47357	0.015044	0.138398	-3.00422
MSTRG.64101.1	-1.65887	2.191532	-3.47294	0.015054	0.138437	-3.00499
lnc-MARCKS-19:1	1.27681	2.235311	3.472525	0.015062	0.138473	-3.00551
MSTRG.31192.1	-1.71497	3.026195	-3.47203	0.01507	0.138494	-3.00611
ENST00000647202.1	-1.16	4.589676	-3.47143	0.015081	0.138519	-3.00686
ENST00000419889.1	-1.8518	3.003526	-3.47109	0.015087	0.138544	-3.00728
NONHSAT150112.1	1.129971	3.198486	3.470616	0.015095	0.138594	-3.00785
lnc-EPSTI1-4:2	-1.13169	1.732936	-3.47008	0.015104	0.138655	-3.00851
lnc-RPN2-3:1	-1.86284	2.555335	-3.46937	0.015117	0.138716	-3.00939
T056970	1.470565	2.160523	3.468396	0.015134	0.138758	-3.01058
lnc-FABP2-3:1	-1.09534	4.036935	-3.46718	0.015155	0.138851	-3.01208
lnc-DDC-2:1	-1.23255	4.606119	-3.46647	0.015168	0.13891	-3.01296
NONHSAT181678.1	1.076839	2.744044	3.465456	0.015185	0.139036	-3.0142
NONHSAT193912.1	1.007924	6.837516	3.464528	0.015202	0.139095	-3.01534
lnc-RREB1-7:1	1.118313	2.219024	3.464513	0.015202	0.139095	-3.01536
NONHSAT174474.1	1.023478	6.065038	3.463926	0.015212	0.139109	-3.01608
lnc-TCEANC-3:1	-1.2613	4.453534	-3.46359	0.015218	0.139109	-3.0165
lnc-DYDC1-1:6	-1.08574	4.132296	-3.46356	0.015219	0.139109	-3.01654
MSTRG.32658.1	1.461191	1.788906	3.463517	0.015219	0.139109	-3.01659
ENST00000549163.1	1.622465	2.543053	3.46271	0.015234	0.1392	-3.01758
NONHSAT197936.1	1.163072	3.655029	3.46203	0.015246	0.139207	-3.01842
NONHSAT157750.1	-1.00715	1.710009	-3.46156	0.015254	0.139242	-3.019
lnc-PRR20C-5:2	1.335817	5.436316	3.460955	0.015265	0.139252	-3.01974
lnc-NKAIN3-12:1	-1.12803	1.656837	-3.46095	0.015265	0.139252	-3.01975
NONHSAT167757.1	1.577299	4.203021	3.460242	0.015277	0.139281	-3.02062
lnc-CYB5R1-1:1	-1.15637	3.274271	-3.45875	0.015304	0.139355	-3.02245
lnc-ENOPH1-2:1	-1.66213	3.455985	-3.45851	0.015308	0.139381	-3.02275
BACH1-IT2:5	-1.22339	1.922399	-3.45835	0.015311	0.139395	-3.02295
T072173	1.256791	1.869474	3.457139	0.015332	0.139565	-3.02444
MSTRG.48741.1	-1.39214	2.367905	-3.45547	0.015362	0.139671	-3.02649
MSTRG.10790.1	-1.34547	4.635334	-3.45525	0.015366	0.139681	-3.02676
lnc-ZFP1-1:2	-1.13724	1.656865	-3.45487	0.015373	0.139681	-3.02724
NONHSAT173077.1	1.474598	2.319573	3.454612	0.015377	0.139681	-3.02755
lnc-KY-1:1	1.324832	3.392573	3.453903	0.01539	0.139729	-3.02843
NONHSAT182771.1	1.14983	3.842688	3.452871	0.015408	0.139807	-3.0297
MSTRG.8952.1	1.247768	1.731661	3.452638	0.015412	0.139807	-3.02999
NONHSAT186995.1	2.155232	3.20754	3.452281	0.015419	0.139807	-3.03043
NONHSAT167034.1	1.121886	3.137509	3.452215	0.01542	0.139807	-3.03051
lnc-TRMT61B-5:5	-1.4997	3.22933	-3.45122	0.015438	0.139912	-3.03173

NONHSAT206179.1	1.048679	3.744786	3.450986	0.015442	0.139912	-3.03202
NONHSAT219029.1	1.160806	3.292937	3.450444	0.015452	0.139924	-3.03269
lnc-NDUFAF4-2:1	-1.09124	2.90348	-3.45041	0.015452	0.139924	-3.03273
MSTRG.57351.11	1.260941	2.396924	3.45012	0.015457	0.139934	-3.03309
lnc-SOBP-3:3	-1.05679	4.218771	-3.44998	0.01546	0.139945	-3.03327
ENST00000652905.1	1.191497	2.424922	3.448928	0.015479	0.14006	-3.03456
NONHSAT207628.1	-1.09588	4.62186	-3.44888	0.01548	0.14006	-3.03462
NONHSAT177958.1	1.775607	1.958267	3.44801	0.015495	0.140067	-3.03569
NR_038292	1.681221	3.134919	3.447883	0.015498	0.140067	-3.03585
lnc-SERPINE3-1:1	-1.36971	2.291116	-3.44734	0.015507	0.140121	-3.03652
NONHSAT197821.1	1.113383	4.666449	3.446842	0.015516	0.140148	-3.03713
NONHSAT224130.1	1.358178	2.864265	3.446455	0.015523	0.140165	-3.03761
ENST00000649539.1	1.014573	4.888019	3.445308	0.015544	0.140223	-3.03902
NONHSAT194244.1	1.337034	2.151283	3.444872	0.015552	0.140223	-3.03956
lnc-ACTA1-3:1	-1.68009	3.429857	-3.44466	0.015556	0.140223	-3.03983
NONHSAT167679.1	1.059401	2.721158	3.444476	0.015559	0.140223	-3.04005
NR_046090	1.320776	1.987374	3.444245	0.015563	0.140226	-3.04034
NONHSAT201133.1	1.676999	3.241314	3.444231	0.015563	0.140226	-3.04035
NONHSAT167079.1	1.33385	5.323282	3.442818	0.015589	0.140329	-3.0421
NONHSAT223586.1	-1.2188	3.1836	-3.44081	0.015625	0.14052	-3.04457
lnc-ULK4-3:2	1.017412	4.252593	3.440764	0.015626	0.14052	-3.04463
lnc-MRPL39-34:1	2.004264	3.209331	3.440763	0.015626	0.14052	-3.04463
NONHSAT172226.1	-1.38798	2.366298	-3.43771	0.015682	0.1408	-3.0484
NONHSAT211408.1	1.136246	1.706775	3.437131	0.015692	0.14082	-3.04912
NONHSAT182916.1	1.207864	3.827772	3.435292	0.015726	0.14102	-3.05139
MSTRG.14269.1	-1.0291	3.599281	-3.43506	0.01573	0.141045	-3.05167
NR_104607	-1.16227	2.970074	-3.43423	0.015745	0.141107	-3.0527
lnc-CCNB1IP1-4:4	-1.28248	3.941327	-3.4339	0.015751	0.141136	-3.05311
lnc-ANKH-5:1	-1.03253	4.105505	-3.43311	0.015766	0.141215	-3.05409
ENST00000569574.1	1.35879	2.694001	3.431896	0.015788	0.141383	-3.05558
NONHSAT182989.1	1.267786	2.100943	3.431397	0.015797	0.141433	-3.0562
lnc-ZNF781-2:2	1.318971	1.942809	3.431247	0.0158	0.141434	-3.05638
lnc-SLC10A6-3:1	1.216469	2.42836	3.430907	0.015806	0.141476	-3.0568
lnc-GPATCH1-1:1	1.039803	1.781638	3.430779	0.015808	0.141485	-3.05696
NONHSAT173346.1	-1.35297	4.175279	-3.43054	0.015813	0.141495	-3.05725
ENST00000623885.1	-1.03272	2.921368	-3.43041	0.015815	0.141495	-3.05742
NONHSAT155321.1	-1.15225	1.723121	-3.42999	0.015823	0.141531	-3.05794
lnc-PRLH-4:1	1.493859	3.695387	3.4299	0.015824	0.141531	-3.05805
NONHSAT195907.1	1.050134	1.631467	3.429503	0.015832	0.141569	-3.05854
NONHSAT186605.1	1.03619	3.769423	3.428709	0.015846	0.141626	-3.05952
NONHSAT188143.1	1.444609	2.219017	3.42835	0.015853	0.141626	-3.05997
NR_033856	1.649909	7.258119	3.428301	0.015854	0.141626	-3.06003
NONHSAT158167.1	-1.45122	3.055423	-3.42779	0.015863	0.141638	-3.06066
T342702	1.059963	6.51665	3.427605	0.015867	0.141653	-3.06089
lnc-ZNF37A-15:1	1.050592	8.295652	3.427485	0.015869	0.141653	-3.06103
lnc-FGF9-15:2	1.082808	2.927911	3.426162	0.015893	0.141818	-3.06267
MSTRG.47230.1	1.200045	4.612568	3.42454	0.015923	0.142023	-3.06468
NONHSAT157831.1	1.076794	4.426828	3.423885	0.015936	0.142059	-3.06549

MSTRG.23219.10	-1.23922	4.072177	-3.42388	0.015936	0.142059	-3.06549
NONHSAT207750.1	1.114175	1.644397	3.423864	0.015936	0.142059	-3.06551
ENST00000458171.1	-1.01507	4.760405	-3.42328	0.015947	0.14207	-3.06624
NONHSAT187419.1	1.5631	2.75344	3.422626	0.015959	0.142108	-3.06704
MSTRG.52996.1	1.144495	4.662939	3.422601	0.015959	0.142108	-3.06707
NONHSAT200032.1	-1.31031	2.119059	-3.42192	0.015972	0.142141	-3.06792
lnc-MIXL1-5:5	1.054811	2.91722	3.421641	0.015977	0.142141	-3.06826
lnc-CPZ-1:1	1.30813	4.10593	3.421564	0.015979	0.142141	-3.06836
NONHSAT220634.1	-1.02023	5.359969	-3.41993	0.016009	0.142347	-3.07038
MSTRG.3224.1	1.112867	5.50253	3.419686	0.016014	0.142359	-3.07068
MSTRG.40078.1	1.332561	2.105813	3.41968	0.016014	0.142359	-3.07069
NONHSAT214492.1	1.760374	2.836447	3.419499	0.016017	0.142365	-3.07091
NONHSAT214325.1	1.054234	2.618848	3.419438	0.016018	0.142365	-3.07099
ENST00000607957.1	-1.01405	3.192625	-3.41873	0.016031	0.142432	-3.07186
lnc-SETBP1-1:1	1.318702	5.518975	3.418234	0.016041	0.142456	-3.07248
ENST00000454040.5	1.445384	2.613314	3.418051	0.016044	0.142456	-3.0727
NONHSAT210343.1	1.405729	2.015907	3.417985	0.016045	0.142456	-3.07279
NONHSAT203565.1	1.28305	7.282543	3.417878	0.016047	0.142461	-3.07292
MSTRG.50035.2	-1.09776	3.247214	-3.41779	0.016049	0.142463	-3.07303
ENST00000630728.1	-1.43937	2.543203	-3.41719	0.01606	0.1425	-3.07377
NONHSAT197588.1	-1.26841	2.441639	-3.41672	0.016069	0.142553	-3.07436
MSTRG.7291.4	-1.44581	3.473892	-3.41625	0.016078	0.142561	-3.07493
lnc-XPNPEP1-3:1	-1.3794	2.440542	-3.41618	0.016079	0.142561	-3.07501
lnc-NDRG2-7:1	-1.10136	2.311232	-3.41608	0.016081	0.142561	-3.07515
NONHSAT187121.1	1.248381	1.924951	3.415779	0.016087	0.142561	-3.07552
NONHSAT186763.1	1.415235	10.17569	3.415627	0.016089	0.142561	-3.0757
NONHSAT162886.1	2.054562	3.455644	3.415613	0.01609	0.142561	-3.07572
NONHSAT178226.1	-1.02381	2.526611	-3.41506	0.0161	0.142602	-3.0764
NR_003714	-1.43673	3.099477	-3.4141	0.016118	0.142735	-3.07759
LINC01491:8	1.647047	2.920097	3.412846	0.016142	0.142833	-3.07915
MSTRG.71691.1	-1.00339	3.976401	-3.41215	0.016155	0.142868	-3.08001
NONHSAT222151.1	1.256182	2.051921	3.411432	0.016168	0.142868	-3.0809
NONHSAT170306.1	1.354963	3.454168	3.411239	0.016172	0.142887	-3.08114
NONHSAT181244.1	1.739123	2.962039	3.410681	0.016182	0.142931	-3.08183
SLC25A25-AS1:23	-1.48026	2.890947	-3.41057	0.016185	0.142931	-3.08197
ENST00000488190.1	1.60151	2.273041	3.410566	0.016185	0.142931	-3.08197
NONHSAT187827.1	1.506949	2.488789	3.409939	0.016196	0.142956	-3.08275
lnc-DLGAP2-8:2	-1.30481	3.633414	-3.40916	0.016211	0.14302	-3.08371
NONHSAT216979.1	-1.26926	2.801189	-3.40904	0.016213	0.143028	-3.08386
lnc-IRF4-20:9	1.406648	2.085645	3.408498	0.016224	0.143097	-3.08453
NONHSAT190327.1	1.727186	1.970579	3.408478	0.016224	0.143097	-3.08456
lnc-TENM4-3:3	1.160073	1.757926	3.408182	0.01623	0.143109	-3.08493
NONHSAT162377.1	1.489203	3.564402	3.405393	0.016282	0.143374	-3.08838
NONHSAT218173.1	1.025343	2.002318	3.405302	0.016284	0.143377	-3.0885
lnc-HIVEP2-6:1	-1.47745	3.700858	-3.40479	0.016294	0.143425	-3.08913
NONHSAT161534.1	1.118642	4.071834	3.404457	0.0163	0.143454	-3.08954
NONHSAT181265.1	1.032583	3.745225	3.404234	0.016305	0.143468	-3.08982
NONHSAT169882.1	1.692449	1.936416	3.404121	0.016307	0.143474	-3.08996

MSTRG.38905.1	1.302455	2.664578	3.40393	0.01631	0.143478	-3.0902
ENST00000451816.2	1.168571	4.672771	3.403752	0.016314	0.143478	-3.09042
NONHSAT166981.1	-1.20497	2.069312	-3.40373	0.016314	0.143478	-3.09045
T203095	-1.5246	2.280447	-3.40277	0.016332	0.143588	-3.09164
MSTRG.29915.15	1.047782	2.19408	3.402262	0.016342	0.143623	-3.09227
ENST00000660165.1	-1.59597	2.898619	-3.40142	0.016358	0.14372	-3.09331
lnc-FAM110B-12:1	-1.34162	2.8247	-3.40089	0.016368	0.143753	-3.09397
ENST00000654182.1	-1.40616	3.659167	-3.40017	0.016382	0.143781	-3.09486
NONHSAT187494.1	1.254052	5.582188	3.399314	0.016398	0.143867	-3.09592
lnc-KATNAL2-12:1	-1.01395	3.480741	-3.399	0.016404	0.143895	-3.09631
lnc-MTTP-1:1	-1.02804	1.609466	-3.3985	0.016414	0.143934	-3.09693
NONHSAT166605.1	1.874111	3.448062	3.398471	0.016415	0.143934	-3.09697
MSTRG.19995.1	-1.17714	2.09529	-3.39761	0.016431	0.143988	-3.09803
MSTRG.66005.1	1.225834	6.883777	3.397549	0.016432	0.143988	-3.09811
ENST00000382488.2	-1.36689	3.013214	-3.39731	0.016437	0.144	-3.09841
ENST00000656547.1	1.095798	3.798408	3.396861	0.016445	0.144053	-3.09896
NONHSAT195638.1	1.284936	2.369163	3.396741	0.016448	0.144053	-3.09911
lnc-KCNA4-9:2	-1.42817	2.720084	-3.39661	0.01645	0.144053	-3.09928
lnc-CEP63-3:1	1.040549	4.412762	3.396506	0.016452	0.144053	-3.09941
NONHSAT220287.1	1.235838	4.252657	3.396094	0.01646	0.144085	-3.09992
NONHSAT196959.1	1.306896	4.183724	3.39584	0.016465	0.144094	-3.10023
lnc-U2AF2-3:3	-1.11009	5.072226	-3.39574	0.016467	0.144094	-3.10035
MSTRG.17352.3	1.400331	2.960922	3.395451	0.016472	0.144094	-3.10071
lnc-RTP4-7:3	1.029533	4.174434	3.394934	0.016482	0.144128	-3.10136
NONHSAT152681.1	-1.29701	2.053711	-3.39434	0.016494	0.144177	-3.10209
NONHSAT186908.1	-1.30444	2.273853	-3.39301	0.01652	0.144319	-3.10374
NONHSAT171825.1	1.62294	2.994742	3.392877	0.016522	0.144319	-3.10391
T241203	-1.27708	2.853539	-3.39215	0.016536	0.144358	-3.10482
lnc-UGT8-3:1	1.140958	3.791838	3.392084	0.016537	0.144358	-3.1049
NONHSAT218055.1	1.438117	2.645746	3.391822	0.016542	0.14439	-3.10522
NONHSAT150405.1	1.230455	4.764958	3.391011	0.016558	0.144427	-3.10623
ENST00000447949.1	-1.41255	2.608889	-3.38996	0.016578	0.144542	-3.10753
MSTRG.7159.1	-1.3703	2.827781	-3.38996	0.016578	0.144542	-3.10753
NONHSAT178938.1	1.122903	2.179268	3.389475	0.016588	0.144565	-3.10814
ENST00000433933.1	-1.11275	4.534295	-3.38922	0.016593	0.144565	-3.10845
lnc-DHRSX-3:1	1.47727	2.188622	3.387336	0.016629	0.144729	-3.11079
lnc-IFNA8-3:1	-1.02613	1.608725	-3.38702	0.016636	0.144729	-3.11118
NONHSAT221976.1	-1.26656	2.021939	-3.38691	0.016638	0.144729	-3.11132
ENST00000433952.1	-1.35457	2.38859	-3.38647	0.016646	0.144757	-3.11187
ENST00000425802.1	-1.11313	1.894789	-3.38579	0.016659	0.144847	-3.11271
lnc-POMGNT2-1:2	1.30678	2.488985	3.384812	0.016679	0.144895	-3.11393
lnc-VWA3B-3:1	-1.53927	2.473307	-3.38465	0.016682	0.144895	-3.11413
MSTRG.46661.1	1.881874	6.394525	3.384614	0.016682	0.144895	-3.11417
NONHSAT215448.1	-1.06897	2.840944	-3.38459	0.016683	0.144895	-3.1142
lnc-ATP6V1E1-3:1	-1.41312	2.58792	-3.38339	0.016706	0.144971	-3.1157
NONHSAT204943.1	-1.21627	2.384101	-3.38247	0.016724	0.145073	-3.11684
NONHSAT183988.1	1.308724	1.827837	3.382025	0.016733	0.145117	-3.11739
NONHSAT199398.1	1.258605	2.285524	3.382002	0.016733	0.145117	-3.11742

MSTRG.46007.1	-1.09597	1.901112	-3.38199	0.016734	0.145117	-3.11744
NONHSAT188981.1	-1.06709	3.130969	-3.38162	0.016741	0.145151	-3.11789
T264838	1.201727	3.631225	3.379895	0.016775	0.145359	-3.12004
NONHSAT220733.1	-1.31503	2.019956	-3.37983	0.016776	0.145359	-3.12012
ENST00000513849.1	1.180956	2.79434	3.379417	0.016784	0.145392	-3.12063
NONHSAT179473.1	1.919244	3.120814	3.378866	0.016795	0.145453	-3.12132
T275431	1.182808	2.220498	3.378389	0.016804	0.145492	-3.12191
lnc-SLC22A9-1:1	1.035484	5.799671	3.377584	0.01682	0.145573	-3.12291
MSTRG.69717.3	1.303063	3.255992	3.377398	0.016824	0.145573	-3.12315
lnc-ZSWIM2-10:1	-1.1324	3.768854	-3.37737	0.016824	0.145573	-3.12318
NONHSAT155724.1	2.327035	3.76898	3.377182	0.016828	0.145573	-3.12341
NONHSAT201733.1	1.229367	3.830771	3.377116	0.016829	0.145573	-3.1235
MSTRG.22132.2	1.356657	1.788146	3.3771	0.01683	0.145573	-3.12352
lnc-MYLIP-5:1	1.061851	2.59529	3.377096	0.01683	0.145573	-3.12352
T191068	1.311004	5.15419	3.375893	0.016854	0.145648	-3.12502
NONHSAT161021.1	1.142533	3.792189	3.375272	0.016866	0.14568	-3.12579
lnc-BDH2-4:1	-1.30202	10.36061	-3.37516	0.016868	0.14568	-3.12593
NONHSAT218381.1	-1.70506	3.486274	-3.37503	0.01687	0.145689	-3.12609
lnc-SORCS3-4:3	1.683513	2.815075	3.374601	0.016879	0.145719	-3.12663
MSTRG.40483.1	1.079642	5.153518	3.374589	0.016879	0.145719	-3.12664
NR_003361	-1.65954	3.278582	-3.37457	0.01688	0.145719	-3.12667
NONHSAT158118.1	-1.35818	4.081465	-3.374	0.016891	0.145766	-3.12738
lnc-SP140L-1:3	-1.00576	5.348487	-3.37315	0.016908	0.145856	-3.12843
NONHSAT175460.1	2.088214	2.524389	3.373108	0.016909	0.145856	-3.12848
T264682	1.787988	2.348508	3.372896	0.016913	0.145857	-3.12875
NONHSAT171420.1	-1.67458	3.572616	-3.37265	0.016918	0.145857	-3.12906
NONHSAT185720.1	1.100703	3.781183	3.372558	0.016919	0.145857	-3.12917
lnc-BTG3-9:1	-1.32681	2.065718	-3.37252	0.01692	0.145857	-3.12922
NONHSAT186529.1	1.303191	2.776261	3.370967	0.016951	0.146006	-3.13115
ENST00000613067.1	-1.14229	2.511989	-3.36993	0.016972	0.146006	-3.13244
lnc-PRDM8-4:4	-1.00917	2.913522	-3.36987	0.016973	0.146006	-3.13252
MSTRG.37775.1	1.207887	4.225432	3.369589	0.016978	0.146006	-3.13286
NONHSAT201918.1	1.331972	2.560052	3.369263	0.016985	0.146006	-3.13327
T293539	1.274628	4.205236	3.368783	0.016994	0.146006	-3.13387
ENST00000424635.1	-1.11155	1.663758	-3.36785	0.017013	0.14605	-3.13503
MSTRG.13328.1	-1.7179	2.890647	-3.36781	0.017014	0.14605	-3.13508
NONHSAT200933.1	-1.3504	3.543833	-3.36707	0.017029	0.146093	-3.136
NONHSAT159058.1	1.176348	3.843261	3.367059	0.017029	0.146093	-3.13601
ENST00000662873.1	-1.67639	2.680094	-3.3653	0.017064	0.146253	-3.13821
lnc-USP38-1:1	1.101355	3.688881	3.364912	0.017072	0.146259	-3.13869
T153267	-1.03519	6.257282	-3.36474	0.017075	0.146259	-3.1389
NR_003686	-1.03279	3.829937	-3.36444	0.017081	0.146275	-3.13927
MSTRG.60692.16	-1.62064	2.860068	-3.36419	0.017086	0.146287	-3.13959
NONHSAT216693.1	-1.1128	4.054935	-3.36228	0.017125	0.14644	-3.14197
NONHSAT186819.1	1.689476	3.341817	3.36214	0.017127	0.14644	-3.14214
ENST00000483289.2	1.480039	2.849133	3.361824	0.017134	0.146444	-3.14254
ENST00000605021.1	-1.05947	1.817931	-3.36166	0.017137	0.146444	-3.14274
ENST00000654657.1	-1.29452	2.063573	-3.36152	0.01714	0.146444	-3.14292

lnc-CC2D1B-2:1	-1.18572	3.965596	-3.36146	0.017141	0.146444	-3.14298
lnc-AKR1E2-16:1	2.059173	3.341395	3.361323	0.017144	0.146446	-3.14316
NONHSAT153980.1	-1.71325	2.815507	-3.36124	0.017145	0.146446	-3.14327
lnc-ELOVL2-1:2	1.730241	3.386151	3.361123	0.017148	0.14645	-3.14341
NONHSAT157888.1	1.154502	4.881833	3.35958	0.017179	0.146562	-3.14533
lnc-MGAM-1:1	1.165028	5.538647	3.359386	0.017183	0.146568	-3.14557
NONHSAT171050.1	1.56314	2.846851	3.359373	0.017183	0.146568	-3.14559
NONHSAT191633.1	1.06126	4.510766	3.359155	0.017187	0.146593	-3.14586
NONHSAT178639.1	-1.31986	2.477188	-3.35889	0.017193	0.14662	-3.14619
NONHSAT212092.1	1.231558	4.45396	3.358855	0.017193	0.14662	-3.14624
NONHSAT153830.1	1.99221	3.662991	3.358195	0.017207	0.146708	-3.14706
ENST00000434255.1	1.961981	2.996573	3.357764	0.017216	0.146746	-3.1476
NONHSAT167372.1	1.228222	3.83987	3.357648	0.017218	0.146753	-3.14774
NONHSAT168619.1	2.407895	3.898327	3.35748	0.017221	0.14677	-3.14795
lnc-GNLY-1:1	-1.1697	2.461654	-3.35695	0.017232	0.146814	-3.14861
MSTRG.64920.17	1.508952	2.379969	3.356759	0.017236	0.146814	-3.14885
ENST00000514376.1	-1.66045	3.725886	-3.35628	0.017246	0.146829	-3.14945
ENST00000379640.1	1.682296	3.086398	3.355856	0.017254	0.146864	-3.14998
NONHSAT221889.1	-1.37751	3.09346	-3.35566	0.017258	0.146878	-3.15021
NONHSAT190492.1	1.633346	3.451159	3.355628	0.017259	0.146878	-3.15026
ENST00000491909.1	1.039977	2.61984	3.354817	0.017275	0.146981	-3.15127
ENST00000669963.1	1.380597	2.051249	3.354102	0.01729	0.147023	-3.15216
ENST00000651539.1	1.042753	3.049657	3.354032	0.017291	0.147023	-3.15225
ENST00000568384.1	-1.82812	3.042474	-3.35239	0.017325	0.147116	-3.1543
NONHSAT222475.1	1.033912	2.01303	3.352327	0.017326	0.147116	-3.15438
NONHSAT196929.1	1.202651	3.599649	3.352285	0.017327	0.147116	-3.15443
NONHSAT170333.1	1.626552	2.034846	3.352198	0.017328	0.147116	-3.15454
lnc-ZNF674-5:1	-1.25692	4.686504	-3.35163	0.01734	0.147129	-3.15525
ENST00000660070.1	1.447511	4.72795	3.350704	0.017359	0.147208	-3.1564
NONHSAT171730.1	1.011263	2.117216	3.35041	0.017365	0.147247	-3.15677
NONHSAT172570.1	1.239999	5.64347	3.349515	0.017383	0.147356	-3.15789
MSTRG.54686.1	1.812516	3.091388	3.349316	0.017387	0.147374	-3.15813
lnc-RASGRP1-7:1	-1.47831	2.508725	-3.34895	0.017395	0.1474	-3.15859
NONHSAT213799.1	-1.18675	4.383649	-3.34823	0.017409	0.147488	-3.15949
lnc-RUNDC1-3:1	-1.77559	3.50099	-3.34789	0.017416	0.147534	-3.15991
ENST00000595595.1	-1.91112	3.249747	-3.3472	0.01743	0.147625	-3.16077
lnc-NCBP2-AS2-9:2	-1.29102	3.064416	-3.34706	0.017433	0.147628	-3.16095
ENST00000615176.1	-1.60955	2.381857	-3.34667	0.017441	0.147661	-3.16144
lnc-ZIC4-1:2	1.378642	3.847416	3.345623	0.017463	0.147721	-3.16274
lnc-DACH2-2:1	-1.22035	2.724549	-3.34546	0.017466	0.147721	-3.16295
NONHSAT196942.1	2.032298	4.266377	3.345235	0.017471	0.147735	-3.16323
lnc-KLHL13-1:1	-1.24811	4.558127	-3.34457	0.017484	0.147771	-3.16406
ENST00000559959.1	-1.60166	3.333479	-3.34456	0.017485	0.147771	-3.16407
NONHSAT156612.1	1.065613	3.250928	3.344464	0.017487	0.147771	-3.16419
lnc-ILK-8:1	-1.32571	3.017782	-3.34439	0.017488	0.147771	-3.16429
NONHSAT191854.1	1.172895	2.054976	3.343456	0.017507	0.147858	-3.16545
NONHSAT196199.1	1.077428	3.153577	3.342951	0.017518	0.147896	-3.16608
lnc-STARD5-2:1	1.652014	2.792699	3.340762	0.017563	0.14803	-3.16882

MSTRG.19173.1	2.080803	2.852762	3.340268	0.017573	0.148066	-3.16943
lnc-RTL1-10:1	-1.10766	4.268333	-3.34012	0.017576	0.14808	-3.16962
NONHSAT193198.1	1.694246	2.030408	3.33968	0.017585	0.148119	-3.17017
ENST00000534594.1	-1.09249	4.661359	-3.33716	0.017637	0.148401	-3.17331
ENST00000598079.1	1.035638	4.832182	3.336003	0.017661	0.148559	-3.17476
lnc-ERI1-10:1	1.24758	4.412634	3.335235	0.017677	0.14866	-3.17572
lnc-RGMB-6:1	-1.05134	3.309004	-3.33497	0.017683	0.148678	-3.17606
NONHSAT167408.1	1.405086	3.989371	3.334736	0.017688	0.148678	-3.17635
NONHSAT170064.1	1.141241	3.811869	3.33449	0.017693	0.148678	-3.17666
ENST00000416076.1	-1.01754	3.812076	-3.33421	0.017699	0.148679	-3.177
ENST00000525133.2	-1.60985	2.745557	-3.33399	0.017703	0.148679	-3.17727
ENST00000623789.1	1.346125	1.790103	3.333886	0.017706	0.148679	-3.17741
T352292	1.01298	2.250745	3.333865	0.017706	0.148679	-3.17744
LINC00113:4	1.416506	4.919487	3.332954	0.017725	0.14878	-3.17858
NONHSAT198545.1	1.358833	2.197402	3.332781	0.017729	0.14878	-3.17879
T089078	-1.02077	3.373407	-3.33228	0.017739	0.148831	-3.17942
lnc-RIMS3-5:1	1.126676	3.410005	3.331875	0.017748	0.148879	-3.17992
lnc-ALG12-5:3	-1.12914	3.374433	-3.3314	0.017758	0.148879	-3.18052
ENST00000621331.1	-1.51746	2.677197	-3.32918	0.017804	0.149053	-3.1833
lnc-UBASH3B-7:1	-2.14322	3.668003	-3.32843	0.01782	0.149147	-3.18423
lnc-DCLRE1B-12:4	1.174676	2.053783	3.327836	0.017832	0.14918	-3.18498
NONHSAT187424.1	1.147854	6.468466	3.327138	0.017847	0.149233	-3.18585
NONHSAT214483.1	1.20171	9.260794	3.326732	0.017855	0.14926	-3.18636
NONHSAT179786.1	1.02441	8.138284	3.326358	0.017863	0.149268	-3.18683
NONHSAT201849.1	1.584891	2.342964	3.325876	0.017873	0.14927	-3.18743
ENST00000572608.1	-1.04304	3.647985	-3.32583	0.017874	0.14927	-3.18749
lnc-KMT5A-20:1	1.271955	6.610511	3.325081	0.01789	0.149278	-3.18843
NONHSAT207029.1	-1.08626	2.867802	-3.32467	0.017899	0.149313	-3.18893
lnc-CWH43-8:1	-1.65262	2.462154	-3.32457	0.017901	0.14932	-3.18907
ENST00000522028.1	1.312198	1.853745	3.324328	0.017906	0.14932	-3.18937
ENST00000567777.1	1.183011	2.817673	3.323958	0.017914	0.149328	-3.18983
NONHSAT199981.1	1.335562	3.58377	3.323321	0.017927	0.149354	-3.19063
lnc-PTGIR-4:1	-1.30001	2.190947	-3.32258	0.017943	0.149424	-3.19155
ENST00000658855.1	1.289242	2.067606	3.322273	0.017949	0.149448	-3.19194
lnc-B3GALT2-2:1	-1.72007	2.733712	-3.32223	0.01795	0.149448	-3.192
NONHSAT217763.1	1.520559	2.192745	3.322024	0.017955	0.149471	-3.19225
MSTRG.48258.1	1.457679	2.62766	3.321851	0.017958	0.149489	-3.19247
ENST00000432703.1	-1.00496	3.163986	-3.32142	0.017967	0.149512	-3.19301
NONHSAT224278.1	1.343631	2.478556	3.321305	0.01797	0.149512	-3.19315
lnc-ALK-3:1	1.020295	3.620603	3.320516	0.017987	0.149601	-3.19414
lnc-FSHR-7:1	1.837284	1.950749	3.318809	0.018023	0.14973	-3.19628
NONHSAT223935.1	1.079397	2.408127	3.318537	0.018029	0.149752	-3.19662
MSTRG.12383.1	-1.33499	2.930598	-3.31818	0.018036	0.149766	-3.19707
MSTRG.623.1	1.691471	3.288118	3.318116	0.018038	0.149766	-3.19715
MSTRG.11688.1	1.613182	1.922358	3.317788	0.018045	0.14978	-3.19756
MSTRG.27438.45	-1.00299	3.501211	-3.31768	0.018047	0.14978	-3.19769
MSTRG.40085.1	1.020066	1.591137	3.317148	0.018058	0.149839	-3.19836
ENST00000565271.1	-1.4327	3.488542	-3.31649	0.018072	0.149905	-3.19918

NONHSAT168417.1	1.364691	2.131188	3.31538	0.018096	0.150012	-3.20057
NONHSAT210868.1	1.184601	2.513906	3.315203	0.0181	0.150012	-3.2008
lnc-UGT8-10:1	1.280806	3.442054	3.314882	0.018107	0.150012	-3.2012
NONHSAT218493.1	1.572899	3.173134	3.314797	0.018108	0.150012	-3.2013
lnc-ANGPTL5-1:1	1.462958	4.062185	3.314648	0.018112	0.150012	-3.20149
T161830	-1.12779	1.808964	-3.31438	0.018117	0.150033	-3.20183
T310278	-1.06896	2.396425	-3.31393	0.018127	0.150063	-3.20239
NONHSAT187073.1	-1.79314	2.932407	-3.31383	0.018129	0.150066	-3.20252
lnc-UMODL1-8:1	-1.23416	2.203408	-3.31349	0.018136	0.150098	-3.20294
lnc-FOXD4L5-28:1	1.362828	3.018785	3.312788	0.018151	0.150103	-3.20382
MSTRG.62695.1	1.416997	2.244395	3.31269	0.018154	0.150103	-3.20395
NONHSAT186821.1	1.635138	3.034881	3.312603	0.018155	0.150103	-3.20406
T351259	1.270866	1.738706	3.312261	0.018163	0.150103	-3.20448
NONHSAT209258.1	-1.1729	1.943468	-3.31225	0.018163	0.150103	-3.2045
MSTRG.1901.1	1.051348	1.631935	3.312242	0.018163	0.150103	-3.20451
lnc-SECISBP2L-2:1	-1.14943	4.646786	-3.31189	0.018171	0.150128	-3.20494
MSTRG.38790.1	1.40695	2.805382	3.311145	0.018187	0.150199	-3.20588
NONHSAT197213.1	1.31618	2.861834	3.310548	0.0182	0.150256	-3.20663
NR_040080	1.273999	3.555558	3.310149	0.018208	0.150315	-3.20713
lnc-NABP1-1:2	1.334406	2.410342	3.308639	0.018241	0.150457	-3.20903
T038232	1.05905	1.700678	3.308179	0.018251	0.15048	-3.2096
lnc-GMNC-4:1	-1.04333	4.00302	-3.30731	0.018269	0.150511	-3.21069
lnc-DNASE1-8:1	1.047771	8.235745	3.307188	0.018272	0.15052	-3.21084
lnc-SENP6-2:12	-1.80612	3.243992	-3.307	0.018276	0.15053	-3.21108
NONHSAT197417.1	1.196896	2.15857	3.306686	0.018283	0.150549	-3.21147
NR_002947	1.165938	2.061837	3.306343	0.01829	0.150577	-3.21191
NONHSAT153897.1	1.013406	1.981735	3.305758	0.018303	0.150628	-3.21264
NONHSAT203276.1	1.05682	2.390788	3.305327	0.018312	0.150655	-3.21318
lnc-LMOD1-5:1	1.314821	3.050426	3.305076	0.018318	0.150678	-3.21349
lnc-PIGF-2:1	-1.15657	2.198998	-3.30459	0.018328	0.150697	-3.2141
lnc-FAM178B-1:1	-1.19507	2.216814	-3.30453	0.018329	0.150697	-3.21418
NR_125407	1.025802	5.066976	3.304139	0.018338	0.150703	-3.21467
NONHSAT201612.1	1.07015	3.182105	3.303904	0.018343	0.150724	-3.21496
lnc-MYO16-8:1	1.189872	2.062941	3.303681	0.018348	0.150728	-3.21524
lnc-PWP2-2:3	1.544539	2.328769	3.302817	0.018367	0.150845	-3.21633
ENST00000617211.1	-1.01673	6.688226	-3.3019	0.018386	0.150942	-3.21748
lnc-SLC2A9-4:1	-1.17171	4.21913	-3.30137	0.018398	0.150942	-3.21814
lnc-RGCC-1:1	1.194453	2.625021	3.301319	0.018399	0.150942	-3.21821
ENST00000427182.1	-1.35418	2.379526	-3.3013	0.0184	0.150942	-3.21824
lnc-CHRNA5-3:3	1.768059	2.199781	3.299796	0.018432	0.151085	-3.22012
NONHSAT170745.1	1.526748	7.827657	3.299768	0.018433	0.151085	-3.22016
lnc-NBAS-10:1	1.136932	4.229659	3.299137	0.018447	0.151108	-3.22095
lnc-KLHL1-11:1	-1.61991	2.668079	-3.2985	0.01846	0.151142	-3.22174
NONHSAT154188.1	1.345035	1.844638	3.297844	0.018475	0.15118	-3.22257
lnc-TRMT11-2:2	-1.35283	2.37541	-3.2977	0.018478	0.151194	-3.22275
MSTRG.32668.1	1.438361	2.990353	3.297544	0.018481	0.151206	-3.22295
MSTRG.1479.1	1.942029	2.602551	3.297424	0.018484	0.151206	-3.2231
ENST00000663238.1	1.10126	5.668528	3.297063	0.018492	0.151258	-3.22355

lnc-SCYL3-2:1	-1.23421	3.669649	-3.29682	0.018497	0.151278	-3.22386
NONHSAT192036.1	-1.48178	3.820976	-3.29613	0.018512	0.151347	-3.22472
lnc-EPB41-3:1	1.235268	4.87164	3.296086	0.018513	0.151347	-3.22478
ENST00000507389.1	1.080565	1.563392	3.295151	0.018534	0.15143	-3.22595
lnc-PACRGL-12:2	-1.38433	2.287432	-3.2946	0.018546	0.151461	-3.22664
MSTRG.39322.1	1.093431	1.594061	3.294038	0.018558	0.15153	-3.22735
ENST00000591742.1	1.067692	4.171915	3.293635	0.018567	0.151566	-3.22786
lnc-RWDD3-1:8	1.023385	3.084047	3.292571	0.018591	0.151646	-3.22919
ENST00000554515.1	1.848851	2.692998	3.29242	0.018594	0.151661	-3.22938
MSTRG.49618.5	1.008331	1.998019	3.29207	0.018602	0.151685	-3.22982
lnc-LIPM-1:2	-1.20329	4.534335	-3.29202	0.018603	0.151685	-3.22989
ENST00000606105.1	-1.10115	4.45223	-3.29201	0.018603	0.151685	-3.22989
MSTRG.54890.1	-1.68711	3.477018	-3.2916	0.018612	0.151724	-3.23042
MSTRG.64103.1	-1.44896	2.512115	-3.29159	0.018612	0.151724	-3.23042
NONHSAT174806.1	2.0137	3.345881	3.291376	0.018617	0.151734	-3.2307
NONHSAT171377.1	1.658188	3.234518	3.291128	0.018622	0.151758	-3.23101
SNAI3-AS1:21	1.290561	3.718183	3.29092	0.018627	0.151772	-3.23127
lnc-COG6-7:1	1.547721	2.207239	3.290749	0.018631	0.151772	-3.23148
NONHSAT161158.1	-1.32593	4.015118	-3.29056	0.018635	0.151775	-3.23173
ENST00000648110.1	1.48715	2.282853	3.289256	0.018664	0.151855	-3.23336
lnc-MS4A4A-1:1	1.60755	2.544114	3.289225	0.018664	0.151855	-3.2334
NONHSAT197099.1	1.330889	1.840618	3.288686	0.018676	0.151886	-3.23408
MSTRG.13005.1	-1.16114	2.370908	-3.28855	0.018679	0.151886	-3.23425
ENST00000317596.3	-1.22856	3.311811	-3.28828	0.018685	0.151902	-3.23458
NONHSAT164688.1	1.484872	2.041336	3.28676	0.018719	0.152043	-3.2365
lnc-TMEM170A-2:1	-1.26934	2.117929	-3.28594	0.018737	0.152081	-3.23753
ENST00000654106.1	-1.18065	2.841233	-3.28589	0.018738	0.152081	-3.2376
NONHSAT164610.1	1.254776	3.070074	3.285874	0.018739	0.152081	-3.23761
T256644	1.20908	6.884218	3.28586	0.018739	0.152081	-3.23763
lnc-ACBD4-1:2	1.229634	3.529152	3.285574	0.018745	0.15209	-3.23799
NONHSAT214981.1	1.89869	2.103446	3.28552	0.018746	0.15209	-3.23806
NONHSAT204512.1	1.187049	2.11685	3.285351	0.01875	0.15209	-3.23827
T351389	-1.5953	4.262875	-3.28529	0.018751	0.15209	-3.23834
lnc-PRDM9-11:1	-1.62453	2.884366	-3.28523	0.018753	0.15209	-3.23842
lnc-PLA2G2D-2:1	1.882237	3.525858	3.285184	0.018754	0.15209	-3.23848
ENST00000562127.1	1.689979	6.478451	3.284972	0.018759	0.152094	-3.23875
MSTRG.51542.1	2.183082	2.769998	3.283855	0.018783	0.152186	-3.24015
ENST00000503268.2	1.043515	2.056808	3.283725	0.018786	0.152197	-3.24031
ENST00000665447.1	1.532108	2.44095	3.283479	0.018792	0.152202	-3.24062
NONHSAT197931.1	-1.21947	2.315328	-3.28344	0.018793	0.152202	-3.24067
NONHSAT188228.1	-1.42063	1.902579	-3.28293	0.018804	0.152251	-3.24131
NONHSAT197038.1	1.092734	2.912546	3.282885	0.018805	0.152251	-3.24137
ENST00000613780.4	-1.11347	3.379093	-3.28244	0.018815	0.152263	-3.24193
lnc-STK26-11:1	1.559105	2.993801	3.28216	0.018821	0.152295	-3.24228
lnc-PFDN4-3:2	1.463907	6.210101	3.282096	0.018823	0.152295	-3.24236
lnc-SLITRK5-13:2	1.611187	2.42306	3.281856	0.018828	0.152318	-3.24267
T346504	1.762216	2.59769	3.281822	0.018829	0.152318	-3.24271
NONHSAT223451.1	1.331367	1.743073	3.281185	0.018843	0.152362	-3.24351

MSTRG.70417.1	1.039838	3.70959	3.280754	0.018853	0.152397	-3.24405
ENST00000469794.1	-1.30337	5.806897	-3.27924	0.018887	0.152487	-3.24596
lnc-HLX-6:1	1.001388	3.358759	3.279142	0.018889	0.152487	-3.24608
T258078	-1.24714	4.131447	-3.27855	0.018902	0.152533	-3.24682
NONHSAT167853.1	-1.25435	2.296675	-3.27849	0.018903	0.152533	-3.2469
NONHSAT169981.1	1.033014	4.388274	3.278261	0.018908	0.152561	-3.24719
lnc-ZNF267-5:1	1.237057	3.161361	3.277776	0.018919	0.152601	-3.2478
NONHSAT209445.1	1.159267	1.765104	3.277507	0.018925	0.152612	-3.24814
ENST00000397750.7	-1.16231	5.459948	-3.27575	0.018965	0.152784	-3.25035
ENST00000564059.1	1.322397	2.042246	3.275232	0.018976	0.152841	-3.251
NONHSAT184982.1	1.074662	1.632651	3.275062	0.01898	0.152852	-3.25121
lnc-TBX4-3:1	-1.23634	2.105584	-3.27462	0.01899	0.152879	-3.25178
lnc-MED13L-7:1	1.027682	4.088851	3.273753	0.01901	0.152952	-3.25286
NONHSAT158331.1	1.332388	2.056661	3.273584	0.019014	0.152969	-3.25308
NONHSAT222154.1	1.817742	2.857298	3.272721	0.019033	0.153089	-3.25416
ENST00000434589.1	1.042866	3.775832	3.272044	0.019048	0.153102	-3.25502
T309646	-1.01637	3.561188	-3.27194	0.019051	0.153102	-3.25515
NONHSAT188112.1	1.928078	3.417932	3.271728	0.019056	0.153102	-3.25541
NONHSAT214142.1	1.387196	3.098944	3.271338	0.019064	0.153102	-3.25591
ENST00000660833.1	-1.32124	2.488875	-3.27001	0.019094	0.153265	-3.25758
NONHSAT216855.1	-1.31712	5.660517	-3.26987	0.019098	0.153278	-3.25775
lnc-SS18-6:1	-2.19414	3.892415	-3.26972	0.019101	0.153294	-3.25795
MSTRG.70907.1	-1.66933	3.435543	-3.26889	0.01912	0.153371	-3.25899
lnc-PPP2R3A-1:1	-2.05683	2.098789	-3.26863	0.019126	0.153394	-3.25932
MSTRG.63216.1	1.434833	2.698942	3.268472	0.019129	0.153394	-3.25951
ENST00000654026.1	1.644107	2.716584	3.268422	0.01913	0.153394	-3.25958
MSTRG.51854.1	-1.18872	2.763093	-3.26779	0.019145	0.153463	-3.26038
ENST00000445459.2	-2.24788	3.529003	-3.26667	0.01917	0.153552	-3.26178
MSTRG.36946.1	1.09315	2.426038	3.266159	0.019182	0.153559	-3.26243
NONHSAT210411.1	1.386868	2.827058	3.265999	0.019186	0.153559	-3.26263
lnc-NUTM2G-1:1	-1.12002	4.331624	-3.26582	0.01919	0.153559	-3.26286
MSTRG.27081.1	1.155521	3.959953	3.26526	0.019202	0.153559	-3.26356
MSTRG.54104.1	1.323671	2.077678	3.265251	0.019203	0.153559	-3.26357
NONHSAT186351.1	1.701631	2.494641	3.264972	0.019209	0.153588	-3.26393
NONHSAT150085.1	-1.07594	2.585604	-3.26494	0.01921	0.153588	-3.26397
NONHSAT190119.1	1.094355	3.321318	3.263249	0.019248	0.153775	-3.2661
T098583	1.118467	4.296026	3.262901	0.019256	0.153775	-3.26654
lnc-NSMCE1-6:1	1.456149	1.957082	3.262892	0.019257	0.153775	-3.26655
NONHSAT218713.1	1.554008	5.257541	3.262731	0.01926	0.153775	-3.26675
ENST00000663020.1	1.041574	1.601118	3.260945	0.019301	0.154003	-3.269
NONHSAT187284.1	1.250935	2.04522	3.260937	0.019301	0.154003	-3.26901
MSTRG.19434.1	-1.18739	2.451666	-3.26073	0.019306	0.154003	-3.26927
ENST00000418966.1	1.137084	7.382124	3.260079	0.019321	0.15409	-3.27009
lnc-ZNF41-1:1	1.55278	2.344973	3.259132	0.019343	0.154213	-3.27129
NONHSAT224281.1	1.954584	2.728932	3.258317	0.019362	0.154284	-3.27232
NONHSAT181417.1	1.204006	1.66776	3.257428	0.019382	0.154312	-3.27344
lnc-NXPH3-1:1	1.281213	2.49255	3.25721	0.019387	0.154317	-3.27371
ENST00000563464.1	-1.35881	2.466	-3.25653	0.019403	0.154344	-3.27458

ENST00000431442.2	-1.2153	2.688872	-3.2565	0.019403	0.154344	-3.27461
MSTRG.68058.1	-1.06676	4.464649	-3.25629	0.019408	0.154344	-3.27487
ENST00000614876.1	-1.49179	2.489161	-3.25605	0.019414	0.154344	-3.27518
MSTRG.9388.1	1.310192	4.374143	3.255877	0.019418	0.154344	-3.27539
lnc-DHX37-20:1	1.071912	3.145867	3.255718	0.019421	0.154344	-3.27559
T203653	1.428909	2.893369	3.254931	0.01944	0.154438	-3.27659
T197874	1.03516	2.240283	3.254417	0.019452	0.154484	-3.27724
lnc-PRORY-5:1	1.589779	2.012204	3.254235	0.019456	0.154484	-3.27747
MSTRG.60676.1	1.585959	3.257417	3.253933	0.019463	0.154499	-3.27785
NR_110559	1.431611	3.666084	3.253499	0.019473	0.154541	-3.2784
NONHSAT207185.1	-1.35549	3.178879	-3.25235	0.0195	0.154682	-3.27985
NONHSAT213836.1	1.356512	3.22494	3.251364	0.019522	0.154738	-3.28109
lnc-UTP23-5:1	1.294637	2.060046	3.250523	0.019542	0.154828	-3.28215
NONHSAT199406.1	1.192275	2.617423	3.250495	0.019543	0.154828	-3.28219
NONHSAT197244.1	1.159497	4.861683	3.250366	0.019546	0.154835	-3.28235
NONHSAT214389.1	-2.02778	3.906987	-3.24987	0.019557	0.154858	-3.28297
LINC00567:4	1.184532	3.737918	3.249387	0.019568	0.154911	-3.28359
NONHSAT157802.1	1.052372	3.703482	3.249116	0.019575	0.154931	-3.28393
ENST00000648448.1	1.600872	3.472914	3.248297	0.019594	0.155051	-3.28496
NONHSAT163852.1	2.115936	2.554282	3.247593	0.01961	0.155157	-3.28585
T257300	1.13341	5.547653	3.247106	0.019622	0.155214	-3.28647
NONHSAT210356.1	1.263817	2.916088	3.246624	0.019633	0.155226	-3.28708
lnc-ISX-5:1	1.664215	2.341028	3.246545	0.019635	0.155226	-3.28718
T083987	-1.39696	3.853894	-3.24649	0.019636	0.155226	-3.28724
NONHSAT221575.1	-1.4161	4.419072	-3.24594	0.019649	0.15527	-3.28794
lnc-KRTAP10-12-2:1	1.094545	5.122736	3.245154	0.019667	0.155371	-3.28893
NONHSAT194721.1	-1.45831	2.865404	-3.24485	0.019674	0.155371	-3.28932
NONHSAT221251.1	1.075061	1.852385	3.244796	0.019676	0.155371	-3.28938
NONHSAT224201.1	-1.15707	2.904911	-3.24437	0.019686	0.155399	-3.28992
NONHSAT158179.1	1.05232	3.539835	3.243993	0.019694	0.155412	-3.2904
lnc-ANXA10-1:1	1.320334	2.96617	3.243992	0.019694	0.155412	-3.2904
lnc-NAA35-4:1	-1.07763	4.626291	-3.24388	0.019697	0.155412	-3.29055
MSTRG.15684.1	1.084993	4.51793	3.243785	0.019699	0.155412	-3.29066
NONHSAT166310.1	2.096739	3.702957	3.243575	0.019704	0.155413	-3.29093
NONHSAT174618.1	1.215905	2.442666	3.24238	0.019732	0.155498	-3.29244
NR_104061	1.468006	2.507741	3.240333	0.019781	0.155662	-3.29502
MSTRG.18216.22	1.124576	3.927989	3.240311	0.019781	0.155662	-3.29505
lnc-TDRD9-3:1	1.066552	2.127968	3.240268	0.019782	0.155662	-3.29511
lnc-ARHGAP29-7:1	1.143593	3.872496	3.239846	0.019792	0.155716	-3.29564
lnc-PPP2R2D-8:1	1.45393	2.508899	3.238316	0.019828	0.155842	-3.29757
lnc-NME3-2:2	1.386054	2.349029	3.237863	0.019839	0.155882	-3.29815
NONHSAT210350.1	-1.35521	1.966619	-3.23774	0.019842	0.155882	-3.2983
lnc-SPACA7-26:1	1.213307	2.150175	3.237465	0.019848	0.155891	-3.29865
NONHSAT205841.1	1.313176	1.713316	3.237282	0.019853	0.155912	-3.29888
MSTRG.1673.2	1.081268	4.814378	3.236885	0.019862	0.15596	-3.29938
lnc-PCLO-2:1	1.175628	1.705187	3.23632	0.019875	0.156004	-3.3001
MSTRG.68535.1	1.287416	2.530726	3.236153	0.019879	0.156013	-3.30031
LINC02140:12	-1.23217	2.489639	-3.23521	0.019902	0.156086	-3.3015

lnc-CDC42BPB-5:8	-1.35217	2.59167	-3.23517	0.019903	0.156086	-3.30155
lnc-MCC-2:1	1.158043	3.792378	3.234409	0.019921	0.156168	-3.30251
lnc-COG3-5:1	-1.5624	4.363953	-3.23383	0.019935	0.156198	-3.30325
MSTRG.3511.1	1.70456	2.398747	3.233003	0.019954	0.156239	-3.30429
NONHSAT185446.1	1.066264	3.914484	3.232793	0.019959	0.156239	-3.30456
NONHSAT163888.1	1.438743	4.906402	3.232717	0.019961	0.156239	-3.30465
lnc-OGFRL1-7:1	-1.49768	2.345019	-3.23246	0.019967	0.156239	-3.30498
lnc-VEGFC-4:1	1.007358	5.707314	3.232033	0.019977	0.156283	-3.30552
ENST00000508985.1	1.022148	3.267377	3.231033	0.020001	0.156407	-3.30679
lnc-CYLC2-7:1	1.033859	1.565686	3.229313	0.020042	0.156657	-3.30896
lnc-SDSL-9:1	1.325096	1.767134	3.2285	0.020062	0.156749	-3.30999
ENST00000419957.6	1.096673	3.374981	3.228352	0.020065	0.156764	-3.31018
NONHSAT170130.1	1.001809	4.850761	3.227999	0.020074	0.15681	-3.31063
lnc-RLIM-1:2	1.142338	2.295752	3.227888	0.020076	0.15681	-3.31077
lnc-IFT74-7:1	1.390634	2.000271	3.227847	0.020077	0.15681	-3.31082
MSTRG.50374.1	1.545365	5.121426	3.227677	0.020081	0.156823	-3.31103
ENST00000567305.1	-1.06849	4.508595	-3.22745	0.020087	0.156824	-3.31132
lnc-FAM83A-5:1	-1.08052	4.598673	-3.22641	0.020112	0.156921	-3.31263
NONHSAT161307.1	1.714617	2.79549	3.226209	0.020117	0.156947	-3.31289
MSTRG.48462.1	1.001518	3.104516	3.224886	0.020148	0.157069	-3.31457
lnc-MRPL19-14:1	-1.0943	1.780523	-3.22459	0.020156	0.15707	-3.31494
ENST00000507403.1	1.258102	3.511522	3.224583	0.020156	0.15707	-3.31495
NONHSAT216460.1	1.037211	2.468444	3.224315	0.020162	0.157096	-3.31529
MSTRG.3690.1	1.770164	2.843178	3.223908	0.020172	0.157124	-3.3158
MSTRG.37326.1	1.446582	2.487308	3.223624	0.020179	0.157132	-3.31616
NONHSAT197480.1	1.252051	2.011738	3.223614	0.020179	0.157132	-3.31618
lnc-ARSJ-1:8	-1.13184	4.581528	-3.22246	0.020207	0.157221	-3.31764
ENST00000603521.1	-1.05257	5.433166	-3.2224	0.020208	0.157221	-3.31772
lnc-SLC11A2-4:1	-1.18218	4.072579	-3.22223	0.020213	0.157221	-3.31793
NONHSAT222202.1	1.78611	2.789488	3.21938	0.020281	0.157514	-3.32154
lnc-IGFBP7-2:1	1.327761	2.144005	3.219068	0.020289	0.15756	-3.32193
lnc-CRTC3-4:1	1.34776	2.196753	3.218713	0.020298	0.1576	-3.32238
NONHSAT155897.1	-1.57802	3.378576	-3.21851	0.020303	0.157605	-3.32265
T350762	1.140271	4.88834	3.217774	0.02032	0.157622	-3.32357
MSTRG.1887.1	-1.02075	2.485316	-3.21766	0.020323	0.15763	-3.32371
PRKAG2-AS1:4	-1.02015	1.811767	-3.21623	0.020358	0.157779	-3.32553
NONHSAT221748.1	1.635603	2.511491	3.215455	0.020377	0.157902	-3.32651
T363691	1.168704	2.049971	3.215291	0.020381	0.157908	-3.32672
NONHSAT154639.1	-1.3194	1.795305	-3.21495	0.020389	0.157941	-3.32716
T145022	-1.04771	3.094086	-3.21482	0.020392	0.157941	-3.32732
NONHSAT201178.1	1.072228	2.968331	3.214677	0.020396	0.157941	-3.3275
MSTRG.71160.16	1.092264	4.76913	3.213761	0.020418	0.158015	-3.32866
NONHSAT196914.1	-1.28666	1.891919	-3.2136	0.020422	0.158021	-3.32886
NONHSAT161203.1	1.012242	3.804944	3.212891	0.020439	0.158107	-3.32976
NONHSAT201466.1	1.113216	4.172389	3.212406	0.020451	0.158168	-3.33038
ENST00000662277.1	-1.4311	2.631774	-3.212	0.020461	0.158218	-3.33089
lnc-KIN-5:1	1.334135	2.472873	3.211698	0.020469	0.15826	-3.33128
lnc-CLK4-3:1	1.136632	3.748106	3.210054	0.020509	0.158353	-3.33336

lnc-ITPRIP-6:1	1.093847	6.629594	3.209838	0.020514	0.158379	-3.33364
ENST00000370380.2	-1.33502	2.003498	-3.20948	0.020523	0.158379	-3.33408
NONHSAT170715.1	-1.58888	2.996233	-3.20895	0.020536	0.15838	-3.33476
NONHSAT187861.1	1.017656	3.231606	3.208804	0.02054	0.158384	-3.33495
lnc-FAM3B-3:1	-1.06135	3.474574	-3.20865	0.020543	0.158396	-3.33514
ENST00000430651.1	1.000575	8.474413	3.2086	0.020545	0.158396	-3.33521
lnc-ZFP42-8:1	-1.2556	1.829368	-3.20835	0.020551	0.158396	-3.33552
lnc-SLC25A48-3:3	1.592887	2.279997	3.208319	0.020552	0.158396	-3.33556
lnc-RHOV-1:1	-1.28731	2.751522	-3.20817	0.020555	0.158396	-3.33575
ENST00000421002.1	1.054374	6.069504	3.207952	0.020561	0.158411	-3.33603
NONHSAT163191.1	-1.27578	4.008533	-3.20726	0.020578	0.158459	-3.33691
lnc-MACC1-1:11	-1.06292	3.067497	-3.2069	0.020587	0.158497	-3.33737
lnc-TRMT11-3:1	-1.17331	2.946283	-3.20668	0.020592	0.158497	-3.33765
MSTRG.29187.1	1.380742	2.11608	3.2054	0.020623	0.158642	-3.33927
ENST00000507424.1	1.175011	1.630467	3.205399	0.020623	0.158642	-3.33927
NONHSAT197816.1	-2.07149	3.579081	-3.20482	0.020638	0.158693	-3.34001
lnc-C1orf74-3:1	-1.12711	4.091572	-3.2041	0.020656	0.158805	-3.34092
lnc-ZNF649-3:6	-1.24198	2.471079	-3.20392	0.02066	0.158815	-3.34115
ENST00000651165.1	1.394122	2.392636	3.203493	0.020671	0.158848	-3.34168
lnc-ABL2-1:1	-1.27616	3.136016	-3.20272	0.02069	0.158897	-3.34266
NONHSAT186971.1	-1.40994	1.866268	-3.20265	0.020691	0.158898	-3.34275
MSTRG.29530.2	-1.10858	4.833158	-3.20219	0.020703	0.158914	-3.34334
NONHSAT196754.1	1.402567	2.223258	3.201701	0.020715	0.158971	-3.34396
ENST00000668286.1	1.386026	6.664199	3.201317	0.020725	0.159007	-3.34445
NONHSAT161115.1	-1.16452	3.449956	-3.20077	0.020738	0.159063	-3.34514
MSTRG.16812.3	1.303519	1.884302	3.199937	0.020759	0.15913	-3.3462
NONHSAT210419.1	1.318082	8.286115	3.199735	0.020764	0.15914	-3.34646
NONHSAT186288.1	-1.16335	2.282887	-3.19944	0.020771	0.159158	-3.34683
lnc-ELP3-1:1	-1.21589	3.965045	-3.19927	0.020775	0.159158	-3.34705
ENST00000660450.1	-1.31858	2.09283	-3.19899	0.020782	0.159158	-3.34741
NONHSAT149434.1	-1.05376	3.737737	-3.19855	0.020793	0.159184	-3.34796
MSTRG.65993.1	-1.05431	2.311172	-3.19837	0.020798	0.159204	-3.34819
NONHSAT215088.1	-1.06351	2.679742	-3.19812	0.020804	0.159231	-3.34851
ENST00000448963.5	1.244512	2.141468	3.196666	0.02084	0.159387	-3.35035
ENST00000658162.1	1.127806	7.679237	3.196116	0.020854	0.159477	-3.35105
LINC01337:1	-1.556	3.958348	-3.19484	0.020886	0.159603	-3.35268
NONHSAT172699.1	-1.42256	4.879839	-3.19432	0.020899	0.159666	-3.35334
lnc-ATP6V0D1-1:2	1.193864	1.907769	3.193577	0.020918	0.159733	-3.35428
NONHSAT153522.1	1.199719	2.654959	3.19344	0.020921	0.159733	-3.35445
ENST00000510203.1	1.177889	4.826669	3.193335	0.020924	0.159733	-3.35458
MSTRG.65892.3	1.036978	2.689269	3.192552	0.020943	0.159775	-3.35558
lnc-NCOR2-7:1	1.927976	3.346433	3.191341	0.020974	0.159886	-3.35712
NONHSAT173357.1	-1.37261	3.261281	-3.19116	0.020978	0.159909	-3.35735
MSTRG.70295.1	1.194272	2.129299	3.190873	0.020986	0.159928	-3.35771
MSTRG.34997.2	1.326722	2.224144	3.190373	0.020998	0.159967	-3.35835
lnc-KANSL1-7:3	-1.1111	1.793035	-3.19029	0.021	0.159967	-3.35845
lnc-DSG1-13:2	-1.20836	1.657216	-3.19029	0.021	0.159967	-3.35846
NONHSAT218785.1	-1.16945	2.485545	-3.18924	0.021027	0.160079	-3.35978

NONHSAT175806.1	1.673513	2.0335	3.18922	0.021027	0.160079	-3.35981
MSTRG.38899.7	1.77238	3.669874	3.188765	0.021039	0.160151	-3.36039
lnc-UBA1-1:1	1.261518	2.84528	3.187269	0.021077	0.160366	-3.36229
NONHSAT197195.1	-1.23982	2.919741	-3.18675	0.02109	0.160421	-3.36295
NONHSAT196992.1	1.34373	6.949708	3.1867	0.021091	0.160421	-3.36302
lnc-DCUN1D1-12:1	1.59739	2.841448	3.186444	0.021097	0.160428	-3.36334
NONHSAT182409.1	-1.02649	5.425981	-3.18625	0.021102	0.160451	-3.36359
NONHSAT169688.1	-1.02086	1.615345	-3.18598	0.021109	0.160465	-3.36393
NONHSAT201319.1	1.379933	1.808087	3.185975	0.021109	0.160465	-3.36394
lnc-SPTLC3-7:1	1.783409	2.101417	3.185531	0.021121	0.160471	-3.36451
NONHSAT203064.1	1.040373	3.680349	3.185171	0.02113	0.160504	-3.36496
ENST00000548779.1	-1.01119	5.188241	-3.18502	0.021134	0.160521	-3.36516
NONHSAT203399.1	1.611536	2.827442	3.184237	0.021153	0.160611	-3.36615
ENST00000663056.1	1.9175	7.9493	3.183617	0.021169	0.160651	-3.36694
lnc-AKR1B1-3:2	-1.18204	6.457705	-3.18277	0.021191	0.160681	-3.36801
MSTRG.51547.1	1.433267	2.41081	3.182373	0.021201	0.160681	-3.36852
NONHSAT223779.1	1.107357	3.690804	3.181653	0.021219	0.160708	-3.36944
lnc-CDH12-8:1	1.980318	3.565326	3.181569	0.021221	0.160708	-3.36954
lnc-CDK17-12:1	-1.15338	2.175584	-3.18127	0.021229	0.16074	-3.36993
NONHSAT206189.1	1.089425	3.966765	3.181137	0.021232	0.16074	-3.37009
lnc-LRIG1-5:1	1.334271	4.711417	3.181067	0.021234	0.16074	-3.37018
NONHSAT216824.1	-1.11882	1.796204	-3.17937	0.021277	0.160946	-3.37234
NONHSAT187409.1	1.200633	4.958334	3.178252	0.021306	0.161053	-3.37377
NONHSAT189010.1	1.235337	2.071222	3.177951	0.021314	0.161053	-3.37415
MSTRG.50367.1	1.260347	3.224579	3.177882	0.021315	0.161053	-3.37424
MSTRG.22862.1	-1.43316	2.943658	-3.17782	0.021317	0.161054	-3.37432
lnc-SUMO1-2:3	-1.23846	1.76988	-3.17763	0.021322	0.161067	-3.37456
lnc-TMSB15A-6:1	-1.01327	2.823289	-3.17752	0.021325	0.161076	-3.3747
ENST00000505408.1	1.044948	3.512943	3.175877	0.021367	0.161309	-3.37679
lnc-EXD2-9:1	-1.00528	3.274848	-3.17547	0.021377	0.161337	-3.37731
MSTRG.21431.1	-1.20457	3.022621	-3.17531	0.021381	0.161337	-3.37751
lnc-SLC6A15-2:1	1.827052	2.547255	3.175229	0.021384	0.161337	-3.37761
NONHSAT156399.1	1.185481	3.789512	3.174768	0.021395	0.161353	-3.3782
ENST00000562917.1	1.223236	2.432388	3.1747	0.021397	0.161353	-3.37829
lnc-EFCAB14-1:2	-1.00611	3.755999	-3.17456	0.021401	0.161353	-3.37847
lnc-MTRNR2L13-11:1	-1.14418	2.095025	-3.17428	0.021408	0.161353	-3.37882
MSTRG.23779.1	1.346796	2.635556	3.173859	0.021419	0.161411	-3.37936
NONHSAT189106.1	1.375091	3.483577	3.173772	0.021421	0.161415	-3.37947
lnc-SLC39A10-2:2	1.414063	2.74174	3.173511	0.021428	0.161438	-3.3798
MSTRG.21837.2	-1.33765	2.7391	-3.17347	0.021429	0.161438	-3.37985
NONHSAT198275.1	1.11858	1.727397	3.173215	0.021435	0.161462	-3.38018
ENST00000610044.1	1.420872	2.472105	3.17316	0.021437	0.161462	-3.38025
NONHSAT169943.1	1.109634	7.054066	3.172401	0.021456	0.161513	-3.38121
NONHSAT218017.1	1.116177	2.631019	3.172401	0.021456	0.161513	-3.38121
MSTRG.54202.1	1.403682	1.77503	3.171921	0.021469	0.161551	-3.38183
NONHSAT221999.1	2.067108	3.620861	3.170963	0.021493	0.161596	-3.38305
ENST00000636210.1	1.599964	6.454199	3.169856	0.021522	0.161622	-3.38446
NONHSAT160749.1	-1.18258	2.474563	-3.16831	0.021562	0.161778	-3.38643

NONHSAT213998.1	1.015035	1.876943	3.168145	0.021566	0.161796	-3.38663
lnc-ZEB1-6:1	-1.02324	4.825147	-3.16797	0.021571	0.161796	-3.38686
NONHSAT191699.1	1.04164	4.741301	3.167648	0.021579	0.161811	-3.38727
ENST00000565668.2	-1.30605	4.229228	-3.16668	0.021604	0.16192	-3.3885
NONHSAT194280.1	1.750888	3.225471	3.165971	0.021623	0.161993	-3.38941
NONHSAT175007.1	1.298171	3.995867	3.165955	0.021623	0.161993	-3.38943
NONHSAT190210.1	1.732419	5.256474	3.164522	0.021661	0.162143	-3.39125
NONHSAT201234.1	1.103239	1.746158	3.164279	0.021667	0.162155	-3.39156
lnc-SMIM14-4:1	1.158834	7.030053	3.163263	0.021694	0.162254	-3.39286
NONHSAT154813.1	1.247207	3.548525	3.163131	0.021697	0.162254	-3.39303
lnc-PDE10A-1:4	1.076988	7.03209	3.162378	0.021717	0.162308	-3.39398
ENST00000441348.1	-1.01291	2.698359	-3.16186	0.02173	0.162349	-3.39464
NONHSAT223893.1	-1.31459	2.575718	-3.16152	0.021739	0.162392	-3.39508
NONHSAT224455.1	1.156775	4.110161	3.159707	0.021787	0.162542	-3.39739
MSTRG.16859.1	1.383459	2.02712	3.159622	0.021789	0.162542	-3.3975
NONHSAT187765.1	1.520027	3.103482	3.159248	0.021799	0.16256	-3.39798
MSTRG.60246.1	1.857072	2.069875	3.159108	0.021802	0.16256	-3.39816
lnc-DAP-14:1	1.462405	2.782221	3.158899	0.021808	0.16256	-3.39842
lnc-NCOA3-13:1	1.486747	2.655823	3.158881	0.021808	0.16256	-3.39844
lnc-NUP214-2:1	-1.31937	2.816962	-3.15764	0.021841	0.162621	-3.40002
LINC01422:4	1.143654	1.871923	3.157299	0.02185	0.162654	-3.40046
NONHSAT186868.1	1.013389	4.468043	3.157181	0.021853	0.162665	-3.40061
MSTRG.47804.1	-1.04491	4.784813	-3.15683	0.021862	0.162702	-3.40106
ENST00000654498.1	2.256528	2.148158	3.156176	0.021879	0.162802	-3.40189
NONHSAT217255.1	1.461327	2.25143	3.153856	0.021941	0.163042	-3.40486
lnc-FGF16-1:1	-1.52664	4.149932	-3.15366	0.021946	0.163067	-3.4051
lnc-CTNNB1-4:1	1.059988	1.867254	3.152828	0.021968	0.163115	-3.40617
lnc-PAXIP1-6:1	-1.12957	3.211089	-3.15199	0.02199	0.163216	-3.40724
ENST00000668059.1	1.284133	2.143297	3.151127	0.022013	0.163313	-3.40834
NONHSAT188127.1	1.329326	2.159672	3.150745	0.022023	0.163365	-3.40882
lnc-PRTG-1:1	-1.00217	3.010025	-3.1499	0.022046	0.163458	-3.4099
ENST00000574365.1	-1.37933	1.915983	-3.1497	0.022051	0.163475	-3.41016
ENST00000655724.1	-1.11735	1.636821	-3.14937	0.02206	0.163488	-3.41058
MSTRG.54157.1	-1.29262	3.121324	-3.14928	0.022062	0.163488	-3.41069
MSTRG.51163.1	-1.24019	2.200019	-3.14899	0.02207	0.163519	-3.41107
lnc-TSPYL5-4:1	1.036688	3.540604	3.148364	0.022086	0.163606	-3.41187
NONHSAT153665.1	1.317033	2.137388	3.147033	0.022122	0.163724	-3.41357
lnc-SCGB2B2-10:2	1.307486	4.67791	3.146987	0.022123	0.163724	-3.41362
NONHSAT196574.1	1.447913	2.37672	3.146636	0.022133	0.163761	-3.41407
NONHSAT207605.1	1.7978	2.821588	3.145767	0.022156	0.163894	-3.41518
NONHSAT179213.1	1.125917	1.686488	3.145666	0.022158	0.163894	-3.41531
MSTRG.63714.1	1.053795	3.232717	3.1453	0.022168	0.163945	-3.41578
T172511	1.199185	2.77623	3.144218	0.022197	0.164052	-3.41716
NONHSAT173381.1	1.180213	4.265466	3.143367	0.02222	0.164101	-3.41825
ENST00000668106.1	1.264604	1.655263	3.142869	0.022233	0.164163	-3.41888
lnc-ALDH7A1-3:1	1.613349	2.438842	3.142506	0.022243	0.164186	-3.41935
lnc-RBM26-3:1	1.577962	3.939739	3.141864	0.02226	0.164218	-3.42017
ENST00000655341.1	1.397844	1.762411	3.141765	0.022263	0.164226	-3.42029

lnc-TLE3-15:3	-1.06375	2.022525	-3.14164	0.022266	0.164238	-3.42045
lnc-MYO10-18:1	1.017391	5.136201	3.141132	0.02228	0.164303	-3.4211
lnc-PLEKHM1-10:1	-1.28779	1.774111	-3.1408	0.022289	0.164345	-3.42153
lnc-FAM103A1-3:1	2.53998	4.198452	3.139935	0.022312	0.164457	-3.42263
ENST00000443897.1	1.175373	1.705961	3.139784	0.022316	0.164473	-3.42282
ENST00000520762.1	1.811109	3.75294	3.139433	0.022326	0.164484	-3.42327
MSTRG.38357.1	1.076456	3.532669	3.139128	0.022334	0.164497	-3.42366
MSTRG.29597.1	-1.08284	2.456994	-3.13892	0.022339	0.164505	-3.42392
lnc-EFCAB2-7:1	1.311261	2.272797	3.138689	0.022346	0.164533	-3.42423
T086049	1.404552	3.16159	3.138176	0.02236	0.164568	-3.42488
MSTRG.17818.1	1.886391	2.657383	3.138167	0.02236	0.164568	-3.42489
NONHSAT153651.1	1.803516	3.246494	3.137809	0.02237	0.164615	-3.42535
MSTRG.28891.1	-1.08279	2.052148	-3.13688	0.022395	0.164752	-3.42654
lnc-TRIM13-3:1	1.499698	3.302186	3.136709	0.022399	0.164765	-3.42676
lnc-OR4C13-7:3	1.302295	2.344056	3.136647	0.022401	0.164765	-3.42684
lnc-DUSP26-6:1	-1.63844	3.176028	-3.13651	0.022405	0.164765	-3.42701
NONHSAT222020.1	1.310159	2.225376	3.136289	0.022411	0.164767	-3.42729
lnc-POTED-6:1	1.618273	2.469812	3.136257	0.022412	0.164767	-3.42733
MSTRG.52316.1	1.386876	3.340053	3.135652	0.022428	0.164851	-3.42811
MSTRG.21439.1	1.052047	3.508446	3.135557	0.022431	0.164851	-3.42823
lnc-LMBR1-11:2	-1.49701	2.51643	-3.13543	0.022434	0.164851	-3.42839
MSTRG.59591.1	1.286641	3.593535	3.135374	0.022435	0.164851	-3.42846
NONHSAT155757.1	1.082065	2.050149	3.134866	0.022449	0.164876	-3.42911
MSTRG.29096.1	-1.16176	2.856658	-3.13397	0.022474	0.164919	-3.43025
lnc-NCAM2-7:2	1.751496	2.078775	3.133897	0.022476	0.164919	-3.43035
lnc-MAP1LC3B2-14:26	1.157215	1.999795	3.133733	0.02248	0.164919	-3.43056
MSTRG.16275.1	1.493045	5.699968	3.133605	0.022483	0.164919	-3.43072
ENST00000457548.1	1.440985	3.37188	3.133605	0.022483	0.164919	-3.43072
ENST00000655836.1	1.723616	2.478806	3.133327	0.022491	0.164955	-3.43108
lnc-ACSL1-4:1	1.620174	1.914487	3.13283	0.022505	0.165018	-3.43172
MSTRG.14113.1	1.5505	3.782006	3.131745	0.022534	0.165151	-3.4331
NONHSAT224443.1	1.074724	2.127226	3.131739	0.022534	0.165151	-3.43311
NONHSAT167792.1	1.543815	2.520982	3.131321	0.022546	0.165199	-3.43365
ENST00000619407.4	-1.02704	2.958104	-3.13094	0.022556	0.165238	-3.43413
MSTRG.29398.1	1.616219	2.471649	3.130737	0.022562	0.165238	-3.43439
lnc-MRPL49-1:1	1.775178	3.152689	3.129854	0.022586	0.165304	-3.43552
NONHSAT175662.1	1.392979	1.815016	3.129787	0.022587	0.165304	-3.43561
ENST00000596207.2	-1.32566	2.300884	-3.12957	0.022593	0.165304	-3.43588
ENST00000564471.1	-1.21302	3.207376	-3.12941	0.022598	0.165304	-3.43609
lnc-JADE1-4:1	1.515503	2.908917	3.128701	0.022617	0.165374	-3.437
ENST00000508031.1	-1.33204	1.824968	-3.12829	0.022628	0.165397	-3.43753
lnc-XRCC2-12:2	1.518685	2.784772	3.127565	0.022648	0.16551	-3.43845
ENST00000445097.2	1.131254	1.742977	3.126909	0.022666	0.165523	-3.43929
NONHSAT185783.1	-1.31598	2.572926	-3.12691	0.022666	0.165523	-3.43929
lnc-MFSD9-9:1	1.702614	2.574809	3.126288	0.022683	0.165553	-3.44009
lnc-ORMDL2-4:1	-1.04866	2.792917	-3.1262	0.022686	0.165553	-3.4402
lnc-MANEA-5:2	1.614131	2.408906	3.126128	0.022688	0.165553	-3.44029
NONHSAT187214.1	1.125393	3.9841	3.126001	0.022691	0.165555	-3.44045

NONHSAT214556.1	1.590172	3.054741	3.12588	0.022694	0.165555	-3.44061
MSTRG.28644.42	-1.27517	2.592495	-3.12536	0.022709	0.165629	-3.44128
T036823	1.081819	1.582243	3.125088	0.022716	0.165642	-3.44162
lnc-IRS4-1:1	1.689992	2.775763	3.124369	0.022736	0.165762	-3.44254
lnc-HSP90B1-4:1	-1.13224	3.009553	-3.12366	0.022755	0.165844	-3.44345
ENST00000418420.1	1.104713	3.99348	3.12343	0.022762	0.165858	-3.44374
NONHSAT150490.1	1.485106	2.371702	3.123399	0.022763	0.165858	-3.44378
lnc-ATP11B-6:1	-1.10943	3.710052	-3.12331	0.022765	0.165858	-3.4439
lnc-RASGRF1-3:1	1.362279	4.203164	3.123295	0.022766	0.165858	-3.44392
NONHSAT179086.1	1.795466	2.975587	3.12294	0.022775	0.165872	-3.44437
NONHSAT180660.1	-1.03411	5.047088	-3.12279	0.022779	0.165872	-3.44456
lnc-NXPH1-3:1	-1.04776	1.979666	-3.12277	0.02278	0.165872	-3.44459
MSTRG.11972.1	1.402558	2.247898	3.122658	0.022783	0.165878	-3.44473
MSTRG.20414.1	1.799922	2.568657	3.122495	0.022788	0.165886	-3.44494
lnc-FZD4-9:1	-1.52997	2.829969	-3.12226	0.022794	0.16591	-3.44524
lnc-TMEM50B-7:4	1.175708	4.24269	3.122126	0.022798	0.165919	-3.44541
NONHSAT210905.1	1.0221	4.008563	3.12196	0.022802	0.165919	-3.44563
lnc-GDPD4-3:3	1.181683	2.090025	3.121781	0.022807	0.165934	-3.44585
NONHSAT153416.1	1.425776	7.561436	3.121546	0.022814	0.165949	-3.44616
NONHSAT162894.1	1.213944	1.985974	3.121069	0.022827	0.165993	-3.44677
lnc-GPR89B-11:2	1.861343	2.32038	3.120698	0.022837	0.16602	-3.44724
lnc-SCRN3-7:1	-1.01032	2.191622	-3.11939	0.022873	0.166162	-3.44891
NONHSAT215876.1	1.171357	2.990076	3.118853	0.022888	0.166199	-3.4496
ENST00000669934.1	1.716851	2.539485	3.11876	0.022891	0.166199	-3.44972
lnc-ECSCR-3:1	-1.13348	2.646901	-3.11855	0.022897	0.166199	-3.45
NONHSAT157933.1	-1.09819	2.631317	-3.11847	0.022899	0.166199	-3.45009
ENST00000567598.1	1.240029	7.136107	3.118263	0.022905	0.166199	-3.45036
lnc-TERF1-1:2	-1.15014	3.135843	-3.11818	0.022907	0.166199	-3.45047
NONHSAT221393.1	1.897991	2.298044	3.118118	0.022909	0.166199	-3.45055
lnc-TRAF3IP2-4:1	-1.51547	3.617109	-3.11716	0.022935	0.166247	-3.45177
MSTRG.45426.1	-1.3182	2.550063	-3.11704	0.022939	0.166247	-3.45193
lnc-VTI1A-3:1	-1.68494	2.924452	-3.11694	0.022941	0.166247	-3.45205
lnc-ALAS2-2:1	-1.45518	3.215871	-3.11596	0.022968	0.166292	-3.45331
NONHSAT212429.1	-1.15494	4.53062	-3.11587	0.022971	0.166292	-3.45343
ENST00000422609.1	1.500361	2.697191	3.115704	0.022976	0.166292	-3.45364
NONHSAT169556.1	-1.30296	3.660424	-3.11563	0.022978	0.166292	-3.45373
T305477	-1.07724	2.601445	-3.11561	0.022978	0.166292	-3.45376
MSTRG.72280.1	1.121831	2.16977	3.113959	0.023024	0.166468	-3.45587
lnc-LRGUK-5:1	-1.29893	3.814562	-3.11278	0.023057	0.166598	-3.45739
NONHSAT188147.1	1.461057	2.272946	3.112589	0.023063	0.166608	-3.45763
NONHSAT213978.1	-1.40876	3.888285	-3.11257	0.023063	0.166608	-3.45766
lnc-ESM1-7:1	1.488164	3.852404	3.111817	0.023084	0.166697	-3.45862
NONHSAT206006.1	1.504061	1.859526	3.111581	0.023091	0.166707	-3.45892
lnc-NMI-5:1	1.130668	4.918059	3.111255	0.0231	0.166707	-3.45934
lnc-KLHL1-7:1	1.415029	3.346915	3.11096	0.023108	0.166707	-3.45972
T223724	-1.36065	2.521823	-3.11093	0.023109	0.166707	-3.45976
NONHSAT158302.1	1.072623	1.682977	3.110685	0.023116	0.166715	-3.46007
NONHSAT174565.1	1.045827	2.212903	3.110665	0.023116	0.166715	-3.4601

lnc-AUH-2:9	1.119537	3.911792	3.110498	0.023121	0.166737	-3.46031
lnc-TRIM49B-1:1	1.328671	2.236949	3.109362	0.023153	0.166871	-3.46177
ENST00000657788.1	-1.56846	2.611005	-3.10907	0.023161	0.166893	-3.46214
NONHSAT189214.1	1.686391	3.399006	3.108951	0.023164	0.166893	-3.46229
NONHSAT208046.1	1.047175	2.01057	3.108844	0.023167	0.166893	-3.46243
MSTRG.63755.1	1.291726	2.27055	3.108044	0.02319	0.166978	-3.46346
NONHSAT187168.1	1.031754	4.172279	3.108017	0.023191	0.166978	-3.46349
lnc-STK26-6:1	1.235496	4.347872	3.107954	0.023192	0.166978	-3.46357
lnc-CDH26-2:1	1.341895	1.932326	3.107488	0.023206	0.167024	-3.46417
ENST00000660073.1	1.194577	1.727555	3.107358	0.023209	0.167037	-3.46434
NONHSAT206905.1	1.215431	2.544701	3.107245	0.023212	0.167037	-3.46448
ENST00000443744.2	-1.39785	3.827515	-3.10669	0.023228	0.1671	-3.46519
NONHSAT223531.1	-1.32972	2.123199	-3.10664	0.023229	0.1671	-3.46526
lnc-SLITRK6-13:1	1.306523	2.524927	3.10634	0.023238	0.167149	-3.46564
NONHSAT208493.1	1.126685	1.644486	3.105752	0.023254	0.167232	-3.4664
NONHSAT211111.1	1.316116	2.217907	3.105339	0.023266	0.167292	-3.46693
NONHSAT217573.1	-1.05203	2.523657	-3.10488	0.023279	0.167292	-3.46751
lnc-KLF4-2:1	-1.23702	2.54941	-3.10446	0.023291	0.167292	-3.46805
NONHSAT211139.1	1.003411	3.725492	3.103967	0.023305	0.167325	-3.46869
T068170	1.31245	2.238952	3.103172	0.023327	0.167413	-3.46971
MSTRG.20718.1	-1.49467	3.707585	-3.10296	0.023333	0.167426	-3.46997
lnc-WDR89-3:1	-1.34488	3.322445	-3.10282	0.023337	0.167426	-3.47016
NR_029429	-1.24486	3.18512	-3.10211	0.023357	0.16754	-3.47107
lnc-ANKRD20A3-7:1	1.112162	4.330424	3.100904	0.023392	0.16768	-3.47261
ENST00000552015.1	-1.20286	4.424809	-3.10033	0.023408	0.167772	-3.47335
NONHSAT186984.1	1.103729	2.798227	3.099627	0.023428	0.167833	-3.47425
NONHSAT171502.1	1.267872	2.313992	3.099335	0.023436	0.167855	-3.47463
MSTRG.40412.1	1.613541	2.499418	3.097684	0.023483	0.168122	-3.47675
MSTRG.67175.1	-1.25142	2.96138	-3.09732	0.023494	0.168154	-3.47722
lnc-PACRGL-10:1	1.306269	2.4558	3.096983	0.023503	0.168163	-3.47765
NONHSAT218753.1	1.687064	2.337849	3.096694	0.023511	0.168176	-3.47802
ENST00000609422.1	-1.35839	2.221387	-3.09624	0.023524	0.168208	-3.4786
NONHSAT210660.1	1.730529	3.100968	3.096065	0.023529	0.168214	-3.47883
NONHSAT177274.1	1.05205	4.529681	3.095886	0.023534	0.168232	-3.47906
NONHSAT185100.1	1.005955	1.869138	3.095441	0.023547	0.168242	-3.47963
MSTRG.33075.1	1.321395	2.20639	3.095423	0.023548	0.168242	-3.47965
lnc-RAB6C-9:1	-1.37517	2.596562	-3.09539	0.023549	0.168242	-3.47969
NONHSAT168011.1	1.223481	8.961171	3.095132	0.023556	0.16826	-3.48002
lnc-ABHD17C-1:1	1.028949	1.914381	3.094546	0.023573	0.168281	-3.48078
NONHSAT194908.1	1.67752	2.527507	3.093889	0.023592	0.168336	-3.48162
NONHSAT154834.1	-1.11294	4.411064	-3.09285	0.023621	0.168429	-3.48295
ENST00000564492.1	1.598974	2.866732	3.092435	0.023633	0.168459	-3.48349
NONHSAT183014.1	1.204308	3.219451	3.092301	0.023637	0.168459	-3.48366
lnc-NUDT21-1:1	-1.39101	2.497906	-3.09219	0.02364	0.168469	-3.4838
MSTRG.789.1	1.555535	5.953189	3.091808	0.023651	0.168524	-3.48429
NONHSAT166031.1	-1.05058	2.193055	-3.09167	0.023655	0.16854	-3.48447
NONHSAT170664.1	1.927784	4.182325	3.091485	0.023661	0.168554	-3.48471
NONHSAT156183.1	1.210244	3.690146	3.090733	0.023682	0.168608	-3.48567

NONHSAT198813.1	1.576589	2.960932	3.090627	0.023685	0.168608	-3.48581
lnc-LONRF1-7:1	1.146141	2.215677	3.08938	0.023721	0.168736	-3.48741
lnc-SPOPL-8:1	-1.28594	1.747545	-3.0892	0.023726	0.168736	-3.48764
ENST00000606818.1	1.492314	2.780074	3.088796	0.023738	0.168748	-3.48816
MSTRG.58046.1	1.440775	2.514016	3.088227	0.023754	0.168841	-3.48889
MSTRG.9267.1	-1.38377	2.939961	-3.08652	0.023804	0.169029	-3.49108
lnc-SLC1A2-4:1	1.216462	2.739633	3.086441	0.023806	0.169029	-3.49119
lnc-OTP-2:1	-1.02867	5.208224	-3.08576	0.023826	0.169084	-3.49206
ENST00000661594.1	1.209513	2.446621	3.085654	0.023829	0.169084	-3.4922
lnc-COL11A1-3:1	1.127294	1.641821	3.085276	0.02384	0.169098	-3.49269
lnc-SBNO1-2:1	1.609751	4.33362	3.085192	0.023842	0.169098	-3.49279
LINC01687:15	1.068808	4.609377	3.084798	0.023854	0.169129	-3.4933
lnc-SBDS-23:1	-1.05951	2.535469	-3.08446	0.023864	0.169176	-3.49374
NONHSAT189680.1	-1.08209	3.602761	-3.08333	0.023896	0.169312	-3.49518
NONHSAT153641.1	1.031086	3.197539	3.083317	0.023897	0.169312	-3.4952
lnc-TMED5-5:1	-1.6126	2.316217	-3.08319	0.0239	0.169312	-3.49537
MSTRG.34072.1	-1.18051	2.397407	-3.08313	0.023902	0.169312	-3.49545
lnc-TMEM98-1:5	-1.06904	2.054362	-3.08285	0.02391	0.169312	-3.49581
lnc-ITGA5-1:1	-1.25704	3.203269	-3.08205	0.023933	0.169422	-3.49683
NONHSAT177883.1	1.328389	5.472529	3.08172	0.023943	0.169458	-3.49726
NONHSAT161671.1	-1.39975	2.769743	-3.08144	0.023951	0.169463	-3.49762
NONHSAT168915.1	1.839078	3.526517	3.081418	0.023952	0.169463	-3.49765
lnc-BRF1-50:1	1.64275	2.221192	3.07953	0.024007	0.169691	-3.50007
lnc-ARHGEF7-6:1	1.297901	2.865213	3.079364	0.024012	0.169699	-3.50029
NONHSAT202213.1	-1.33664	2.937043	-3.07932	0.024013	0.169699	-3.50035
NONHSAT223781.1	1.196963	2.275856	3.078242	0.024045	0.169779	-3.50173
NONHSAT193204.1	1.539877	2.425588	3.078124	0.024048	0.169779	-3.50188
NONHSAT174522.1	2.12133	3.72321	3.077609	0.024063	0.169825	-3.50254
NONHSAT207683.1	1.366938	2.229127	3.076162	0.024105	0.169971	-3.5044
MSTRG.66537.1	-1.72456	3.022176	-3.07571	0.024119	0.169991	-3.50499
ENST00000502668.2	1.853611	5.968382	3.075488	0.024125	0.170005	-3.50527
NONHSAT171291.1	1.323697	1.74871	3.075235	0.024133	0.170032	-3.5056
MSTRG.34440.1	1.541017	2.955001	3.075106	0.024136	0.170047	-3.50576
NONHSAT205027.1	1.409451	2.224716	3.074807	0.024145	0.17008	-3.50615
NONHSAT222335.1	-1.41091	6.999551	-3.07379	0.024175	0.170184	-3.50745
ENST00000597028.1	-1.19254	3.060864	-3.07371	0.024177	0.170184	-3.50755
MSTRG.33022.1	-1.93563	2.657408	-3.07178	0.024234	0.170351	-3.51004
MSTRG.51151.6	1.494269	1.846647	3.07166	0.024238	0.170351	-3.5102
NONHSAT181341.1	1.488256	2.846501	3.071656	0.024238	0.170351	-3.5102
NONHSAT166019.1	1.193658	2.139751	3.071527	0.024242	0.170351	-3.51037
ENST00000671082.1	1.052024	2.071653	3.071464	0.024244	0.170351	-3.51045
T132814	1.243388	2.210424	3.070925	0.02426	0.170393	-3.51114
lnc-FBXW11-8:1	1.711506	7.261239	3.070574	0.02427	0.170407	-3.5116
lnc-ARAP2-11:1	1.374077	3.996505	3.070399	0.024275	0.170417	-3.51182
lnc-DENND3-7:1	1.030074	4.674121	3.069843	0.024292	0.17048	-3.51254
NBAT1:11	-1.45356	2.702295	-3.06963	0.024298	0.170493	-3.51281
ENST00000523121.1	1.230453	2.044871	3.069383	0.024305	0.170504	-3.51313
lnc-MAX-8:1	1.088569	1.603085	3.069291	0.024308	0.170504	-3.51325

lnc-TAB3-2:1	1.213671	2.00713	3.067375	0.024365	0.170694	-3.51571
NONHSAT170325.1	1.748412	3.069796	3.067361	0.024365	0.170694	-3.51573
ENST00000668987.1	1.002144	9.089447	3.067172	0.024371	0.170694	-3.51598
NONHSAT216194.1	1.622761	3.100545	3.067164	0.024371	0.170694	-3.51599
MSTRG.48593.1	-1.44361	2.245571	-3.06673	0.024384	0.170705	-3.51654
NONHSAT158414.1	1.245606	3.445928	3.066413	0.024393	0.170747	-3.51695
lnc-DNASE1L3-3:1	-2.19665	3.648014	-3.06605	0.024404	0.1708	-3.51743
ENST00000420865.6	1.824034	1.971183	3.06526	0.024428	0.170928	-3.51844
MSTRG.54168.1	1.550583	2.817459	3.064354	0.024455	0.170989	-3.5196
MSTRG.39770.1	1.595028	2.349149	3.064141	0.024461	0.170989	-3.51988
MSTRG.70427.1	-1.16042	4.297193	-3.06397	0.024466	0.170989	-3.5201
NONHSAT181208.1	1.260499	1.737927	3.063954	0.024467	0.170989	-3.52012
lnc-IGSF10-8:1	-1.08295	3.578236	-3.06395	0.024467	0.170989	-3.52012
LINC02384:41	1.237065	2.455333	3.06395	0.024467	0.170989	-3.52013
lnc-BCL2L15-2:1	-1.09089	3.860538	-3.06388	0.024469	0.170989	-3.52021
ENST00000657182.1	1.161513	1.615004	3.063535	0.024479	0.171022	-3.52066
NONHSAT206808.1	-1.04865	2.200049	-3.06349	0.02448	0.171022	-3.52071
NONHSAT197711.1	-1.21471	2.801498	-3.06334	0.024485	0.171022	-3.5209
lnc-OCA2-6:1	1.918021	2.602051	3.063133	0.024491	0.171027	-3.52118
lnc-C1orf115-1:1	-1.23694	2.37157	-3.06279	0.024501	0.17104	-3.52162
ENST00000519550.1	1.486155	2.106427	3.062242	0.024518	0.171116	-3.52232
T049910	1.476998	1.907118	3.061541	0.024539	0.171205	-3.52323
lnc-BMP6-16:1	1.06234	1.99606	3.061378	0.024544	0.171227	-3.52344
ENST00000449895.5	-1.03315	2.37342	-3.06038	0.024573	0.171376	-3.52472
NONHSAT164553.1	1.218124	2.066695	3.059544	0.024598	0.171487	-3.5258
lnc-OR10X1-1:1	1.187041	2.536789	3.059534	0.024599	0.171487	-3.52581
lnc-PDK3-8:1	-1.44686	3.438161	-3.05923	0.024608	0.171519	-3.5262
lnc-SLF1-8:1	1.242241	3.646426	3.058082	0.024642	0.171612	-3.52769
NONHSAT218556.1	1.386581	2.212304	3.057613	0.024656	0.171659	-3.52829
NONHSAT210756.1	-1.04091	2.737375	-3.05714	0.024671	0.17169	-3.5289
NONHSAT192059.1	1.554436	2.042817	3.057044	0.024674	0.17169	-3.52902
lnc-LHFPL2-2:1	-1.37202	2.30302	-3.05702	0.024674	0.17169	-3.52906
T210151	1.054923	3.42767	3.056817	0.02468	0.171695	-3.52932
NR_111906	-1.02501	3.069734	-3.05659	0.024687	0.171711	-3.52961
NONHSAT198524.1	1.187797	2.765274	3.056146	0.024701	0.171753	-3.53018
MSTRG.28361.3	1.385791	2.404671	3.055878	0.024709	0.171786	-3.53053
NONHSAT220074.1	-1.89417	2.542113	-3.05587	0.024709	0.171786	-3.53053
lnc-CNOT8-1:1	1.178156	2.73109	3.055788	0.024711	0.171793	-3.53064
ENST00000648961.1	1.403204	3.379364	3.054606	0.024747	0.171945	-3.53217
T279692	1.080562	2.096715	3.054281	0.024757	0.171945	-3.53259
NONHSAT151124.1	1.706017	1.950779	3.054193	0.02476	0.171945	-3.5327
NONHSAT222110.1	1.438554	2.755587	3.053814	0.024771	0.171989	-3.53319
lnc-ITGB3BP-11:1	-1.27824	3.790414	-3.05367	0.024775	0.171989	-3.53337
ENST00000659117.1	-1.55822	4.037767	-3.05312	0.024792	0.172033	-3.53408
ENST00000654363.1	1.493998	8.65842	3.053018	0.024795	0.172042	-3.53421
NONHSAT149190.1	1.328803	2.714132	3.052767	0.024803	0.172065	-3.53454
ENST00000648756.1	1.015937	11.52133	3.05251	0.024811	0.17209	-3.53487
NONHSAT205229.1	1.421252	2.398038	3.052227	0.024819	0.17209	-3.53523

MSTRG.61489.1	-1.35597	2.010644	-3.05217	0.024821	0.17209	-3.53531
lnc-NBN-5:1	-1.06367	3.264627	-3.05154	0.02484	0.172171	-3.53612
NONHSAT170347.1	1.022826	4.204656	3.051504	0.024841	0.172171	-3.53617
lnc-TMEM43-3:2	1.532088	3.350475	3.050676	0.024866	0.172309	-3.53723
NONHSAT167717.1	-1.2769	1.75597	-3.05007	0.024885	0.172346	-3.53802
lnc-TMC6-1:1	1.979166	2.925946	3.049566	0.0249	0.172389	-3.53867
NONHSAT162359.1	1.621142	2.924488	3.04928	0.024909	0.172414	-3.53903
NONHSAT172479.1	1.309314	3.759176	3.049103	0.024914	0.172439	-3.53926
lnc-GPD2-4:1	1.003405	4.18224	3.04893	0.024919	0.172445	-3.53949
NONHSAT171706.1	-1.27439	3.230306	-3.0487	0.024926	0.172453	-3.53978
MSTRG.29681.1	1.104129	1.917134	3.04734	0.024968	0.172617	-3.54154
NONHSAT202366.1	-1.31723	2.618848	-3.04613	0.025005	0.172757	-3.5431
lnc-MBTPS2-1:1	1.093614	4.780834	3.044718	0.025048	0.172962	-3.54492
lnc-GOLGA4-3:2	-1.21164	2.235743	-3.04418	0.025064	0.173004	-3.54561
NONHSAT160999.1	1.462677	2.355636	3.042946	0.025102	0.173135	-3.54721
NONHSAT192529.1	1.68046	2.947355	3.04243	0.025118	0.17322	-3.54787
MSTRG.60180.12	-1.01117	4.020636	-3.04211	0.025128	0.173265	-3.54829
NONHSAT170418.1	1.346324	1.719585	3.04204	0.02513	0.173268	-3.54838
ENST00000664362.1	1.667465	3.22797	3.041328	0.025152	0.173366	-3.5493
MSTRG.45322.1	1.256401	6.273185	3.041042	0.02516	0.173403	-3.54967
lnc-OFD1-3:1	1.054532	1.583329	3.040394	0.02518	0.173474	-3.5505
MSTRG.4614.16	-1.33703	2.705063	-3.04004	0.025191	0.173497	-3.55096
ENST00000649888.1	1.229366	1.976703	3.040009	0.025192	0.173497	-3.551
lnc-MPP2-1:3	1.43187	2.27989	3.039803	0.025199	0.173505	-3.55127
NONHSAT178822.1	1.128344	2.034086	3.039645	0.025203	0.173514	-3.55147
NONHSAT167482.1	1.35022	4.12745	3.039476	0.025209	0.173528	-3.55169
MSTRG.34306.1	1.913406	2.924527	3.039358	0.025212	0.17354	-3.55184
NONHSAT218810.1	1.796986	2.002266	3.037602	0.025267	0.173699	-3.55411
NONHSAT157725.1	1.483912	2.644384	3.03734	0.025275	0.173743	-3.55445
lnc-PLEKHH2-5:2	-1.21397	3.387115	-3.03655	0.025299	0.173853	-3.55546
lnc-NTF3-4:1	1.319971	2.73644	3.036545	0.025299	0.173853	-3.55547
NONHSAT210302.1	-1.08038	4.281831	-3.03642	0.025303	0.173868	-3.55564
lnc-LIPG-6:1	-1.07856	5.157346	-3.03577	0.025323	0.173936	-3.55648
lnc-GGN-4:1	-1.99156	3.965871	-3.03407	0.025376	0.174182	-3.55867
NONHSAT156088.1	1.184842	2.583888	3.034064	0.025376	0.174182	-3.55868
NONHSAT162008.1	1.634284	2.847281	3.03367	0.025388	0.174215	-3.55919
lnc-PPP1R3D-8:1	-1.38583	2.366883	-3.03312	0.025405	0.174278	-3.5599
NONHSAT219928.1	1.526773	2.306055	3.032968	0.02541	0.174278	-3.56009
ENST00000458063.1	1.218505	3.978971	3.031716	0.025449	0.174398	-3.56171
lnc-GGPS1-15:2	-1.34733	2.542434	-3.0317	0.025449	0.174398	-3.56173
lnc-INHBB-2:1	1.096003	3.060453	3.031573	0.025454	0.174414	-3.5619
NONHSAT161257.1	-1.09472	3.349617	-3.02986	0.025507	0.174673	-3.56411
NONHSAT179780.1	1.513459	2.866981	3.029312	0.025524	0.174723	-3.56482
MSTRG.15587.1	1.101618	2.377288	3.02925	0.025526	0.174723	-3.5649
ENST00000667827.1	-1.47801	2.804118	-3.02924	0.025526	0.174723	-3.56491
T302771	-1.33556	3.4774	-3.0287	0.025543	0.174767	-3.56561
NONHSAT187216.1	-1.01381	3.08053	-3.02833	0.025555	0.174788	-3.56609
ENST00000420237.1	1.586572	2.965566	3.028004	0.025565	0.174816	-3.56651

NONHSAT222288.1	1.110247	1.847745	3.027944	0.025567	0.174816	-3.56659
NONHSAT222083.1	-1.06253	4.117361	-3.02753	0.02558	0.174848	-3.56712
MSTRG.1729.22	1.527298	3.936185	3.027045	0.025595	0.174914	-3.56775
LINC02348:14	1.11356	2.913065	3.026417	0.025615	0.174947	-3.56856
MSTRG.11905.1	1.235885	8.009674	3.026324	0.025618	0.174947	-3.56868
lnc-EBF3-15:1	2.083905	3.482411	3.02593	0.02563	0.174947	-3.56919
lnc-HIST1H2AH-2:1	1.463176	4.553558	3.025867	0.025632	0.174947	-3.56927
ENST00000658546.1	1.30779	1.897213	3.02393	0.025693	0.17518	-3.57178
NONHSAT173652.1	-1.14915	3.353714	-3.02377	0.025698	0.17518	-3.57198
NONHSAT169970.1	-1.20434	3.068288	-3.02362	0.025703	0.17518	-3.57218
MSTRG.9529.1	-1.08583	3.053496	-3.02179	0.02576	0.175357	-3.57454
T103473	-1.17441	3.247819	-3.02129	0.025776	0.175396	-3.57519
NONHSAT164702.1	1.024157	4.134621	3.020949	0.025787	0.175435	-3.57563
NONHSAT176788.1	1.76697	2.199643	3.020609	0.025798	0.175472	-3.57607
MSTRG.34927.1	-1.55927	2.362403	-3.02027	0.025808	0.175476	-3.5765
MSTRG.56306.1	-1.02343	4.048597	-3.02012	0.025813	0.175476	-3.5767
lnc-FECH-4:2	-1.1716	2.927747	-3.02003	0.025816	0.175476	-3.57682
NONHSAT167325.1	1.20538	2.813128	3.019752	0.025825	0.175476	-3.57718
MSTRG.39762.1	1.583726	3.293007	3.019711	0.025826	0.175476	-3.57723
NONHSAT197678.1	1.659884	7.54325	3.019603	0.025829	0.175487	-3.57737
MSTRG.36189.1	1.198762	3.688888	3.01914	0.025844	0.175563	-3.57797
lnc-ZNF727-3:1	1.024228	3.849541	3.018727	0.025857	0.175593	-3.57851
NONHSAT197131.1	1.635596	3.674596	3.018565	0.025862	0.175616	-3.57872
T254246	1.163222	4.240549	3.017778	0.025887	0.175746	-3.57973
NONHSAT218146.1	1.110886	3.487737	3.017269	0.025903	0.175812	-3.58039
lnc-CEACAM19-2:1	-1.35195	2.565823	-3.01701	0.025912	0.175818	-3.58072
MSTRG.38435.1	-1.4616	2.613108	-3.0158	0.02595	0.175914	-3.5823
NONHSAT174356.1	1.116827	3.885149	3.015043	0.025974	0.175974	-3.58327
NONHSAT201267.1	-1.52956	3.394014	-3.01497	0.025977	0.175974	-3.58337
NONHSAT154209.1	1.570996	2.931477	3.014278	0.025999	0.176051	-3.58426
lnc-DEGS1-2:1	-1.69265	3.711637	-3.01419	0.026001	0.176051	-3.58438
lnc-EML4-3:1	-1.15537	1.834301	-3.01298	0.02604	0.176194	-3.58594
NONHSAT221834.1	-1.26059	2.92305	-3.01264	0.026051	0.176217	-3.58639
T298308	-1.39337	2.635636	-3.0126	0.026052	0.176217	-3.58644
lnc-SF3B1-3:1	1.400234	2.103486	3.01197	0.026072	0.17627	-3.58725
NONHSAT160228.1	1.354404	5.39064	3.010423	0.026122	0.17644	-3.58925
lnc-FAM208B-1:1	1.716413	2.214569	3.010094	0.026132	0.176475	-3.58968
lnc-ALDH3A2-7:1	1.118176	5.358584	3.009364	0.026156	0.176559	-3.59063
NONHSAT158928.1	-1.11708	3.398991	-3.00788	0.026204	0.176752	-3.59255
lnc-KIAA2013-6:1	-1.23789	2.079741	-3.00755	0.026214	0.176789	-3.59297
MSTRG.64653.1	1.297304	4.616604	3.00673	0.02624	0.176867	-3.59404
MSTRG.34778.1	1.219893	1.894426	3.006601	0.026245	0.176867	-3.5942
ENST00000670865.1	1.384042	3.054782	3.00642	0.02625	0.176867	-3.59444
NONHSAT210656.1	1.776757	3.152668	3.006363	0.026252	0.176867	-3.59451
NONHSAT208397.1	1.341587	2.10455	3.006004	0.026264	0.176884	-3.59498
T119156	-1.01243	1.859929	-3.00591	0.026267	0.176884	-3.5951
lnc-CARHSP1-4:1	-1.09327	5.178346	-3.00591	0.026267	0.176884	-3.5951
ENST00000457890.1	1.112054	1.969769	3.005733	0.026273	0.176889	-3.59533

MSTRG.45998.1	1.418644	1.786455	3.005095	0.026293	0.176956	-3.59616
MSTRG.19827.14	1.049334	1.602802	3.004902	0.026299	0.176956	-3.59641
ENST00000548901.1	-1.07508	3.570539	-3.00486	0.026301	0.176956	-3.59646
ENST00000499765.2	-1.09751	4.789211	-3.00472	0.026305	0.176956	-3.59664
lnc-PPIAL4F-3:5	1.004739	3.252924	3.004143	0.026324	0.176973	-3.59739
ENST00000654979.1	-1.07585	5.163009	-3.00359	0.026342	0.177002	-3.59811
NONHSAT173590.1	1.233678	1.723665	3.002623	0.026373	0.17714	-3.59936
lnc-DAPK2-3:2	-1.13944	3.907547	-3.00231	0.026383	0.177184	-3.59976
NONHSAT175562.1	1.950567	2.53372	3.00196	0.026395	0.177215	-3.60022
ENST00000567851.5	-1.13892	3.739194	-3.00173	0.026402	0.177234	-3.60052
ENST00000653421.1	-1.27351	3.236048	-3.00154	0.026408	0.177247	-3.60077
lnc-ZNF521-3:1	-1.1424	2.281566	-3.00094	0.026428	0.177317	-3.60154
NONHSAT158629.1	1.287256	2.587785	3.000499	0.026442	0.17739	-3.60211
MSTRG.5095.1	1.597812	2.854435	2.999822	0.026464	0.177491	-3.60299
NONHSAT187552.1	1.778559	3.690381	2.999324	0.02648	0.177534	-3.60363
lnc-DRD1-2:1	-1.09452	2.918471	-2.99929	0.026481	0.177534	-3.60368
NONHSAT179288.1	2.212102	3.292664	2.998589	0.026504	0.177611	-3.60459
lnc-SH3GL3-3:1	-1.71371	2.461523	-2.99822	0.026516	0.177661	-3.60506
T285360	1.942258	3.357656	2.998122	0.026519	0.177661	-3.60519
lnc-RLF-2:1	1.086217	2.201031	2.997918	0.026526	0.177663	-3.60546
NONHSAT158876.1	1.478697	5.011047	2.997581	0.026537	0.177705	-3.60589
NONHSAT219183.1	-1.00169	1.851404	-2.99744	0.026542	0.177705	-3.60608
lnc-DHRS7-4:1	-1.23707	3.722726	-2.99727	0.026547	0.177716	-3.60629
NONHSAT179531.1	1.010858	7.534978	2.996598	0.026569	0.177805	-3.60717
lnc-KSR1-3:3	-1.03418	2.053167	-2.99612	0.026585	0.177887	-3.60779
ENST00000560647.5	-1.02305	2.007006	-2.9959	0.026592	0.177896	-3.60808
MSTRG.1520.1	1.050109	6.445113	2.993939	0.026656	0.178041	-3.61062
NONHSAT197169.1	1.60939	2.953919	2.993855	0.026659	0.178041	-3.61073
MSTRG.45987.1	-1.05361	1.727301	-2.9925	0.026703	0.178158	-3.61248
lnc-PPA2-5:1	-1.51535	3.516901	-2.99244	0.026705	0.178158	-3.61256
NONHSAT191693.1	1.220256	2.588479	2.992116	0.026716	0.17818	-3.61298
NONHSAT154670.1	-1.26639	3.488315	-2.99152	0.026735	0.178261	-3.61375
ENST00000464382.2	1.185245	2.381613	2.990596	0.026766	0.178314	-3.61495
lnc-DOCK7-8:1	1.533344	2.956988	2.988933	0.026821	0.178525	-3.61711
NONHSAT197775.1	1.490593	3.002366	2.988745	0.026827	0.178546	-3.61735
lnc-DNAH8-2:8	-1.13986	1.87064	-2.98856	0.026833	0.178559	-3.6176
lnc-RNF38-5:1	-1.39721	3.018868	-2.98803	0.026851	0.178613	-3.61829
NONHSAT174989.1	-1.53631	2.483515	-2.98801	0.026851	0.178613	-3.61831
NONHSAT215111.1	1.476868	2.203767	2.987159	0.026879	0.178718	-3.61941
ENST00000568150.3	-1.82031	3.331119	-2.98663	0.026897	0.178763	-3.62009
NONHSAT217760.1	1.274217	1.670395	2.986352	0.026906	0.178801	-3.62046
NONHSAT156532.1	-1.53601	3.026101	-2.98623	0.02691	0.178806	-3.62062
ENST00000429450.1	-1.25502	2.807842	-2.98596	0.026919	0.178852	-3.62097
NONHSAT152675.1	1.662986	2.508242	2.985266	0.026942	0.17892	-3.62187
ENST00000644636.1	1.174626	1.895231	2.985118	0.026947	0.17892	-3.62206
HLA-F-AS1:9	-1.1216	3.857195	-2.98483	0.026957	0.178927	-3.62244
ENST00000608316.1	-1.18508	2.960487	-2.98479	0.026958	0.178927	-3.62249
lnc-MID1-5:1	1.197799	1.987444	2.984571	0.026965	0.178927	-3.62277

NONHSAT223944.1	1.608554	2.977608	2.983971	0.026985	0.178972	-3.62355
NONHSAT217843.1	1.052607	3.662104	2.983367	0.027005	0.179035	-3.62433
NONHSAT186764.1	-1.4938	2.706726	-2.9831	0.027014	0.17907	-3.62468
NONHSAT156458.1	-1.1113	3.026657	-2.98297	0.027018	0.17908	-3.62484
MSTRG.65067.1	1.422601	2.306908	2.982664	0.027028	0.17908	-3.62525
NONHSAT222342.1	-1.12353	3.324956	-2.98248	0.027035	0.17908	-3.62549
NONHSAT168969.1	-1.31977	2.412356	-2.98217	0.027045	0.17908	-3.62589
lnc-PRDM2-2:1	1.272431	2.437037	2.982101	0.027047	0.17908	-3.62598
MSTRG.54468.1	1.664478	2.742653	2.981233	0.027076	0.17919	-3.62711
lnc-HIST1H2AA-4:1	1.387986	3.139323	2.980898	0.027087	0.179238	-3.62754
lnc-RCHY1-7:1	1.157624	2.711658	2.980717	0.027093	0.17925	-3.62778
NONHSAT199127.1	1.132278	3.437226	2.980684	0.027094	0.17925	-3.62782
NONHSAT172339.1	-1.04104	2.202249	-2.98049	0.027101	0.179268	-3.62807
lnc-ZNF100-8:1	1.355328	4.035406	2.979831	0.027123	0.179324	-3.62893
NONHSAT179044.1	1.222483	2.47385	2.979691	0.027127	0.179328	-3.62911
ENST00000660197.1	1.757609	2.430675	2.979224	0.027143	0.179384	-3.62971
NONHSAT219923.1	1.145626	3.932508	2.978969	0.027152	0.179394	-3.63005
NONHSAT218963.1	1.113932	3.544772	2.978456	0.027169	0.17946	-3.63071
lnc-DET1-1:19	1.995389	2.771013	2.97811	0.02718	0.179494	-3.63116
NONHSAT204151.1	1.377206	3.588441	2.977981	0.027185	0.179494	-3.63133
MSTRG.44208.7	-1.23594	2.188388	-2.97781	0.027191	0.179494	-3.63156
lnc-NR4A2-6:1	1.015596	5.773658	2.977419	0.027203	0.179524	-3.63206
lnc-EXT1-2:1	1.097073	2.640201	2.976874	0.027222	0.17961	-3.63277
NR_024065	-1.12352	5.526867	-2.97621	0.027244	0.179674	-3.63363
MAGI1-IT1:2	-1.63013	3.502826	-2.97604	0.02725	0.179701	-3.63385
NONHSAT191621.1	-1.35003	3.318884	-2.97586	0.027256	0.179726	-3.63408
lnc-C3orf80-1:1	1.342571	1.750094	2.975498	0.027268	0.17974	-3.63455
NONHSAT217095.1	-1.1017	3.019659	-2.97514	0.02728	0.179806	-3.63502
NONHSAT205277.1	1.71837	2.015993	2.974937	0.027287	0.179815	-3.63528
NONHSAT210818.1	-1.16393	3.008679	-2.97474	0.027294	0.179826	-3.63554
lnc-PLEKHA3-19:1	1.096938	3.369867	2.973505	0.027335	0.180011	-3.63714
NR_126564	1.786365	2.810635	2.97344	0.027337	0.180011	-3.63723
lnc-ABHD12B-2:1	-1.08311	1.795982	-2.97342	0.027338	0.180011	-3.63726
MSTRG.67959.1	1.273419	6.921525	2.973084	0.027349	0.180037	-3.63769
CARMN:23	1.085144	2.822694	2.973048	0.02735	0.180037	-3.63774
NONHSAT190141.1	1.027348	8.22393	2.971143	0.027415	0.180246	-3.64021
MSTRG.63532.1	1.357811	2.251442	2.970868	0.027424	0.180284	-3.64057
NONHSAT208093.1	-1.15785	2.564432	-2.96999	0.027454	0.180408	-3.64171
lnc-ACTN1-2:1	-1.90949	3.077537	-2.96965	0.027465	0.180425	-3.64215
lnc-MFSD8-12:1	-1.06655	4.231849	-2.96906	0.027485	0.180487	-3.64292
lnc-IFRD1-1:1	1.181311	2.114311	2.969052	0.027485	0.180487	-3.64293
lnc-KCNJ18-11:1	1.125769	1.691436	2.967756	0.027529	0.180534	-3.64462
lnc-KCNA5-4:1	1.197463	2.021455	2.967504	0.027538	0.18055	-3.64494
NONHSAT173782.1	-1.05381	2.562238	-2.96733	0.027544	0.180577	-3.64517
NONHSAT219494.1	1.11698	2.486003	2.967127	0.027551	0.180599	-3.64543
MSTRG.16151.1	-1.28013	2.013376	-2.96606	0.027587	0.180754	-3.64682
MSTRG.6988.1	-1.0616	4.197708	-2.96591	0.027592	0.180775	-3.64702
NONHSAT208391.1	-1.26	2.2436	-2.96579	0.027596	0.180775	-3.64718

NONHSAT205906.1	-1.49729	3.733592	-2.96546	0.027607	0.180794	-3.6476
lnc-FOXQ1-12:4	1.635595	2.619994	2.965004	0.027623	0.180836	-3.64819
NONHSAT169746.1	1.070012	3.873043	2.964969	0.027624	0.180836	-3.64824
lnc-RAD23B-2:1	-1.10104	2.504952	-2.96451	0.02764	0.180878	-3.64884
ENST00000450440.1	1.378699	3.488051	2.964036	0.027656	0.180948	-3.64945
MSTRG.56148.5	-1.02132	4.870131	-2.96382	0.027663	0.180971	-3.64974
NONHSAT193185.1	1.279673	2.016786	2.963379	0.027678	0.181001	-3.65031
lnc-COA3-1:1	-1.52718	2.128858	-2.96321	0.027684	0.181016	-3.65053
lnc-ITGA2-3:1	1.189628	3.301382	2.963207	0.027684	0.181016	-3.65053
ENST00000321214.2	1.384458	3.06752	2.962066	0.027723	0.181118	-3.65202
NONHSAT178125.1	1.749133	4.824785	2.961895	0.027729	0.18112	-3.65224
NONHSAT164743.1	-1.13176	2.522092	-2.96094	0.027762	0.18121	-3.65348
T259942	-1.22878	2.597176	-2.96092	0.027763	0.18121	-3.6535
MSTRG.2600.1	-1.54986	3.009397	-2.9606	0.027774	0.181247	-3.65393
MSTRG.60741.1	-1.00412	3.001585	-2.96041	0.02778	0.181253	-3.65417
NONHSAT201335.1	1.055395	3.431752	2.960242	0.027786	0.181257	-3.65439
NONHSAT159669.1	1.864248	2.439912	2.960186	0.027788	0.181257	-3.65446
NONHSAT206392.1	-2.26245	2.532205	-2.95965	0.027806	0.181317	-3.65516
lnc-POTEB3-1:6	-1.53462	4.515442	-2.95961	0.027808	0.181317	-3.65521
NONHSAT211115.1	-1.00726	3.969876	-2.95905	0.027827	0.18132	-3.65593
ENST00000650435.1	-1.02606	2.422409	-2.95903	0.027827	0.18132	-3.65596
lnc-KPNA4-1:2	-1.35733	3.399791	-2.95883	0.027834	0.181322	-3.65623
NONHSAT159064.1	1.173417	2.447402	2.958493	0.027846	0.181368	-3.65666
ENST00000659624.1	-1.07462	2.142494	-2.95828	0.027853	0.181402	-3.65694
lnc-NDFIP2-30:1	1.302833	2.107985	2.957756	0.027871	0.181449	-3.65762
lnc-ULK4-13:1	1.033352	1.533436	2.957653	0.027875	0.18146	-3.65776
NONHSAT201039.1	1.16378	1.606844	2.956387	0.027918	0.181662	-3.6594
MSTRG.9929.60	-1.38251	3.077173	-2.95593	0.027934	0.181718	-3.66
lnc-C6orf222-2:1	1.570515	2.978622	2.955209	0.027959	0.181833	-3.66094
NONHSAT167930.1	-1.375	3.451719	-2.95504	0.027965	0.181846	-3.66115
MSTRG.17759.1	1.816451	3.127159	2.954608	0.02798	0.181898	-3.66172
NONHSAT219666.1	1.570498	2.617629	2.954322	0.02799	0.181938	-3.66209
MSTRG.64700.1	1.300901	3.09158	2.953898	0.028004	0.181975	-3.66264
T271716	-1.10088	1.799804	-2.9535	0.028018	0.18203	-3.66316
NONHSAT155747.1	-1.08637	3.823985	-2.95294	0.028038	0.182104	-3.66389
T125434	1.661601	2.524601	2.952842	0.028041	0.182104	-3.66402
NONHSAT192308.1	1.995594	3.625153	2.952357	0.028058	0.182146	-3.66465
MSTRG.6207.19	-1.44672	4.119891	-2.95198	0.028071	0.182172	-3.66514
NONHSAT190488.1	-1.27125	2.16996	-2.95183	0.028076	0.182187	-3.66533
lnc-ARHGAP15-15:1	-1.24539	4.02438	-2.95152	0.028087	0.182218	-3.66574
NONHSAT174094.1	1.67057	2.352111	2.951463	0.028089	0.182218	-3.66581
NONHSAT196989.1	-1.09976	1.647205	-2.95083	0.028111	0.182279	-3.66664
ENST00000430988.1	1.332499	2.249309	2.949717	0.028149	0.182399	-3.66809
NONHSAT156716.1	-1.24163	1.773564	-2.94917	0.028168	0.182455	-3.6688
NONHSAT154620.1	1.440591	3.438825	2.949091	0.028171	0.182455	-3.6689
NONHSAT181574.1	2.074489	2.612852	2.948719	0.028184	0.182455	-3.66939
lnc-DISC1-7:1	-1.17759	1.767449	-2.948	0.028209	0.182455	-3.67032
NONHSAT188106.1	1.240095	3.60843	2.947679	0.02822	0.182455	-3.67074

ENST00000569431.1	1.060148	1.719542	2.947663	0.028221	0.182455	-3.67076
lnc-SHH-2:1	-1.25209	1.985798	-2.94738	0.028231	0.182463	-3.67113
MSTRG.15545.1	-1.30464	3.331654	-2.94734	0.028232	0.182463	-3.67118
lnc-TXNDC2-8:1	-1.54836	3.53609	-2.94681	0.028251	0.18253	-3.67188
ENST00000548217.5	1.154208	4.002024	2.946753	0.028253	0.18253	-3.67195
ENST00000422231.6	-1.00637	1.89844	-2.9467	0.028254	0.18253	-3.67201
NONHSAT221635.1	1.426095	2.256206	2.94651	0.028261	0.18255	-3.67227
ENST00000654400.1	1.684496	7.410432	2.945165	0.028308	0.182697	-3.67402
lnc-NCBP2-AS2-6:2	-1.06866	3.282456	-2.94512	0.02831	0.182697	-3.67408
ENST00000609657.1	-1.12271	3.462443	-2.94432	0.028338	0.182788	-3.67512
NONHSAT175647.1	-1.09779	3.686335	-2.9438	0.028356	0.182801	-3.67579
NONHSAT210794.1	-1.51026	2.369621	-2.94374	0.028358	0.182801	-3.67587
lnc-HSF2BP-1:1	1.123094	2.670621	2.942516	0.028401	0.18295	-3.67747
NONHSAT200153.1	-1.13204	2.152528	-2.94203	0.028418	0.183025	-3.6781
NONHSAT202451.1	1.180265	2.515418	2.941366	0.028442	0.183105	-3.67897
NONHSAT205584.1	2.044268	2.968568	2.940266	0.02848	0.183253	-3.6804
LINC01150:1	1.22388	7.063382	2.940006	0.02849	0.183253	-3.68074
T255242	1.996551	2.837492	2.938942	0.028527	0.183391	-3.68213
ENST00000604992.1	1.328047	7.419673	2.938642	0.028538	0.183431	-3.68252
lnc-SLC24A2-3:1	1.096298	2.374124	2.938245	0.028552	0.183476	-3.68304
ENST00000661878.1	1.007042	2.167865	2.93821	0.028553	0.183476	-3.68308
lnc-FAM13C-4:1	-1.2235	3.420154	-2.93801	0.02856	0.183481	-3.68334
lnc-SMIM3-1:1	1.086405	1.97896	2.93772	0.02857	0.183517	-3.68372
NONHSAT217123.1	1.06833	2.579036	2.937478	0.028579	0.183543	-3.68404
NONHSAT177183.1	1.475212	2.902166	2.937253	0.028587	0.183547	-3.68433
MSTRG.32279.75	-1.31781	3.935082	-2.93556	0.028647	0.183791	-3.68653
ENST00000588697.1	1.348077	2.640579	2.935062	0.028664	0.183826	-3.68719
MSTRG.63299.1	1.139745	2.276203	2.934846	0.028672	0.183826	-3.68747
ENST00000649558.1	-1.18623	2.832171	-2.93404	0.028701	0.183921	-3.68852
NONHSAT153751.1	1.03402	5.131435	2.933906	0.028705	0.183927	-3.68869
ENST00000657385.1	-1.91359	2.472348	-2.9338	0.028709	0.183927	-3.68883
ENST00000565861.1	1.658353	7.467835	2.932798	0.028745	0.184035	-3.69014
lnc-FGFR1OP-16:1	1.037419	7.959075	2.932639	0.02875	0.184048	-3.69035
lnc-HIBADH-1:4	-1.15106	2.395612	-2.93243	0.028758	0.184058	-3.69062
lnc-JCAD-9:1	1.192898	6.496565	2.931713	0.028783	0.184154	-3.69155
NONHSAT211882.1	1.073926	5.084572	2.930029	0.028844	0.184375	-3.69375
NONHSAT205769.1	1.117541	4.475242	2.929675	0.028856	0.184432	-3.69421
ENST00000657087.1	1.444569	2.310478	2.929579	0.02886	0.184442	-3.69434
ENST00000524707.1	-1.29217	2.047708	-2.92929	0.02887	0.184473	-3.69471
MSTRG.32939.1	1.778004	4.013741	2.928339	0.028904	0.184569	-3.69596
ENST00000509496.1	1.548002	3.449886	2.928257	0.028907	0.184569	-3.69606
NONHSAT220597.1	-1.63462	2.710835	-2.9282	0.028909	0.184569	-3.69613
NONHSAT164986.1	1.1099	7.438027	2.928158	0.028911	0.184569	-3.69619
NONHSAT167554.1	1.264685	3.024363	2.928011	0.028916	0.18459	-3.69638
NONHSAT171275.1	1.167827	3.452206	2.927913	0.028919	0.18459	-3.69651
MSTRG.49171.1	-1.03347	3.020011	-2.92748	0.028935	0.184614	-3.69708
ENST00000439198.1	-1.00182	1.594208	-2.92733	0.02894	0.184621	-3.69727
lnc-ADAM19-2:4	1.240296	1.971515	2.927038	0.028951	0.184625	-3.69765

NONHSAT172299.1	1.276379	1.739983	2.926994	0.028952	0.184625	-3.69771
lnc-OR13C4-6:1	1.124157	4.502438	2.926589	0.028967	0.184659	-3.69824
NONHSAT222600.1	1.30154	1.72584	2.926383	0.028974	0.184664	-3.69851
MSTRG.53009.1	1.089507	5.206778	2.926371	0.028975	0.184664	-3.69852
NONHSAT211984.1	1.285813	2.70712	2.925389	0.02901	0.184748	-3.69981
MSTRG.42932.2	-1.02508	3.089976	-2.92524	0.029015	0.184748	-3.7
lnc-HMGB2-12:1	1.388669	2.699701	2.925153	0.029018	0.184748	-3.70011
lnc-SP5-1:1	1.485754	3.075977	2.924859	0.029029	0.184763	-3.7005
lnc-UBL5-1:4	-1.01181	4.16511	-2.92452	0.029041	0.184794	-3.70094
lnc-HOXC13-5:1	1.523694	2.927183	2.923512	0.029077	0.184979	-3.70225
MSTRG.15938.8	1.01624	4.416947	2.923454	0.02908	0.18498	-3.70233
NONHSAT222896.1	1.822021	2.911884	2.923116	0.029092	0.185023	-3.70277
NONHSAT165022.1	1.105015	6.888597	2.923014	0.029095	0.185023	-3.7029
NONHSAT215142.1	-1.23614	3.32739	-2.92148	0.029151	0.185153	-3.7049
ENST00000669561.1	-1.20446	2.543028	-2.92114	0.029163	0.185175	-3.70535
lnc-CTSK-1:1	-1.09968	2.725503	-2.92104	0.029167	0.185184	-3.70548
NONHSAT197602.1	1.00156	8.264224	2.92096	0.02917	0.185185	-3.70559
MSTRG.62326.1	1.002673	2.162327	2.920662	0.02918	0.185225	-3.70598
NONHSAT171819.1	-1.49471	2.226061	-2.92022	0.029196	0.185267	-3.70655
MSTRG.66079.1	-1.18175	1.683674	-2.92022	0.029196	0.185267	-3.70655
T009776	1.226933	6.001119	2.919815	0.029211	0.185313	-3.70708
NONHSAT224019.1	1.202279	1.705438	2.919743	0.029214	0.185313	-3.70718
ENST00000568634.1	-1.06631	1.636343	-2.91913	0.029236	0.18541	-3.70798
MSTRG.26735.22	1.146835	3.497036	2.918944	0.029243	0.18541	-3.70822
lnc-MAPK6-3:10	-1.09432	3.826723	-2.91851	0.029258	0.185415	-3.70878
ENST00000522426.1	1.005792	1.88794	2.918419	0.029262	0.185426	-3.70891
MSTRG.1679.1	-1.27336	2.025868	-2.9176	0.029291	0.18556	-3.70998
NONHSAT162663.1	1.096159	4.695931	2.917427	0.029298	0.18556	-3.7102
ENST00000652941.1	1.839406	4.400823	2.91708	0.02931	0.185625	-3.71065
lnc-PSD-2:1	1.008373	4.08268	2.915219	0.029378	0.185848	-3.71309
NONHSAT175498.1	-1.51461	3.82566	-2.91429	0.029412	0.185993	-3.7143
NONHSAT171467.1	1.510763	3.026455	2.913951	0.029424	0.186035	-3.71474
NONHSAT202874.1	1.564618	2.790282	2.913693	0.029434	0.186066	-3.71508
ENST00000555539.1	1.352381	1.872632	2.913151	0.029454	0.186091	-3.71579
ENST00000417695.2	1.01475	5.662006	2.912662	0.029472	0.186091	-3.71643
NONHSAT218140.1	1.024048	2.102445	2.912644	0.029472	0.186091	-3.71645
NONHSAT211220.1	1.217495	3.518167	2.9126	0.029474	0.186091	-3.71651
NONHSAT208430.1	-1.27662	4.403773	-2.91171	0.029506	0.186181	-3.71767
ENST00000523365.1	1.853953	2.519254	2.911639	0.029509	0.186185	-3.71776
NONHSAT186506.1	1.21442	2.532327	2.911051	0.029531	0.186251	-3.71853
lnc-OR4F21-4:8	1.776987	2.650152	2.911011	0.029532	0.186251	-3.71858
ENST00000414161.1	1.316986	3.472143	2.910948	0.029534	0.186251	-3.71867
lnc-NCOA3-30:1	1.516455	2.995691	2.910294	0.029558	0.186318	-3.71952
NONHSAT159459.1	-1.25498	6.600511	-2.90962	0.029583	0.186431	-3.7204
T273532	1.044154	1.618427	2.908918	0.029609	0.186511	-3.72132
lnc-TRPV2-4:3	1.867025	4.243442	2.908586	0.029621	0.186563	-3.72175
ENST00000540906.1	1.265619	3.80118	2.90838	0.029629	0.186565	-3.72202
NONHSAT160868.1	1.128224	2.90171	2.908261	0.029633	0.186565	-3.72218

NONHSAT201976.1	1.003131	2.403315	2.90773	0.029653	0.186598	-3.72287
NONHSAT196094.1	-1.27779	1.802907	-2.90722	0.029671	0.18666	-3.72354
NONHSAT188959.1	1.341094	2.923671	2.90688	0.029684	0.186715	-3.72398
lnc-SERINC1-1:1	-1.31494	5.259483	-2.90676	0.029688	0.186715	-3.72413
MSTRG.7970.21	1.187043	2.106804	2.906436	0.0297	0.186715	-3.72456
NONHSAT157974.1	1.14687	2.099354	2.906354	0.029703	0.186715	-3.72467
lnc-MED10-23:1	-1.34076	2.839908	-2.90635	0.029703	0.186715	-3.72467
MSTRG.43589.2	1.195607	2.140861	2.905828	0.029723	0.186785	-3.72536
MSTRG.44556.1	1.064931	2.290902	2.905046	0.029752	0.186915	-3.72638
lnc-S1PR1-9:3	-1.00233	2.432191	-2.9048	0.029761	0.186919	-3.7267
lnc-RBMX-6:1	-1.06491	2.317786	-2.90437	0.029776	0.186989	-3.72726
NONHSAT158289.1	1.026927	2.49793	2.904349	0.029777	0.186989	-3.72729
NONHSAT193970.1	-1.68455	3.307897	-2.90306	0.029825	0.187198	-3.72898
MSTRG.35264.1	1.385287	3.817967	2.90305	0.029825	0.187198	-3.72899
lnc-RIPOR3-2:1	1.013253	4.737571	2.902989	0.029828	0.187198	-3.72907
NONHSAT223819.1	1.581463	2.702642	2.902808	0.029834	0.187208	-3.72931
NONHSAT189424.1	-1.2501	2.434135	-2.90265	0.02984	0.18722	-3.72952
NONHSAT186650.1	1.233835	2.317437	2.902312	0.029853	0.18723	-3.72996
MSTRG.9806.6	-1.35211	2.030584	-2.90119	0.029894	0.187397	-3.73142
NONHSAT201900.1	-1.17201	3.129574	-2.89996	0.02994	0.187592	-3.73303
NONHSAT216334.1	1.194048	3.230065	2.899846	0.029944	0.187604	-3.73318
MSTRG.717.1	1.006797	3.786793	2.899557	0.029955	0.187639	-3.73356
lnc-CCDC7-11:1	1.689855	2.632779	2.898756	0.029985	0.187759	-3.73461
ENST00000670797.1	-1.03213	3.445296	-2.89826	0.030004	0.187791	-3.73526
NONHSAT149645.1	1.016033	2.77122	2.898049	0.030011	0.187816	-3.73553
lnc-CRACR2A-1:3	1.014588	6.537003	2.897255	0.030041	0.187902	-3.73657
lnc-VAX2-4:1	1.318403	2.781479	2.896923	0.030054	0.187939	-3.737
NONHSAT185413.1	1.555704	2.21158	2.896218	0.03008	0.188003	-3.73793
ENST00000412809.1	1.316038	5.268434	2.896202	0.03008	0.188003	-3.73795
T377451	1.804786	2.361435	2.896055	0.030086	0.188005	-3.73814
lnc-MRPL47-6:1	1.122759	3.833022	2.895385	0.030111	0.188058	-3.73902
MSTRG.23397.1	-1.48195	2.915932	-2.89525	0.030116	0.188058	-3.7392
NONHSAT205586.1	1.064228	3.307126	2.89522	0.030117	0.188058	-3.73923
lnc-MYOM1-10:2	-1.11033	3.278507	-2.89486	0.030131	0.18812	-3.73971
NONHSAT205885.1	1.061258	1.642079	2.894217	0.030155	0.18819	-3.74054
T215273	1.364696	1.827066	2.894217	0.030155	0.18819	-3.74055
lnc-TES-2:1	-1.37915	4.378471	-2.8942	0.030156	0.18819	-3.74057
MSTRG.63562.1	-1.51545	3.67822	-2.89346	0.030183	0.188232	-3.74154
lnc-PEX2-5:1	-1.21907	2.912653	-2.89338	0.030186	0.188232	-3.74164
NONHSAT164036.1	1.345259	2.827473	2.892094	0.030235	0.188383	-3.74332
NONHSAT210334.1	1.902831	3.090744	2.89198	0.030239	0.188383	-3.74347
lnc-ANXA10-4:1	1.247686	4.210275	2.891849	0.030244	0.188383	-3.74365
NONHSAT214973.1	1.061005	1.64389	2.891844	0.030244	0.188383	-3.74365
lnc-SIRT1-7:1	-1.43809	3.090004	-2.89144	0.030259	0.188426	-3.74418
lnc-SLC16A9-6:1	1.044772	4.080643	2.890887	0.03028	0.188426	-3.7449
lnc-APOL2-5:1	-1.21488	2.732962	-2.89081	0.030283	0.188426	-3.745
lnc-ZBBX-6:1	-1.05677	1.875138	-2.88965	0.030327	0.188582	-3.74652
NONHSAT210185.1	-1.03051	3.572496	-2.88949	0.030333	0.188604	-3.74674

NONHSAT220824.1	-1.10326	2.454263	-2.88892	0.030354	0.188684	-3.74747
lnc-VCPIP1-2:1	-1.00032	2.144601	-2.88815	0.030383	0.18883	-3.74848
T231747	-1.32299	2.806486	-2.88776	0.030398	0.188861	-3.74899
lnc-CAV1-5:1	-1.32112	2.823088	-2.88766	0.030402	0.188861	-3.74913
NONHSAT210208.1	1.52235	3.985006	2.887612	0.030404	0.188861	-3.74919
NONHSAT216268.1	1.720923	2.360669	2.887445	0.03041	0.18888	-3.74941
MSTRG.60713.1	1.213817	3.590422	2.887203	0.030419	0.188883	-3.74973
NONHSAT223975.1	-1.07627	4.233894	-2.88718	0.03042	0.188883	-3.74975
lnc-ACE-5:1	-1.15185	4.019109	-2.88676	0.030436	0.18895	-3.7503
lnc-CALCRL-3:1	1.2543	2.493964	2.886051	0.030463	0.18904	-3.75124
NONHSAT155153.1	1.205847	2.692242	2.886039	0.030463	0.18904	-3.75125
ENST00000582692.2	-1.24182	1.795309	-2.88556	0.030482	0.189091	-3.75188
ENST00000625027.2	-1.40579	2.77097	-2.88419	0.030533	0.18926	-3.75367
LINC02290:25	-1.37535	2.38961	-2.88415	0.030535	0.18926	-3.75372
NONHSAT186963.1	1.026956	2.300932	2.884072	0.030538	0.18926	-3.75383
MSTRG.35747.33	-1.18589	1.941769	-2.88298	0.03058	0.189443	-3.75526
NONHSAT193263.1	1.034028	1.761407	2.882423	0.030601	0.189528	-3.75599
lnc-RPS4Y2-3:1	1.075616	3.672305	2.88232	0.030605	0.189541	-3.75612
NONHSAT198093.1	-1.41306	2.697706	-2.88149	0.030636	0.1897	-3.75721
lnc-ELAVL2-6:1	1.013487	4.361499	2.881447	0.030638	0.1897	-3.75727
lnc-MEI4-14:1	-1.11627	3.996843	-2.88135	0.030642	0.189702	-3.75739
NONHSAT207277.1	2.061614	3.524724	2.880992	0.030655	0.18975	-3.75786
MSTRG.42167.1	1.762068	5.269181	2.88036	0.03068	0.189849	-3.75869
lnc-AKR1C2-6:1	1.355564	2.614278	2.879525	0.030712	0.189922	-3.75978
lnc-FAM122A-1:2	-1.13885	3.644283	-2.8795	0.030713	0.189922	-3.75982
lnc-C5orf67-5:1	1.155827	4.185048	2.87936	0.030718	0.189922	-3.76
ENST00000656476.1	1.087076	1.754802	2.878816	0.030739	0.189942	-3.76071
MSTRG.36323.1	1.902207	2.752044	2.878296	0.030759	0.189992	-3.7614
NONHSAT186791.1	-1.05948	4.243285	-2.87806	0.030768	0.190018	-3.76171
MSTRG.45687.1	-1.3269	1.906416	-2.87773	0.03078	0.190068	-3.76214
NONHSAT190512.1	1.284097	3.5979	2.877491	0.03079	0.190112	-3.76245
lnc-ORC3-3:1	1.093769	5.654	2.87709	0.030805	0.190161	-3.76298
NONHSAT174158.1	1.985988	3.759928	2.877007	0.030808	0.190169	-3.76308
NONHSAT223998.1	1.556878	5.370307	2.876609	0.030823	0.190182	-3.76361
MSTRG.71801.2	1.385301	3.586984	2.876529	0.030826	0.190189	-3.76371
lnc-HEY1-1:8	1.66068	3.359531	2.876397	0.030832	0.190197	-3.76388
lnc-GBP5-5:1	1.210265	1.868955	2.876157	0.030841	0.190219	-3.7642
lnc-PRDM6-2:1	-1.16605	2.926074	-2.87595	0.030849	0.190245	-3.76448
NONHSAT205602.1	1.478115	2.491595	2.875828	0.030853	0.190251	-3.76463
ENST00000540136.1	-1.03896	1.591559	-2.87515	0.030879	0.190341	-3.76552
NONHSAT215265.1	-1.23894	4.515728	-2.87457	0.030902	0.190389	-3.76628
ENST00000606235.1	1.137986	3.841991	2.874559	0.030902	0.190389	-3.76629
NONHSAT166818.1	-1.18536	1.699474	-2.87384	0.03093	0.190475	-3.76724
lnc-AKAP14-6:1	-1.23429	5.446832	-2.873	0.030963	0.190553	-3.76834
ENST00000607727.1	1.202227	3.406899	2.872759	0.030972	0.190584	-3.76865
MSTRG.62019.1	-1.20974	2.994511	-2.87229	0.03099	0.190638	-3.76927
MSTRG.31397.1	1.81489	2.045192	2.871901	0.031005	0.190658	-3.76978
NR_027255	1.056221	3.563975	2.870467	0.03106	0.190803	-3.77166

MSTRG.61389.2	1.806438	4.014489	2.869825	0.031085	0.190923	-3.7725
ENST00000591422.1	1.13498	1.792257	2.869442	0.0311	0.190943	-3.773
NONHSAT214319.1	-1.87876	2.909096	-2.86861	0.031133	0.191061	-3.7741
MSTRG.42956.1	1.569698	4.337481	2.868307	0.031144	0.191097	-3.77449
lnc-CAPG-1:1	1.035539	3.146601	2.868099	0.031152	0.19111	-3.77476
lnc-SFXN1-1:1	-1.07286	1.745108	-2.86797	0.031157	0.19111	-3.77493
ENST00000611634.1	1.527554	2.590311	2.867964	0.031158	0.19111	-3.77494
lnc-DLGAP1-1:1	1.536148	4.63214	2.867655	0.03117	0.191113	-3.77535
NONHSAT161193.1	1.073074	4.813289	2.867524	0.031175	0.191113	-3.77552
NONHSAT158446.1	1.351102	4.069323	2.867436	0.031178	0.191113	-3.77563
MSTRG.49178.1	1.871442	2.869343	2.867398	0.03118	0.191113	-3.77568
LINC02074:12	1.071533	2.141207	2.867398	0.03118	0.191113	-3.77568
lnc-ANTXR1-1:1	-1.46879	2.088922	-2.86729	0.031184	0.191118	-3.77582
NONHSAT222090.1	1.45044	2.887603	2.866874	0.0312	0.191149	-3.77637
lnc-OIT3-2:1	-1.01364	4.527384	-2.86666	0.031208	0.191189	-3.77666
ENST00000607611.1	-1.05419	5.106033	-2.8662	0.031226	0.191223	-3.77725
lnc-FKBP3-6:1	-1.2929	2.981369	-2.86581	0.031242	0.191275	-3.77777
NONHSAT168323.1	1.47965	2.489775	2.865569	0.031251	0.191275	-3.77808
lnc-OLFML3-4:1	1.150896	1.901494	2.865546	0.031252	0.191275	-3.77811
NONHSAT165239.1	1.156209	2.14215	2.865409	0.031257	0.191289	-3.77829
ENST00000570210.1	1.605088	7.336471	2.865152	0.031267	0.191311	-3.77863
lnc-FCRL1-1:4	1.762711	3.205891	2.864761	0.031282	0.191374	-3.77914
ENST00000566912.2	-1.37881	2.906895	-2.86262	0.031366	0.191689	-3.78195
lnc-JPH3-6:2	1.179047	3.506216	2.862254	0.03138	0.191737	-3.78243
lnc-GMDS-22:1	1.08354	3.030846	2.861621	0.031405	0.1918	-3.78326
lnc-RC3H1-3:1	1.019345	1.922797	2.861354	0.031416	0.19184	-3.78361
NONHSAT163857.1	1.574643	3.262865	2.861055	0.031427	0.191847	-3.78401
MSTRG.57865.1	1.288395	2.102822	2.861038	0.031428	0.191847	-3.78403
NONHSAT158497.1	1.050115	2.750941	2.861037	0.031428	0.191847	-3.78403
lnc-CCND1-2:1	1.194932	3.351075	2.860647	0.031443	0.191899	-3.78454
NONHSAT222474.1	-1.15393	2.104702	-2.86063	0.031444	0.191899	-3.78456
lnc-SNAI1-3:1	-1.53994	3.082771	-2.86058	0.031446	0.191899	-3.78463
NONHSAT215450.1	1.432986	3.375351	2.860107	0.031465	0.191954	-3.78525
NONHSAT186627.1	1.62043	2.224169	2.860022	0.031468	0.191963	-3.78536
NONHSAT197546.1	1.310298	2.151753	2.859701	0.031481	0.192001	-3.78578
NONHSAT169829.1	-1.1983	2.361427	-2.85967	0.031482	0.192001	-3.78583
NONHSAT178698.1	1.879633	3.362928	2.858692	0.03152	0.192102	-3.78711
lnc-TP53INP2-1:1	1.701314	8.349001	2.858632	0.031523	0.192102	-3.78719
NONHSAT195745.1	1.164762	6.896391	2.858311	0.031535	0.192139	-3.78761
NONHSAT197053.1	1.060934	3.745544	2.858026	0.031547	0.192139	-3.78798
lnc-ATXN10-2:1	-1.18184	2.090673	-2.85748	0.031568	0.192217	-3.7887
ENST00000659799.1	-1.1413	3.578188	-2.857	0.031587	0.192257	-3.78932
MSTRG.38265.1	1.306971	2.6256	2.856863	0.031592	0.192264	-3.78951
ENST00000542577.2	1.222142	2.501928	2.85657	0.031604	0.19228	-3.78989
lnc-PIK3C3-2:4	-1.09203	2.502795	-2.85639	0.031611	0.192301	-3.79013
NONHSAT180184.1	1.326334	2.278894	2.856165	0.03162	0.19232	-3.79043
MSTRG.52420.1	1.186468	2.133526	2.856114	0.031622	0.19232	-3.79049
NONHSAT218820.1	1.091308	3.224909	2.856018	0.031626	0.192323	-3.79062

lnc-LRGUK-4:1	-1.15417	4.178719	-2.85589	0.031631	0.192323	-3.79079
NONHSAT156420.1	1.864332	3.229476	2.855336	0.031653	0.192401	-3.79151
NONHSAT197114.1	1.297201	4.034533	2.855324	0.031653	0.192401	-3.79153
lnc-GAS1-4:1	1.243479	2.171479	2.854714	0.031677	0.192461	-3.79233
lnc-TOX3-10:1	1.518389	6.884146	2.854708	0.031678	0.192461	-3.79234
NONHSAT205868.1	1.074771	1.813231	2.854551	0.031684	0.192487	-3.79254
NR_125941	1.536922	2.652609	2.854182	0.031698	0.192521	-3.79303
NONHSAT217282.1	1.202301	3.236237	2.85369	0.031718	0.19256	-3.79367
lnc-NUCB2-2:1	-1.00337	4.402081	-2.85322	0.031737	0.192569	-3.79429
MSTRG.7456.1	-1.06144	2.424386	-2.85215	0.031779	0.192705	-3.79569
NONHSAT202778.1	1.135611	1.665177	2.851912	0.031788	0.19274	-3.79601
NONHSAT198775.1	1.329408	4.520081	2.85114	0.031819	0.192764	-3.79702
lnc-SPTSSB-7:1	1.491263	2.967949	2.850817	0.031832	0.192795	-3.79745
MSTRG.36930.2	1.159371	2.226706	2.850683	0.031837	0.192795	-3.79762
lnc-PPP2R3C-2:1	1.708798	3.607944	2.849921	0.031868	0.192826	-3.79862
NONHSAT201299.1	1.15652	2.248748	2.848827	0.031911	0.19294	-3.80006
lnc-ASCC3-4:1	-1.53277	2.418684	-2.84743	0.031967	0.193079	-3.80189
NONHSAT221747.1	1.546552	2.467136	2.846587	0.032001	0.193214	-3.803
lnc-NEDD1-8:1	1.471718	2.318418	2.846122	0.03202	0.193268	-3.80362
MSTRG.2923.1	1.258855	4.260297	2.84603	0.032023	0.193268	-3.80374
NONHSAT180745.1	1.084036	2.141662	2.845658	0.032038	0.193288	-3.80422
ENST00000655873.1	1.33061	2.272595	2.845295	0.032053	0.193342	-3.8047
lnc-PSD3-4:1	1.258596	2.256594	2.844447	0.032087	0.193456	-3.80582
ENST00000514411.1	1.371379	2.82152	2.844439	0.032087	0.193456	-3.80583
NONHSAT201086.1	1.453243	2.711385	2.844244	0.032095	0.19348	-3.80608
NONHSAT209963.1	1.610682	2.571402	2.843444	0.032127	0.193628	-3.80714
T304305	-1.20978	2.474465	-2.84291	0.032149	0.193723	-3.80784
lnc-PABPC1-2:1	-1.08092	3.075876	-2.8424	0.032169	0.193801	-3.80851
NONHSAT157010.1	2.230113	3.046362	2.842141	0.032179	0.193851	-3.80885
T016182	-1.1313	2.302566	-2.84012	0.032261	0.194132	-3.81151
NONHSAT197418.1	1.145984	1.888425	2.840029	0.032265	0.194132	-3.81162
NONHSAT190050.1	1.008533	3.770357	2.838146	0.032341	0.194388	-3.8141
NONHSAT210822.1	1.452956	1.740479	2.838121	0.032342	0.194388	-3.81413
NONHSAT153965.1	1.208467	1.85651	2.837972	0.032348	0.194395	-3.81433
NONHSAT174274.1	1.427578	2.377719	2.837947	0.032349	0.194395	-3.81436
NONHSAT201823.1	1.201605	3.584154	2.836774	0.032396	0.194535	-3.8159
MSTRG.49407.3	1.026278	3.646933	2.836608	0.032403	0.194535	-3.81612
NONHSAT190296.1	1.749553	3.736449	2.836017	0.032427	0.194634	-3.8169
ENST00000573260.1	-1.1745	1.937417	-2.83497	0.03247	0.194739	-3.81828
NONHSAT215015.1	1.163633	2.065715	2.834578	0.032486	0.194765	-3.81879
NONHSAT150633.1	1.528606	4.967338	2.833379	0.032534	0.194877	-3.82037
ENST00000658879.1	1.446135	1.826169	2.833349	0.032536	0.194877	-3.82041
lnc-LYPLAL1-7:1	-1.24861	3.207566	-2.83325	0.03254	0.194877	-3.82054
MSTRG.39891.1	1.07552	3.480695	2.833213	0.032541	0.194877	-3.82059
NONHSAT180797.1	1.013799	1.610725	2.832913	0.032553	0.194893	-3.82098
lnc-CCDC28A-3:1	1.641246	4.002425	2.831927	0.032594	0.195053	-3.82228
lnc-SELENOV-4:1	1.716951	3.120689	2.831672	0.032604	0.195057	-3.82261
NONHSAT196031.1	1.375176	2.904348	2.831055	0.032629	0.195134	-3.82343

lnc-C2orf91-5:1	1.201943	1.677623	2.831023	0.032631	0.195134	-3.82347
NONHSAT167509.1	1.004456	3.404017	2.83098	0.032632	0.195134	-3.82352
NONHSAT171149.1	1.486018	2.679265	2.830766	0.032641	0.195146	-3.82381
lnc-MBOAT1-9:1	1.535485	1.928004	2.83074	0.032642	0.195146	-3.82384
NONHSAT182791.1	-1.39749	3.86035	-2.83006	0.03267	0.195247	-3.82474
ENST00000605007.1	-1.13673	5.075627	-2.82983	0.032679	0.195247	-3.82504
NONHSAT197519.1	-1.50271	1.967428	-2.82969	0.032685	0.195247	-3.82522
lnc-C14orf132-2:1	-1.45029	3.589055	-2.82935	0.032699	0.19529	-3.82567
NONHSAT214775.1	1.368915	3.317803	2.828303	0.032742	0.195399	-3.82705
lnc-UGGT1-8:1	1.025756	2.747833	2.828018	0.032754	0.195431	-3.82742
NONHSAT205772.1	1.363261	2.719506	2.827633	0.032769	0.195485	-3.82793
lnc-E2F6-6:1	1.502318	2.965342	2.82761	0.03277	0.195485	-3.82796
NONHSAT153422.1	1.151183	2.157235	2.827346	0.032781	0.195499	-3.82831
MSTRG.70036.1	1.620969	3.028322	2.826843	0.032802	0.195556	-3.82897
MSTRG.1195.2	1.210706	2.342817	2.826726	0.032807	0.195556	-3.82912
NONHSAT154396.1	1.059626	3.250892	2.825606	0.032853	0.195745	-3.8306
lnc-ATG2B-18:1	1.21978	3.182312	2.825528	0.032856	0.195752	-3.8307
NONHSAT158010.1	1.549412	2.430207	2.8251	0.032874	0.195808	-3.83126
lnc-SSTR2-1:2	1.118646	1.833453	2.825021	0.032877	0.195808	-3.83137
lnc-FBXL3-1:6	-1.12002	3.703932	-2.82462	0.032894	0.195818	-3.83189
T379777	1.709166	3.032228	2.823993	0.032919	0.195841	-3.83272
MSTRG.34711.1	-1.08074	2.742513	-2.82383	0.032926	0.195841	-3.83293
ENST00000635331.1	-1.36703	3.018726	-2.82361	0.032935	0.195866	-3.83323
lnc-GPBP1-9:2	1.244648	5.768678	2.82315	0.032954	0.19594	-3.83383
lnc-C1D-8:1	1.428869	2.856532	2.822798	0.032969	0.195971	-3.83429
NONHSAT218590.1	-1.46203	2.985789	-2.82268	0.032974	0.195971	-3.83444
T315345	2.13986	4.04258	2.822473	0.032982	0.195994	-3.83472
NONHSAT214616.1	1.491394	6.537516	2.822007	0.033002	0.196072	-3.83533
lnc-ZNF587-2:1	-1.39822	2.660906	-2.82195	0.033004	0.196072	-3.83541
NONHSAT200360.1	1.564398	2.91064	2.821855	0.033008	0.196072	-3.83553
lnc-GRM4-2:2	-1.1321	2.222163	-2.82183	0.033009	0.196072	-3.83557
lnc-FOXP1-4:1	1.146086	2.514665	2.821661	0.033016	0.196082	-3.83579
NONHSAT189307.1	1.287321	2.630697	2.821482	0.033023	0.196111	-3.83602
MSTRG.22405.1	1.956421	5.537124	2.820527	0.033063	0.196207	-3.83728
MSTRG.12929.1	-1.07849	3.363505	-2.81993	0.033088	0.196291	-3.83807
MSTRG.5234.11	1.230797	2.738708	2.819764	0.033095	0.196303	-3.83829
NONHSAT173841.1	1.33146	2.104732	2.819293	0.033114	0.196361	-3.83891
lnc-UFM1-3:1	1.054448	4.378856	2.818309	0.033155	0.196498	-3.8402
MSTRG.57030.11	1.577751	2.554206	2.817139	0.033204	0.196591	-3.84174
NONHSAT165189.1	-1.1094	1.668599	-2.81695	0.033211	0.196614	-3.84199
lnc-ZSWIM6-7:1	1.689806	2.595794	2.816677	0.033223	0.19666	-3.84235
NONHSAT191789.1	1.369161	2.887254	2.815777	0.033261	0.196824	-3.84354
NONHSAT221212.1	1.920302	2.687817	2.814883	0.033298	0.196899	-3.84472
NONHSAT221244.1	1.582889	2.820847	2.814708	0.033305	0.196899	-3.84495
NR_102371	1.025656	3.617857	2.814503	0.033314	0.196899	-3.84522
lnc-RAD21-1:6	1.081225	2.776517	2.814499	0.033314	0.196899	-3.84522
NONHSAT205197.1	1.016896	1.641218	2.813173	0.033369	0.197093	-3.84697
ENST00000555303.1	-1.07066	1.664797	-2.81316	0.03337	0.197093	-3.84698

ENST00000577835.1	1.109256	2.437138	2.811857	0.033425	0.197295	-3.8487
NONHSAT202019.1	-1.3277	3.191289	-2.81136	0.033446	0.197353	-3.84936
NGF-AS1:6	1.395193	3.144116	2.811006	0.03346	0.197384	-3.84982
NONHSAT211423.1	1.098043	1.598103	2.810275	0.033491	0.197482	-3.85079
ENST00000655303.1	1.148071	5.316812	2.81018	0.033495	0.197494	-3.85091
NONHSAT175098.1	1.367194	2.194324	2.809862	0.033508	0.197515	-3.85133
lnc-STARD5-1:1	-1.12609	3.103586	-2.80874	0.033556	0.197608	-3.85281
NONHSAT208296.1	-1.14624	4.195438	-2.80862	0.033561	0.197608	-3.85297
NONHSAT218646.1	-1.00155	2.921456	-2.80817	0.03358	0.197646	-3.85356
NONHSAT201607.1	1.561064	2.53461	2.807773	0.033597	0.197694	-3.85408
ENST00000652848.1	-1.14396	1.694014	-2.80759	0.033604	0.197717	-3.85432
NR_047508	-1.44361	3.016649	-2.80709	0.033625	0.197777	-3.85499
LINC01268:11	-1.13334	4.913537	-2.80613	0.033666	0.197949	-3.85625
lnc-CDH6-21:1	-1.39501	1.96547	-2.80496	0.033716	0.198134	-3.85779
lnc-C17orf62-6:1	-1.33276	2.649524	-2.80484	0.033721	0.19814	-3.85795
ENST00000666585.1	-1.28216	3.000404	-2.80475	0.033724	0.198143	-3.85807
ENST00000526947.1	-1.42304	3.023552	-2.80408	0.033753	0.198236	-3.85895
NONHSAT214027.1	1.274324	1.872716	2.803297	0.033786	0.19836	-3.85998
MSTRG.35550.1	-1.51273	3.875265	-2.80292	0.033802	0.1984	-3.86049
NONHSAT174848.1	-1.33914	2.743328	-2.80238	0.033825	0.198451	-3.86119
lnc-HNRNPA1P48-18:1	-1.24243	3.434592	-2.80235	0.033826	0.198451	-3.86123
lnc-PRPS1-4:2	1.447984	2.932049	2.801416	0.033866	0.198553	-3.86246
lnc-PPP1R3G-7:1	-1.05786	2.365442	-2.80128	0.033872	0.198561	-3.86264
NONHSAT150141.1	1.609771	3.097305	2.800763	0.033894	0.198616	-3.86332
NONHSAT211848.1	1.45987	2.360638	2.800723	0.033896	0.198616	-3.86338
ENST00000449780.1	1.367485	2.295	2.800113	0.033922	0.198719	-3.86418
T067871	1.084622	2.198973	2.799366	0.033953	0.198833	-3.86517
NONHSAT167738.1	1.311578	5.952872	2.799332	0.033955	0.198833	-3.86521
ENST00000648175.1	1.401956	5.741709	2.799013	0.033969	0.198855	-3.86563
NONHSAT167976.1	1.104024	10.62312	2.797969	0.034013	0.198953	-3.86701
lnc-ZNF664-3:3	-1.24106	2.136695	-2.79757	0.03403	0.19896	-3.86753
NONHSAT207609.1	-1.37384	2.135031	-2.79706	0.034052	0.19902	-3.86821
NONHSAT210695.1	-1.3843	3.020205	-2.79686	0.034061	0.19906	-3.86848
lnc-HNRNPA1P48-17:1	1.289059	2.090339	2.796748	0.034065	0.19907	-3.86862
MSTRG.31648.85	-1.0008	1.610429	-2.7966	0.034072	0.19907	-3.86882
NONHSAT187302.1	1.122295	5.828258	2.796522	0.034075	0.19907	-3.86892
MSTRG.43849.2	1.056781	4.157347	2.796269	0.034086	0.199115	-3.86925
ENST00000668805.1	-1.10587	3.010247	-2.79576	0.034108	0.199134	-3.86993
NONHSAT216462.1	1.184962	1.903495	2.795699	0.03411	0.199134	-3.87
NONHSAT204935.1	1.703701	3.83549	2.795646	0.034113	0.199134	-3.87008
NONHSAT212141.1	1.085052	4.184109	2.795641	0.034113	0.199134	-3.87008
NONHSAT168816.1	-1.28013	2.674206	-2.79499	0.034141	0.199225	-3.87094
NONHSAT177949.1	1.289036	1.726337	2.794971	0.034142	0.199225	-3.87097
lnc-CLEC4M-3:1	-1.19148	5.462491	-2.79387	0.034189	0.19938	-3.87242
T283245	1.212405	4.234197	2.793503	0.034205	0.199439	-3.8729
lnc-TPK1-3:1	1.046263	5.906838	2.792637	0.034242	0.199615	-3.87404
NONHSAT179030.1	1.296794	4.435848	2.791944	0.034272	0.199738	-3.87496
NONHSAT199992.1	1.263064	2.489719	2.79186	0.034276	0.199748	-3.87507

ENST00000508581.1	1.115847	1.688546	2.791272	0.034301	0.199815	-3.87585
NONHSAT161209.1	1.205841	4.883647	2.791117	0.034308	0.199829	-3.87605
T150949	1.557357	3.071278	2.790097	0.034352	0.199915	-3.8774
NONHSAT200649.1	1.070668	3.352063	2.789469	0.034379	0.199992	-3.87823
lnc-USP6-3:1	-1.24726	3.624303	-2.78892	0.034403	0.200107	-3.87895
NONHSAT217900.1	-1.01443	3.767982	-2.78858	0.034418	0.200182	-3.8794
NONHSAT170374.1	-1.09186	3.815015	-2.78807	0.03444	0.200253	-3.88007
ENST00000666383.1	1.102336	3.555095	2.787854	0.034449	0.200254	-3.88036
lnc-NTMT1-7:2	1.253703	1.758501	2.787835	0.03445	0.200254	-3.88038
ENST00000435702.1	1.067041	6.785998	2.787491	0.034465	0.200306	-3.88084
MSTRG.37338.1	-1.46453	2.726708	-2.7872	0.034477	0.200321	-3.88122
lnc-ZNF614-1:2	1.327366	6.789842	2.786878	0.034491	0.200371	-3.88165
lnc-DYNAP-36:1	1.256881	2.579664	2.78594	0.034532	0.200514	-3.88288
NONHSAT221543.1	1.311705	2.759229	2.785528	0.03455	0.200523	-3.88343
lnc-LSM11-5:1	-1.36997	1.931235	-2.78467	0.034587	0.200661	-3.88457
T224929	-1.25306	2.739917	-2.78333	0.034646	0.200812	-3.88633
lnc-LAMA1-6:1	1.602598	2.833001	2.783129	0.034655	0.200834	-3.8866
T347696	-1.24193	3.900608	-2.78203	0.034702	0.200958	-3.88804
lnc-OLFML3-3:1	1.690402	5.150703	2.781225	0.034738	0.201083	-3.88911
IDH2-DT:16	1.179088	2.872659	2.780807	0.034756	0.201098	-3.88966
NONHSAT191408.1	1.354492	2.161864	2.780777	0.034757	0.201098	-3.8897
NONHSAT219981.1	1.070018	2.026556	2.77983	0.034799	0.201239	-3.89095
MSTRG.66755.1	1.31672	2.497374	2.779557	0.034811	0.201262	-3.89131
ENST00000569778.1	-1.10917	2.311683	-2.77919	0.034827	0.201299	-3.8918
lnc-ATP6V1G3-4:4	1.432871	7.584146	2.778915	0.034839	0.201334	-3.89216
lnc-TCIM-4:1	-1.22744	2.682783	-2.77837	0.034863	0.201402	-3.89288
NONHSAT172924.1	-1.22415	1.969035	-2.77758	0.034898	0.201509	-3.89392
NONHSAT172358.1	1.563568	2.40944	2.777229	0.034913	0.201569	-3.89439
ENST00000556212.1	1.234341	3.187819	2.777212	0.034914	0.201569	-3.89441
lnc-ADGRA3-107:2	-1.00391	4.17251	-2.77681	0.034932	0.201644	-3.89494
NONHSAT200427.1	1.524166	2.341944	2.776028	0.034966	0.201709	-3.89598
MSTRG.42822.5	1.174034	2.554891	2.775788	0.034977	0.201724	-3.89629
ENST00000454622.2	1.293764	4.900713	2.775741	0.034979	0.201724	-3.89635
lnc-KHDRBS2-2:1	1.485111	2.63269	2.775534	0.034988	0.201758	-3.89663
lnc-ATP6V1C1-12:1	1.734175	2.614201	2.775487	0.03499	0.201758	-3.89669
T365986	1.153452	3.682712	2.775387	0.034994	0.201768	-3.89682
GAS1RR:14	1.306227	1.681719	2.774028	0.035054	0.201975	-3.89862
NONHSAT205534.1	-1.00331	1.745511	-2.77286	0.035106	0.202088	-3.90016
NONHSAT206003.1	1.575889	2.485729	2.77257	0.035119	0.202104	-3.90054
NONHSAT199849.1	1.061075	4.315771	2.772337	0.035129	0.202129	-3.90085
MSTRG.64186.1	1.416926	2.345779	2.772087	0.03514	0.202156	-3.90118
NONHSAT176146.1	1.073471	3.022688	2.771281	0.035176	0.202295	-3.90225
lnc-EXTL2-3:1	-1.12424	1.688486	-2.77103	0.035187	0.20233	-3.90258
ENST00000451295.1	-1.12483	2.633693	-2.77096	0.03519	0.20233	-3.90267
MSTRG.61091.1	-1.30869	2.312385	-2.77072	0.035201	0.202346	-3.90299
PCGEM1:5	1.110749	3.427239	2.77041	0.035215	0.202369	-3.9034
lnc-KLHL8-2:1	1.013584	1.606748	2.770169	0.035225	0.202369	-3.90372
NONHSAT177628.1	-1.42832	3.223855	-2.76999	0.035233	0.202369	-3.90395

NONHSAT199656.1	1.475946	7.769392	2.769623	0.03525	0.202407	-3.90444
lnc-MRPL39-19:1	1.248319	2.330259	2.768935	0.03528	0.202502	-3.90535
lnc-DUSP10-7:1	-1.11452	3.07124	-2.76893	0.03528	0.202502	-3.90535
NONHSAT164390.1	1.547362	3.217014	2.768705	0.035291	0.202537	-3.90565
lnc-C3orf58-4:1	-1.17341	1.729222	-2.76852	0.035299	0.202572	-3.90589
MSTRG.9702.1	-1.11338	2.469581	-2.76816	0.035315	0.202633	-3.90638
NONHSAT205759.1	1.731923	3.38223	2.768097	0.035318	0.202635	-3.90646
NONHSAT174569.1	-1.23031	3.709106	-2.76757	0.035341	0.202656	-3.90715
NONHSAT167611.1	-1.50454	2.840233	-2.76757	0.035341	0.202656	-3.90715
NONHSAT179604.1	-1.01163	4.310155	-2.76755	0.035342	0.202656	-3.90718
lnc-TC2N-3:1	-1.18854	5.163458	-2.76691	0.03537	0.202754	-3.90802
lnc-SLC28A1-3:1	-1.31055	3.012793	-2.76659	0.035385	0.202825	-3.90845
LINC02322:6	1.318192	3.962253	2.766247	0.0354	0.202855	-3.9089
lnc-UBE2E3-4:1	-1.82977	3.469365	-2.76556	0.035431	0.202965	-3.9098
NONHSAT201779.1	-1.19806	1.941528	-2.76533	0.035441	0.202985	-3.91011
ENST00000649665.1	1.721945	2.573641	2.764966	0.035457	0.203012	-3.9106
NONHSAT214965.1	1.330494	2.796303	2.764847	0.035463	0.203029	-3.91075
LINC01226:10	1.233151	3.852575	2.764389	0.035483	0.203107	-3.91136
NONHSAT178395.1	-1.17589	2.329493	-2.76307	0.035543	0.203278	-3.91311
lnc-FAM207A-5:1	-1.22326	2.690702	-2.76264	0.035562	0.203321	-3.91367
NONHSAT179398.1	1.02168	5.525582	2.762557	0.035565	0.203321	-3.91378
LINC00620:11	1.372286	3.683596	2.762201	0.035581	0.203374	-3.91425
MSTRG.51248.1	1.421718	3.557578	2.762156	0.035583	0.203374	-3.91431
NONHSAT221592.1	1.425334	2.302415	2.762079	0.035587	0.203382	-3.91441
NONHSAT182371.1	1.090262	2.318809	2.76195	0.035593	0.203392	-3.91458
NONHSAT167634.1	1.175414	1.716161	2.761842	0.035598	0.203408	-3.91473
MSTRG.62122.1	1.130166	1.759722	2.761399	0.035618	0.203473	-3.91531
MSTRG.38714.3	1.169171	5.560516	2.761319	0.035621	0.203473	-3.91542
MSTRG.72700.1	-1.12523	1.954636	-2.75902	0.035725	0.20372	-3.91846
NONHSAT202134.1	-1.12422	4.042662	-2.7578	0.03578	0.203957	-3.92007
NONHSAT191331.1	-1.45347	2.588898	-2.75744	0.035796	0.20399	-3.92055
NONHSAT206541.1	1.039495	1.981034	2.757064	0.035813	0.203999	-3.92105
MSTRG.52476.1	1.125165	2.313594	2.756592	0.035834	0.204091	-3.92167
T158189	1.504589	2.159838	2.756423	0.035842	0.204123	-3.9219
lnc-FAM92A-2:2	-1.30616	3.631098	-2.75604	0.035859	0.204154	-3.9224
LINC01105:12	1.079568	2.987208	2.755084	0.035903	0.204307	-3.92367
NONHSAT178303.1	2.041104	5.598493	2.754726	0.035919	0.20432	-3.92414
ENST00000669514.1	1.074296	5.54112	2.754708	0.03592	0.20432	-3.92416
NR_120650	1.662671	2.638603	2.754514	0.035929	0.204327	-3.92442
lnc-CELSR1-2:3	1.153313	1.779405	2.754329	0.035937	0.204348	-3.92467
NONHSAT167675.1	1.10549	4.269257	2.753643	0.035968	0.204414	-3.92557
NONHSAT176558.1	1.808147	2.796878	2.753466	0.035976	0.204414	-3.92581
NONHSAT157362.1	1.016112	2.355894	2.753401	0.035979	0.204419	-3.92589
NONHSAT167252.1	1.258078	2.162443	2.75089	0.036094	0.204805	-3.92922
ENST00000658911.1	-1.32886	1.88385	-2.75047	0.036113	0.204865	-3.92977
MSTRG.20193.11	1.210729	2.061174	2.750259	0.036122	0.204875	-3.93005
ENST00000657329.1	1.035189	2.148787	2.749774	0.036145	0.204932	-3.93069
MSTRG.49299.3	-1.0958	3.941936	-2.74959	0.036153	0.204942	-3.93094

lnc-SLC12A8-4:1	-1.19527	4.313615	-2.7494	0.036162	0.204972	-3.9312
lnc-OR56A3-1:1	1.434075	2.945615	2.749103	0.036175	0.205015	-3.93158
NONHSAT197603.1	1.423624	2.834618	2.748868	0.036186	0.205021	-3.93189
NONHSAT187598.1	1.909552	4.738349	2.748854	0.036187	0.205021	-3.93191
MSTRG.38686.4	1.264846	4.370605	2.748775	0.03619	0.205029	-3.93202
NONHSAT188086.1	1.216178	2.125175	2.748437	0.036206	0.205092	-3.93246
MSTRG.43870.19	1.147597	4.346901	2.747719	0.036239	0.205188	-3.93342
lnc-SERTM2-5:1	-1.20973	3.138624	-2.74677	0.036282	0.205331	-3.93468
ENST00000657346.1	1.025705	2.131669	2.746408	0.036299	0.205401	-3.93515
LINC02360:5	-1.02592	1.98461	-2.74593	0.036321	0.205491	-3.93579
lnc-DPPA4-1:2	1.238841	7.914297	2.74586	0.036324	0.205491	-3.93588
ENST00000451697.1	1.629301	3.179839	2.745601	0.036336	0.20553	-3.93622
lnc-LECT2-2:1	-1.19875	5.698202	-2.74496	0.036365	0.205627	-3.93707
NONHSAT161564.1	1.046937	1.790092	2.744831	0.036371	0.205627	-3.93724
NONHSAT150603.1	-1.28546	4.958814	-2.74469	0.036378	0.205629	-3.93743
NONHSAT165018.1	1.017594	1.851426	2.744639	0.03638	0.205631	-3.93749
ENST00000560034.1	-1.37455	5.64066	-2.74454	0.036385	0.205641	-3.93763
ENST00000665850.1	-1.06978	3.321025	-2.74438	0.036392	0.205663	-3.93783
NONHSAT187986.1	-1.17053	3.030821	-2.74412	0.036404	0.205719	-3.93818
lnc-HECTD2-1:1	-1.47878	3.372808	-2.74399	0.03641	0.205731	-3.93836
T040935	-1.52368	2.879366	-2.74388	0.036415	0.205736	-3.9385
lnc-FOXQ1-26:1	-1.08469	2.689964	-2.74347	0.036434	0.205818	-3.93904
lnc-TFAP2E-7:1	1.382944	3.810291	2.743433	0.036436	0.205818	-3.93909
lnc-PRKACB-7:1	-1.0075	1.89822	-2.74326	0.036444	0.205828	-3.93932
NONHSAT174247.1	1.123784	1.685638	2.74305	0.036453	0.205848	-3.9396
NONHSAT163745.1	-1.00984	2.53369	-2.74287	0.036461	0.205883	-3.93983
lnc-FCGR3B-4:11	1.077571	3.036038	2.742464	0.03648	0.205966	-3.94037
MSTRG.26270.2	1.789254	3.076605	2.741211	0.036538	0.206186	-3.94203
NONHSAT164633.1	-1.02578	1.769307	-2.74118	0.03654	0.206186	-3.94207
lnc-IFNK-5:1	1.0897	3.782859	2.740973	0.036549	0.206217	-3.94235
MSTRG.65860.1	-1.29716	2.075273	-2.74047	0.036573	0.206242	-3.94302
lnc-PRPF18-12:1	-1.01774	3.603211	-2.74005	0.036592	0.206307	-3.94357
lnc-GLUD1-1:1	-1.18025	4.630023	-2.73955	0.036615	0.206391	-3.94423
lnc-MSH2-1:4	1.041424	7.804069	2.738982	0.036641	0.206478	-3.94498
lnc-GCNT2-5:1	-1.00204	8.438896	-2.73791	0.036691	0.206614	-3.94641
lnc-ACKR3-7:1	1.570293	3.171026	2.737415	0.036714	0.206652	-3.94706
NONHSAT166827.1	-1.06798	1.946558	-2.73734	0.036718	0.206652	-3.94716
NONHSAT204280.1	-1.13954	1.648236	-2.73688	0.036739	0.206687	-3.94777
lnc-MBL2-6:2	-1.09795	5.553179	-2.73675	0.036745	0.206703	-3.94794
ENST00000587836.1	-1.23353	2.618852	-2.73657	0.036754	0.206715	-3.94818
lnc-IRF1-7:1	-1.06715	4.03354	-2.73638	0.036762	0.206715	-3.94843
lnc-CCDC57-3:1	-1.27603	3.25092	-2.73629	0.036766	0.206725	-3.94855
lnc-GPR27-6:1	1.35769	2.279972	2.735251	0.036815	0.206836	-3.94993
lnc-CXCL12-5:1	-1.18778	3.628701	-2.7352	0.036817	0.206836	-3.94999
ENST00000671426.1	1.631699	2.639453	2.73498	0.036828	0.206861	-3.95029
MSTRG.56553.1	-1.37087	2.652076	-2.73462	0.036844	0.206909	-3.95076
MSTRG.69335.1	1.645288	2.630896	2.734238	0.036862	0.206964	-3.95127
lnc-POU4F2-1:1	1.555782	2.225836	2.733354	0.036904	0.207102	-3.95244

NONHSAT163851.1	1.033201	3.43989	2.73292	0.036924	0.20712	-3.95302
MSTRG.45900.1	1.327981	3.67713	2.732895	0.036925	0.20712	-3.95305
NONHSAT154287.1	-1.00855	4.168985	-2.73243	0.036947	0.20722	-3.95367
MSTRG.51866.2	-1.23748	2.857917	-2.7323	0.036953	0.207225	-3.95383
NONHSAT175302.1	1.026058	5.240939	2.731404	0.036995	0.207373	-3.95503
NONHSAT201967.1	1.263641	2.351088	2.731303	0.037	0.207377	-3.95516
NONHSAT191858.1	1.362359	5.939781	2.730672	0.037029	0.207473	-3.956
lnc-ZFYVE1-2:1	1.100741	2.0657	2.73049	0.037038	0.207486	-3.95624
NONHSAT171806.1	1.20793	2.594249	2.730193	0.037052	0.207532	-3.95663
NONHSAT183119.1	-1.20219	1.698592	-2.73018	0.037052	0.207532	-3.95664
ENST00000580524.1	1.516922	2.530071	2.730071	0.037057	0.207542	-3.95679
lnc-NDFIP2-15:1	1.178139	2.099017	2.729943	0.037063	0.207546	-3.95696
lnc-EPB42-3:1	1.265646	2.637487	2.729908	0.037065	0.207546	-3.95701
lnc-CD109-8:1	1.715069	5.862136	2.728477	0.037132	0.207701	-3.95891
lnc-LAD1-1:2	1.691562	2.46595	2.728449	0.037134	0.207701	-3.95894
NR_104655	1.514224	1.863154	2.727096	0.037197	0.207896	-3.96074
ENST00000659604.1	1.358327	1.835196	2.726941	0.037205	0.207913	-3.96094
lnc-GLIPR1L1-1:7	1.504766	3.018543	2.726654	0.037218	0.207949	-3.96132
lnc-NPHP3-ACAD11-1:2	1.000284	7.220766	2.726341	0.037233	0.208014	-3.96174
NONHSAT218514.1	1.396166	2.725479	2.725715	0.037263	0.208106	-3.96257
NONHSAT179949.1	1.185263	6.865972	2.725642	0.037266	0.208106	-3.96266
lnc-CERK-10:3	1.336489	2.823248	2.725421	0.037276	0.208107	-3.96296
lnc-NXPH2-9:1	1.126125	1.647252	2.723687	0.037358	0.208306	-3.96526
NONHSAT205944.1	1.330928	2.218799	2.722561	0.037412	0.208459	-3.96675
ENST00000663964.1	1.212098	2.072004	2.722412	0.037419	0.208487	-3.96695
NONHSAT154367.1	-1.16431	1.665224	-2.72214	0.037432	0.208537	-3.9673
ENST00000653890.1	1.299191	2.924863	2.721607	0.037457	0.208585	-3.96802
lnc-URGCP-MRPS24-5:3	-1.06682	2.79586	-2.72122	0.037476	0.208676	-3.96853
NONHSAT224528.1	1.766497	3.33326	2.721095	0.037482	0.208683	-3.9687
NONHSAT205565.1	-1.08238	3.32587	-2.71938	0.037563	0.208848	-3.97097
lnc-FAM122C-3:1	1.444758	2.972334	2.719222	0.037571	0.208859	-3.97118
ENST00000510059.1	1.051611	3.280959	2.717268	0.037664	0.209156	-3.97377
NR_038844	-1.43957	3.063725	-2.71626	0.037712	0.209287	-3.97511
lnc-SALL2-2:3	-1.10049	3.55963	-2.71581	0.037734	0.209326	-3.9757
lnc-BARHL2-1:2	-1.0456	3.188041	-2.7156	0.037744	0.20936	-3.97599
NONHSAT198987.1	-1.06655	2.473524	-2.71543	0.037752	0.209381	-3.97621
NONHSAT202694.1	1.484461	4.18516	2.715176	0.037764	0.209438	-3.97655
MSTRG.46731.1	1.026404	3.554721	2.714419	0.0378	0.209508	-3.97755
ENST00000652712.1	-1.07673	2.084779	-2.71381	0.03783	0.209593	-3.97837
lnc-DHDH-1:1	-1.30416	2.00531	-2.71318	0.03786	0.209651	-3.9792
lnc-IKZF1-15:5	1.458978	3.08249	2.712619	0.037887	0.209703	-3.97994
ENST00000417513.1	-1.33708	2.661953	-2.71213	0.03791	0.209776	-3.98058
T042458	1.218126	2.849239	2.711975	0.037918	0.209783	-3.98079
NR_125389	-1.52211	3.00251	-2.71193	0.03792	0.209783	-3.98085
ENST00000664712.1	1.194351	2.265461	2.711536	0.037939	0.209877	-3.98138
ENST00000531379.1	-1.20459	2.374066	-2.71132	0.037949	0.2099	-3.98166

MSTRG.5887.3	1.123681	3.932884	2.709405	0.038042	0.210191	-3.98421
lnc-CD302-1:1	-1.20578	4.079417	-2.70906	0.038059	0.210236	-3.98467
lnc-ERICH1-11:1	1.1585	9.880765	2.708925	0.038065	0.210245	-3.98484
MSTRG.10303.8	-1.26222	1.718008	-2.70883	0.03807	0.210245	-3.98497
NONHSAT177953.1	1.133804	3.931021	2.708739	0.038074	0.210245	-3.98509
ENST00000561979.1	-1.0527	1.632702	-2.70823	0.038099	0.210291	-3.98577
NONHSAT188245.1	1.523442	6.087063	2.70812	0.038104	0.210293	-3.98591
lnc-PFKP-11:5	-1.2234	2.462145	-2.70812	0.038104	0.210293	-3.98591
NONHSAT171422.1	1.356097	4.557863	2.707258	0.038146	0.210454	-3.98706
NONHSAT149624.1	1.050677	4.14976	2.706275	0.038193	0.210579	-3.98836
ENST00000655799.1	1.488917	2.851105	2.706121	0.038201	0.210597	-3.98857
NONHSAT186991.1	-1.16155	1.772984	-2.70563	0.038225	0.210636	-3.98922
NONHSAT149731.1	1.116714	6.298148	2.705216	0.038245	0.210701	-3.98977
NONHSAT164752.1	1.010638	4.719446	2.704678	0.038271	0.210776	-3.99048
MSTRG.36569.1	-1.22929	3.494909	-2.70311	0.038347	0.210975	-3.99257
ENST00000301683.7	-1.45866	3.917071	-2.70308	0.038349	0.210975	-3.99261
ENST00000662918.1	1.437201	2.188098	2.702575	0.038373	0.211055	-3.99327
lnc-SMYD2-1:1	-1.23065	2.724728	-2.70256	0.038374	0.211055	-3.9933
lnc-LTN1-1:1	-1.20154	3.394386	-2.70237	0.038383	0.211067	-3.99355
NONHSAT187452.1	1.598452	2.99282	2.702046	0.038399	0.211136	-3.99398
lnc-HABP4-2:7	1.044356	7.940066	2.701999	0.038401	0.211137	-3.99404
MSTRG.21155.1	1.386516	2.732777	2.70173	0.038415	0.211187	-3.9944
lnc-BCAT1-6:1	1.071001	3.510869	2.701626	0.03842	0.211203	-3.99453
LINC02020:25	1.644641	3.132126	2.701352	0.038433	0.211242	-3.9949
ENST00000668827.1	1.503328	2.272705	2.701252	0.038438	0.211243	-3.99503
lnc-CUX2-1:1	-1.4322	2.760652	-2.70073	0.038464	0.211318	-3.99573
MSTRG.44627.1	-1.23964	1.753852	-2.70055	0.038472	0.211343	-3.99597
MSTRG.43249.1	1.071431	1.984961	2.700287	0.038485	0.211364	-3.99631
NONHSAT160653.1	1.629509	3.281055	2.69939	0.038529	0.211468	-3.9975
NONHSAT177546.1	1.081996	3.992653	2.699003	0.038548	0.211528	-3.99802
NONHSAT170034.1	1.205552	2.578718	2.698459	0.038575	0.211614	-3.99874
ENST00000660770.1	1.142752	2.270749	2.698355	0.03858	0.211622	-3.99888
lnc-TNK1-2:1	-1.38992	1.805049	-2.69824	0.038585	0.211623	-3.99903
T022079	1.157655	3.90012	2.698118	0.038591	0.211628	-3.99919
lnc-HECA-12:2	1.025559	3.147196	2.696942	0.038649	0.211753	-4.00076
lnc-CHIC1-7:1	1.204197	5.069862	2.696814	0.038655	0.211753	-4.00093
NONHSAT210535.1	2.052978	3.511781	2.696771	0.038657	0.211753	-4.00098
NONHSAT152756.1	1.291434	2.841505	2.696728	0.03866	0.211753	-4.00104
NR_126002	-1.63621	2.814971	-2.69662	0.038665	0.211753	-4.00119
lnc-ITGB8-12:1	-1.12379	4.136673	-2.69655	0.038668	0.211753	-4.00127
ENST00000586176.1	1.035979	2.40397	2.696369	0.038677	0.211776	-4.00152
lnc-MYT1L-1:4	1.658692	2.675408	2.696219	0.038685	0.211794	-4.00172
NONHSAT185618.1	1.016016	1.927888	2.695577	0.038716	0.211885	-4.00257
lnc-ZFP64-4:1	1.274743	3.730307	2.693986	0.038795	0.212143	-4.00468
lnc-ZDHHC7-3:1	1.04881	4.387323	2.693244	0.038831	0.212216	-4.00567
lnc-GBE1-5:8	-1.10467	3.144493	-2.69249	0.038869	0.212317	-4.00667
ENST00000606489.1	1.362881	8.668282	2.69117	0.038934	0.212508	-4.00842
NONHSAT156616.1	-1.0074	2.240257	-2.69098	0.038943	0.212517	-4.00867

lnc-SLC35G2-5:1	1.217987	3.362783	2.690927	0.038946	0.21252	-4.00875
lnc-FOXO4-3:1	1.337278	2.892023	2.69065	0.03896	0.212561	-4.00912
lnc-SERPINB1-1:10	-1.03196	3.970862	-2.69033	0.038976	0.212602	-4.00954
MSTRG.39327.26	1.202714	2.198413	2.690167	0.038984	0.212617	-4.00976
lnc-GSPT2-2:1	-1.19294	3.90395	-2.68986	0.038999	0.212672	-4.01017
NONHSAT221226.1	-1.15598	2.185138	-2.68936	0.039023	0.212737	-4.01082
ENST00000418426.1	1.353328	1.812563	2.689052	0.039039	0.212763	-4.01124
ENST00000648642.1	-1.31139	2.797498	-2.68892	0.039045	0.212786	-4.01141
lnc-C12orf74-5:5	1.078133	2.412678	2.688796	0.039052	0.21281	-4.01158
MSTRG.21408.1	1.54291	2.145559	2.688664	0.039058	0.212813	-4.01176
NONHSAT157688.1	1.448296	1.876518	2.688581	0.039062	0.212813	-4.01187
NONHSAT217091.1	1.046896	1.95304	2.687963	0.039093	0.21294	-4.01269
ENST00000657226.1	-1.02673	5.231741	-2.68793	0.039095	0.21294	-4.01273
NONHSAT153465.1	-1.52807	2.921592	-2.68763	0.03911	0.21301	-4.01313
NONHSAT193349.1	1.005943	5.071122	2.687343	0.039124	0.213065	-4.01351
NONHSAT209903.1	1.164132	2.052279	2.687151	0.039134	0.213106	-4.01377
MSTRG.47137.1	1.117682	4.521971	2.686879	0.039147	0.21314	-4.01413
NONHSAT193073.1	1.212712	2.904928	2.686854	0.039148	0.21314	-4.01416
NONHSAT210837.1	1.131012	1.653905	2.686759	0.039153	0.213143	-4.01429
ENST00000585243.1	-1.30313	4.657163	-2.68603	0.03919	0.213273	-4.01526
lnc-ANGPTL2-5:1	1.011318	11.56168	2.685706	0.039206	0.213329	-4.01569
ENST00000392385.2	1.350207	2.81678	2.685472	0.039217	0.213355	-4.016
ENST00000544089.1	1.42292	2.176923	2.685364	0.039223	0.213359	-4.01614
lnc-CSE1L-2:1	1.530806	3.198278	2.685038	0.039239	0.213396	-4.01658
MSTRG.65705.1	1.742845	3.330086	2.685022	0.03924	0.213396	-4.0166
ENST00000567624.1	-1.19776	2.959741	-2.68486	0.039248	0.213408	-4.01682
lnc-SLC9A6-1:3	-1.14699	2.931438	-2.68485	0.039248	0.213408	-4.01683
NONHSAT158271.1	1.555368	3.07264	2.684808	0.03925	0.213408	-4.01688
NONHSAT163195.1	1.212573	2.521541	2.684009	0.03929	0.213533	-4.01794
NONHSAT206663.1	1.021553	2.202608	2.683712	0.039305	0.213581	-4.01834
MSTRG.24169.1	-1.49283	3.059926	-2.68366	0.039308	0.213581	-4.01841
lnc-CAMKK2-2:1	-1.15287	3.768674	-2.68223	0.03938	0.213719	-4.02032
lnc-RNASEH2B-7:1	1.251883	2.092289	2.681359	0.039423	0.213829	-4.02147
NONHSAT152513.1	-1.09053	4.534618	-2.68078	0.039452	0.213909	-4.02224
lnc-EMILIN3-2:1	-1.06452	2.627581	-2.68071	0.039456	0.213912	-4.02232
MSTRG.53121.1	1.109179	2.865637	2.68042	0.03947	0.213924	-4.02272
NONHSAT156173.1	1.42379	2.479359	2.68006	0.039488	0.213999	-4.0232
NONHSAT216820.1	-1.08818	2.624179	-2.67996	0.039494	0.214015	-4.02333
NONHSAT189869.1	1.304033	2.285221	2.679869	0.039498	0.214028	-4.02345
lnc-SPRY4-1:1	1.134117	1.861094	2.679251	0.039529	0.214136	-4.02427
NONHSAT149441.1	-1.17308	3.997056	-2.67902	0.039541	0.214144	-4.02458
NONHSAT158086.1	1.495585	2.37996	2.678572	0.039563	0.214193	-4.02517
lnc-CACYBP-3:1	-1.11247	2.434825	-2.67795	0.039595	0.21428	-4.026
NONHSAT186509.1	1.185663	1.679918	2.677899	0.039597	0.21428	-4.02607
ENST00000414896.1	-1.44854	3.047062	-2.67601	0.039693	0.214531	-4.02859
NONHSAT169649.1	1.248651	3.557048	2.675876	0.0397	0.214555	-4.02876
NR_033977	1.213898	2.57129	2.6758	0.039703	0.214564	-4.02886
MSTRG.29072.1	-1.02656	3.831564	-2.67482	0.039753	0.214743	-4.03016

ENST00000617323.4	1.224346	3.641941	2.673365	0.039827	0.214942	-4.0321
MSTRG.71724.1	-1.34662	3.254915	-2.67319	0.039836	0.214942	-4.03234
ENST00000414098.2	-1.19449	2.775835	-2.67305	0.039843	0.214956	-4.03252
T314610	1.592173	3.089888	2.672725	0.039859	0.214985	-4.03295
lnc-SERPINI1-17:4	1.55547	3.088238	2.672432	0.039874	0.215	-4.03334
lnc-CPS1-5:2	1.311918	2.859304	2.672374	0.039877	0.215	-4.03342
NR_121639	1.297826	2.146964	2.67194	0.039899	0.21504	-4.034
NONHSAT187278.1	1.067326	2.141164	2.671723	0.03991	0.215077	-4.03428
NONHSAT187968.1	1.044411	2.548267	2.671701	0.039911	0.215077	-4.03431
NONHSAT178146.1	-1.78057	2.25875	-2.6712	0.039937	0.215179	-4.03498
NONHSAT166117.1	1.281812	2.20718	2.670934	0.03995	0.21523	-4.03534
NONHSAT207337.1	1.750237	2.700885	2.670392	0.039978	0.215343	-4.03606
NONHSAT197162.1	-1.11281	1.727482	-2.67002	0.039997	0.215378	-4.03656
MSTRG.52659.1	1.569768	2.878512	2.669253	0.040036	0.215518	-4.03757
ENST00000670979.1	-1.33628	2.612474	-2.66921	0.040038	0.215518	-4.03762
lnc-P4HA2-3:3	-1.13644	1.892654	-2.6685	0.040075	0.215634	-4.03857
NONHSAT164467.1	1.401186	2.665124	2.668437	0.040078	0.21564	-4.03866
NONHSAT168350.1	-1.20008	2.701482	-2.6675	0.040126	0.215795	-4.03991
NONHSAT151784.1	-1.289	2.336397	-2.66688	0.040158	0.215885	-4.04074
NONHSAT199944.1	1.675694	4.697678	2.666597	0.040172	0.215927	-4.04111
lnc-GAPT-9:5	-1.48439	2.962167	-2.66645	0.040179	0.215933	-4.0413
NONHSAT207923.1	1.088821	4.351333	2.666222	0.040191	0.215949	-4.04161
NONHSAT158216.1	1.462034	2.933063	2.666209	0.040192	0.215949	-4.04162
NONHSAT215132.1	1.414256	2.580139	2.666087	0.040198	0.215953	-4.04179
NONHSAT211798.1	1.11046	3.950748	2.665688	0.040219	0.215953	-4.04232
MSTRG.49528.3	1.891633	3.278827	2.665627	0.040222	0.215953	-4.0424
NONHSAT222629.1	1.355813	2.476254	2.665559	0.040225	0.215958	-4.04249
lnc-TIMM21-12:1	-1.64011	2.651365	-2.66522	0.040243	0.215958	-4.04294
NONHSAT167483.1	1.010088	7.266028	2.665165	0.040245	0.215958	-4.04301
NONHSAT218284.1	1.214868	5.427507	2.66514	0.040247	0.215958	-4.04305
NONHSAT186781.1	1.048219	4.32868	2.665072	0.04025	0.215958	-4.04314
NONHSAT182975.1	-1.3362	1.731533	-2.66494	0.040257	0.215958	-4.04332
NONHSAT168400.1	1.159754	2.61446	2.664906	0.040259	0.215958	-4.04336
NONHSAT149630.1	1.142639	2.00268	2.664505	0.040279	0.216021	-4.04389
NONHSAT186205.1	1.045087	2.118082	2.664443	0.040283	0.216027	-4.04397
ENST00000429282.5	1.41548	3.243825	2.664362	0.040287	0.216038	-4.04408
ENST00000652118.1	-1.04363	2.238955	-2.66422	0.040294	0.216066	-4.04428
NONHSAT173088.1	1.443546	2.469276	2.663836	0.040314	0.216125	-4.04478
NONHSAT200298.1	1.159682	2.188804	2.663785	0.040316	0.216128	-4.04485
ENST00000560446.1	1.814522	2.558917	2.663725	0.04032	0.216133	-4.04493
NONHSAT205518.1	1.232865	2.480317	2.663488	0.040332	0.216164	-4.04525
T226787	1.555576	4.097929	2.663058	0.040354	0.216259	-4.04582
lnc-COMMD6-1:1	-1.34401	3.361194	-2.66238	0.040389	0.216401	-4.04673
ENST00000652912.1	-1.14893	1.9525	-2.66173	0.040423	0.216481	-4.04759
MYCNUT:3	1.257194	2.360079	2.661637	0.040427	0.216491	-4.04771
lnc-PLXDC1-3:2	1.503348	3.142785	2.66157	0.040431	0.216498	-4.0478
NR_125920	1.608236	5.410829	2.660904	0.040465	0.216557	-4.04869
T338552	1.231862	3.902716	2.66059	0.040481	0.216557	-4.0491

NONHSAT174714.1	1.133068	2.510037	2.660505	0.040486	0.216557	-4.04922
T098648	-1.07692	1.787298	-2.66031	0.040495	0.216581	-4.04947
NONHSAT197598.1	1.290416	2.866061	2.65965	0.04053	0.216707	-4.05036
T192410	-1.10197	2.189654	-2.65837	0.040596	0.216911	-4.05206
NONHSAT195993.1	-1.03753	1.672608	-2.658	0.040615	0.217001	-4.05255
ENST00000648074.1	1.133198	3.144414	2.657862	0.040622	0.217006	-4.05274
NONHSAT211802.1	1.524435	2.418714	2.657666	0.040633	0.217026	-4.053
ENST00000574352.1	1.361702	2.895433	2.657434	0.040645	0.217067	-4.05331
MSTRG.49784.1	1.028593	2.668201	2.657244	0.040654	0.217085	-4.05356
ENST00000621730.1	-1.19585	2.653988	-2.65713	0.040661	0.217085	-4.05372
MSTRG.60809.21	1.799146	6.368067	2.657033	0.040665	0.217085	-4.05384
lnc-FH-1:1	1.108958	3.585323	2.656785	0.040678	0.217085	-4.05417
lnc-TRAPPC3L-3:1	-1.07737	4.55065	-2.65676	0.040679	0.217085	-4.0542
lnc-KLLN-4:1	-1.37105	2.870389	-2.65649	0.040694	0.217105	-4.05456
NONHSAT219566.1	-1.10758	3.32772	-2.65577	0.040731	0.217206	-4.05552
NONHSAT222150.1	1.361142	3.841467	2.655397	0.04075	0.217237	-4.05602
lnc-KIF18A-7:1	-1.35438	2.775956	-2.65537	0.040752	0.217237	-4.05605
lnc-SLC16A1-6:3	-1.83901	2.435222	-2.65508	0.040767	0.217255	-4.05644
MSTRG.36081.1	-1.10815	4.529259	-2.65425	0.04081	0.217356	-4.05755
ENST00000583062.1	1.377169	2.435125	2.654239	0.040811	0.217356	-4.05756
lnc-RDH11-5:1	-1.1732	2.046407	-2.65407	0.04082	0.217367	-4.05779
lnc-RBM23-3:2	-1.52526	3.194835	-2.65347	0.040851	0.217477	-4.05859
NONHSAT172078.1	1.097415	2.954341	2.653132	0.040869	0.217515	-4.05904
MSTRG.24145.1	1.259674	2.228958	2.652224	0.040916	0.217645	-4.06025
lnc-KCNH1-4:1	1.421796	2.614232	2.651969	0.040929	0.217677	-4.06059
ENST00000671418.1	-1.09274	1.625166	-2.65178	0.040939	0.217695	-4.06083
T022371	1.178169	4.824289	2.651064	0.040977	0.217833	-4.06179
ENST00000661911.1	1.079425	2.538791	2.651046	0.040978	0.217833	-4.06182
lnc-GLRX5-2:2	-1.41164	2.136711	-2.64935	0.041066	0.218119	-4.06408
NONHSAT207308.1	1.043028	6.701918	2.648426	0.041115	0.218246	-4.06531
lnc-UBP1-5:1	-1.22934	3.590334	-2.6484	0.041116	0.218246	-4.06534
lnc-CHST12-4:2	-1.42007	2.649419	-2.64821	0.041126	0.218275	-4.06559
NONHSAT158090.1	1.04522	9.965811	2.648171	0.041128	0.218276	-4.06565
LINC00474:4	1.086832	3.470603	2.647673	0.041155	0.218369	-4.06631
NONHSAT203167.1	1.11763	4.074932	2.647383	0.04117	0.218398	-4.0667
lnc-ZNF732-10:2	-1.14463	3.446394	-2.64592	0.041247	0.218607	-4.06865
NONHSAT164569.1	1.586269	2.346862	2.645591	0.041264	0.218645	-4.06908
NONHSAT193799.1	1.066417	5.840348	2.645101	0.04129	0.218719	-4.06974
NONHSAT153627.1	1.06521	1.97132	2.645021	0.041294	0.21873	-4.06984
NONHSAT197526.1	-1.18821	1.997924	-2.64452	0.041321	0.218824	-4.07052
lnc-PRDM2-8:1	1.154091	4.399731	2.64407	0.041345	0.218884	-4.07111
lnc-NMUR2-4:1	1.245581	3.000765	2.644013	0.041348	0.218884	-4.07119
MSTRG.69881.2	1.550689	3.942096	2.643714	0.041363	0.218916	-4.07159
NONHSAT187888.1	-1.02626	2.403402	-2.64362	0.041368	0.218916	-4.07171
NONHSAT202688.1	1.016129	2.815607	2.643473	0.041376	0.218922	-4.07191
lnc-DOC2B-2:2	1.123175	3.435631	2.643219	0.04139	0.21897	-4.07224
ENST00000659612.1	-1.05884	1.639805	-2.64238	0.041434	0.219124	-4.07336
NONHSAT159216.1	1.065128	4.944504	2.641441	0.041484	0.21924	-4.07461

lnc-ASB4-7:1	-1.17676	3.548248	-2.6414	0.041486	0.21924	-4.07467
MSTRG.16669.1	-1.10384	6.781655	-2.6409	0.041513	0.219322	-4.07534
MIF-AS1:5	-1.0445	1.922995	-2.64049	0.041534	0.219346	-4.07588
NONHSAT162938.1	1.002379	2.325786	2.640256	0.041547	0.219376	-4.07619
NONHSAT221961.1	1.245556	1.719017	2.640077	0.041556	0.219381	-4.07643
ENST00000663952.1	1.261901	4.050906	2.640038	0.041558	0.219381	-4.07648
MSTRG.61097.1	-1.02113	1.733409	-2.63997	0.041562	0.219384	-4.07657
ENST00000662076.1	1.270962	4.448362	2.639939	0.041564	0.219384	-4.07662
NONHSAT183366.1	-1.07856	2.43366	-2.63989	0.041566	0.219387	-4.07668
lnc-NTRK2-2:4	1.46658	2.406931	2.639516	0.041586	0.219427	-4.07718
lnc-MPEG1-4:1	1.328345	2.331131	2.639278	0.041599	0.219458	-4.0775
lnc-RNF38-1:3	-1.39333	2.652685	-2.63927	0.041599	0.219458	-4.07751
NONHSAT187328.1	1.122001	1.627903	2.638499	0.04164	0.219582	-4.07854
lnc-S100B-2:1	-1.14054	3.023845	-2.63801	0.041666	0.219622	-4.07919
lnc-DTWD1-4:1	-1.10363	2.391869	-2.63792	0.041671	0.219622	-4.07931
ENST00000514293.1	-1.17722	3.683313	-2.63759	0.041689	0.219624	-4.07975
NONHSAT166505.1	-1.05945	4.652422	-2.63699	0.041721	0.21965	-4.08055
MSTRG.67002.13	-1.05121	4.511962	-2.63669	0.041736	0.219673	-4.08094
NONHSAT201897.1	1.028539	6.285318	2.636241	0.041761	0.219746	-4.08155
lnc-FEV-4:1	-1.3683	5.838674	-2.63597	0.041775	0.219811	-4.08191
MSTRG.56757.1	1.335992	1.975541	2.635858	0.041781	0.219831	-4.08206
lnc-ARSG-3:1	1.006364	2.797573	2.635499	0.0418	0.219897	-4.08254
MSTRG.1889.1	-1.04972	2.039585	-2.63485	0.041835	0.220039	-4.0834
NONHSAT182761.1	1.225032	2.086875	2.634329	0.041863	0.220101	-4.0841
MSTRG.62617.1	1.074828	2.857294	2.633653	0.041899	0.220223	-4.085
ENST00000611626.1	1.010612	1.973059	2.633609	0.041902	0.220223	-4.08506
lnc-AADACL4-3:1	-1.42427	2.563495	-2.63336	0.041915	0.220248	-4.08539
NONHSAT199599.1	-1.34516	2.899202	-2.63259	0.041956	0.220419	-4.08642
lnc-TGFBR2-7:1	-1.14533	3.662816	-2.6322	0.041977	0.220447	-4.08694
ENST00000641922.1	1.528801	2.99044	2.631873	0.041995	0.220447	-4.08737
ENST00000448786.1	1.180314	2.728055	2.631508	0.042014	0.220529	-4.08786
NONHSAT160966.1	1.191584	3.477321	2.630517	0.042068	0.220687	-4.08918
lnc-DMRTA2-9:1	-1.13002	2.925359	-2.63042	0.042073	0.220687	-4.08931
lnc-PTGER4-1:2	1.769976	2.692121	2.630036	0.042094	0.220739	-4.08982
lnc-TBXT-4:1	1.177966	2.8302	2.629654	0.042114	0.220814	-4.09033
NONHSAT218595.1	-1.14696	1.983026	-2.62952	0.042121	0.220837	-4.09051
NONHSAT201317.1	1.024082	2.326088	2.628344	0.042185	0.221043	-4.09208
lnc-SENP2-1:2	-1.06453	2.397119	-2.62833	0.042186	0.221043	-4.0921
NONHSAT169902.1	1.106067	1.920706	2.6282	0.042193	0.221051	-4.09227
MSTRG.67637.2	-1.0577	3.577419	-2.62776	0.042217	0.221114	-4.09286
lnc-SLC44A3-2:1	1.27922	2.71535	2.627633	0.042223	0.221131	-4.09303
lnc-CLHC1-1:1	-1.15018	2.595577	-2.62707	0.042254	0.221202	-4.09379
lnc-BMP6-15:1	-1.04022	2.200808	-2.62572	0.042327	0.221383	-4.09558
NONHSAT190795.1	-1.30923	2.143266	-2.62535	0.042347	0.221403	-4.09607
lnc-ORAOV1-4:1	-1.47706	3.721059	-2.62532	0.042349	0.221403	-4.09611
ENST00000611182.1	-1.01095	3.604192	-2.62522	0.042354	0.221403	-4.09625
lnc-GUCY1A1-3:1	-1.00742	1.966975	-2.62516	0.042357	0.221403	-4.09632
NONHSAT195128.1	1.076188	2.389073	2.625158	0.042357	0.221403	-4.09633

NONHSAT162452.1	1.102244	3.390292	2.625094	0.042361	0.221403	-4.09642
NONHSAT178111.1	1.409356	3.377423	2.624552	0.04239	0.221455	-4.09714
NONHSAT218053.1	1.380957	6.547191	2.62441	0.042398	0.221455	-4.09733
lnc-MDM2-1:1	-1.03482	4.070314	-2.62412	0.042414	0.221507	-4.09771
lnc-GLRA1-1:3	-1.30608	3.157855	-2.62336	0.042455	0.221666	-4.09873
NONHSAT164011.1	-1.06844	2.21334	-2.62262	0.042496	0.221797	-4.09972
NONHSAT158248.1	1.836492	5.158307	2.621622	0.04255	0.22193	-4.10105
lnc-MAGEA1-2:1	1.442272	1.909269	2.62136	0.042564	0.221958	-4.1014
lnc-AGT-6:9	-1.04782	2.495521	-2.62132	0.042566	0.221958	-4.10145
lnc-GMDS-6:15	-1.2094	3.319656	-2.62093	0.042588	0.221966	-4.10197
lnc-VLDLR-2:1	-1.69715	1.95115	-2.62039	0.042617	0.222065	-4.10269
NONHSAT216115.1	1.543449	3.465922	2.619667	0.042657	0.222153	-4.10366
lnc-UBN2-1:1	-1.2277	2.174543	-2.6186	0.042715	0.222352	-4.10508
lnc-RHNO1-2:1	-1.02141	4.957399	-2.61781	0.042758	0.22251	-4.10614
NONHSAT214386.1	1.264453	4.98527	2.617412	0.04278	0.222576	-4.10667
NONHSAT205453.1	1.597521	5.44198	2.61635	0.042838	0.222755	-4.10809
lnc-KHNYN-12:1	1.48711	3.475506	2.616063	0.042854	0.222771	-4.10847
ENST00000429661.1	1.264553	2.155057	2.615968	0.042859	0.222783	-4.10859
MSTRG.45361.1	1.23272	7.309093	2.615906	0.042863	0.222786	-4.10868
NONHSAT154816.1	1.171842	2.805528	2.615825	0.042867	0.222786	-4.10879
ENST00000481312.1	-1.19722	2.573874	-2.61552	0.042884	0.222806	-4.10919
lnc-FADS6-1:2	1.06293	6.878023	2.614893	0.042918	0.222929	-4.11003
T271557	1.161706	2.688791	2.614661	0.042931	0.222962	-4.11034
NR_046114	1.286437	2.753151	2.613901	0.042973	0.223076	-4.11135
lnc-ANKRD10-7:1	-1.0709	1.872276	-2.61336	0.043003	0.223139	-4.11208
NONHSAT201160.1	1.429728	2.719403	2.613154	0.043014	0.22315	-4.11235
lnc-ANXA2-3:1	1.198227	4.363011	2.613093	0.043018	0.22315	-4.11243
MSTRG.35260.1	-1.04703	2.08966	-2.61309	0.043018	0.22315	-4.11244
ENST00000577420.1	-1.13885	2.812938	-2.61274	0.043037	0.223177	-4.1129
MSTRG.27436.1	1.175164	1.959505	2.612355	0.043058	0.223248	-4.11342
MSTRG.71676.1	1.169029	2.094511	2.612019	0.043077	0.223248	-4.11387
NONHSAT220855.1	1.521467	1.948507	2.611971	0.04308	0.223248	-4.11393
MSTRG.25522.1	1.236358	2.724597	2.611971	0.04308	0.223248	-4.11393
ENST00000589084.1	1.001594	4.705854	2.611948	0.043081	0.223248	-4.11396
T049721	1.508717	8.537879	2.611192	0.043123	0.223377	-4.11497
T171865	-1.26409	2.469554	-2.61112	0.043127	0.223377	-4.11507
MSTRG.8028.1	1.469626	3.037518	2.610584	0.043156	0.223427	-4.11578
ENST00000506612.1	1.363018	2.939966	2.61056	0.043158	0.223427	-4.11582
MSTRG.13818.1	1.168255	2.148113	2.610464	0.043163	0.22344	-4.11594
NONHSAT194402.1	1.208293	2.076361	2.609766	0.043202	0.22356	-4.11687
ENST00000661937.1	1.438797	2.416002	2.609418	0.043221	0.223625	-4.11734
lnc-NDC1-1:2	1.024116	2.725662	2.609078	0.04324	0.223654	-4.11779
ENST00000418218.5	1.474549	2.943955	2.608699	0.043261	0.223718	-4.1183
ENST00000515264.1	-1.1592	4.168332	-2.6087	0.043261	0.223718	-4.11831
NONHSAT165004.1	1.393378	2.324528	2.608273	0.043285	0.223777	-4.11887
MSTRG.10994.1	-1.23124	2.530423	-2.60816	0.043291	0.223793	-4.11903
NONHSAT154751.1	-1.33652	3.554722	-2.60771	0.043316	0.22384	-4.11962
ENST00000453180.5	-1.05396	2.153936	-2.60744	0.043331	0.223884	-4.11998

NONHSAT201577.1	1.073355	1.889293	2.607205	0.043344	0.223901	-4.1203
MSTRG.46673.1	1.076215	9.065996	2.60671	0.043371	0.223979	-4.12096
MSTRG.49234.1	-1.17787	2.597035	-2.6062	0.0434	0.224101	-4.12163
ENST00000630360.1	1.535587	3.316777	2.605936	0.043415	0.224141	-4.12199
lnc-LAPTM4B-4:1	2.079123	4.605081	2.605346	0.043447	0.224245	-4.12278
lnc-RGS18-9:1	1.087812	3.33521	2.604277	0.043507	0.224387	-4.12421
NONHSAT205598.1	1.329413	2.618804	2.604253	0.043508	0.224387	-4.12424
lnc-ZC3H12D-19:1	-1.01007	2.430503	-2.60393	0.043526	0.224431	-4.12467
lnc-GLUD2-12:1	-1.14703	2.770285	-2.60382	0.043533	0.224431	-4.12482
lnc-GLA-1:1	1.260678	3.816365	2.603361	0.043558	0.224478	-4.12543
KCNMA1-AS2:1	-1.11342	2.855783	-2.60318	0.043569	0.224503	-4.12568
lnc-DDX18-4:1	1.269262	3.333827	2.602926	0.043583	0.224544	-4.12601
lnc-USP9Y-11:1	1.209652	2.727569	2.602753	0.043592	0.224567	-4.12624
lnc-SLC2A3-4:1	1.654199	1.882039	2.602265	0.04362	0.22465	-4.12689
ENST00000663658.1	1.636242	2.576801	2.601919	0.043639	0.224716	-4.12736
ENST00000542197.1	1.410472	2.769461	2.601867	0.043642	0.22472	-4.12743
lnc-NIPAL1-2:1	1.716415	7.738251	2.601654	0.043654	0.224726	-4.12771
lnc-PAK2-2:1	-1.24195	3.199389	-2.60146	0.043665	0.224733	-4.12797
lnc-BLVRB-2:1	-1.56819	3.214602	-2.60065	0.04371	0.224886	-4.12905
ENST00000579673.1	1.339303	2.793683	2.600576	0.043714	0.224886	-4.12915
lnc-CDC5L-6:1	1.131897	4.703501	2.600533	0.043717	0.224886	-4.12921
MSTRG.9368.1	1.399616	4.449932	2.600091	0.043742	0.224957	-4.1298
NONHSAT204454.1	1.425491	3.884899	2.600051	0.043744	0.224957	-4.12985
ENST00000666509.1	-1.13536	3.106096	-2.59971	0.043763	0.22502	-4.13031
NONHSAT180688.1	1.271641	2.830469	2.599672	0.043765	0.22502	-4.13036
MSTRG.51158.1	1.570021	2.534042	2.599271	0.043788	0.225099	-4.13089
lnc-CNN1-1:1	1.156016	2.101119	2.599101	0.043797	0.225099	-4.13112
NONHSAT182156.1	1.130953	2.626683	2.599087	0.043798	0.225099	-4.13114
NONHSAT166449.1	1.313903	3.929046	2.599022	0.043802	0.225099	-4.13123
MSTRG.32782.1	-1.76181	3.372621	-2.59889	0.043809	0.225099	-4.1314
NONHSAT172134.1	1.442202	2.137892	2.598606	0.043825	0.225111	-4.13178
ENST00000504552.5	1.068805	3.803534	2.597576	0.043883	0.225298	-4.13316
NONHSAT161313.1	1.405097	3.640044	2.597539	0.043885	0.225298	-4.13321
NONHSAT213161.1	1.545709	3.026121	2.596758	0.043929	0.225444	-4.13425
MSTRG.18248.1	1.600867	3.68224	2.596663	0.043935	0.225455	-4.13438
lnc-LY6G5C-1:2	1.096302	8.539956	2.595293	0.044012	0.225653	-4.13621
lnc-KLHDC4-74:4	-1.3511	6.572006	-2.59508	0.044024	0.225673	-4.13649
lnc-PLCG1-2:1	-1.1834	7.803195	-2.59235	0.044179	0.225994	-4.14014
NONHSAT164883.1	-1.20592	3.220265	-2.59176	0.044212	0.226086	-4.14093
NONHSAT197672.1	1.403704	2.851142	2.590722	0.044271	0.226284	-4.14232
NONHSAT214369.1	1.181822	1.963134	2.590394	0.04429	0.226333	-4.14276
MSTRG.65639.1	1.23343	3.410941	2.59017	0.044303	0.226365	-4.14306
NONHSAT169761.1	1.029688	3.843628	2.589983	0.044313	0.226395	-4.14331
NONHSAT175877.1	-1.15679	2.396974	-2.58987	0.04432	0.226395	-4.14346
lnc-TM6SF1-4:1	1.065544	4.091122	2.589225	0.044357	0.226513	-4.14432
NONHSAT172930.1	-1.06484	4.068376	-2.58919	0.044359	0.226513	-4.14437
MSTRG.32507.1	1.139926	2.463593	2.588407	0.044403	0.226638	-4.14541
lnc-GJD4-1:1	1.492581	5.078633	2.588125	0.044419	0.226697	-4.14579

NONHSAT222034.1	1.062302	4.135719	2.587687	0.044444	0.226767	-4.14638
ENST00000556205.1	1.085676	2.632746	2.587219	0.044471	0.226835	-4.147
ENST00000662661.1	1.163941	3.524676	2.586643	0.044504	0.226925	-4.14777
MSTRG.10786.1	-1.13067	2.51294	-2.58664	0.044505	0.226925	-4.14778
lnc-BUB1-2:1	1.555538	2.539187	2.586158	0.044532	0.227008	-4.14842
NONHSAT194802.1	1.069266	1.60694	2.585345	0.044578	0.227136	-4.14951
NONHSAT212984.1	1.462753	2.949862	2.585153	0.044589	0.227174	-4.14976
MSTRG.30012.1	-1.01298	3.91761	-2.58409	0.04465	0.227351	-4.15119
ENST00000651142.1	1.884554	4.142341	2.584076	0.044651	0.227351	-4.1512
NONHSAT195105.1	1.035875	2.926169	2.583933	0.044659	0.227381	-4.15139
NONHSAT181360.1	1.04046	2.038191	2.583822	0.044666	0.227385	-4.15154
lnc-KIAA1551-8:2	1.357263	2.382038	2.583802	0.044667	0.227385	-4.15157
T292163	1.248398	3.080644	2.583362	0.044692	0.227475	-4.15216
NONHSAT160178.1	1.381849	2.700467	2.58263	0.044734	0.227602	-4.15314
NONHSAT201286.1	1.038394	3.66217	2.581855	0.044779	0.227714	-4.15417
NONHSAT200230.1	1.050672	6.695392	2.581736	0.044786	0.227737	-4.15433
NONHSAT167553.1	1.488022	3.575277	2.581426	0.044804	0.227783	-4.15475
T298925	-1.17847	5.098976	-2.58117	0.044818	0.22781	-4.15509
NONHSAT209990.1	1.088488	3.638881	2.581109	0.044822	0.227818	-4.15517
NONHSAT154604.1	1.219147	2.221576	2.580952	0.044831	0.227849	-4.15538
NONHSAT184200.1	1.332261	2.853217	2.580847	0.044837	0.227849	-4.15552
lnc-MYOCOS-4:1	-1.21697	3.538203	-2.58077	0.044842	0.227849	-4.15563
MSTRG.8224.1	-1.21302	4.041175	-2.58	0.044886	0.227994	-4.15666
lnc-CPEB2-17:1	1.237749	5.38103	2.578579	0.044968	0.228146	-4.15856
NONHSAT153532.1	-1.08164	2.033778	-2.57838	0.04498	0.228153	-4.15882
MSTRG.837.1	-1.03954	3.951072	-2.57832	0.044983	0.228153	-4.1589
lnc-RAB11A-4:1	1.077996	2.01795	2.578293	0.044985	0.228153	-4.15894
ENST00000503844.1	1.813632	3.039424	2.577962	0.045004	0.228224	-4.15938
ENST00000561254.1	-1.35761	2.548298	-2.57782	0.045012	0.22824	-4.15958
NONHSAT197128.1	-1.03356	3.167484	-2.57779	0.045014	0.22824	-4.15961
lnc-DEK-2:1	-1.05641	5.365397	-2.57741	0.045036	0.228336	-4.16011
NONHSAT223852.1	1.076906	8.469042	2.577058	0.045056	0.228375	-4.16059
NONHSAT174795.1	1.299241	2.060722	2.576455	0.045091	0.22844	-4.1614
MSTRG.32671.24	-1.08196	2.426525	-2.57645	0.045092	0.22844	-4.16141
NONHSAT177166.1	1.158127	2.918117	2.5756	0.045141	0.228523	-4.16254
lnc-VRK2-15:1	-1.31157	4.303346	-2.57489	0.045182	0.228582	-4.16349
ENST00000607844.1	-1.00797	5.890937	-2.57447	0.045207	0.228673	-4.16405
NONHSAT210164.1	1.346203	1.701833	2.573874	0.045241	0.228749	-4.16485
lnc-ERCC6L2-7:1	1.115675	4.908777	2.573761	0.045248	0.228749	-4.165
ENST00000669185.1	1.232625	1.771376	2.573304	0.045275	0.228833	-4.16561
ENST00000657981.1	1.889587	3.809989	2.572826	0.045302	0.228891	-4.16625
ENST00000655381.1	1.199972	2.071361	2.572549	0.045319	0.228912	-4.16662
lnc-LIFR-7:1	1.396019	2.913042	2.572505	0.045321	0.228912	-4.16668
NONHSAT209485.1	1.431973	2.990951	2.572388	0.045328	0.228924	-4.16684
lnc-PTPRZ1-12:1	1.591369	3.256232	2.572197	0.045339	0.228953	-4.16709
NONHSAT204264.1	-1.0928	2.94081	-2.57178	0.045364	0.229	-4.16765
lnc-TEX47-4:1	-1.364	2.571272	-2.57117	0.045399	0.229103	-4.16847
NONHSAT211608.1	1.029862	2.662441	2.570542	0.045436	0.229218	-4.16931

NONHSAT213189.1	-1.75404	4.290555	-2.56986	0.045476	0.229371	-4.17022
lnc-INPP5F-1:1	-1.15003	3.472658	-2.56871	0.045543	0.229574	-4.17176
lnc-RASEF-5:1	-1.7703	3.548323	-2.56835	0.045564	0.229645	-4.17223
lnc-SLC30A5-3:1	1.563468	9.373162	2.568249	0.04557	0.229664	-4.17237
ENST00000665225.1	1.941156	2.733743	2.567865	0.045593	0.229675	-4.17289
NONHSAT197729.1	1.31187	2.454403	2.567748	0.0456	0.229697	-4.17305
MSTRG.39541.1	1.31662	3.260061	2.567519	0.045613	0.229697	-4.17335
NONHSAT153338.1	1.227581	1.682359	2.566509	0.045673	0.229828	-4.1747
NONHSAT197283.1	-1.46749	2.48927	-2.566	0.045702	0.229894	-4.17538
MSTRG.44994.1	1.384067	8.355577	2.565412	0.045737	0.229992	-4.17617
T191042	1.339795	1.74472	2.565325	0.045742	0.229992	-4.17629
lnc-ADCY7-1:2	1.19255	2.22072	2.565023	0.04576	0.230058	-4.17669
NONHSAT214246.1	1.128412	2.311832	2.564961	0.045764	0.230064	-4.17677
ENST00000656677.1	1.019721	1.829234	2.564704	0.045779	0.230116	-4.17712
NONHSAT184146.1	1.601689	2.641111	2.564637	0.045783	0.230116	-4.17721
NONHSAT209205.1	1.831091	2.937716	2.564045	0.045818	0.230237	-4.178
MSTRG.52417.1	-1.06811	4.258146	-2.56401	0.04582	0.230237	-4.17805
ENST00000446081.5	1.389797	2.538503	2.563893	0.045827	0.230255	-4.1782
ENST00000657859.1	-1.565	3.664463	-2.56381	0.045831	0.230256	-4.17831
NONHSAT180289.1	1.327172	2.505342	2.563673	0.04584	0.230275	-4.1785
NONHSAT220997.1	-1.04488	2.450366	-2.56326	0.045864	0.230327	-4.17905
ENST00000648775.1	1.112818	7.793854	2.562981	0.045881	0.230354	-4.17943
T249506	1.703785	3.064593	2.562978	0.045881	0.230354	-4.17943
lnc-FIGNL1-4:1	1.368593	1.800408	2.562726	0.045896	0.230406	-4.17977
NONHSAT202513.1	-1.18973	2.025096	-2.56239	0.045916	0.230444	-4.18022
ENST00000431130.2	-1.20807	2.2632	-2.56233	0.045919	0.230444	-4.1803
MSTRG.49187.2	1.493292	3.259012	2.562186	0.045928	0.230464	-4.18049
NONHSAT214036.1	-1.12784	1.902726	-2.56165	0.04596	0.230567	-4.18121
NONHSAT160516.1	-1.20476	3.175637	-2.56128	0.045982	0.230619	-4.18171
NONHSAT187007.1	1.365425	2.895322	2.561205	0.045986	0.23062	-4.1818
NONHSAT190969.1	-1.03789	2.381748	-2.56079	0.046011	0.230675	-4.18236
NONHSAT159460.1	-1.02682	2.666223	-2.56039	0.046034	0.230735	-4.1829
lnc-HES1-8:1	-1.11506	2.817019	-2.56005	0.046054	0.230775	-4.18335
NONHSAT198795.1	-1.19429	2.040883	-2.56002	0.046056	0.230775	-4.18338
MSTRG.69589.1	1.144659	2.548991	2.56002	0.046056	0.230775	-4.18339
ENST00000602704.1	-1.32183	2.560967	-2.5599	0.046063	0.230776	-4.18355
T187581	1.397881	2.905797	2.559389	0.046094	0.230839	-4.18423
lnc-CCDC171-4:1	1.263564	4.337127	2.558674	0.046136	0.230958	-4.18519
ENST00000666528.1	1.443046	2.670978	2.558146	0.046168	0.231046	-4.1859
MSTRG.47445.1	-1.7569	2.952185	-2.55776	0.046191	0.231122	-4.18641
lnc-CHRNA4-4:2	1.091713	1.833232	2.557613	0.046199	0.231148	-4.18661
ENST00000423208.2	1.523893	2.99629	2.557264	0.04622	0.231194	-4.18708
MSTRG.51404.5	1.162784	2.078358	2.557167	0.046226	0.231194	-4.18721
NONHSAT177333.1	1.496272	2.987625	2.556357	0.046274	0.23135	-4.18829
NONHSAT208713.1	1.065322	1.709059	2.555889	0.046302	0.231395	-4.18892
T304975	-1.63426	3.823432	-2.55497	0.046357	0.231603	-4.19015
lnc-FCN1-4:1	-1.00954	4.620866	-2.55469	0.046374	0.231653	-4.19052
ENST00000597450.1	1.157832	6.270838	2.554407	0.046391	0.231663	-4.1909

NONHSAT198045.1	1.075138	2.417827	2.554269	0.046399	0.231664	-4.19109
lnc-LY86-11:1	1.033276	5.352157	2.554021	0.046414	0.231681	-4.19142
lnc-MB21D2-1:2	1.922065	3.794377	2.553669	0.046435	0.231739	-4.19189
LINC02542:7	-1.38879	2.944235	-2.55328	0.046458	0.231799	-4.19241
ENST00000559394.2	-1.01125	1.997213	-2.55268	0.046494	0.23193	-4.19322
MSTRG.29582.7	1.151319	1.638649	2.552651	0.046496	0.23193	-4.19325
NONHSAT158712.1	1.282009	1.726205	2.551747	0.04655	0.232128	-4.19447
NONHSAT218176.1	1.339777	2.940105	2.551552	0.046562	0.232128	-4.19473
NONHSAT166571.1	1.034921	3.251581	2.551151	0.046586	0.232196	-4.19526
lnc-PGRMC1-2:1	1.061552	3.456849	2.550654	0.046616	0.232264	-4.19593
NONHSAT173356.1	-1.19916	3.124009	-2.54948	0.046687	0.232393	-4.19751
MSTRG.230.1	1.23912	2.410153	2.549359	0.046694	0.232393	-4.19766
T247847	1.337854	2.911678	2.54893	0.04672	0.232423	-4.19824
ENST00000650029.1	1.244845	2.376693	2.548361	0.046754	0.232516	-4.199
lnc-HEY1-2:1	-1.06662	3.467911	-2.54815	0.046767	0.232545	-4.19928
lnc-ZG16-4:1	1.189257	10.23806	2.548014	0.046775	0.232573	-4.19947
NONHSAT149376.1	1.033599	3.439885	2.547981	0.046777	0.232573	-4.19951
NONHSAT190081.1	1.016638	3.491014	2.547799	0.046788	0.232593	-4.19975
lnc-ZNF217-2:3	1.651463	3.01456	2.546377	0.046874	0.232871	-4.20166
LLPH-DT:9	-1.04754	1.965681	-2.54572	0.046914	0.232932	-4.20254
NONHSAT190384.1	1.402058	2.129883	2.545499	0.046927	0.232943	-4.20283
ENST00000625268.2	1.186048	3.227781	2.545314	0.046938	0.232973	-4.20308
NONHSAT222449.1	1.478197	2.593919	2.54495	0.04696	0.233048	-4.20357
lnc-WASF3-1:1	1.371965	2.960946	2.54433	0.046998	0.233211	-4.2044
lnc-QKI-8:1	1.002364	1.954949	2.544288	0.047001	0.233211	-4.20446
ENST00000430468.1	1.325634	2.228575	2.543852	0.047027	0.233281	-4.20504
lnc-COLGALT2-2:1	1.447493	2.986913	2.543627	0.047041	0.233308	-4.20534
NONHSAT154277.1	1.402184	2.924856	2.54309	0.047073	0.233401	-4.20606
lnc-NDUFA10-8:2	1.047141	4.756812	2.542968	0.047081	0.233415	-4.20622
lnc-PTPRZ1-8:1	-1.14481	2.03207	-2.54269	0.047098	0.233442	-4.2066
lnc-LSM7-3:1	1.173588	3.830517	2.542518	0.047108	0.233482	-4.20683
lnc-AK3-5:2	1.496473	3.05278	2.541735	0.047156	0.233615	-4.20788
ENST00000648967.1	-1.00308	3.04261	-2.54147	0.047172	0.233655	-4.20823
NONHSAT155872.1	1.41224	2.524478	2.541449	0.047173	0.233655	-4.20826
lnc-ACVR1-4:1	1.480966	2.526654	2.541016	0.0472	0.233763	-4.20884
lnc-DAD1-4:1	1.17346	2.680314	2.540715	0.047218	0.233807	-4.20924
NONHSAT182326.1	-1.34008	3.181968	-2.54007	0.047257	0.233946	-4.21011
lnc-SAP30-2:1	1.560574	2.569185	2.538669	0.047343	0.234182	-4.21198
ENST00000669903.1	1.164148	2.79322	2.538413	0.047359	0.23422	-4.21233
lnc-SEM1-4:1	-1.07003	3.458557	-2.53828	0.047366	0.234233	-4.2125
lnc-HERPUD2-8:1	1.250336	4.362447	2.538213	0.047371	0.234242	-4.2126
MSTRG.41630.1	-1.0065	2.498504	-2.53758	0.04741	0.234366	-4.21345
NONHSAT171942.1	-1.03868	2.386362	-2.53664	0.047467	0.234536	-4.21471
T356680	-1.28579	2.704864	-2.53623	0.047492	0.234608	-4.21525
lnc-PSMA8-5:3	-1.29371	4.431028	-2.53595	0.047509	0.234658	-4.21563
NONHSAT165560.1	-1.08141	2.532245	-2.53569	0.047525	0.234696	-4.21597
ENST00000419499.1	1.305035	3.868532	2.535354	0.047546	0.234725	-4.21643
NONHSAT162148.1	1.109851	6.09696	2.535118	0.047561	0.234748	-4.21674

MSTRG.4118.1	-1.22052	2.309117	-2.53504	0.047565	0.234748	-4.21685
NONHSAT163671.1	1.358225	2.96243	2.534988	0.047569	0.234748	-4.21692
NONHSAT214047.1	-1.23265	2.515348	-2.53491	0.047574	0.234748	-4.21703
lnc-RGPD4-5:1	-1.15038	1.679757	-2.53419	0.047618	0.234865	-4.21799
lnc-SLC4A1-2:1	-1.13846	2.5663	-2.5339	0.047636	0.23493	-4.21838
lnc-ERGIC1-2:2	-1.11169	3.787961	-2.5337	0.047648	0.234932	-4.21864
lnc-CNTNAP3B-14:1	1.488723	3.120315	2.532521	0.04772	0.235211	-4.22022
MSTRG.6162.1	-1.09545	2.933233	-2.53218	0.047741	0.23529	-4.22068
lnc-UBE2R2-1:1	1.007351	3.227587	2.531942	0.047756	0.235329	-4.221
ENST00000432706.1	1.162712	3.591413	2.531319	0.047795	0.235429	-4.22184
ENST00000657062.1	-1.03365	2.152646	-2.53128	0.047797	0.235429	-4.22189
NONHSAT186713.1	-1.03291	2.102629	-2.53101	0.047813	0.235472	-4.22224
NONHSAT158528.1	-1.32269	2.685239	-2.53017	0.047866	0.235515	-4.22338
ENST00000421604.1	-1.1745	3.75949	-2.52993	0.04788	0.235561	-4.22369
MSTRG.27510.1	1.403973	2.942022	2.52963	0.047899	0.235601	-4.2241
NONHSAT187942.1	1.272195	2.039199	2.529387	0.047914	0.235601	-4.22442
MSTRG.16317.1	1.088153	3.67804	2.528637	0.047961	0.23575	-4.22543
NONHSAT217770.1	1.137376	1.649925	2.528613	0.047962	0.23575	-4.22546
ENST00000661295.1	1.059665	1.900356	2.528149	0.047991	0.235841	-4.22608
MSTRG.45254.1	-1.41742	2.133464	-2.52762	0.048024	0.23591	-4.22679
ENST00000578539.1	-1.01597	3.981687	-2.52708	0.048058	0.236035	-4.22752
lnc-TRMT13-2:1	-1.47363	2.953819	-2.52702	0.048061	0.236036	-4.2276
ENST00000661365.1	1.239564	2.193454	2.526753	0.048078	0.23608	-4.22796
NONHSAT219922.1	1.248297	2.256672	2.52647	0.048095	0.236131	-4.22834
NR_120463	1.016929	1.767781	2.526365	0.048102	0.236131	-4.22848
MSTRG.48929.1	1.400525	2.416272	2.526333	0.048104	0.236131	-4.22852
NONHSAT177887.1	1.11233	2.514925	2.526013	0.048124	0.236194	-4.22895
lnc-PPIL4-9:1	1.175493	3.551656	2.52549	0.048156	0.236273	-4.22965
ENST00000621825.1	-1.1234	1.837691	-2.52543	0.04816	0.23628	-4.22973
ENST00000418471.1	1.093702	1.979775	2.525341	0.048166	0.236296	-4.22985
NONHSAT153555.1	-1.15436	3.729442	-2.52505	0.048184	0.236351	-4.23024
lnc-AMIGO1-2:1	-1.53744	3.323432	-2.52495	0.04819	0.236351	-4.23037
NONHSAT162403.1	1.449409	2.408393	2.524681	0.048207	0.236371	-4.23073
lnc-KATNA1-2:2	-1.17018	4.075064	-2.52333	0.048291	0.236478	-4.23254
NONHSAT173036.1	1.149125	2.156773	2.522463	0.048345	0.236625	-4.23371
PTOV1-AS1:8	-1.1057	3.523348	-2.52246	0.048345	0.236625	-4.23371
MSTRG.23195.1	1.406967	2.875679	2.522168	0.048364	0.236666	-4.2341
lnc-ZCCHC2-2:1	1.028233	2.404494	2.521749	0.04839	0.236687	-4.23467
lnc-FAM84B-3:1	-1.15911	3.158008	-2.52118	0.048426	0.236811	-4.23543
MSTRG.25638.1	-1.03811	3.452843	-2.52057	0.048464	0.236905	-4.23624
MSTRG.70718.1	1.134224	2.151399	2.518794	0.048575	0.237249	-4.23863
NONHSAT159195.1	1.860168	2.303499	2.518617	0.048586	0.237277	-4.23887
MSTRG.29630.1	1.276424	3.425961	2.518304	0.048606	0.237325	-4.23929
MSTRG.33677.10	1.181204	8.557936	2.517948	0.048629	0.237375	-4.23976
NONHSAT161216.1	1.195797	1.97977	2.517455	0.04866	0.237452	-4.24042
NONHSAT194338.1	1.097265	2.453757	2.515974	0.048753	0.237709	-4.24241
MSTRG.61301.1	1.081061	3.367357	2.515773	0.048766	0.237748	-4.24268
NONHSAT218474.1	1.551395	8.571469	2.515514	0.048782	0.237774	-4.24303

T035679	1.168504	2.165593	2.515499	0.048783	0.237774	-4.24305
lnc-SLC25A29-2:1	-1.11809	1.672353	-2.51446	0.048848	0.237943	-4.24443
NONHSAT181428.1	1.710682	3.60735	2.513295	0.048922	0.238166	-4.246
ENST00000655326.1	1.279227	1.907987	2.512987	0.048942	0.238215	-4.24642
NONHSAT167074.1	-1.41094	1.81357	-2.5128	0.048954	0.238262	-4.24667
ENST00000656125.1	1.185779	2.600566	2.512403	0.048979	0.238361	-4.2472
ENST00000666926.1	-1.28987	3.096932	-2.51158	0.049031	0.238557	-4.2483
MSTRG.25769.1	1.098315	2.100864	2.510797	0.049081	0.23873	-4.24936
MSTRG.13494.1	-1.37945	2.612345	-2.51066	0.04909	0.238759	-4.24954
lnc-GHSR-3:1	-1.46581	4.11015	-2.51054	0.049097	0.238762	-4.24969
T034239	1.239753	2.120361	2.510073	0.049127	0.238839	-4.25033
NONHSAT154548.1	-1.1197	2.512979	-2.50915	0.049186	0.238989	-4.25156
MSTRG.26757.1	1.251518	4.615138	2.508862	0.049204	0.239029	-4.25195
NONHSAT191639.1	1.429884	2.332123	2.508754	0.049211	0.239051	-4.2521
NONHSAT162315.1	1.712188	3.090174	2.508647	0.049218	0.239061	-4.25224
lnc-F8-2:1	-1.26097	2.796886	-2.50793	0.049264	0.239146	-4.25321
NONHSAT197981.1	1.303831	2.274407	2.507409	0.049297	0.239233	-4.2539
lnc-GPR132-1:1	1.740376	2.642195	2.506974	0.049325	0.239328	-4.25448
ENST00000506895.1	-1.29626	3.218813	-2.50686	0.049332	0.239329	-4.25464
ENST00000530572.1	-1.34728	2.184656	-2.50677	0.049337	0.239331	-4.25475
MSTRG.42849.1	-1.00477	4.18815	-2.50643	0.049359	0.23938	-4.25521
ADGRD1-AS1:3	1.426466	2.412333	2.506304	0.049367	0.239396	-4.25538
ENST00000668381.1	1.162894	4.709338	2.505998	0.049387	0.239445	-4.25579
lnc-IL2RA-7:1	-1.28975	2.656816	-2.50528	0.049433	0.239602	-4.25676
NONHSAT169894.1	1.229402	1.943178	2.505233	0.049436	0.239602	-4.25682
NONHSAT174308.1	1.10003	2.142538	2.505077	0.049446	0.239627	-4.25703
NONHSAT207179.1	-1.15698	2.044942	-2.50503	0.049449	0.239631	-4.2571
ENST00000419226.5	1.061821	2.612705	2.504828	0.049462	0.239636	-4.25736
MSTRG.7128.1	-1.13117	3.826216	-2.50447	0.049485	0.239677	-4.25784
lnc-GLTP-1:1	1.09016	2.599357	2.503078	0.049574	0.239902	-4.25971
lnc-PDZD8-6:1	-1.18126	4.000295	-2.50307	0.049575	0.239902	-4.25973
lnc-TYW1-6:1	1.073507	6.822025	2.503062	0.049575	0.239902	-4.25973
NONHSAT180765.1	1.197582	2.943265	2.502737	0.049596	0.239974	-4.26017
NONHSAT196222.1	1.087424	2.695904	2.502418	0.049617	0.240051	-4.2606
NONHSAT199055.1	-1.33907	1.819537	-2.50232	0.049623	0.240071	-4.26073
lnc-PPP4R3C-2:2	-1.19786	2.793207	-2.50175	0.04966	0.240155	-4.26149
MSTRG.13807.1	-1.02925	2.549731	-2.50169	0.049663	0.240155	-4.26157
lnc-ZNF502-1:1	-1.0424	3.752993	-2.50146	0.049678	0.240157	-4.26188
NONHSAT218765.1	1.451001	1.847846	2.500541	0.049738	0.240301	-4.26312
ENST00000667541.1	1.102322	2.102137	2.499763	0.049788	0.240429	-4.26416
lnc-ATP6V0A4-1:1	-1.38312	3.386375	-2.49966	0.049794	0.240449	-4.2643
MSTRG.38762.1	-1.06642	3.709254	-2.49945	0.049808	0.240495	-4.26459
NONHSAT188295.1	-1.46282	3.379928	-2.49944	0.049809	0.240495	-4.26459
MSTRG.41172.1	-1.09282	2.400482	-2.49897	0.049839	0.240547	-4.26523
NONHSAT202187.1	1.001595	2.433013	2.498556	0.049866	0.240621	-4.26578
NONHSAT204864.1	-1.40153	2.129543	-2.49848	0.049871	0.240632	-4.26588
NR_033752	1.165792	8.918187	2.49814	0.049893	0.240704	-4.26634
ENST00000665842.1	1.28689	2.530144	2.497462	0.049937	0.240816	-4.26725

lnc-THEMIS-4:1	-2.13651	3.773792	-2.49732	0.049946	0.240822	-4.26744

Table S5. Identification of different proteins in the HGDILnc1 pull-down group compared to the antisense HGDILnc1 pull-down by Mass spectrometry

Protein Group	Protein ID	Accession	-10lgP	Coverage (%)	Coverage (%) Sample 1	Area Sample 1	#Peptides	#Unique	#Spec Sample 1	Avg. Mass
127	164	P06733	154.75	23	23	7.07E+06	6	6	12	47169
5	42	P62736	146.49	25	25	0	15	1	69	42009
100	109	Q13085	144.59	7	7	1.76E+06	14	14	14	265551
58	170	P0CG39	142.27	9	9	8.60E+04	8	1	33	117390
178	145	Q71U36	128.45	20	20	1.35E+05	7	1	8	50136
173	268	O60812	117.29	20	20	0	6	1	8	32142
173	267	B7ZW38	117.29	20	20	0	6	1	8	32029
173	266	P0DMR1	117.29	20	20	0	6	1	8	32029
115	154	Q96I24	115.41	20	20	1.02E+06	11	6	14	61640
276	272	P12273	114.89	36	36	8.05E+05	4	4	4	16572
174	265	B2RXH8	113.6	20	20	1.22E+05	6	1	8	32072
285	370	P07148	106.67	36	36	2.23E+06	3	3	4	14208
195	200	P61604	102.98	62	62	7.62E+06	7	7	7	10932
212	238	Q96PU8	101.38	21	21	1.15E+06	5	5	6	37671
247	341	P05387	99.87	34	34	1.39E+06	3	3	4	11665
235	244	P12235	98.02	16	16	5.68E+04	5	1	5	33065
296	393	P31948	89	9	9	1.22E+05	3	3	3	62639
211	378	P62937	88.82	19	19	2.26E+06	3	3	5	18012
306	375	P24534	87.14	16	16	7.46E+05	3	3	3	24764
167	369	P62807	86.96	26	26	2.33E+05	3	1	9	13906
167	368	P57053	86.96	26	26	2.33E+05	3	1	9	13944
167	367	Q99877	86.96	26	26	2.33E+05	3	1	9	13922
167	366	Q5QNW6	86.96	26	26	2.33E+05	3	1	9	13920
167	365	Q93079	86.96	26	26	2.33E+05	3	1	9	13892

167	364	P58876	86.96	26	26	2.33E+05	3	1	9	13936
167	363	Q99879	86.96	26	26	2.33E+05	3	1	9	13989
167	362	O60814	86.96	26	26	2.33E+05	3	1	9	13890
167	361	Q99880	86.96	26	26	2.33E+05	3	1	9	13952
320	340	P25311	86	11	11	8.98E+05	3	3	3	34259
268	301	P00558	83.63	14	14	3.48E+05	4	4	4	44615
271	351	Q92879	81.97	7	7	2.26E+06	3	3	3	52063
275	474	P07737	81.16	21	21	3.47E+06	2	2	4	15054
213	258	P14923	80.84	6	6	4.29E+05	4	4	5	81745
186	311	Q14204	78.27	2	2	4.86E+05	5	5	5	532412
201	533	P49321	78.04	12	12	7.21E+05	2	2	7	85238
360	459	P29692	76.21	9	9	2.89E+05	2	2	2	31122
277	426	P07339	74.75	15	15	2.08E+05	3	3	3	44552
286	389	P27348	74.61	13	13	5.17E+05	3	2	4	27764
327	417	P62995	74.05	10	10	2.45E+05	2	2	2	33666
449	715	Q9Y5S9	73.97	15	15	1.67E+06	1	1	2	19889
336	675	O95777	73.43	25	25	1.38E+06	1	1	3	10403
352	478	Q9BQ39	73.07	4	4	1.92E+04	2	1	2	82565
299	339	P02788	72.94	4	4	1.14E+05	3	1	3	78182
566	716	P02753	72.76	11	11	7.29E+04	1	1	1	23010
450	717	Q14257	71.35	6	6	9.96E+04	1	1	2	36876
364	515	Q01105	71.01	5	5	2.64E+05	1	1	1	33489
364	523	P0DME0	71.01	5	5	2.64E+05	1	1	1	34882
420	678	Q9NRR5	69.66	3	3	6.76E+05	1	1	2	63853
420	718	Q9UMX0	69.66	3	3	6.76E+05	1	1	2	62519
420	719	Q9UHD9	69.66	3	3	6.76E+05	1	1	2	65696
335	543	P60903	69.64	28	28	1.55E+06	2	2	3	11203
451	720	Q9HAF1	68.73	12	12	2.26E+05	1	1	2	21635

432	557	O00299	68.6	15	15	1.13E+05	2	2	2	26923
274	412	O14556	68.46	6	6	0	2	1	3	44501
330	472	Q86SE5	67.96	7	7	1.16E+05	2	1	3	32331
510	672	O95865	67.59	7	7	2.47E+04	1	1	1	29644
280	382	Q9UNX3	67.38	17	17	1.76E+06	4	4	4	17256
511	617	P13667	67.32	3	3	8.11E+04	1	1	1	72933
302	402	Q13201	67.32	2	2	2.35E+04	2	2	2	138110
311	595	P55795	67.01	6	6	4.35E+05	2	1	3	49264
376	486	P26368	66.82	6	6	1.53E+05	2	2	2	53501
303	413	Q12996	66.14	3	3	4.69E+05	2	2	2	82922
220	410	Q9UPN3	66.02	0	0	7.89E+04	2	2	2	838323
419	674	P63173	64.47	19	19	1.27E+05	2	2	2	8218
422	721	Q9NQ50	64.38	9	9	5.87E+05	1	1	2	24490
567	722	P45973	63.99	8	8	1.01E+05	1	1	1	22225
456	605	O75822	63.76	10	10	7.35E+04	1	1	1	29062
483	620	Q13404	63.72	14	14	1.84E+05	1	1	1	16495
483	723	Q15819	63.72	14	14	1.84E+05	1	1	1	16363
399	494	P57721	63.37	7	7	5.01E+05	2	1	2	39465
97	229	P17066	63.22	5	5	0	5	1	5	71028
506	641	P09382	63.21	12	12	3.61E+05	1	1	1	14716
568	724	O60927	62.8	20	20	4.43E+05	1	1	1	13952
252	725	Q13185	61.65	9	9	2.30E+06	1	1	5	20811
252	726	P83916	61.65	9	9	2.30E+06	1	1	5	21418
452	727	Q9NWV4	59.73	20	20	1.90E+05	1	1	2	18048
110	499	P22090	59.33	10	10	1.07E+05	3	1	4	29456
429	544	P42224	57.43	4	4	2.49E+05	2	2	2	87335
569	728	Q92922	56.49	3	3	3.40E+04	1	1	1	122867
570	729	P41208	55.73	10	10	2.36E+05	1	1	1	19738

513	680	Q6P587	55.5	7	7	3.98E+04	1	1	1	24843
288	397	P33240	54.29	5	5	1.97E+05	3	2	4	60959
408	527	Q9Y265	53.97	6	6	1.63E+04	2	2	2	50228
361	469	Q04637	53.27	1	1	1.89E+05	2	2	2	175490
571	733	P20810	48.9	3	3	3.90E+04	1	1	1	76573
443	626	Q96CT7	48.05	6	6	5.95E+04	1	1	1	25835
403	518	P42285	48.03	2	2	3.41E+04	2	2	2	117805
341	414	P07237	47.68	5	5	3.67E+05	3	3	3	57116
573	735	O15371	47.6	2	2	7.49E+04	1	1	1	63973
574	736	P62857	46.79	16	16	5.89E+04	1	1	1	7841
405	520	Q99439	46.23	6	6	3.36E+05	2	1	2	33697
575	737	P49720	45.4	9	9	0	1	1	1	22949
576	739	A6NKH3	45.38	9	9	3.34E+05	1	1	1	10583
346	609	P06276	44.84	5	5	0	2	1	3	68418
516	686	Q8IVM0	44.8	4	4	4.21E+04	1	1	1	35822
477	594	P42167	44.49	6	6	1.52E+05	1	1	1	50670
366	569	P17096	44.31	26	26	1.56E+06	2	2	2	11676
577	740	P49458	44.17	13	13	2.15E+05	1	1	1	10112
515	685	O75368	42.84	16	16	2.98E+04	1	1	1	12774
382	526	Q14694	42.18	3	3	1.08E+05	2	2	2	87134
582	745	Q96P63	41.62	4	4	6.98E+04	1	1	1	46276
400	501	Q96C19	41.27	7	7	4.83E+05	2	2	2	26697
440	601	P31483	41.11	7	7	3.74E+05	2	2	2	42963
583	748	P12277	40.14	4	4	2.43E+05	1	1	1	42644
517	687	Q99714	39.78	8	8	5.31E+04	1	1	1	26923
585	751	Q14498	39.02	2	2	2.33E+04	1	1	1	59380
586	753	P17081	38.92	5	5	3.66E+05	1	1	1	22659
383	553	Q8IZP2	38.26	10	10	1.75E+05	2	2	2	27407

383	555	P50502	38.26	6	6	1.75E+05	2	2	2	41332
383	623	Q8NFI4	38.26	6	6	1.75E+05	2	2	2	41378
590	760	P02042	38.24	7	7	2.24E+05	1	1	1	16055
590	759	P69891	38.24	7	7	2.24E+05	1	1	1	16140
591	765	Q5JTV8	37.44	2	2	5.90E+04	1	1	1	66248
593	768	P51149	35.93	6	6	2.28E+04	1	1	1	23490
520	698	Q08J23	35.53	1	1	1.61E+05	1	1	1	86471
277	13035	P35321	35.37	9	9	1.65E+05	1	1	1	9877
277	13034	P22528	35.37	9	9	1.65E+05	1	1	1	9888
484	622	O94979	35.28	1	1	1.20E+05	1	1	1	133015
444	654	O75223	35.18	6	6	1.16E+05	1	1	1	21008
363	504	Q9UJ83	35.15	4	4	1.76E+05	2	2	2	63729
595	773	Q9Y295	34.93	3	3	1.12E+05	1	1	1	40542
491	658	P48594	34.59	3	3	3.20E+04	1	1	1	44854
491	657	P29508	34.59	3	3	3.20E+04	1	1	1	44565
491	700	P30740	34.59	3	3	3.20E+04	1	1	1	42742
491	701	P50452	34.59	3	3	3.20E+04	1	1	1	42767
491	702	O75830	34.59	2	2	3.20E+04	1	1	1	46145
491	776	P50453	34.59	3	3	3.20E+04	1	1	1	42404
414	660	Q8TAV4	34.3	5	5	9.05E+05	2	2	2	32135
156	397	Q14141	33.93	4	4	5.20E+04	2	1	3	49717
156	496	Q92599	33.93	3	3	5.20E+04	2	1	3	55756
370	559	P49591	33.91	2	2	1.09E+05	1	1	1	58777
597	777	P84022	33.86	3	3	6.55E+04	1	1	1	48081
597	779	Q15797	33.86	2	2	6.55E+04	1	1	1	52260
597	778	Q99717	33.86	2	2	6.55E+04	1	1	1	52258
597	781	O15198	33.86	2	2	6.55E+04	1	1	1	52493
597	780	Q15796	33.86	2	2	6.55E+04	1	1	1	52306

598	785	O15131	33.3	2	2	4.91E+04	1	1	1	60349
598	784	O60684	33.3	2	2	4.91E+04	1	1	1	60030
598	786	P52294	33.3	2	2	4.91E+04	1	1	1	60222
521	704	Q92552	32.69	3	3	1.35E+04	1	1	1	47611
228	808	P10599	32.51	9	9	7.07E+04	1	1	1	11737
600	798	P50579	32.34	3	3	2.20E+04	1	1	1	52892
601	803	P21108	32.08	4	4	4.66E+04	1	1	1	34839
601	802	P11908	32.08	4	4	4.66E+04	1	1	1	34769
601	801	P60891	32.08	4	4	4.66E+04	1	1	1	34834
355	602	B2RPK0	31.92	11	11	1.28E+06	2	2	2	24238
172	648	P05165	31.83	2	2	5.60E+04	2	2	2	80059
226	13037	P17858	31.77	1	1	1.70E+05	1	1	1	85018
226	6751	P08237	31.77	1	1	1.70E+05	1	1	1	85183
523	712	P33176	30.81	2	2	5.46E+04	1	1	1	109685
469	651	Q13561	30.74	4	4	0	1	1	1	44231
467	630	Q15393	30.67	1	1	4.68E+04	1	1	1	135577
524	713	Q13418	30.57	2	2	1.00E+05	1	1	1	51419
453	838	Q13425	30.56	2	2	7.68E+04	1	1	1	57950
604	833	O43324	30.54	6	6	8.19E+04	1	1	1	19811
605	834	P31944	30.1	4	4	1.24E+05	1	1	1	27680
610	846	P55327	29.2	14	14	2.33E+05	1	1	1	24327
493	764	Q9BVV6	29.14	1	1	0	1	1	1	169306
518	691	Q13595	28.44	3	3	4.73E+04	1	1	1	32689
527	794	Q96AC1	27.9	1	1	7.90E+04	1	1	1	77861
197	128	Q9BVA1	27.51	2	2	2.10E+05	1	1	1	49953
197	127	Q13885	27.51	2	2	2.10E+05	1	1	1	49907
197	240	A6NNZ2	27.51	2	2	2.10E+05	1	1	1	49573
197	236	Q3ZCM7	27.51	2	2	2.10E+05	1	1	1	49776

197	504	Q9H4B7	27.51	2	2	2.10E+05	1	1	1	50327
284	13045	P50461	27.14	5	5	0	1	1	1	20969
470	661	Q14966	26.38	0	0	1.17E+06	1	1	1	220623
612	873	O95400	26.21	7	7	2.38E+04	1	1	1	37646
613	874	Q5SSJ5	26.12	2	2	3.42E+04	1	1	1	61207
191	2246	Q8IWZ3	26.09	0	0	1.90E+04	1	1	1	269455
615	876	P42765	26.05	2	2	1.17E+05	1	1	1	41924
614	875	Q96HY6	26.04	5	5	2.34E+04	1	1	1	35611
531	835	Q8TCY9	26.01	1	1	0	1	1	1	104987
530	824	Q8N163	25.98	2	2	2.33E+04	1	1	1	102902
212	572	Q92901	25.87	2	2	2.36E+05	1	1	1	46296
494	855	Q71UI9	25.1	7	7	2.60E+06	1	1	1	13509
494	853	P0C0S5	25.1	7	7	2.60E+06	1	1	1	13553
617	890	P22735	25	2	2	2.27E+04	1	1	1	89787
618	897	Q13177	24.59	5	5	0	1	1	1	58043
620	899	P31949	24.31	9	9	1.07E+05	1	1	1	11740
621	902	P49448	24.12	1	1	2.35E+04	1	1	1	61434
621	901	P00367	24.12	1	1	2.35E+04	1	1	1	61398
623	905	P02008	24.04	5	5	4.14E+05	1	1	1	15637
464	529	Q02413	24.03	2	2	1.31E+05	1	1	1	113748
626	911	Q96HS1	23.91	2	2	2.61E+05	1	1	1	32004
238	1853	Q8TF72	23.91	0	0	1.14E+06	1	1	1	216856
258	13053	O43583	23.9	4	4	0	1	1	1	22092
624	908	Q8WVM8	23.77	2	2	7.14E+04	1	1	1	72380
625	910	Q9Y5L4	23.56	15	15	2.85E+04	1	1	1	10500
239	603	Q96A08	23.54	6	6	5.33E+05	1	1	1	14167
261	8497	Q14676	23.49	0	0	3.14E+04	1	1	1	226664
183	2827	P24043	23.4	0	0	2.26E+05	1	1	1	343906

183	13060	Q9H2M9	23.4	1	1	2.26E+05	1	1	1	155984
492	703	O75874	23.29	3	3	1.03E+05	1	1	1	46659
627	916	P54727	23.25	4	4	3.46E+05	1	1	1	43171
217	192	P0DPH7	22.77	2	2	7.55E+04	1	1	1	49960
217	271	P68366	22.77	2	2	7.55E+04	1	1	1	49924
217	98	Q9BQE3	22.77	2	2	7.55E+04	1	1	1	49895
217	191	P0DPH8	22.77	2	2	7.55E+04	1	1	1	49960
216	794	P02452	22.76	1	1	1.54E+05	1	1	1	138942
423	912	O43852	22.7	8	8	1.56E+04	1	1	2	37107
192	1175	P54132	22.69	1	1	1.10E+05	1	1	1	159000
538	915	Q9NP90	22.4	5	5	2.47E+05	1	1	1	22719
538	945	P59190	22.4	5	5	2.47E+05	1	1	1	24391
290	4718	Q14721	22.39	1	1	0	1	1	1	95878
268	13062	Q9P0V9	22.35	2	2	5.90E+04	1	1	1	52593
268	10357	Q9UH03	22.35	3	3	5.90E+04	1	1	1	40704
242	232	Q9BZZ5	22.32	2	2	6.39E+04	1	1	1	59005
539	919	Q14157	22.24	2	2	5.84E+04	1	1	1	114534
540	921	P09471	22.17	3	3	1.57E+05	1	1	1	40051
540	965	Q14344	22.17	3	3	1.57E+05	1	1	1	44050
540	967	Q03113	22.17	3	3	1.57E+05	1	1	1	44279
270	1107	Q6NXT2	22.11	5	5	3.07E+05	1	1	1	15214
533	868	Q8WUM4	21.99	1	1	0	1	1	1	96023
634	974	Q9BTW9	21.94	1	1	3.95E+05	1	1	1	132600
635	924	P54253	21.89	1	1	2.26E+05	1	1	1	86923
272	6287	Q9NRE2	21.62	1	1	0	1	1	1	115005
462	871	P15924	21.56	0	0	1.11E+05	1	1	1	331774
636	979	Q86UE4	21.5	2	2	2.12E+05	1	1	1	63837
638	982	O75340	21.48	5	5	3.76E+04	1	1	1	21868

294	13069	Q969Q0	21.34	8	8	9.49E+04	1	1	1	12469
245	4053	P22087	21.31	3	3	4.26E+04	1	1	1	33784
245	13068	A6NHQ2	21.31	3	3	4.26E+04	1	1	1	34803
641	987	O15344	21.18	3	3	2.19E+05	1	1	1	75251
541	928	Q9H9S3	21.09	2	2	1.24E+05	1	1	1	52248
642	988	P08572	21.06	1	1	1.03E+05	1	1	1	167553
424	930	Q08554	21.04	2	2	3.66E+04	1	1	1	99987
275	5019	O60610	21.03	2	2	6.21E+04	1	1	1	141347
181	1615	A7E2Y1	20.95	0	0	1.05E+05	1	1	1	225843
247	13073	Q9ULK4	20.86	1	1	1.74E+05	1	1	1	156473
647	995	Q10589	20.81	4	4	0	1	1	1	19769
648	996	Q13243	20.78	3	3	2.52E+05	1	1	1	31264
648	998	Q13247	20.78	3	3	2.52E+05	1	1	1	39587
648	999	Q08170	20.78	2	2	2.52E+05	1	1	1	56678
649	1009	O00442	20.6	2	2	1.50E+06	1	1	1	39337
496	880	Q9BYK8	20.46	0	0	8.88E+04	1	1	1	294649
653	1013	Q8NFT6	20.14	2	2	2.15E+06	1	1	1	67243
655	1016	Q9NZM1	20.05	1	1	7.31E+04	1	1	1	234706
656	1018	P30041	20.05	4	4	6.14E+04	1	1	1	25035
546	971	P39748	20	2	2	1.99E+05	1	1	1	42593

Table S6. Significant different expression of mRNA after HGDILnc1 knockdown.

SYMBOL	logFC	logCPM	PValue	FDR
HAL	-3.2712	6.520292	3.50E-119	8.55E-115
IGFBP1	-2.76111	11.07292	7.88E-107	9.63E-103
CA9	-3.43572	4.970436	3.32E-84	2.70E-80
STC2	-2.30575	6.644919	4.63E-72	2.83E-68
DDIT4	-1.84768	8.462628	2.03E-57	9.90E-54
ALDOC	-2.39734	5.372477	2.94E-53	1.20E-49
HDAC11	-1.85321	6.680445	7.26E-48	2.53E-44
SERPINE1	-2.75489	4.622899	7.32E-46	2.24E-42
PPP1R15A	1.75098	6.659637	7.68E-45	2.08E-41
H19	-2.28555	5.711457	5.61E-41	1.37E-37
PSAT1	-1.59383	8.042648	7.14E-41	1.59E-37
INHBE	-2.73272	4.363555	7.64E-40	1.55E-36
GDF15	-1.62362	9.552687	4.76E-39	8.95E-36
PHGDH	-1.92769	6.264504	4.27E-38	7.45E-35
IGFBP3	-1.57502	8.501782	2.15E-37	3.50E-34
NDRG1	-1.83392	5.326546	4.54E-37	6.93E-34
FABP1	-1.58229	6.733578	4.87E-37	7.00E-34
SDS	-2.06154	5.056807	9.01E-37	1.22E-33
TRIB3	-1.81811	5.662201	3.32E-36	4.27E-33
ITGB4	-1.59086	6.62366	2.92E-35	3.57E-32
AMDHD1	-1.55811	6.543797	4.13E-35	4.81E-32
MROH3P	9.855106	-0.01151	1.00E-34	1.11E-31
HSPA8	1.474886	10.83443	3.41E-34	3.62E-31
ASNS	-1.69385	7.841802	4.04E-34	4.12E-31
PCK2	-1.70765	7.093445	2.23E-33	2.18E-30
S100A14	-1.64528	5.813194	5.04E-32	4.74E-29
FAM71A	9.788184	-0.07482	9.05E-31	8.19E-28
ADAM1A	3.793371	1.625977	2.49E-30	2.17E-27
CRH	-1.33027	9.451363	1.15E-28	9.69E-26
OSGIN1	1.585665	5.303269	1.36E-27	1.11E-24
ANKRD37	-2.97653	2.992517	2.38E-27	1.88E-24
MTHFD2	-1.254	7.955938	3.74E-27	2.86E-24
FGF18	6.562095	-0.04875	7.34E-27	5.44E-24
F2	-1.30305	6.93037	1.17E-26	8.38E-24
BHLHE41	6.455022	-0.16117	1.55E-26	1.09E-23
IDH2	-1.25985	7.643307	1.88E-26	1.28E-23
TMEM230	1.386294	5.804345	2.02E-26	1.33E-23
CYP24A1	-1.24476	9.124385	2.63E-25	1.69E-22
TCIM	-1.37559	8.470645	6.61E-25	4.14E-22
NBEAP3	-9.9826	0.093635	9.02E-25	5.51E-22
LINC00313	-2.71661	2.586432	1.96E-24	1.17E-21
MYO1D	-1.18486	7.702432	2.56E-24	1.49E-21
SERPING1	-1.28244	6.624349	3.10E-24	1.76E-21
PCSK9	-1.18566	7.941833	3.35E-24	1.86E-21
RNA5-8SN3	2.943119	2.912503	5.18E-24	2.78E-21
RNA5-8SN2	2.946403	2.188953	5.24E-24	2.78E-21

SLC16A3	-1.40582	5.469055	5.88E-24	3.05E-21
GPX8	-1.88045	4.548887	1.21E-23	6.15E-21
HILPDA	-1.3653	6.669527	1.55E-23	7.71E-21
KLF10	1.576918	5.254523	5.60E-23	2.74E-20
ACSS2	-1.22063	6.684286	7.36E-23	3.52E-20
ADAMTSL4	-1.67063	4.464304	1.59E-22	7.48E-20
NUPR1	-1.28659	6.775076	1.94E-22	8.94E-20
H1-0	-1.17183	7.203909	3.10E-22	1.40E-19
CCND2	8.89898	-0.9201	5.00E-22	2.22E-19
LRP1	-1.24186	7.266623	1.06E-21	4.64E-19
CBS	-1.2816	7.108638	1.58E-21	6.76E-19
CXCR5	9.663357	-0.19109	3.02E-21	1.27E-18
AKAP12	-1.27832	8.823417	3.25E-21	1.34E-18
A2M	-1.07888	12.60893	3.49E-21	1.42E-18
PRECSIT	5.842113	-0.4436	5.51E-21	2.21E-18
PRKCA	-1.17256	7.287423	1.03E-20	4.06E-18
TNS1	-2.09616	3.271925	1.17E-20	4.55E-18
SPAG4	-2.10042	3.514013	1.72E-20	6.55E-18
SPON1	8.685053	-1.11722	2.38E-20	8.94E-18
PYGM	5.34417	0.62653	3.83E-20	1.42E-17
PYGL	-1.22773	5.662246	4.17E-20	1.52E-17
C1RL	-1.31492	5.564276	6.25E-20	2.25E-17
SLC1A4	-1.36264	5.269246	6.93E-20	2.45E-17
CFAP251	3.769192	1.488079	8.91E-20	3.11E-17
GARS1	-1.02706	8.626545	1.88E-19	6.46E-17
AHSG	-1.75947	4.30311	2.14E-19	7.24E-17
LHPP	-1.43035	4.889894	2.27E-19	7.61E-17
SNORD3B-1	3.683901	0.530159	2.99E-19	9.86E-17
SYNE1	1.657769	4.30709	3.06E-19	9.96E-17
CP	-1.1858	9.092945	3.11E-19	9.99E-17
SCARA5	-1.36976	4.899844	4.06E-19	1.29E-16
GAA	-1.45784	4.958727	5.00E-19	1.56E-16
SEMA4B	-1.25658	5.493917	5.92E-19	1.83E-16
UCKL1-AS1	4.154108	0.528896	6.22E-19	1.89E-16
IER3	-1.09086	7.781036	6.28E-19	1.89E-16
ANGPTL8	-2.68843	2.47409	1.60E-18	4.77E-16
APOL6	-1.68916	4.038175	2.13E-18	6.28E-16
KIF21B	-2.59333	4.111907	2.31E-18	6.73E-16
ALDH1L2	-2.44434	2.874696	4.23E-18	1.22E-15
ZNF610	8.571417	-1.21558	5.56E-18	1.58E-15
UTP14A	1.14786	5.940536	6.96E-18	1.95E-15
HPN	-1.67113	4.032525	7.89E-18	2.19E-15
PMEPA1	5.586647	-0.08704	8.72E-18	2.39E-15
FAM13B	1.656109	4.569071	1.30E-17	3.52E-15
AC012236	-3.29032	0.878027	1.61E-17	4.32E-15
SLC45A3	-1.48229	4.562058	1.72E-17	4.55E-15
BNIP3	-1.0497	6.806153	1.88E-17	4.90E-15
BHLHE40	-1.00426	7.255583	1.88E-17	4.90E-15

GLRX	-1.05446	6.200444	2.71E-17	6.98E-15
PAQR8	-1.03259	6.127	3.32E-17	8.46E-15
KDELR3	-1.14277	5.567937	3.44E-17	8.60E-15
SNHG12	1.788301	3.55384	3.45E-17	8.60E-15
MST1P2	-1.92241	3.70738	4.37E-17	1.08E-14
PECAM1	-1.28504	5.188587	4.41E-17	1.08E-14
CARS1	-1.04206	6.601521	5.91E-17	1.43E-14
TP53INP2	-1.02208	6.626482	6.10E-17	1.46E-14
SLC2A9	-2.03722	2.984047	6.53E-17	1.55E-14
PEAK3	8.667292	-1.13988	9.50E-17	2.23E-14
S100A9	-1.10445	7.664801	1.05E-16	2.44E-14
CKB	-1.2455	5.289761	1.24E-16	2.87E-14
ARG2	-1.28956	5.11588	1.45E-16	3.30E-14
ENO2	-2.18214	3.15491	1.93E-16	4.37E-14
KCNK6	-1.1567	5.28883	1.97E-16	4.41E-14
SERINC2	-1.38953	4.848099	2.47E-16	5.50E-14
EFNB2	4.358572	0.249623	4.10E-16	9.02E-14
CCNG2	-1.71756	3.709937	4.31E-16	9.35E-14
BNIP3L	-1.28491	5.136905	4.32E-16	9.35E-14
JUN	1.127604	5.334801	5.14E-16	1.10E-13
LINC01671	-3.16263	1.138795	6.23E-16	1.32E-13
VLDLR	-2.16294	2.124964	6.75E-16	1.42E-13
GPR157	-1.03362	6.375892	6.85E-16	1.43E-13
OTOAP1	8.184468	-1.57251	7.26E-16	1.49E-13
SLC7A1	-1.03062	6.783539	7.93E-16	1.61E-13
LIF	-1.56047	3.944987	8.60E-16	1.72E-13
PLCB1	-1.31702	5.24037	9.64E-16	1.91E-13
AC004893	3.487971	0.134722	1.13E-15	2.21E-13
GCNA	3.498614	1.170664	1.49E-15	2.88E-13
CDKL2	8.255583	-1.51208	1.68E-15	3.21E-13
CFB	-1.41503	4.323604	2.06E-15	3.88E-13
SCUBE2	4.878798	-0.73541	2.25E-15	4.20E-13
DHTKD1	-1.00616	5.928806	3.22E-15	5.88E-13
HMOX1	1.039817	8.071874	3.69E-15	6.68E-13
EID3	1.841771	3.133473	4.80E-15	8.63E-13
ANKRD1	2.372909	1.75702	6.34E-15	1.11E-12
RNA5-8SN1	3.07897	1.105771	6.65E-15	1.16E-12
CILP2	3.124841	0.173583	6.68E-15	1.16E-12
NR4A1	-1.00286	6.864663	7.25E-15	1.24E-12
SMARCE1P6	8.42314	-1.35607	7.85E-15	1.31E-12
CEACAM1	-1.35989	4.889943	8.38E-15	1.39E-12
BMERB1	-1.132	5.324395	1.09E-14	1.77E-12
NNMT	-1.33134	4.697966	2.22E-14	3.57E-12
FADS2	-1.2919	5.249806	3.30E-14	5.21E-12
NECAB2	3.263542	-0.14502	3.43E-14	5.35E-12
FXYD2	-1.06025	7.883985	3.44E-14	5.35E-12
WIPF3	-1.63854	3.504223	4.27E-14	6.60E-12
ATP2B2	-3.061	0.287682	5.29E-14	8.02E-12

MINDY2	1.819568	3.320135	5.80E-14	8.68E-12
MST1L	-1.92325	2.967088	6.29E-14	9.37E-12
NPM1P46	7.971933	-1.75891	6.39E-14	9.46E-12
CHRNA10	2.396076	0.820291	9.23E-14	1.34E-11
WIPI1	-1.06864	6.744356	1.22E-13	1.75E-11
AC091173	4.652842	-0.44111	1.24E-13	1.78E-11
QRICH2	2.238021	2.17006	1.32E-13	1.88E-11
GAL3ST1	-2.20216	1.458189	1.71E-13	2.41E-11
UBE2L6	-1.27853	4.383547	1.81E-13	2.53E-11
MUC2	-2.32808	2.848725	2.15E-13	2.95E-11
CDC25A	1.022347	5.500157	2.31E-13	3.14E-11
FERMT3	3.459011	1.136155	2.86E-13	3.87E-11
ABCC3	-1.27183	4.430085	3.27E-13	4.36E-11
THBS1	2.289148	1.715248	3.34E-13	4.44E-11
MYO7B	-2.90123	0.74807	3.64E-13	4.80E-11
UBD	1.699143	3.924585	4.02E-13	5.28E-11
MST1	-1.43485	3.896292	4.24E-13	5.54E-11
SMAD7	1.377202	3.970812	4.60E-13	5.94E-11
PDK1	-1.20563	5.39254	4.81E-13	6.19E-11
VAT1L	1.465313	3.818052	5.02E-13	6.42E-11
ABAT	-1.70968	3.068896	5.23E-13	6.66E-11
LINC00163	7.961453	-1.76263	5.62E-13	7.11E-11
CTH	-1.17988	4.853964	7.43E-13	9.31E-11
LRRN4CL	8.012523	-1.72262	8.65E-13	1.07E-10
NBR2	-1.82245	2.726391	1.10E-12	1.34E-10
PLK2	4.758364	-0.559	1.23E-12	1.50E-10
ABCG8	7.80494	-1.90373	1.26E-12	1.52E-10
P4HA2	-1.04217	5.165893	1.27E-12	1.53E-10
AL590064	-1.04091	5.227641	1.36E-12	1.63E-10
FLRT2	1.003021	5.270856	1.42E-12	1.69E-10
CERCAM	-1.00778	5.290172	1.63E-12	1.93E-10
ABCA2	-1.02345	5.204746	1.86E-12	2.17E-10
HLA-DMB	-1.00593	6.246642	1.93E-12	2.23E-10
ACHE	2.152928	1.400034	2.39E-12	2.75E-10
FXYD3	-1.22556	4.417504	2.62E-12	2.98E-10
AK7	-1.37886	4.243212	2.90E-12	3.27E-10
PPFIA4	-2.82188	0.780629	3.67E-12	4.10E-10
XPNPEP2	-1.56879	3.154901	3.85E-12	4.28E-10
COL7A1	1.565554	3.50101	4.31E-12	4.76E-10
VASH2	-1.91013	1.964521	4.97E-12	5.47E-10
KCTD12	4.008932	-1.11543	5.02E-12	5.50E-10
CARMIL2	1.925855	1.690692	5.47E-12	5.94E-10
FER1L4	-1.41756	3.805714	6.02E-12	6.51E-10
LRRC37A3	2.347395	0.578263	6.20E-12	6.67E-10
DMTN	-1.66799	3.336422	6.23E-12	6.68E-10
POP1	1.421846	4.299998	7.53E-12	8.00E-10
PKLR	-1.127	5.114027	8.16E-12	8.59E-10
PDE6G	7.769634	-1.93475	8.53E-12	8.90E-10

ZNF256	1.704232	2.604523	9.38E-12	9.71E-10
UPK1A	-1.11801	4.621945	1.10E-11	1.12E-09
ACTBP14	8.05629	-1.693	1.24E-11	1.25E-09
FRG2	7.674985	-2.02309	1.29E-11	1.29E-09
BCAT1	-1.15862	6.551614	1.34E-11	1.33E-09
KCNB1	3.598443	-0.34786	1.47E-11	1.45E-09
SCGN	8.138662	-1.59849	1.49E-11	1.46E-09
AMHR2	7.876459	-1.84151	1.82E-11	1.78E-09
PLOD2	-1.34642	3.947097	2.62E-11	2.53E-09
SULF2	4.541547	-1.41123	2.68E-11	2.58E-09
PABPC1L	-1.21053	5.150529	3.28E-11	3.12E-09
CNTNAP1	-1.40975	3.432661	3.59E-11	3.39E-09
F2RL2	2.176362	0.980367	3.65E-11	3.44E-09
PTGS1	-2.87233	0.113944	5.25E-11	4.93E-09
ATF3	2.289178	4.411857	5.52E-11	5.17E-09
ARL14	1.744391	1.92286	5.80E-11	5.37E-09
HOXA2	2.46354	0.832256	6.21E-11	5.72E-09
IRF4	7.782359	-1.92785	6.30E-11	5.78E-09
AC099489	4.024466	-1.30973	6.53E-11	5.98E-09
NPIPB5	1.719541	5.195861	7.77E-11	7.08E-09
RPPH1	3.299078	1.039592	1.01E-10	9.02E-09
AQP4	-2.40768	0.207096	1.08E-10	9.56E-09
SMG1P1	1.152706	4.799	1.11E-10	9.82E-09
GATA2	1.145154	4.229953	1.14E-10	9.95E-09
STARD8	-1.42886	3.212298	1.15E-10	9.98E-09
PCK1	-1.53374	3.929693	1.15E-10	9.98E-09
DEPP1	-1.70995	2.650504	1.18E-10	1.02E-08
SPRR2D	7.486671	-2.17947	1.24E-10	1.07E-08
ABCA7	-1.0512	4.877715	1.31E-10	1.11E-08
LGI4	-2.71255	0.976588	1.34E-10	1.13E-08
MELTF	-1.06651	4.635268	1.36E-10	1.15E-08
TTLL6	-1.26894	3.944528	1.51E-10	1.26E-08
ASIC1	-1.44542	3.148919	1.63E-10	1.36E-08
GPT2	-1.14571	5.655779	1.68E-10	1.39E-08
CCDC154	7.879852	-1.84633	1.91E-10	1.58E-08
SNX19	2.40944	0.779402	2.05E-10	1.68E-08
AC113194	7.539664	-2.13698	2.05E-10	1.68E-08
ACBD4	-1.07912	4.653311	2.15E-10	1.75E-08
UGT2B10	1.648498	2.016325	2.27E-10	1.84E-08
AC006111	2.831597	-0.41267	2.29E-10	1.85E-08
ZNF671	1.457573	2.814655	2.44E-10	1.96E-08
SLC22A1	4.964211	-1.53814	2.54E-10	2.03E-08
SMIM14	-1.34578	3.7464	2.61E-10	2.08E-08
SLC2A10	-7.46223	-2.25898	2.82E-10	2.23E-08
SLC16A5	-1.20742	4.452266	2.99E-10	2.36E-08
VGF	2.070708	1.521956	3.02E-10	2.37E-08
FOXD4L6	7.403664	-2.25302	3.06E-10	2.40E-08
TMEM214	-1.0013	7.026562	3.20E-10	2.50E-08

PDE9A	-1.28106	3.792475	3.41E-10	2.65E-08
LBP	-1.7553	2.021184	3.64E-10	2.80E-08
ELMO3	1.727149	1.725236	3.89E-10	2.96E-08
RNASE4	-1.00714	4.960081	4.07E-10	3.09E-08
DAO	-1.85407	1.755644	4.63E-10	3.49E-08
PAK6	7.417859	-2.24054	4.91E-10	3.69E-08
SDHAF4	-1.67629	2.112341	5.10E-10	3.81E-08
C5AR2	-1.64975	2.308992	5.23E-10	3.90E-08
DHRS2	1.796302	2.06871	6.42E-10	4.74E-08
YIPF2	-1.00882	4.628989	6.47E-10	4.75E-08
ZNF416	1.356051	3.311187	6.86E-10	5.00E-08
PDZD2	3.319796	-0.06539	7.75E-10	5.62E-08
H6PD	-1.96309	5.825877	8.15E-10	5.89E-08
SLC12A3	-1.19121	3.865538	8.42E-10	6.05E-08
RNF157	-1.36312	3.754925	1.00E-09	7.04E-08
CLDN6	3.43849	-0.19548	1.02E-09	7.15E-08
ATP2A3	-1.35412	3.397781	1.03E-09	7.20E-08
NACAD	4.919999	-1.54908	1.09E-09	7.55E-08
CCDC15-DT	4.284081	-1.01159	1.20E-09	8.34E-08
SLC6A9	-1.42296	2.914497	1.25E-09	8.63E-08
FXYD5	-1.06939	4.660204	1.26E-09	8.64E-08
ARHGEF37	-2.06995	0.672319	1.38E-09	9.39E-08
CRYL1	-1.30602	3.413332	1.39E-09	9.41E-08
GDA	-1.34731	4.589883	1.40E-09	9.45E-08
RAB4B	-1.13613	4.346282	1.50E-09	1.01E-07
CD3EAP	1.779604	3.830035	1.56E-09	1.05E-07
RASA4	-1.31436	3.297617	1.66E-09	1.11E-07
EHD2	-1.22437	3.831645	1.72E-09	1.15E-07
LRRC43	7.424881	-2.24202	1.74E-09	1.15E-07
CSF2RA	-1.1099	4.08599	1.75E-09	1.15E-07
SUMO4	7.472012	-2.19673	1.80E-09	1.18E-07
AC010378	-1.5165	2.767249	1.81E-09	1.18E-07
LINC02212	-1.73187	1.937217	1.81E-09	1.18E-07
NOC2LP2	1.57483	2.541607	1.90E-09	1.24E-07
C2	-1.67027	2.076443	1.96E-09	1.27E-07
BNIPL	2.6744	-0.6203	2.12E-09	1.37E-07
MIP	7.501338	-2.16697	2.16E-09	1.39E-07
ERFE	-1.95696	1.009234	2.27E-09	1.46E-07
COL12A1	5.117856	-1.38987	2.33E-09	1.49E-07
SLC38A3	-2.48369	0.875912	2.47E-09	1.57E-07
DCAF4L1	2.143804	0.150817	2.51E-09	1.59E-07
UGT1A1	1.091113	4.85047	2.56E-09	1.62E-07
CIDEC	-1.34999	2.970859	3.17E-09	1.97E-07
CORO2A	-1.0757	4.066211	3.31E-09	2.06E-07
AC027373	3.753871	-1.21742	3.56E-09	2.20E-07
AL589993	7.340828	-2.29571	3.63E-09	2.24E-07
ESR1	7.305102	-2.33658	3.93E-09	2.40E-07
TNS2	-1.08449	4.103628	4.02E-09	2.45E-07

C5AR1	-2.87753	4.020007	4.12E-09	2.50E-07
GNG4	-1.04049	4.483377	4.59E-09	2.77E-07
KRT18P4	2.533711	-0.04213	4.82E-09	2.90E-07
LINC02236	7.253071	-2.37371	5.22E-09	3.12E-07
PFKFB4	-1.11053	4.034194	5.38E-09	3.20E-07
SHISA9	7.637062	-2.04399	5.71E-09	3.38E-07
AL359844	7.273438	-2.35785	5.77E-09	3.40E-07
FBXW8	-1.05311	4.335269	5.86E-09	3.45E-07
SGK1	1.826675	1.493228	6.45E-09	3.76E-07
ARC	7.440759	-2.2238	6.60E-09	3.83E-07
LINC02593	1.540925	1.892048	6.61E-09	3.83E-07
AHNAK2	-1.27206	3.747208	6.84E-09	3.95E-07
FES	1.77554	1.648094	7.31E-09	4.19E-07
SCAT8	-1.1601	3.81151	7.78E-09	4.44E-07
FMNL1	-1.05613	4.085891	7.83E-09	4.46E-07
ACE	-1.4597	2.872816	8.67E-09	4.88E-07
PPIEL	2.929122	-0.2033	9.18E-09	5.14E-07
GPR176-DT	2.529637	0.370802	9.55E-09	5.34E-07
AC091230	7.496559	-2.1639	9.89E-09	5.51E-07
FAM131C	-3.8417	-0.29782	9.90E-09	5.51E-07
DLGAP1	-1.201	3.663472	1.04E-08	5.78E-07
AC106886	3.000694	1.888646	1.18E-08	6.54E-07
LINC02320	-4.61072	-1.12415	1.26E-08	6.92E-07
ADSS1	-1.58559	1.745932	1.28E-08	7.00E-07
FAM171A1	7.202312	-2.4106	1.29E-08	7.01E-07
MUC5B	-2.38023	-0.38966	1.30E-08	7.09E-07
RNF14P3	3.885297	-1.20996	1.38E-08	7.51E-07
LINC02404	-1.29662	2.8579	1.41E-08	7.62E-07
RNVU1-7	4.041654	-1.48883	1.41E-08	7.62E-07
TRMT112P6	8.901076	-0.92802	1.48E-08	7.95E-07
TYRO3P	3.934643	-1.16265	1.49E-08	7.96E-07
EPHX3	2.036501	0.770187	1.50E-08	8.02E-07
ACTG1P3	3.5721	-1.49379	1.53E-08	8.14E-07
NKILA	7.414372	-2.24782	1.64E-08	8.61E-07
CCT6P3	1.305159	2.921166	1.64E-08	8.61E-07
KANK3	-2.11767	0.560799	1.66E-08	8.70E-07
PIGZ	-1.98299	1.523899	1.77E-08	9.22E-07
ZNF710-AS1	-1.47148	2.254441	1.77E-08	9.23E-07
CHAC1	-1.4658	6.053819	1.98E-08	1.03E-06
ADM2	-1.61763	3.768887	1.99E-08	1.03E-06
AC012447	2.484987	0.177806	2.02E-08	1.04E-06
AGAP2	-1.65448	1.547503	2.10E-08	1.08E-06
KRT81	-1.69918	2.060871	2.11E-08	1.08E-06
DEPTOR	-1.22501	3.252467	2.22E-08	1.13E-06
CGREF1	-1.33759	2.929836	2.30E-08	1.15E-06
C6orf141	-1.73155	1.095649	2.38E-08	1.19E-06
CEL	3.483682	-1.08126	2.48E-08	1.24E-06
EVL	-1.1444	4.016602	2.68E-08	1.33E-06

LDHD	-1.95747	0.567004	2.73E-08	1.35E-06
KRT8P39	3.440474	-0.90039	2.78E-08	1.37E-06
VCAM1	1.254983	3.435427	2.91E-08	1.43E-06
NR0B1	3.556527	0.201356	2.92E-08	1.43E-06
LINC00957	-1.65635	1.316452	2.94E-08	1.44E-06
TRPV2	-2.07466	0.091127	3.01E-08	1.47E-06
MYLK2	2.569222	-0.4445	3.08E-08	1.50E-06
RUFY1-AS1	4.567269	-1.86062	3.22E-08	1.56E-06
TCEA1	-1.0321	6.257413	3.25E-08	1.57E-06
MEIS3	-1.24874	3.661798	3.35E-08	1.61E-06
5_8S_rRNA	2.94516	-1.32938	3.36E-08	1.61E-06
KRT7-AS	3.870152	-1.33813	3.36E-08	1.61E-06
LOX	-2.00551	0.119833	3.52E-08	1.69E-06
AP001053	5.027092	-2.21504	3.65E-08	1.73E-06
C11orf49	-1.01283	4.602786	3.81E-08	1.80E-06
FBXL16	-1.72382	1.504773	4.18E-08	1.96E-06
MORC2-AS1	9.85705	-0.00359	4.37E-08	2.04E-06
ANKRD18DP	7.267901	-2.36633	4.37E-08	2.04E-06
TOMM6	4.390477	5.685201	4.69E-08	2.17E-06
EPPK1	2.067966	-0.05136	4.82E-08	2.23E-06
TRIP6	2.143778	-0.11955	5.34E-08	2.44E-06
FUT3	-2.2972	0.138878	5.43E-08	2.48E-06
ZNF667	2.715758	-0.50628	5.62E-08	2.56E-06
LINC02728	4.579862	-1.83978	5.67E-08	2.58E-06
PRKCG	-1.37332	2.554826	5.69E-08	2.58E-06
GPR35	-1.1606	3.512438	6.01E-08	2.71E-06
SNORD3A	1.736852	1.479927	6.22E-08	2.81E-06
CLDN11	4.041415	-0.97016	6.31E-08	2.83E-06
INSM2	4.63659	-1.78753	6.31E-08	2.83E-06
OPRL1	-2.7547	-0.01926	6.37E-08	2.86E-06
TLE2	-1.3667	2.796837	6.56E-08	2.93E-06
LTK	7.223788	-2.39447	6.62E-08	2.96E-06
LGR4	-1.32827	7.306512	6.95E-08	3.09E-06
GYS2	-4.41307	-0.01203	7.04E-08	3.11E-06
TEF	1.242958	2.825588	7.72E-08	3.37E-06
CCK	1.075998	3.835193	7.79E-08	3.39E-06
TAT	-1.29928	3.138036	7.80E-08	3.39E-06
COL6A2	-1.45737	2.116086	7.80E-08	3.39E-06
NCF2	6.986082	-2.58635	8.12E-08	3.51E-06
KRT80	2.019878	0.455621	8.30E-08	3.57E-06
HEY1	1.695411	0.894978	8.36E-08	3.59E-06
CLYBL	-1.15689	3.59865	8.56E-08	3.65E-06
PIP5KL1	-2.1109	0.313606	8.60E-08	3.67E-06
WNK4	-1.09094	3.600769	8.88E-08	3.77E-06
ULBP1	-1.33126	2.79744	8.89E-08	3.77E-06
LINC00552	4.612039	-0.97058	9.11E-08	3.86E-06
B4GALNT2	-1.033	4.212107	9.79E-08	4.12E-06
MORN4	-1.21655	2.984893	1.03E-07	4.31E-06

DRG1P1	3.105711	-0.56637	1.05E-07	4.38E-06
SHANK2	-1.39818	5.333429	1.06E-07	4.41E-06
NUP210L	5.075037	-2.18214	1.07E-07	4.47E-06
SULT1B1	-1.81063	0.531882	1.08E-07	4.51E-06
FRG2C	4.622192	-1.80841	1.16E-07	4.77E-06
NPIPB3	1.153838	3.4818	1.17E-07	4.81E-06
P3H3	-1.7222	1.105977	1.19E-07	4.86E-06
IMPDH1P5	3.078226	-1.41392	1.24E-07	5.07E-06
AC026691	7.572458	-2.08209	1.24E-07	5.07E-06
C11orf96	2.267116	-0.28971	1.35E-07	5.48E-06
FAM20A	-1.47419	2.357162	1.36E-07	5.48E-06
TIMP3	-1.39508	2.107323	1.42E-07	5.68E-06
ERVK9-11	1.276427	2.55431	1.49E-07	5.93E-06
TBC1D8B	-1.03701	5.016041	1.49E-07	5.94E-06
NPIPB11	1.642694	1.197602	1.51E-07	6.00E-06
ENO3	1.015686	3.799234	1.67E-07	6.60E-06
NGEF	-1.07067	3.858124	1.67E-07	6.60E-06
MYH3	1.640855	0.879837	1.71E-07	6.71E-06
AC010536	2.597249	-0.84506	1.75E-07	6.85E-06
CACNG6	-1.13977	4.116635	1.76E-07	6.89E-06
C2orf81	-1.74425	1.105546	2.07E-07	8.07E-06
FAM234A	-1.02233	4.883942	2.13E-07	8.29E-06
TMEM150B	-1.31504	2.114094	2.16E-07	8.39E-06
MIR1244-1	8.972974	2.302441	2.21E-07	8.53E-06
PPP1R14A	-1.17638	3.351357	2.40E-07	9.19E-06
KCNMB4	-1.31214	2.545224	2.42E-07	9.26E-06
SARM1	1.042971	6.191165	2.55E-07	9.69E-06
MIR181A1HG	6.874584	-2.67461	2.70E-07	1.02E-05
PHF24	4.477342	-1.9523	2.71E-07	1.02E-05
HLA-DOA	-2.16948	-0.15406	2.73E-07	1.03E-05
NDUFA9P1	7.042222	-2.5458	2.73E-07	1.03E-05
FXYD6	-1.29854	1.932095	2.78E-07	1.04E-05
COL6A1	-1.04879	3.72753	2.89E-07	1.08E-05
EYS	-1.2538	2.669473	3.03E-07	1.13E-05
SLC1A2	-1.53964	1.557652	3.09E-07	1.14E-05
SNORD3B-2	3.299359	-1.25515	3.10E-07	1.15E-05
JAK3	-1.70731	1.967339	3.11E-07	1.15E-05
KLF15	-1.22773	2.925574	3.42E-07	1.25E-05
Z93241	3.862669	-1.80038	3.50E-07	1.27E-05
MYRF-AS1	-6.948	-2.68607	3.50E-07	1.27E-05
MALAT1	-1.0212	6.636995	3.52E-07	1.27E-05
GPR75	1.704851	0.972052	3.53E-07	1.28E-05
NPHP3-ACAD11	-9.30322	-0.55489	3.61E-07	1.30E-05
HMGB1P5	1.130087	5.623426	3.71E-07	1.33E-05
GPR137B	-1.00705	3.848287	3.77E-07	1.35E-05
CHRNB2	1.947271	0.02645	3.99E-07	1.42E-05
SVEP1	6.944926	-2.62007	4.04E-07	1.43E-05
C3orf35	2.083285	0.018218	4.19E-07	1.48E-05

JDP2	-1.76473	3.837656	4.29E-07	1.51E-05
KAZN	-2.2485	1.401927	4.34E-07	1.52E-05
AASS	-1.24298	3.632402	4.53E-07	1.58E-05
CDH23	-1.10644	3.17496	4.63E-07	1.61E-05
PLB1	2.184227	-0.35166	4.81E-07	1.66E-05
VSTM1	6.79412	-2.74276	5.03E-07	1.73E-05
SH2D1B	-2.07139	0.7562	5.59E-07	1.89E-05
SLCO2B1	-1.01529	5.448382	5.64E-07	1.90E-05
TP53TG3E	-8.23368	-1.58352	5.66E-07	1.91E-05
KLF4	1.368035	3.953739	5.86E-07	1.97E-05
ICAM5	-1.29185	2.219067	5.93E-07	1.99E-05
AL442063	6.819151	-2.72378	6.17E-07	2.06E-05
KRT18P37	4.754662	-2.02349	6.23E-07	2.08E-05
AC006372	5.389181	-2.54095	6.51E-07	2.17E-05
ENTPD2	-1.92281	0.268748	6.60E-07	2.19E-05
FJX1	2.140469	-0.57206	6.83E-07	2.26E-05
SLC44A3-AS1	-1.2425	2.199946	6.96E-07	2.30E-05
HK2	-1.35428	1.585054	7.02E-07	2.31E-05
VSTM5	3.666364	-0.9204	7.22E-07	2.36E-05
AP001425	-2.21684	0.194086	7.37E-07	2.40E-05
SAMD11	1.342147	3.125682	7.41E-07	2.40E-05
LINC02803	6.970986	-2.60006	7.51E-07	2.43E-05
ART4	2.156108	-0.55749	7.55E-07	2.44E-05
RIMS3	-1.0885	3.462361	7.57E-07	2.45E-05
BRSK1	-1.23672	2.27126	7.75E-07	2.50E-05
LINC00334	6.991854	-2.59107	7.99E-07	2.57E-05
AP000757	-1.3449	1.461314	8.06E-07	2.59E-05
H4C5	2.511223	-0.47698	8.28E-07	2.64E-05
OPN3	-1.37387	2.610972	8.58E-07	2.72E-05
SBK1	-1.46874	1.378387	8.89E-07	2.81E-05
HSPE1-MOB4	-11.2767	1.362848	9.08E-07	2.86E-05
TRAPPC6A	-1.05514	3.384276	9.40E-07	2.94E-05
ACTA1	6.853013	-2.69163	9.41E-07	2.94E-05
DLX3	4.43282	-1.96781	9.54E-07	2.98E-05
CREB3L1	-1.70213	1.645596	9.63E-07	3.00E-05
ACVRL1	-1.65836	1.017393	9.98E-07	3.10E-05
RSPH6A	6.773228	-2.75141	1.02E-06	3.17E-05
CD300A	7.038006	-2.5475	1.04E-06	3.22E-05
BZW1P2	1.302004	1.836618	1.04E-06	3.23E-05
ST3GAL1	-1.06911	3.380667	1.05E-06	3.24E-05
FZD2	-1.04666	3.45452	1.05E-06	3.24E-05
CLDN7	-1.22851	2.843652	1.07E-06	3.30E-05
RAPGEF4	-1.17352	2.567489	1.10E-06	3.39E-05
SMG1P4	1.683641	1.208975	1.12E-06	3.44E-05
CCDC17	1.680974	0.69478	1.16E-06	3.55E-05
AC079601	6.759501	-2.76612	1.22E-06	3.71E-05
GRM2	6.745653	-2.78118	1.22E-06	3.71E-05
CUZD1	4.221356	0.560837	1.22E-06	3.72E-05

AC090192	1.889709	0.191608	1.23E-06	3.74E-05
LINC00858	-4.89906	-2.50025	1.27E-06	3.82E-05
DSCR10	-1.63533	0.386926	1.28E-06	3.86E-05
AL645728	2.557192	-0.78986	1.29E-06	3.89E-05
LY96	-3.05507	-0.47343	1.31E-06	3.94E-05
SH3BP2	-1.95797	2.010328	1.44E-06	4.29E-05
C3P1	-1.25353	2.564994	1.44E-06	4.29E-05
BICD1P1	7.042321	-2.5381	1.46E-06	4.34E-05
ASS1P5	4.215705	-2.13833	1.48E-06	4.39E-05
PLAT	-1.70876	0.073039	1.49E-06	4.41E-05
BAIAP3	-1.21416	3.204742	1.55E-06	4.57E-05
MAGED4B	-1.25039	2.384522	1.57E-06	4.60E-05
RPH3AL	-1.0338	3.965362	1.58E-06	4.64E-05
TUSC8	3.624391	-1.25485	1.59E-06	4.65E-05
ARMH1	-2.01732	-0.1674	1.61E-06	4.71E-05
SMG1P2	1.124824	3.176494	1.62E-06	4.71E-05
ZMYND15	-1.18446	2.847136	1.63E-06	4.73E-05
AL590763	4.62817	-2.30591	1.63E-06	4.73E-05
ITGA3	-1.03008	4.110153	1.64E-06	4.76E-05
LINC01669	-3.96906	0.911224	1.69E-06	4.88E-05
AL049836	-2.74913	-1.39044	1.69E-06	4.88E-05
RTEL1P1	6.770634	-2.77137	1.69E-06	4.88E-05
IL21R	-3.45562	-0.46416	1.69E-06	4.88E-05
AL031710	4.693713	-2.49094	1.75E-06	5.02E-05
ASPG	-2.93865	-1.30005	1.76E-06	5.04E-05
TMEM91	-1.80202	0.353248	1.77E-06	5.07E-05
BPIFB9P	6.807551	-2.73779	1.91E-06	5.44E-05
PAX8	-1.31852	1.521835	1.92E-06	5.47E-05
FAM117A	-1.02649	3.452121	2.00E-06	5.66E-05
ITGA10	-1.18293	2.662897	2.05E-06	5.77E-05
LIMD2	-1.13517	2.765103	2.06E-06	5.80E-05
ALDH6A1	-1.05706	3.151848	2.08E-06	5.86E-05
HNF4A-AS1	-6.64949	-2.92126	2.09E-06	5.86E-05
AL034417	2.300446	-0.90555	2.10E-06	5.89E-05
AL162741	2.608375	-1.32946	2.15E-06	6.04E-05
PARVB	-1.08753	3.24065	2.17E-06	6.07E-05
KRT8P15	6.714644	-2.81271	2.19E-06	6.12E-05
LINC01963	1.300703	2.036833	2.40E-06	6.61E-05
AC091806	-1.00394	3.712181	2.42E-06	6.66E-05
FAM13A	-1.04536	3.813517	2.42E-06	6.66E-05
AC119673	3.785035	-2.04853	2.44E-06	6.70E-05
CYS1	-1.93429	0.779152	2.45E-06	6.73E-05
RIMBP3B	-7.71626	-2.03892	2.55E-06	6.98E-05
FRG2EP	6.733176	-2.80583	2.62E-06	7.16E-05
KRT17	2.960304	-1.42982	2.66E-06	7.26E-05
SYT7	-1.24324	2.050117	2.75E-06	7.47E-05
LCT	4.365822	-2.02724	2.88E-06	7.82E-05
THSD4	1.259447	1.753733	2.89E-06	7.85E-05

ALOXE3	1.768246	1.037333	2.91E-06	7.87E-05
MAGED4	-1.4085	2.302153	2.92E-06	7.90E-05
FGF8	6.660647	-2.85424	2.95E-06	7.95E-05
AC073288	6.735905	-2.77537	2.97E-06	8.00E-05
KRT18P5	2.818094	-1.28612	2.98E-06	8.03E-05
SV2A	1.507558	1.086435	3.08E-06	8.27E-05
AC124276	6.662223	-2.85359	3.09E-06	8.29E-05
DQX1	-1.12547	2.745065	3.20E-06	8.55E-05
SLC6A3	6.592142	-2.89104	3.22E-06	8.58E-05
ALPK3	1.823896	0.172831	3.40E-06	9.03E-05
PKD1L2	-1.335	1.355387	3.40E-06	9.04E-05
LY6G6C	6.693678	-2.81105	3.66E-06	9.68E-05
H2AC12	2.242847	-0.93105	3.69E-06	9.75E-05
ERFL	-1.59539	0.066552	3.75E-06	9.89E-05
ABCA3	-1.04973	3.449392	3.78E-06	9.95E-05
CYP4F26P	1.010038	3.342917	3.85E-06	0.000101
ZNF229	1.29029	1.965786	3.88E-06	0.000102
SEMA6B	-1.05858	3.119834	3.89E-06	0.000102
SLC1A7	-4.28431	-2.03815	3.89E-06	0.000102
ROCK1P1	1.07304	3.171781	3.93E-06	0.000103
RBPMS2	-1.01235	3.499576	4.04E-06	0.000105
REM2	6.711811	-2.81381	4.20E-06	0.000109
TRANK1	-1.30749	1.894153	4.34E-06	0.000112
ZDHHC8	-1.07935	2.913017	4.60E-06	0.000119
PLCD1	-1.05893	3.283662	4.60E-06	0.000119
H4C2	4.838269	-2.34614	4.63E-06	0.000119
SAMD12	-1.32854	2.640338	4.87E-06	0.000125
AKNA	-1.12554	3.259931	4.88E-06	0.000125
LINC00239	-1.37496	1.556462	5.05E-06	0.000129
S100A2	1.575615	0.331654	5.14E-06	0.000131
TTC25	-1.55946	0.884141	5.14E-06	0.000131
SLC26A4-AS1	6.581032	-2.91662	5.26E-06	0.000134
SNAPC4	1.050112	3.382165	5.34E-06	0.000135
FLJ31104	1.94183	-0.6404	5.41E-06	0.000137
CSF3R	-2.72249	-1.03891	5.52E-06	0.000139
EIF4BP2	2.940381	-1.69738	5.65E-06	0.000142
AC004585	-6.57221	-2.98042	5.83E-06	0.000147
CPLX2	2.577966	0.721368	5.84E-06	0.000147
C3orf18	-1.0194	3.320885	5.94E-06	0.000149
ZSWIM4	-1.31414	1.606022	6.17E-06	0.000154
AC040173	6.664962	-2.83196	6.18E-06	0.000154
TMEM8B	-1.10518	2.563596	6.32E-06	0.000157
LIMD1-AS1	-2.67552	1.745948	6.66E-06	0.000165
PTENP1	2.252511	-0.01671	6.72E-06	0.000166
PRODH	-1.25492	1.771579	6.82E-06	0.000168
AHRR	2.686384	-1.64953	6.86E-06	0.000169
AC026954	6.773316	-0.49133	7.03E-06	0.000172
MARCHF3	-1.07302	2.751755	7.34E-06	0.000179

PRSS22	-2.35982	0.241935	7.37E-06	0.000179
HPSE2	7.079503	-2.54631	7.37E-06	0.000179
AC073130	3.152632	-2.15351	7.48E-06	0.000181
KCNN1	-1.27612	1.923654	7.64E-06	0.000185
ABCG4	-1.5602	1.028953	7.67E-06	0.000185
SPIB	4.127904	-2.21129	7.73E-06	0.000186
AC008074	2.377983	-1.46366	7.73E-06	0.000186
AC105942	-1.06999	2.826396	7.76E-06	0.000187
RRH	6.536128	-2.93348	7.78E-06	0.000187
ADRB2	4.138605	-2.20054	8.34E-06	0.000199
LDHBP1	6.400944	-3.04038	8.40E-06	0.0002
ALPK1	-1.06866	3.588826	8.54E-06	0.000202
LAT	1.503871	0.431813	8.80E-06	0.000207
HOXD9	2.695111	-1.06718	9.02E-06	0.000212
ZDHHC11B	1.097618	2.753365	9.06E-06	0.000212
GNG7	-1.58818	0.564066	9.36E-06	0.000218
LRRC3	-1.26891	1.788379	9.50E-06	0.000221
RBM23	2.173763	-0.12091	9.57E-06	0.000222
AC078983	6.646499	-2.83887	9.87E-06	0.000228
AC023157	1.431202	1.10848	9.89E-06	0.000228
PCDH9	1.673012	4.24918	1.02E-05	0.000235
CCDC136	-1.68373	0.212004	1.05E-05	0.00024
IGF2BP2-AS1	6.592866	-2.87986	1.07E-05	0.000243
OSCAR	-2.9986	-0.33583	1.09E-05	0.000246
CELF6	7.425476	-2.22001	1.20E-05	0.000269
FOXD4L3	6.691609	-2.83265	1.20E-05	0.000269
CAPN8	-1.08363	2.681795	1.20E-05	0.000269
SYT11	-1.17457	2.074578	1.20E-05	0.000269
TBC1D5	-1.03188	4.718472	1.20E-05	0.000269
GDPD2	6.598109	-2.89936	1.25E-05	0.000277
H1-4	1.987915	-0.87873	1.27E-05	0.00028
RND3	1.320725	1.564153	1.27E-05	0.000281
H4C11	1.377464	1.404556	1.28E-05	0.000282
TMEM106A	2.933489	-1.69643	1.28E-05	0.000282
ACBD7	-1.10253	2.350079	1.28E-05	0.000283
PART1	-1.38231	0.960523	1.32E-05	0.000291
PCAT6	-1.31453	1.598745	1.33E-05	0.000292
AC092868	-1.3288	0.874026	1.33E-05	0.000292
MAFB	3.44831	-1.14073	1.33E-05	0.000292
GFY	-1.21868	1.711684	1.34E-05	0.000292
PALM	-1.11269	3.424051	1.34E-05	0.000293
TRIM67	2.057404	-0.34287	1.34E-05	0.000293
KRT8P36	2.511736	-1.51853	1.36E-05	0.000296
SLFN14	6.382537	-3.04684	1.40E-05	0.000303
MAGEA2B	1.129645	4.475326	1.41E-05	0.000305
NRCAM	1.007815	3.060353	1.42E-05	0.000307
SLC29A4	-1.15839	3.712387	1.44E-05	0.00031
MAP3K14-AS1	-1.30243	2.011342	1.45E-05	0.000313

JCADP1	6.4006	-3.04074	1.45E-05	0.000313
EFHD1	-1.42413	0.448433	1.47E-05	0.000315
CCNJL	-1.39556	5.625615	1.48E-05	0.000317
TBR1	6.872973	-2.66572	1.53E-05	0.000327
GAS6	1.766845	0.183479	1.54E-05	0.000328
RNVU1-31	6.354665	-3.06891	1.54E-05	0.000329
AL358473	-3.253	-1.58412	1.54E-05	0.000329
VTRNA1-3	4.185203	-2.18034	1.57E-05	0.000333
KLC3	-1.18863	1.635381	1.60E-05	0.000337
CDKN1C	4.180542	-2.20157	1.62E-05	0.000341
PCDH10	4.457376	-2.30139	1.64E-05	0.000344
CRYM	-1.14746	2.018145	1.66E-05	0.000347
CERNA1	-1.54577	0.328863	1.67E-05	0.000348
SERINC4	3.425328	-1.74103	1.70E-05	0.000354
AC022210	-1.4389	0.995001	1.70E-05	0.000354
ZNF296	1.037867	2.525323	1.72E-05	0.000357
TAC1	6.619791	-2.85907	1.73E-05	0.00036
ADAM20P1	4.181006	-2.17913	1.73E-05	0.00036
ZNF556	6.497182	-2.95851	1.75E-05	0.000362
CHD5	2.142904	-0.85013	1.75E-05	0.000363
AC074212	-1.85025	0.66533	1.76E-05	0.000364
UCN2	3.324335	-1.20843	1.76E-05	0.000365
PAX2	6.552867	-2.91627	1.78E-05	0.000366
ST14	1.573898	0.710688	1.80E-05	0.000371
ARHGEF4	-1.22721	1.185977	1.81E-05	0.000373
NR1D1	1.08636	3.062028	1.83E-05	0.000375
RPL36AP21	6.597268	-2.88854	1.84E-05	0.000376
MIA-RAB4B	4.108343	0.642758	1.84E-05	0.000376
AC108690	-1.66623	0.65349	1.84E-05	0.000376
CDK5R1	1.282847	1.985785	1.86E-05	0.000379
GPR84	6.498166	-2.97008	1.86E-05	0.000379
AC109326	2.393468	-1.15405	1.88E-05	0.000383
EPOR	-1.16693	3.417725	1.89E-05	0.000384
MIRLET7BHG	2.342547	-0.91798	1.89E-05	0.000385
AL590714	1.088936	2.241819	1.94E-05	0.000391
FGR	-6.53338	-3.01383	1.94E-05	0.000391
CSTA	-1.46736	1.099058	1.94E-05	0.000391
ARRDC4	1.492467	0.610604	1.94E-05	0.000392
MIR22HG	1.08876	3.564582	1.96E-05	0.000394
PAQR4	1.598996	3.665472	1.96E-05	0.000394
C1GALT1C1	-2.18652	4.564698	2.00E-05	0.0004
OR1F1	-1.66815	-0.36087	2.05E-05	0.000409
AC090360	6.4321	-3.01691	2.10E-05	0.000417
PPM1M	-1.44971	3.329328	2.10E-05	0.000418
TMEM92	-1.57171	0.319757	2.11E-05	0.000418
HSD17B12	-1.04078	7.151617	2.14E-05	0.000423
SLC25A45	-1.07969	3.569836	2.15E-05	0.000426
RNU4ATAC	4.012822	-1.84569	2.19E-05	0.000433

CDK18	-1.5021	2.889526	2.22E-05	0.000438
CYP21A2	6.486953	-2.96239	2.27E-05	0.000446
SARDH	-1.05348	2.507942	2.27E-05	0.000446
TMEM121B	6.615497	-2.86043	2.35E-05	0.00046
SYNE3	-1.39065	0.981394	2.42E-05	0.000469
AL928921	-1.52276	1.080414	2.42E-05	0.000469
AC244033	-1.29204	0.678882	2.43E-05	0.000469
CKM	2.622252	-1.50344	2.43E-05	0.00047
NYAP1	-1.22983	1.424265	2.51E-05	0.000484
A4GALT	-1.0074	2.51292	2.55E-05	0.000491
RMRP	3.773387	0.578805	2.60E-05	0.0005
H3C12	2.24796	-1.13381	2.61E-05	0.000501
MMP23B	6.310409	-3.10998	2.61E-05	0.000501
AC004898	6.329044	-3.10378	2.66E-05	0.00051
SRPX	-1.14793	3.19264	2.70E-05	0.000516
TNRC18P1	2.974347	-1.04143	2.72E-05	0.00052
MCHR1	7.200828	-2.43223	2.72E-05	0.00052
TEPP	-1.85343	-1.10047	2.88E-05	0.000546
ADH1A	1.120969	1.668551	2.88E-05	0.000546
KRT8P41	6.288396	-3.11712	2.89E-05	0.000547
AC023154	6.455418	-2.99661	2.94E-05	0.000555
AC026740	2.143025	-1.14359	2.94E-05	0.000555
SNORD3D	2.839066	-1.79557	2.94E-05	0.000555
AC062037	1.746882	-0.31356	2.98E-05	0.00056
CAMKV	-1.45867	0.215574	3.03E-05	0.000567
SLCO4C1	-2.10103	-0.4981	3.04E-05	0.000569
SNORC	-1.03807	2.895084	3.05E-05	0.00057
RNVU1-27	2.729076	-1.78459	3.11E-05	0.000581
TMEM178A	-2.00562	0.114504	3.12E-05	0.000581
SERPINA5	-1.16701	2.057474	3.13E-05	0.000583
D2HGDH	-1.16501	2.15956	3.15E-05	0.000585
CALN1	-1.01472	2.645592	3.17E-05	0.000589
C1orf105	2.095134	-0.54094	3.21E-05	0.000596
ORAI2	-1.0949	3.611491	3.21E-05	0.000596
RPL17-C18orf32	-12.422	2.502356	3.22E-05	0.000597
EGR2	6.41278	-3.03661	3.27E-05	0.000606
AC069288	2.779129	2.24578	3.33E-05	0.000613
AJ239322	6.268501	-3.13748	3.35E-05	0.000616
DSC3	1.078065	2.787621	3.36E-05	0.000618
TNFRSF1B	-2.24363	-0.12263	3.44E-05	0.00063
RAPSN	6.770515	-2.76149	3.47E-05	0.000634
ADAM19	-1.60554	3.199299	3.47E-05	0.000635
H4C8	1.577434	0.471898	3.48E-05	0.000636
ZNF528	2.31475	-0.50738	3.53E-05	0.000644
AC124852	7.077399	-2.50741	3.65E-05	0.000663
DCAF13P3	6.349234	-3.07076	3.77E-05	0.000682
L3MBTL2-AS1	6.403171	-3.0268	3.78E-05	0.000683
ARL4D	-1.08045	2.732312	3.78E-05	0.000683

JAKMIP3	1.902328	-0.99711	3.80E-05	0.000687
NTN5	-2.22462	-1.31062	3.86E-05	0.000695
MIR193BHG	1.832156	0.04439	3.93E-05	0.000706
AC123595	4.378673	-2.73525	3.95E-05	0.000708
LINC02609	-1.50336	0.296933	3.97E-05	0.000711
SCAMP5	1.457738	2.683871	4.01E-05	0.000717
ERICH2	-1.73402	-0.35823	4.14E-05	0.000736
ADAMTS4	7.287054	-2.35086	4.19E-05	0.000745
NCAM2	-1.76167	0.284714	4.21E-05	0.000747
FLJ13224	3.266553	-1.62093	4.32E-05	0.000763
HDC	6.249818	-3.15731	4.38E-05	0.000773
FBXW4P1	1.867392	-0.86017	4.39E-05	0.000773
UBE2FP1	1.613545	-0.37987	4.52E-05	0.000793
PRR19	1.109742	1.532395	4.52E-05	0.000793
MYO7A	-1.45202	0.65843	4.53E-05	0.000794
SLC5A5	-1.16264	1.861518	4.72E-05	0.000825
ATP8B3	-1.05739	2.61152	4.83E-05	0.00084
PTENP1-AS	6.496952	-2.94699	4.84E-05	0.000841
SLC35G5	6.418716	-3.034	4.85E-05	0.000841
Z99774	4.675133	-2.93813	4.87E-05	0.000844
YBX2	-1.0308	3.083988	4.88E-05	0.000845
CHMP1B2P	-1.0312	2.291369	4.98E-05	0.000861
TNS4	-1.30453	1.470806	5.32E-05	0.000912
SMG1P3	1.385035	2.157567	5.35E-05	0.000915
NAAA	1.584933	0.077891	5.36E-05	0.000917
FXYD1	-1.77198	-0.10366	5.43E-05	0.000927
OSTCP1	6.236921	-3.1614	5.45E-05	0.000929
HSH2D	-1.19901	1.750531	5.53E-05	0.00094
BMP2	1.017497	2.367744	5.57E-05	0.000945
CFAP97D1	3.742607	-1.73226	5.70E-05	0.000963
RASSF2	-1.59606	0.262716	5.72E-05	0.000966
WNT9A	1.58585	0.05951	5.80E-05	0.000977
AL355385	-6.28847	-1.04869	6.11E-05	0.001023
CD226	6.946865	-2.60131	6.15E-05	0.001028
SCN5A	-6.39785	-3.10088	6.15E-05	0.001028
MST1R	-1.33523	0.426776	6.21E-05	0.001037
AL133517	-1.55731	0.143259	6.27E-05	0.001045
SRGAP2B	-1.07158	3.951407	6.29E-05	0.001046
PPP1R1C	-1.87737	0.206337	6.42E-05	0.001066
RPL4P6	1.896847	-0.23899	6.45E-05	0.00107
SELPLG	3.09615	-0.88747	6.52E-05	0.001078
FAM209A	3.339752	-2.21279	6.66E-05	0.0011
DAG1	2.094039	5.881093	6.70E-05	0.001106
ETS1	1.319496	1.332382	6.71E-05	0.001108
MAP3K14	1.133896	4.745102	6.72E-05	0.001108
RASAL1	-2.0125	-0.52733	6.75E-05	0.001111
IL15RA	1.946914	-0.71893	6.84E-05	0.001124
DOK6	-1.01095	3.057325	6.86E-05	0.001125

PKD1L1	3.096845	-0.83776	6.88E-05	0.001127
AC006128	-1.71326	0.424026	6.89E-05	0.001128
CYP2E1	2.054809	-0.88107	6.94E-05	0.001133
SMIM10L2B	-1.0419	1.694497	6.97E-05	0.001138
PIK3R5	-2.66489	-1.40855	6.99E-05	0.001139
SLC35G6	4.150117	-2.21224	7.05E-05	0.001147
APBB1	-1.009	2.436039	7.19E-05	0.001166
ABCG5	6.132452	-3.22358	7.22E-05	0.00117
RAB3C	-6.27986	-3.19946	7.24E-05	0.001171
AL031985	-1.13583	1.136508	7.32E-05	0.001182
VSIG1	1.076447	1.58742	7.35E-05	0.001186
EFEMP2	-1.2317	1.28981	7.47E-05	0.001202
C1GALT1P1	6.267353	-3.13744	7.50E-05	0.001204
TNK2-AS1	6.475735	-2.96541	7.56E-05	0.001213
FARP1-AS1	4.342003	-2.73387	7.59E-05	0.001216
HSD11B1-AS1	-6.99505	-2.64773	7.60E-05	0.001217
RNU1-1	3.248192	-2.12661	7.63E-05	0.001221
DSCAML1	-2.08092	-0.16025	7.86E-05	0.001251
DCLK2	1.038109	2.590012	7.89E-05	0.001251
AIF1	-7.03397	-2.60318	7.93E-05	0.001256
LINC02595	1.319647	0.159227	8.15E-05	0.001284
C1orf189	2.736234	-1.10812	8.29E-05	0.001305
AC116535	4.144615	-2.48964	8.30E-05	0.001305
LINC01943	3.952081	-2.35161	8.34E-05	0.00131
RPL36A-HNRNPH2	-10.6575	0.747853	8.41E-05	0.001318
AC009506	1.009554	2.156192	8.43E-05	0.001321
ITGAM	2.484585	-0.41464	8.45E-05	0.001324
CD300LB	6.047411	-3.29562	8.53E-05	0.001334
AC005082	6.079765	-3.26227	8.68E-05	0.001354
KCNJ14	1.483735	0.518651	8.69E-05	0.001356
ZNF254	-1.07127	2.580943	8.86E-05	0.001377
PTK7	-1.14572	1.733283	8.95E-05	0.001389
SH3GL1P1	1.94948	0.219832	9.00E-05	0.001397
RIPOR3	-1.77819	-0.53461	9.19E-05	0.001421
ZFP28	1.03363	1.882186	9.21E-05	0.001421
TFF2	-1.33764	0.553623	9.21E-05	0.001421
HOXD8	6.402483	-3.01478	9.31E-05	0.001435
FOXD4L4	3.369364	-2.36054	9.36E-05	0.001441
OR52A1	6.183638	-3.19309	9.41E-05	0.001448
H4C3	1.90542	-0.13731	9.61E-05	0.001473
RPS20P22	2.881291	-1.44164	9.66E-05	0.001479
TMOD2	-1.47213	0.789106	9.67E-05	0.001479
HSP90AB4P	4.417357	-2.00147	9.70E-05	0.001482
LINC01711	6.823887	-2.70246	9.72E-05	0.001483
MAGEB2	1.042139	1.799188	9.78E-05	0.00149
GRID2IP	-3.46949	-2.43399	0.0001	0.001522
KRT8P46	2.229246	-0.87835	0.000101	0.001522

KLLN	1.41899	0.66081	0.000101	0.001529
GMPR	6.13298	-3.23827	0.000103	0.001551
SLC16A4	-1.36545	0.312546	0.000104	0.001566
AL162615	2.80124	-2.00348	0.000105	0.001576
SFRP1	-6.37478	-3.12306	0.000106	0.00158
ODAM	1.760359	-0.28965	0.000108	0.001616
KDELC1P1	6.812049	-2.71632	0.000109	0.00162
AL096854	-3.26369	-1.81648	0.00011	0.001631
ANXA13	-3.27322	-1.28113	0.00011	0.001632
PON1	1.152927	0.708983	0.000117	0.001732
HSPD1P7	6.156202	-3.23157	0.00012	0.001766
MIR1244-2	2.555659	2.261241	0.00012	0.001773
H4C15	1.249201	2.283961	0.00012	0.001773
KRT8P12	1.018502	2.21435	0.00012	0.001774
RASA4CP	-1.6343	1.651946	0.000122	0.001794
IFITM3P3	6.45431	-2.97307	0.000126	0.001837
TLCD3B	-6.34994	-3.14628	0.000126	0.001843
AL121581	-7.9268	-1.85512	0.000128	0.001868
VWCE	2.110355	-0.96239	0.000128	0.001869
NPC1L1	-1.34368	-0.04702	0.000129	0.001873
OPRD1	3.147892	-2.21492	0.000131	0.001901
TUBBP10	3.205149	-2.01759	0.000131	0.001901
AC003005	6.809585	-2.72178	0.000133	0.001932
FBXO36	-1.12632	1.629944	0.000137	0.001977
SSC5D	3.757538	-1.44353	0.000138	0.001996
CCDC153	-1.05851	1.418804	0.000139	0.002009
GRAMD4P8	6.228486	-3.17897	0.00014	0.002013
PNPLA3	-1.02308	1.519205	0.000141	0.002024
RFPL4AL1	6.958756	-2.61455	0.000144	0.002069
ZNF98	1.134584	0.950089	0.000146	0.002094
LAMC3	-6.09513	-3.32174	0.000147	0.002107
ZNF460	1.223375	1.063213	0.000148	0.002112
ZBTB20	1.522156	0.021942	0.000149	0.002123
KRT18P59	2.856456	-1.87707	0.000153	0.002173
SNORD3C	3.622333	-2.16326	0.000154	0.002183
NGFR	1.602032	-0.26584	0.000154	0.002188
TRIM39-RPP21	10.25581	0.384538	0.000154	0.002191
AL133163	5.959866	-3.34303	0.000154	0.002191
KIF1A	-1.15056	0.690988	0.000157	0.002219
SRGN	-1.17833	0.825691	0.000157	0.00223
ECHDC3	-1.39261	1.176388	0.000158	0.002231
DBH-AS1	-1.26049	0.502418	0.000161	0.002277
GALNT6	-1.02089	1.685719	0.000165	0.002328
METTL27	-1.50989	-0.12349	0.00017	0.002383
AC072061	1.510219	-0.11536	0.000171	0.002393
LINC02709	1.576369	-0.10809	0.000171	0.002399
BHMT	2.303935	-1.40612	0.000176	0.002449
CHST7	-1.08951	1.355208	0.000176	0.002453

MAGEB6	1.416679	-0.09872	0.000176	0.002453
TMC8	-1.08529	1.176371	0.000179	0.002491
GP5	6.177675	-3.18001	0.00018	0.002496
C11orf86	-2.41063	-0.01404	0.000182	0.002517
FUT1	-2.42335	-0.72436	0.000184	0.002537
MT-TH	1.254293	1.806772	0.000184	0.002546
AL391825	1.986838	-0.6972	0.000184	0.002546
OLFML3	2.593689	-1.66294	0.000185	0.00255
TM6SF2	-1.22911	1.097156	0.000187	0.002572
SLC16A6	-1.35418	0.354649	0.000188	0.002587
SLC22A31	-1.65387	-0.24142	0.000189	0.002595
SEMA7A	1.061019	1.474351	0.000191	0.002611
LRRC37A2	1.040087	1.893202	0.000193	0.00264
RPA4	1.916471	-1.2757	0.000193	0.002641
P2RY4	-3.29088	-2.30723	0.000193	0.002641
LINC00528	6.111441	-3.26054	0.000196	0.002677
PCDH19	6.670506	-2.81872	0.000199	0.00271
FLJ31356	3.456586	-2.28154	0.0002	0.002723
LIMS4	8.480908	-1.28273	0.000201	0.002733
ZNF404	-1.1901	0.618063	0.000202	0.002739
PPP1R3G	-3.13203	-2.13147	0.000206	0.002792
LGALS9	1.752739	-0.46425	0.000208	0.00281
ASPHD2	-1.41197	0.072439	0.000208	0.002814
ADGRF4	1.778784	-0.9907	0.000209	0.00283
NRTN	-1.09567	1.602755	0.000211	0.002851
TM4SF1-AS1	-1.10236	1.256469	0.000212	0.002857
CFLAR-AS1	1.43845	-0.03809	0.000212	0.002858
ADM5	1.638261	1.419886	0.000212	0.002861
AZGP1	-1.41793	-0.17862	0.000213	0.002868
CGB2	-1.522	-0.38776	0.000215	0.002884
ORAI1	-1.01429	4.628348	0.000215	0.002889
HMGN2P4	3.547057	-2.67068	0.000219	0.002938
LINC00589	-6.75565	-2.83478	0.000223	0.002975
ZBED6	2.889247	4.790191	0.000224	0.002991
HSPE1P7	6.689436	-2.82299	0.000229	0.003051
AC104958	-1.00931	1.829518	0.000233	0.003094
MIR6821	3.048647	-2.60616	0.000233	0.003095
GRIN3B	-4.03643	-2.07087	0.000234	0.003102
FAM71F2	2.566044	-1.89859	0.000235	0.003109
AKR1B10	2.856302	-1.58497	0.000235	0.003109
RN7SL751P	3.239411	-2.67217	0.000235	0.003115
CT45A7	1.243221	1.830082	0.000236	0.003124
COX6B2	-1.63442	-0.34943	0.000237	0.003132
HNRNPCP1	1.472245	-0.39664	0.000238	0.00314
CREB5	2.982287	-0.99216	0.00024	0.00317
LINC01305	-6.85141	-2.77696	0.000248	0.003242
NPM1P39	1.065324	1.840472	0.000248	0.003242
LINC02761	-5.9062	-3.44895	0.000249	0.003258

RAB3A	-1.0465	1.009188	0.000252	0.003285
MSI1	-1.15787	0.788983	0.000254	0.003298
FUT6	-1.12642	1.277768	0.000255	0.00331
ARHGAP19-SLIT1	-6.16076	-3.26778	0.000258	0.003342
PCED1B	-1.7966	1.409547	0.000258	0.003347
RFPL4A	6.695545	-2.84167	0.000259	0.003356
ADAMTS1	6.787819	-2.75528	0.000264	0.003405
AQP8	6.117409	-3.24308	0.000264	0.003408
Z95331	1.240148	0.562178	0.000265	0.003416
SNHG27	2.508574	-1.81205	0.000267	0.00344
IL17C	5.980644	-3.34836	0.000269	0.003456
TCP11L2	-1.08038	2.020565	0.000272	0.003492
VMO1	-1.24402	0.299198	0.000273	0.0035
AFF1	-1.01138	4.23719	0.00028	0.003572
CHST6	-1.76686	1.907197	0.000283	0.003599
GAPDHP52	3.882511	-2.713	0.000285	0.00362
RNU1-27P	2.394759	-2.03115	0.000289	0.003662
MMP25	1.719101	0.16108	0.00029	0.003667
CCT8L1P	6.014336	-3.32181	0.000293	0.003699
TNF	1.73336	-0.93268	0.000295	0.003727
ALOX5	-1.44451	-0.37095	0.000297	0.003739
ZNF791	-1.98157	2.415329	0.000299	0.003759
BCAR3-AS1	-1.23898	0.872713	0.000299	0.003759
AL008628	6.089932	-3.28358	0.0003	0.003764
AL139274	1.532633	-0.53236	0.0003	0.003767
GOLGA8N	-2.19099	-1.76812	0.000302	0.003782
ZNF497	7.912115	-1.78726	0.000305	0.003812
SMIM2	-5.94895	-3.41788	0.000306	0.003822
MIR3189	-1.44631	-0.42826	0.000308	0.003846
HTRA1	-1.11145	1.253966	0.000308	0.003848
HCAR3	-1.35781	0.060123	0.00031	0.003865
RRN3P2	2.404275	-1.34109	0.000317	0.003934
CD177	-1.61762	-0.53199	0.000317	0.003934
ARHGAP9	2.343749	-0.30987	0.000324	0.003994
SRRM3	-1.02415	1.715102	0.000326	0.004014
TPBGL	-1.41979	-0.00901	0.000326	0.004014
NAIPP2	1.718513	-0.64031	0.000327	0.00403
AL512791	1.698668	-0.9942	0.000329	0.004047
MIR210HG	-1.12918	1.625789	0.00033	0.004051
PAQR6	-1.01741	1.924539	0.000337	0.004121
PRKXP1	2.912478	-2.3891	0.000341	0.004157
LINC00508	-1.17877	0.995669	0.000344	0.004187
EIF4HP2	2.038562	-0.86727	0.000347	0.004211
SEC14L1P1	1.994247	-0.33776	0.000348	0.004231
NUDT16P1	-1.0429	1.481439	0.000351	0.004252
AC092053	2.23028	-1.93855	0.000354	0.004289
RC3H1	1.357708	3.687861	0.000356	0.0043

AC091607	3.958466	-0.578	0.000357	0.004311
LINC01679	3.520475	-1.92293	0.000358	0.004315
AC003006	-6.66608	-2.91568	0.000358	0.004315
LCAT	1.065022	1.617835	0.000358	0.004315
AC023043	-1.18601	0.321476	0.000358	0.004318
SYT17	-1.58933	-0.16604	0.000366	0.004397
FHIT	-1.2218	1.040175	0.000371	0.004447
AP001626	-2.5505	-1.27395	0.000372	0.004452
LINC01132	2.164386	-0.50761	0.000373	0.004459
FAM83A-AS1	6.063408	-3.27442	0.000376	0.004494
CD274	-1.64804	-0.53285	0.000377	0.004497
EPHA1	-1.23324	1.047882	0.000383	0.004564
ZNF551	1.605736	3.451032	0.000385	0.004584
CYP26A1	-1.89011	0.408625	0.000386	0.004588
ULBP2	-1.2945	0.657647	0.000389	0.004627
ZC3H6	-1.10687	0.660066	0.000391	0.004642
AC005532	2.781093	-2.24204	0.000393	0.004656
AGGF1P2	5.832796	-3.44295	0.000398	0.004705
AL139124	4.381159	-2.73183	0.000399	0.004713
SLC25A34	1.731796	-0.38108	0.000412	0.004854
PTHLH	2.403918	-0.84083	0.000413	0.004865
KCNAB2	-1.01811	1.430506	0.00042	0.004936
PRRT1	-1.33046	-0.06583	0.000432	0.00506
AC018742	-6.50489	-3.02319	0.000434	0.005069
PRR15	-1.24684	0.851545	0.000439	0.005123
EMP1	6.580503	-2.91802	0.000439	0.005123
SLIT2	6.471392	-2.97849	0.00044	0.005124
LETM1P2	3.021749	-2.17882	0.000444	0.005161
LINC01488	3.64614	-2.33905	0.000446	0.005174
FDPSP4	3.83312	-2.46802	0.000448	0.00519
HIGD2B	6.560092	-2.91342	0.00045	0.005216
MGAT3	-1.57512	-1.11347	0.000451	0.005223
RTBDN	-1.14006	0.369813	0.000453	0.005242
FRG2B	3.196906	-1.91168	0.000455	0.005253
DPEP2NB	5.982723	-3.33044	0.000455	0.005255
CLDN9	1.439191	-0.29591	0.000456	0.005257
SLC6A13	4.050453	-2.33051	0.000457	0.005262
AIF1L	-1.3573	2.334463	0.000479	0.005488
LINC00167	3.313599	-2.23835	0.000486	0.00555
ZNF772	1.270935	1.006074	0.000497	0.005664
LINC00565	3.569918	-2.64666	0.000507	0.005775
HNRNPA1P54	5.742715	-3.48566	0.000508	0.005776
ZIK1	2.088139	0.662657	0.000514	0.005839
RBM15	-1.07355	2.813314	0.000515	0.005839
TMEM88	2.359794	-0.76117	0.000515	0.005842
PSG8	5.811211	-3.44856	0.000515	0.005842
FBLN2	-1.12837	0.626094	0.000516	0.00585
RSPH10B	3.645813	-2.14761	0.000518	0.005865

GGTA2P	2.5485	-2.18191	0.00052	0.005878
AATBC	2.422329	-1.43043	0.000521	0.005882
CCL2	2.971896	-2.35425	0.000523	0.005899
PPIAP72	6.682323	-2.82434	0.000524	0.005913
MT-TS2	1.492961	1.749436	0.000525	0.005918
SLC1A6	-1.25811	1.647945	0.000526	0.005918
FAM43A	2.423168	-1.2804	0.000526	0.005918
CLEC10A	2.537571	-1.40327	0.000537	0.006023
ASS1P2	3.069361	-2.26229	0.000538	0.006036
PRAF2	-1.06381	4.46559	0.000541	0.006063
SNORD88B	1.505084	0.297971	0.000544	0.006094
ACTBP8	1.481248	-0.43129	0.000545	0.006098
RASA4B	-1.34367	2.785097	0.000545	0.006098
MIR7-3HG	3.784218	-2.47377	0.000546	0.006108
MIR3682	2.803379	-2.4789	0.00055	0.006138
UCP3	1.384508	0.026417	0.000551	0.006146
PCSK6-AS1	6.476157	-2.95289	0.000558	0.006204
ADAMTS13	-1.26388	1.073162	0.000563	0.006256
TSPOAP1-AS1	1.359266	0.084041	0.000567	0.006286
AL133500	8.202373	-1.53341	0.000568	0.006296
CHST8	6.42928	-3.03136	0.00057	0.006316
ZNF503	1.083595	0.83516	0.000571	0.006321
AL354928	1.81194	-1.72338	0.000571	0.006321
SLC22A8	-1.47646	-0.40438	0.000587	0.006483
SYNGR3	-1.36206	2.588298	0.000588	0.00649
UBTFL6	5.700671	-3.51576	0.000589	0.006497
SLC6A19	1.407329	1.122093	0.000592	0.006517
SLIT1	2.143569	-1.98652	0.000597	0.00656
RPL12P37	6.084114	-3.26926	0.000615	0.006733
AL161662	3.196276	-2.31683	0.000615	0.006733
SNURF	1.611329	1.688163	0.000618	0.006757
LINC01004	1.287017	0.198017	0.000623	0.0068
DOK3	1.240212	1.010447	0.000642	0.006969
LAT2	1.230226	0.575352	0.000646	0.007009
NIPSNAP3B	-1.48217	-0.58983	0.000647	0.00701
MYPN	1.611844	-0.41981	0.000647	0.00701
FER1L6	1.676143	-0.86205	0.000648	0.007014
SHH	3.008278	-2.31065	0.000648	0.007014
ELOVL2-AS1	1.054794	0.867176	0.000648	0.007014
AC090970	1.539074	-0.80087	0.000659	0.007127
TMEM61	-2.14727	-0.80496	0.000664	0.007161
ADAM20	6.419048	-2.99586	0.000669	0.007192
KRT18P16	5.724361	-3.4902	0.000669	0.007192
SNORD14D	1.977676	-0.55725	0.000674	0.007233
TIMM23B-AGAP6	2.145736	0.750993	0.000676	0.007246
MSS51	1.823503	-0.86153	0.000678	0.007259
TMEM154	-3.4505	-1.10409	0.000679	0.007263

RPS4XP1	2.218207	-1.931	0.000682	0.007286
RIMS2	6.149237	-3.23379	0.000684	0.007302
CYP4F22	-1.3826	-0.24864	0.000685	0.007313
TRIM40	-2.58852	-1.62206	0.00069	0.007361
CDRT4	3.743007	1.188578	0.000691	0.007361
AL590787	5.856798	-3.43683	0.000692	0.00737
MYO15A	-2.39622	-1.46876	0.000697	0.007412
ETV7	-6.41561	-3.09532	0.000698	0.007416
KLK10	-1.69985	-1.25502	0.0007	0.007433
OTUD7A	-2.88423	-1.578	0.000701	0.007433
DMBX1	5.768706	-3.47886	0.000704	0.007461
GSDMB	-1.12207	2.715728	0.00071	0.007519
AL132777	3.18817	-2.71598	0.000717	0.007581
LINC01315	-1.3494	0.080131	0.000719	0.007594
RUFY4	-5.75238	-3.55068	0.000722	0.007622
OVOL1	3.501999	-1.92028	0.000726	0.007649
FOXD4L1	2.974278	-1.84824	0.000726	0.007651
VEGFD	2.209809	-1.24442	0.000732	0.007694
AC079781	-1.03007	0.965504	0.000733	0.007705
CARTPT	6.377576	-3.0472	0.000741	0.007765
RNF185	-1.26556	5.483838	0.000742	0.007773
SPOCK2	1.090959	0.925053	0.000752	0.00786
HVCN1	-2.63563	-1.75629	0.000755	0.007877
TAF1L	1.563608	0.451693	0.000761	0.00793
PLEKHG6	-1.13351	0.373549	0.000762	0.007936
HTR7P1	1.116762	1.11296	0.000765	0.00796
AC010207	2.000841	-1.6964	0.000772	0.008014
NOXA1	-1.19386	0.11872	0.000772	0.008014
TTLL1	-1.1951	0.471868	0.000779	0.008062
TPRXL	6.614297	-2.84972	0.00078	0.008074
ALPK2	-1.57138	-0.62644	0.000785	0.008112
MIR3648-1	2.344147	-1.85078	0.000788	0.008131
KIF4B	1.280532	0.003494	0.000794	0.008191
ETV3L	6.432702	-3.01625	0.000797	0.008205
AC022028	5.640389	-3.55778	0.000798	0.008214
MIR3658	5.218117	-1.25834	0.000801	0.008234
AL513303	3.244753	-1.32546	0.000802	0.008241
AL354740	4.210232	4.343613	0.000803	0.008241
AC100847	-1.6515	-0.98801	0.000808	0.008281
TMEM265	-2.77399	-2.65053	0.000813	0.008313
H2AC21	3.042826	-2.4639	0.000816	0.008332
AC016245	5.837367	-3.44184	0.000817	0.008343
SEMA3C	1.073192	1.697427	0.000821	0.008373
EXD1	-3.41611	-2.52627	0.000824	0.008404
AURKAP1	1.75948	-0.24945	0.000833	0.008483
B4GALNT4	-1.01412	1.023317	0.000839	0.00853
RNU1-28P	3.073142	-2.2494	0.000845	0.008584
CNFN	-1.10069	0.991547	0.000847	0.008592

SAMD14	1.969127	-1.92058	0.000861	0.008716
RNF208	-1.32563	0.984549	0.000862	0.008719
HSPA8P5	1.229715	0.194001	0.000862	0.008719
ADAMTS7	-1.93477	-1.04876	0.000862	0.008719
AL139805	2.322624	-1.80203	0.000865	0.008744
LINC00336	6.37395	-3.07674	0.000867	0.008757
METTL14-DT	6.554873	-2.96391	0.000872	0.008798
TPPP2	-3.58849	-2.84168	0.000877	0.008828
CALCRL	-1.4643	0.193627	0.000888	0.008923
FOXD1	2.008415	-0.85225	0.000892	0.008941
NINJ2	-1.35184	0.511244	0.000904	0.009041
ANAPC1P4	-8.10695	-1.66403	0.000907	0.009063
FBXW10	6.24754	-3.12903	0.000907	0.009063
AC068506	-1.44563	-0.22282	0.000928	0.009236
TCF7L1	-1.42538	-0.2358	0.000931	0.009256
LINC01909	1.831198	-1.80743	0.000937	0.0093
MEI1	-1.70511	-1.25467	0.00094	0.009318
CCIN	6.130676	-3.23998	0.000957	0.009465
GPR132	-1.31978	-0.14033	0.00096	0.009479
MBNL3	1.359676	0.577813	0.00096	0.009479
PKIA	-1.07107	0.41927	0.000963	0.009497
ZNF672	1.540642	4.232076	0.00097	0.009559
FDPSP1	-2.2731	-1.66555	0.000972	0.009569
DCAF8L1	1.086651	0.177962	0.000977	0.009609
GAS6-DT	3.295991	-2.12899	0.001001	0.009805
PTPRN	1.691425	-1.23804	0.001002	0.009805
SOST	6.235943	-3.13255	0.001004	0.009813
AP002851	1.523161	-0.27048	0.001004	0.009813
PTGR1	1.15895	0.990491	0.001007	0.009833
TMEM51-AS1	-1.3552	-0.02446	0.001013	0.009878
KRT18P63	3.266221	-1.88655	0.001014	0.009892
PCP4L1	-1.23301	0.551894	0.001017	0.009913
EPHX4	-1.1133	0.226819	0.001019	0.009923
ZNF462	6.316263	-3.12265	0.00102	0.00993
GDF9	1.140225	0.144736	0.001025	0.00997
AC135721	-2.42241	-1.77905	0.001027	0.009976
FBXO27	-1.32733	2.801832	0.001033	0.010024
AC134312	4.664639	-3.35586	0.00104	0.010086
AC092301	-6.30489	-3.17565	0.001042	0.010096
LINC02475	-2.52545	-1.18946	0.001045	0.010103
EGR3	2.487458	-1.88984	0.001063	0.01025
NPTX1	2.206992	-0.7497	0.001067	0.010282
CORIN	6.255901	-3.14067	0.001069	0.010301
RPL35AP26	5.668179	-3.54409	0.00107	0.010307
FBLL1	1.584497	-0.38652	0.001074	0.010334
Z83847	2.269723	-2.05232	0.001076	0.01034
NPIPB12	1.16421	2.508852	0.001078	0.010343
TMEM240	2.967465	-1.65291	0.001078	0.010343

CLCP2	5.926759	-3.40024	0.001087	0.010408
SNAP25-AS1	-1.08139	1.391865	0.001089	0.01042
RN7SKP203	5.547057	-3.61552	0.001091	0.010431
NCF1B	6.343006	-3.08533	0.001101	0.01052
AC139100	1.907161	-2.09268	0.00112	0.010686
AC005180	5.62721	-3.5539	0.001123	0.01071
ZNF804A	3.428139	-2.75424	0.001125	0.010726
HAVCR2	-1.3522	-0.5728	0.001136	0.010817
EOMES	2.304113	-1.74849	0.001138	0.01083
IRF1-AS1	-2.17572	-1.29346	0.001143	0.010868
HOXB9	1.272815	-0.33899	0.001152	0.010914
ZNF528-AS1	2.943316	-2.08043	0.001176	0.011096
AC006252	-1.172	0.3685	0.001182	0.011149
LINC00942	6.248696	-3.12918	0.001189	0.011195
ARHGDIB	1.873456	-1.06863	0.001194	0.011229
CREB3L2	-1.11165	6.300199	0.001201	0.011292
TBX21	6.176207	-3.19511	0.001207	0.01134
AL133243	1.492369	-1.11551	0.001221	0.011453
WNT2B	-1.3736	0.32733	0.001222	0.011462
AC004854	2.600941	-2.48601	0.001225	0.011481
RPL23AP6	3.480953	-2.70499	0.00123	0.011505
RN7SK	1.531387	0.799173	0.001234	0.011533
TMEM63C	-2.0407	-0.27161	0.001236	0.011546
CRISPLD2	3.272492	-2.07921	0.001247	0.011628
TTYH1	3.856499	-3.11406	0.00125	0.01164
AGGF1P1	5.551689	-3.6148	0.001257	0.011686
PIGR	-1.57312	-1.39472	0.001258	0.011686
Z92544	1.88627	-0.86767	0.001259	0.011692
TRIM72	6.273225	-3.16591	0.001259	0.011693
HNRNPA1P30	6.180475	-3.19306	0.001265	0.011727
AL161938	6.267266	-3.10936	0.001271	0.011763
KPNA2P3	-7.90466	-1.84466	0.001276	0.011796
FCRLA	2.958817	-2.36832	0.001277	0.011796
MT-TL2	1.556805	2.088278	0.001296	0.011934
DDX50P1	1.831171	-1.0569	0.001306	0.012
HAS1	6.159905	-3.19925	0.001314	0.012054
AC092073	-8.00762	-1.78031	0.001338	0.012247
CDH5	-1.22669	-0.08422	0.001341	0.012262
SPDYE13	6.106443	-3.23012	0.001356	0.012378
LHFPL3-AS2	-1.48582	-0.04194	0.001358	0.012389
AC027228	1.081324	0.700316	0.001371	0.01248
LINC02105	-6.37611	-3.13873	0.001375	0.012512
AC097717	3.991017	-3.00704	0.001378	0.012528
RHOQ-AS1	-1.38651	-0.78288	0.001407	0.012767
RNVU1-25	3.873713	-3.09743	0.001419	0.012856
AP000944	2.031517	-1.68426	0.001435	0.012969
NLGN3	1.414403	0.224181	0.001436	0.012973
RASL10A	3.368796	-2.02788	0.001456	0.013127

LCAL1	3.644823	-2.60799	0.001459	0.013144
B3GNT4	-1.00684	1.336153	0.00146	0.013144
SETP17	3.385115	-2.77455	0.001469	0.013198
SPTBN5	1.675134	-1.12618	0.001487	0.013324
SSPOP	-1.0268	0.95876	0.001498	0.013401
LAG3	1.585348	-1.33757	0.00151	0.013493
AC124068	1.595954	-0.92332	0.001513	0.013501
AL662797	1.51004	-0.02867	0.00155	0.01379
AL137784	-1.83756	0.142396	0.001551	0.013798
AL139220	-2.04216	-1.70669	0.001557	0.013831
NRBF2P6	1.854317	0.263783	0.00156	0.01386
TGM4	6.078449	-3.2537	0.001563	0.013876
SMCR2	6.150571	-3.23421	0.001567	0.013891
AL732509	-1.16674	0.272851	0.001595	0.014113
AC137936	4.400127	-3.26041	0.001597	0.014122
ENPP3	-2.24616	-1.395	0.001601	0.014152
LMX1B	-1.29178	-0.14769	0.001605	0.014179
GIPC3	6.174722	-3.18034	0.001608	0.014199
LINC02614	-1.39313	-0.97485	0.001616	0.014246
AP001025	6.133969	-3.23887	0.001624	0.014299
AC011455	-1.17377	-0.32413	0.001641	0.01441
TBC1D3I	7.007858	-2.60312	0.001682	0.014698
ACTG1P14	3.659193	-2.88766	0.001685	0.014717
FAM149A	-1.45459	-0.31354	0.001701	0.014844
GSTA3	-2.06911	-0.46531	0.001704	0.014863
PHYHD1	3.519272	-2.70409	0.001706	0.014873
LINC01770	-1.0614	0.255126	0.00172	0.014974
CCER2	1.57067	-0.4258	0.00174	0.015131
PZP	-1.21105	-0.20753	0.001745	0.015166
AP002381	1.250858	-0.37709	0.001748	0.015192
ZNF844	1.431088	-0.5653	0.001749	0.015196
GALNT18	-1.3672	-0.81332	0.001782	0.015435
CICP13	5.564354	-3.58999	0.001795	0.015526
SYCP3	6.208199	-3.16996	0.001796	0.015528
PSME2P2	1.180903	-0.26605	0.001812	0.015622
DDR2	1.060774	1.115967	0.00182	0.015672
CT45A5	1.245357	2.061711	0.001829	0.01572
LINC01659	-1.16252	0.219723	0.001829	0.01572
BLACAT1	-6.10348	-3.31957	0.00183	0.015721
AC069224	1.577444	-0.76191	0.001832	0.015737
HAS3	1.530106	-0.12579	0.001837	0.015772
SETBP1-DT	-1.61794	-1.52928	0.001838	0.015772
TCHH	3.279024	-2.23458	0.001838	0.015772
LINC01882	1.056416	0.537484	0.001865	0.01597
HCLS1	2.775243	-1.40495	0.001871	0.015996
AC009133	2.005275	-1.21736	0.001872	0.016003
AC012676	1.285128	-0.82527	0.001878	0.016046
POTEM	1.73938	-0.59705	0.001879	0.016046

INMT-MINDY4	-7.7901	-1.97462	0.001893	0.016145
MED8-AS1	-1.25728	-0.6303	0.001894	0.016147
ZNF841	1.131882	2.947972	0.001895	0.016147
INSYN1	1.69781	-0.94965	0.001921	0.016336
AL023806	1.731899	-1.65196	0.001929	0.016394
AC084876	1.448477	-1.18173	0.001947	0.016529
PLPPR3	-1.06168	1.153374	0.001957	0.016598
RNU1-2	1.668829	-1.68703	0.001967	0.016667
SLC5A11	2.055421	-0.46911	0.00197	0.016681
RNVU1-6	3.36069	-2.55659	0.001978	0.016743
CD79A	2.015037	-0.61433	0.002005	0.016944
EN2	1.643082	-1.37316	0.002035	0.017165
KITLG	1.388561	-0.56966	0.002039	0.017192
ATP6V1FNB	1.112224	-0.26111	0.002045	0.017228
DENND6A-DT	6.039689	-3.2983	0.002052	0.017264
PRSS35	1.729442	-1.42151	0.002069	0.017359
ZNF628	-1.41092	2.106915	0.002072	0.017372
NTN1	-1.0422	0.505141	0.002075	0.017382
SPATA12	1.153802	0.23919	0.002098	0.017536
LINC02158	6.002428	-3.30765	0.002101	0.017543
SPICE1	-1.25797	2.992496	0.002112	0.017626
NEK2P4	2.007602	-2.08929	0.002112	0.017626
BGLAP	2.699377	-2.68297	0.002135	0.017771
MAGEB17	6.040027	-3.31508	0.00214	0.017797
TSPAN18	-1.31541	-0.96249	0.002145	0.017829
AL049775	1.052252	0.884268	0.002155	0.017901
FNDC7	6.018552	-3.3212	0.002162	0.017943
RAB4B-EGLN2	3.623479	0.618601	0.002164	0.017954
AC027702	3.506911	-2.74169	0.002169	0.017988
RUNDC3B	2.108745	-1.29097	0.002177	0.018048
KCNH2	-1.27585	-0.6406	0.002201	0.018214
ZNF562	1.287055	3.794057	0.002206	0.018233
SCRT1	6.092026	-3.25006	0.00223	0.018399
PYY	-1.92364	-1.99616	0.00224	0.018456
ARHGEF35-AS1	-1.40237	3.925997	0.002247	0.018506
COL11A2	1.525347	-0.41128	0.002249	0.018512
TPD52L1	-1.20526	0.192282	0.002257	0.018567
BMS1P17	2.51578	-0.32116	0.002261	0.018592
CYP4A22-AS1	-6.12092	-3.29631	0.002291	0.018788
AC078883	-2.24597	-2.10424	0.002316	0.018968
VHLL	5.914764	-3.366	0.002329	0.019057
DENND6B	-1.10779	0.015588	0.002333	0.019069
LINC01764	1.292807	-0.01366	0.002348	0.019162
AL589666	1.232472	0.658912	0.002353	0.019201
PEAR1	-1.31227	-0.45891	0.002358	0.019227
PPIAP3	2.882684	-2.72692	0.002362	0.019232
CENPVL2	5.378782	-3.72252	0.002377	0.019339
AL513348	5.965016	-3.3533	0.002433	0.019691

CATIP-AS1	-1.0932	0.583181	0.002434	0.019691
TDRD6	5.957829	-3.37321	0.002439	0.019707
KRT8P28	5.347416	-3.7291	0.002443	0.01973
BCAR4	5.98162	-3.31377	0.00246	0.019842
NDUFA4L2	-1.2057	0.332269	0.002465	0.019875
LINC00943	3.534762	-2.67918	0.002466	0.019884
MIR3648-2	2.828278	-2.47855	0.002469	0.019896
BTG3-AS1	1.208867	-0.57312	0.00247	0.019896
NOS3	-1.28447	-0.54846	0.002483	0.019982
LINC00514	1.769049	-0.62567	0.002492	0.020049
NXPH4	-1.14283	0.681688	0.00252	0.02024
RAB43	-1.30649	3.610738	0.002527	0.020281
EXOSC6	1.774211	2.13526	0.002528	0.020287
RSPH10B2	2.77004	-2.02043	0.00259	0.020716
DFFBP1	1.628794	-0.99405	0.002597	0.020762
CGB7	-1.18424	-0.2999	0.002604	0.020796
RNF138P1	3.691041	-3.20987	0.002607	0.020801
RNF151	2.695562	-2.20511	0.002627	0.02093
AC090181	1.860546	-1.88891	0.002629	0.020937
SLCO5A1	5.944272	-3.359	0.002652	0.021095
COL17A1	3.414805	-2.76322	0.002665	0.021181
AC010619	4.848931	-3.40258	0.002673	0.021223
AC002350	1.193699	-0.68016	0.002687	0.021322
NCCRP1	1.250182	-0.0862	0.002707	0.021427
DHFR2	-1.57554	1.584795	0.002709	0.021433
AC012443	1.094957	-0.00925	0.002715	0.021469
AL445931	5.889929	-3.37247	0.00272	0.021495
CRACDL	-1.40379	-0.86418	0.002724	0.021511
AL354836	1.271642	-0.25276	0.00273	0.021533
AL161431	-1.4315	-0.79656	0.002751	0.021671
TMEM163	5.930471	-3.38056	0.002799	0.021971
AL669818	5.276389	-3.7688	0.002816	0.022084
LBH	6.66477	-2.79922	0.002832	0.022178
LRIT3	5.92546	-3.36347	0.002844	0.022237
SYCE1L	-1.39304	-1.02339	0.002848	0.02225
COPZ2	2.728362	-1.95002	0.002851	0.022268
SALL3	5.27023	-3.77004	0.002866	0.022351
ECT2L	3.350692	-2.80369	0.00287	0.022366
VN1R1	5.81634	-3.42792	0.002873	0.022382
ANKRD65	6.072366	-3.2398	0.00291	0.022603
SMIM1	-1.09528	0.926068	0.002915	0.022632
AL136038	1.832136	-1.9915	0.002927	0.022712
IL3RA	1.617185	-1.36659	0.002947	0.022847
AC018523	6.803592	-2.6913	0.002953	0.022881
SLC2A1-AS1	-2.25502	-1.60286	0.002957	0.022911
GMCL1P2	5.281207	-3.76762	0.002964	0.022953
LTA	2.595977	-1.41781	0.00297	0.022991
AL031709	5.32401	-3.75913	0.002973	0.023003

MMP10	5.344038	-3.7298	0.002975	0.023015
Z73429	-3.40043	-2.75893	0.002981	0.023054
ZBTB37	1.609235	2.973918	0.002983	0.023056
AL162419	5.912343	-3.34883	0.003068	0.02361
AC007365	2.033752	-1.2147	0.003086	0.02373
SYT3	-1.03399	0.040221	0.003097	0.023808
ARHGAP30	1.183818	0.001354	0.003112	0.023891
KRT78	5.883436	-3.41108	0.003135	0.024042
AC096633	5.888774	-3.37244	0.003139	0.024055
LGALS4	1.116151	0.392437	0.00316	0.024194
RPL7P4	5.304494	-3.76287	0.003166	0.024238
SH2D3C	-1.47565	-0.21531	0.003184	0.024356
PKD1L3	5.894302	-3.37167	0.003212	0.024523
SLC4A1APP1	-1.65178	1.256691	0.003223	0.02459
HSPE1P6	5.35196	-3.72847	0.003249	0.024763
KIF26B	3.130276	-2.19936	0.003255	0.02479
OVGP1	-1.16066	-0.11997	0.003265	0.024859
NAV3	5.777944	-3.45659	0.003285	0.02499
TMPRSS9	1.727748	-1.40577	0.00335	0.025412
ZSCAN18	1.125399	0.02334	0.003353	0.025431
ACP5	-1.12923	-0.34727	0.003371	0.025545
SYNC	-1.34284	-0.71604	0.003372	0.025552
LILRB3	2.073798	-2.0456	0.003381	0.025611
PELI2	3.490106	-2.7341	0.003382	0.025611
LMF1	-1.18623	0.317219	0.003392	0.025659
HSD17B3	5.88235	-3.37406	0.003403	0.025728
AC027290	1.654661	-1.31429	0.00342	0.025844
AP003774	5.250948	-3.78641	0.003422	0.025845
TBILA	1.093875	0.286662	0.003438	0.025931
LINC00672	1.562726	-1.03815	0.003442	0.025944
SLC12A5-AS1	-1.58337	-1.49619	0.00347	0.026109
AC012510	1.123589	0.19144	0.003491	0.026234
AC232271	-1.11267	-0.29956	0.003493	0.026239
TMCO1-AS1	1.16303	0.02399	0.003498	0.026266
ZNF227	1.024968	3.884885	0.003503	0.026296
PTMAP4	1.158963	0.094028	0.003556	0.026591
AP000424	-1.23961	-0.64065	0.003561	0.026613
PALMD	1.847881	-1.45633	0.003573	0.026684
CALHM2	1.12781	-0.30723	0.003579	0.026699
CCNT2-AS1	1.139624	0.445432	0.003588	0.026762
AC009452	6.007242	-3.32368	0.0036	0.026825
GZMM	2.57619	-2.04247	0.003604	0.026849
TSPOAP1	6.022694	-3.33753	0.003607	0.026862
C3orf86	-1.04188	1.045685	0.003636	0.027044
TNFRSF14	-1.04016	0.598045	0.003662	0.027209
ANKRD53	2.993919	-2.84147	0.003663	0.027209
DBH	-1.32869	-0.69587	0.003668	0.027235
TEX29	5.946874	-3.3769	0.003683	0.027314

PYGO1	1.048394	1.209716	0.003709	0.027464
ST8SIA3	5.71075	-3.4926	0.003713	0.027479
F13B	1.094576	0.821574	0.003713	0.027479
RGMA	1.869228	-0.58091	0.00372	0.027513
KCNAB3	1.035414	0.084575	0.003721	0.027513
TUBA3FP	1.036061	0.412623	0.003729	0.027559
MAP1LC3A	3.307143	-2.83689	0.003754	0.027694
CDH4	1.067307	0.172915	0.003756	0.0277
PAX5	5.892312	-3.40967	0.003779	0.027818
AC016642	-1.0631	0.216323	0.003783	0.02784
H2BC8	1.588879	-0.41891	0.003818	0.028015
SERPINE2	1.055972	-0.12419	0.003853	0.028219
AL359736	-7.44032	-2.29772	0.00386	0.028263
AC016831	1.235319	-1.02239	0.003912	0.028585
AL033523	2.982889	-3.05195	0.003937	0.028723
CDC37P1	-1.13653	-0.16094	0.00396	0.028872
FOXL2	3.48612	-2.71337	0.003981	0.029003
HMGN3-AS1	1.105771	0.264818	0.003988	0.029041
CTRB2	5.766795	-3.49981	0.004059	0.029475
RPS27AP8	5.200182	-3.80969	0.004064	0.029504
AC234031	-5.77031	-3.54674	0.004067	0.029516
AC084734	5.763644	-3.46022	0.00408	0.029582
HLX	5.752373	-3.48326	0.004105	0.02974
ZNF433-AS1	1.80973	-1.60505	0.004121	0.029836
GSTA1	1.436996	-0.20683	0.004131	0.029882
TGFA	5.907994	-3.40468	0.004148	0.029995
TESK2	-1.05698	0.904759	0.00415	0.029997
LINC00242	1.277129	-0.14003	0.004156	0.030033
ZNF135	2.554352	-1.60346	0.004199	0.030302
GIPR	-1.04656	1.900172	0.00423	0.030462
C1orf210	-2.00072	-0.94058	0.004253	0.030598
LINC01140	-3.10791	-2.62159	0.004301	0.030907
EPB41L4A	-5.05371	-1.32972	0.004307	0.030945
DUX4L9	5.132791	-3.84926	0.004335	0.031118
NOVA2	-1.37835	-0.9812	0.004339	0.031135
WFIKKN2	5.769609	-3.49968	0.004345	0.031171
AL135925	1.190765	-0.46256	0.004348	0.03118
AKR1B1P7	5.831102	-3.4057	0.00435	0.031182
TBC1D29P	5.833577	-3.42433	0.004369	0.031294
ABCB6	1.044407	0.929264	0.004411	0.0315
NTNG2	1.558738	-1.1437	0.004416	0.031523
SDAD1P1	-1.08078	0.067543	0.004439	0.031662
AL354920	1.159861	0.104697	0.004445	0.031675
AL133477	1.402315	-1.62112	0.004447	0.031675
KRTAP5-AS1	2.298164	-1.54819	0.004447	0.031675
RIC3	2.705	-2.30134	0.004448	0.031675
MSX1	1.951484	-1.61366	0.004448	0.031675
CHRM5	2.165346	-1.63354	0.004451	0.031676

H4C14	2.001642	1.905586	0.004451	0.031676
AC099509	-1.67251	-1.38879	0.004494	0.031899
CFP	2.997634	-1.82776	0.004496	0.031906
NME5	5.189193	-3.81153	0.004498	0.031906
CDHR1	-5.79236	-3.51878	0.004516	0.031985
FP326651	-1.96481	-1.63478	0.004525	0.03203
AC020893	3.560445	-3.28948	0.004528	0.032042
TEX35	1.616569	-1.71951	0.004536	0.032071
CCDC81	2.619581	-1.90799	0.004548	0.032133
H2AC14	1.478627	-0.77568	0.004561	0.032218
ZFP91-CNTF	-7.16427	-2.47893	0.004565	0.032228
FABP6	-1.131	-0.42836	0.004582	0.032312
CT867976	5.745148	-3.46429	0.004588	0.032338
AC026803	1.516275	-1.36022	0.004588	0.032338
Z84466	6.596253	-2.8882	0.004597	0.032376
IRX4	5.859229	-3.39903	0.004624	0.032529
OBSCN-AS1	-2.01513	-1.33837	0.004638	0.032607
KCNV2	2.626114	-2.75523	0.004639	0.032607
DNAJC5B	1.191415	-0.23553	0.004643	0.032621
ZNF84	1.344228	4.156989	0.004661	0.032697
USP8P2	3.917626	-3.58076	0.004673	0.032775
NCOA4P2	5.698158	-3.53723	0.004701	0.032919
PTAFR	-1.01004	0.885357	0.004702	0.032921
CASKIN1	-1.04997	0.434133	0.004708	0.032943
LINC01176	3.223262	-2.91167	0.00471	0.032944
ZNF577	1.005284	1.113829	0.00472	0.033
KRT86	-1.12937	0.128379	0.004733	0.033058
ZNF534	5.621676	-3.5546	0.00476	0.033215
ACTL7B	5.684198	-3.51955	0.004782	0.033333
RPS3AP2	5.209131	-3.8078	0.004787	0.033355
AL390026	5.148118	-3.84621	0.004814	0.033523
MROH2A	5.792879	-3.49392	0.004816	0.033523
AL441883	7.357774	-2.26117	0.00482	0.033537
AC007611	-1.13747	-0.18854	0.004856	0.033725
H3C1	3.155244	-2.201	0.004869	0.033779
NRADDP	2.481483	-2.25478	0.004901	0.033945
SLC39A2	5.723476	-3.51001	0.004923	0.034066
DHH	5.747037	-3.48366	0.004928	0.034093
AC015912	1.42616	-1.39483	0.004941	0.034172
AC010285	5.305437	-3.73748	0.004944	0.034184
MIA	6.398941	-3.00117	0.004956	0.034237
YBX1P4	3.748808	-3.19727	0.005013	0.034545
CD22	-1.26906	-0.66799	0.005057	0.034768
BTN3A3	-1.22123	2.325094	0.005088	0.034936
TSPY26P	1.378303	0.227152	0.005089	0.034936
RGCC	-2.69544	-1.83467	0.005109	0.035054
AL133390	1.882243	-0.95778	0.005157	0.035272
LINC00632	-1.42254	-1.41781	0.00518	0.035383

CSNK1G2-AS1	5.771011	-3.49878	0.00519	0.03541
SLC2A12	-1.81901	0.989977	0.005266	0.03582
NDUFAF4P1	4.502601	-3.20487	0.005301	0.035986
PPIC-AS1	5.619558	-3.55548	0.005315	0.036062
HIGD2AP1	6.07487	-3.30654	0.005333	0.036163
RAB40AL	1.572003	-0.95231	0.005351	0.036249
PXDN	-5.69086	-3.58757	0.005362	0.03627
GAU1	5.651329	-3.52698	0.005365	0.036281
PNMA5	-5.67697	-3.59063	0.005395	0.036408
HEXA-AS1	1.65275	-0.97875	0.005421	0.036559
LYPD1	5.641556	-3.5721	0.005429	0.036603
AL772307	7.254877	-2.32065	0.00544	0.036665
AC092268	-1.61957	-1.85929	0.005454	0.03674
LINC02067	4.454492	-2.2437	0.005484	0.036901
HTRA3	1.455968	-1.59614	0.005486	0.036903
FOXD4	2.183146	-2.45365	0.005504	0.036995
FAM3B	5.791974	-3.49446	0.005532	0.037152
C5orf24	1.717229	3.095007	0.005535	0.037156
AC022217	2.287009	-2.85895	0.005548	0.037228
SLC52A1	-5.72057	-3.58139	0.005563	0.0373
KCCAT198	-5.78037	-3.54467	0.005563	0.0373
DLX6	5.238267	-3.77606	0.005568	0.037322
ESAM	1.0792	-0.15402	0.005577	0.037366
CNKSR1	1.354755	0.053516	0.005587	0.037401
SOSTDC1	-5.71687	-3.55835	0.005606	0.037517
KLHDC7B-DT	5.803094	-3.43061	0.005629	0.037641
ISM2	-1.35229	-1.22668	0.005692	0.037986
AL031281	-3.47281	-0.82443	0.005695	0.037987
AC131971	2.327774	-2.46266	0.005703	0.038014
NT5C1B	-1.8722	-2.27564	0.00572	0.038098
FYN	-1.12812	0.065755	0.005743	0.038201
AC084880	1.58849	-1.80276	0.005768	0.038352
PRKAG2-AS1	-1.44321	-0.57255	0.005828	0.038673
SH2D7	-1.09441	-0.63068	0.005839	0.038727
WASIR1	-5.56569	-3.66316	0.005842	0.038728
MMP19	1.032161	0.385175	0.005868	0.038853
ACTL6B	5.70291	-3.51482	0.005873	0.038879
AC022816	2.375746	-1.45067	0.005904	0.039006
PAG1	1.880834	-1.47319	0.005913	0.039059
CENPVL1	5.157732	-3.84438	0.005924	0.039121
RPSAP55	1.450082	-1.4988	0.005945	0.039239
ABCA6	-1.20723	-0.83844	0.005958	0.039316
EEF1AKMT3	-1.08312	1.873907	0.005996	0.03953
CXCR4	2.727372	-2.53918	0.006034	0.039716
HMGB1P31	-1.85806	-2.04058	0.00605	0.039792
SDK2	-1.32834	-0.35047	0.006053	0.039802
HEPH	-1.16034	-0.31274	0.006058	0.039824
RNVU1-30	2.031585	-1.99284	0.006092	0.040018

JPH3	2.41965	-1.41717	0.006093	0.040018
TP53AIP1	5.595896	-3.56067	0.00611	0.04012
AC245041	1.731164	-1.31082	0.006124	0.040203
GPR135	1.026995	0.363087	0.006132	0.040244
LBHD1	-1.05207	5.391861	0.006142	0.040299
RPSAP61	1.147126	-0.70391	0.006156	0.040378
RNU6-8	1.275615	-1.02829	0.006164	0.040403
PRR5L	5.573598	-3.6102	0.006181	0.040466
LINC01532	3.278386	-2.86829	0.006194	0.040533
PIANP	-2.55919	-1.92446	0.006204	0.040587
AF196969	-3.89082	-1.75639	0.006214	0.040643
TFDP3	3.345844	-2.82375	0.006307	0.041149
ARMS2	5.795876	-3.45194	0.006332	0.04127
SAG	5.606034	-3.58071	0.006344	0.041324
AL450384	1.154813	-0.7705	0.006387	0.041561
LRMDA	-3.24612	-3.01939	0.006436	0.04182
HSF5	5.626944	-3.55392	0.006473	0.042044
AC080038	4.216321	-3.62231	0.006478	0.042063
LINC02361	-1.93181	-1.93015	0.00649	0.042118
EEF1A1P3	5.166848	-3.84302	0.0065	0.042173
LINC01876	-1.01036	0.332978	0.00654	0.042385
MYH7B	1.219973	-0.96122	0.006554	0.042446
RHBDL1	1.492359	-1.15348	0.006556	0.042446
AC012254	2.248804	-0.49662	0.006558	0.042449
SYCE2	-1.02531	-0.33849	0.006574	0.042528
BCHE	-1.30924	-0.32832	0.006639	0.042867
TUBA3E	5.650248	-3.56999	0.006649	0.042892
TTC6	1.041227	0.023741	0.006657	0.042925
AL450992	5.044482	-3.90272	0.00667	0.042987
FAM180B	5.804096	-3.45112	0.006728	0.043293
CASP16P	-1.25919	0.225183	0.006744	0.043386
AL670729	1.760135	-2.11494	0.00676	0.043469
SOCAR	5.534581	-3.61879	0.006771	0.043524
CCNA1	6.363937	-3.08057	0.006847	0.043921
GLS2	-1.92591	-1.7082	0.006869	0.044037
CST7	5.545625	-3.61644	0.006893	0.044156
PGAM1P6	5.040451	-3.89379	0.006909	0.04424
YAP1P1	5.540761	-3.59459	0.006938	0.04438
ACRBP	2.798988	-2.26739	0.006944	0.044404
ANKLE1	1.303499	-0.71924	0.00695	0.044418
NPAS4	5.529818	-3.6196	0.007005	0.044655
HSPB7	5.731252	-3.46803	0.007021	0.044715
AC005775	-1.68196	-0.02421	0.007049	0.044842
C1orf220	1.820279	-0.26893	0.007084	0.045016
PLAG1	-1.06828	0.516402	0.007107	0.04512
IMPDH1P8	1.271864	-1.19158	0.007108	0.04512
LINC02747	2.017093	-1.46561	0.00711	0.04512
CTRL	1.194944	0.629754	0.007133	0.045233

AC016821	-2.67011	-2.71746	0.007137	0.045233
C20orf203	5.612399	-3.55667	0.007141	0.045246
ANAPC1P6	-6.50847	-3.03663	0.007227	0.045701
LINC02594	-3.26471	-2.97327	0.007227	0.045701
LINC01087	1.340422	-0.86822	0.007242	0.045757
RPSAP2	5.586611	-3.56255	0.007328	0.046203
SLC13A2	-1.54342	-0.42616	0.007406	0.046593
A2MP1	3.50474	-2.69658	0.007421	0.046639
AC131159	2.031383	-2.55494	0.007441	0.046747
SNORA50C	1.393575	-1.3016	0.007488	0.047021
CPAMD8	-1.11747	-0.14957	0.007511	0.047117
AC020604	3.302989	-2.88796	0.007568	0.047387
CTAGE3P	1.203037	-0.65912	0.007578	0.047417
FOXG1-AS1	5.535563	-3.59574	0.007608	0.047555
PDE6B	-1.52776	-1.30944	0.007644	0.047716
AC139149	3.197426	-2.72195	0.007658	0.047754
ZNF670-ZNF695	1.05428	0.002913	0.007666	0.047768
NDUFB4P11	5.139892	-3.84795	0.007744	0.048131
FGF19	-1.15836	-0.9726	0.007769	0.048241
HOXA11	5.563548	-3.63561	0.007784	0.048284
AL359182	5.521495	-3.6446	0.007802	0.04836
AL807752	2.227054	-2.78007	0.007831	0.048515
RFX4	5.496298	-3.62645	0.007889	0.048822
IL17RE	1.185099	-1.02977	0.007895	0.048851
NPM1P29	5.473914	-3.65412	0.007913	0.048932
LINC01436	-5.42838	-3.74277	0.007915	0.048934
LINC00598	-1.07435	-0.19809	0.007959	0.049159
BDKRB2	-1.05462	5.480755	0.008098	0.049938
NECTIN4	6.185028	-3.17662	0.008108	0.049986

Table S7.1 Clinical characteristics for patients with small bowel vessel information in Cohort 1

Clinical characteristics	Total (n = 82)
Age, Median (Q1,Q3)	61 (56, 64)
Marriage, n (%)	
No	2 (2.44)
Yes	80 (97.56)
Sex, n (%)	
Male	37 (45.12)
Female	45 (54.88)
Number of bleeding in 1 year before enrollement, n (%)	
4	12 (14.63)
5	25 (30.49)
6	30 (36.59)
7	10 (12.2)
8	4 (4.88)
10	1 (1.22)
Average Hb concentration in 1 year before enrollement (g/L), Mean ± SD	81.7 ± 12.82

Table S7.2 Clinical characteristics for patients with small bowel vessel information in Cohort 2

Variables	Total (n = 10)
Age, Median (Q1,Q3)	59 (52.25, 69)
Marriage, n (%)	
No	0 (0)
Yes	10 (100)
Sex, n (%)	
Male	4 (40)
Female	6 (60)

Table S7.3 Clinical characteristics for patients with small bowel vessel information in Cohort 3

Variables	Total (n = 30)
Age, Median (Q1,Q3)	62 (54.25, 65.75)
Marriage, n (%)	
No	1 (3.33)
Yes	29 (96.67)
Sex, n (%)	
Male	11 (36.67)
Female	19 (63.33)

Table S8 Sequences of primers, siRNA, and shRNA used in the study.

**Sequence of primers used for real-time PCR**		
Gene	Primer (Forward)	Primer (Reverse）
HGDILnc1 (MSTRG.6431.1)	GCCTACCATTCATTCTACCGT	GGCAGACTCCAGAGGAAGTTA
lnc-STK25-2:1	GTGTGTTGGGTGTTGCGTATGTAC	ACAGCCCCAATACACACATGCC
MSTRG.67348	TCACTCTTCTTTCCCAGGCTGGAG	GAGGCAGGAGAATCGCTTGAACC
VEGFA	AGGGCAGAATCATCACGAAGT	AGGGTCTCGATTGGATGGCA
TDGF1	TTTGAACTGGGATTAGTTGCCG	GGGGCCAAATGCTGTCATCT
ADAMTS9	ATTAGAGACCCTGAGCGAATACG	GAAGTGGACGTTCGTGGGAA
E2F2	CGTCCCTGAGTTCCCAACC	GCGAAGTGTCATACCGAGTCTT
SIM2	CCATTTAGGCTTATCCCAGGTG	GGTCATCTCATCGTGGTCAGA
NeuroD1	ATGACCAAATCGTACAGCGAG	GTTCATGGCTTCGAGGTCGT
CBX2	GCGAATTGAAGGGCTAAGA	GGGCTTGGAGAGCTGTTAGTC
ZFP57	CAACATCCCAGTCTGAGAGATGA	GGGTCCTGTCCATAGTCCCA
ZNF727	TGGGAAATGCGAGTGCTAAC	AGCTGGGTGTTTGGCTACTG
ARX	GTGCAAGGCTCCCCTAAGAG	CGTTCTCGCGGTACGACTT
ZNF716	AAGGCGAGCGTTCAGAGTAA	CGATAGAGTCAATGGCAGCTT
L3MBTL4	TGATCCCCGACATCCATCG	GGCCTTCAAGTAATCCATCCAA
NHLH2	TTCATCCGCCACAAATCTTA	TTCTGTGCCTTCCATCTCTATG
NKX6-1	AGGGCTCGTTTGGCCTATTC	AGAGGCTTATTGTAGTCGTCGT
TBXT	TATGAGCCTCGAATCCACATAGT	CCTCGTTCTGATAAGCAGTCAC
β-actin	AGAGCCTCGCCTTTGCCGATCC	CTGGGCCTCGTCGCCCACATA
HIF-1α	GAACGTCGAAAAGAAAAGTCTCG	CCTTATCAAGATGCGAACTCACA
SLC2A1	GGCCAAGAGTGTGCTAAAGAA	ACAGCGTTGATGCCAGACAG
SLC2A2	GCTGCTCAACTAATCACCATGC	TGGTCCCAATTTTGAAAACCCC
SLC2A3	GCTGGGCATCGTTGTTGGA	GCACTTTGTAGGATAGCAGGAAG
HK1	GCTCTCCGATGAAACTCTCATAG	GGACCTTACGAATGTTGGCAA
HK2	TGCCACCAGACTAAACTAGACG	CCCGTGCCCACAATGAGAC
GPI	CAAGGACCGCTTCAACCACTT	CCAGGATGGGTGTGTTTGACC
PFKL	GTACCTGGCGCTGGTATCTG	CCTCTCACACATGAAGTTCTCC
PFKM	GGTGCCCGTGTCTTCTTTGT	AAGCATCATCGAAACGCTCTC
PFKP	CGCCTACCTCAACGTGGTG	ACCTCCAGAACGAAGGTCCTC
PFKFB2	AGTCCTACGACTTCTTTCGGC	TCTCCTCAGTGAGATACGCCT
PFKFB3	TTGGCGTCCCCACAAAAGT	AGTTGTAGGAGCTGTACTGCTT
PFKFB4	CAACATCGTGCAAGTGAAACTG	GACTCGTAGGAGTTCTCATAGCA
ALDOA	ATGCCCTACCAATATCCAGCA	GCTCCCAGTGGACTCATCTG
ALDOC	ATGCCTCACTCGTACCCAG	TTTCCACCCCAATTTGGCTCA
PGK1	TGGACGTTAAAGGGAAGCGG	GCTCATAAGGACTACCGACTTGG
ENO1	GCCGTGAACGAGAAGTCCTG	ACGCCTGAAGAGACTCGGT
ENO2	AGCCTCTACGGGCATCTATGA	TTCTCAGTCCCATCCAACTCC
ENO3	GGCTGGTTACCCAGACAAGG	TCGTACTTCCCATTGCGATAGAA
PKLR	TCAAGGCCGGGATGAACATTG	CTGAGTGGGGAACCTGCAAAG
**Sequence of siRNAs**		
siRNA	Forward	Reverse

si-NC	UUCUCCGAACGUGUCACGUTT	ACGUGACACGUUCGGAGAATT
si-CBX2	GCUGGUCCUCCAAACAUAATT	UUAUGUUUGGAGGACCAGCTT
si-SIM2	AGAUCAAGCUGUACAGUAATT	UUACUGUACAGCUUGAUCUTT
si-ZNF716	GAUUCAUACUGGAGAGAAATT	UUUCUCUCCAGUAUGAAUCTT
si-ZNF727	GUGCAAAGAAUGUGGCAAATT	UUUGCCACAUUCUUUGCACTT
si-ARX	CGGAGGAGCCCGUGCAAAATT	UUUUGCACGGGCUCCUCCGTT
si-L3MBTL4	GUUGAUGACCAAAGAGUAATT	UUACUCUUUGGUCAUCAACTT
si-NHLH2	GCGUGGAAGCCUUCAACUUTT	AAGUUGAAGGCUUCCACGCTT
si-TBXT	GGAAAGAAGUGAUCACAAATT	UUUGUGAUCACUUCUUUCCTT
si-NKX6.1	ACAAAGACGGGAAGAGAAATT	UUUCUCUUCCCGUCUUUGUTT
si-ZFP57	GAAAGAGCUUCGAGAACAATT	UUGUUCUCGAAGCUCUUUCTT
si-NEUROD1	GCAACUUCUCUUUCAAACATT	UGUUUGAAAGAGAAGUUGCTT
shNC	GATCCGTTCTCCGAACGTGTCACGTAATTCAAGAGATTACGTGACACGTTCGGAGAATTTTTTC	
shHGDILnc1 #1	GATCCGCTGGAATGTGGGCCTACCATTCATTTCAAGAGAATGAATGGTAGGCCCACATTCCAGCTTTTTTG	
shHGDILnc1 #2	GATCCGATACTAACTCTGACAGCATCTTGATTCAAGAGATCAAGATGCTGTCAGAGTTAGTATCTTTTTTG	
si-NC	UUCUCCGAACGUGUCACGUTT	ACGUGACACGUUCGGAGAATT
si-ENO1 #1	CCAGACCCAUUAAGUAUATT	AUAUACUUAAUGGGUCUGGTT
si-ENO1 #2	GCUGCAGGUGUGUCAUUUATT	UAAAUGACACACCUGCAGCTT
si-ALDOC #1	GCCUGUCCCAUCAAGUAUATT	UAUACUUGAUGGGACAGGCTT
si-ALDOC #2	CUACCAGAAAGAUGAUAAUTT	AUUAUCAUCUUUCUGGUAGTT
**Sequence of primers used for ChIP-PCR**		
HGDILnc1 P1	TGAAGCAACACACCAAGCACC	ACTCTAGCGGACCATGCTGT
HGDILnc1 P2	AGGCAAAAAGACGATGGTGTCA	CACATGTGTAATCCATGAGGCAAGA
HGDILnc1 P3	GCTCATAGCACGACAAAGTTGC	TCAAAATGATTAGAAAATAAATGGCT
HGDILnc1 P4	ACTGAGGTTCCCAGACTGGC	GCTGGGCAGTTCTCTACTTTTCC
HGDILnc1 P5	GCATGCCATAGATACAGATTTCGGT	GTGAGAAGAGTTGCTCAGTTGTTCT
HGDILnc1 P6	AGCTACTTGCCTTGCCCTCA	TCAAGCTGGAATATACTTGACCGTCA
HGDILnc1 P7	AAATGAGTCATAAAGGTGCAGTGCA	ATTCTCCAAACATCCCGCACG
P53 Promoter	TGAAAGCACTGTGTTCCTTAG	AACTCCCAGCAGCCACGAGGA
ALDOC promoter	GGGTGAGGACAGAGAAGCTCA	GGCCATGACCTTGCGTTCAT
ALDOC promoter for RNA POLL II binding	GAGGGCAGTCCCTAACAGC	GTAAATGAGGCTGCGGATGT
**Sequence of primers used for HGDILnc1 RACE**		
5'-RACE R1	CAGGTGAGAAAACGGAGGCATAGAGCAT	
5'-RACE R2	GGAGGGTTGAGCCAGGTTTTGGGT	

3'-RACE F1	CCCTAGCATTCTAGGACAGCACCATATAAG	
3'-RACE F2	GACAGGCTGAGTTTGAAGAACAACTC	
mMSTRG.6431-F	GGAATGTGGGCCTACCATT	
mMSTRG.6431-R	CTGATGTGAGAAGGAGCTGAA	

Table S8. Antibodies used in the study

Antibodies	Source	Identifier
CD31	Abcam	ab76533
ENO1	Proteintech	11204-1-AP
NeuroD1	Cell Signaling Technology	4373S
ALDOC	Proteintech	14884-1-AP
HIF1a	Proteintech	20960-1-AP
SLC2A1	Abcam	ab115730
H2B	Abcam	ab1790
FLAG-tag	MBL	M185-3
MYC-tag	Cell Signaling Technology	2365
H2BK5ac	Cell Signaling Technology	12799
H2BK12ac	Active Motif	39669
H2BK15ac	Active Motif	61322
H2BK16ac	Active Motif	39122
H2BK20ac	Active Motif	ab177430
RNA POL II	Active Motif	39497
SUMO1	Abcam	ab133352
SUMO2/3	Abcam	ab81371
Ubiquitin	Cell Signaling Technology	3936
HK2	Santa cruz biotechnology	sc-374169
GPI	Santa cruz biotechnology	sc-271459
PFKL	Santa cruz biotechnology	sc-393713
PFKP	Proteintech	13389-1-AP
PFKFB3	Proteintech	13763-1-AP
ALDOA	Santa cruz biotechnology	sc-390733
PGK1	Santa cruz biotechnology	sc-130335
ENO2	Proteintech	10149-1-AP
ENO3	Santa cruz biotechnology	sc-100811
PKLR	Santa cruz biotechnology	sc-133222
β-actin	KangChen	KC-5A08
