# Supplementary material for: Hypoxia and low-glucose environments co-induced HGDILnc1 promote glycolysis and angiogenesis
Source: Cell Death Discov. 2024 Mar 12;10:132. doi: 10.1038/s41420-024-01903-w (PMC10933424; doi:10.1038/s41420-024-01903-w)

Fig S1C

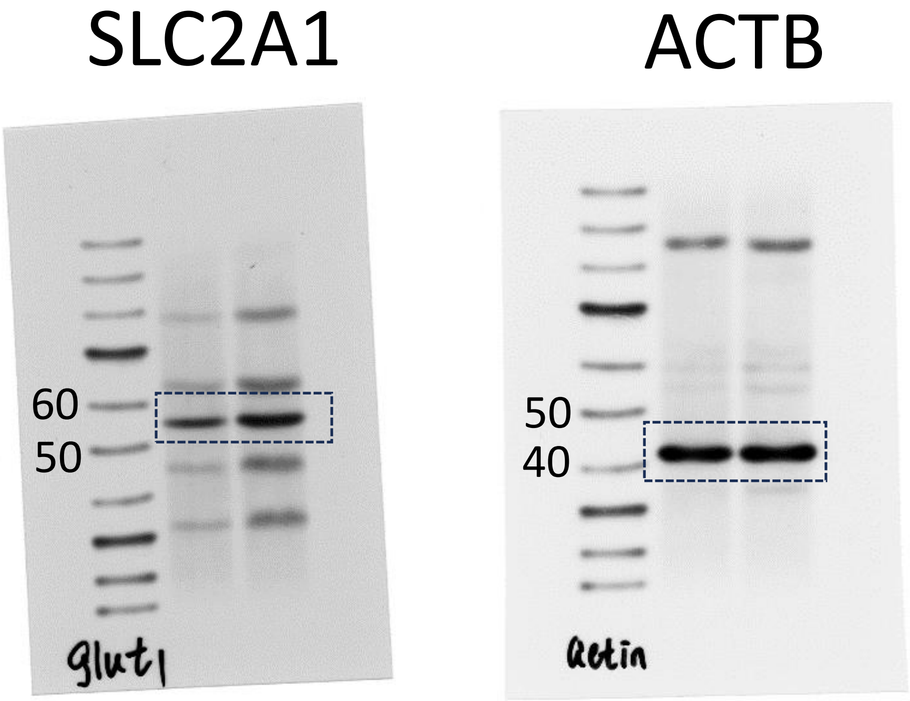

Fig S1D

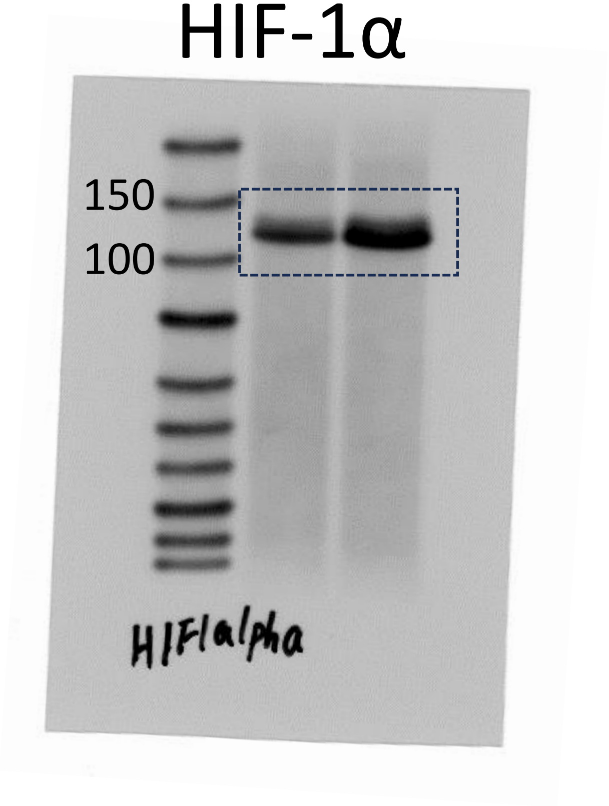

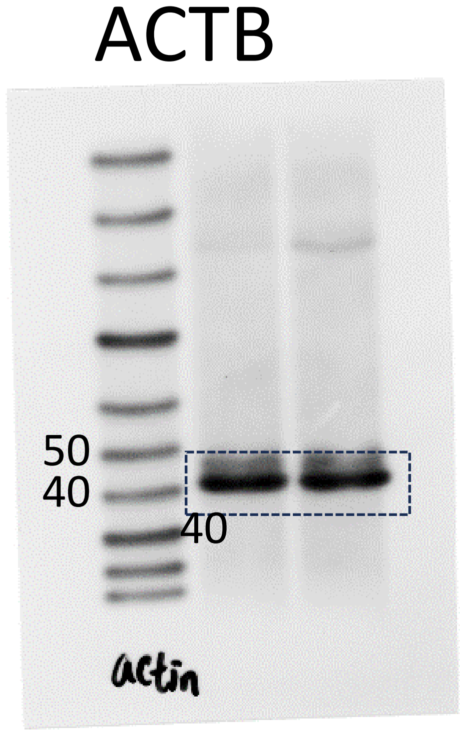

Fig 3C

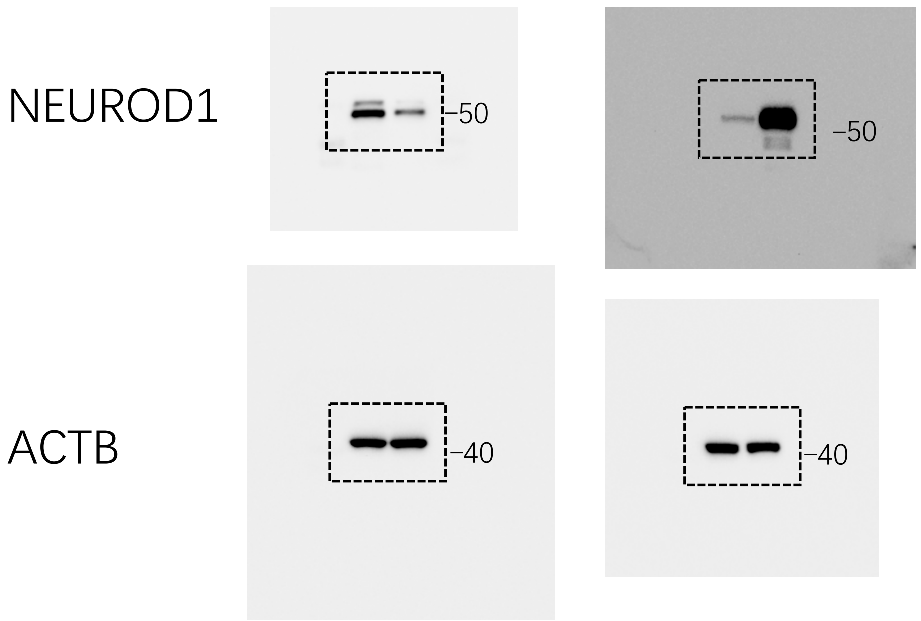

Fig 3H and Fig S3D

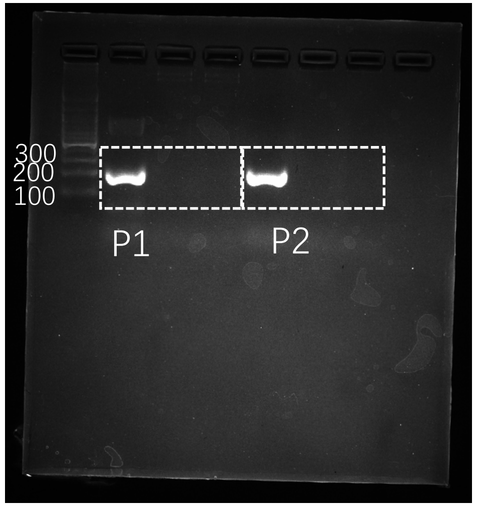

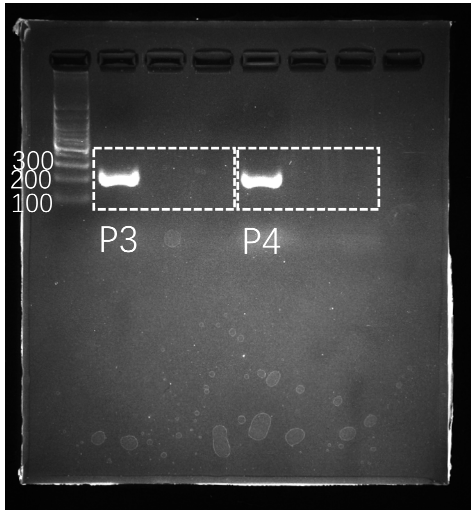

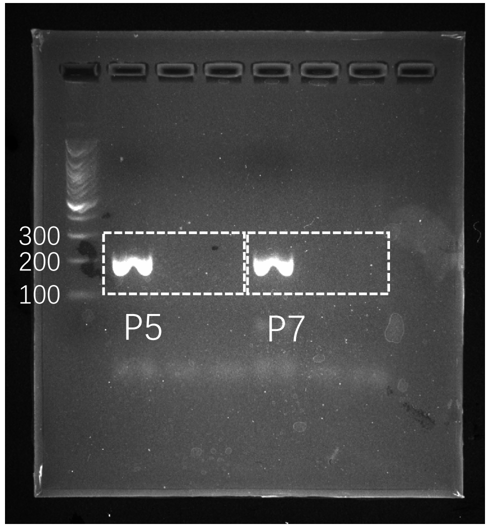

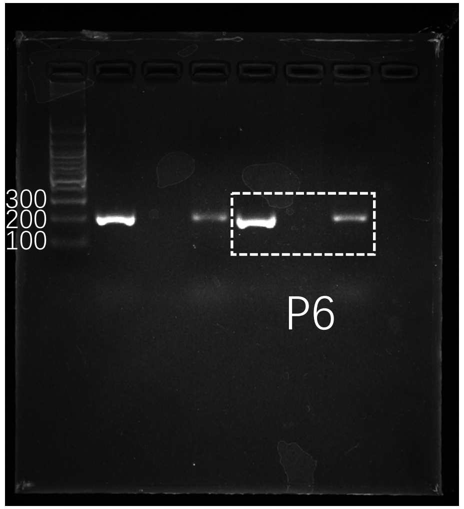

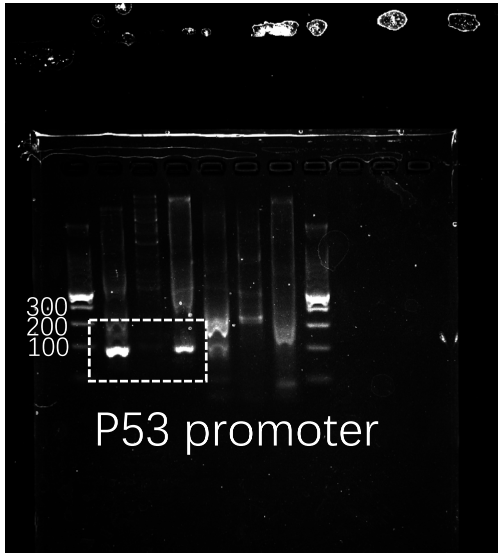

Fig 4B

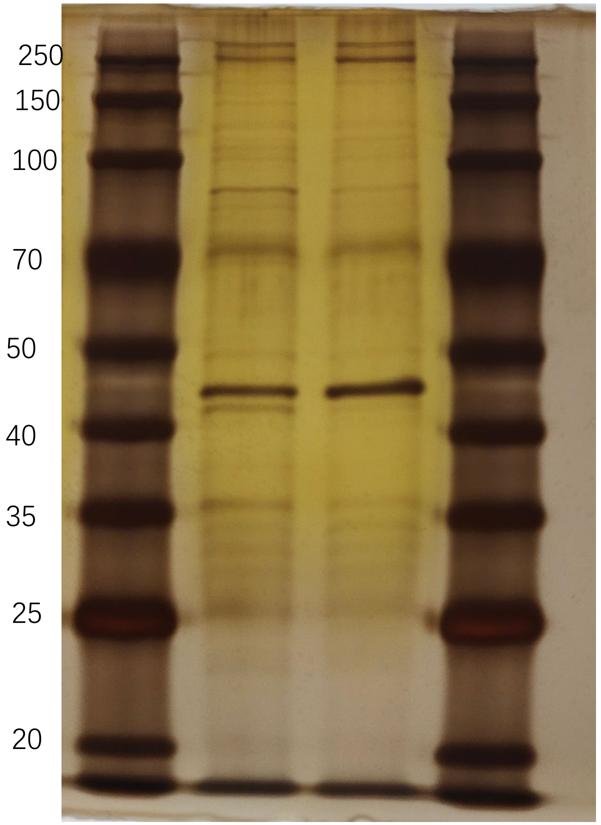

Fig 4I

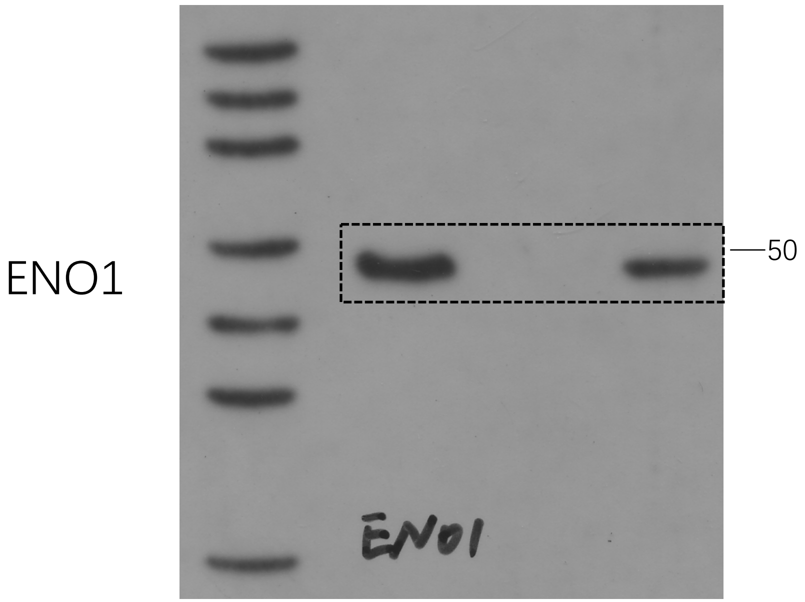

Fig 4L

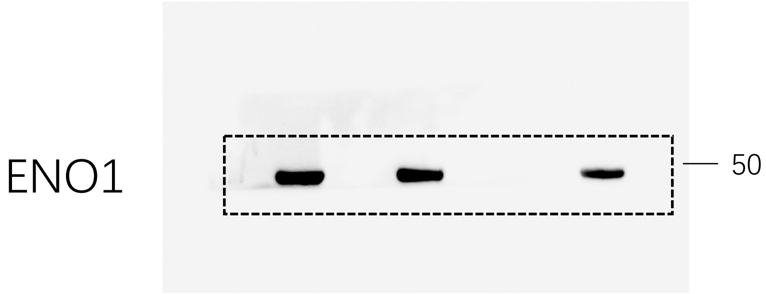

Fig 5A

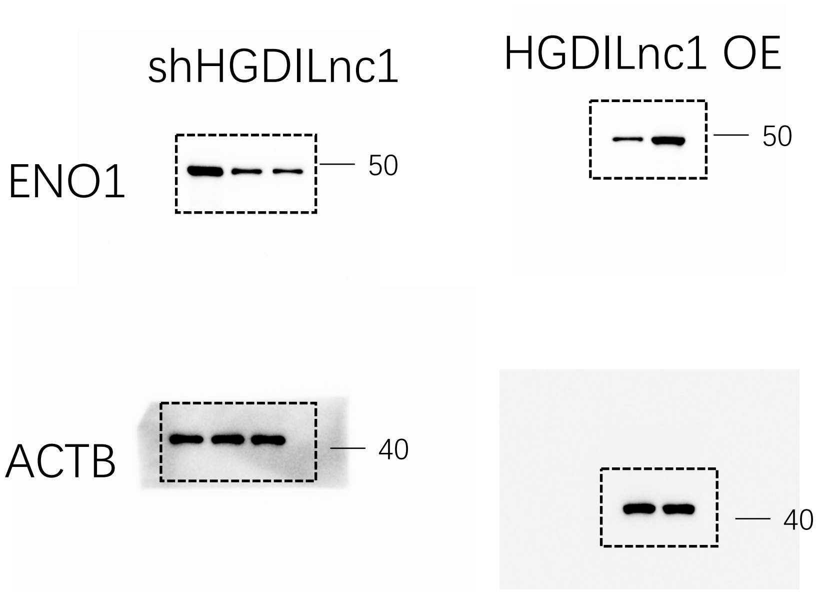

Fig 5C

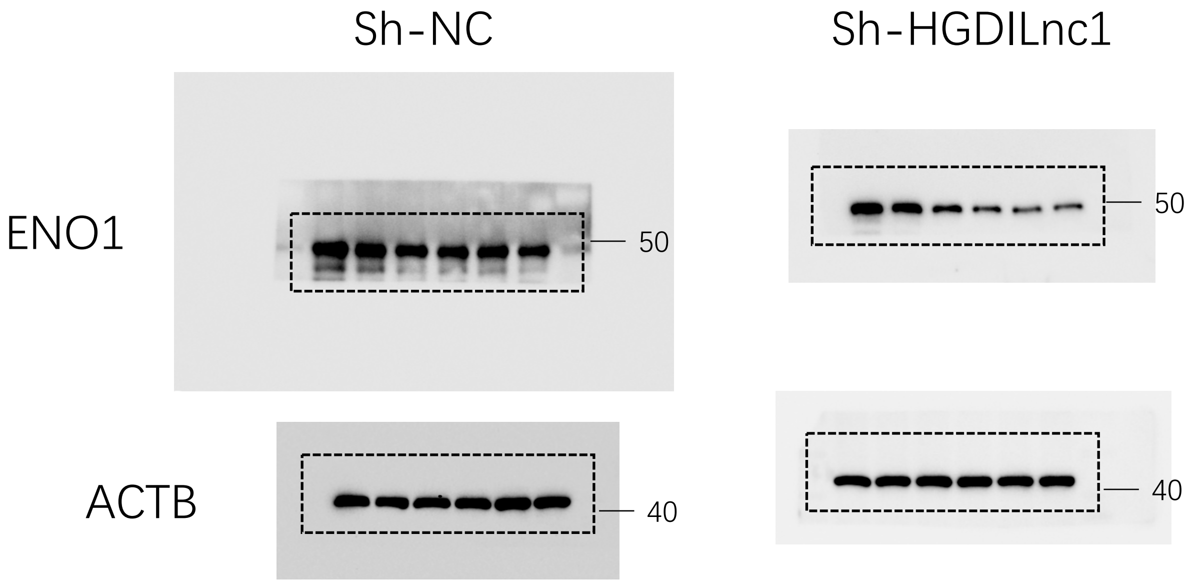

Fig 5D

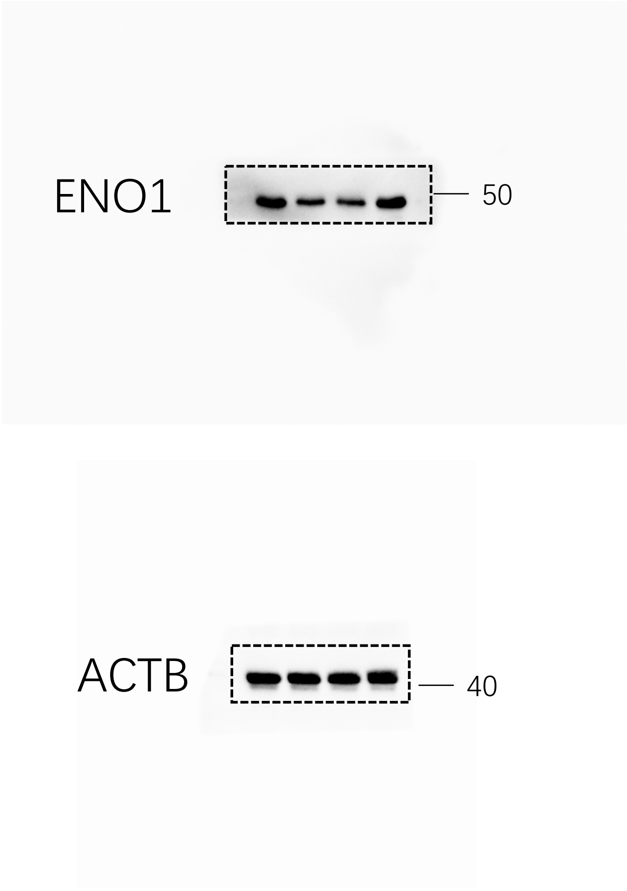

Fig 5E

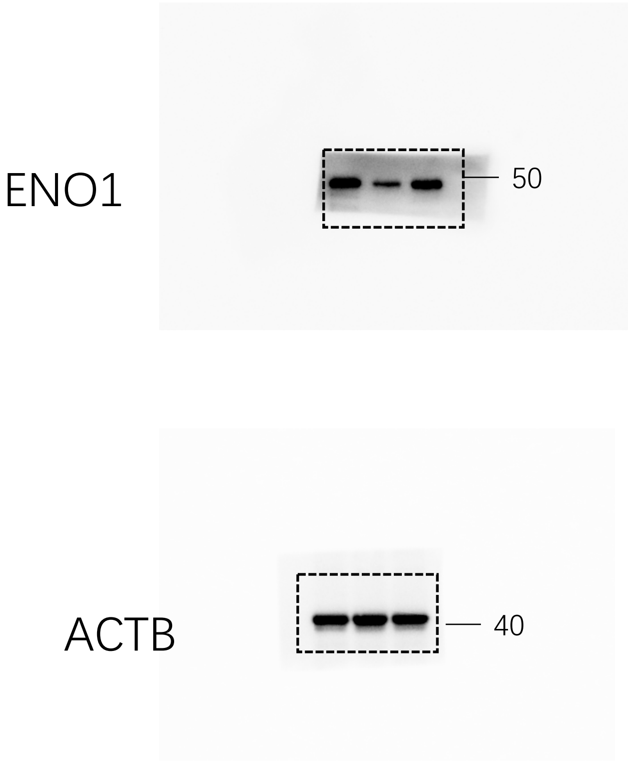

Fig 5F

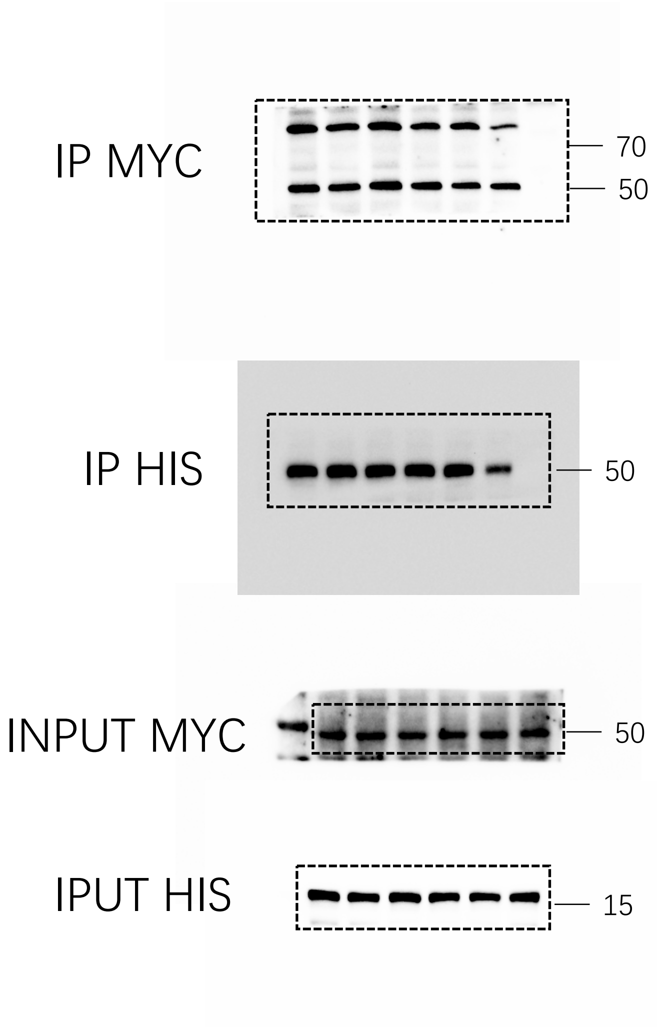

Fig 5G

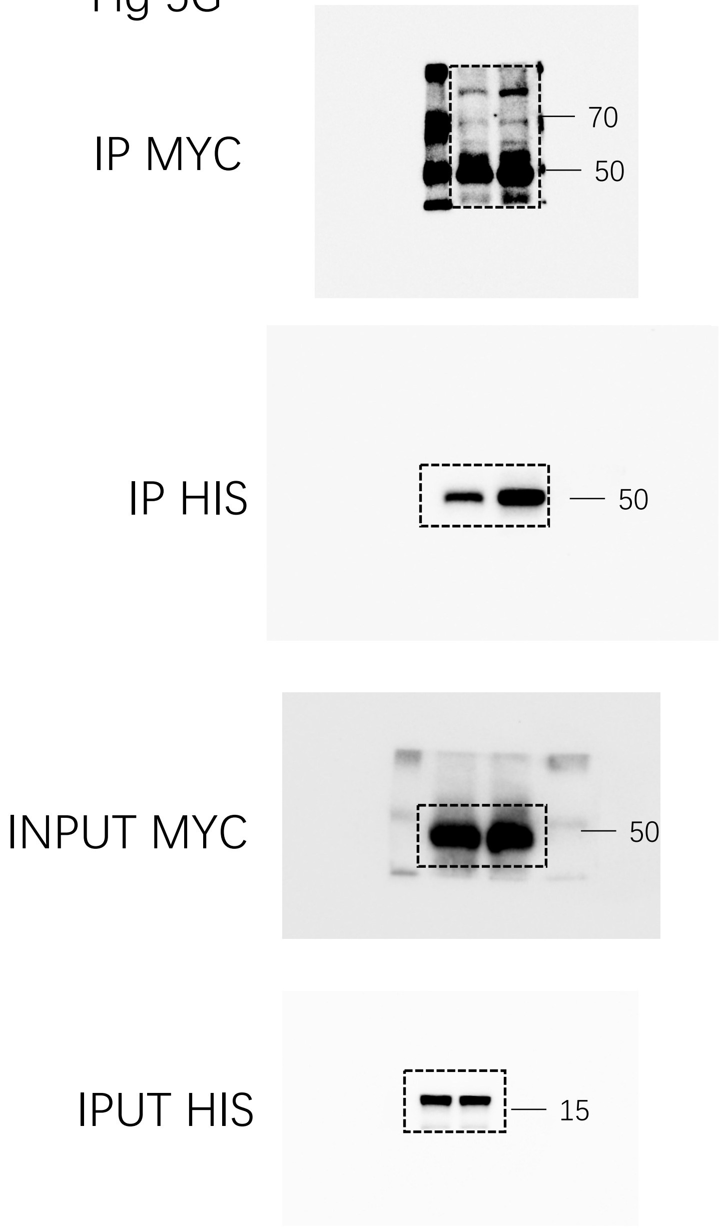

Fig 5H

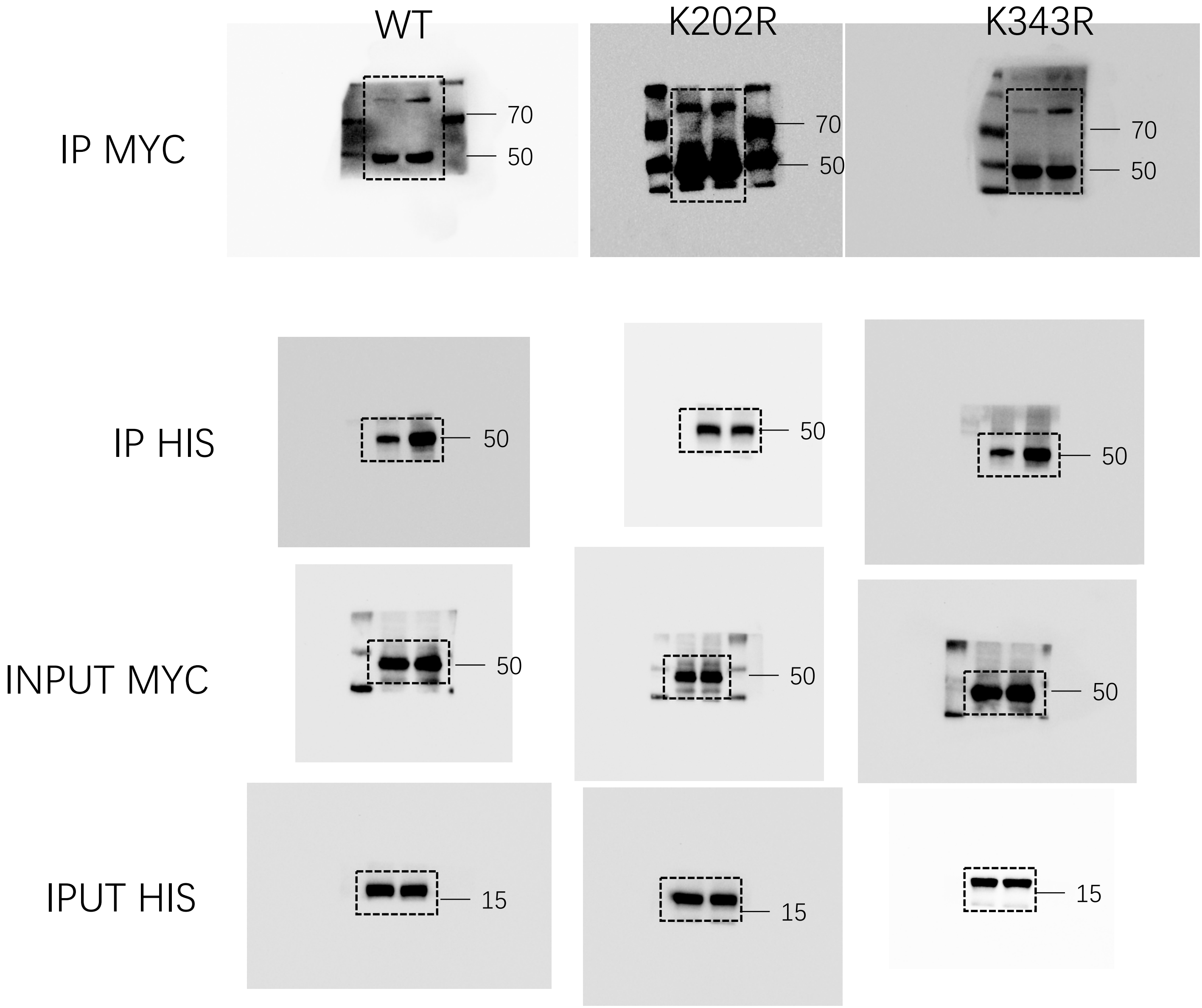
WT
K202R
K343R
IP MYC
70
50
70
50
70
50
IP HIS
50
50
50
INPUT MYC
50
50
50
IPUT HIS
15
15
15

Fig 5I

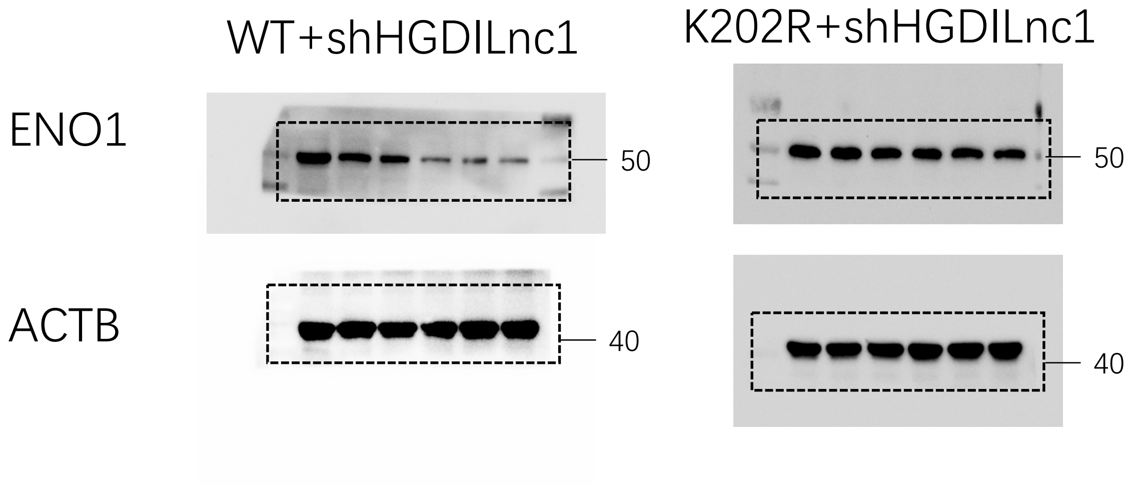

Fig 5J

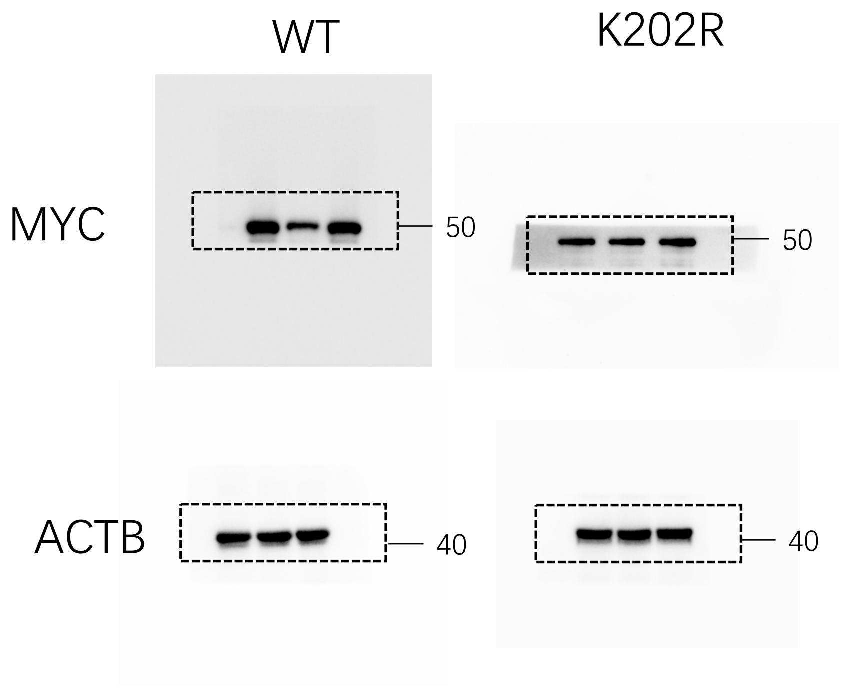

Fig 5K

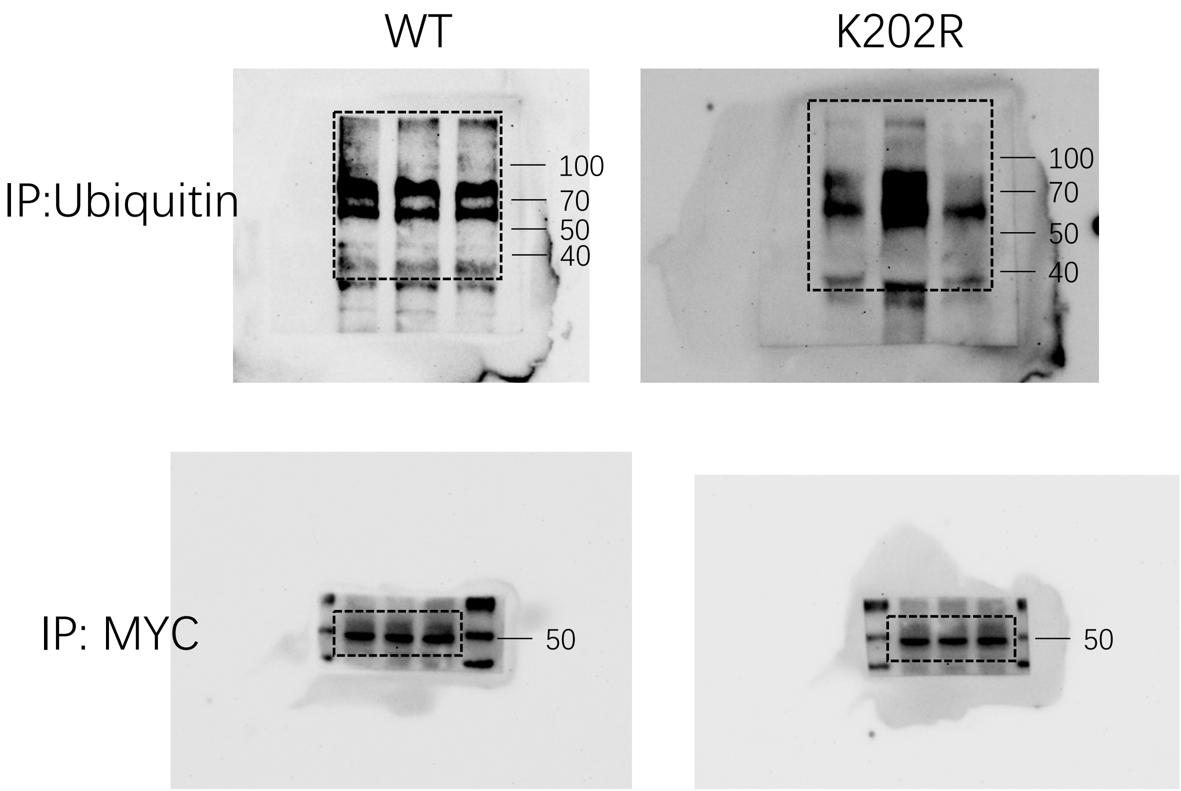

Fig S5C

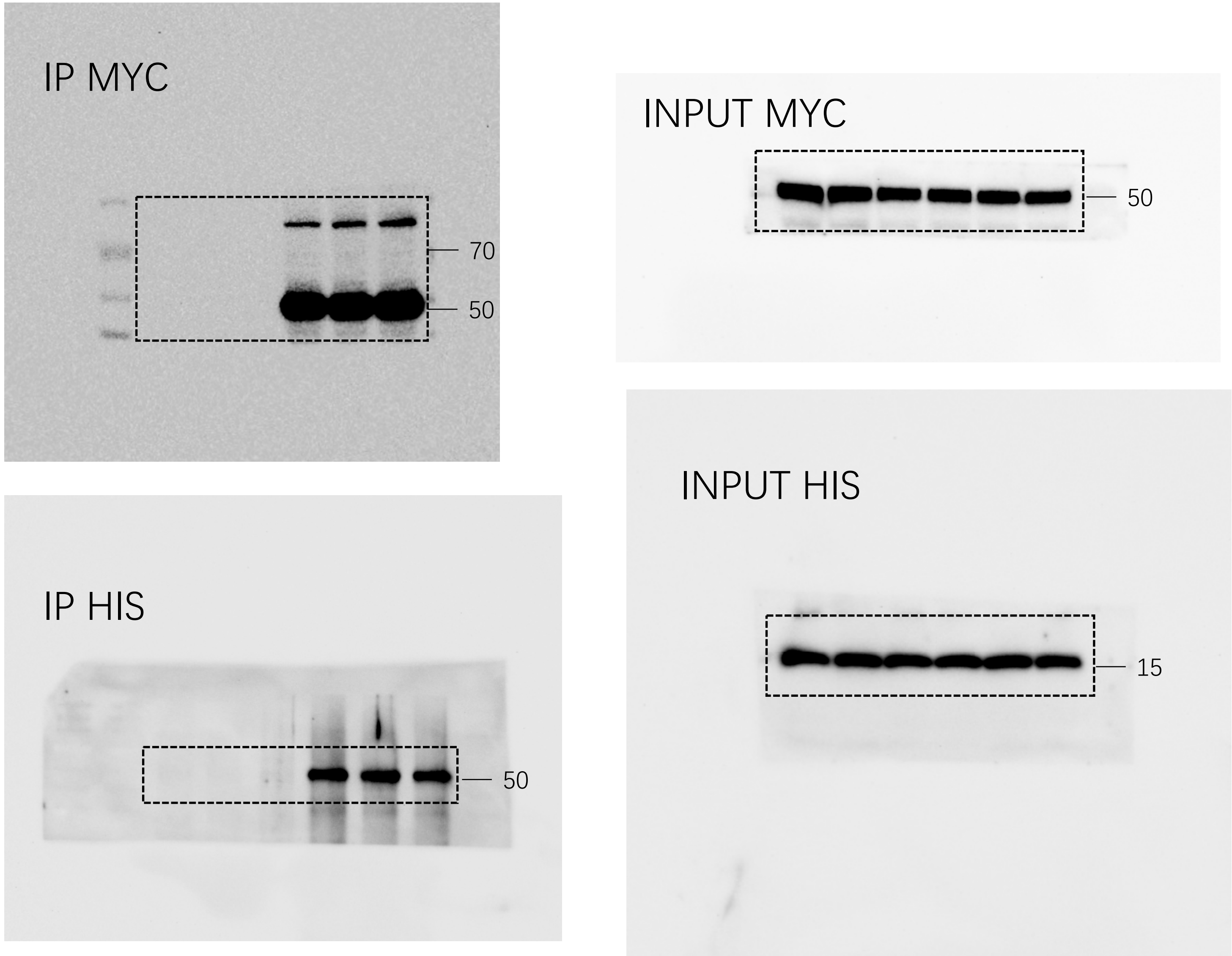

Fig 6C

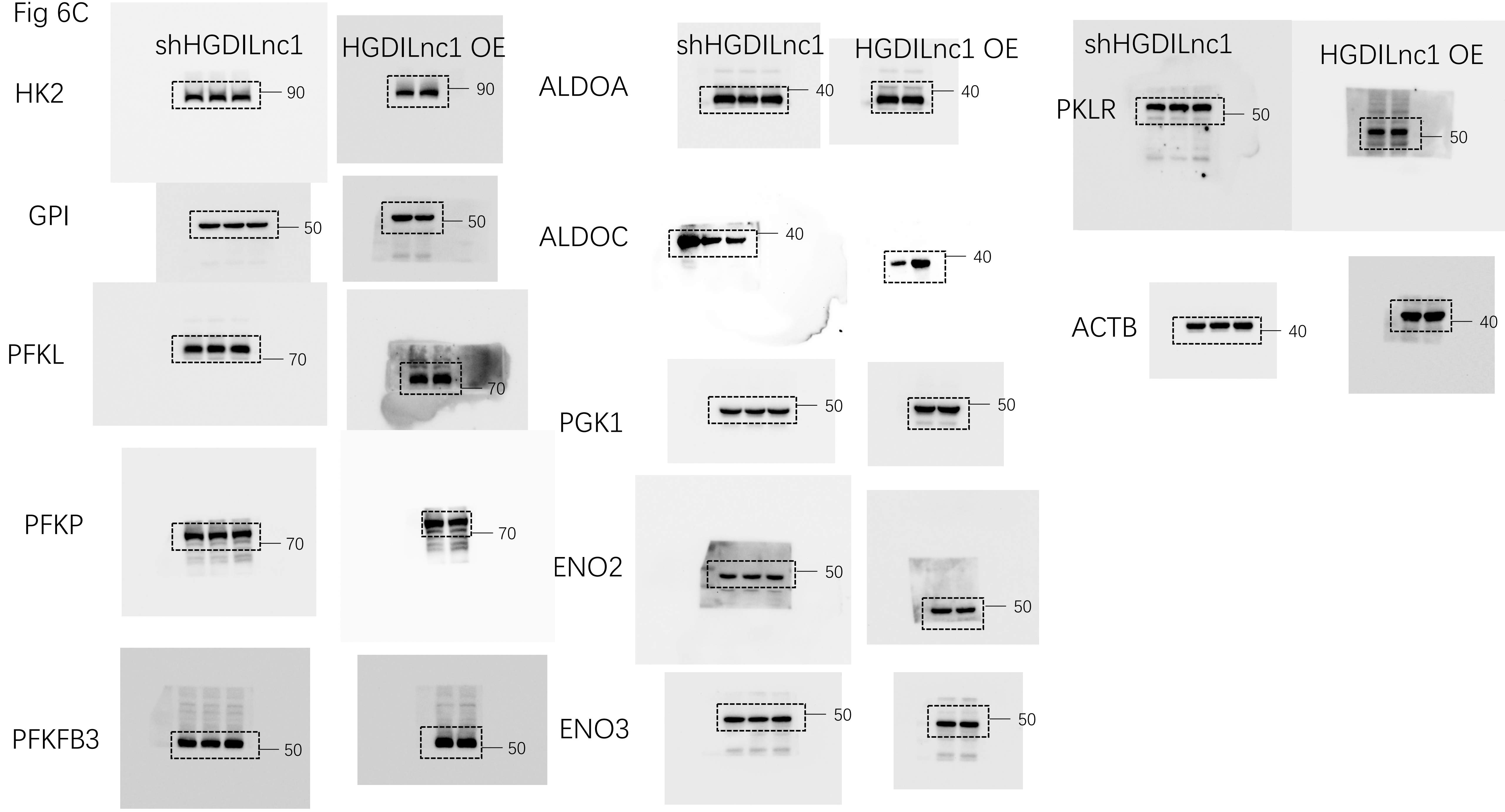

shHGDILnc1
HGDILnc1 OE
HK2
90
90
GPI
50
50
PFKL
70
70
PFKP
70
70
PFKFB3
50
50
shHGDILnc1
HGDILnc1 OE
ALDOA
40
40
ALDOC
40
40
PGK1
50
50
ENO2
50
50
ENO3
50
50
shHGDILnc1
HGDILnc1 OE
PKLR
50
50
ACTB
40
40

Fig 6D

H2B

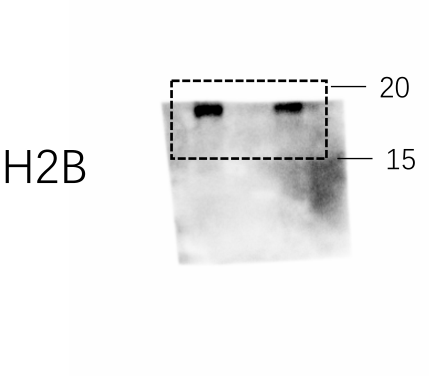

Fig 6G

H2BK16ac

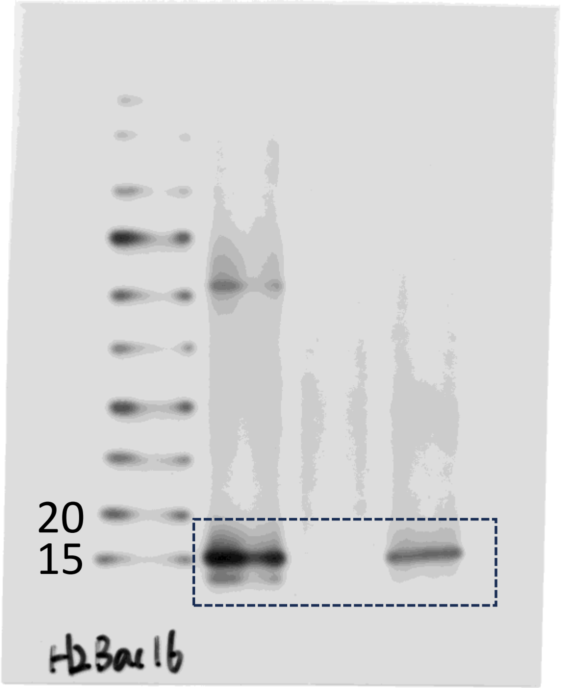

Fig S6J

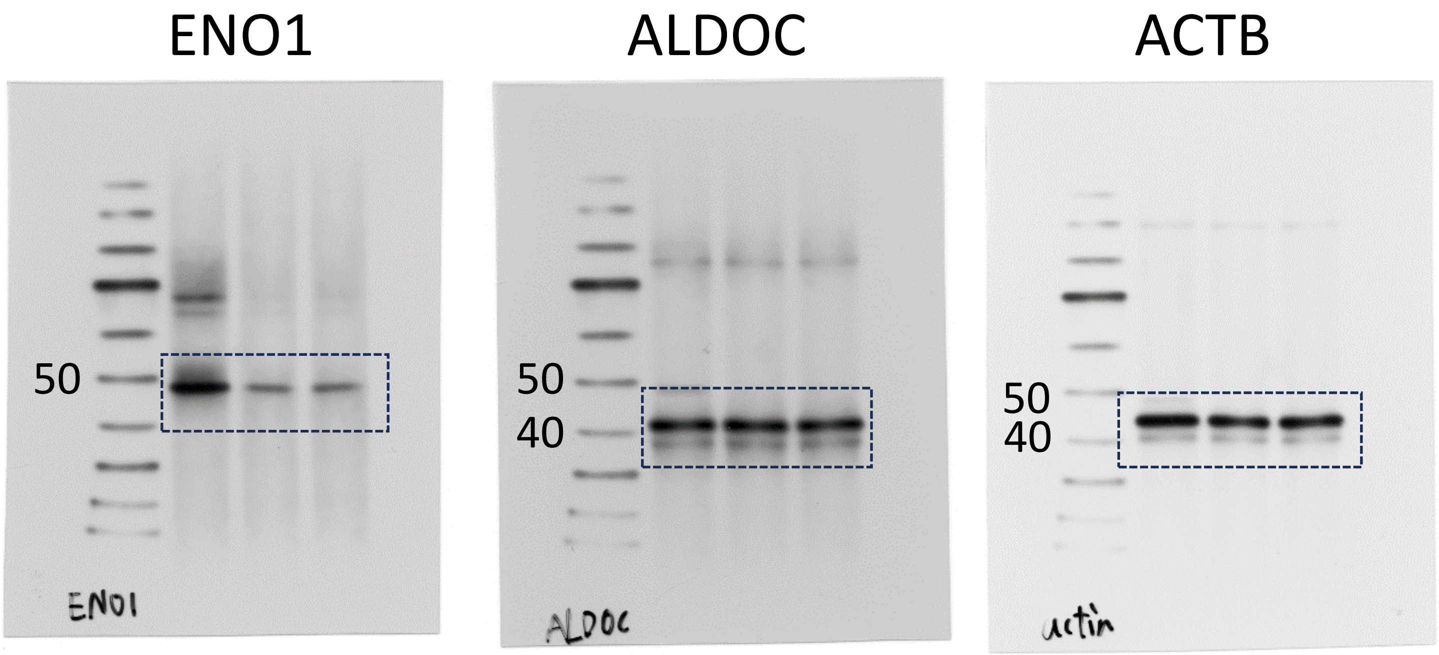

Fig S6K

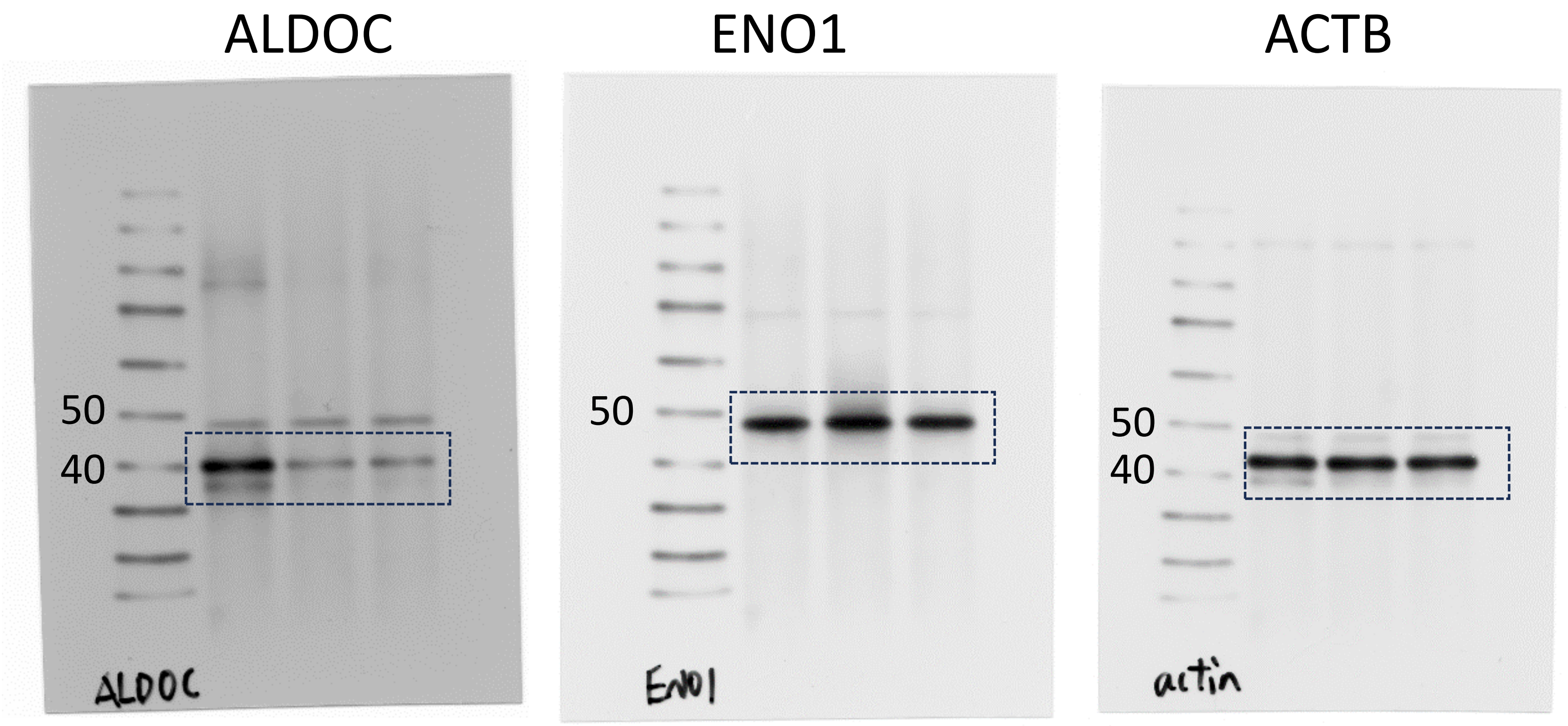

Supplement: Supplementary file 2 — Full and uncropped western blots [file 41420_2024_1903_MOESM2_ESM.pdf]
